# Disrupting hate: The effect of deplatforming hate organizations on their online audience

**DOI:** 10.1073/pnas.2214080120

**Published:** 2023-06-05

**Authors:** Daniel Robert Thomas, Laila A. Wahedi

**Affiliations:** ^a^Meta, Menlo Park, CA 94025

**Keywords:** hate speech, deplatforming, social networks, leadership removal

## Abstract

How does removing the leadership of online hate organizations from online platforms change behavior in their target audience? We study the effects of six network disruptions of designated and banned hate-based organizations on Facebook, in which known members of the organizations were removed from the platform, by examining the online engagements of the audience of the organization. Using a differences-in-differences approach, we show that on average the network disruptions reduced the consumption and production of hateful content, along with engagement within the network among periphery members. Members of the audience closest to the core members exhibit signs of backlash in the short term, but reduce their engagement within the network and with hateful content over time. The results suggest that strategies of targeted removals, such as leadership removal and network degradation efforts, can reduce the ability of hate organizations to successfully operate online.

## Significance

How does removing the leadership of online hate organizations from online platforms change behavior in their target audience? We study the effects of six network disruptions of designated and banned hate-based organizations on Facebook, in which known members of the organizations were removed from the platform, by examining the online engagements of the audience of the organization. We show that on average, the network disruptions reduced the consumption and production of hateful content, along with engagement within the network among audience members. The results suggest that strategies of targeted removals, such as leadership removal and network degradation efforts, can reduce the ability of hate organizations to successfully operate online.

Hate-based, terrorist, and criminal organizations attempt to use online platforms to spread their ideology, recruit new members, and coordinate existing members (1).^*^ They attempt to leverage online networks because of their ability to spread information quickly and widely. To counter this, social media platforms reduce the ability of such organizations to organize on their platforms, often by removing the members, or “deplatforming” them.^†^ To what extent does deplatforming successfully mitigate harm? To answer this question, we employ a differences-in-differences design, taking advantage of the staggered timing of six exogenous organization-level deplatforming events, and granular daily user-level observations.^‡^ We show a strong overall negative effect: Deplatforming reduces hate on the platform.

We study the effects of “strategic network disruptions” (SNDs), a method of deplatforming in which identifiable core members of a hate-based organization are removed from the platform all at once, eliminating the online leadership of the organization. The goal of this approach is to disrupt the operation of the organization by removing key actors at the same time in order to make it more difficult for them to rebuild their audiences and regroup. If the approach is successful, then the organization’s online target audience should be exposed to less radicalizing content and hate, and decrease their production of it. We study the effects of the disruptions on this target audience.

We find that disruptions create a healthier platform on average: Members of the audience of the hate-based organizations reduce their consumption and production of hateful content, and engage less with other audience members. However, these average results mask substantial heterogeneity between audience members who engaged most with the core prior to disruption, and those least engaged. While the least engaged subgroup reduces its engagement with hateful content and with other audience members following the disruptions, the most engaged subgroup exhibits signs of backlash, increasing its consumption and production of hateful content,^§^ and engaging more with other audience members. However, the backlash is short-lived. Within two months, even the subgroup closest to the organization reduces its engagement with hateful content and with the rest of the network.

Our results paint an optimistic picture: SNDs improve the quality of content created and consumed by the subgroup of the audience most at risk for influence—those least close to the organization. While disruptions appear to inflame a portion of the audience in the short term, this subgroup was closest to the organization, and therefore likely to already be under their influence. Moreover, any backlash appears to reverse in the long term. This suggests that disrupting the leadership of a problematic organization can reduce their ability to influence their targets and grow the organization. Efforts to reduce the influence of hate organizations and inhibit hate speech online can impact the offline behaviors of both the perpetrators of hate and their target communities (2). Perpetrators of hate crimes acknowledge the influence of online communities (3, 4) and use of internet and social media is associated with greater offline hate crime (5, 7). Exposure to hate speech creates fear and trauma in targets (8, 9), as well as reducing their civic engagement and participation in public debate (10).

Our study contributes to a growing literature on the effects of deplatforming on the health of online platforms. Past research on Twitter, Reddit, and Telegram has shown that suspending or removing users or communities can reduce the use of hate speech and the size of illicit communities (11–14). We build on these studies by offering a credible identification strategy and granular data. We show that deplatforming has a causal effect on platform health, and moreover that the removal of only several hundred accounts can have a large impact on the behavior of their audience. In terms of approaches to reducing hate speech online (15, 16), we show that network disruptions can on average decrease hate speech in heavily ideological networks.

We also build on literature on the effects of offline leadership removal on the degradation of institutional structures that enable active participation of members (17, 18). We show that removing the core members of hate-based networks can lead to the degradation of the overall network, suggesting that targeted leader removals can be effective in online contexts.

## Conceptual Framework

Like any ideological organization, hate organizations use social media platforms with broad user bases to organize themselves, maintain engagement and interest among members and supporters, recruit and gain sympathy, and shift public opinion in support of their social goals (1). Recruiting, gaining sympathizers, and shifting public opinion all involve propagandizing to broader populations than their support base itself.

We argue that network disruptions on these platforms should be uniquely able to disrupt hate organizations for two reasons. First, removing the organization leaders all at once prevents organizations from reconstructing their networks: Disruptions make it harder for returning members to find one another again because there are no remaining accounts to help coordinate those returning to the platform. Organizations must also change their language and behavior on the platform if they return, making it more difficult to find other returning members and recreate the organization’s network. Second, disruptions cut off hate organizations from their broader target audience, making it harder for the organization to reach its sympathizers to direct them to alternative communication mediums. While the most dedicated members and sympathizers might seek out the organization, the targets of recruitment and public opinion propaganda can lose all contact. The result is that the hate organization can no longer shape the behavior of its target audience.

However, for network disruptions to successfully prevent hate organizations from reaching their target audiences—and therefore create healthier platforms—they must overcome several challenges. First, while disruptions remove the entirety of the identifiable leadership of hate organizations, these users may be able to return to the platform by creating new accounts and reconstituting their network. We refer to this as return. Second, the disruptions may not prevent potential sympathizers from engaging with the organization through other means outside of the platform leading them to engage in similar hateful behavior after the disruptions (19). We refer to this as Reach Through Alternative Media. Finally, the audience of these organizations may seek out alternative sources of hate on the platform, substituting engagement with the removed organization with engagement with outside, similar content. Alternatively, a competing hate organization may be able to replace their position and co-opt their audience. Thus, potential sympathizers would be exposed to similar content, even after the removals. We refer to this as Push vs Pull factors. These three factors suggest the following five observable implications, which we empirically evaluate.

### Return.

If the network disruptions successfully prevent the organizations from reaching their target audience, we expect to see a decrease in the consumption of hateful content, as this audience would no longer be exposed to the organization’s propagandizing. Conversely, if the organization were able to quickly return to the platform, we would expect to see no change in the hate consumed by the target audience.

**H1: Consumption of hateful content by members of the target audience should decrease.**

Moreover, if the disruptions are successful, then the network surrounding the removed accounts should degrade, as the organization can no longer organize its target audience. However, if deplatforming were unsuccessful, and hate actors were able to return and reconstitute their networks, then we would expect the communities surrounding them to remain intact.

**H2: The audience network should engage less with itself.**

### Reach through Alternative Media.

If the network disruptions are successful, hate organizations should no longer have effects on the behavior and ideology of their audience. Hate organizations affect the behavior of their audience by normalizing the use of hateful rhetoric, leading audience members to produce such content themselves (20–22). If the target audience is no longer being exposed to this content, its behavior should shift to producing less hateful content. Conversely, if hate organizations are able to reconstitute or reach their target audience through other means, norms will not change and thus the behavior of the target audience will not change.

**H3: Production of hateful content by the target audience should decrease.**

### Push vs Pull Factors.

To reduce harm to the target audience, disruptions must make the platform as a whole healthier for the audience, and lead them to encounter less hateful content overall. Since some users seek hate content (pull factors), the reduction in hate consumption should not solely be attributed to the mechanical effect of removing the hateful content posted by organization leaders. If hate consumption were a result of users seeking out hate organizations, then disruptions will only be effective until users can find alternative sources of hate. Accounting for the reduction in available hate content, do users consume less hate postdisruption or do they start to substitute their hate consumption from other sources?

**H4: Consumption of hateful content should decrease, even excluding hate produced by the organization.**

However, the extent to which a hate organization can reach its target audience depends in part on whether the consumer seeks hate content. We should therefore expect to see any effect of the disruption mitigated by how close the user was to the organization. Users who interacted often with the disrupted organization should be more willing to seek the organization or similar organizations out through alternative means, and their behavior should therefore change the least. Conversely, we expect the effect of cutting off less engaged users to be stronger.

**H5: The effects of the disruptions will be weakest for users who engaged the most with the organization, and the strongest for those who engaged the least.**

## Materials and Methods

### Data.

We study the effect of SNDs by examining the effect of the disruptions on the target audience members of four hateful organizations.^¶^ Six separate disruptions took place in a staggered manner during the study period. In each of the disruptions, several hundred core organization members were removed from the platform. *SI Appendix*, Fig. A.1 shows the staggered timing of the disruptions. Users were considered part of the audience if they either viewed more than four pieces of unique content produced by removed members or directly engaged with removed members in the month prior to the disruption. We randomly sampled 10% of users from each of the six audiences. While few users overlap between the different audiences, in the cases when they do we assign them to the earlier disrupted cluster. Our primary dataset is composed of observations at the user-day level, spanning 44 d, such that the last cluster is never treated. In total, our dataset includes daily observations for 26,359 deidentified individuals.^#^

Our analysis is limited to a small number of clusters because only six comparable disruptions took place in our time window. To overcome this limitation, we also conduct our analysis on a second dataset for robustness and to examine long-term effects, in which we include sample control groups based on their distance from audience members in an embedding space before treatment occurred. To generate these sampled control groups, we first take a random sample of all Facebook users, then calculate the cosine similarity between these users and users in the hate org audiences in a general-purpose embedding space trained on user interactions with content (23). This embedding space identifies users who have similar behavior patterns and engage with similar content and topics. We then restrict our population to users who are between 0.8 and 0.9 similarity to capture users with similar behavior and interests, but who were not viewing the same content. From this population, we randomly sample users with similar levels of activity to the audiences, in terms of counts of daily content viewed. We then have six additional never-treated control groups, which allows us to extend the data to a period of 73 d. Summary statistics by disruption are shown in the *SI Appendix*.

### Target Audience Subgroups.

We disaggregate the audiences based on a user’s level of engagement with the removed users in the two weeks prior to the first disruption, measured in terms of their views of these users’ content. We create three levels of audience members: those in the top quartile of views, between greater than 0 views and the top quartile, and those who had 0 views. The breakdown of the subgroups is shown in *SI Appendix*, Table A.2. The subgroups are largely similar within the audiences defined by the disruptions in terms of age, gender, and time on the platform. The subgroups also correspond to friendship with the removed users: Those with the greatest engagement with the removed users are also more likely to be Facebook friends with these users (*SI Appendix*).

### Outcome and Treatment Variables.

Our treatment variable is a binary indicator of whether a user is in an audience of an organization that has been disrupted at time t. We estimate the effect of the disruptions on three sets of outcomes: consumption, creation, and network behavior. Summary statistics for all outcome variables are shown in *SI Appendix*, Table A.4. We introduce the three sets of outcomes here.

The first set is consumption of hateful content. Content is classified as hateful if it exceeded a threshold on a Facebook hate speech classifier.^ǁ^ We analyze the effects on a count of views of hateful content, the ratio of views of hateful content to total content and views of hateful content not produced by removed members. For all ratio variables, observations are missing when the denominator is 0. We also estimate OLS models where the denominator enters as a covariate instead, shown in *SI Appendix*. In *SI Appendix*, we show that our results are robust to a measure of ideologically aligned content as classified by a dictionary-based approach of slurs, ideologically aligned phrases, and organization names collected from external hate databases and expanded by subject matter experts.

The second set is creation of hateful content. In this set, we examine four outcomes: hateful comments, the ratio of hateful comments to total comments, hateful engagements within the audience, and hateful engagements outside of the audience. These engagements encompass posts, comments, reactions, tags, and other types of interactions.

The third set is network behavior: We estimate the effect of the disruptions on users’ inward and outward engagements with other members of their audience, and the ratio of these engagements to total engagements.

### Empirical Approach.

To study the effects of the SNDs, we leverage exogenous variation in the timing of the disruptions to employ a staggered differences-in-differences design to estimate the average treatment effect on the treated (ATT), employing counterfactual estimators (24). The timing of the disruptions was dictated by the ability of investigators to collect and analyze sufficient evidence to identify the members of the online organizations, and was not related to the online behavior of the removed members or members of the audience.^**^ In this approach, we employ either the fixed effect counterfactual estimator or the interactive fixed-effect counterfactual estimator depending on which model returns a lower F-statistic in an equivalence test for pretrends (The comparison of the F-statistics is shown in *SI Appendix*). All models include standard errors clustered at the disruption level and estimated by bootstrapping 500 times.

For each outcome, we display coefficient plots of the ATT for the entire sample and for the subgroups using the dataset without sampled control groups and with data before when the last cluster is disrupted. We also show a plot of the estimated ATT per day for one outcome in each family, with plots for all manuscript outcomes in *SI Appendix*. We then focus on the longer-term effects of ideologically aligned consumption and production in order to test our hypothesis on backlash. For these outcomes, we show the ATT for each outcome with the full sample and by subgroup, comparing the results from the models with sampled control groups, one with data ending 44 d into the panel and the other extending 73 d. For robustness, we show in *SI Appendix* that the results are robust to standard two-way fixed effects estimators, to *P*-values calculated using wild-cluster bootstrapping due to our small number of clusters (25), and to time fixed effects estimators (In the case of time-only fixed effects estimators, results are largely stable except for the backlash effects noted below. However, such effects are found in all other approaches). We also employ dynamic event study estimators (26) with the results shown in *SI Appendix* and discussed below. In *SI Appendix*, we also include plots of an equivalence test to check whether pretreatment ATTs exceed an equivalence range, and results from placebo tests on the last two pretreatment periods (24).^††^

**Fig. 1.**
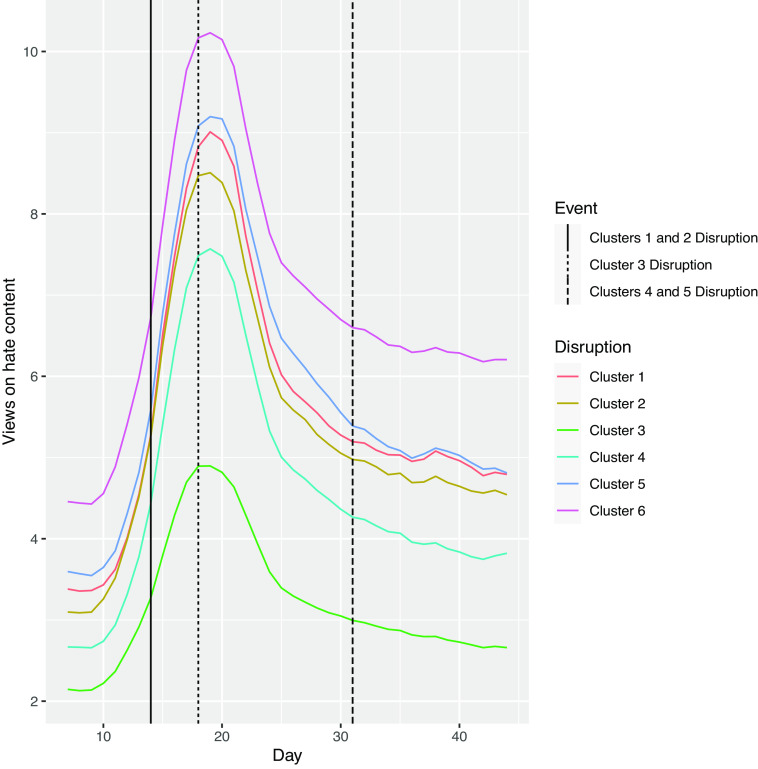
Views on hateful content by organization over time: All six organizations have similar time trends prior to the initial disruptions. The spike in hateful content corresponds with the beginning of the George Floyd protests. Note that it is difficult to discern treatment effects from this descriptive plot because treatment effects are a combination of effects over the postdisruption study period. Figs. 2–5 for magnitude of the treatment effects.

All six organizations have similar trends in views of hateful content in the days prior to the initial disruptions, supporting the parallel trends assumption (Fig. 1). Plots for all other outcomes are available in *SI Appendix*.

## Results

### Consumption.

**Fig. 2.**
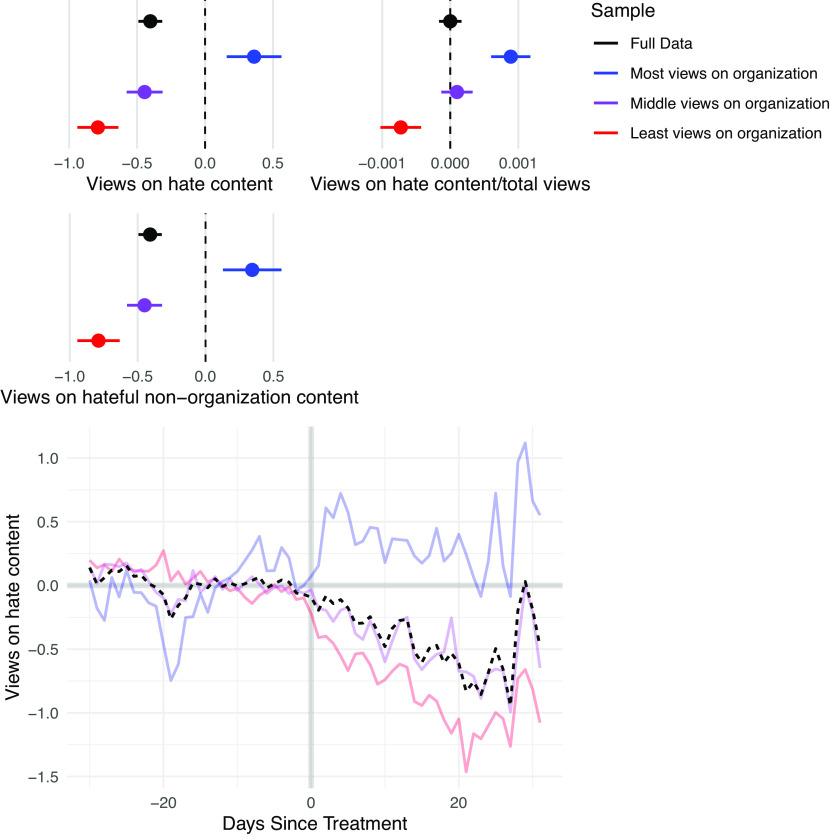
Effect of network disruptions on consumption of hateful content. Points represent the estimates for each model. Lines represent 95% confidence intervals. Beneath each plot is the outcome variable. The samples are defined by predisruption views of organization content, with “Most views on organization” representing the sample that viewed the most content and “Least views on organization” representing the sample that viewed the least content. There is a negative average effect on the consumption of hateful content and nonorganization hateful content, but the closest subgroup increases its consumption of such content.

We find three noteworthy results (Fig. 2). First, on average, users view nearly half a piece of hateful content less per day following the disruptions, a significant decrease given that users viewed 4.9 pieces of hateful content on average per day. Moreover, this is not due to the mechanical effect of removing the removed users’ content: Accounting for content produced by the removed users, users still saw an average decrease in hateful content consumed. The ATT by period plot shows that the decrease is stable overtime, and does not display substantial pretrends.

Second, the average result masks substantial heterogeneity among subgroups of users in different positions in the network. The subgroup that had the lowest levels of engagement with the removed organization prior to disruption received healthier content, consuming less hate, total and relative. Users in the subgroup that engaged most with the organization exhibited a short-term backlash, increasing their total and relative views of hateful content.

Third, we found that on average users reduced their total content consumption, with the majority of the reduction being among users in the subgroup farthest from the disruption, while users in the subgroup exhibiting backlash increased their consumption. However, while the effects of these two groups balanced out on average, this reduction in total consumption did not drive the findings, as users in the subgroup farthest from the disruption still saw a smaller proportion of hate, while those in the subgroup closest to the disruption saw relatively more hate in the short term.^‡‡^

### Production.

**Fig. 3.**
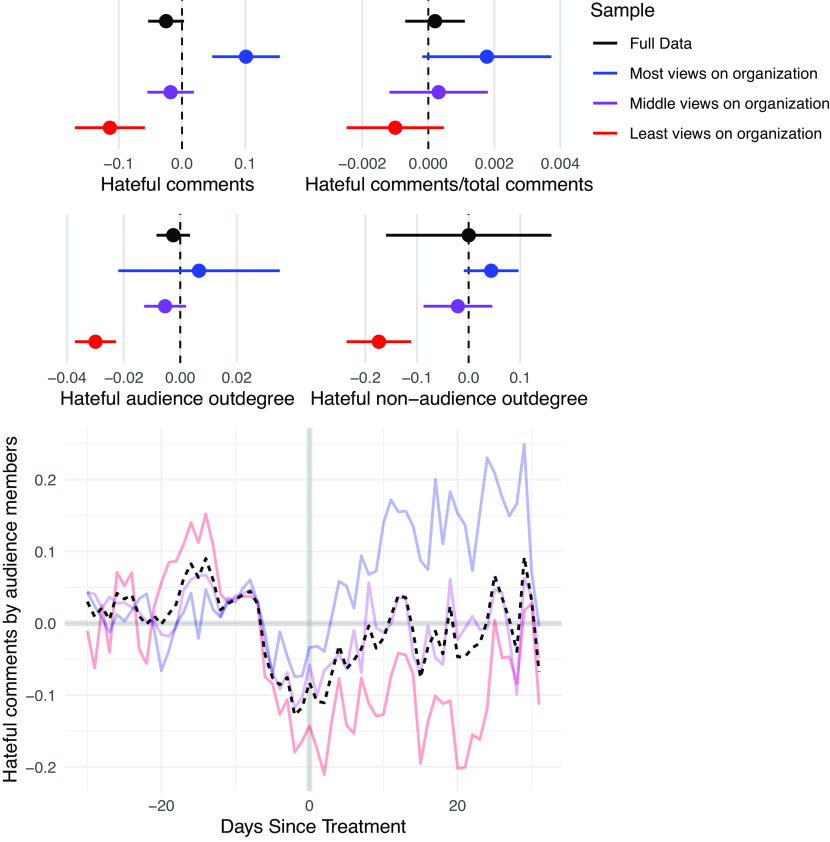
Effect of network disruptions on production of hateful content. Points represent the estimates for each model. Lines represent 95% confidence intervals. Beneath each plot is the outcome variable. The samples are defined by predisruption views of organization content, with “Most views on organization” representing the sample that viewed the most content and “Least views on organization” representing the sample that viewed the least content. There is an average negative effect, but the subgroup that engaged the most with the removed users increases its production of such content, exhibiting signs of backlash.

The results for production show a similar pattern to consumption (Fig. 3): While the average effect is modest or null, the effect is dependent on distance to the removed users. On average, members of the audience produced slightly less hateful content each day, but there was no effect on the ratio of hateful content to total content produced, as users reduced their overall content production. There is no average effect on hateful engagements inside or outside the audience.

However, while the subgroup that engaged less with the organization reduced its production of hateful content and its hateful engagements inside and outside of the audience, the subgroup that engaged the most increased its total and relative production, again exhibiting signs of backlash against the disruption.

### Network Behavior.

**Fig. 4.**
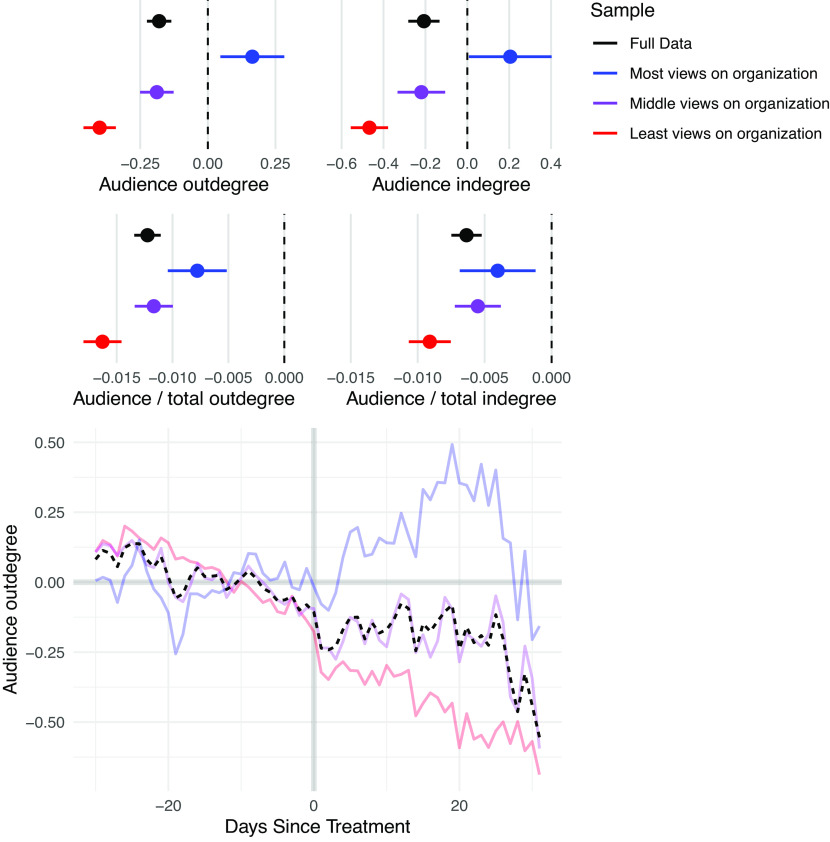
Effect of network disruptions on engagement within the audience. Points represent the estimates for each model. Lines represent 95% confidence intervals. Beneath each plot is the outcome variable. The samples are defined by predisruption views of organization content, with “Most views on organization” representing the sample that viewed the most content and “Least views on organization” representing the sample that viewed the least content. On average, engagement within the audience decreases following disruptions, although the subgroup closest to the organization increases its engagement. All subgroups decrease their engagement in the audience relative to total engagement.

Finally, we analyze the effect of the disruptions on social behavior within the audience (Fig. 4). On average, the disruptions cause users to reduce their engagement within their audience, both in terms of engagements received and engagements given, and in terms of the ratio of engagements within the audience to total engagements. This indicates that the disruptions lead users to engage with other communities more often. However, once again the result is dependent on distance to the removed users. The subgroup closest to the organization increased its engagement within this community in absolute count, although it decreased compared to total engagements, indicating that this subgroup increased its engagements outside of the audience following disruptions as well.

### Longer-Term Results.

The bulk of our results indicate that SNDs create a healthier experience for users in the audience and degrade the target audience network. However, we also note that the subgroup that engaged most with the removed users before the disruptions occurred exhibited signs of backlash by increasing its consumption and production of hateful content and its level of engagement within the audience. We now turn to investigating whether this backlash subsides over time, as it should if the disruptions cause long-term network degradation.

**Fig. 5.**
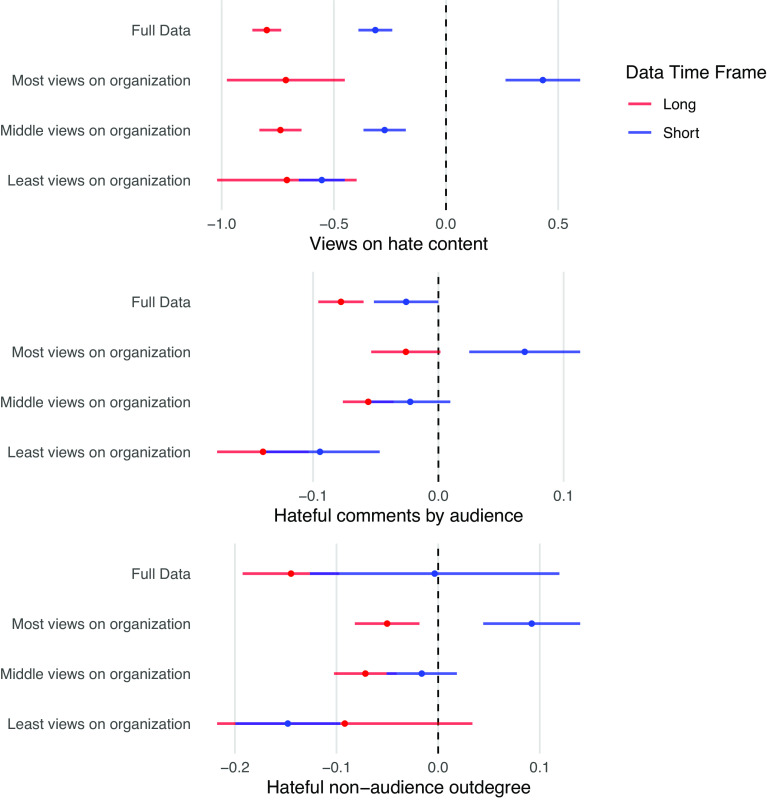
Effect of disruptions on backlash outcomes in the short and longer-term. Points represent the estimates for each model. Lines represent 95% confidence intervals. Beneath each plot is the outcome variable. The samples are defined by predisruption views of organization content, with “Most views on organization” representing the sample that viewed the most content and “Least views on organization” representing the sample that viewed the least content. While the subgroup closest to the organization increased its consumption and production of hateful content in the short term, this effect becomes negative in the longer-term. Moreover, the effect on hateful nonaudience outdegree becomes negative.

To determine whether the average effect changes in the longer run, we employ the same counterfactual estimators, but compare estimates from the short- and longer-term datasets with the sampled control groups included.^§§^ We estimate the effects of the disruptions on views and production of hateful content and hateful engagements inside and outside the audience (Fig. 5). We find that while in the short-term dataset the group with the most predisruption views of the removed users increased their views of hateful content after the disruptions, in the longer run, this effect becomes negative. Meanwhile, the effect remains negative for the other subgroups. This same pattern holds for the production of hateful content: We find a decrease in the average amount of hateful content posted daily by this subgroup in the longer term. Moreover, following the degradation of the audience network, this subgroup does not respond by increasing its hate speech in other communities, as would be expected if these users were simply seeking hateful communities on the platform. In the longer run, the disruptions have a negative effect on hateful engagements outside the audience.

One concern in comparing the short-term and longer-term effects is that in the longer-term dataset, one additional organization is treated. To show that this disruption is not solely driving the change in average treatment effects that we see, we estimate organization-specific treatment effects, shown in *SI Appendix*.^¶¶^ For nearly every organization and outcome, the ATTs are either less positive or become negative in the longer run.^##^ This finding suggests that it is not the newly treated organization that is driving our longer-term results, and its consistency suggests that the negative longer-run effects may be externally valid for many types of organizations. As noted above, we also employ dynamic event study estimators (26). While the short-term results from this approach are noisier, they largely confirm our key findings: In the short term, we find heterogenous effects based on the audience subgroups defined above, but in the longer-term find a consistent negative effect for the entire sample.

## Discussion

The results paint an optimistic picture of the ability of network disruptions to impede the ability of hateful organizations to reach their target audience online. On average, disruptions decrease the consumption and production of hateful content, along with engagement between members of the audience. Moreover, although the most-connected users exhibited signs of backlash in the short term, these effects dissipate over time: In the long term, the closest subgroup also reduces its engagement with hateful content. Considering our three sets of observable implications, our findings support our proposed mechanism.

We find that the audience reduces the amount of hate it consumes, and that the cohesiveness of the audience network degrades after a disruption: users interact with nonaudience members more than audience members as a proportion of their total interactions. If the hate organization were to reconstitute, the community that surrounded it would maintain its cohesion, and its members would have continued to consume hate.

Our evidence suggests that these organizations were not able to reach their broader audience through alternative means. Recent research has found that deplatforming efforts can lead banned organizations to reconstitute themselves on other platforms (28). Our analysis only captures the effects of these disruptions on Facebook, meaning we cannot observe their behavior on alternative platforms directly and cannot measure the full effects of deplatforming on social media as a whole. However, if the organization were able to continue to reach its audience that remained on Facebook, we would expect them to continue to act in accordance with the organization’s goals and produce hateful content. Instead, we see hate production decrease.

The evidence of backlash suggests that audience members did not immediately have another platform on which they could engage with the organization. If the most closely engaged users could easily connect to the organization elsewhere, they would have decreased their engagement immediately. Instead, they increased their engagement on Facebook. Even if these members ultimately do reach the organizations elsewhere, the disruptions successfully created friction in the ability of the organization to organize its closest audience. Moreover, many major platforms engage in data sharing after hate and terror disruptions through programs such as Tech Against Terror, reducing the effect of spillover.

We also find that the reduction in hate consumption was not driven solely by the level of readily available hate content. If the audience had sought out hateful content and the hate organization simply existed in an otherwise hateful community, then we would expect to see a constant level of hateful content. Instead, our evidence is consistent with hateful organizations pushing hateful content into the audience and creating a hateful community: Even excluding hate produced by the disrupted organization, the audience consumed less hate after the disruption. Moreover, without the hate organization pushing hateful content, even the users closest to the hate organization reduce their consumption over time.

Our unique data allow us to observe the real engagement networks of these organizations on Facebook, improving upon past studies which rely on qualitative or simulated mapping of ties between actors (31). These data suggest that hate organizations do indeed use networks to spread their ideology to their target audience (32, 33), and that dismantling these networks can protect their target audience from this influence.

The findings presented here have several implications for the future study of targeted efforts to disrupt illicit organizations. First, the time frame matters for drawing appropriate conclusions. Our findings indicate that disruptions may take time to have their full effects, especially as they rely on the degradation of ties between members to accomplish their end goal. Initial backlashes are not complete evidence of inefficacy. Second, the network position of audience members matters for the effect of disruptions. If researchers only measure the effects of disruptions on the most loyal members, they may miss the effect of disruptions on the ability of organizations to recruit and maintain ties with people who are more loosely exposed to the group.

This study contributes to the understanding of the effects of deplatforming events broadly. The findings generalize most strongly to social networking sites where interactions between users can be used to form a community, compared to social media sites that depend more on individual recommendations. They also generalize most strongly to disruptions against organizations that leverage the ability to form a community for recruitment and propaganda. Criminal organizations such as cartels or trafficking organizations may engage differently with their on-platform audiences.

Further research can help us understand when these findings will apply to different deplatforming approaches. While we show that SNDs are effective in reducing hate, the counterfactual in our study is not-yet disrupted groups. Future work may compare, for example, disruptions that remove organizations all at once versus those that remove key figures over time. While the approach studied here may be more effective in preventing organizations from replatforming, it could potentially provoke greater backlash than an overtime approach. Similarly, we cannot compare deplatforming to other counterhate actions, such as tagging posts as hateful, or using counter speech. While we show that deplatforming is effective, more research is necessary to determine its ideal form.

## Supplementary Material

Appendix 01 (PDF)

## Data Availability

We are unable to share the individual-level data that is used for our analysis. See https://about.meta.com/privacy-progress/. Aggregate data are provided in *SI Appendix*.

## Footnotes

^*^Organizations that proclaim a violent mission, engage in violence, and represent ideologies that promote hate are prohibited on Meta platforms. Praise, support, and representation of these organizations are removed, and members are deplatformed. Read more here: https://transparency.fb.com/policies/community-standards/dangerous-individuals-organizations/.

^†^Removing members is only one of many approaches platforms take to combat hate-based, terrorist, and criminal organizations. For example, see https://about.fb.com/news/2020/05/combating-hate-and-dangerous-organizations/ for an overview of enforcement approaches by Meta.

^‡^While we do not name the organizations, we study here due to security concerns, we note that all of the organizations meet the definition for “Dangerous Individuals and Organizations” on Facebook as outlined here: https://transparency.fb.com/policies/community-standards/dangerous-individuals-organizations/.

^§^Hate speech that violates Facebook’s community standards is removed. Exposure occurred either preremoval or on borderline content. Meta defines hate speech as “direct attack against people... on the basis of protected characteristics.” Read more here: https://transparency.fb.com/policies/community-standards/hate-speech/. Borderline content may attack concepts or institutions that could be interpreted to stand in for a group of people, or consist of offensive or divisive jokes and memes that do not constitute direct attacks. For information about borderline content, read more here: https://transparency.fb.com/en-gb/features/approach-to-ranking/content-distribution-guidelines/content-borderline-to-the-community-standards.

^¶^This study was conducted at Meta and not reviewed by the IRB, but was reviewed by an internal privacy review process. For more information please see https://about.meta.com/privacy-progress/. All analysis was conducted on deidentified data.

^#^None of the data in the study can be linked back to original user accounts. Data aggregated at the cluster-day are available as tables in supplementary material of this paper. We are unable to share the individual-level data underlying the analysis in the paper.

^ǁ^The classifier is a production classifier used at Facebook. In accordance with product guidance, we manually reviewed approximately 50 randomly sampled posts to evaluate a threshold at the classifier that captured violating content at roughly .4 precision, and with higher precision for borderline/divisive content. For information about borderline content, read more here: https://transparency.fb.com/en-gb/features/approach-to-ranking/content-distribution-guidelines/content-borderline-to-the-community-standards.

^**^While the timing of an investigation is not random, there are no observable controls to predict timing because we do not have the ability to detect organization activity before detailed investigations. This lack of detection means that the decision to prioritize an organization is not driven by patterns in the outcome variables. Moreover, because these disruptions take weeks to complete, these disruptions were in effect coprioritized and the staggered timing was due to investigative timing.

^††^While the placebo test fails with *P*-values from the DIM approach, it passes with *P*-values from the equivalence test. However: i) the *t* test is likely to suffer from limited power, especially with the limited number of clusters (24) and ii) our results are robust to other estimators, indicating that model selection is not driving the results.

^‡‡^Results for total engagement are in *SI Appendix*. Following disruptions, engagement drops across the sample on average. The number of daily views drops by more than 20. However, audience members closest to the removed users maintain similar levels of engagement, which is inconsistent with this subgroup changing platforms to follow the removed organization.

^§§^The longer-term dataset requires use of the sampled control groups so that there are never-treated clusters over the sample frame. We compare it to the short-term effects using the sampled control groups so that the samples are directly comparable.

^¶¶^Because the counterfactuals are imputed in our empirical approach, these treatment effects are simply the difference between realized treated outcomes and their counterfactuals averaged within organizations.

^##^There is only one exception: for Cluster 4, the ATT for hateful comments increases.
