# Supplementary material for: Disrupting hate: The effect of deplatforming hate organizations on their online audience
Source: Proc Natl Acad Sci U S A. 2023 Jun 5;120(24):e2214080120. doi: 10.1073/pnas.2214080120 (PMC10268571; doi:10.1073/pnas.2214080120)
Supplement: Supplementary file 1 — Appendix 01 (PDF) [file pnas.2214080120.sapp.pdf]

Appendix: Disrupting Hate: The Effect of Deplatforming Hate Organizations on their Online Audience

Daniel Robert Thomas, Laila A. Wahedi *Meta, Meta*

Contents

Summary statistics by disruption **2**

Audience subgroup summaries **3**

Treatment status over time by organization **5**

Summary statistics for outcomes with different data sets **6**

Plots of outcomes over time by disruption **8**

F test comparisons for Two-Way Fixed Effects and Interactive Fixed Effects **20**

Equivalence plots for main outcomes with short time frame and no sampled control groups **22**

Placebo test results for main outcomes with short time frame and no sampled control groups **34**

Tabular results for average ATT for short time frame with no sampled control groups **35**

Effects over time plots for all manuscript outcomes **38**

Effect of disruptions on consumption and production of ideologically aligned content **41**

Effect of disruptions on total engagement **42**

Tabular results for average ATT for short time frame with sampled control groups **43**

Tabular results for average ATT for long timeframe with sampled control groups **45**

Effects over time plots for all manuscript outcomes for long timeframe with sampled control groups **48**

Two Way Fixed Effects (OLS) for manuscript outcomes for short time frame with no sampled control groups **51**

Alternative Two Way Fixed Effects (OLS) models for ratio outcomes **54**

OLS with only time fixed effects for short time frame with no sampled control groups **56**

Two Way Fixed Effects (OLS) for short time frame with p-values from wild cluster bootstrapping **59**

Organization comparison plots for backlash outcomes **62**

Robustness: Dynamic differences-in-differences (Callaway and Sant'Anna 2021) for short time frame with no sampled control groups **66**

Robustness: Dynamic differences-in-differences (Callaway and Sant'Anna 2021) for short time frame with sampled control groups **136**

Robustness: Dynamic differences-in-differences (Callaway and Sant'Anna 2021) for long time frame with sampled control groups **206**

Tables of outcome variables aggregated at disruption and day level **316**

Summary statistics by disruption

Disruption	N	Mean Age	SD Age	Mean Gender	SD Gender	Mean Days Since Creation	SD Days Since Creation
Cluster 1	863	44.71	15.28	1.59	0.51	3421	1252
Cluster 2	1578	41.68	14.50	1.62	0.50	3459	1345
Cluster 3	8100	39.42	15.52	1.61	0.51	3160	1334
Cluster 4	3974	43.20	14.10	1.62	0.50	3430	1307
Cluster 5	3336	40.68	14.68	1.65	0.49	3357	1324
Cluster 6	8508	35.42	14.30	1.78	0.44	3318	1358
Sampled Cluster 1 Control	158	33.67	12.49	1.68	0.47	1754	1317
Sampled Cluster 2 Control	177	34.85	15.83	1.71	0.45	1643	1342
Sampled Cluster 3 Control	239	34.27	14.96	1.61	0.50	1519	1300
Sampled Cluster 4 Control	181	33.69	12.02	1.65	0.49	1627	1329
Sampled Cluster 5 Control	198	34.34	12.58	1.63	0.48	1619	1340
Sampled Cluster 6 Control	196	34.10	14.73	1.61	0.51	1713	1302

Table A.1: Summary statistics for the demographics of the audiences

Audience subgroup summaries

Organization	Audience Level	N	Mean Age	SD Age	Mean Gender	SD Gender	Mean Days Since Creation	SD Days Since Creation
Cluster 1	1	285	45.95	15.83	1.65	0.49	3391	1304
Cluster 1	2	356	44.21	14.38	1.60	0.50	3439	1188
Cluster 1	3	222	43.94	15.90	1.51	0.54	3432	1287
Cluster 2	1	489	44.76	15.26	1.62	0.50	3542	1260
Cluster 2	2	655	39.70	13.06	1.63	0.50	3398	1425
Cluster 2	3	434	41.21	15.12	1.59	0.51	3460	1311
Cluster 3	1	1353	41.70	15.18	1.60	0.52	3362	1311
Cluster 3	2	3522	38.27	14.87	1.62	0.50	3190	1267
Cluster 3	3	3225	39.71	16.22	1.60	0.51	3042	1402
Cluster 4	1	1082	42.98	13.03	1.64	0.49	3516	1239
Cluster 4	2	1649	42.23	13.49	1.63	0.50	3461	1304
Cluster 4	3	1243	44.68	15.60	1.58	0.51	3316	1362
Cluster 5	1	721	42.49	14.89	1.67	0.49	3293	1354
Cluster 5	2	1566	39.28	14.26	1.66	0.49	3368	1320
Cluster 5	3	1049	41.53	14.97	1.63	0.49	3384	1309
Cluster 6	1	1957	33.88	12.72	1.84	0.39	3243	1362
Cluster 6	2	3346	34.71	14.07	1.79	0.43	3407	1294
Cluster 6	3	3205	37.10	15.25	1.72	0.47	3271	1415

Table A.2: Subgroup demographics. Subgroups are largely similar within disruptions.

Disruption	Subgroup	Number	Number friends with organization	Prop. friends with organization
Cluster 4	1	1082	1041	0.9621
Cluster 4	2	1649	1407	0.8532
Cluster 4	3	1243	563	0.4529
Cluster 1	1	285	260	0.9123
Cluster 1	2	356	238	0.6685
Cluster 1	3	222	92	0.4144
Cluster 6	1	1957	1231	0.6290
Cluster 6	2	3346	1514	0.4525
Cluster 6	3	3205	1092	0.3407
Cluster 3	1	1353	1213	0.8965
Cluster 3	2	3522	2629	0.7465
Cluster 3	3	3225	1754	0.5439
Cluster 5	1	721	654	0.9071
Cluster 5	2	1566	999	0.6379
Cluster 5	3	1048	448	0.4275
Cluster 2	1	489	467	0.9550
Cluster 2	2	655	476	0.7267
Cluster 2	3	434	207	0.4770

Table A.3: Relationship between subgroups and being friends with organization members. Those who engage more with the organization are also friends with at least one organization member more often than those who engage less.

Treatment status over time by organization

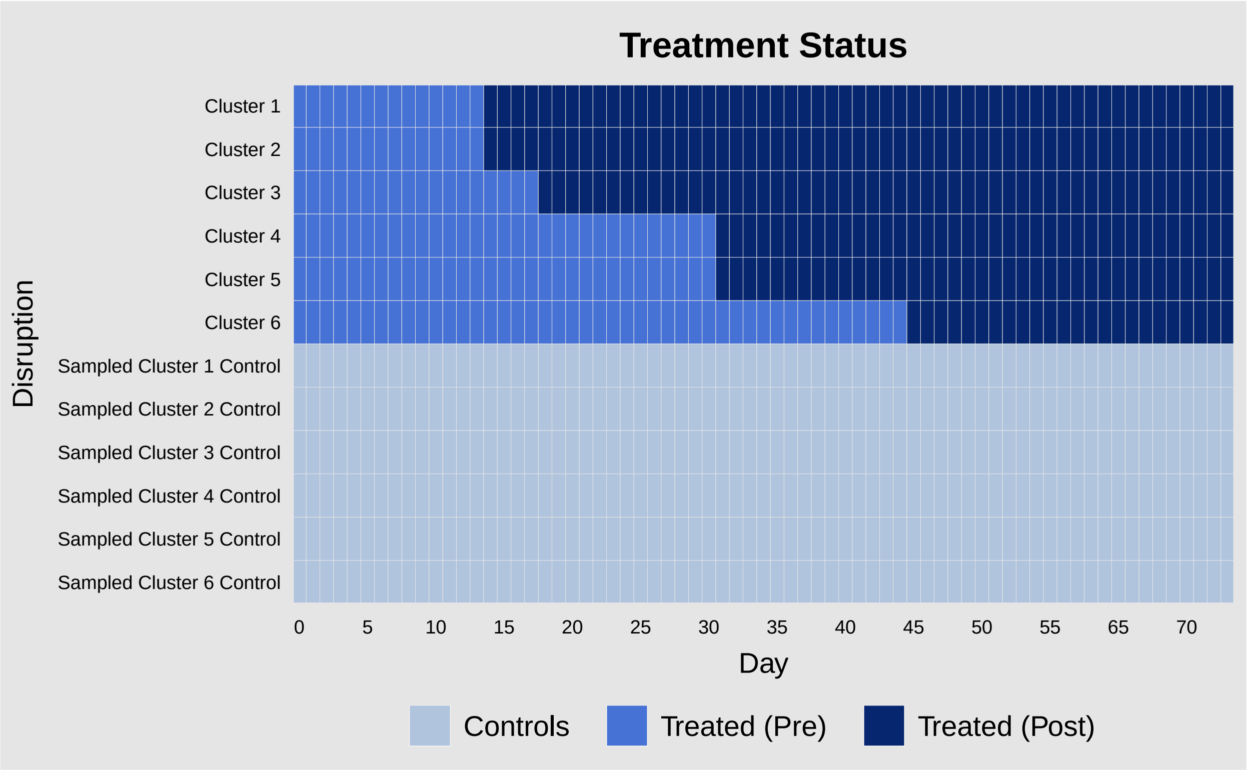

Figure A.1: Treatment status of organizations over time, including sampled control groups.

Summary statistics for outcomes with different data sets

Statistic	N	Mean	St. Dev.	Min	Max
Views on hate content	1,186,155	4.942	8.632	0	421
Views on hate content/total views	1,122,349	0.012	0.021	0.000	1.000
Views on non-organization content that is hateful	1,186,155	4.936	8.628	0	421
Hateful comments by audience members	1,186,155	0.558	2.656	0	254
Hateful comments/total comments	743,156	0.034	0.101	0.000	1.000
Hateful audience outdegree	1,186,155	0.092	0.529	0	37
Hateful non-audience outdegree	1,186,155	0.845	3.112	0	277
Audience indegree	1,186,155	1.270	6.155	0	653
Audience outdegree	1,186,155	1.274	3.865	0	223
Audience indegree/total indegree	916,080	0.054	0.137	0.000	1.000
Audience outdegree/total outdegree	985,292	0.070	0.152	0.000	1.000

Table A.4: Summary statistics for outcome variables with primary (short timeframe) dataset.

Statistic	N	Mean	St. Dev.	Min	Max
Views on hate content	1,237,860	4.777	8.516	0	421
Views on hate content/total views	1,169,553	0.012	0.021	0.000	1.000
Views on non-organization content that is hateful	1,237,860	4.772	8.513	0	421
Hateful comments by audience members	1,237,860	0.535	2.603	0	254
Hateful comments/total comments	756,486	0.033	0.100	0.000	1.000
Hateful audience outdegree	1,237,860	0.088	0.518	0	37
Hateful non-audience outdegree	1,237,860	0.811	3.051	0	277
Audience indegree	1,237,860	1.217	6.030	0	653
Audience outdegree	1,237,860	1.220	3.792	0	223
Audience indegree/total indegree	934,545	0.053	0.136	0.000	1.000
Audience outdegree/total outdegree	1,012,522	0.068	0.150	0.000	1.000

Table A.5: Summary statistics for outcome variables with short timeframe dataset with sampled control groups.

Statistic	N	Mean	St. Dev.	Min	Max
Views on hate content	1,897,610	4.375	7.900	0	421
Views on hate content/total views	1,782,250	0.011	0.020	0.000	1.000
Views on non-organization content that is hateful	1,897,610	4.372	7.897	0	421
Hateful comments by audience members	1,897,610	0.501	2.472	0	254
Hateful comments/total comments	1,147,537	0.032	0.099	0.000	1.000
Hateful audience outdegree	1,897,610	0.078	0.479	0	37
Hateful non-audience outdegree	1,897,610	0.761	2.911	0	277
Audience indegree	1,897,610	1.139	5.763	0	653
Audience outdegree	1,897,610	1.139	3.609	0	223
Audience indegree/total indegree	1,419,451	0.051	0.133	0.000	1.000
Audience outdegree/total outdegree	1,542,687	0.064	0.143	0.000	1.000

Table A.6: Summary statistics for outcome variables with long timeframe dataset with sampled control groups.

Plots of outcomes over time by disruption

Views on hateful content

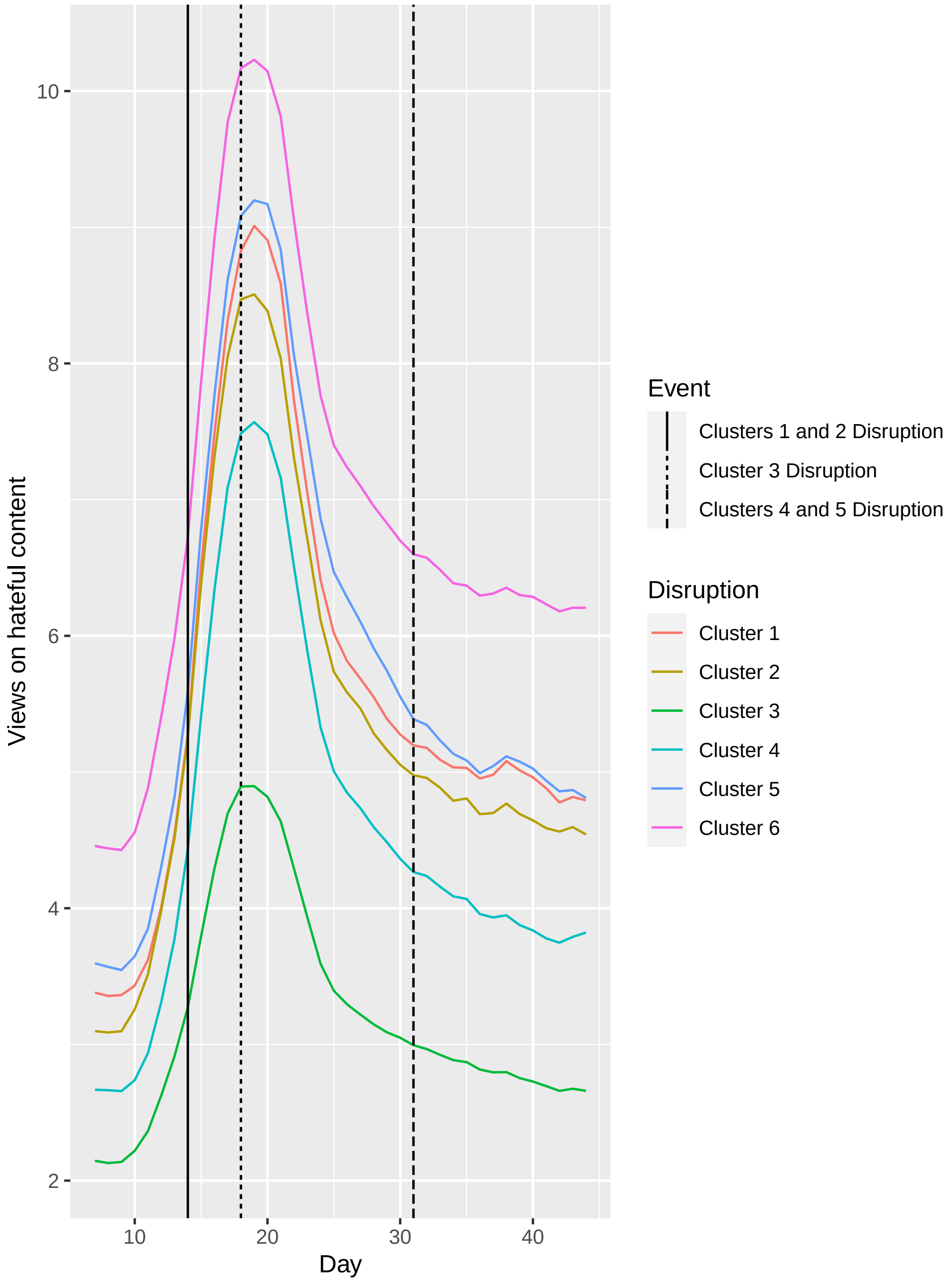

Views on hateful content/total views

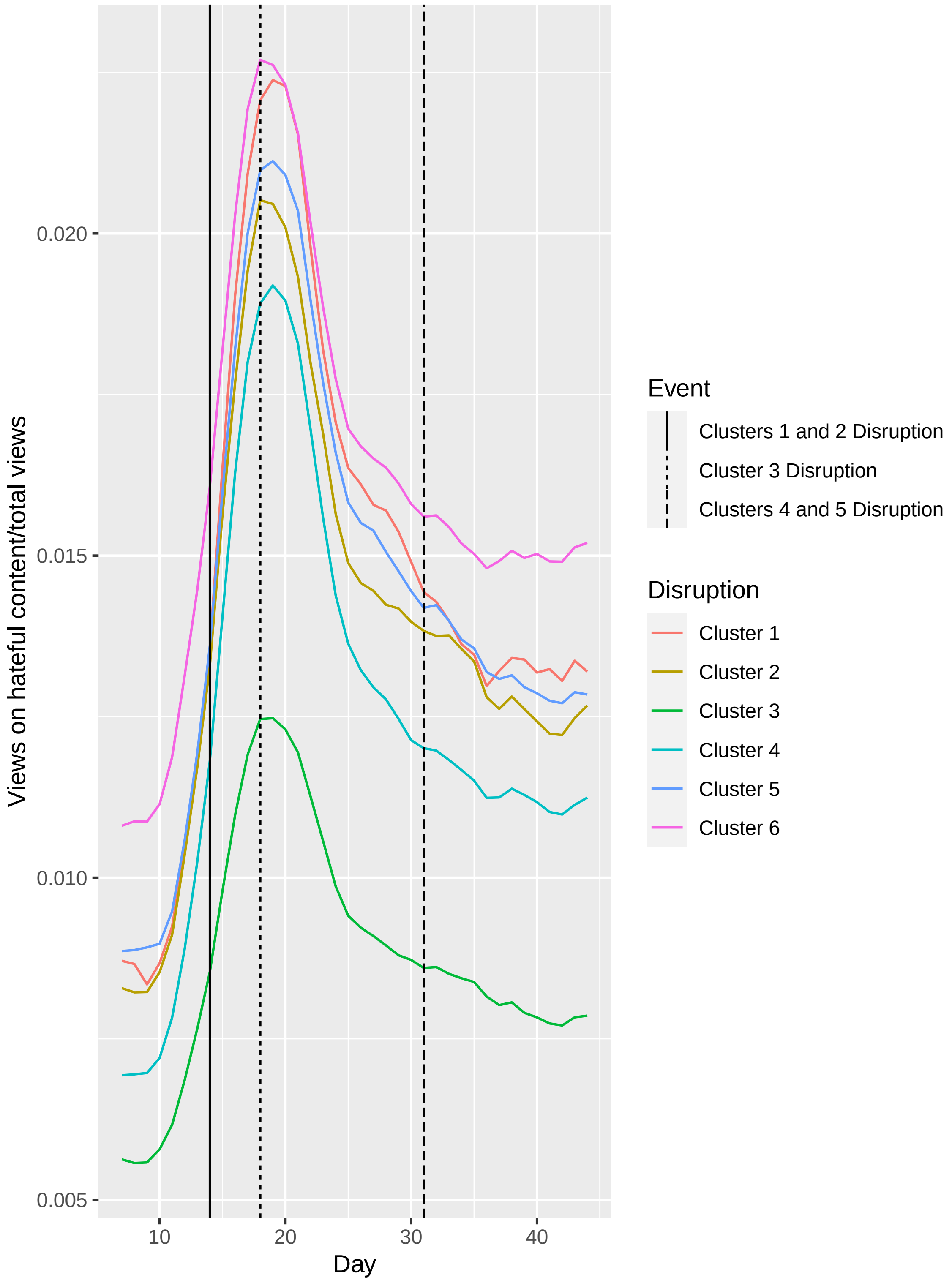

Views on non-organization content that is hateful

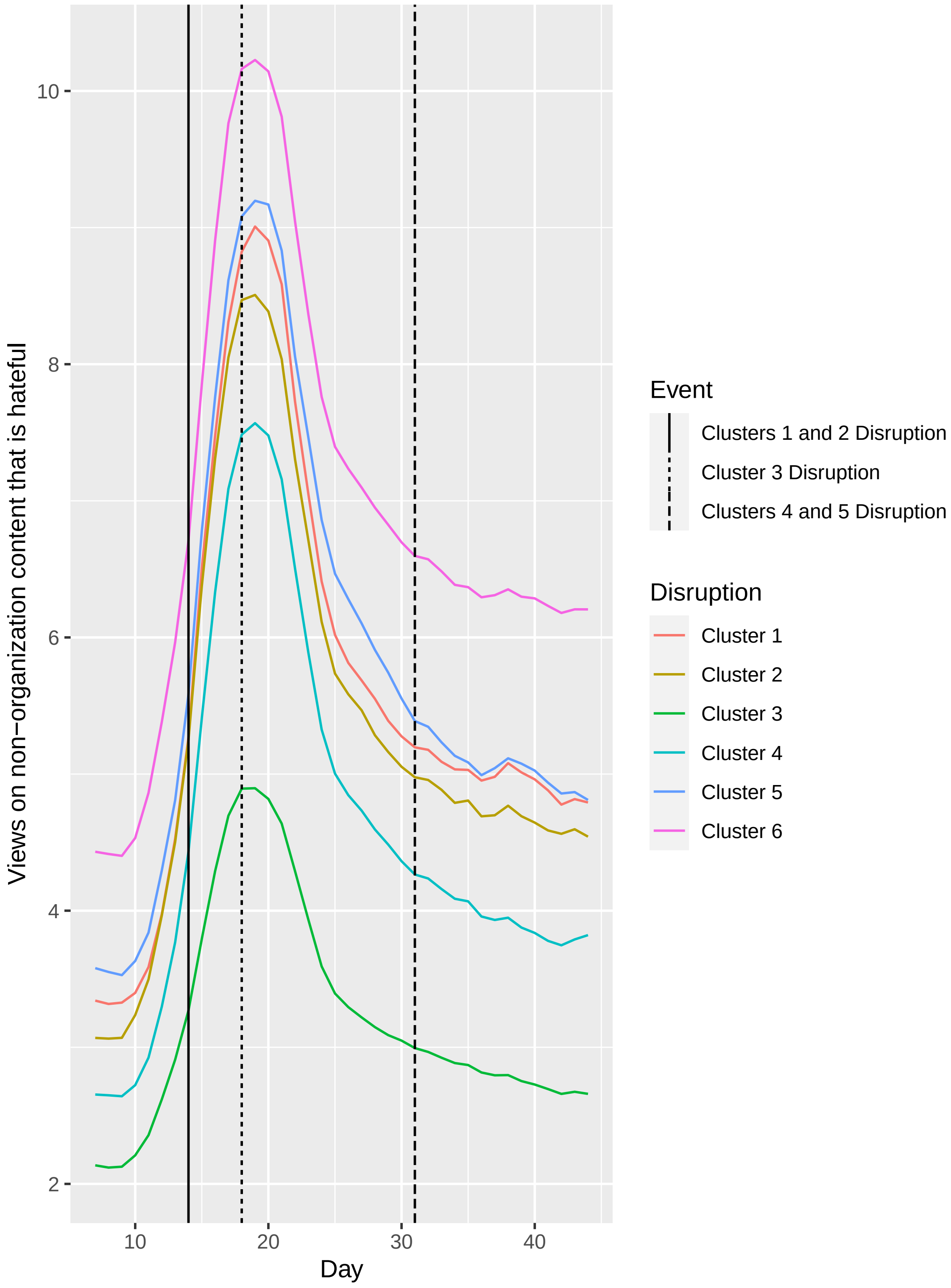

Hateful comments by audience members

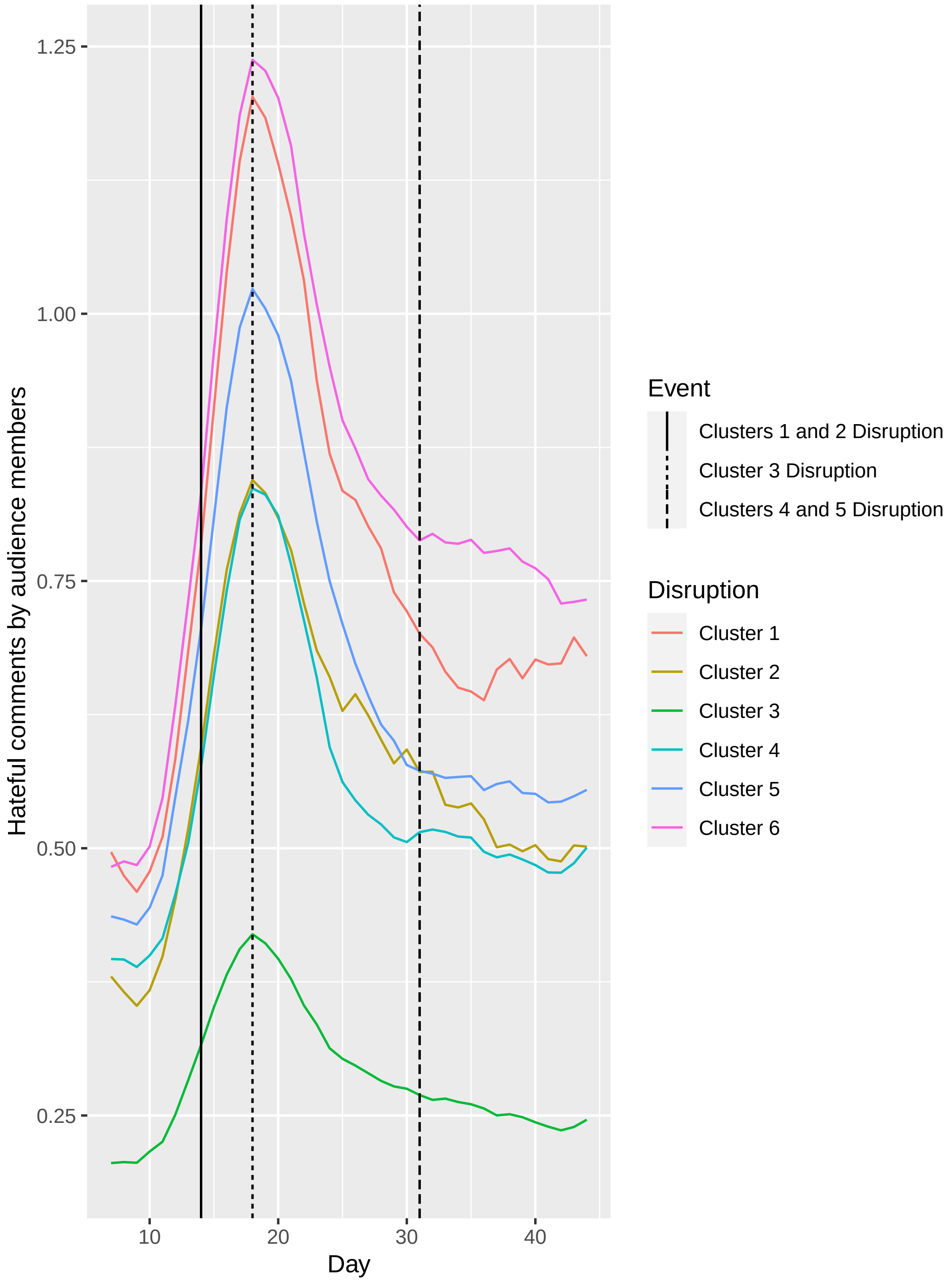

Hateful comments/total comments

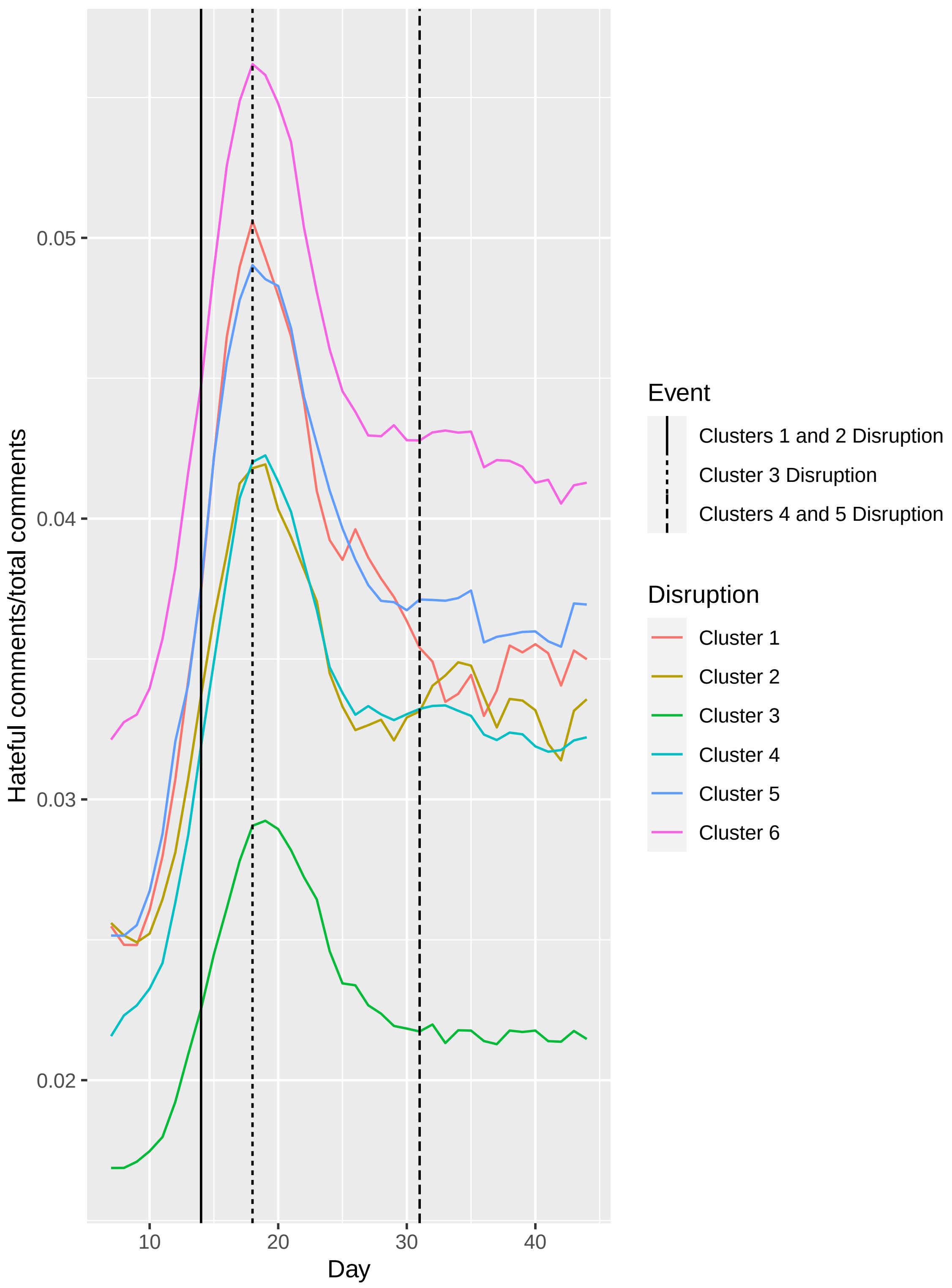

Hateful audience outdegree

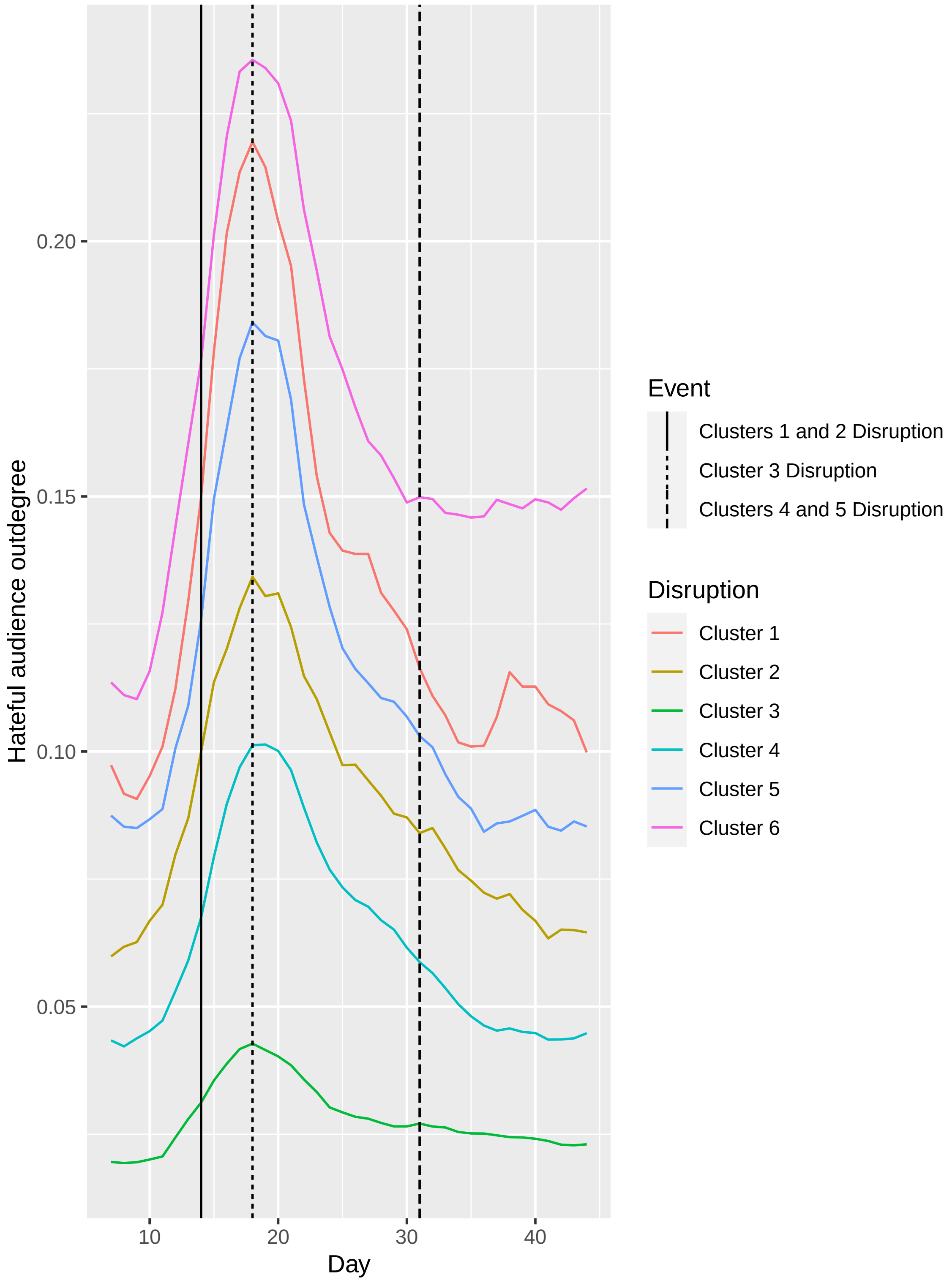

Hateful non-audience outdegree

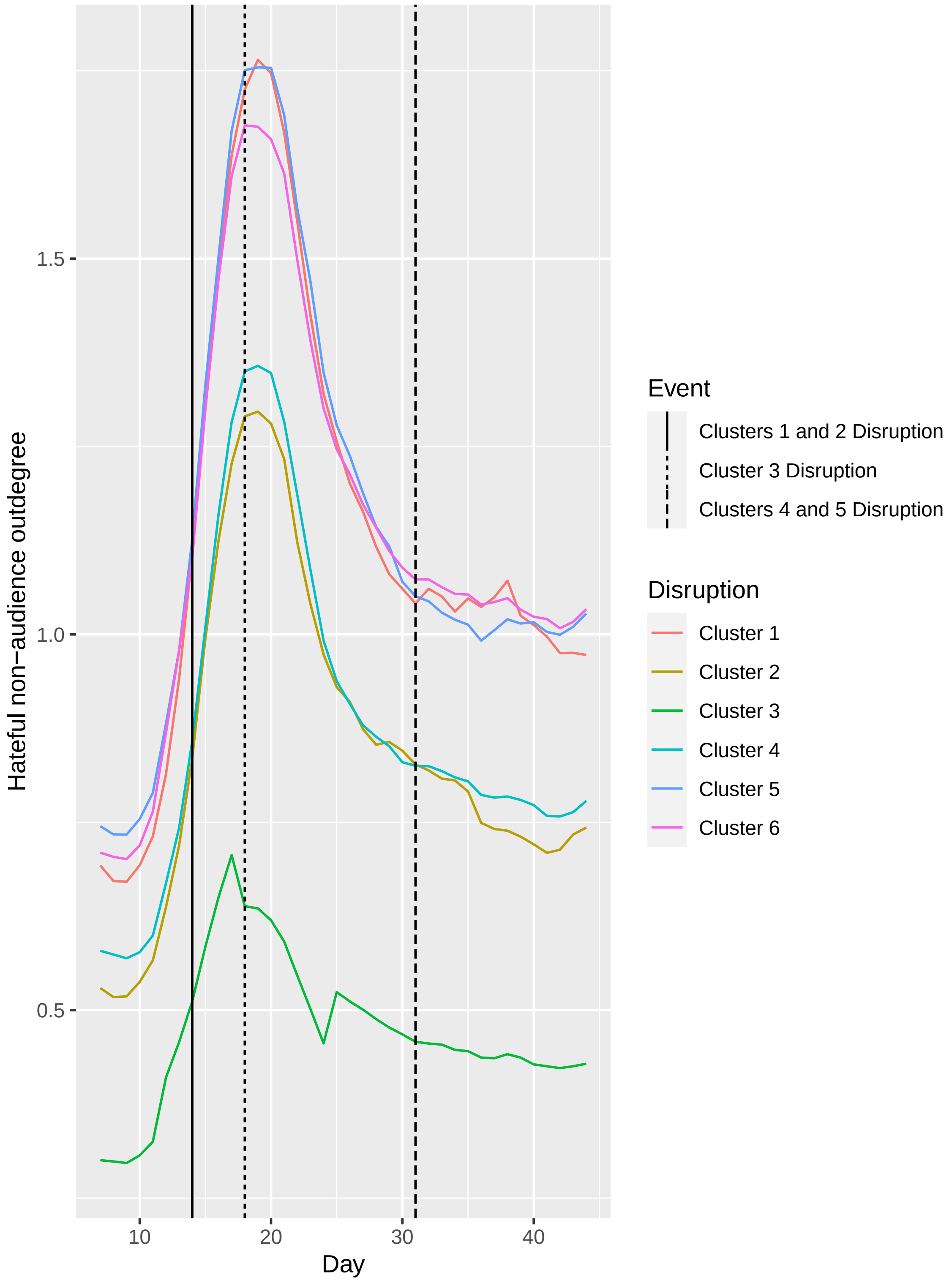

Audience indegree

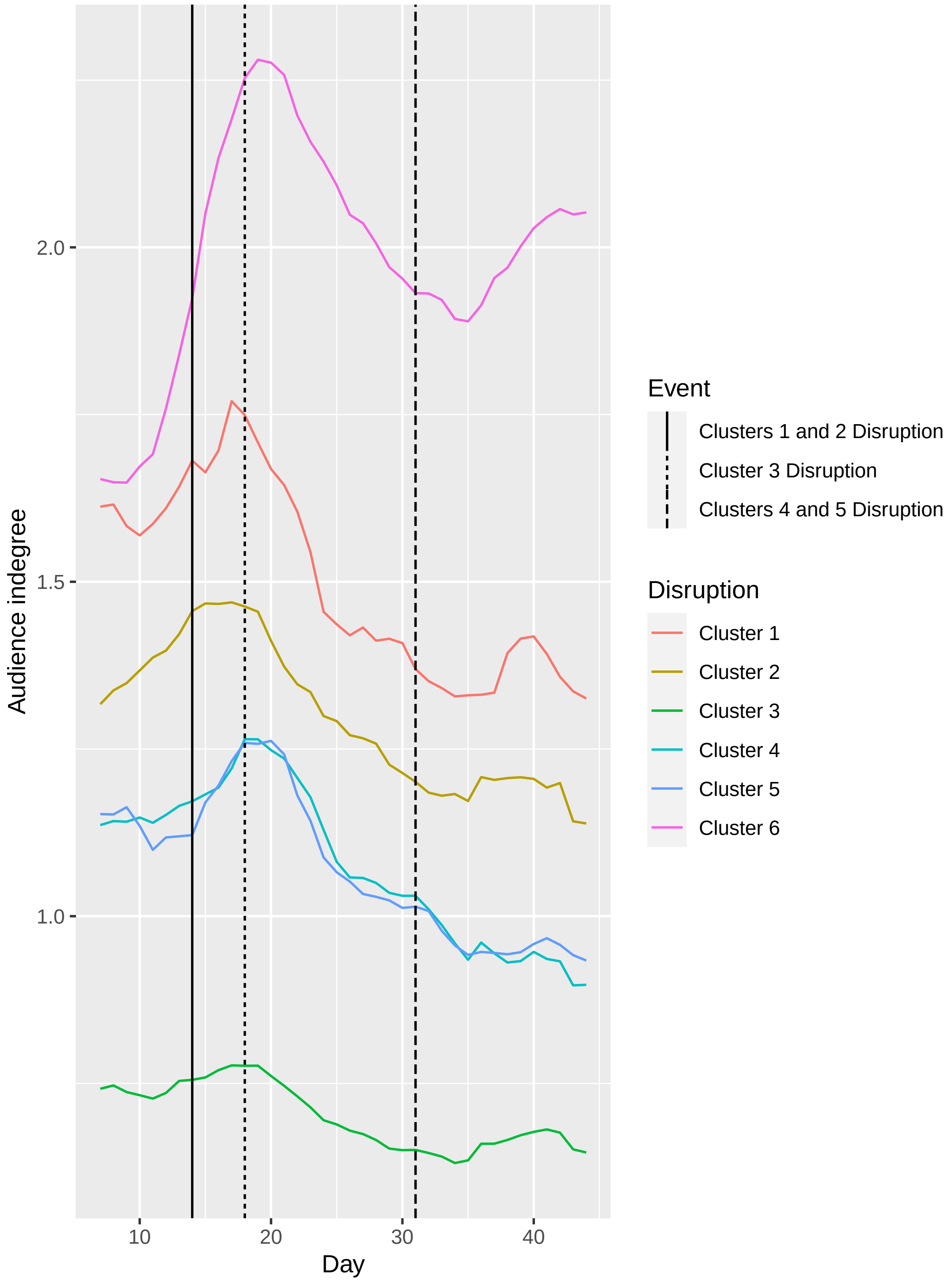

Audience outdegree

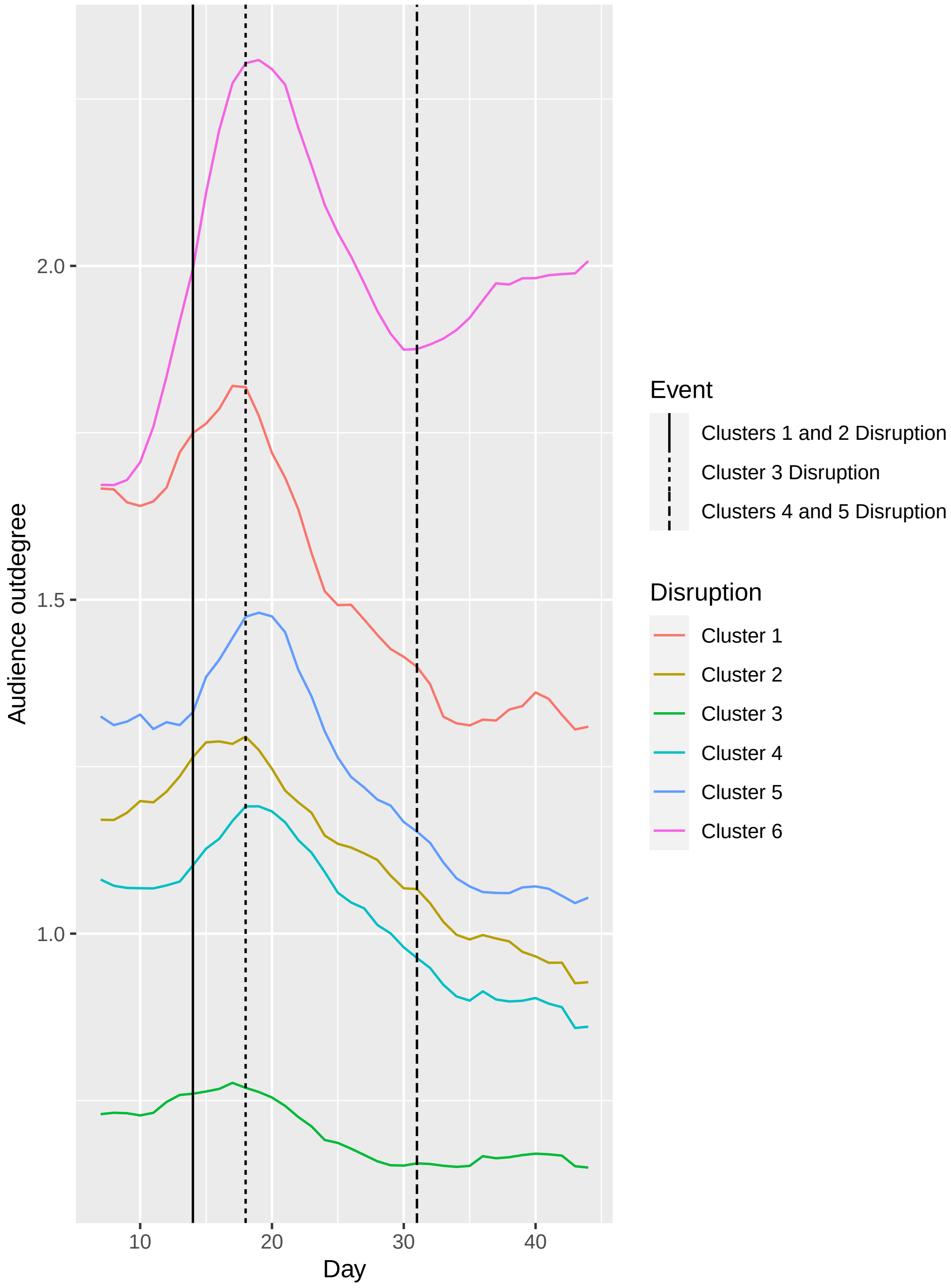

Audience indegree/total indegree

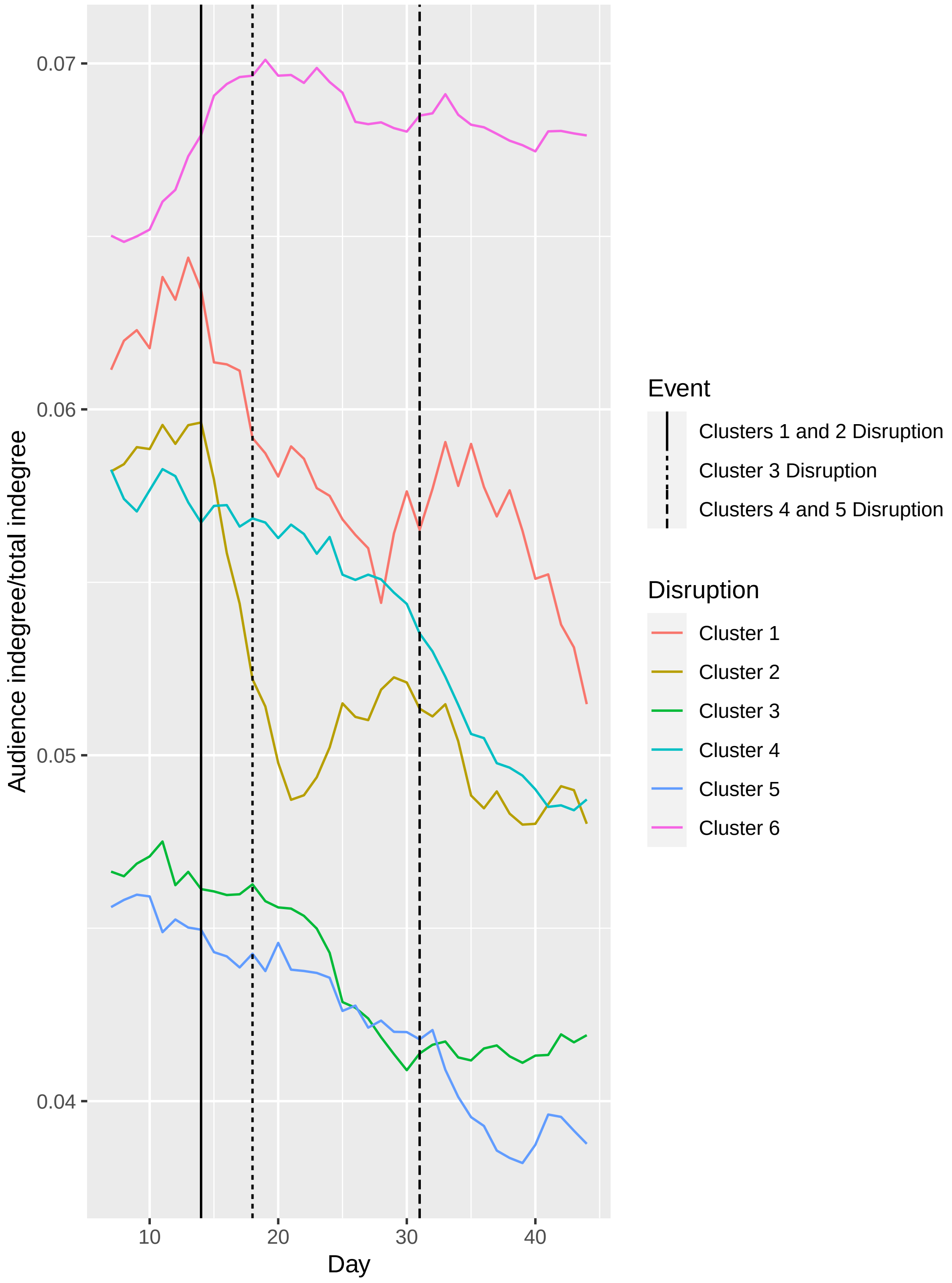

Audience outdegree/total outdegree

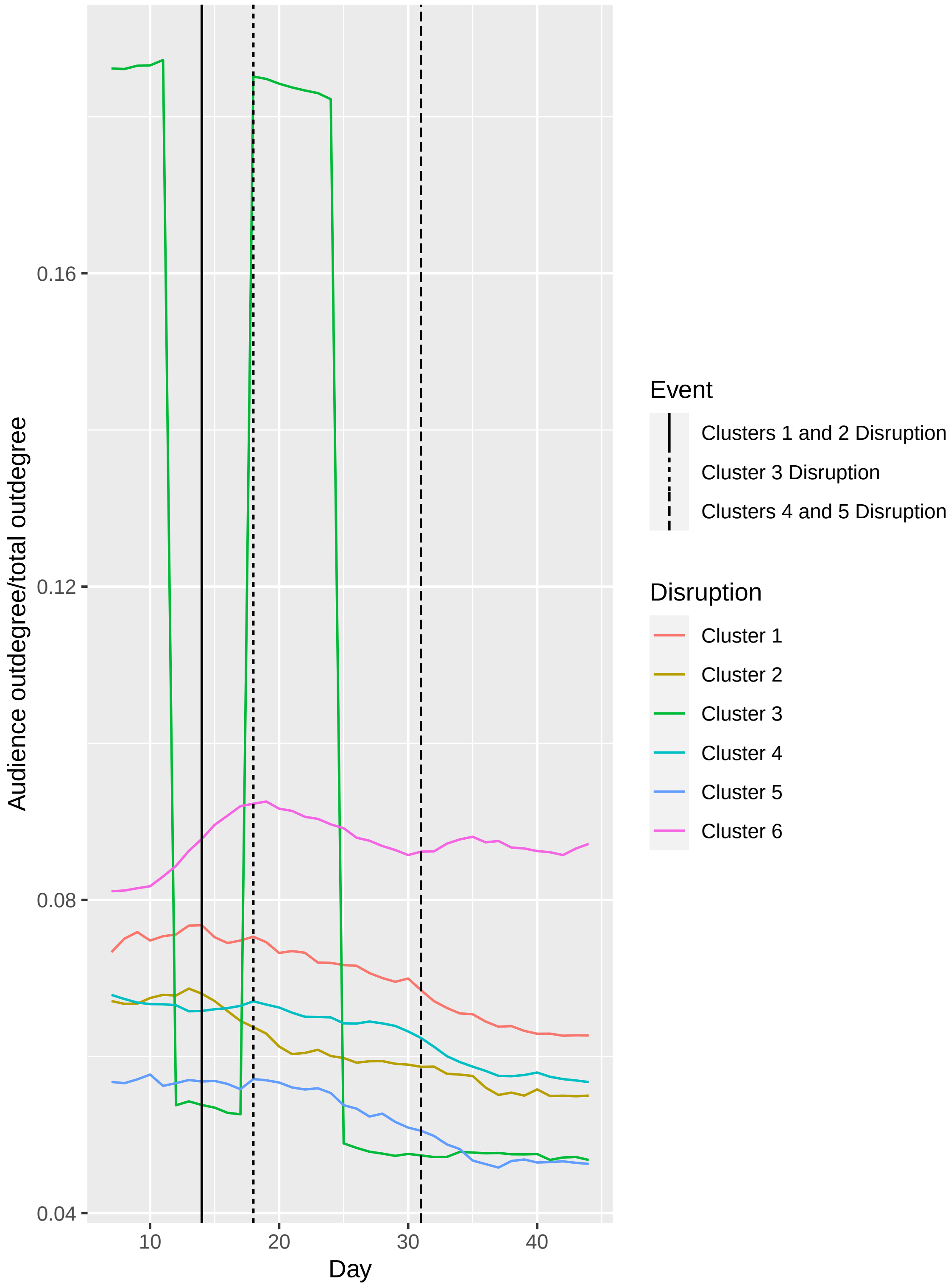

F test comparisons for Two-Way Fixed Effects and Interactive Fixed Effects

Table A.7: F test for zero average residuals in pre-treatment periods with full sample. The model with the lower F-Stat was chosen.

Outcome	TWFE F-Stat	TWFE p-value	IFE F-Stat	IFE p-value
Views on hateful content	32.299	0	3.878	0
Views on hateful content/total views	38.679	0	25.002	0
Hateful comments by audience members	9.575	0	7.698	0
Hateful comments/total comments	7.835	0	7.620	0
Views on non-organization content that is hateful	33.383	0	3.999	0
Audience outdegree	10.521	0	12.110	0
Audience indegree	3.694	0	3.244	0
Hateful audience outdegree	10.027	0	8.674	0
Hateful non-audience outdegree	17.025	0	6.561	0
Audience indegree/total indegree	3.404	0	3.468	0
Audience outdegree/total outdegree	105.305	0	138.852	0

Equivalence plots for main outcomes with short time frame and no sampled control groups

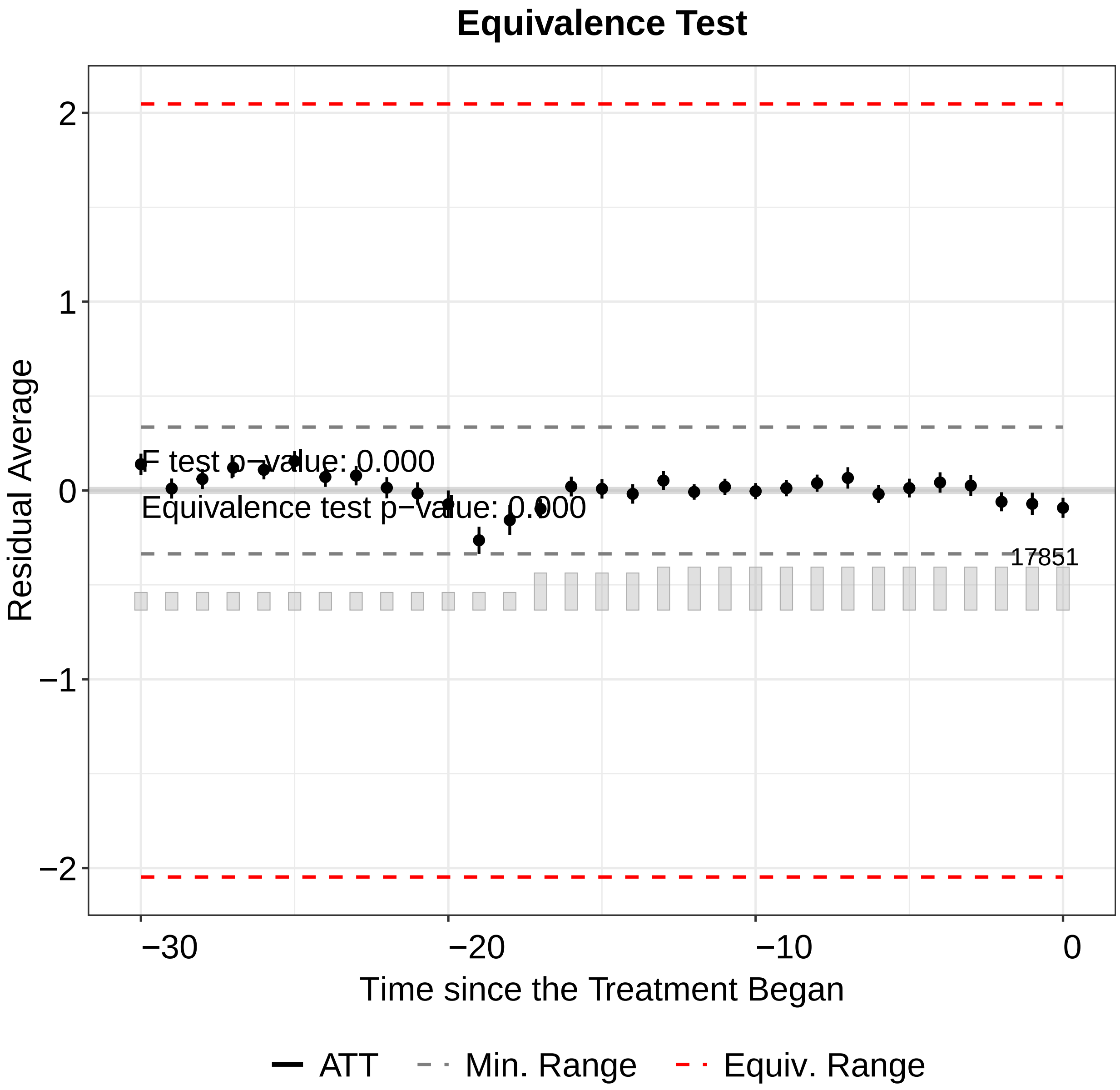
Equivalence Test
Residual Average
2
1
0
−1
−2
F test p−value: 0.000
Equivalence test p−value: 0.000
17851
−30
−20
−10
0
Time since the Treatment Began
ATT
Min. Range
Equiv. Range

Views on hateful content/total views

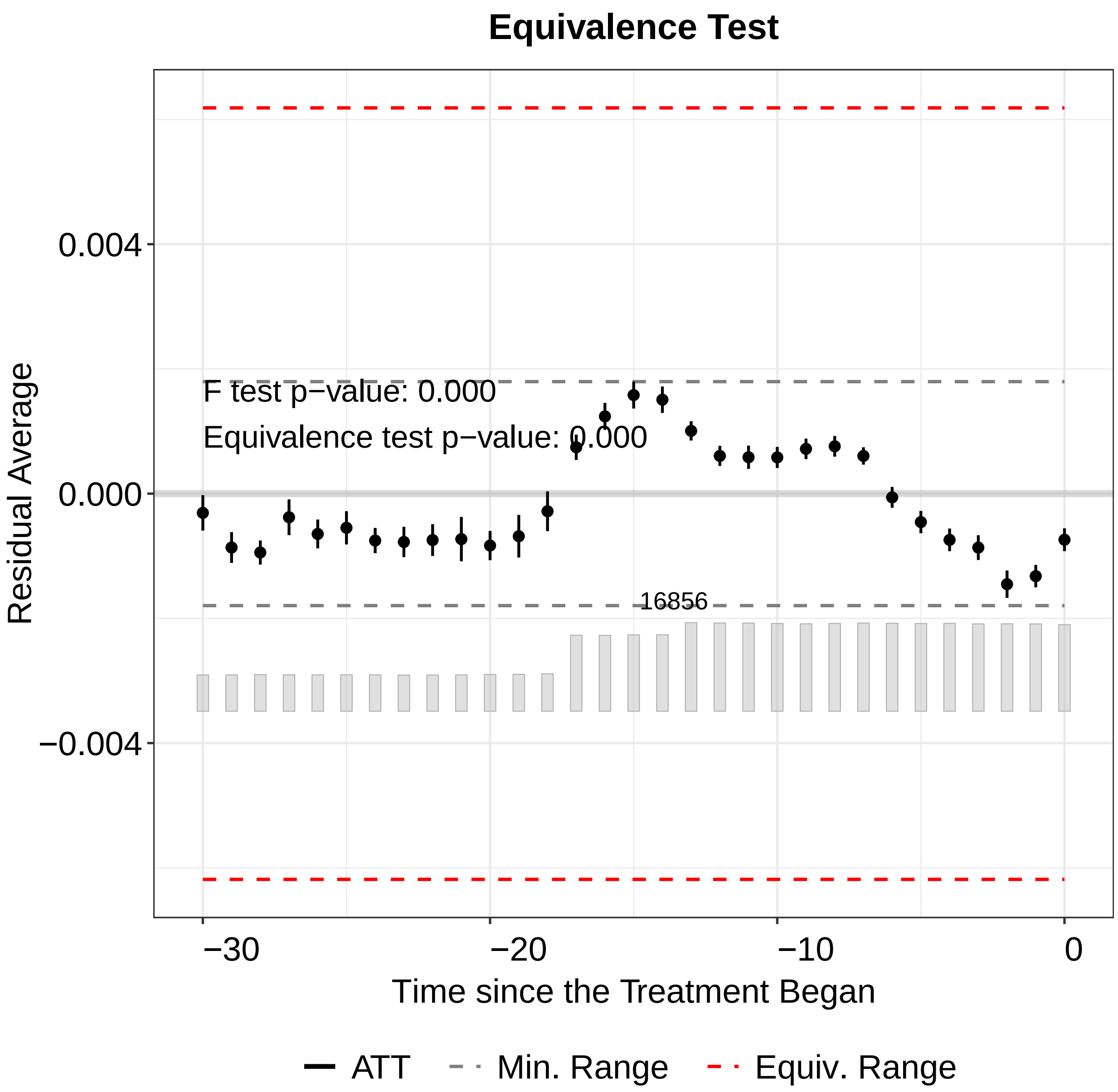

Views on non-organization content that is hateful

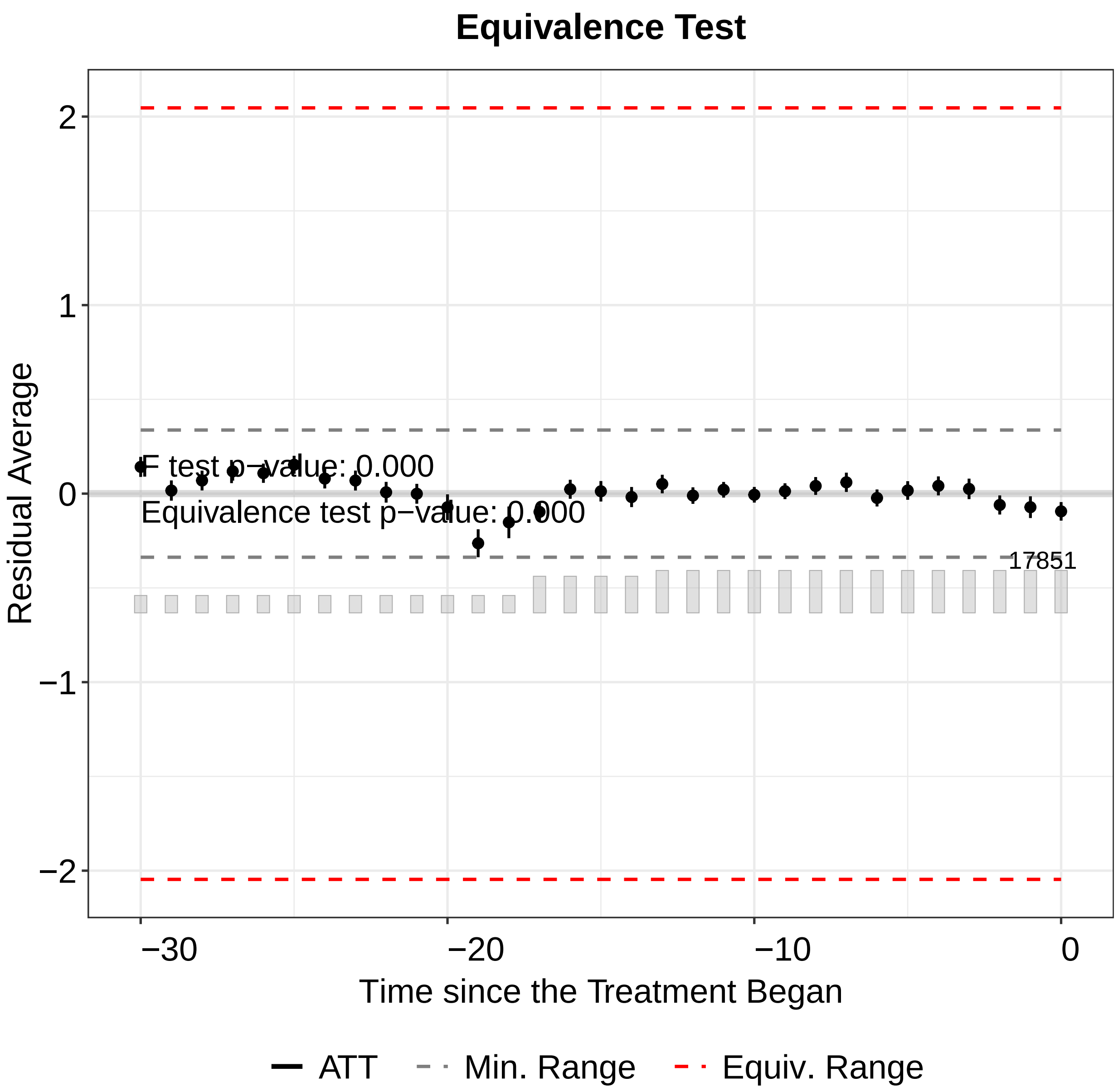

Hateful comments by audience members

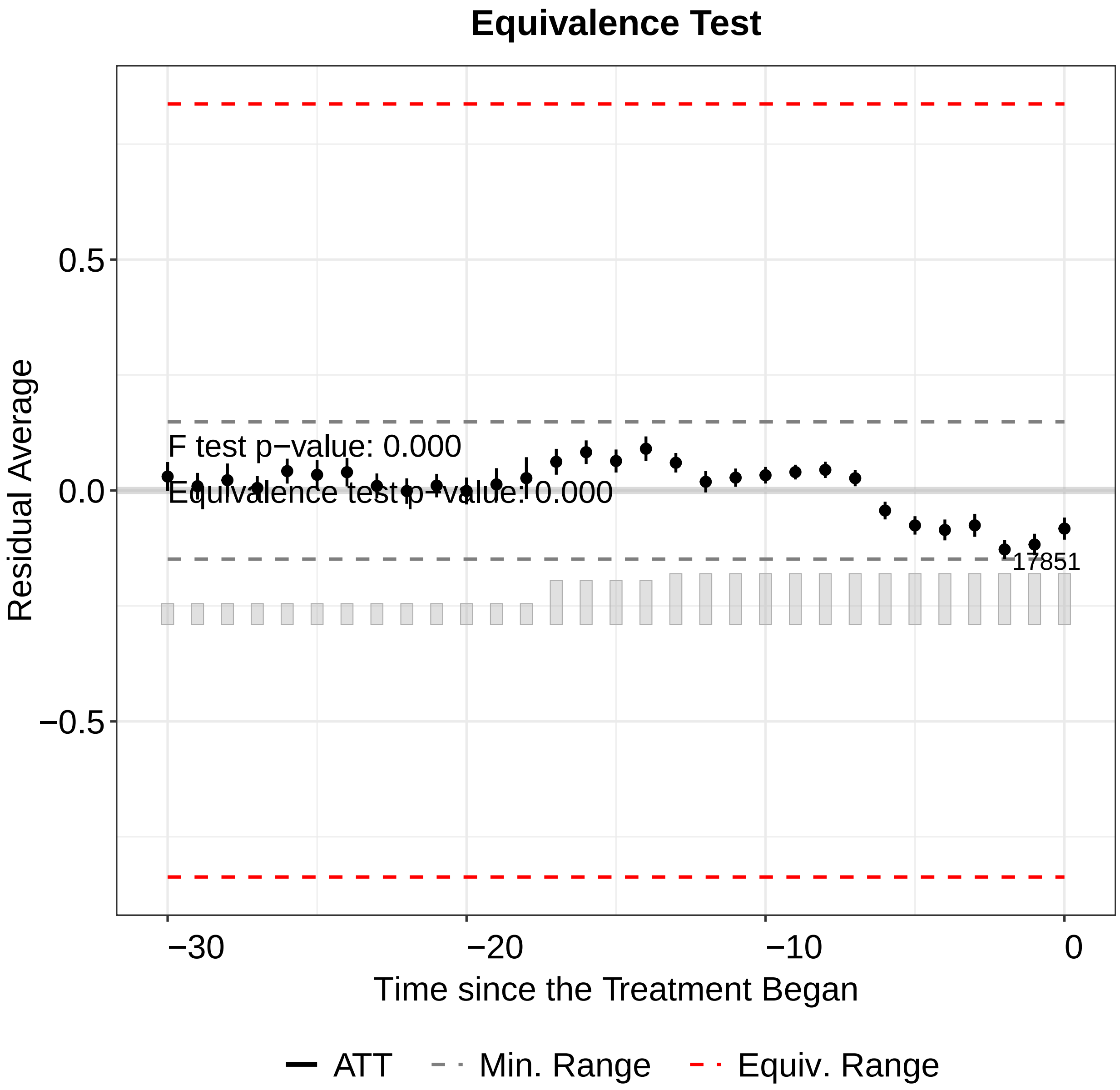

Hateful comments/total comments

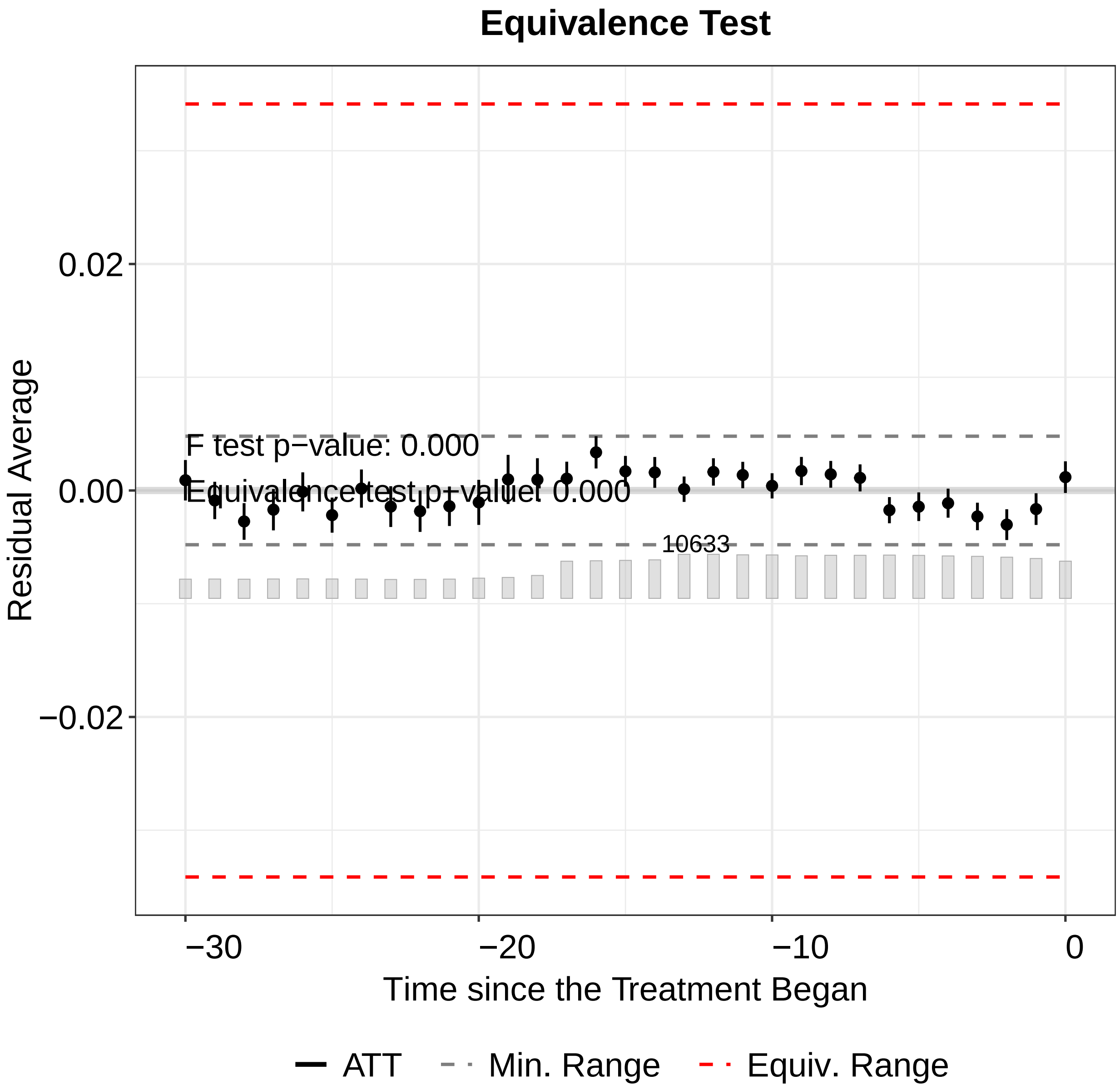

Hateful audience outdegree

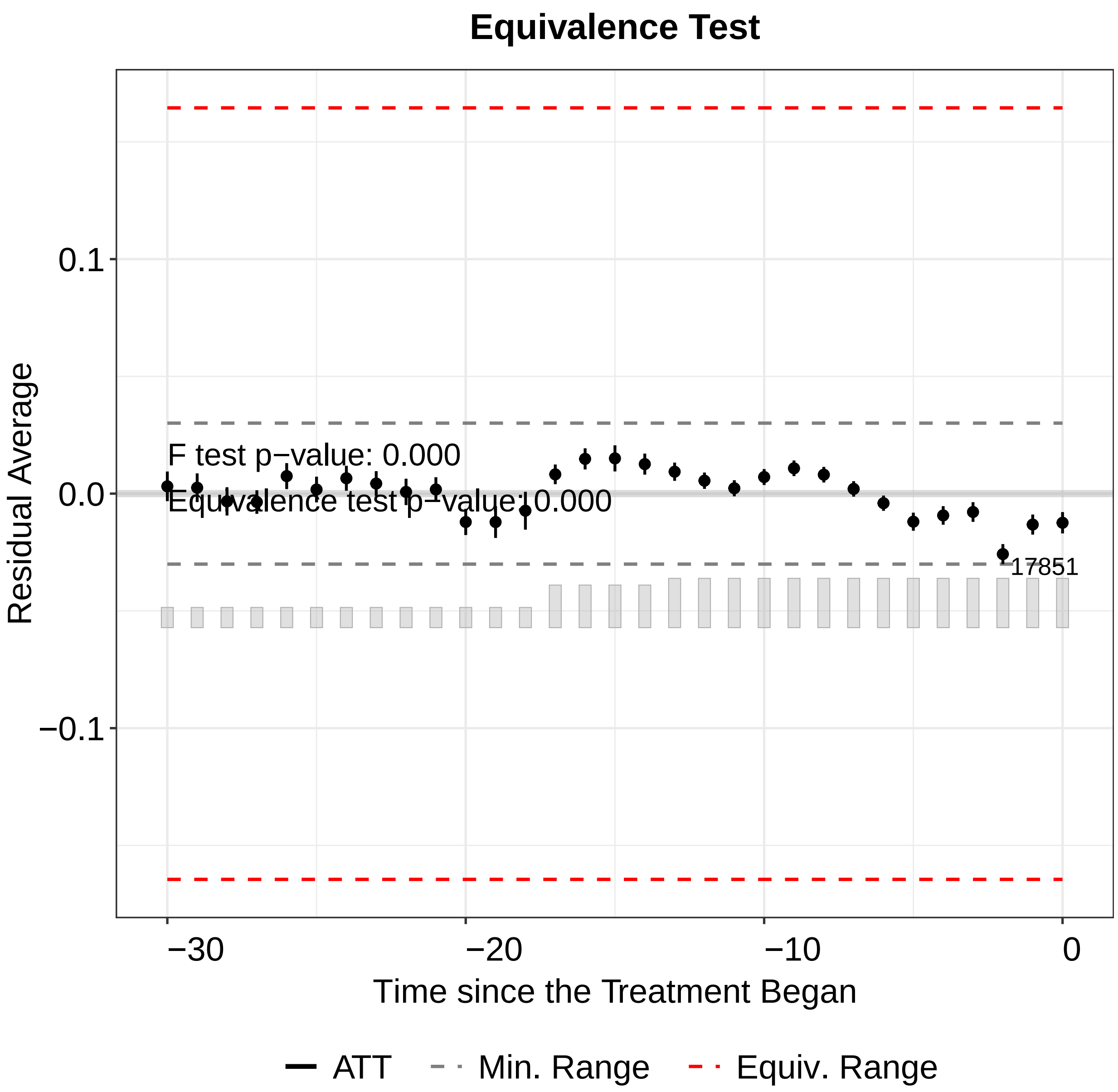

Hateful non-audience outdegree

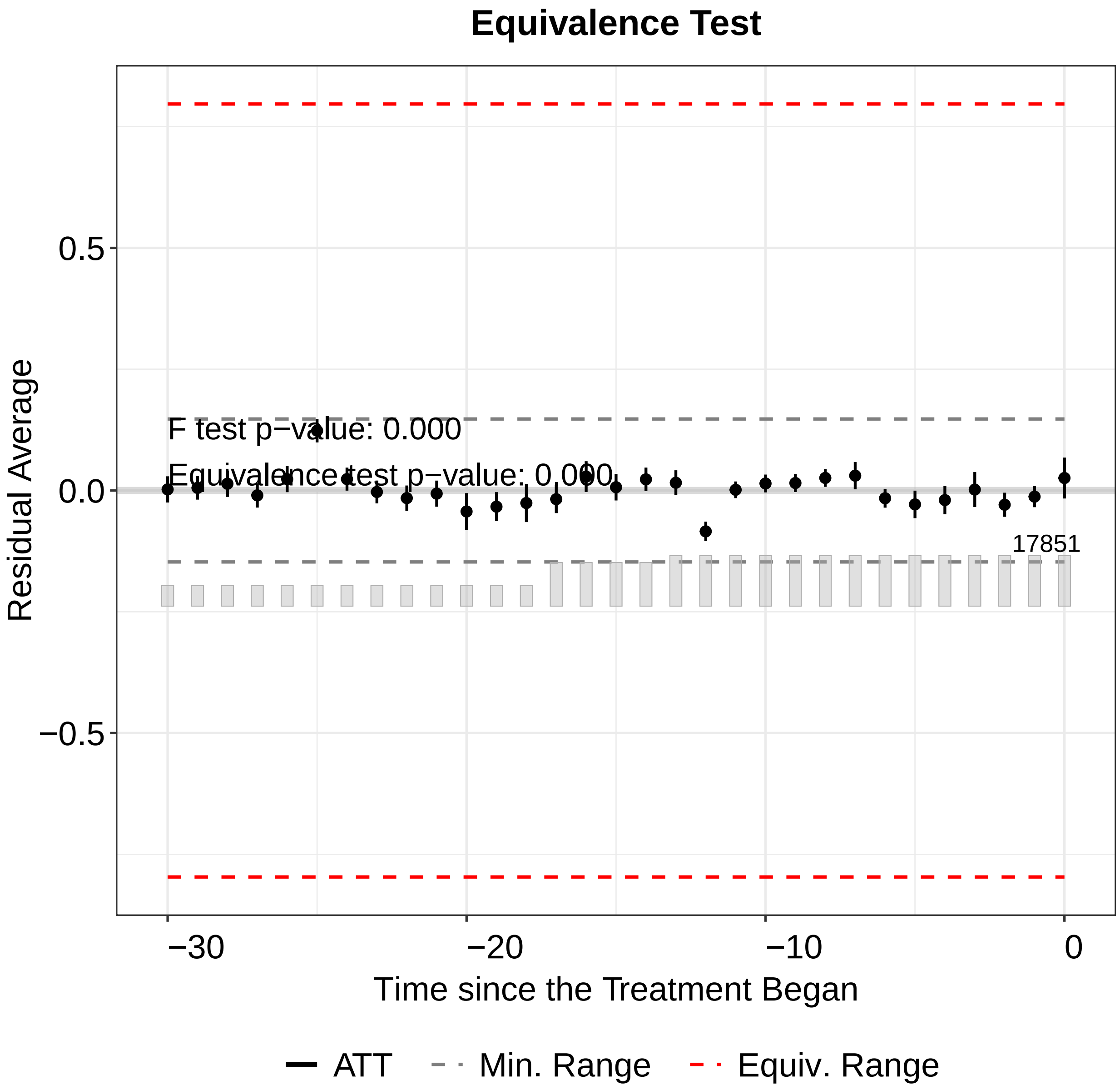

Audience indegree

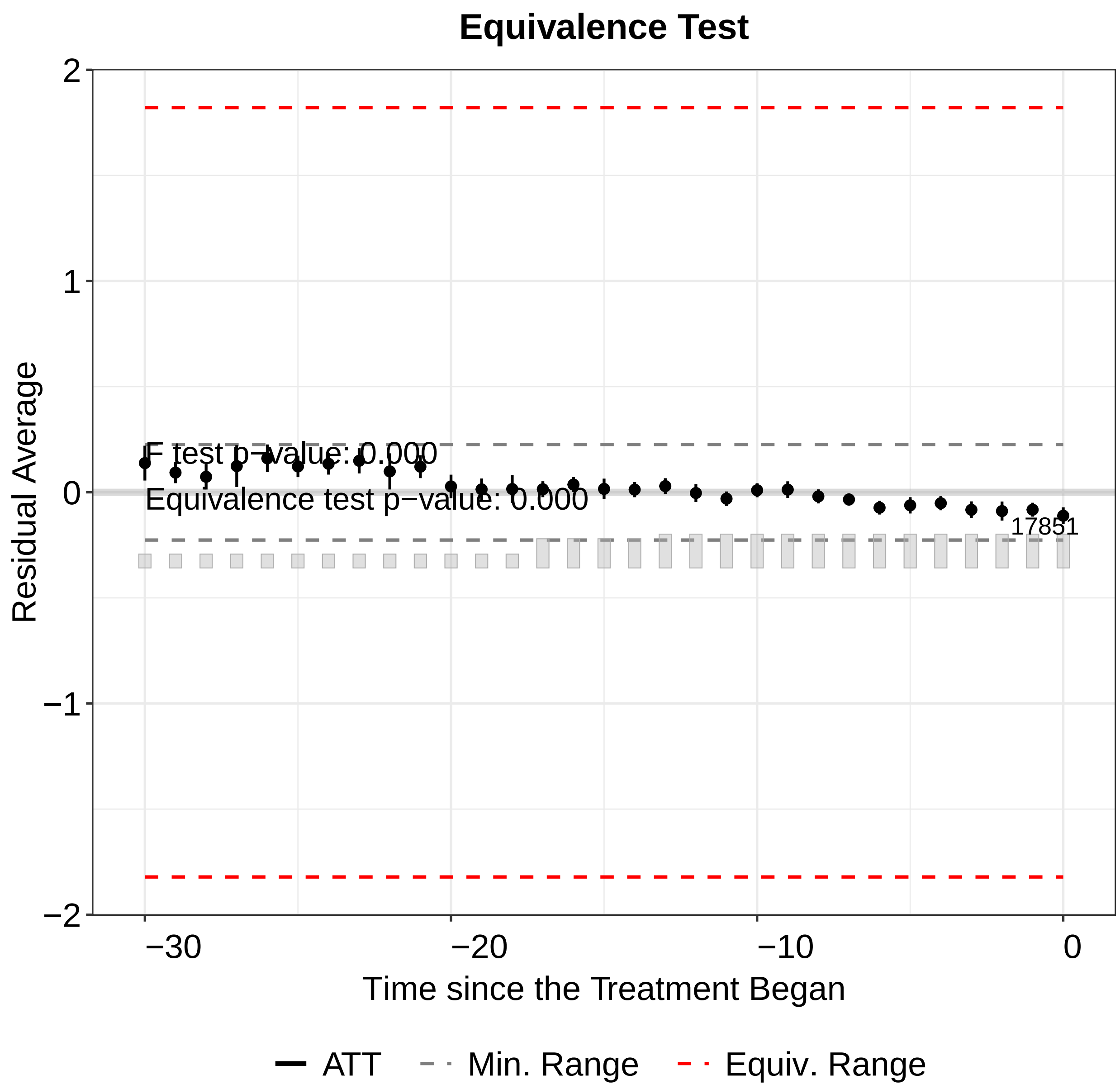

Audience outdegree

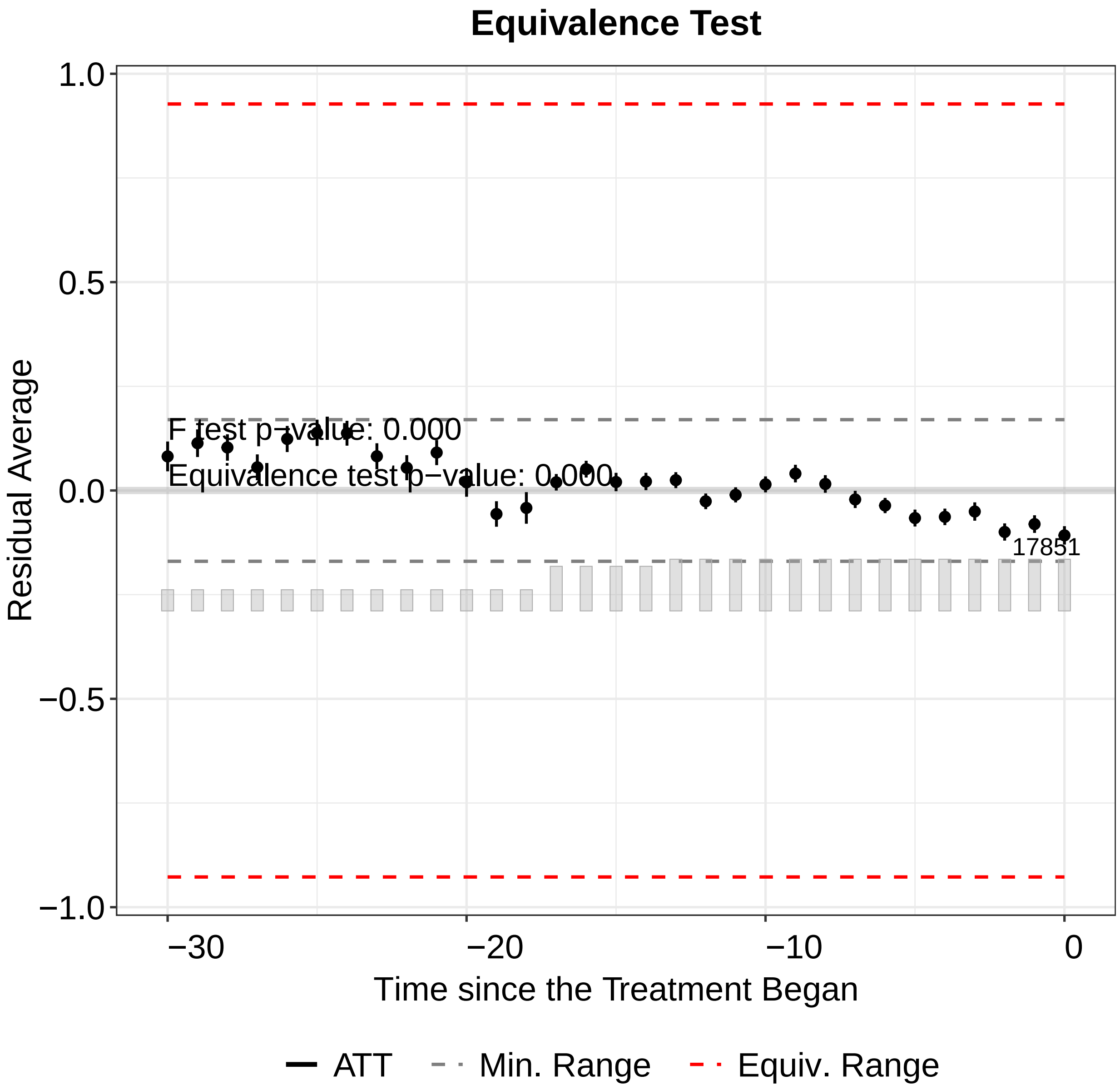

Audience indegree/total indegree

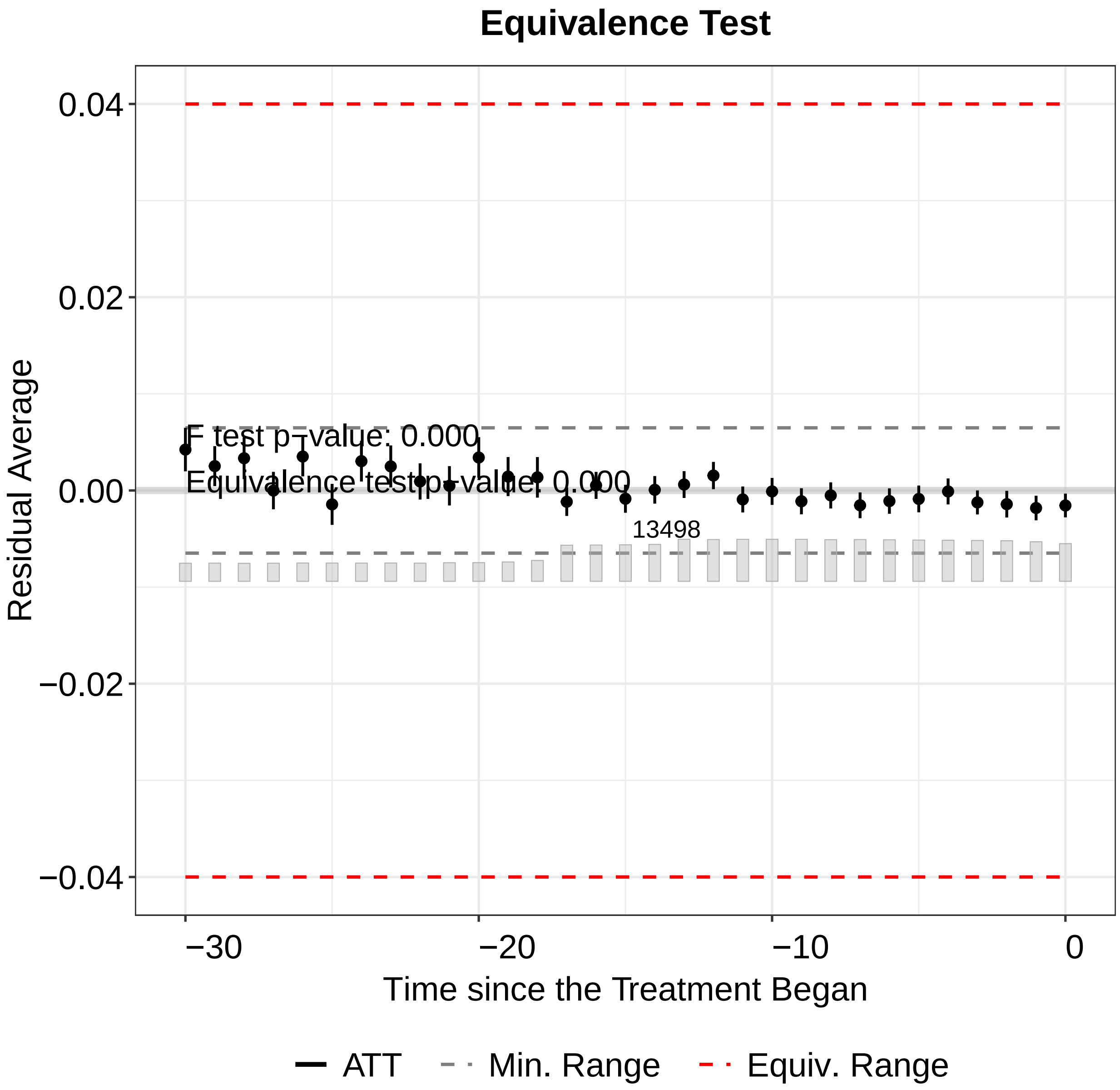

Audience outdegree/total outdegree

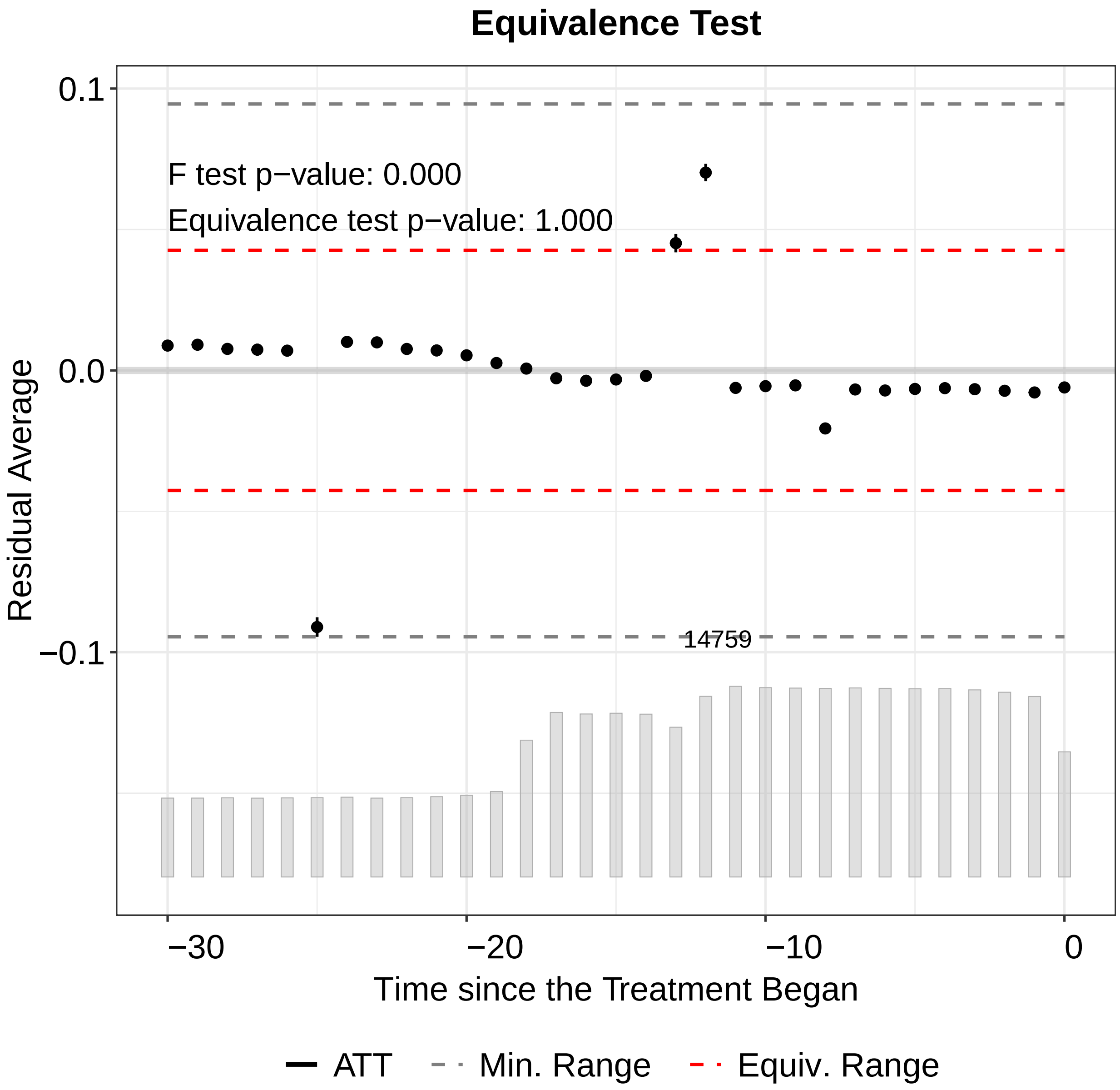

Placebo test results for main outcomes with short time frame and no sampled control groups

Outcome	Placebo p-value	Placebo equivalence p-value
Views on hateful content	6.23036000990673e-06	0
Views on hateful content/total views	0	0
Views on non-organization content that is hateful	1.06404552726591e-05	0
Hateful comments by audience members	0	0
Hateful comments/total comments	0.0248776176039796	0
Hateful audience outdegree	0	0
Hateful non-audience outdegree	0.569233829028701	0
Audience indegree	1.0962936114467e-07	0
Audience outdegree	0	0
Audience indegree/total indegree	0.000198934155375197	0
Audience outdegree/total outdegree	0	0

Tabular results for average ATT for short time frame with no sampled control groups

Table A.8: Effect of disruptions on Views on hateful content

Sample	ATT.avg	S.E.	CI.lower	CI.upper	p.value
Full Data	-0.4026167	0.0446714	-0.4901710	-0.3150623	0.000000
Most views on organization	0.3608362	0.1027127	0.1595230	0.5621494	0.000443
Middle views on organization	-0.4445142	0.0675101	-0.5768315	-0.3121968	0.000000
Least views on organization	-0.7884671	0.0771311	-0.9396414	-0.6372929	0.000000

Table A.9: Effect of disruptions on Views on hateful content/total views

Sample	ATT.avg	S.E.	CI.lower	CI.upper	p.value
Full Data	0.0000007	0.0000841	-0.0001642	0.0001656	0.9931567
Most views on organization	0.0008897	0.0001470	0.0006015	0.0011778	0.0000000
Middle views on organization	0.0000993	0.0001174	-0.0001308	0.0003293	0.3977487
Least views on organization	-0.0007264	0.0001525	-0.0010253	-0.0004275	0.0000019

Table A.10: Effect of disruptions on Hateful comments by audience members

Sample	ATT.avg	S.E.	CI.lower	CI.upper	p.value
Full Data	-0.0252038	0.0144227	-0.0534718	0.0030642	0.0805495
Most views on organization	0.1011454	0.0272152	0.0478045	0.1544862	0.0002020
Middle views on organization	-0.0180461	0.0187338	-0.0547636	0.0186714	0.3354004
Least views on organization	-0.1140474	0.0281606	-0.1692411	-0.0588537	0.0000512

Table A.11: Effect of disruptions on Hateful comments/total comments

Sample	ATT.avg	S.E.	CI.lower	CI.upper	p.value
Full Data	0.0002060	0.0004598	-0.0006951	0.0011072	0.6540582
Most views on organization	0.0017742	0.0009969	-0.0001797	0.0037282	0.0751301
Middle views on organization	0.0003132	0.0007596	-0.0011755	0.0018020	0.6800649
Least views on organization	-0.0010006	0.0007507	-0.0024719	0.0004707	0.1825389

Table A.12: Effect of disruptions on Views on non-organization content that is hateful

Sample	ATT.avg	S.E.	CI.lower	CI.upper	p.value
Full Data	-0.4081367	0.0435787	-0.4935495	-0.3227239	0.0000000
Most views on organization	0.3439099	0.1099919	0.1283298	0.5594899	0.0017679
Middle views on organization	-0.4489917	0.0658521	-0.5780594	-0.3199239	0.0000000
Least views on organization	-0.7883808	0.0795583	-0.9443121	-0.6324494	0.0000000

Table A.13: Effect of disruptions on Audience outdegree

Sample	ATT.avg	S.E.	CI.lower	CI.upper	p.value
Full Data	-0.1800894	0.0229127	-0.2249974	-0.1351813	0.0000000
Most views on organization	0.1644274	0.0602424	0.0463544	0.2825004	0.0063444
Middle views on organization	-0.1885656	0.0316152	-0.2505302	-0.1266010	0.0000000
Least views on organization	-0.4000417	0.0305189	-0.4598576	-0.3402258	0.0000000

Table A.14: Effect of disruptions on Audience indegree

Sample	ATT.avg	S.E.	CI.lower	CI.upper	p.value
Full Data	-0.2071472	0.0378997	-0.2814293	-0.1328652	0.0000000
Most views on organization	0.2050177	0.1007227	0.0076048	0.4024307	0.0418040
Middle views on organization	-0.2193103	0.0580175	-0.3330226	-0.1055980	0.0001568
Least views on organization	-0.4672208	0.0454231	-0.5562485	-0.3781931	0.0000000

Table A.15: Effect of disruptions on Hateful audience outdegree

Sample	ATT.avg	S.E.	CI.lower	CI.upper	p.value
Full Data	-0.0024659	0.0030129	-0.0083710	0.0034392	0.4130968
Most views on organization	0.0066441	0.0145640	-0.0219009	0.0351891	0.6482477
Middle views on organization	-0.0053615	0.0037678	-0.0127464	0.0020233	0.1547438
Least views on organization	-0.0299857	0.0036969	-0.0372315	-0.0227398	0.0000000

Table A.16: Effect of disruptions on Hateful non-audience outdegree

Sample	ATT.avg	S.E.	CI.lower	CI.upper	p.value
Full Data	0.0002815	0.0816046	-0.1596606	0.1602236	0.9972478
Most views on organization	0.0436071	0.0270035	-0.0093189	0.0965330	0.1063399
Middle views on organization	-0.0207245	0.0339628	-0.0872903	0.0458413	0.5417206
Least views on organization	-0.1736932	0.0318226	-0.2360643	-0.1113221	0.0000000

Table A.17: Effect of disruptions on Audience indegree/total indegree

Sample	ATT.avg	S.E.	CI.lower	CI.upper	p.value
Full Data	-0.0063571	0.0005771	-0.0074882	-0.0052260	0.0000000
Most views on organization	-0.0040284	0.0014453	-0.0068612	-0.0011957	0.0053153
Middle views on organization	-0.0055099	0.0008766	-0.0072280	-0.0037917	0.0000000
Least views on organization	-0.0090988	0.0007989	-0.0106646	-0.0075330	0.0000000

Table A.18: Effect of disruptions on Audience outdegree/total outdegree

Sample	ATT.avg	S.E.	CI.lower	CI.upper	p.value
Full Data	-0.0122419	0.0006026	-0.0134229	-0.0110609	0
Most views on organization	-0.0077856	0.0013453	-0.0104223	-0.0051488	0
Middle views on organization	-0.0116771	0.0008718	-0.0133857	-0.0099685	0
Least views on organization	-0.0162708	0.0008683	-0.0179727	-0.0145690	0

Effects over time plots for all manuscript outcomes

Consumption

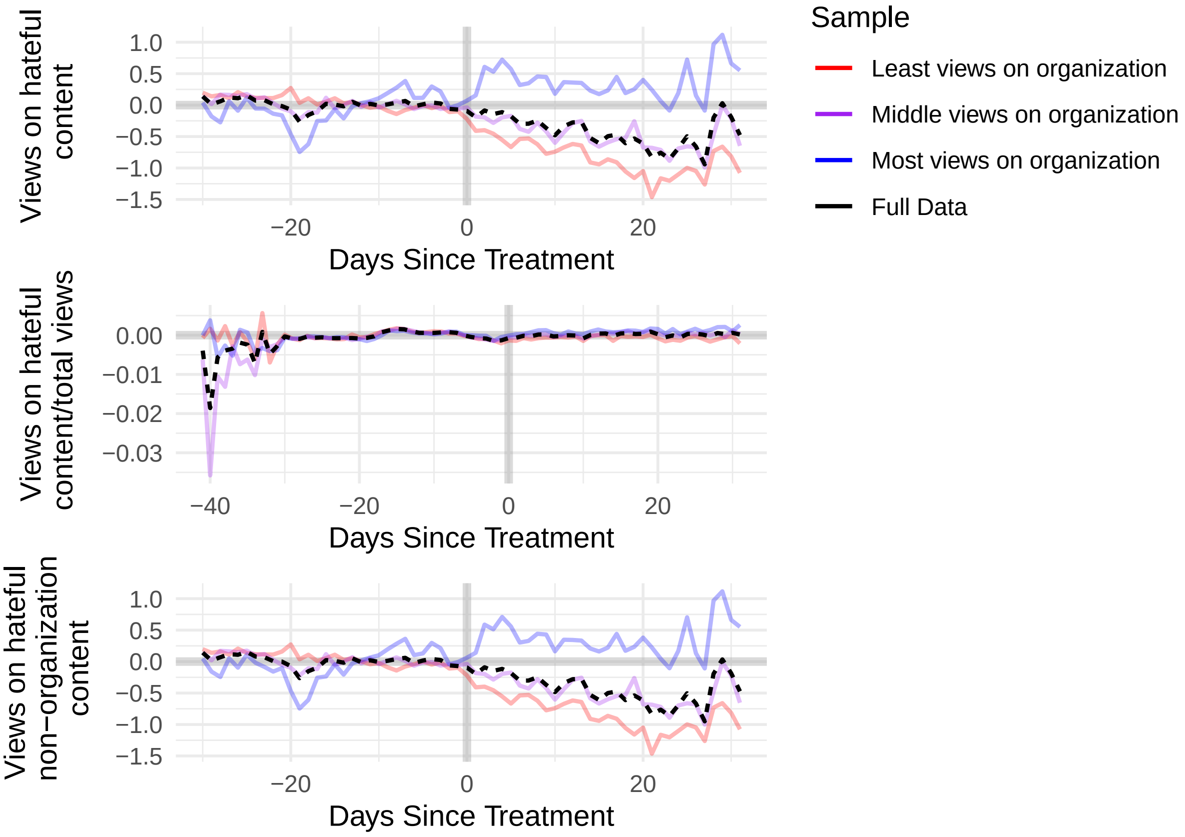

Production

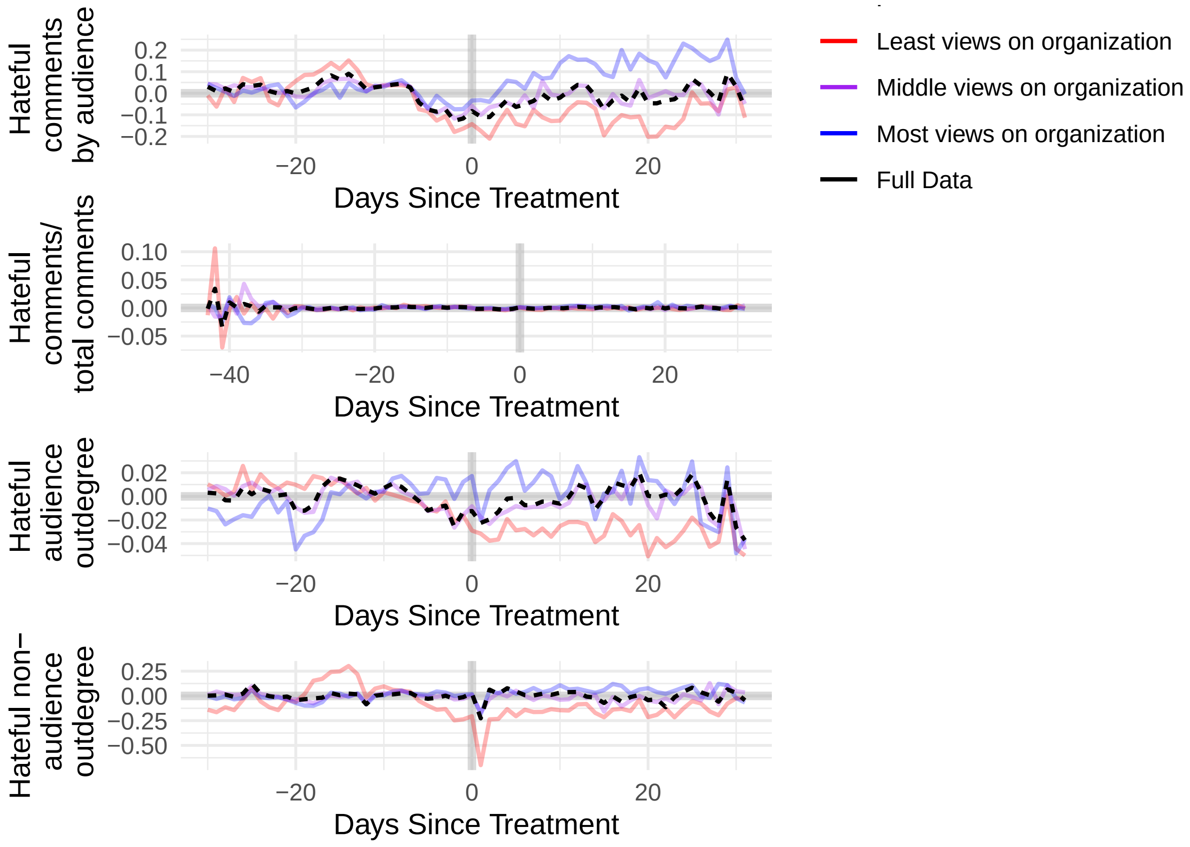
Hateful comments by audience
0.2
0.1
0.0
−0.1
−0.2
−20
0
20
Days Since Treatment
Hateful comments/ total comments
0.10
0.05
0.00
−0.05
−40
−20
0
20
Days Since Treatment
Hateful audience outdegree
0.02
0.00
−0.02
−0.04
−20
0
20
Days Since Treatment
Hateful non−audience outdegree
0.25
0.00
−0.25
−0.50
−20
0
20
Days Since Treatment
Least views on organization
Middle views on organization
Most views on organization
Full Data

Network behavior

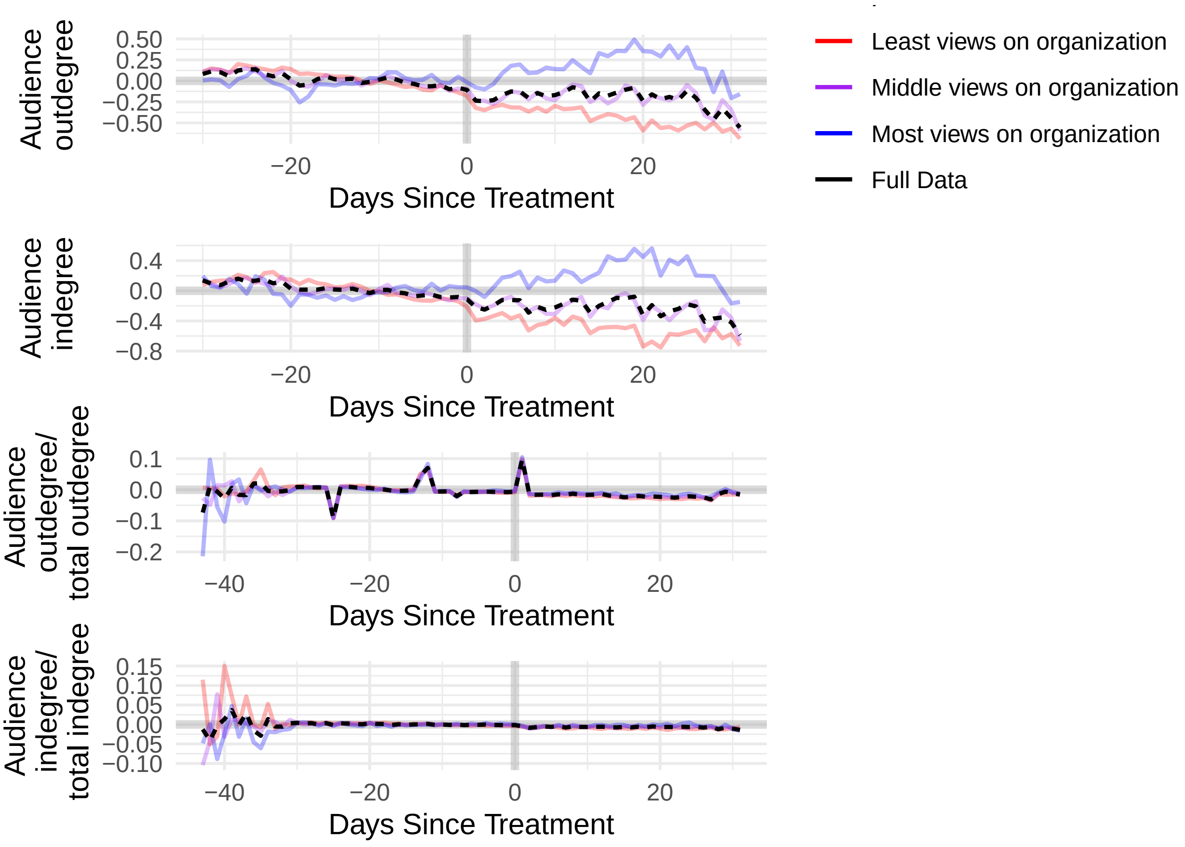

Effect of disruptions on consumption and production of ideologically aligned content

Table A.19: Effect of disruptions on Ideologically aligned terms views

Sample	ATT.avg	S.E.	CI.lower	CI.upper	p.value
Full Data	-0.1222066	0.0165703	-0.1546837	-0.0897295	0.0000000
Most views on organization	0.0927612	0.0389955	0.0163315	0.1691909	0.0173705
Middle views on organization	-0.1540366	0.0258974	-0.2047946	-0.1032786	0.0000000
Least views on organization	-0.2244562	0.0250164	-0.2734875	-0.1754249	0.0000000

Table A.20: Effect of disruptions on Ideologically aligned terms views/Total views

Sample	ATT.avg	S.E.	CI.lower	CI.upper	p.value
Full Data	-0.0000455	0.0000492	-0.0001420	5.10e-05	0.3555419
Most views on organization	0.0003666	0.0000839	0.0002021	5.31e-04	0.0000125
Middle views on organization	-0.0001290	0.0000702	-0.0002666	8.60e-06	0.0661957
Least views on organization	-0.0002332	0.0001002	-0.0004296	-3.69e-05	0.0198882

Table A.21: Effect of disruptions on Ideologically aligned comments by audience members

Sample	ATT.avg	S.E.	CI.lower	CI.upper	p.value
Full Data	-0.0061836	0.0030674	-0.0121956	-0.0001716	0.0438097
Most views on organization	0.0207883	0.0058222	0.0093770	0.0321996	0.0003563
Middle views on organization	-0.0011577	0.0041969	-0.0093834	0.0070680	0.7826648
Least views on organization	-0.0291491	0.0056645	-0.0402513	-0.0180470	0.0000003

Table A.22: Effect of disruptions on Ideologically aligned comments/total comments

Sample	ATT.avg	S.E.	CI.lower	CI.upper	p.value
Full Data	0.0000747	0.0001921	-0.0003018	0.0004511	0.6974845
Most views on organization	0.0006570	0.0004793	-0.0002824	0.0015965	0.1704491
Middle views on organization	0.0001561	0.0002940	-0.0004201	0.0007323	0.5953552
Least views on organization	-0.0004184	0.0003059	-0.0010180	0.0001812	0.1714664

Effect of disruptions on total engagement

Table A.23: Effect of disruptions on Total views of content

Sample	ATT.avg	S.E.	CI.lower	CI.upper	p.value
Full Data	-21.702596	1.886544	-25.40015	-18.005038	0.0000000
Most views on organization	-6.290998	4.788375	-15.67604	3.094045	0.1889114
Middle views on organization	-19.719365	3.053820	-25.70474	-13.733987	0.0000000
Least views on organization	-34.535401	2.965923	-40.34850	-28.722299	0.0000000

Table A.24: Effect of disruptions on Total outdegree

Sample	ATT.avg	S.E.	CI.lower	CI.upper	p.value
Full Data	-1.2662548	0.1595925	-1.5790505	-0.9534592	0.0000000
Most views on organization	0.7193552	0.3033516	0.1247971	1.3139134	0.0177228
Middle views on organization	-1.0355603	0.2163987	-1.4596941	-0.6114266	0.0000017
Least views on organization	-2.8253224	0.3174818	-3.4475753	-2.2030696	0.0000000

Table A.25: Effect of disruptions on Total indegree

Sample	ATT.avg	S.E.	CI.lower	CI.upper	p.value
Full Data	-4.197962	1.7650889	-7.6574729	-0.7384517	0.0173913
Most views on organization	1.028719	0.6646712	-0.2740125	2.3314506	0.1216918
Middle views on organization	-2.046944	0.9688723	-3.9458987	-0.1479892	0.0346258
Least views on organization	-9.701019	4.5699625	-18.6579814	-0.7440576	0.0337724

Tabular results for average ATT for short time frame with sampled control groups

Table A.26: Effect of disruptions on Views on hateful content

Sample	ATT.avg	S.E.	CI.lower	CI.upper	p.value
Full Data	-0.3151528	0.0384450	-0.3905036	-0.2398020	0e+00
Most views on organization	0.4315875	0.0848818	0.2652222	0.5979528	4e-07
Middle views on organization	-0.2737100	0.0480621	-0.3679100	-0.1795099	0e+00
Least views on organization	-0.5537361	0.0523091	-0.6562601	-0.4512121	0e+00

Table A.27: Effect of disruptions on Views on hateful content/total views

Sample	ATT.avg	S.E.	CI.lower	CI.upper	p.value
Full Data	0.0000238	0.0000707	-0.0001147	0.0001623	0.7363851
Most views on organization	0.0009046	0.0001311	0.0006476	0.0011616	0.0000000
Middle views on organization	0.0001031	0.0001028	-0.0000985	0.0003046	0.3161863
Least views on organization	-0.0005286	0.0001363	-0.0007957	-0.0002615	0.0001050

Table A.28: Effect of disruptions on Views on non-organization content that is hateful

Sample	ATT.avg	S.E.	CI.lower	CI.upper	p.value
Full Data	-0.3193179	0.0362052	-0.3902787	-0.2483570	0e+00
Most views on organization	0.4291235	0.0847520	0.2630125	0.5952344	4e-07
Middle views on organization	-0.2765560	0.0479759	-0.3705870	-0.1825251	0e+00
Least views on organization	-0.5536216	0.0523076	-0.6561426	-0.4511006	0e+00

Table A.29: Effect of disruptions on Hateful comments by audience members

Sample	ATT.avg	S.E.	CI.lower	CI.upper	p.value
Full Data	-0.0257379	0.0131199	-0.0514525	-0.0000234	0.0497921
Most views on organization	0.0689081	0.0225802	0.0246518	0.1131644	0.0022754
Middle views on organization	-0.0224675	0.0163076	-0.0544298	0.0094947	0.1682847
Least views on organization	-0.0944791	0.0242901	-0.1420868	-0.0468715	0.0001004

Table A.30: Effect of disruptions on Hateful comments/total comments

Sample	ATT.avg	S.E.	CI.lower	CI.upper	p.value
Full Data	0.0002634	0.0004476	-0.0006140	0.0011408	0.5562681
Most views on organization	0.0017740	0.0009266	-0.0000421	0.0035901	0.0555587
Middle views on organization	0.0004418	0.0006900	-0.0009106	0.0017942	0.5219534
Least views on organization	-0.0007216	0.0007537	-0.0021988	0.0007556	0.3383677

Table A.31: Effect of disruptions on Hateful audience outdegree

Sample	ATT.avg	S.E.	CI.lower	CI.upper	p.value
Full Data	-0.0031727	0.0025944	-0.0082575	0.0019122	0.2213594
Most views on organization	0.0135324	0.0088783	-0.0038688	0.0309335	0.1274572
Middle views on organization	-0.0065904	0.0031396	-0.0127440	-0.0004368	0.0358078
Least views on organization	-0.0246268	0.0031850	-0.0308693	-0.0183843	0.0000000

Table A.32: Effect of disruptions on Hateful non-audience outdegree

Sample	ATT.avg	S.E.	CI.lower	CI.upper	p.value
Full Data	-0.0034409	0.0626131	-0.1261604	0.1192786	0.9561749
Most views on organization	0.0920413	0.0243350	0.0443455	0.1397371	0.0001554
Middle views on organization	-0.0161645	0.0176384	-0.0507351	0.0184061	0.3594364
Least views on organization	-0.1479424	0.0263248	-0.1995382	-0.0963467	0.0000000

Table A.33: Effect of disruptions on Audience indegree

Sample	ATT.avg	S.E.	CI.lower	CI.upper	p.value
Full Data	-0.2003304	0.0333413	-0.2656782	-0.1349826	0.0000000
Most views on organization	0.0727585	0.0675733	-0.0596827	0.2051997	0.2815989
Middle views on organization	-0.1948741	0.0479748	-0.2889029	-0.1008453	0.0000487
Least views on organization	-0.3918253	0.0362949	-0.4629621	-0.3206885	0.0000000

Table A.34: Effect of disruptions on Audience outdegree

Sample	ATT.avg	S.E.	CI.lower	CI.upper	p.value
Full Data	-0.1762565	0.0192446	-0.2139752	-0.1385378	0.0000000
Most views on organization	0.0265541	0.0421107	-0.0559813	0.1090896	0.5283158
Middle views on organization	-0.1767287	0.0240067	-0.2237810	-0.1296764	0.0000000
Least views on organization	-0.3270059	0.0252425	-0.3764802	-0.2775316	0.0000000

Table A.35: Effect of disruptions on Audience indegree/total indegree

Sample	ATT.avg	S.E.	CI.lower	CI.upper	p.value
Full Data	-0.0063214	0.0005768	-0.0074518	-0.0051909	0.0000000
Most views on organization	-0.0047866	0.0013758	-0.0074832	-0.0020901	0.0005031
Middle views on organization	-0.0053820	0.0007966	-0.0069433	-0.0038207	0.0000000
Least views on organization	-0.0086546	0.0007325	-0.0100903	-0.0072189	0.0000000

Table A.36: Effect of disruptions on Audience outdegree/total outdegree

Sample	ATT.avg	S.E.	CI.lower	CI.upper	p.value
Full Data	-0.0120886	0.0005976	-0.0132599	-0.0109173	0
Most views on organization	-0.0092987	0.0013298	-0.0119050	-0.0066924	0
Middle views on organization	-0.0112862	0.0008514	-0.0129548	-0.0096175	0
Least views on organization	-0.0149279	0.0008220	-0.0165389	-0.0133169	0

Tabular results for average ATT for long timeframe with sampled control groups

Table A.37: Effect of disruptions on Views on hateful content

Sample	ATT.avg	S.E.	CI.lower	CI.upper	p.value
Full Data	-0.7989785	0.0328001	-0.8632655	-0.7346916	0.0e+00
Most views on organization	-0.7140833	0.1340065	-0.9767313	-0.4514354	1.0e-07
Middle views on organization	-0.7381267	0.0478459	-0.8319030	-0.6443504	0.0e+00
Least views on organization	-0.7093820	0.1585811	-1.0201952	-0.3985688	7.7e-06

Table A.38: Effect of disruptions on Views on hateful content/total views

Sample	ATT.avg	S.E.	CI.lower	CI.upper	p.value
Full Data	-0.0010697	0.0000844	-0.0012352	-0.0009042	0
Most views on organization	-0.0007302	0.0001106	-0.0009470	-0.0005134	0
Middle views on organization	-0.0009248	0.0000901	-0.0011014	-0.0007483	0
Least views on organization	-0.0014009	0.0001142	-0.0016248	-0.0011770	0

Table A.39: Effect of disruptions on Views on non-organization content that is hateful

Sample	ATT.avg	S.E.	CI.lower	CI.upper	p.value
Full Data	-0.7979036	0.0335466	-0.8636537	-0.7321535	0.0e+00
Most views on organization	-0.7015842	0.1288960	-0.9542157	-0.4489527	1.0e-07
Middle views on organization	-0.7383135	0.0512270	-0.8387166	-0.6379105	0.0e+00
Least views on organization	-0.7092799	0.1549143	-1.0129063	-0.4056536	4.7e-06

Table A.40: Effect of disruptions on Hateful comments by audience members

Sample	ATT.avg	S.E.	CI.lower	CI.upper	p.value
Full Data	-0.0777296	0.0092045	-0.0957702	-0.0596890	0.0000000
Most views on organization	-0.0259417	0.0141088	-0.0535945	0.0017110	0.0659608
Middle views on organization	-0.0559581	0.0103389	-0.0762218	-0.0356943	0.0000001
Least views on organization	-0.1399490	0.0186703	-0.1765422	-0.1033559	0.0000000

Table A.41: Effect of disruptions on Hateful comments/total comments

Sample	ATT.avg	S.E.	CI.lower	CI.upper	p.value
Full Data	-0.0010250	0.0005109	-0.0020264	-0.0000236	0.0448351
Most views on organization	0.0001657	0.0007276	-0.0012604	0.0015918	0.8198733
Middle views on organization	-0.0007232	0.0006203	-0.0019389	0.0004926	0.2436730
Least views on organization	-0.0020174	0.0006465	-0.0032846	-0.0007503	0.0018059

Table A.42: Effect of disruptions on Hateful audience outdegree

Sample	ATT.avg	S.E.	CI.lower	CI.upper	p.value
Full Data	-0.0195047	0.0016070	-0.0226544	-0.0163551	0
Most views on organization	-0.0319411	0.0055755	-0.0428688	-0.0210133	0
Middle views on organization	-0.0173176	0.0020675	-0.0213699	-0.0132654	0
Least views on organization	-0.0253511	0.0019695	-0.0292113	-0.0214909	0

Table A.43: Effect of disruptions on Hateful non-audience outdegree

Sample	ATT.avg	S.E.	CI.lower	CI.upper	p.value
Full Data	-0.1447911	0.0242806	-0.1923802	-0.0972020	0.0000000
Most views on organization	-0.0502146	0.0162224	-0.0820099	-0.0184193	0.0019656
Middle views on organization	-0.0716185	0.0157570	-0.1025017	-0.0407354	0.0000055
Least views on organization	-0.0918812	0.0640820	-0.2174796	0.0337171	0.1516271

Table A.44: Effect of disruptions on Audience indegree

Sample	ATT.avg	S.E.	CI.lower	CI.upper	p.value
Full Data	-0.2615763	0.0212380	-0.3032021	-0.2199505	0
Most views on organization	-0.2598409	0.0436936	-0.3454787	-0.1742030	0
Middle views on organization	-0.2281616	0.0315318	-0.2899628	-0.1663603	0
Least views on organization	-0.3126848	0.0238514	-0.3594327	-0.2659368	0

Table A.45: Effect of disruptions on Audience outdegree

Sample	ATT.avg	S.E.	CI.lower	CI.upper	p.value
Full Data	-0.2540554	0.0123114	-0.2781853	-0.2299255	0
Most views on organization	-0.3228661	0.0288956	-0.3795004	-0.2662317	0
Middle views on organization	-0.2198782	0.0166161	-0.2524453	-0.1873112	0
Least views on organization	-0.2678722	0.0150458	-0.2973615	-0.2383830	0

Table A.46: Effect of disruptions on Audience indegree/total indegree

Sample	ATT.avg	S.E.	CI.lower	CI.upper	p.value
Full Data	-0.0074826	0.0004312	-0.0083277	-0.0066375	0
Most views on organization	-0.0101620	0.0008893	-0.0119050	-0.0084189	0
Middle views on organization	-0.0063567	0.0006086	-0.0075496	-0.0051639	0
Least views on organization	-0.0072777	0.0005123	-0.0082818	-0.0062735	0

Table A.47: Effect of disruptions on Audience outdegree/total outdegree

Sample	ATT.avg	S.E.	CI.lower	CI.upper	p.value
Full Data	-0.0149596	0.0003735	-0.0156916	-0.0142276	0
Most views on organization	-0.0181786	0.0007743	-0.0196962	-0.0166610	0
Middle views on organization	-0.0139031	0.0005685	-0.0150175	-0.0127888	0
Least views on organization	-0.0141052	0.0005299	-0.0151438	-0.0130665	0

Effects over time plots for all manuscript outcomes for long timeframe with sampled control groups

Consumption

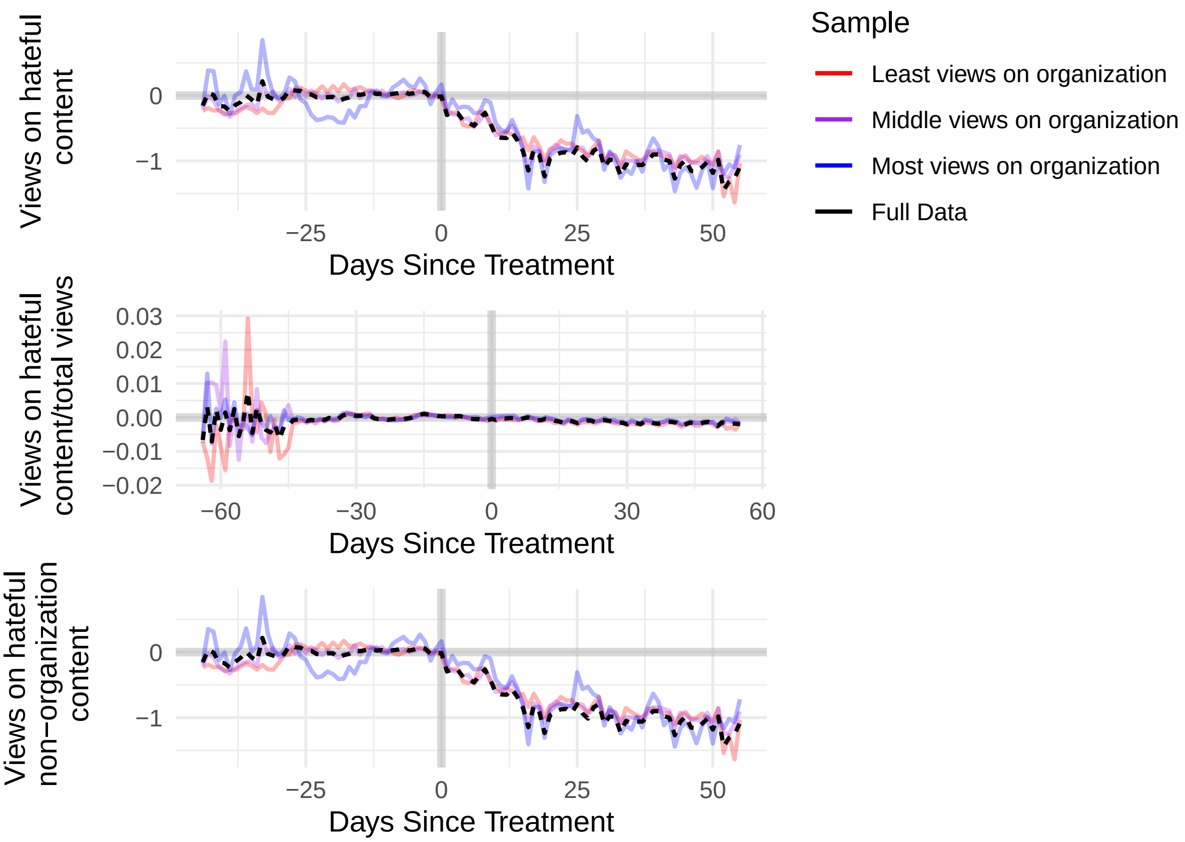

Production

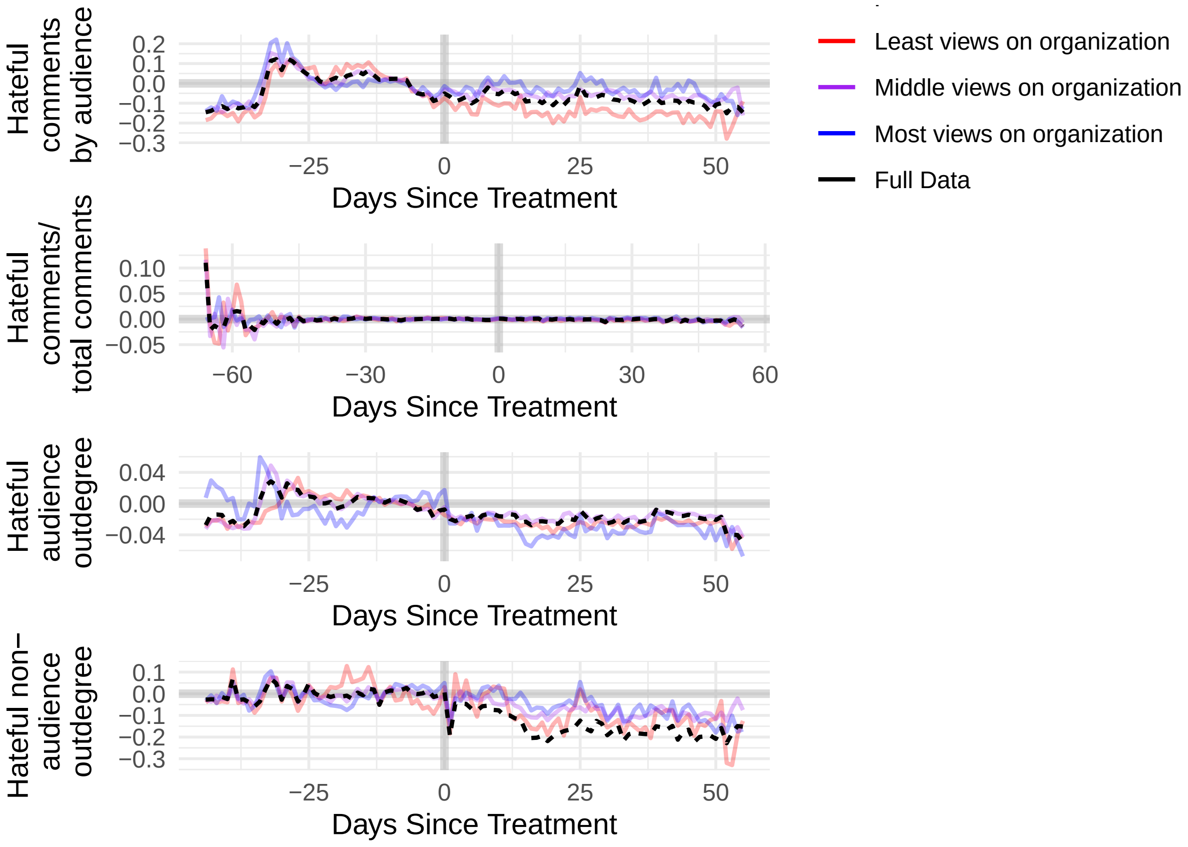
Hateful comments by audience
0.2
0.1
0.0
−0.1
−0.2
−0.3
−25
0
25
50
Days Since Treatment
Hateful comments/ total comments
0.10
0.05
0.00
−0.05
−60
−30
0
30
60
Days Since Treatment
Hateful audience outdegree
0.04
0.00
−0.04
−25
0
25
50
Days Since Treatment
Hateful non-audience outdegree
0.1
0.0
−0.1
−0.2
−0.3
−25
0
25
50
Days Since Treatment
Least views on organization
Middle views on organization
Most views on organization
Full Data

Network behavior

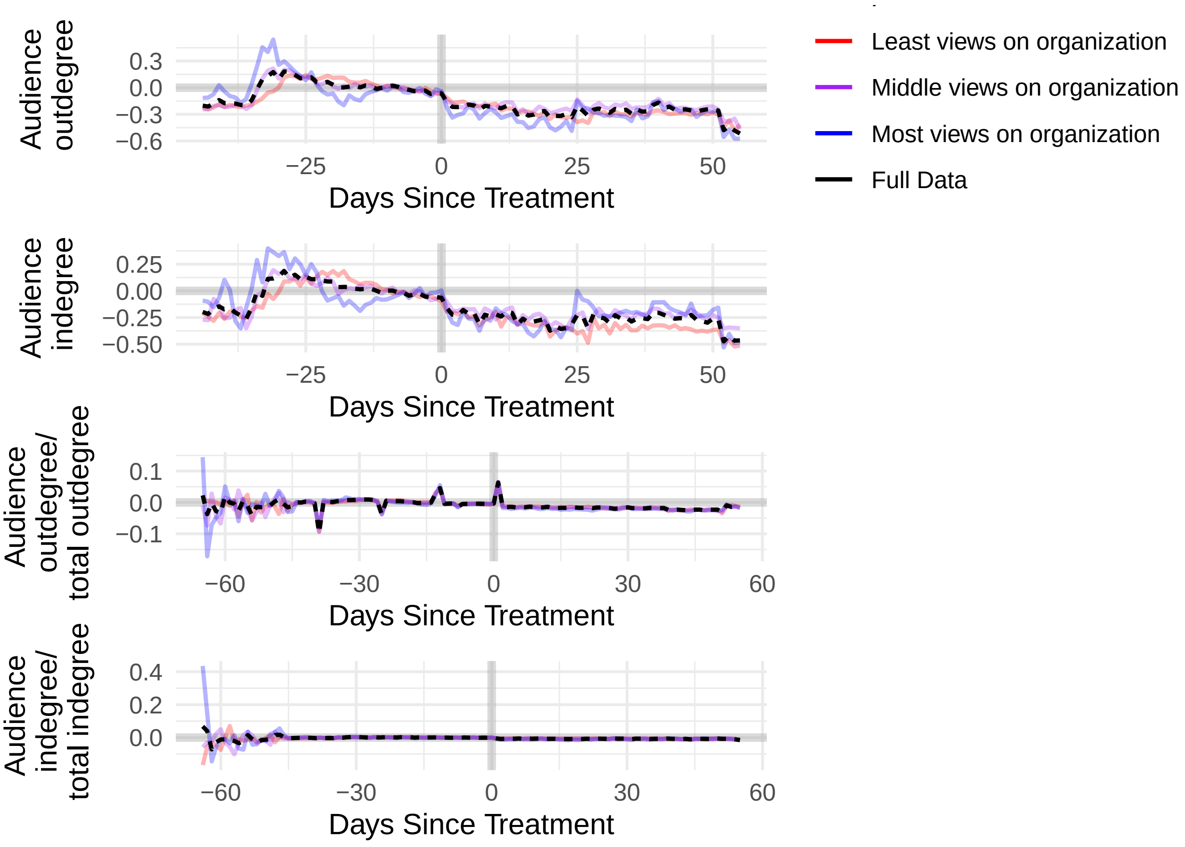

Two Way Fixed Effects (OLS) for manuscript outcomes for short time frame with no sampled control groups

Table A.48: Two way fixed effects OLS estimates for Views on hate content with short time frame sample

Sample	Estimate	Std..Error	t.value	Pr...t..	nobs
Full Data	-0.1990560	0.1066475	-1.866486	0.1209584	1186155
Most views on organization	0.3907787	0.2421269	1.613941	0.1674601	264915
Middle views on organization	-0.2455907	0.0804987	-3.050866	0.0283962	499230
Least views on organization	-0.5689806	0.1711436	-3.324581	0.0209029	422010

Table A.49: Two way fixed effects OLS estimates for Views on hate content/total views with short time frame sample

Sample	Estimate	Std..Error	t.value	Pr...t..	nobs
Full Data	-0.0000834	0.0002687	-0.3103631	0.7688050	1122349
Most views on organization	0.0008187	0.0004249	1.9268320	0.1119439	257132
Middle views on organization	0.0000011	0.0003140	0.0036426	0.9972345	480312
Least views on organization	-0.0008192	0.0002474	-3.3114352	0.0212069	384905

Table A.50: Two way fixed effects OLS estimates for Views on non-organization content that is hateful with short time frame sample

Sample	Estimate	Std..Error	t.value	Pr...t..	nobs
Full Data	-0.2032864	0.1084142	-1.875089	0.1196288	1186155
Most views on organization	0.3800011	0.2379185	1.597190	0.1711134	264915
Middle views on organization	-0.2489584	0.0810974	-3.069869	0.0277880	499230
Least views on organization	-0.5688594	0.1710978	-3.324762	0.0208987	422010

Table A.51: Two way fixed effects OLS estimates for Hateful comments by audience members with short time frame sample

Sample	Estimate	Std..Error	t.value	Pr...t..	nobs
Full Data	-0.0297189	0.0237445	-1.251613	0.2660752	1186155
Most views on organization	0.0774399	0.0229021	3.381343	0.0196455	264915
Middle views on organization	-0.0205614	0.0189886	-1.082826	0.3283189	499230
Least views on organization	-0.1128372	0.0476145	-2.369807	0.0639680	422010

Table A.52: Two way fixed effects OLS estimates for Hateful comments/total comments with short time frame sample

Sample	Estimate	Std..Error	t.value	Pr...t..	nobs
Full Data	-0.0001676	0.0004982	-0.3364560	0.7501884	743156
Most views on organization	0.0022326	0.0015330	1.4563419	0.2050749	172521
Middle views on organization	-0.0001556	0.0002919	-0.5330512	0.6168301	299120
Least views on organization	-0.0018622	0.0005742	-3.2431208	0.0228698	271515

Table A.53: Two way fixed effects OLS estimates for Hateful audience outdegree with short time frame sample

Sample	Estimate	Std..Error	t.value	Pr...t..	nobs
Full Data	-0.0046072	0.0047561	-0.9686978	0.3771864	1186155
Most views on organization	0.0342608	0.0177816	1.9267589	0.1119544	264915
Middle views on organization	-0.0075116	0.0040516	-1.8539812	0.1229180	499230
Least views on organization	-0.0285906	0.0093054	-3.0724673	0.0277059	422010

Table A.54: Two way fixed effects OLS estimates for Hateful non-audience outdegree with short time frame sample

Sample	Estimate	Std..Error	t.value	Pr...t..	nobs
Full Data	-0.0532186	0.0216565	-2.457393	0.0574122	1186155
Most views on organization	0.0810481	0.0521654	1.553675	0.1809743	264915
Middle views on organization	-0.0207713	0.0162307	-1.279752	0.2567946	499230
Least views on organization	-0.1819459	0.0358169	-5.079886	0.0038348	422010

Table A.55: Two way fixed effects OLS estimates for Audience indegree with short time frame sample

Sample	Estimate	Std..Error	t.value	Pr...t..	nobs
Full Data	-0.1907099	0.0734890	-2.595080	0.0485375	1186155
Most views on organization	0.1397435	0.0738916	1.891197	0.1171798	264915
Middle views on organization	-0.1955748	0.0809642	-2.415572	0.0604468	499230
Least views on organization	-0.4144298	0.1452488	-2.853240	0.0356879	422010

Table A.56: Two way fixed effects OLS estimates for Audience outdegree with short time frame sample

Sample	Estimate	Std..Error	t.value	Pr...t..	nobs
Full Data	-0.1659381	0.0655338	-2.532100	0.0523957	1186155
Most views on organization	0.1123000	0.0989031	1.135454	0.3076642	264915
Middle views on organization	-0.1745024	0.0630847	-2.766161	0.0395448	499230
Least views on organization	-0.3514164	0.1306717	-2.689308	0.0433345	422010

Table A.57: Two way fixed effects OLS estimates for Audience indegree/total indegree with short time frame sample

Sample	Estimate	Std..Error	t.value	Pr...t..	nobs
Full Data	-0.0054320	0.0013118	-4.140845	0.0089897	916080
Most views on organization	-0.0027445	0.0015983	-1.717167	0.1465965	209509
Middle views on organization	-0.0046203	0.0015691	-2.944613	0.0320848	375263
Least views on organization	-0.0091136	0.0027116	-3.361013	0.0200857	331308

Table A.58: Two way fixed effects OLS estimates for Audience outdegree/total outdegree with short time frame sample

Sample	Estimate	Std..Error	t.value	Pr...t..	nobs
Full Data	-0.0044286	0.0070695	-0.6264301	0.5585174	985292
Most views on organization	-0.0005328	0.0052642	-0.1012202	0.9233094	227333
Middle views on organization	-0.0036095	0.0070674	-0.5107227	0.6312882	413933
Least views on organization	-0.0094451	0.0086688	-1.0895532	0.3256137	344026

Alternative Two Way Fixed Effects (OLS) models for ratio outcomes

In the manuscript, we present results for ratio outcomes, where observations are missing when the denominator is 0. Here, we present results for models where the numerator of the ratios is the outcome, and the denominator enters as a covariate. Results are largely consistent with the ratio approach.

Table A.59: Two way fixed effects OLS estimates for Views on hate content with short time frame sample, controlling for total views

Sample	Estimate	Std..Error	t.value	Pr...t..	nobs
Full Data	-0.0005002	0.1156095	-0.0043262	0.9967155	1186155
Most views on organization	0.4802098	0.2371796	2.0246671	0.0987806	264915
Middle views on organization	-0.0830893	0.0730298	-1.1377454	0.3067912	499230
Least views on organization	-0.2914535	0.0989491	-2.9454886	0.0320523	422010

Table A.60: Two way fixed effects OLS estimates for Hateful comments by audience members with short time frame sample, controlling for total comments

Sample	Estimate	Std..Error	t.value	Pr...t..	nobs
Full Data	0.0204585	0.0107410	1.9047071	0.1151656	1186155
Most views on organization	0.0710032	0.0188401	3.7687354	0.0130399	264915
Middle views on organization	0.0128305	0.0068082	1.8845608	0.1181825	499230
Least views on organization	-0.0028961	0.0173356	-0.1670578	0.8738707	422010

Table A.61: Two way fixed effects OLS estimates for Audience indegree with short time frame sample, controlling for total indegree

Sample	Estimate	Std..Error	t.value	Pr...t..	nobs
Full Data	-0.1746829	0.0630675	-2.769776	0.0393758	1186155
Most views on organization	0.1095120	0.0576241	1.900454	0.1157957	264915
Middle views on organization	-0.1678045	0.0811144	-2.068740	0.0933892	499230
Least views on organization	-0.3904689	0.1263690	-3.089910	0.0271622	422010

Table A.62: Two way fixed effects OLS estimates for Audience outdegree with short time frame sample, controlling for total outdegree

Sample	Estimate	Std..Error	t.value	Pr...t..	nobs
Full Data	-0.0944399	0.0413727	-2.282661	0.0713004	1186155
Most views on organization	0.0758254	0.0654636	1.158283	0.2990627	264915
Middle views on organization	-0.1059871	0.0451826	-2.345751	0.0659073	499230
Least views on organization	-0.2774829	0.0983211	-2.822212	0.0370122	422010

OLS with only time fixed effects for short time frame with no sampled control groups

Table A.63: OLS estimates for Views on hate content with short time frame sample and 1-way time fixed effects

Sample	Estimate	Std. Error	t value	Pr(>|t|)	nobs
Full Data	-2.385344	0.6963019	-3.425732	0.0187217	1186155
Most views on organization	-2.867152	0.9881707	-2.901474	0.0337327	264915
Middle views on organization	-2.650097	0.6649459	-3.985432	0.0104739	499230
Least views on organization	-1.742042	0.5224030	-3.334672	0.0206728	422010

Table A.64: OLS estimates for Views on hate content/total views with short time frame sample and 1-way time fixed effects

Sample	Estimate	Std. Error	t value	Pr(>|t|)	nobs
Full Data	-0.0044476	0.0015280	-2.910796	0.0333688	1122349
Most views on organization	-0.0037076	0.0016541	-2.241394	0.0750832	257132
Middle views on organization	-0.0046611	0.0015391	-3.028415	0.0291342	480312
Least views on organization	-0.0045588	0.0015050	-3.029209	0.0291078	384905

Table A.65: OLS estimates for Views on non-organization content that is hateful with short time frame sample and 1-way time fixed effects

Sample	Estimate	Std. Error	t value	Pr(>|t|)	nobs
Full Data	-2.384133	0.6961565	-3.424709	0.0187425	1186155
Most views on organization	-2.864452	0.9878503	-2.899682	0.0338032	264915
Middle views on organization	-2.649000	0.6648280	-3.984488	0.0104838	499230
Least views on organization	-1.741714	0.5223468	-3.334402	0.0206789	422010

Table A.66: OLS estimates for Hateful comments by audience members with short time frame sample and 1-way time fixed effects

Sample	Estimate	Std. Error	t value	Pr(>|t|)	nobs
Full Data	-0.3353320	0.1081065	-3.101866	0.0267965	1186155
Most views on organization	-0.2350926	0.1279616	-1.837212	0.1255971	264915
Middle views on organization	-0.3257870	0.1015308	-3.208751	0.0237618	499230
Least views on organization	-0.4004488	0.1349493	-2.967402	0.0312506	422010

Table A.67: OLS estimates for Hateful comments/total comments with short time frame sample and 1-way time fixed effects

Sample	Estimate	Std. Error	t value	Pr(>|t|)	nobs
Full Data	-0.0129245	0.0038358	-3.369412	0.0199025	743156
Most views on organization	-0.0106277	0.0047787	-2.223958	0.0767454	172521
Middle views on organization	-0.0141045	0.0036659	-3.847444	0.0120323	299120
Least views on organization	-0.0129485	0.0045765	-2.829309	0.0367045	271515

Table A.68: OLS estimates for Hateful audience outdegree with short time frame sample and 1-way time fixed effects

Sample	Estimate	Std. Error	t value	Pr(>|t|)	nobs
Full Data	-0.0871111	0.0244997	-3.555604	0.0162903	1186155
Most views on organization	-0.0996063	0.0398918	-2.496912	0.0546964	264915
Middle views on organization	-0.0898639	0.0216602	-4.148813	0.0089203	499230
Least views on organization	-0.0743013	0.0165479	-4.490079	0.0064583	422010

Table A.69: OLS estimates for Hateful non-audience outdegree with short time frame sample and 1-way time fixed effects

Sample	Estimate	Std. Error	t value	Pr(>|t|)	nobs
Full Data	-0.4220383	0.1411774	-2.989418	0.0304677	1186155
Most views on organization	-0.2111062	0.1253536	-1.684086	0.1529835	264915
Middle views on organization	-0.4188248	0.1136828	-3.684154	0.0142319	499230
Least views on organization	-0.5445824	0.2287567	-2.380618	0.0631166	422010

Table A.70: OLS estimates for Audience indegree with short time frame sample and 1-way time fixed effects

Sample	Estimate	Std. Error	t value	Pr(>|t|)	nobs
Full Data	-0.9053440	0.2682272	-3.375288	0.0197754	1186155
Most views on organization	-1.2585997	0.4275030	-2.944072	0.0321049	264915
Middle views on organization	-0.9433804	0.2831111	-3.332191	0.0207291	499230
Least views on organization	-0.6218708	0.1629610	-3.816072	0.0124228	422010

Table A.71: OLS estimates for Audience outdegree with short time frame sample and 1-way time fixed effects

Sample	Estimate	Std. Error	t value	Pr(>|t|)	nobs
Full Data	-0.9179528	0.2412213	-3.805438	0.0125585	1186155
Most views on organization	-1.2708372	0.3741343	-3.396740	0.0193193	264915
Middle views on organization	-0.9318440	0.2513725	-3.707025	0.0138977	499230
Least views on organization	-0.6532548	0.1443432	-4.525706	0.0062501	422010

Table A.72: OLS estimates for Audience indegree/total indegree with short time frame sample and 1-way time fixed effects

Sample	Estimate	Std. Error	t value	Pr(>|t|)	nobs
Full Data	-0.0183966	0.0049728	-3.699418	0.0140079	916080
Most views on organization	-0.0231040	0.0065133	-3.547186	0.0164366	209509
Middle views on organization	-0.0196003	0.0054423	-3.601492	0.0155188	375263
Least views on organization	-0.0131111	0.0040760	-3.216654	0.0235533	331308

Table A.73: OLS estimates for Audience outdegree/total outdegree with short time frame sample and 1-way time fixed effects

Sample	Estimate	Std. Error	t value	Pr(>|t|)	nobs
Full Data	-0.0171349	0.0118069	-1.4512583	0.2064152	985292
Most views on organization	-0.0261036	0.0153778	-1.6974905	0.1503625	227333
Middle views on organization	-0.0193820	0.0130765	-1.4822041	0.1983847	413933
Least views on organization	-0.0082785	0.0119591	-0.6922303	0.5196183	344026

Two Way Fixed Effects (OLS) for short time frame with p-values from wild cluster bootstrapping

Table A.74: Effect of disruptions on Views on hateful content

Sample	Estimate	Std..Error	t.value	Pr...t..
Full Data	-0.1990560	0.0333643	-5.966141	0e+00
Most views on organization	0.3907787	0.0638648	6.118839	0e+00
Middle views on organization	-0.2455907	0.0460569	-5.332327	1e-07
Least views on organization	-0.5689806	0.0713067	-7.979338	0e+00

Table A.75: Effect of disruptions on Views on hateful content/total views

Sample	Estimate	Std..Error	t.value	Pr...t..
Full Data	-0.0000834	0.0001004	-0.8308849	0.4060388
Most views on organization	0.0008187	0.0000969	8.4491605	0.0000000
Middle views on organization	0.0000011	0.0000930	0.0122944	0.9901907
Least views on organization	-0.0008192	0.0001945	-4.2108405	0.0000254

Table A.76: Effect of disruptions on Views on non-organization content that is hateful

Sample	Estimate	Std..Error	t.value	Pr...t..
Full Data	-0.2032864	0.0321678	-6.319565	0e+00
Most views on organization	0.3800011	0.0615454	6.174323	0e+00
Middle views on organization	-0.2489584	0.0505967	-4.920452	9e-07
Least views on organization	-0.5688594	0.0699426	-8.133234	0e+00

Table A.77: Effect of disruptions on Hateful comments by audience members

Sample	Estimate	Std..Error	t.value	Pr...t..
Full Data	-0.0297189	0.0119448	-2.488011	0.0128461
Most views on organization	0.0774399	0.0117854	6.570820	0.0000000
Middle views on organization	-0.0205614	0.0084137	-2.443793	0.0145341
Least views on organization	-0.1128372	0.0270131	-4.177133	0.0000295

Table A.78: Effect of disruptions on Hateful comments/total comments

Sample	Estimate	Std..Error	t.value	Pr...t..
Full Data	-0.0001676	0.0003679	-0.4555627	0.6487046
Most views on organization	0.0022326	0.0006790	3.2881951	0.0010085
Middle views on organization	-0.0001556	0.0005987	-0.2599051	0.7949371
Least views on organization	-0.0018622	0.0003409	-5.4630702	0.0000000

Table A.79: Effect of disruptions on Hateful audience outdegree

Sample	Estimate	Std..Error	t.value	Pr...t..
Full Data	-0.0046072	0.0013304	-3.463075	0.0005341
Most views on organization	0.0342608	0.0067760	5.056209	0.0000004
Middle views on organization	-0.0075116	0.0017533	-4.284249	0.0000183
Least views on organization	-0.0285906	0.0017682	-16.169179	0.0000000

Table A.80: Effect of disruptions on Hateful non-audience outdegree

Sample	Estimate	Std..Error	t.value	Pr...t..
Full Data	-0.0532186	0.0081273	-6.548167	0.0000000
Most views on organization	0.0810481	0.0170379	4.756931	0.0000020
Middle views on organization	-0.0207713	0.0147252	-1.410598	0.1583638
Least views on organization	-0.1819459	0.0158629	-11.469893	0.0000000

Table A.81: Effect of disruptions on Audience indegree

Sample	Estimate	Std..Error	t.value	Pr...t..
Full Data	-0.1907099	0.0358611	-5.318018	0.0000001
Most views on organization	0.1397435	0.1142809	1.222807	0.2214037
Middle views on organization	-0.1955748	0.0253319	-7.720481	0.0000000
Least views on organization	-0.4144298	0.0331712	-12.493658	0.0000000

Table A.82: Effect of disruptions on Audience outdegree

Sample	Estimate	Std..Error	t.value	Pr...t..
Full Data	-0.1659381	0.0264880	-6.264645	0.0000000
Most views on organization	0.1123000	0.0524835	2.139721	0.0323783
Middle views on organization	-0.1745024	0.0203001	-8.596134	0.0000000
Least views on organization	-0.3514164	0.0241938	-14.525059	0.0000000

Table A.83: Effect of disruptions on Audience indegree/total indegree

Sample	Estimate	Std..Error	t.value	Pr...t..
Full Data	-0.0054320	0.0007521	-7.222582	0.0000000
Most views on organization	-0.0027445	0.0011800	-2.325875	0.0200262
Middle views on organization	-0.0046203	0.0010791	-4.281744	0.0000185
Least views on organization	-0.0091136	0.0007052	-12.922743	0.0000000

Table A.84: Effect of disruptions on Audience outdegree/total outdegree

Sample	Estimate	Std..Error	t.value	Pr...t..
Full Data	-0.0044286	0.0003893	-11.3753929	0.0000000
Most views on organization	-0.0005328	0.0016660	-0.3198363	0.7490927
Middle views on organization	-0.0036095	0.0004417	-8.1711358	0.0000000
Least views on organization	-0.0094451	0.0006213	-15.2025003	0.0000000

Organization comparison plots for backlash outcomes

Views on hate content

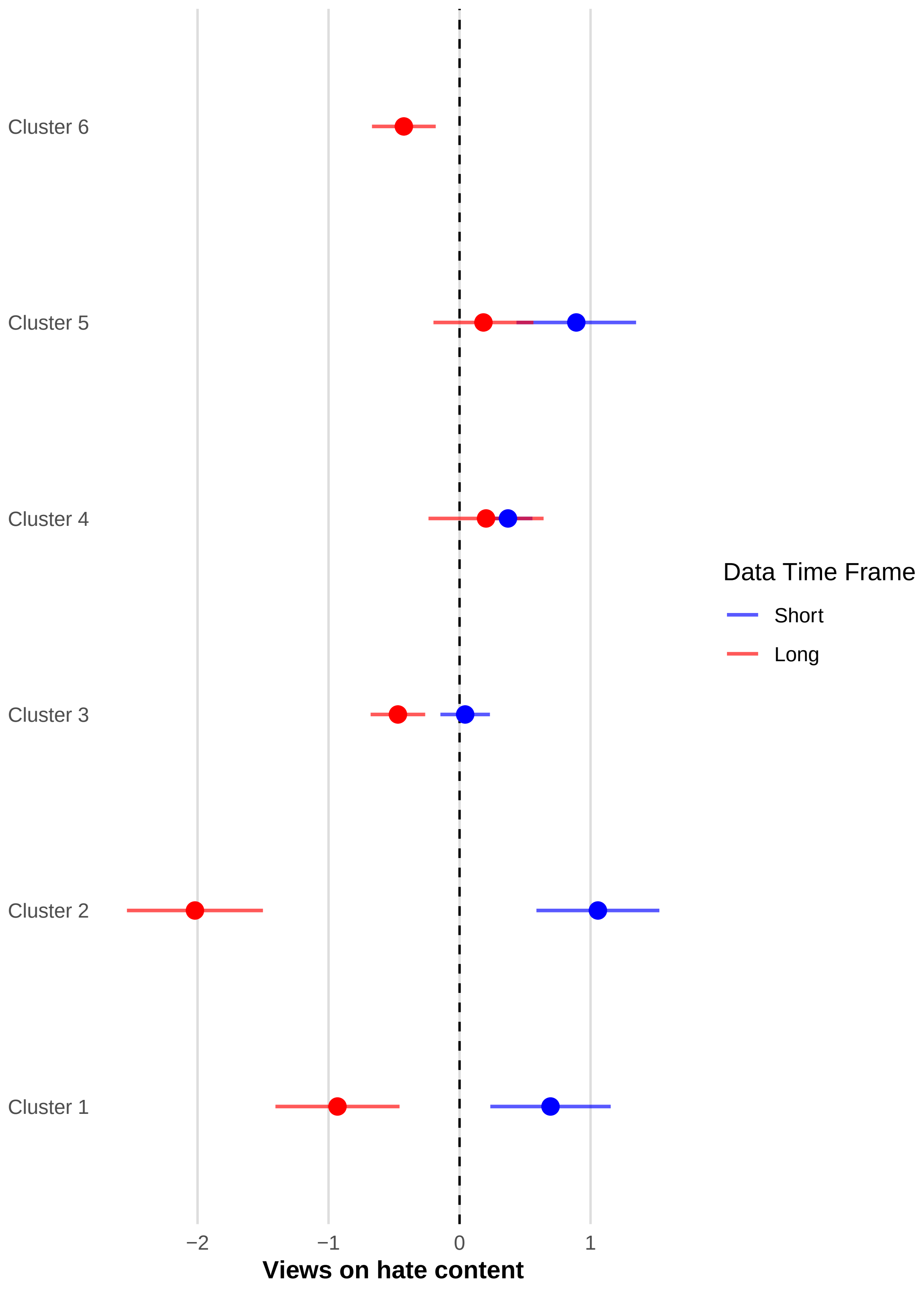

Hateful comments by audience members

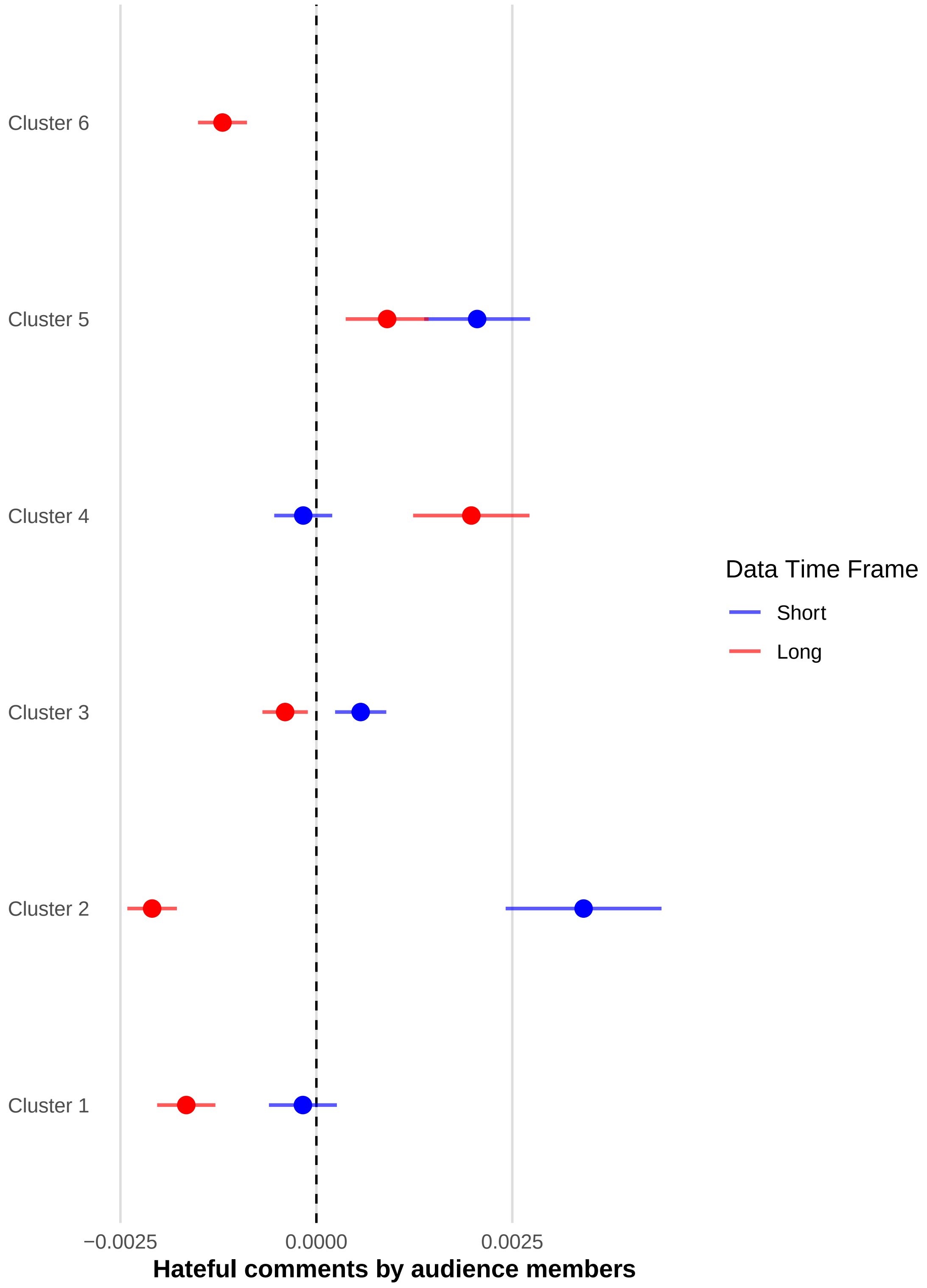

Hateful non-audience outdegree

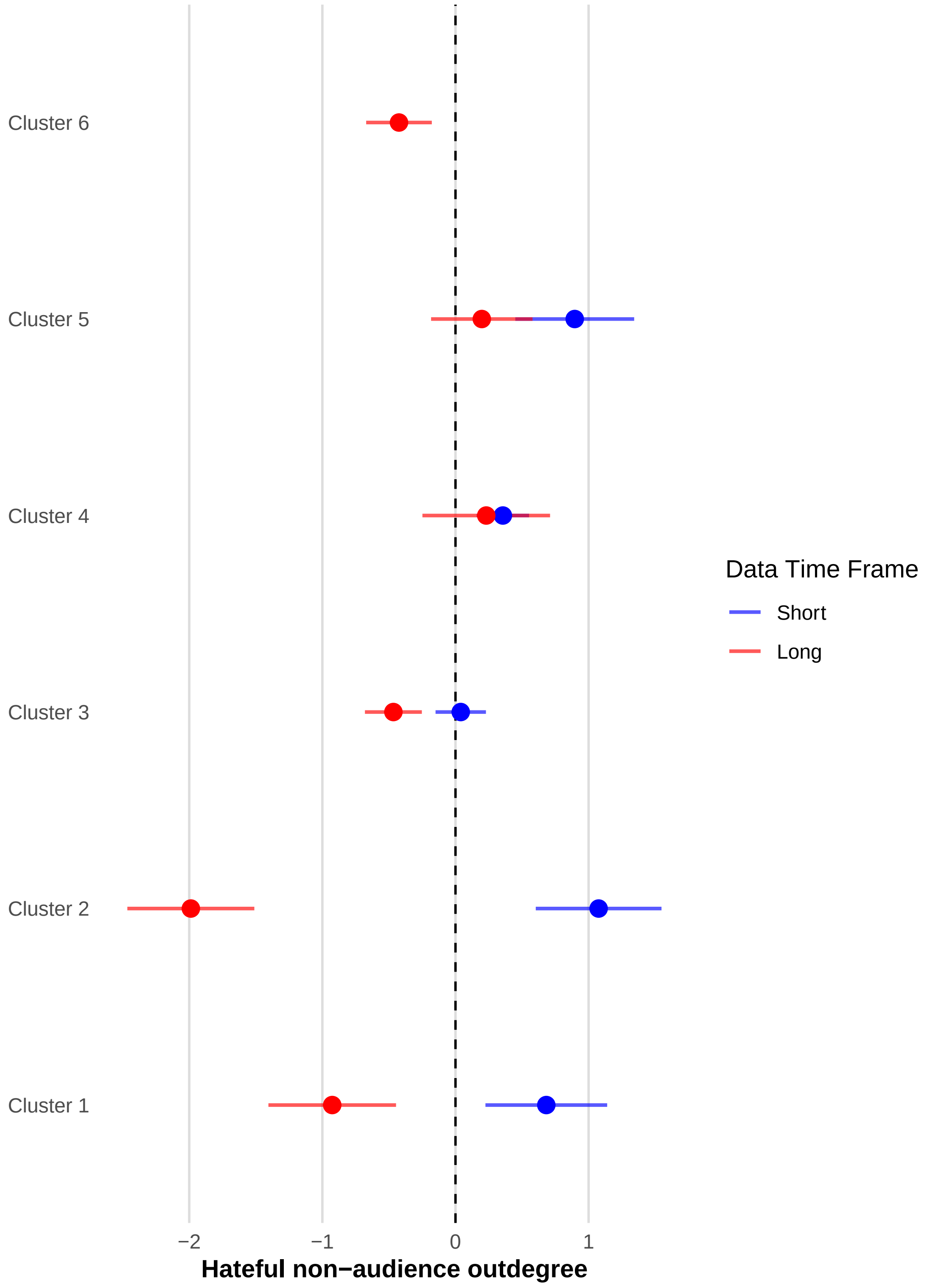

Robustness: Dynamic differences-in-differences (Callaway and Sant'Anna 2021) for short time frame with no sampled control groups

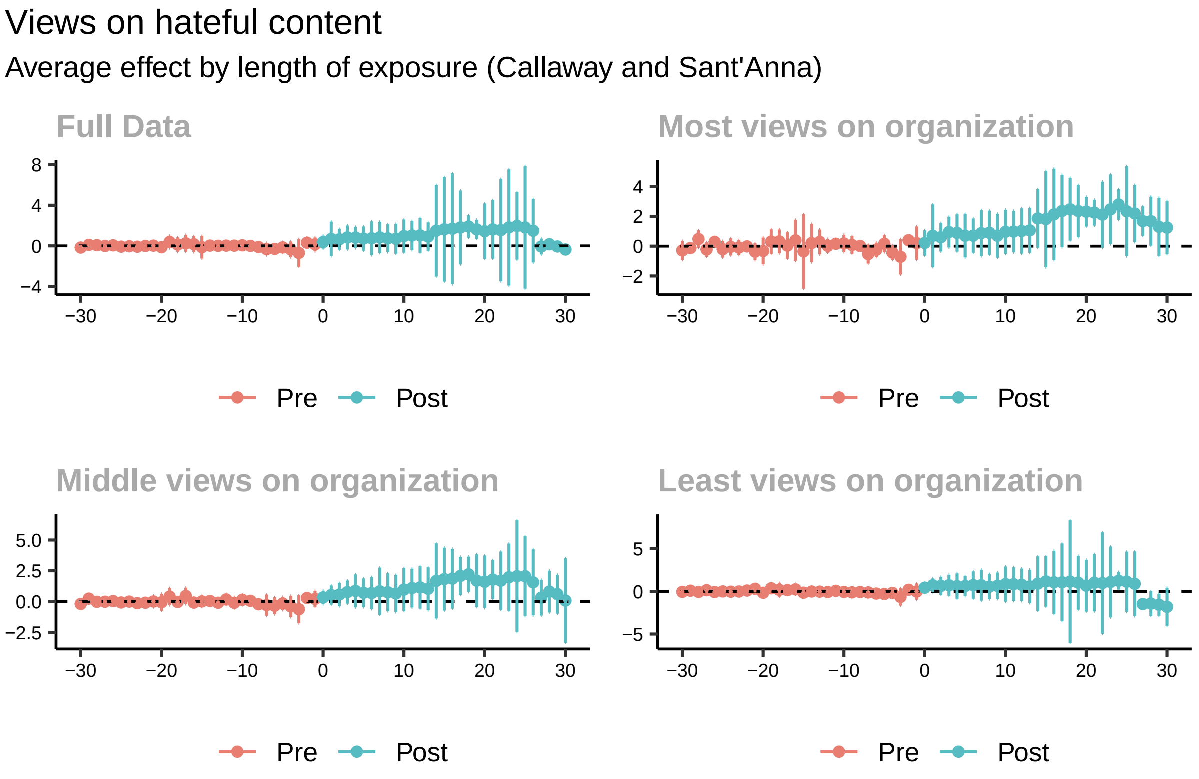

Long timeframe with sampled control groups

sample	outcome	event.time	estimate	std.error	conf.low	conf.high
Full	Views on hateful content	-30	-0.1640932	0.0901646	-0.4217024	0.0935160
Full	Views on hateful content	-29	0.0989312	0.0302151	0.0126037	0.1852587
Full	Views on hateful content	-28	0.0695868	0.0919996	-0.1932652	0.3324388
Full	Views on hateful content	-27	-0.0109624	0.0711998	-0.2143875	0.1924626
Full	Views on hateful content	-26	0.0557531	0.1104261	-0.2597453	0.3712515
Full	Views on hateful content	-25	-0.0742849	0.0912056	-0.3348685	0.1862987
Full	Views on hateful content	-24	-0.0404187	0.0880951	-0.2921152	0.2112778
Full	Views on hateful content	-23	-0.0796664	0.0427098	-0.2016925	0.0423597
Full	Views on hateful content	-22	-0.0118867	0.0365097	-0.1161985	0.0924252
Full	Views on hateful content	-21	0.0236875	0.1360391	-0.3649897	0.4123648
Full	Views on hateful content	-20	-0.1301127	0.1646941	-0.6006601	0.3404346
Full	Views on hateful content	-19	0.3831354	0.2147620	-0.2304611	0.9967320
Full	Views on hateful content	-18	0.1285521	0.2480018	-0.5800137	0.8371179
Full	Views on hateful content	-17	0.2526431	0.2875149	-0.5688156	1.0741017
Full	Views on hateful content	-16	0.1660627	0.2733162	-0.6148288	0.9469542

Full	Views on hateful content	-15	-0.1313075	0.3866289	-1.2359448	0.9733298
Full	Views on hateful content	-14	0.0272486	0.1217547	-0.3206168	0.3751139
Full	Views on hateful content	-13	0.0081647	0.0737656	-0.2025910	0.2189203
Full	Views on hateful content	-12	0.0257712	0.1128795	-0.2967368	0.3482792
Full	Views on hateful content	-11	0.0235002	0.1118199	-0.2959803	0.3429808
Full	Views on hateful content	-10	0.0574760	0.1203258	-0.2863068	0.4012589
Full	Views on hateful content	-9	-0.0023155	0.0372457	-0.1087302	0.1040991
Full	Views on hateful content	-8	-0.1105797	0.0808400	-0.3415477	0.1203884
Full	Views on hateful content	-7	-0.3197107	0.2131626	-0.9287375	0.2893161
Full	Views on hateful content	-6	-0.2973333	0.1587193	-0.7508101	0.1561435
Full	Views on hateful content	-5	-0.1505173	0.1878334	-0.6871760	0.3861414
Full	Views on hateful content	-4	-0.3429218	0.2698623	-1.1139452	0.4281015
Full	Views on hateful content	-3	-0.6919654	0.4745514	-2.0478059	0.6638752
Full	Views on hateful content	-2	0.3114085	0.1735263	-0.1843734	0.8071904
Full	Views on hateful content	-1	0.1516242	0.2342888	-0.5177622	0.8210105
Full	Views on hateful content	0	0.3706243	0.2219720	-0.2635719	1.0048205
Full	Views on hateful content	1	0.6868816	0.5929713	-1.0072963	2.3810595
Full	Views on hateful content	2	0.6450347	0.3593706	-0.3817229	1.6717924
Full	Views on hateful content	3	0.8289314	0.4113283	-0.3462744	2.0041372
Full	Views on hateful content	4	0.8197029	0.3662905	-0.2268256	1.8662314
Full	Views on hateful content	5	0.7167114	0.4155501	-0.4705565	1.9039794
Full	Views on hateful content	6	0.7473501	0.5802452	-0.9104681	2.4051684
Full	Views on hateful content	7	0.8377900	0.5343033	-0.6887676	2.3643476
Full	Views on hateful content	8	0.7430487	0.4778586	-0.6222409	2.1083384
Full	Views on hateful content	9	0.6973852	0.5112950	-0.7634356	2.1582060
Full	Views on hateful content	10	0.9661469	0.5686388	-0.6585108	2.5908046
Full	Views on hateful content	11	1.0204603	0.4963976	-0.3977970	2.4387176
Full	Views on hateful content	12	1.0345871	0.5952879	-0.6662097	2.7353840
Full	Views on hateful content	13	0.9166666	0.4856505	-0.4708851	2.3042183
Full	Views on hateful content	14	1.4916457	1.5753577	-3.0093081	5.9925995
Full	Views on hateful content	15	1.6304867	1.7976147	-3.5054774	6.7664508
Full	Views on hateful content	16	1.6863297	1.9104139	-3.7719127	7.1445722
Full	Views on hateful content	17	1.8113209	1.2706488	-1.8190491	5.4416909
Full	Views on hateful content	18	1.9057874	0.3864553	0.8016462	3.0099286
Full	Views on hateful content	19	1.6267748	0.3294232	0.6855799	2.5679697
Full	Views on hateful content	20	1.4418071	0.9538297	-1.2833793	4.1669935
Full	Views on hateful content	21	1.6222667	1.0059093	-1.2517163	4.4962498
Full	Views on hateful content	22	1.5488358	1.7589691	-3.4767140	6.5743856
Full	Views on hateful content	23	1.8149038	1.9991088	-3.8967491	7.5265566
Full	Views on hateful content	24	1.9622640	1.1565957	-1.3422449	5.2667729
Full	Views on hateful content	25	1.8164144	2.1081579	-4.2068025	7.8396312

Full	Views on hateful content	26	1.4893808	1.0892444	-1.6226987	4.6014602
Full	Views on hateful content	27	-0.0623142	0.2704737	-0.8350845	0.7104561
Full	Views on hateful content	28	0.1590992	0.1157298	-0.1715524	0.4897509
Full	Views on hateful content	29	-0.0575788	0.0586357	-0.2251067	0.1099492
Full	Views on hateful content	30	-0.3549450	0.0093802	-0.3817453	-0.3281448

sample	outcome	event.time	estimate	std.error	conf.low	conf.high
Most	Views on hateful content	-30	-0.2887458	0.2821952	-0.9213646	0.3438729
Most	Views on hateful content	-29	-0.1260209	0.0571887	-0.2542253	0.0021834
Most	Views on hateful content	-28	0.4774194	0.2624155	-0.1108576	1.0656964
Most	Views on hateful content	-27	-0.2201966	0.2160858	-0.7046129	0.2642197
Most	Views on hateful content	-26	0.2814249	0.1473578	-0.0489185	0.6117683
Most	Views on hateful content	-25	-0.2061742	0.2471883	-0.7603155	0.3479670
Most	Views on hateful content	-24	-0.0492205	0.2556640	-0.6223623	0.5239212
Most	Views on hateful content	-23	-0.0933115	0.2226494	-0.5924419	0.4058189
Most	Views on hateful content	-22	-0.0188587	0.1276814	-0.3050919	0.2673745
Most	Views on hateful content	-21	-0.3674938	0.2513771	-0.9310253	0.1960377
Most	Views on hateful content	-20	-0.3238845	0.3986326	-1.2175301	0.5697611
Most	Views on hateful content	-19	0.3048566	0.3642637	-0.5117415	1.1214548
Most	Views on hateful content	-18	0.3173440	0.3543897	-0.4771187	1.1118066
Most	Views on hateful content	-17	0.0367979	0.3930374	-0.8443044	0.9179003
Most	Views on hateful content	-16	0.3861146	0.6115286	-0.9847964	1.7570256
Most	Views on hateful content	-15	-0.3525587	1.1130385	-2.8477432	2.1426257
Most	Views on hateful content	-14	0.2066715	0.5715356	-1.0745840	1.4879271
Most	Views on hateful content	-13	0.2802958	0.3698373	-0.5487969	1.1093886
Most	Views on hateful content	-12	0.0204814	0.2050061	-0.4390966	0.4800594
Most	Views on hateful content	-11	0.1597965	0.1178397	-0.1043738	0.4239668
Most	Views on hateful content	-10	0.1792622	0.2578538	-0.3987886	0.7573130
Most	Views on hateful content	-9	0.0770243	0.2600869	-0.5060326	0.6600812
Most	Views on hateful content	-8	0.0082354	0.1104632	-0.2393986	0.2558693
Most	Views on hateful content	-7	-0.5306694	0.2807259	-1.1599944	0.0986556
Most	Views on hateful content	-6	-0.2451021	0.2127752	-0.7220968	0.2318927
Most	Views on hateful content	-5	0.1600524	0.2554641	-0.4126413	0.7327461
Most	Views on hateful content	-4	-0.4301260	0.2188764	-0.9207980	0.0605461
Most	Views on hateful content	-3	-0.7138847	0.5272533	-1.8958693	0.4680998
Most	Views on hateful content	-2	0.3959615	0.1213760	0.1238634	0.6680595
Most	Views on hateful content	-1	0.1998550	0.4968988	-0.9140813	1.3137914
Most	Views on hateful content	0	0.2135940	0.3771391	-0.6318679	1.0590558
Most	Views on hateful content	1	0.6943254	0.9345646	-1.4007603	2.7894110
Most	Views on hateful content	2	0.5969742	0.4241006	-0.3537648	1.5477132
Most	Views on hateful content	3	0.9429602	0.4583226	-0.0844971	1.9704176

Most	Views on hateful content	4	0.8897469	0.5585065	-0.3623003	2.1417940
Most	Views on hateful content	5	0.7005497	0.6474538	-0.7508975	2.1519970
Most	Views on hateful content	6	0.7025373	0.5110877	-0.4432076	1.8482822
Most	Views on hateful content	7	0.8703990	0.6869135	-0.6695079	2.4103060
Most	Views on hateful content	8	0.8986793	0.6590285	-0.5787157	2.3760744
Most	Views on hateful content	9	0.6978896	0.6534213	-0.7669355	2.1627147
Most	Views on hateful content	10	0.9647652	0.6570616	-0.5082205	2.4377510
Most	Views on hateful content	11	0.9768326	0.6204159	-0.4140015	2.3676668
Most	Views on hateful content	12	1.0147375	0.6732318	-0.4944982	2.5239732
Most	Views on hateful content	13	1.0568102	0.6640172	-0.4317684	2.5453888
Most	Views on hateful content	14	1.8543599	0.8729554	-0.1026118	3.8113315
Most	Views on hateful content	15	1.8097666	1.4371408	-1.4119830	5.0315162
Most	Views on hateful content	16	2.1290395	1.3652451	-0.9315360	5.1896149
Most	Views on hateful content	17	2.3572173	1.0799562	-0.0638040	4.7782386
Most	Views on hateful content	18	2.4708155	0.9353253	0.3740245	4.5676066
Most	Views on hateful content	19	2.3515525	0.7781943	0.6070142	4.0960908
Most	Views on hateful content	20	2.3133407	0.4439566	1.3180889	3.3085926
Most	Views on hateful content	21	2.2472957	0.3845129	1.3853035	3.1092879
Most	Views on hateful content	22	2.1046587	0.9874047	-0.1088827	4.3182002
Most	Views on hateful content	23	2.4660048	1.0425736	0.1287869	4.8032226
Most	Views on hateful content	24	2.7915376	0.4537624	1.7743033	3.8087718
Most	Views on hateful content	25	2.3398849	1.3462547	-0.6781183	5.3578881
Most	Views on hateful content	26	2.1901123	0.8523732	0.2792814	4.1009432
Most	Views on hateful content	27	1.6784887	0.4406810	0.6905799	2.6663974
Most	Views on hateful content	28	1.6758948	0.7305285	0.0382125	3.3135771
Most	Views on hateful content	29	1.2957824	0.8673807	-0.6486919	3.2402568
Most	Views on hateful content	30	1.2442937	0.7900104	-0.5267337	3.0153210

sample	outcome	event.time	estimate	std.error	conf.low	conf.high
Middle	Views on hateful content	-30	-0.1933987	0.1086583	-0.4712573	0.0844599
Middle	Views on hateful content	-29	0.2391305	0.0426878	0.1299702	0.3482908
Middle	Views on hateful content	-28	-0.0218228	0.1381103	-0.3749955	0.3313500
Middle	Views on hateful content	-27	-0.0120644	0.1105031	-0.2946406	0.2705117
Middle	Views on hateful content	-26	0.0391182	0.0653614	-0.1280226	0.2062590
Middle	Views on hateful content	-25	-0.0764202	0.1251521	-0.3964565	0.2436161
Middle	Views on hateful content	-24	-0.0024988	0.0975379	-0.2519205	0.2469229
Middle	Views on hateful content	-23	-0.1290395	0.0663058	-0.2985954	0.0405163
Middle	Views on hateful content	-22	-0.0919419	0.0325115	-0.1750795	-0.0088043
Middle	Views on hateful content	-21	0.0046431	0.1821611	-0.4611753	0.4704616
Middle	Views on hateful content	-20	-0.0586918	0.2690524	-0.7467068	0.6293232
Middle	Views on hateful content	-19	0.4003271	0.2694179	-0.2886224	1.0892766

Middle	Views on hateful content	-18	-0.0327679	0.1532567	-0.4246725	0.3591367
Middle	Views on hateful content	-17	0.4561173	0.2619312	-0.2136874	1.1259219
Middle	Views on hateful content	-16	-0.0846713	0.1651462	-0.5069794	0.3376369
Middle	Views on hateful content	-15	0.0104997	0.1746367	-0.4360773	0.4570768
Middle	Views on hateful content	-14	0.0467867	0.1000826	-0.2091423	0.3027157
Middle	Views on hateful content	-13	-0.0959929	0.0727588	-0.2820502	0.0900644
Middle	Views on hateful content	-12	0.1721922	0.1755274	-0.2766628	0.6210471
Middle	Views on hateful content	-11	-0.1022753	0.1833370	-0.5711008	0.3665501
Middle	Views on hateful content	-10	0.1446152	0.1696877	-0.2893065	0.5785370
Middle	Views on hateful content	-9	0.0612869	0.0842428	-0.1541369	0.2767106
Middle	Views on hateful content	-8	-0.2165999	0.0438452	-0.3287200	-0.1044798
Middle	Views on hateful content	-7	-0.2887625	0.3337837	-1.1423070	0.5647820
Middle	Views on hateful content	-6	-0.3377171	0.2688708	-1.0252677	0.3498334
Middle	Views on hateful content	-5	-0.1583769	0.2078927	-0.6899957	0.3732418
Middle	Views on hateful content	-4	-0.3973291	0.3368288	-1.2586604	0.4640022
Middle	Views on hateful content	-3	-0.6209583	0.4432983	-1.7545509	0.5126344
Middle	Views on hateful content	-2	0.2885289	0.1180794	-0.0134212	0.5904790
Middle	Views on hateful content	-1	0.2171409	0.2532719	-0.4305205	0.8648023
Middle	Views on hateful content	0	0.3362219	0.2134703	-0.2096598	0.8821035
Middle	Views on hateful content	1	0.5313967	0.3037744	-0.2454085	1.3082019
Middle	Views on hateful content	2	0.5653021	0.3673883	-0.3741753	1.5047795
Middle	Views on hateful content	3	0.7670909	0.3642675	-0.1644061	1.6985878
Middle	Views on hateful content	4	0.8674345	0.5214242	-0.4659402	2.2008091
Middle	Views on hateful content	5	0.7119434	0.4554239	-0.4526566	1.8765434
Middle	Views on hateful content	6	0.6922750	0.5062097	-0.6021933	1.9867434
Middle	Views on hateful content	7	0.8365299	0.7492993	-1.0795618	2.7526216
Middle	Views on hateful content	8	0.7551769	0.5986073	-0.7755685	2.2859224
Middle	Views on hateful content	9	0.6552808	0.5897674	-0.8528597	2.1634212
Middle	Views on hateful content	10	0.9698659	0.6776473	-0.7629990	2.7027309
Middle	Views on hateful content	11	1.0973839	0.6140447	-0.4728377	2.6676055
Middle	Views on hateful content	12	1.1497436	0.6712258	-0.5667004	2.8661876
Middle	Views on hateful content	13	1.0394235	0.6771592	-0.6921931	2.7710402
Middle	Views on hateful content	14	1.6784957	1.1908106	-1.3666192	4.7236106
Middle	Views on hateful content	15	1.8255523	0.9960442	-0.7215102	4.3726149
Middle	Views on hateful content	16	1.8600650	0.9478916	-0.5638627	4.2839928
Middle	Views on hateful content	17	2.0830562	0.6058574	0.5337708	3.6323416
Middle	Views on hateful content	18	2.2135650	0.5600916	0.7813109	3.6458190
Middle	Views on hateful content	19	1.6992455	0.8381981	-0.4441764	3.8426675
Middle	Views on hateful content	20	1.6073386	0.8326220	-0.5218243	3.7365015
Middle	Views on hateful content	21	1.7879557	0.6151554	0.2148939	3.3610176
Middle	Views on hateful content	22	1.6946039	0.9249197	-0.6705805	4.0597882

Middle	Views on hateful content	23	1.9759208	1.0682899	-0.7558868	4.7077284
Middle	Views on hateful content	24	2.0608637	1.7717486	-2.4698132	6.5915406
Middle	Views on hateful content	25	2.0597410	1.2643091	-1.1733227	5.2928046
Middle	Views on hateful content	26	1.5596148	1.0464958	-1.1164613	4.2356909
Middle	Views on hateful content	27	0.3158103	0.5648476	-1.1286057	1.7602263
Middle	Views on hateful content	28	0.8108127	0.6624342	-0.8831497	2.5047750
Middle	Views on hateful content	29	0.6006871	0.6148412	-0.9715712	2.1729455
Middle	Views on hateful content	30	0.0815616	1.3424856	-3.3514133	3.5145364

sample	outcome	event.time	estimate	std.error	conf.low	conf.high
Least	Views on hateful content	-30	-0.0603217	0.0679958	-0.2113271	0.0906837
Least	Views on hateful content	-29	0.0751493	0.1187863	-0.1886517	0.3389503
Least	Views on hateful content	-28	-0.0695978	0.1558930	-0.4158055	0.2766099
Least	Views on hateful content	-27	0.1304497	0.1937206	-0.2997657	0.5606650
Least	Views on hateful content	-26	-0.0816879	0.2112815	-0.5509027	0.3875269
Least	Views on hateful content	-25	-0.0085557	0.1109004	-0.2548438	0.2377324
Least	Views on hateful content	-24	-0.0397465	0.1196669	-0.3055033	0.2260102
Least	Views on hateful content	-23	-0.0200935	0.0638808	-0.1619602	0.1217733
Least	Views on hateful content	-22	0.0925241	0.0536926	-0.0267167	0.2117649
Least	Views on hateful content	-21	0.3030380	0.1945842	-0.1290953	0.7351713
Least	Views on hateful content	-20	-0.1672153	0.0883837	-0.3634982	0.0290676
Least	Views on hateful content	-19	0.3545159	0.0759709	0.1857993	0.5232324
Least	Views on hateful content	-18	0.1724139	0.3634424	-0.6347202	0.9795481
Least	Views on hateful content	-17	0.1375400	0.2147474	-0.3393717	0.6144517
Least	Views on hateful content	-16	0.2349888	0.2835006	-0.3946103	0.8645879
Least	Views on hateful content	-15	-0.1574628	0.2044585	-0.6115250	0.2965995
Least	Views on hateful content	-14	-0.0177719	0.1042118	-0.2492060	0.2136621
Least	Views on hateful content	-13	-0.0338537	0.1302394	-0.3230897	0.2553824
Least	Views on hateful content	-12	-0.0795778	0.1811876	-0.4819600	0.3228043
Least	Views on hateful content	-11	0.0616509	0.1233325	-0.2122463	0.3355481
Least	Views on hateful content	-10	-0.0733056	0.1409799	-0.3863942	0.2397829
Least	Views on hateful content	-9	-0.1163850	0.1137838	-0.3690766	0.1363066
Least	Views on hateful content	-8	-0.0795468	0.1570117	-0.4282390	0.2691454
Least	Views on hateful content	-7	-0.1269226	0.0887930	-0.3241146	0.0702693
Least	Views on hateful content	-6	-0.2599361	0.1803895	-0.6605459	0.1406736
Least	Views on hateful content	-5	-0.3105912	0.1800395	-0.7104236	0.0892412
Least	Views on hateful content	-4	-0.1872057	0.2387077	-0.7173286	0.3429172
Least	Views on hateful content	-3	-0.6580058	0.4517351	-1.6612209	0.3452092
Least	Views on hateful content	-2	0.1821385	0.1287620	-0.1038165	0.4680935
Least	Views on hateful content	-1	-0.0189873	0.4244494	-0.9616062	0.9236315
Least	Views on hateful content	0	0.4253834	0.2632489	-0.1592407	1.0100075

Least	Views on hateful content	1	0.7103481	0.3578469	-0.0843597	1.5050558
Least	Views on hateful content	2	0.6327921	0.4720808	-0.4156068	1.6811909
Least	Views on hateful content	3	0.7128442	0.5501113	-0.5088452	1.9345335
Least	Views on hateful content	4	0.6096476	0.6718269	-0.8823480	2.1016432
Least	Views on hateful content	5	0.6180340	0.5220922	-0.5414304	1.7774984
Least	Views on hateful content	6	0.7312036	0.7196078	-0.8669042	2.3293113
Least	Views on hateful content	7	0.7180407	0.8103495	-1.0815866	2.5176680
Least	Views on hateful content	8	0.5470516	0.6627672	-0.9248244	2.0189275
Least	Views on hateful content	9	0.6396120	0.6976490	-0.9097296	2.1889536
Least	Views on hateful content	10	0.8481398	0.9233593	-1.2024599	2.8987395
Least	Views on hateful content	11	0.8404267	0.8834491	-1.1215404	2.8023938
Least	Views on hateful content	12	0.7694750	0.8511357	-1.1207303	2.6596803
Least	Views on hateful content	13	0.5716186	0.8791018	-1.3806939	2.5239311
Least	Views on hateful content	14	0.9008246	1.4335640	-2.2828397	4.0844889
Least	Views on hateful content	15	1.1597806	1.3257025	-1.7843444	4.1039056
Least	Views on hateful content	16	1.0605395	1.6624585	-2.6314547	4.7525336
Least	Views on hateful content	17	1.0621915	2.0445961	-3.4784553	5.6028382
Least	Views on hateful content	18	1.1390592	3.2278006	-6.0292523	8.3073708
Least	Views on hateful content	19	1.0098024	1.4145373	-2.1316073	4.1512120
Least	Views on hateful content	20	0.6660419	1.3647332	-2.3647627	3.6968465
Least	Views on hateful content	21	1.0045093	1.5066557	-2.3414774	4.3504959
Least	Views on hateful content	22	0.9689239	2.6691318	-4.9586939	6.8965418
Least	Views on hateful content	23	1.0969598	1.8664182	-3.0479887	5.2419084
Least	Views on hateful content	24	1.2360449	0.4584531	0.2179104	2.2541793
Least	Views on hateful content	25	1.1314707	1.5816016	-2.3809561	4.6438975
Least	Views on hateful content	26	0.8853821	1.7026208	-2.8958046	4.6665688
Least	Views on hateful content	27	-1.4850305	0.0901846	-1.6853128	-1.2847483
Least	Views on hateful content	28	-1.4292678	0.6514733	-2.8760622	0.0175266
Least	Views on hateful content	29	-1.5645742	0.5782609	-2.8487782	-0.2803702
Least	Views on hateful content	30	-1.8161823	0.9998895	-4.0367408	0.4043761

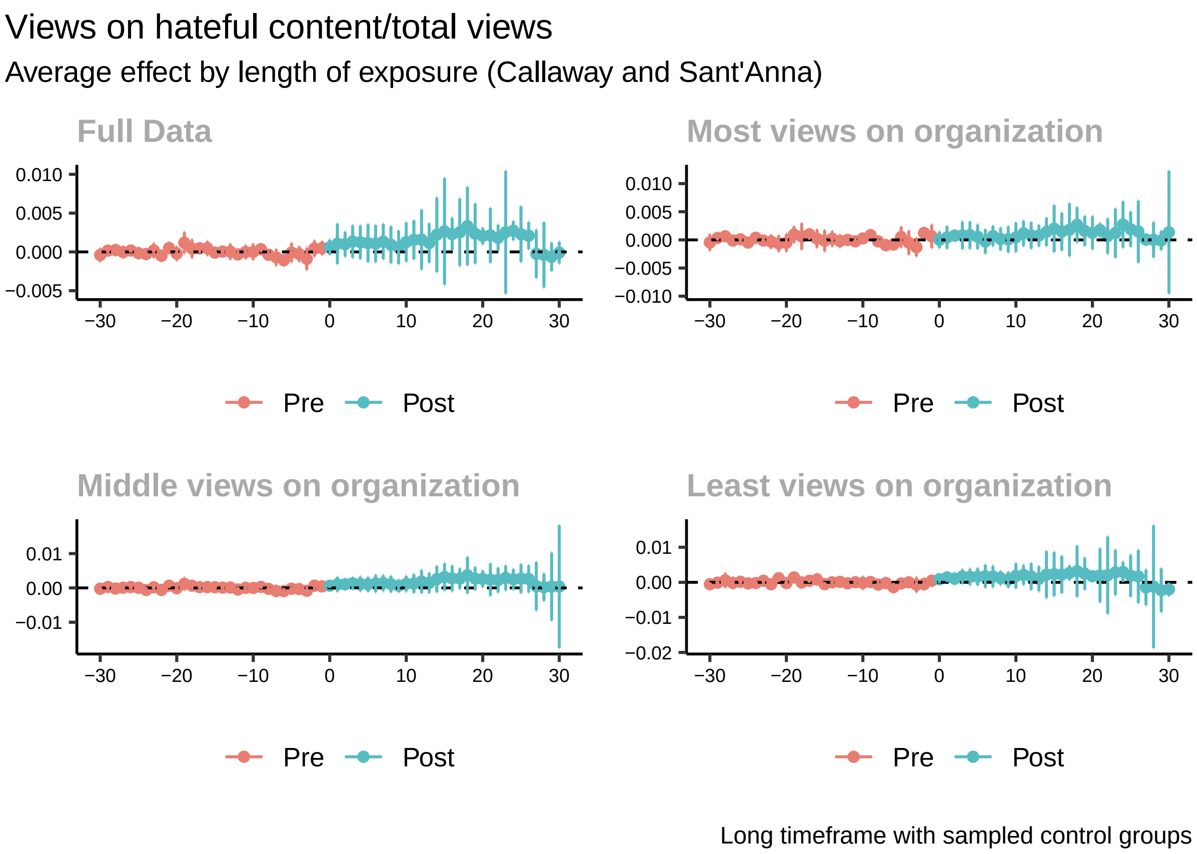

sample	outcome	event.time	estimate	std.error	conf.low	conf.high
Full	Views on hateful content/total views	-30	-0.0003933	0.0003464	-0.0012657	0.0004791
Full	Views on hateful content/total views	-29	0.0001647	0.0001569	-0.0002305	0.0005599
Full	Views on hateful content/total views	-28	0.0002329	0.0002784	-0.0004682	0.0009339
Full	Views on hateful content/total views	-27	-0.0000263	0.0003113	-0.0008102	0.0007575
Full	Views on hateful content/total views	-26	0.0001784	0.0001188	-0.0001208	0.0004777
Full	Views on hateful content/total views	-25	-0.0001617	0.0002627	-0.0008233	0.0004999
Full	Views on hateful content/total views	-24	-0.0002759	0.0002486	-0.0009020	0.0003502
Full	Views on hateful content/total views	-23	0.0001831	0.0003783	-0.0007695	0.0011358
Full	Views on hateful content/total views	-22	-0.0005013	0.0002804	-0.0012074	0.0002048
Full	Views on hateful content/total views	-21	0.0004951	0.0002992	-0.0002584	0.0012487
Full	Views on hateful content/total views	-20	-0.0002088	0.0003509	-0.0010925	0.0006748
Full	Views on hateful content/total views	-19	0.0011803	0.0005419	-0.0001842	0.0025448
Full	Views on hateful content/total views	-18	0.0004170	0.0004722	-0.0007720	0.0016060
Full	Views on hateful content/total views	-17	0.0004649	0.0002561	-0.0001801	0.0011098
Full	Views on hateful content/total views	-16	0.0004676	0.0003661	-0.0004542	0.0013894
Full	Views on hateful content/total views	-15	-0.0000983	0.0001718	-0.0005308	0.0003343
Full	Views on hateful content/total views	-14	0.0000454	0.0001788	-0.0004050	0.0004957
Full	Views on hateful content/total views	-13	0.0000313	0.0003988	-0.0009730	0.0010356
Full	Views on hateful content/total views	-12	-0.0003143	0.0002404	-0.0009196	0.0002910

Full	Views on hateful content/total views	-11	-0.0000176	0.0003498	-0.0008985	0.0008633
Full	Views on hateful content/total views	-10	0.0000033	0.0004102	-0.0010295	0.0010361
Full	Views on hateful content/total views	-9	0.0003474	0.0001633	-0.0000637	0.0007586
Full	Views on hateful content/total views	-8	-0.0003666	0.0002332	-0.0009537	0.0002206
Full	Views on hateful content/total views	-7	-0.0007026	0.0004233	-0.0017686	0.0003634
Full	Views on hateful content/total views	-6	-0.0010668	0.0003005	-0.0018236	-0.0003100
Full	Views on hateful content/total views	-5	-0.0000919	0.0004894	-0.0013243	0.0011405
Full	Views on hateful content/total views	-4	-0.0002663	0.0003864	-0.0012393	0.0007068
Full	Views on hateful content/total views	-3	-0.0008900	0.0005667	-0.0023170	0.0005370
Full	Views on hateful content/total views	-2	0.0004315	0.0004106	-0.0006026	0.0014656
Full	Views on hateful content/total views	-1	0.0004840	0.0003523	-0.0004033	0.0013712
Full	Views on hateful content/total views	0	0.0005989	0.0003865	-0.0003743	0.0015721
Full	Views on hateful content/total views	1	0.0010490	0.0010300	-0.0015447	0.0036426
Full	Views on hateful content/total views	2	0.0009938	0.0006359	-0.0006075	0.0025952
Full	Views on hateful content/total views	3	0.0013446	0.0008233	-0.0007286	0.0034179
Full	Views on hateful content/total views	4	0.0012393	0.0008563	-0.0009169	0.0033955
Full	Views on hateful content/total views	5	0.0011341	0.0009687	-0.0013052	0.0035734
Full	Views on hateful content/total views	6	0.0010672	0.0009446	-0.0013115	0.0034459
Full	Views on hateful content/total views	7	0.0013269	0.0008991	-0.0009370	0.0035909
Full	Views on hateful content/total views	8	0.0009218	0.0009395	-0.0014439	0.0032875
Full	Views on hateful content/total views	9	0.0005764	0.0008555	-0.0015780	0.0027307
Full	Views on hateful content/total views	10	0.0012713	0.0009978	-0.0012414	0.0037840
Full	Views on hateful content/total views	11	0.0015546	0.0009894	-0.0009368	0.0040460
Full	Views on hateful content/total views	12	0.0015750	0.0015269	-0.0022699	0.0054199
Full	Views on hateful content/total views	13	0.0011527	0.0010005	-0.0013667	0.0036720
Full	Views on hateful content/total views	14	0.0022125	0.0019015	-0.0025759	0.0070008
Full	Views on hateful content/total views	15	0.0026470	0.0027270	-0.0042200	0.0095141
Full	Views on hateful content/total views	16	0.0022831	0.0008300	0.0001931	0.0043731
Full	Views on hateful content/total views	17	0.0025300	0.0017224	-0.0018074	0.0068673
Full	Views on hateful content/total views	18	0.0033167	0.0019980	-0.0017144	0.0083479
Full	Views on hateful content/total views	19	0.0023537	0.0015336	-0.0015081	0.0062154
Full	Views on hateful content/total views	20	0.0020447	0.0004213	0.0009837	0.0031057
Full	Views on hateful content/total views	21	0.0021339	0.0013969	-0.0013836	0.0056514
Full	Views on hateful content/total views	22	0.0018181	0.0006676	0.0001371	0.0034991
Full	Views on hateful content/total views	23	0.0025357	0.0031357	-0.0053604	0.0104318
Full	Views on hateful content/total views	24	0.0027387	0.0004873	0.0015116	0.0039658
Full	Views on hateful content/total views	25	0.0022802	0.0014228	-0.0013027	0.0058632
Full	Views on hateful content/total views	26	0.0020919	0.0006906	0.0003527	0.0038310
Full	Views on hateful content/total views	27	-0.0002533	0.0012274	-0.0033441	0.0028376
Full	Views on hateful content/total views	28	-0.0003787	0.0016691	-0.0045817	0.0038244
Full	Views on hateful content/total views	29	-0.0006356	0.0007170	-0.0024411	0.0011698

Full	Views on hateful content/total views	30	-0.0000896	0.0005524	-0.0014806	0.0013014

sample	outcome	event.time	estimate	std.error	conf.low	conf.high
Most	Views on hateful content/total views	-30	-0.0004523	0.0006731	-0.0019157	0.0010111
Most	Views on hateful content/total views	-29	0.0003434	0.0002229	-0.0001411	0.0008279
Most	Views on hateful content/total views	-28	0.0006454	0.0003265	-0.0000644	0.0013552
Most	Views on hateful content/total views	-27	-0.0001067	0.0002239	-0.0005935	0.0003801
Most	Views on hateful content/total views	-26	0.0000993	0.0002233	-0.0003862	0.0005848
Most	Views on hateful content/total views	-25	-0.0004674	0.0001932	-0.0008875	-0.0000473
Most	Views on hateful content/total views	-24	0.0003761	0.0003085	-0.0002945	0.0010467
Most	Views on hateful content/total views	-23	-0.0001137	0.0003884	-0.0009582	0.0007308
Most	Views on hateful content/total views	-22	-0.0003100	0.0005546	-0.0015157	0.0008956
Most	Views on hateful content/total views	-21	-0.0006320	0.0006637	-0.0020748	0.0008109
Most	Views on hateful content/total views	-20	-0.0005131	0.0007215	-0.0020817	0.0010554
Most	Views on hateful content/total views	-19	0.0009351	0.0006746	-0.0005315	0.0024017
Most	Views on hateful content/total views	-18	0.0006112	0.0010699	-0.0017148	0.0029371
Most	Views on hateful content/total views	-17	0.0009906	0.0004430	0.0000275	0.0019537
Most	Views on hateful content/total views	-16	0.0002208	0.0007395	-0.0013868	0.0018285
Most	Views on hateful content/total views	-15	-0.0001396	0.0008676	-0.0020259	0.0017467
Most	Views on hateful content/total views	-14	0.0001934	0.0006285	-0.0011730	0.0015597
Most	Views on hateful content/total views	-13	-0.0001966	0.0005207	-0.0013287	0.0009355
Most	Views on hateful content/total views	-12	-0.0000121	0.0003886	-0.0008569	0.0008327
Most	Views on hateful content/total views	-11	-0.0002459	0.0002947	-0.0008865	0.0003947
Most	Views on hateful content/total views	-10	0.0002656	0.0002177	-0.0002078	0.0007390
Most	Views on hateful content/total views	-9	0.0008464	0.0002764	0.0002454	0.0014473
Most	Views on hateful content/total views	-8	-0.0002636	0.0002386	-0.0007823	0.0002550
Most	Views on hateful content/total views	-7	-0.0009259	0.0004113	-0.0018201	-0.0000318
Most	Views on hateful content/total views	-6	-0.0008202	0.0002492	-0.0013620	-0.0002784
Most	Views on hateful content/total views	-5	0.0004966	0.0008393	-0.0013282	0.0023213
Most	Views on hateful content/total views	-4	-0.0004976	0.0009720	-0.0026108	0.0016155
Most	Views on hateful content/total views	-3	-0.0012946	0.0007454	-0.0029152	0.0003260
Most	Views on hateful content/total views	-2	0.0012267	0.0002057	0.0007795	0.0016739
Most	Views on hateful content/total views	-1	0.0006157	0.0009471	-0.0014434	0.0026748
Most	Views on hateful content/total views	0	0.0000106	0.0006740	-0.0014546	0.0014759
Most	Views on hateful content/total views	1	0.0004332	0.0009177	-0.0015620	0.0024284
Most	Views on hateful content/total views	2	0.0007616	0.0004170	-0.0001449	0.0016681
Most	Views on hateful content/total views	3	0.0007958	0.0011228	-0.0016451	0.0032367
Most	Views on hateful content/total views	4	0.0008287	0.0011041	-0.0015716	0.0032290
Most	Views on hateful content/total views	5	0.0005529	0.0009996	-0.0016203	0.0027262
Most	Views on hateful content/total views	6	-0.0002803	0.0009921	-0.0024372	0.0018766
Most	Views on hateful content/total views	7	0.0006818	0.0008279	-0.0011180	0.0024816

Most	Views on hateful content/total views	8	0.0001479	0.0009136	-0.0018382	0.0021340
Most	Views on hateful content/total views	9	0.0000863	0.0010608	-0.0022199	0.0023924
Most	Views on hateful content/total views	10	0.0004555	0.0011932	-0.0021385	0.0030494
Most	Views on hateful content/total views	11	0.0011625	0.0010240	-0.0010637	0.0033888
Most	Views on hateful content/total views	12	0.0007890	0.0010759	-0.0015500	0.0031280
Most	Views on hateful content/total views	13	0.0007509	0.0008710	-0.0011427	0.0026445
Most	Views on hateful content/total views	14	0.0014219	0.0011453	-0.0010679	0.0039118
Most	Views on hateful content/total views	15	0.0019921	0.0019170	-0.0021755	0.0061597
Most	Views on hateful content/total views	16	0.0014597	0.0015443	-0.0018977	0.0048172
Most	Views on hateful content/total views	17	0.0017975	0.0021574	-0.0028928	0.0064878
Most	Views on hateful content/total views	18	0.0026834	0.0014421	-0.0004519	0.0058186
Most	Views on hateful content/total views	19	0.0015947	0.0012135	-0.0010434	0.0042328
Most	Views on hateful content/total views	20	0.0012662	0.0013464	-0.0016609	0.0041933
Most	Views on hateful content/total views	21	0.0017568	0.0005571	0.0005458	0.0029679
Most	Views on hateful content/total views	22	0.0006644	0.0014393	-0.0024647	0.0037935
Most	Views on hateful content/total views	23	0.0012111	0.0019980	-0.0031326	0.0055548
Most	Views on hateful content/total views	24	0.0027376	0.0018889	-0.0013689	0.0068441
Most	Views on hateful content/total views	25	0.0019015	0.0014323	-0.0012123	0.0050154
Most	Views on hateful content/total views	26	0.0014796	0.0025300	-0.0040206	0.0069799
Most	Views on hateful content/total views	27	0.0000535	0.0004300	-0.0008814	0.0009883
Most	Views on hateful content/total views	28	0.0000102	0.0014307	-0.0031002	0.0031205
Most	Views on hateful content/total views	29	-0.0000931	0.0007745	-0.0017769	0.0015907
Most	Views on hateful content/total views	30	0.0013607	0.0050053	-0.0095210	0.0122425

sample	outcome	event.time	estimate	std.error	conf.low	conf.high
Middle	Views on hateful content/total views	-30	-0.0002295	0.0003968	-0.0010832	0.0006242
Middle	Views on hateful content/total views	-29	0.0002679	0.0004411	-0.0006810	0.0012169
Middle	Views on hateful content/total views	-28	-0.0001787	0.0006427	-0.0015614	0.0012039
Middle	Views on hateful content/total views	-27	0.0000724	0.0004866	-0.0009743	0.0011192
Middle	Views on hateful content/total views	-26	0.0002420	0.0003781	-0.0005714	0.0010555
Middle	Views on hateful content/total views	-25	0.0000474	0.0002326	-0.0004529	0.0005478
Middle	Views on hateful content/total views	-24	-0.0006038	0.0004012	-0.0014668	0.0002593
Middle	Views on hateful content/total views	-23	0.0001857	0.0003151	-0.0004921	0.0008634
Middle	Views on hateful content/total views	-22	-0.0005875	0.0002318	-0.0010862	-0.0000889
Middle	Views on hateful content/total views	-21	0.0006593	0.0003391	-0.0000701	0.0013887
Middle	Views on hateful content/total views	-20	-0.0000926	0.0006331	-0.0014546	0.0012693
Middle	Views on hateful content/total views	-19	0.0011649	0.0008363	-0.0006342	0.0029640
Middle	Views on hateful content/total views	-18	0.0006457	0.0004834	-0.0003941	0.0016855
Middle	Views on hateful content/total views	-17	0.0002239	0.0003985	-0.0006333	0.0010811
Middle	Views on hateful content/total views	-16	0.0002392	0.0002825	-0.0003685	0.0008469
Middle	Views on hateful content/total views	-15	0.0002001	0.0003743	-0.0006051	0.0010053

Middle	Views on hateful content/total views	-14	0.0000930	0.0002891	-0.0005288	0.0007149
Middle	Views on hateful content/total views	-13	0.0001063	0.0004577	-0.0008783	0.0010909
Middle	Views on hateful content/total views	-12	-0.0004762	0.0003653	-0.0012620	0.0003096
Middle	Views on hateful content/total views	-11	0.0000192	0.0006765	-0.0014361	0.0014745
Middle	Views on hateful content/total views	-10	-0.0000157	0.0005279	-0.0011513	0.0011199
Middle	Views on hateful content/total views	-9	0.0003036	0.0002320	-0.0001955	0.0008026
Middle	Views on hateful content/total views	-8	-0.0002775	0.0003517	-0.0010341	0.0004790
Middle	Views on hateful content/total views	-7	-0.0008840	0.0007104	-0.0024123	0.0006443
Middle	Views on hateful content/total views	-6	-0.0009294	0.0004350	-0.0018653	0.0000064
Middle	Views on hateful content/total views	-5	-0.0002359	0.0006542	-0.0016432	0.0011713
Middle	Views on hateful content/total views	-4	-0.0003296	0.0003860	-0.0011600	0.0005009
Middle	Views on hateful content/total views	-3	-0.0008304	0.0005888	-0.0020971	0.0004362
Middle	Views on hateful content/total views	-2	0.0006470	0.0005903	-0.0006228	0.0019168
Middle	Views on hateful content/total views	-1	0.0004423	0.0004125	-0.0004451	0.0013298
Middle	Views on hateful content/total views	0	0.0006268	0.0002942	-0.0000061	0.0012597
Middle	Views on hateful content/total views	1	0.0010497	0.0009844	-0.0010679	0.0031672
Middle	Views on hateful content/total views	2	0.0010464	0.0007199	-0.0005023	0.0025951
Middle	Views on hateful content/total views	3	0.0013262	0.0008081	-0.0004122	0.0030646
Middle	Views on hateful content/total views	4	0.0011493	0.0009873	-0.0009747	0.0032733
Middle	Views on hateful content/total views	5	0.0010401	0.0009834	-0.0010754	0.0031556
Middle	Views on hateful content/total views	6	0.0012850	0.0011625	-0.0012157	0.0037857
Middle	Views on hateful content/total views	7	0.0014065	0.0010738	-0.0009034	0.0037164
Middle	Views on hateful content/total views	8	0.0010920	0.0011056	-0.0012863	0.0034703
Middle	Views on hateful content/total views	9	0.0006083	0.0008415	-0.0012020	0.0024186
Middle	Views on hateful content/total views	10	0.0012080	0.0010501	-0.0010510	0.0034671
Middle	Views on hateful content/total views	11	0.0012666	0.0012665	-0.0014579	0.0039911
Middle	Views on hateful content/total views	12	0.0018573	0.0015700	-0.0015202	0.0052347
Middle	Views on hateful content/total views	13	0.0014473	0.0013609	-0.0014803	0.0043748
Middle	Views on hateful content/total views	14	0.0025563	0.0017728	-0.0012573	0.0063699
Middle	Views on hateful content/total views	15	0.0031757	0.0018305	-0.0007622	0.0071135
Middle	Views on hateful content/total views	16	0.0027615	0.0017323	-0.0009650	0.0064881
Middle	Views on hateful content/total views	17	0.0027107	0.0013863	-0.0002715	0.0056928
Middle	Views on hateful content/total views	18	0.0036865	0.0024584	-0.0016019	0.0089750
Middle	Views on hateful content/total views	19	0.0027006	0.0015559	-0.0006464	0.0060476
Middle	Views on hateful content/total views	20	0.0026181	0.0011157	0.0002181	0.0050181
Middle	Views on hateful content/total views	21	0.0024019	0.0021792	-0.0022860	0.0070899
Middle	Views on hateful content/total views	22	0.0022795	0.0016817	-0.0013383	0.0058973
Middle	Views on hateful content/total views	23	0.0029131	0.0016584	-0.0006546	0.0064808
Middle	Views on hateful content/total views	24	0.0024814	0.0013767	-0.0004802	0.0054430
Middle	Views on hateful content/total views	25	0.0027099	0.0019494	-0.0014837	0.0069034
Middle	Views on hateful content/total views	26	0.0025997	0.0018162	-0.0013074	0.0065069

Middle	Views on hateful content/total views	27	0.0004714	0.0032682	-0.0065593	0.0075020
Middle	Views on hateful content/total views	28	0.0001973	0.0018472	-0.0037764	0.0041711
Middle	Views on hateful content/total views	29	0.0003828	0.0045845	-0.0094794	0.0102451
Middle	Views on hateful content/total views	30	0.0003704	0.0082951	-0.0174741	0.0182148

sample	outcome	event.time	estimate	std.error	conf.low	conf.high
Least	Views on hateful content/total views	-30	-0.0005420	0.0001004	-0.0007720	-0.0003121
Least	Views on hateful content/total views	-29	-0.0001052	0.0000826	-0.0002942	0.0000838
Least	Views on hateful content/total views	-28	0.0005517	0.0008967	-0.0015011	0.0026046
Least	Views on hateful content/total views	-27	-0.0001630	0.0006719	-0.0017012	0.0013752
Least	Views on hateful content/total views	-26	0.0001601	0.0003637	-0.0006724	0.0009926
Least	Views on hateful content/total views	-25	-0.0003129	0.0003826	-0.0011889	0.0005630
Least	Views on hateful content/total views	-24	-0.0002112	0.0003880	-0.0010993	0.0006770
Least	Views on hateful content/total views	-23	0.0004523	0.0004346	-0.0005425	0.0014471
Least	Views on hateful content/total views	-22	-0.0005601	0.0003736	-0.0014154	0.0002951
Least	Views on hateful content/total views	-21	0.0011656	0.0004852	0.0000548	0.0022764
Least	Views on hateful content/total views	-20	-0.0002095	0.0005489	-0.0014662	0.0010471
Least	Views on hateful content/total views	-19	0.0013882	0.0003671	0.0005478	0.0022285
Least	Views on hateful content/total views	-18	-0.0000412	0.0006159	-0.0014512	0.0013688
Least	Views on hateful content/total views	-17	0.0004379	0.0001413	0.0001146	0.0007613
Least	Views on hateful content/total views	-16	0.0008257	0.0006700	-0.0007082	0.0023596
Least	Views on hateful content/total views	-15	-0.0005184	0.0002716	-0.0011401	0.0001033
Least	Views on hateful content/total views	-14	0.0000121	0.0004194	-0.0009480	0.0009722
Least	Views on hateful content/total views	-13	0.0001303	0.0003201	-0.0006025	0.0008630
Least	Views on hateful content/total views	-12	-0.0002812	0.0005655	-0.0015758	0.0010133
Least	Views on hateful content/total views	-11	0.0000266	0.0002417	-0.0005268	0.0005800
Least	Views on hateful content/total views	-10	-0.0001776	0.0008007	-0.0020106	0.0016553
Least	Views on hateful content/total views	-9	0.0000907	0.0004299	-0.0008935	0.0010750
Least	Views on hateful content/total views	-8	-0.0006329	0.0005886	-0.0019804	0.0007145
Least	Views on hateful content/total views	-7	-0.0002167	0.0005700	-0.0015215	0.0010881
Least	Views on hateful content/total views	-6	-0.0013793	0.0005268	-0.0025852	-0.0001734
Least	Views on hateful content/total views	-5	-0.0003287	0.0004380	-0.0013315	0.0006741
Least	Views on hateful content/total views	-4	0.0000032	0.0003883	-0.0008857	0.0008920
Least	Views on hateful content/total views	-3	-0.0006953	0.0009310	-0.0028266	0.0014361
Least	Views on hateful content/total views	-2	-0.0005008	0.0007198	-0.0021485	0.0011469
Least	Views on hateful content/total views	-1	0.0004587	0.0006274	-0.0009774	0.0018949
Least	Views on hateful content/total views	0	0.0008850	0.0005064	-0.0002743	0.0020443
Least	Views on hateful content/total views	1	0.0014024	0.0005864	0.0000600	0.0027448
Least	Views on hateful content/total views	2	0.0010281	0.0007127	-0.0006035	0.0026596
Least	Views on hateful content/total views	3	0.0016547	0.0008855	-0.0003724	0.0036818
Least	Views on hateful content/total views	4	0.0015462	0.0010123	-0.0007712	0.0038635

Least	Views on hateful content/total views	5	0.0016025	0.0010685	-0.0008435	0.0040486
Least	Views on hateful content/total views	6	0.0017270	0.0014251	-0.0015354	0.0049894
Least	Views on hateful content/total views	7	0.0016726	0.0013286	-0.0013688	0.0047141
Least	Views on hateful content/total views	8	0.0012292	0.0008568	-0.0007323	0.0031906
Least	Views on hateful content/total views	9	0.0007671	0.0009806	-0.0014776	0.0030119
Least	Views on hateful content/total views	10	0.0018219	0.0015561	-0.0017404	0.0053842
Least	Views on hateful content/total views	11	0.0020916	0.0012200	-0.0007012	0.0048844
Least	Views on hateful content/total views	12	0.0016766	0.0016546	-0.0021112	0.0054645
Least	Views on hateful content/total views	13	0.0010151	0.0015597	-0.0025554	0.0045855
Least	Views on hateful content/total views	14	0.0022001	0.0029162	-0.0044759	0.0088760
Least	Views on hateful content/total views	15	0.0023459	0.0027272	-0.0038973	0.0085890
Least	Views on hateful content/total views	16	0.0022057	0.0023026	-0.0030655	0.0074769
Least	Views on hateful content/total views	17	0.0026907	0.0009119	0.0006032	0.0047781
Least	Views on hateful content/total views	18	0.0031653	0.0031695	-0.0040904	0.0104211
Least	Views on hateful content/total views	19	0.0024667	0.0019863	-0.0020805	0.0070138
Least	Views on hateful content/total views	20	0.0017392	0.0003234	0.0009989	0.0024796
Least	Views on hateful content/total views	21	0.0019858	0.0033479	-0.0056782	0.0096499
Least	Views on hateful content/total views	22	0.0020442	0.0047969	-0.0089371	0.0130254
Least	Views on hateful content/total views	23	0.0028208	0.0028073	-0.0036057	0.0092474
Least	Views on hateful content/total views	24	0.0029550	0.0012568	0.0000780	0.0058320
Least	Views on hateful content/total views	25	0.0018783	0.0026005	-0.0040749	0.0078315
Least	Views on hateful content/total views	26	0.0016684	0.0033002	-0.0058866	0.0092234
Least	Views on hateful content/total views	27	-0.0014540	0.0021984	-0.0064866	0.0035786
Least	Views on hateful content/total views	28	-0.0012321	0.0076294	-0.0186975	0.0162333
Least	Views on hateful content/total views	29	-0.0022607	0.0027080	-0.0084601	0.0039386
Least	Views on hateful content/total views	30	-0.0019910	0.0008290	-0.0038889	-0.0000932

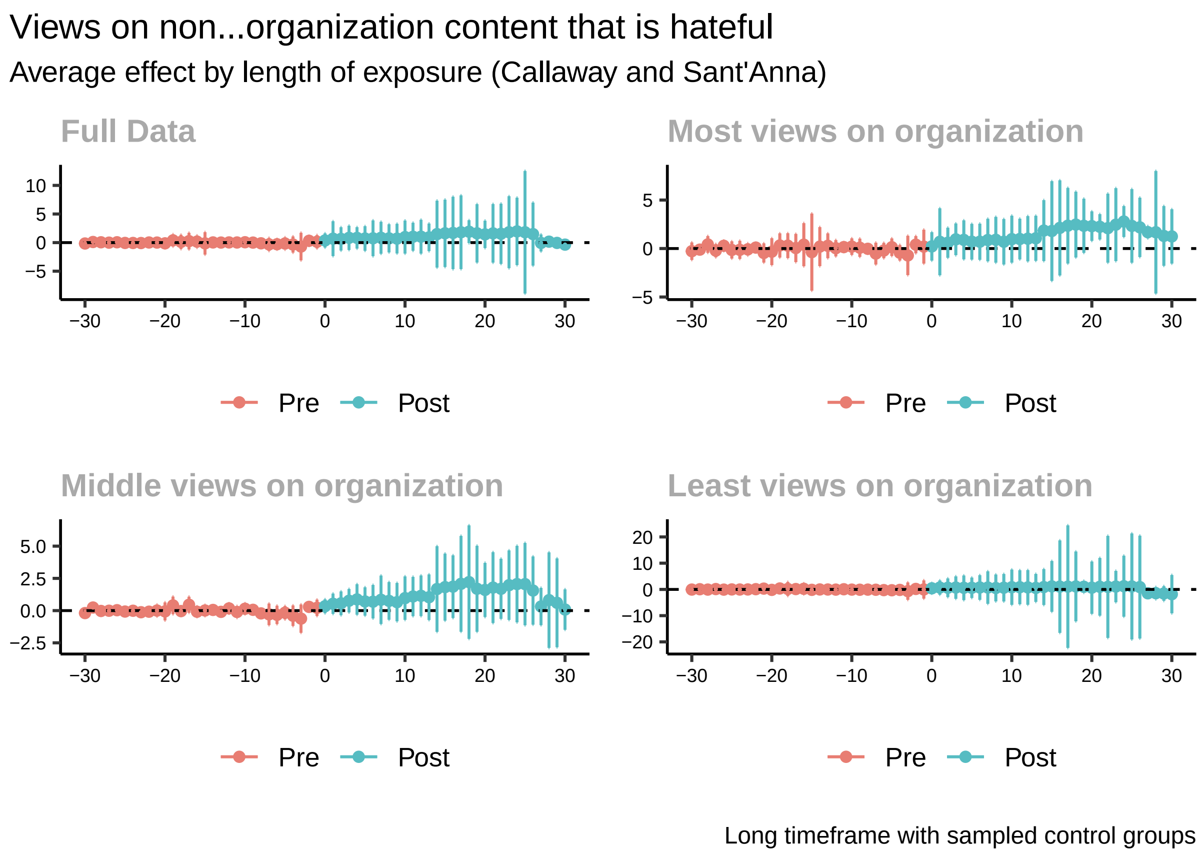

sample	outcome	event.time	estimate	std.error	conf.low	conf.high
Full	Views on nonorganization content that is hateful	-30	-0.1594788	0.0908150	-0.6227216	0.3037639
Full	Views on nonorganization content that is hateful	-29	0.1020140	0.0354047	-0.0785835	0.2826115
Full	Views on nonorganization content that is hateful	-28	0.0545432	0.0838740	-0.3732936	0.4823801
Full	Views on nonorganization content that is hateful	-27	-0.0095735	0.0699134	-0.3661981	0.3470512
Full	Views on nonorganization content that is hateful	-26	0.0548202	0.0890151	-0.3992414	0.5088818
Full	Views on nonorganization content that is hateful	-25	-0.0607390	0.0938336	-0.5393795	0.4179014
Full	Views on nonorganization content that is hateful	-24	-0.0645564	0.0923206	-0.5354794	0.4063666
Full	Views on nonorganization content that is hateful	-23	-0.0764548	0.0587631	-0.3762023	0.2232927
Full	Views on nonorganization content that is hateful	-22	0.0181276	0.0355547	-0.1632351	0.1994902
Full	Views on nonorganization content that is hateful	-21	0.0046228	0.1433108	-0.7263984	0.7356439
Full	Views on nonorganization content that is hateful	-20	-0.1311355	0.1645572	-0.9705333	0.7082623
Full	Views on nonorganization content that is hateful	-19	0.3867036	0.2093350	-0.6811034	1.4545107
Full	Views on nonorganization content that is hateful	-18	0.1223621	0.2428065	-1.1161814	1.3609056
Full	Views on nonorganization content that is hateful	-17	0.2551420	0.2834135	-1.1905362	1.7008202
Full	Views on nonorganization content that is hateful	-16	0.1676340	0.2100046	-0.9035887	1.2388568
Full	Views on nonorganization content that is hateful	-15	-0.1363677	0.3836180	-2.0931839	1.8204485
Full	Views on nonorganization content that is hateful	-14	0.0277707	0.1119525	-0.5432935	0.5988350
Full	Views on nonorganization content that is hateful	-13	0.0062243	0.0664311	-0.3326373	0.3450858
Full	Views on nonorganization content that is hateful	-12	0.0310788	0.1064169	-0.5117482	0.5739058

Full	Views on nonorganization content that is hateful	-11	0.0195447	0.1000301	-0.4907039	0.5297932
Full	Views on nonorganization content that is hateful	-10	0.0600020	0.1286035	-0.5959980	0.7160020
Full	Views on nonorganization content that is hateful	-9	0.0012809	0.0389401	-0.1973507	0.1999126
Full	Views on nonorganization content that is hateful	-8	-0.1235028	0.0597897	-0.4284870	0.1814813
Full	Views on nonorganization content that is hateful	-7	-0.3172152	0.2218541	-1.4488820	0.8144516
Full	Views on nonorganization content that is hateful	-6	-0.2864286	0.1800228	-1.2047160	0.6318587
Full	Views on nonorganization content that is hateful	-5	-0.1603508	0.2095419	-1.2292133	0.9085117
Full	Views on nonorganization content that is hateful	-4	-0.3419166	0.2761066	-1.7503226	1.0664894
Full	Views on nonorganization content that is hateful	-3	-0.6915617	0.4707583	-3.0928759	1.7097526
Full	Views on nonorganization content that is hateful	-2	0.3117832	0.1700187	-0.5554736	1.1790400
Full	Views on nonorganization content that is hateful	-1	0.1514020	0.2283574	-1.0134378	1.3162418
Full	Views on nonorganization content that is hateful	0	0.3723619	0.2418168	-0.8611332	1.6058570
Full	Views on nonorganization content that is hateful	1	0.6866104	0.5962701	-2.3549334	3.7281542
Full	Views on nonorganization content that is hateful	2	0.6445398	0.3984217	-1.3877892	2.6768688
Full	Views on nonorganization content that is hateful	3	0.8301860	0.4147308	-1.2853348	2.9457067
Full	Views on nonorganization content that is hateful	4	0.8206096	0.3664249	-1.0485050	2.6897243
Full	Views on nonorganization content that is hateful	5	0.7172029	0.4210430	-1.4305162	2.8649221
Full	Views on nonorganization content that is hateful	6	0.7467279	0.6122584	-2.3763713	3.8698271
Full	Views on nonorganization content that is hateful	7	0.8402492	0.5467130	-1.9485062	3.6290046
Full	Views on nonorganization content that is hateful	8	0.7431007	0.4802264	-1.7065099	3.1927112
Full	Views on nonorganization content that is hateful	9	0.6971821	0.5068970	-1.8884739	3.2828382
Full	Views on nonorganization content that is hateful	10	0.9661900	0.5653741	-1.9177546	3.8501346
Full	Views on nonorganization content that is hateful	11	1.0204790	0.4794476	-1.4251588	3.4661168
Full	Views on nonorganization content that is hateful	12	1.0336271	0.5794235	-1.9219829	3.9892371
Full	Views on nonorganization content that is hateful	13	0.9152640	0.4731355	-1.4981761	3.3287042
Full	Views on nonorganization content that is hateful	14	1.4888006	1.1463662	-4.3587555	7.3363567
Full	Views on nonorganization content that is hateful	15	1.6273457	1.1533861	-4.2560185	7.5107100
Full	Views on nonorganization content that is hateful	16	1.6846485	1.2406231	-4.6437073	8.0130043
Full	Views on nonorganization content that is hateful	17	1.8085529	1.2618511	-4.6280861	8.2451920
Full	Views on nonorganization content that is hateful	18	1.9043644	0.3922946	-0.0967108	3.9054395
Full	Views on nonorganization content that is hateful	19	1.6237891	0.9989338	-3.4717219	6.7193002
Full	Views on nonorganization content that is hateful	20	1.4393472	0.4690591	-0.9532998	3.8319942
Full	Views on nonorganization content that is hateful	21	1.6189697	1.0043805	-3.5043244	6.7422637
Full	Views on nonorganization content that is hateful	22	1.5458071	1.0313947	-3.7152854	6.8068997
Full	Views on nonorganization content that is hateful	23	1.8119725	1.2323657	-4.4742626	8.0982076
Full	Views on nonorganization content that is hateful	24	1.9590890	1.1546780	-3.9308654	7.8490433
Full	Views on nonorganization content that is hateful	25	1.8128550	2.0995830	-8.8970120	12.5227220
Full	Views on nonorganization content that is hateful	26	1.4860338	1.0806699	-4.0264091	6.9984767
Full	Views on nonorganization content that is hateful	27	-0.0586306	0.2893683	-1.5346836	1.4174224
Full	Views on nonorganization content that is hateful	28	0.1624302	0.0968352	-0.3315215	0.6563819
Full	Views on nonorganization content that is hateful	29	-0.0544829	0.0351294	-0.2336760	0.1247102

Full	Views on nonorganization content that is hateful	30	-0.3517316	0.0008190	-0.3559092	-0.3475539

sample	outcome	event.time	estimate	std.error	conf.low	conf.high
Most	Views on nonorganization content that is hateful	-30	-0.2750509	0.2451733	-1.1733165	0.6232147
Most	Views on nonorganization content that is hateful	-29	-0.1142233	0.0847386	-0.4246885	0.1962419
Most	Views on nonorganization content that is hateful	-28	0.4220041	0.2352619	-0.4399484	1.2839566
Most	Views on nonorganization content that is hateful	-27	-0.2175624	0.1873667	-0.9040362	0.4689115
Most	Views on nonorganization content that is hateful	-26	0.2827209	0.1461039	-0.2525745	0.8180163
Most	Views on nonorganization content that is hateful	-25	-0.1469721	0.2412750	-1.0309554	0.7370112
Most	Views on nonorganization content that is hateful	-24	-0.1364412	0.2539874	-1.0670001	0.7941178
Most	Views on nonorganization content that is hateful	-23	-0.0875409	0.1721998	-0.7184463	0.5433646
Most	Views on nonorganization content that is hateful	-22	0.0977802	0.1326956	-0.3883899	0.5839503
Most	Views on nonorganization content that is hateful	-21	-0.4593236	0.2701737	-1.4491858	0.5305386
Most	Views on nonorganization content that is hateful	-20	-0.3251532	0.3773212	-1.7075824	1.0572760
Most	Views on nonorganization content that is hateful	-19	0.3158604	0.3396882	-0.9286890	1.5604099
Most	Views on nonorganization content that is hateful	-18	0.2958409	0.3490366	-0.9829593	1.5746410
Most	Views on nonorganization content that is hateful	-17	0.0514193	0.3968987	-1.4027380	1.5055766
Most	Views on nonorganization content that is hateful	-16	0.3967402	0.6065294	-1.8254617	2.6189421
Most	Views on nonorganization content that is hateful	-15	-0.3700876	1.0854439	-4.3469361	3.6067609
Most	Views on nonorganization content that is hateful	-14	0.1964463	0.5495398	-1.8169574	2.2098499
Most	Views on nonorganization content that is hateful	-13	0.2796512	0.3487095	-0.9979503	1.5572528
Most	Views on nonorganization content that is hateful	-12	0.0352327	0.2295529	-0.8058030	0.8762684
Most	Views on nonorganization content that is hateful	-11	0.1476277	0.1345455	-0.3453202	0.6405755
Most	Views on nonorganization content that is hateful	-10	0.1949252	0.2315712	-0.6535051	1.0433554
Most	Views on nonorganization content that is hateful	-9	0.0830935	0.2566676	-0.8572851	1.0234721
Most	Views on nonorganization content that is hateful	-8	-0.0296785	0.1331865	-0.5176469	0.4582899
Most	Views on nonorganization content that is hateful	-7	-0.5181099	0.3073859	-1.6443100	0.6080903
Most	Views on nonorganization content that is hateful	-6	-0.2101286	0.2162783	-1.0025289	0.5822718
Most	Views on nonorganization content that is hateful	-5	0.1350805	0.2463246	-0.7674032	1.0375643
Most	Views on nonorganization content that is hateful	-4	-0.4329192	0.2196195	-1.2375610	0.3717227
Most	Views on nonorganization content that is hateful	-3	-0.7127692	0.5527770	-2.7380332	1.3124947
Most	Views on nonorganization content that is hateful	-2	0.3980048	0.2422110	-0.4894078	1.2854174
Most	Views on nonorganization content that is hateful	-1	0.1966459	0.4764457	-1.5489556	1.9422474
Most	Views on nonorganization content that is hateful	0	0.2179586	0.3991577	-1.2444750	1.6803922
Most	Views on nonorganization content that is hateful	1	0.6954227	0.9417324	-2.7548956	4.1457411
Most	Views on nonorganization content that is hateful	2	0.5976971	0.4228884	-0.9516811	2.1470754
Most	Views on nonorganization content that is hateful	3	0.9479506	0.4460539	-0.6863012	2.5822024
Most	Views on nonorganization content that is hateful	4	0.8939392	0.5476341	-1.1124822	2.9003607
Most	Views on nonorganization content that is hateful	5	0.7041094	0.5017858	-1.1343331	2.5425519
Most	Views on nonorganization content that is hateful	6	0.7028505	0.5090372	-1.1621594	2.5678605
Most	Views on nonorganization content that is hateful	7	0.8760699	0.5974458	-1.3128517	3.0649914

Most	Views on nonorganization content that is hateful	8	0.9006702	0.6442708	-1.4598085	3.2611490
Most	Views on nonorganization content that is hateful	9	0.6998232	0.6390235	-1.6414307	3.0410771
Most	Views on nonorganization content that is hateful	10	0.9661026	0.6609493	-1.4554829	3.3876881
Most	Views on nonorganization content that is hateful	11	0.9797780	0.5723713	-1.1172754	3.0768314
Most	Views on nonorganization content that is hateful	12	1.0140810	0.6363155	-1.3172514	3.3454134
Most	Views on nonorganization content that is hateful	13	1.0554898	0.6335917	-1.2658629	3.3768424
Most	Views on nonorganization content that is hateful	14	1.8505830	0.8567018	-1.2882004	4.9893664
Most	Views on nonorganization content that is hateful	15	1.8060590	1.4024801	-3.3323467	6.9444647
Most	Views on nonorganization content that is hateful	16	2.1292324	1.3377794	-2.7721227	7.0305875
Most	Views on nonorganization content that is hateful	17	2.3544882	1.0630393	-1.5402742	6.2492506
Most	Views on nonorganization content that is hateful	18	2.4705931	0.9254466	-0.9200576	5.8612438
Most	Views on nonorganization content that is hateful	19	2.3471045	0.7614996	-0.4428773	5.1370863
Most	Views on nonorganization content that is hateful	20	2.3111241	0.4138970	0.7946886	3.8275595
Most	Views on nonorganization content that is hateful	21	2.2428945	0.3563812	0.9371853	3.5486038
Most	Views on nonorganization content that is hateful	22	2.1016817	0.9715271	-1.4577984	5.6611618
Most	Views on nonorganization content that is hateful	23	2.4623459	1.0267681	-1.2995264	6.2242183
Most	Views on nonorganization content that is hateful	24	2.7869036	0.4345055	1.1949625	4.3788447
Most	Views on nonorganization content that is hateful	25	2.3342698	1.0357960	-1.4606787	6.1292182
Most	Views on nonorganization content that is hateful	26	2.1855252	0.8352340	-0.8746043	5.2456548
Most	Views on nonorganization content that is hateful	27	1.6852477	0.1726888	1.0525506	2.3179448
Most	Views on nonorganization content that is hateful	28	1.6811208	1.7243840	-4.6366760	7.9989177
Most	Views on nonorganization content that is hateful	29	1.3004975	0.8406449	-1.7794566	4.3804516
Most	Views on nonorganization content that is hateful	30	1.2490087	0.7632746	-1.5474761	4.0454935

sample	outcome	event.time	estimate	std.error	conf.low	conf.high
Middle	Views on nonorganization content that is hateful	-30	-0.1914055	0.1148205	-0.4815799	0.0987689
Middle	Views on nonorganization content that is hateful	-29	0.2397722	0.0408648	0.1364987	0.3430458
Middle	Views on nonorganization content that is hateful	-28	-0.0269764	0.1404495	-0.3819205	0.3279677
Middle	Views on nonorganization content that is hateful	-27	-0.0092221	0.1085922	-0.2836564	0.2652121
Middle	Views on nonorganization content that is hateful	-26	0.0331279	0.1033785	-0.2281302	0.2943861
Middle	Views on nonorganization content that is hateful	-25	-0.0730756	0.1287931	-0.3985615	0.2524103
Middle	Views on nonorganization content that is hateful	-24	-0.0117532	0.1010411	-0.2671042	0.2435979
Middle	Views on nonorganization content that is hateful	-23	-0.1253517	0.0547620	-0.2637463	0.0130429
Middle	Views on nonorganization content that is hateful	-22	-0.0833668	0.0349253	-0.1716302	0.0048966
Middle	Views on nonorganization content that is hateful	-21	0.0031844	0.1821650	-0.4571830	0.4635518
Middle	Views on nonorganization content that is hateful	-20	-0.0599485	0.2771426	-0.7603434	0.6404464
Middle	Views on nonorganization content that is hateful	-19	0.4034429	0.2692062	-0.2768953	1.0837811
Middle	Views on nonorganization content that is hateful	-18	-0.0371654	0.1467554	-0.4080459	0.3337150
Middle	Views on nonorganization content that is hateful	-17	0.4554062	0.2460421	-0.1663915	1.0772038
Middle	Views on nonorganization content that is hateful	-16	-0.0864672	0.1625144	-0.4971736	0.3242392
Middle	Views on nonorganization content that is hateful	-15	0.0088402	0.1703699	-0.4217187	0.4393991

Middle	Views on nonorganization content that is hateful	-14	0.0521910	0.1084660	-0.2219244	0.3263064
Middle	Views on nonorganization content that is hateful	-13	-0.0965775	0.0724967	-0.2797911	0.0866361
Middle	Views on nonorganization content that is hateful	-12	0.1709431	0.1569701	-0.2257519	0.5676380
Middle	Views on nonorganization content that is hateful	-11	-0.1037472	0.1816438	-0.5627975	0.3553032
Middle	Views on nonorganization content that is hateful	-10	0.1435122	0.1670872	-0.2787506	0.5657750
Middle	Views on nonorganization content that is hateful	-9	0.0611698	0.0901913	-0.1667616	0.2891012
Middle	Views on nonorganization content that is hateful	-8	-0.2165317	0.0507207	-0.3447132	-0.0883503
Middle	Views on nonorganization content that is hateful	-7	-0.2904260	0.3293011	-1.1226360	0.5417841
Middle	Views on nonorganization content that is hateful	-6	-0.3306927	0.2811440	-1.0412001	0.3798146
Middle	Views on nonorganization content that is hateful	-5	-0.1651001	0.2049818	-0.6831302	0.3529301
Middle	Views on nonorganization content that is hateful	-4	-0.3970532	0.3140300	-1.1906700	0.3965635
Middle	Views on nonorganization content that is hateful	-3	-0.6205112	0.4335789	-1.7162521	0.4752298
Middle	Views on nonorganization content that is hateful	-2	0.2889554	0.1182302	-0.0098360	0.5877468
Middle	Views on nonorganization content that is hateful	-1	0.2165388	0.2501974	-0.4157603	0.8488378
Middle	Views on nonorganization content that is hateful	0	0.3381959	0.2137404	-0.2019688	0.8783607
Middle	Views on nonorganization content that is hateful	1	0.5308671	0.3106659	-0.2542480	1.3159821
Middle	Views on nonorganization content that is hateful	2	0.5648601	0.3640627	-0.3551995	1.4849197
Middle	Views on nonorganization content that is hateful	3	0.7681828	0.3591069	-0.1393525	1.6757180
Middle	Views on nonorganization content that is hateful	4	0.8677513	0.4668312	-0.3120247	2.0475273
Middle	Views on nonorganization content that is hateful	5	0.7120408	0.4343211	-0.3855758	1.8096573
Middle	Views on nonorganization content that is hateful	6	0.6914353	0.5136659	-0.6067015	1.9895721
Middle	Views on nonorganization content that is hateful	7	0.8391273	0.7420641	-1.0362175	2.7144721
Middle	Views on nonorganization content that is hateful	8	0.7548072	0.5761658	-0.7012794	2.2108939
Middle	Views on nonorganization content that is hateful	9	0.6545793	0.5892474	-0.8345671	2.1437258
Middle	Views on nonorganization content that is hateful	10	0.9697831	0.6674291	-0.7169442	2.6565104
Middle	Views on nonorganization content that is hateful	11	1.0972453	0.6028998	-0.4264035	2.6208940
Middle	Views on nonorganization content that is hateful	12	1.1488645	0.6219532	-0.4229361	2.7206651
Middle	Views on nonorganization content that is hateful	13	1.0379485	0.6990924	-0.7287981	2.8046952
Middle	Views on nonorganization content that is hateful	14	1.6762465	1.3141525	-1.6448806	4.9973736
Middle	Views on nonorganization content that is hateful	15	1.8226630	1.0270152	-0.7728104	4.4181365
Middle	Views on nonorganization content that is hateful	16	1.8576401	0.9620685	-0.5737000	4.2889802
Middle	Views on nonorganization content that is hateful	17	2.0803015	1.4697463	-1.6340422	5.7946453
Middle	Views on nonorganization content that is hateful	18	2.2122036	1.7405497	-2.1865148	6.6109219
Middle	Views on nonorganization content that is hateful	19	1.6966564	1.3187430	-1.6360718	5.0293846
Middle	Views on nonorganization content that is hateful	20	1.6053473	0.8310563	-0.4948987	3.7055932
Middle	Views on nonorganization content that is hateful	21	1.7849138	1.0867263	-0.9614616	4.5312893
Middle	Views on nonorganization content that is hateful	22	1.6917297	0.9220971	-0.6385946	4.0220540
Middle	Views on nonorganization content that is hateful	23	1.9731661	1.0664433	-0.7219500	4.6682822
Middle	Views on nonorganization content that is hateful	24	2.0582423	1.1752616	-0.9118796	5.0283643
Middle	Views on nonorganization content that is hateful	25	2.0566874	1.2624625	-1.1338086	5.2471834
Middle	Views on nonorganization content that is hateful	26	1.5564947	1.0447896	-1.0838982	4.1968875

Middle	Views on nonorganization content that is hateful	27	0.3187777	0.5716054	-1.1257837	1.7633391
Middle	Views on nonorganization content that is hateful	28	0.8137800	1.4659557	-2.8909840	4.5185441
Middle	Views on nonorganization content that is hateful	29	0.6033556	1.3616966	-2.8379248	4.0446360
Middle	Views on nonorganization content that is hateful	30	0.0842301	0.6195870	-1.4815907	1.6500508

sample	outcome	event.time	estimate	std.error	conf.low	conf.high
Least	Views on nonorganization content that is hateful	-30	-0.0603217	0.0727624	-0.5909722	0.4703288
Least	Views on nonorganization content that is hateful	-29	0.0751493	0.1191463	-0.7937755	0.9440740
Least	Views on nonorganization content that is hateful	-28	-0.0695978	0.1558930	-1.2065135	1.0673178
Least	Views on nonorganization content that is hateful	-27	0.1304497	0.1905123	-1.2589418	1.5198412
Least	Views on nonorganization content that is hateful	-26	-0.0816879	0.2072939	-1.5934665	1.4300908
Least	Views on nonorganization content that is hateful	-25	-0.0085557	0.1109004	-0.8173442	0.8002328
Least	Views on nonorganization content that is hateful	-24	-0.0397465	0.1196669	-0.9124683	0.8329753
Least	Views on nonorganization content that is hateful	-23	-0.0200935	0.0670729	-0.5092508	0.4690639
Least	Views on nonorganization content that is hateful	-22	0.0925241	0.0559319	-0.3153834	0.5004315
Least	Views on nonorganization content that is hateful	-21	0.3030380	0.1971405	-1.1346926	1.7407686
Least	Views on nonorganization content that is hateful	-20	-0.1672153	0.0914042	-0.8338191	0.4993886
Least	Views on nonorganization content that is hateful	-19	0.3545159	0.0788046	-0.2202004	0.9292321
Least	Views on nonorganization content that is hateful	-18	0.1724139	0.3646879	-2.4872271	2.8320549
Least	Views on nonorganization content that is hateful	-17	0.1376046	0.2170400	-1.4452516	1.7204608
Least	Views on nonorganization content that is hateful	-16	0.2347430	0.2848532	-1.8426700	2.3121559
Least	Views on nonorganization content that is hateful	-15	-0.1572169	0.1969151	-1.5933035	1.2788697
Least	Views on nonorganization content that is hateful	-14	-0.0172550	0.1057476	-0.7884645	0.7539544
Least	Views on nonorganization content that is hateful	-13	-0.0343171	0.1360284	-1.0263621	0.9577280
Least	Views on nonorganization content that is hateful	-12	-0.0799715	0.1775309	-1.3746911	1.2147480
Least	Views on nonorganization content that is hateful	-11	0.0614654	0.1234021	-0.8384970	0.9614277
Least	Views on nonorganization content that is hateful	-10	-0.0733056	0.1418978	-1.1081558	0.9615445
Least	Views on nonorganization content that is hateful	-9	-0.1156899	0.1636972	-1.3095210	1.0781412
Least	Views on nonorganization content that is hateful	-8	-0.0801722	0.1860713	-1.4371762	1.2768319
Least	Views on nonorganization content that is hateful	-7	-0.1268765	0.0870201	-0.7615076	0.5077547
Least	Views on nonorganization content that is hateful	-6	-0.2595886	0.1904102	-1.6482356	1.1290585
Least	Views on nonorganization content that is hateful	-5	-0.3111008	0.1727215	-1.5707455	0.9485440
Least	Views on nonorganization content that is hateful	-4	-0.1868328	0.2328852	-1.8852471	1.5115815
Least	Views on nonorganization content that is hateful	-3	-0.6578203	0.4523398	-3.9567004	2.6410598
Least	Views on nonorganization content that is hateful	-2	0.1814642	0.1276787	-0.7496868	1.1126153
Least	Views on nonorganization content that is hateful	-1	-0.0178541	0.4766242	-3.4938381	3.4581298
Least	Views on nonorganization content that is hateful	0	0.4247612	0.2748497	-1.5796967	2.4292190
Least	Views on nonorganization content that is hateful	1	0.7097692	0.3729593	-2.0101944	3.4297329
Least	Views on nonorganization content that is hateful	2	0.6318330	0.4856690	-2.9101144	4.1737804
Least	Views on nonorganization content that is hateful	3	0.7122143	0.5856354	-3.5587805	4.9832092
Least	Views on nonorganization content that is hateful	4	0.6093433	0.6414158	-4.0684534	5.2871400

Least	Views on nonorganization content that is hateful	5	0.6173791	0.5316312	-3.2597669	4.4945251
Least	Views on nonorganization content that is hateful	6	0.7305591	0.6424051	-3.9544529	5.4155711
Least	Views on nonorganization content that is hateful	7	0.7175655	0.8476930	-5.4645953	6.8997263
Least	Views on nonorganization content that is hateful	8	0.5465023	0.7010228	-4.5660039	5.6590084
Least	Views on nonorganization content that is hateful	9	0.6388889	0.7164568	-4.5861761	5.8639539
Least	Views on nonorganization content that is hateful	10	0.8477582	0.9189073	-5.8537631	7.5492794
Least	Views on nonorganization content that is hateful	11	0.8395328	0.8868639	-5.6282986	7.3073641
Least	Views on nonorganization content that is hateful	12	0.7686375	0.9028454	-5.8157457	7.3530207
Least	Views on nonorganization content that is hateful	13	0.5706792	0.7297330	-4.7512083	5.8925667
Least	Views on nonorganization content that is hateful	14	0.8986756	0.9362450	-5.9292884	7.7266397
Least	Views on nonorganization content that is hateful	15	1.1574472	1.3243092	-8.5006392	10.8155335
Least	Views on nonorganization content that is hateful	16	1.0587553	2.4147915	-16.5521357	18.6696464
Least	Views on nonorganization content that is hateful	17	1.0600162	3.2008067	-22.2832242	24.4032566
Least	Views on nonorganization content that is hateful	18	1.1369851	1.8226133	-12.1551957	14.4291658
Least	Views on nonorganization content that is hateful	19	1.0079875	0.3105523	-1.2568468	3.2728218
Least	Views on nonorganization content that is hateful	20	0.6640732	1.3631851	-9.2775323	10.6056787
Least	Views on nonorganization content that is hateful	21	1.0024351	1.5054172	-9.9764585	11.9813287
Least	Views on nonorganization content that is hateful	22	0.9666432	2.6663505	-18.4788486	20.4121350
Least	Views on nonorganization content that is hateful	23	1.0947318	0.8186601	-4.8756942	7.0651579
Least	Views on nonorganization content that is hateful	24	1.2337642	1.5918509	-10.3754836	12.8430119
Least	Views on nonorganization content that is hateful	25	1.1291900	2.7680053	-19.0576625	21.3160425
Least	Views on nonorganization content that is hateful	26	0.8833079	2.6859671	-18.7052462	20.4718619
Least	Views on nonorganization content that is hateful	27	-1.4850305	0.0901846	-2.1427399	-0.8273211
Least	Views on nonorganization content that is hateful	28	-1.4292678	0.3182312	-3.7501039	0.8915682
Least	Views on nonorganization content that is hateful	29	-1.5645742	0.3825690	-4.3546207	1.2254722
Least	Views on nonorganization content that is hateful	30	-1.8158703	0.9998895	-9.1079889	5.4762483

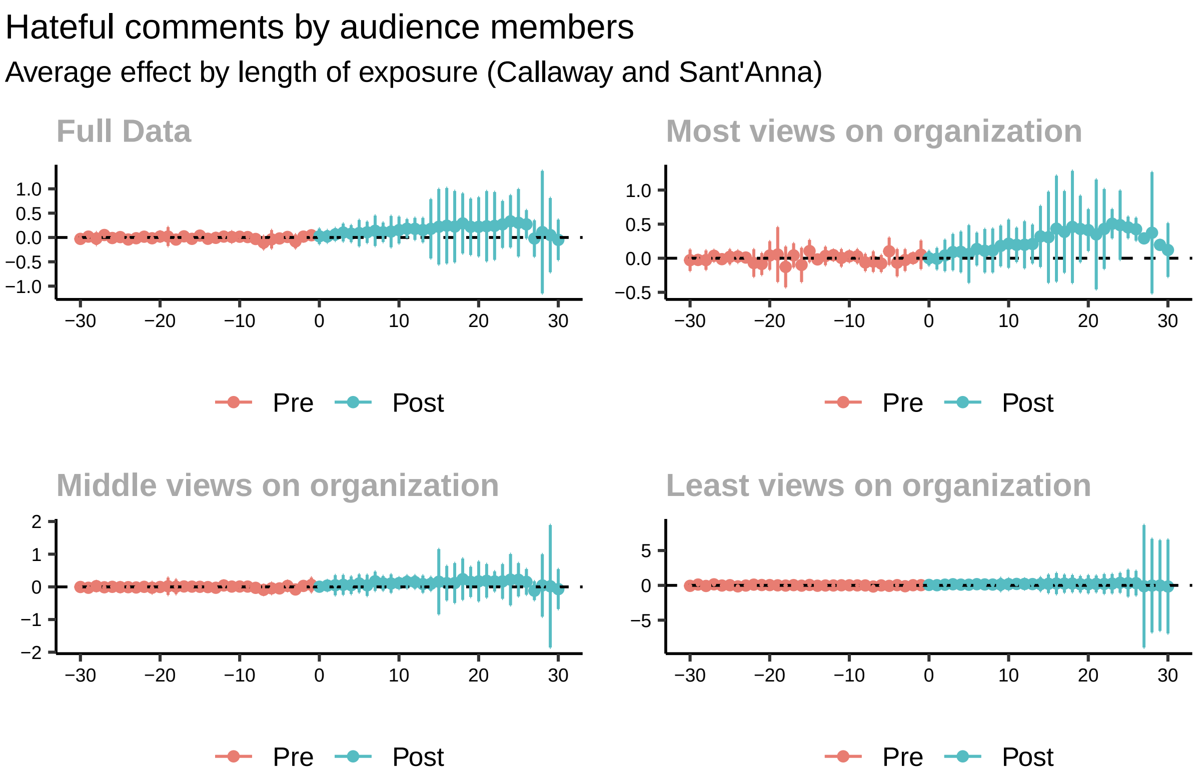

Long timeframe with sampled control groups

sample	outcome	event.time	estimate	std.error	conf.low	conf.high
Full	Hateful comments by audience members	-30	-0.0289174	0.0314492	-0.1046263	0.0467916
Full	Hateful comments by audience members	-29	0.0180176	0.0239475	-0.0396321	0.0756674
Full	Hateful comments by audience members	-28	-0.0233803	0.0541381	-0.1537091	0.1069486
Full	Hateful comments by audience members	-27	0.0505600	0.0265332	-0.0133145	0.1144345
Full	Hateful comments by audience members	-26	-0.0110180	0.0199087	-0.0589451	0.0369090
Full	Hateful comments by audience members	-25	0.0078226	0.0110240	-0.0187160	0.0343611
Full	Hateful comments by audience members	-24	-0.0410848	0.0344426	-0.1239999	0.0418304
Full	Hateful comments by audience members	-23	-0.0155055	0.0176673	-0.0580367	0.0270257
Full	Hateful comments by audience members	-22	0.0163652	0.0370832	-0.0729067	0.1056371
Full	Hateful comments by audience members	-21	-0.0161210	0.0219294	-0.0689125	0.0366705
Full	Hateful comments by audience members	-20	0.0197799	0.0424377	-0.0823821	0.1219420
Full	Hateful comments by audience members	-19	0.0187807	0.0804828	-0.1749688	0.2125302
Full	Hateful comments by audience members	-18	-0.0428122	0.0317430	-0.1192283	0.0336039
Full	Hateful comments by audience members	-17	0.0234145	0.0363429	-0.0640751	0.1109042
Full	Hateful comments by audience members	-16	-0.0275219	0.0280117	-0.0949556	0.0399118
Full	Hateful comments by audience members	-15	0.0382088	0.0263734	-0.0252810	0.1016985
Full	Hateful comments by audience members	-14	-0.0264414	0.0368216	-0.1150834	0.0622007
Full	Hateful comments by audience members	-13	-0.0075337	0.0400246	-0.1038864	0.0888191
Full	Hateful comments by audience members	-12	0.0183811	0.0322975	-0.0593701	0.0961322

Full	Hateful comments by audience members	-11	0.0098401	0.0485464	-0.1070275	0.1267077
Full	Hateful comments by audience members	-10	0.0156315	0.0334289	-0.0648433	0.0961063
Full	Hateful comments by audience members	-9	0.0083860	0.0168741	-0.0322357	0.0490077
Full	Hateful comments by audience members	-8	-0.0309071	0.0214719	-0.0825974	0.0207832
Full	Hateful comments by audience members	-7	-0.1052829	0.0572622	-0.2431326	0.0325668
Full	Hateful comments by audience members	-6	-0.0378657	0.0783105	-0.2263856	0.1506542
Full	Hateful comments by audience members	-5	-0.0200510	0.0255011	-0.0814409	0.0413390
Full	Hateful comments by audience members	-4	0.0110389	0.0269533	-0.0538468	0.0759247
Full	Hateful comments by audience members	-3	-0.0800503	0.0596824	-0.2237261	0.0636255
Full	Hateful comments by audience members	-2	0.0185058	0.0395762	-0.0767675	0.1137791
Full	Hateful comments by audience members	-1	0.0457936	0.0379958	-0.0456753	0.1372625
Full	Hateful comments by audience members	0	0.0242963	0.0662365	-0.1351575	0.1837501
Full	Hateful comments by audience members	1	0.0221233	0.0535798	-0.1068614	0.1511080
Full	Hateful comments by audience members	2	0.0625258	0.0541695	-0.0678785	0.1929301
Full	Hateful comments by audience members	3	0.1016340	0.0763607	-0.0821922	0.2854602
Full	Hateful comments by audience members	4	0.0784775	0.0721416	-0.0951920	0.2521470
Full	Hateful comments by audience members	5	0.0905501	0.1120939	-0.1792981	0.3603983
Full	Hateful comments by audience members	6	0.1064670	0.0899656	-0.1101108	0.3230448
Full	Hateful comments by audience members	7	0.1382591	0.1289445	-0.1721541	0.4486723
Full	Hateful comments by audience members	8	0.1073552	0.0825872	-0.0914603	0.3061708
Full	Hateful comments by audience members	9	0.1213918	0.1334051	-0.1997596	0.4425433
Full	Hateful comments by audience members	10	0.1522568	0.1142712	-0.1228330	0.4273465
Full	Hateful comments by audience members	11	0.1807259	0.0811303	-0.0145822	0.3760340
Full	Hateful comments by audience members	12	0.1776719	0.0936277	-0.0477218	0.4030657
Full	Hateful comments by audience members	13	0.1527510	0.1038158	-0.0971689	0.4026709
Full	Hateful comments by audience members	14	0.1779573	0.2523974	-0.4296491	0.7855637
Full	Hateful comments by audience members	15	0.2203928	0.3224667	-0.5558942	0.9966797
Full	Hateful comments by audience members	16	0.2427474	0.3218426	-0.5320372	1.0175321
Full	Hateful comments by audience members	17	0.2234109	0.3041810	-0.5088562	0.9556779
Full	Hateful comments by audience members	18	0.2897974	0.2558647	-0.3261560	0.9057508
Full	Hateful comments by audience members	19	0.2192271	0.2399388	-0.3583871	0.7968414
Full	Hateful comments by audience members	20	0.2193467	0.2515728	-0.3862745	0.8249680
Full	Hateful comments by audience members	21	0.2319685	0.2990861	-0.4880335	0.9519705
Full	Hateful comments by audience members	22	0.2382440	0.2886119	-0.4565430	0.9330311
Full	Hateful comments by audience members	23	0.2697794	0.1997548	-0.2110984	0.7506571
Full	Hateful comments by audience members	24	0.3323587	0.2222371	-0.2026416	0.8673590
Full	Hateful comments by audience members	25	0.3014303	0.2876433	-0.3910250	0.9938856
Full	Hateful comments by audience members	26	0.2702938	0.1206442	-0.0201379	0.5607255
Full	Hateful comments by audience members	27	-0.0209162	0.1550108	-0.3940799	0.3522474
Full	Hateful comments by audience members	28	0.1107772	0.5227811	-1.1477347	1.3692890
Full	Hateful comments by audience members	29	0.0492748	0.3167494	-0.7132487	0.8117983

Full	Hateful comments by audience members	30	-0.0492651	0.1718090	-0.4628678	0.3643375

sample	outcome	event.time	estimate	std.error	conf.low	conf.high
Most	Hateful comments by audience members	-30	-0.0288613	0.0612875	-0.1857019	0.1279792
Most	Hateful comments by audience members	-29	-0.0247546	0.0270633	-0.0940120	0.0445029
Most	Hateful comments by audience members	-28	-0.0274742	0.0553711	-0.1691741	0.1142257
Most	Hateful comments by audience members	-27	0.0358381	0.0334678	-0.0498092	0.1214854
Most	Hateful comments by audience members	-26	-0.0134820	0.0234244	-0.0734273	0.0464633
Most	Hateful comments by audience members	-25	0.0208550	0.0427585	-0.0885681	0.1302781
Most	Hateful comments by audience members	-24	0.0242877	0.0353812	-0.0662560	0.1148314
Most	Hateful comments by audience members	-23	0.0097829	0.0257189	-0.0560343	0.0756001
Most	Hateful comments by audience members	-22	-0.0702457	0.0785308	-0.2712133	0.1307219
Most	Hateful comments by audience members	-21	-0.0840319	0.0627649	-0.2446532	0.0765894
Most	Hateful comments by audience members	-20	0.0397728	0.0811187	-0.1678176	0.2473632
Most	Hateful comments by audience members	-19	0.0540791	0.1564523	-0.3462969	0.4544551
Most	Hateful comments by audience members	-18	-0.1271985	0.1165075	-0.4253521	0.1709551
Most	Hateful comments by audience members	-17	0.0415820	0.0690741	-0.1351850	0.2183490
Most	Hateful comments by audience members	-16	-0.0979871	0.0979640	-0.3486863	0.1527120
Most	Hateful comments by audience members	-15	0.1050976	0.0637059	-0.0579317	0.2681269
Most	Hateful comments by audience members	-14	-0.0175757	0.0229262	-0.0762460	0.0410947
Most	Hateful comments by audience members	-13	0.0329496	0.0526936	-0.1018981	0.1677974
Most	Hateful comments by audience members	-12	0.0454179	0.0314726	-0.0351235	0.1259592
Most	Hateful comments by audience members	-11	0.0048038	0.0499445	-0.1230088	0.1326164
Most	Hateful comments by audience members	-10	0.0284546	0.0335128	-0.0573079	0.1142171
Most	Hateful comments by audience members	-9	0.0287954	0.0406502	-0.0752321	0.1328230
Most	Hateful comments by audience members	-8	-0.0618800	0.0489997	-0.1872747	0.0635147
Most	Hateful comments by audience members	-7	-0.0496131	0.0584899	-0.1992942	0.1000679
Most	Hateful comments by audience members	-6	-0.0776044	0.0486558	-0.2021191	0.0469102
Most	Hateful comments by audience members	-5	0.1019770	0.0779105	-0.0974032	0.3013571
Most	Hateful comments by audience members	-4	-0.0653540	0.0776924	-0.2641761	0.1334682
Most	Hateful comments by audience members	-3	-0.0266167	0.0618583	-0.1849180	0.1316845
Most	Hateful comments by audience members	-2	0.0014351	0.0315001	-0.0791767	0.0820468
Most	Hateful comments by audience members	-1	0.0511068	0.0816636	-0.1578780	0.2600916
Most	Hateful comments by audience members	0	0.0004692	0.0419555	-0.1068988	0.1078373
Most	Hateful comments by audience members	1	-0.0069836	0.0617656	-0.1650476	0.1510805
Most	Hateful comments by audience members	2	0.0408908	0.0894948	-0.1881348	0.2699164
Most	Hateful comments by audience members	3	0.0905768	0.1036869	-0.1747677	0.3559213
Most	Hateful comments by audience members	4	0.0931323	0.1165732	-0.2051894	0.3914541
Most	Hateful comments by audience members	5	0.0622541	0.1646764	-0.3591681	0.4836764
Most	Hateful comments by audience members	6	0.1349677	0.0933549	-0.1039362	0.3738715
Most	Hateful comments by audience members	7	0.1092327	0.1244080	-0.2091389	0.4276043

Most	Hateful comments by audience members	8	0.1135330	0.1257735	-0.2083333	0.4353992
Most	Hateful comments by audience members	9	0.1808123	0.1163912	-0.1170436	0.4786682
Most	Hateful comments by audience members	10	0.2131746	0.1376553	-0.1390982	0.5654474
Most	Hateful comments by audience members	11	0.1961634	0.0980263	-0.0546950	0.4470217
Most	Hateful comments by audience members	12	0.1974124	0.1343393	-0.1463745	0.5411994
Most	Hateful comments by audience members	13	0.2086881	0.1112654	-0.0760504	0.4934266
Most	Hateful comments by audience members	14	0.3201240	0.1747018	-0.1269543	0.7672024
Most	Hateful comments by audience members	15	0.3078876	0.2606551	-0.3591530	0.9749283
Most	Hateful comments by audience members	16	0.4334710	0.3031894	-0.3424191	1.2093610
Most	Hateful comments by audience members	17	0.3865876	0.2336113	-0.2112456	0.9844208
Most	Hateful comments by audience members	18	0.4594357	0.3211568	-0.3624346	1.2813060
Most	Hateful comments by audience members	19	0.4293814	0.1900762	-0.0570413	0.9158042
Most	Hateful comments by audience members	20	0.4130662	0.1192053	0.1080086	0.7181237
Most	Hateful comments by audience members	21	0.3495645	0.3143800	-0.4549633	1.1540924
Most	Hateful comments by audience members	22	0.4296563	0.2283138	-0.1546201	1.0139327
Most	Hateful comments by audience members	23	0.5064296	0.0834122	0.2929699	0.7198893
Most	Hateful comments by audience members	24	0.4852589	0.1983070	-0.0222272	0.9927449
Most	Hateful comments by audience members	25	0.4518920	0.0616045	0.2942404	0.6095436
Most	Hateful comments by audience members	26	0.4255028	0.0653190	0.2583455	0.5926601
Most	Hateful comments by audience members	27	0.2914080	0.0027349	0.2844091	0.2984070
Most	Hateful comments by audience members	28	0.3735811	0.3471056	-0.5146947	1.2618569
Most	Hateful comments by audience members	29	0.1958127	0.0270658	0.1265489	0.2650765
Most	Hateful comments by audience members	30	0.1203320	0.1534882	-0.2724586	0.5131225

sample	outcome	event.time	estimate	std.error	conf.low	conf.high
Middle	Hateful comments by audience members	-30	-0.0038988	0.0381813	-0.1061554	0.0983579
Middle	Hateful comments by audience members	-29	-0.0274379	0.0321845	-0.1136340	0.0587582
Middle	Hateful comments by audience members	-28	0.0222235	0.0565859	-0.1293241	0.1737710
Middle	Hateful comments by audience members	-27	-0.0125615	0.0271347	-0.0852331	0.0601102
Middle	Hateful comments by audience members	-26	0.0010886	0.0288383	-0.0761456	0.0783229
Middle	Hateful comments by audience members	-25	-0.0090095	0.0553815	-0.1573315	0.1393125
Middle	Hateful comments by audience members	-24	-0.0097245	0.0258628	-0.0789898	0.0595408
Middle	Hateful comments by audience members	-23	-0.0183050	0.0178598	-0.0661369	0.0295269
Middle	Hateful comments by audience members	-22	0.0010225	0.0283130	-0.0748049	0.0768500
Middle	Hateful comments by audience members	-21	-0.0262582	0.0659638	-0.2029215	0.1504051
Middle	Hateful comments by audience members	-20	-0.0036487	0.0424313	-0.1172875	0.1099901
Middle	Hateful comments by audience members	-19	0.0176004	0.0980023	-0.2448680	0.2800687
Middle	Hateful comments by audience members	-18	0.0032444	0.0838530	-0.2213296	0.2278183
Middle	Hateful comments by audience members	-17	0.0123556	0.0274633	-0.0611961	0.0859072
Middle	Hateful comments by audience members	-16	0.0074732	0.0214767	-0.0500454	0.0649917
Middle	Hateful comments by audience members	-15	0.0017915	0.0455993	-0.1203319	0.1239149

Middle	Hateful comments by audience members	-14	-0.0068769	0.0225402	-0.0672438	0.0534901
Middle	Hateful comments by audience members	-13	-0.0276814	0.0486223	-0.1579010	0.1025383
Middle	Hateful comments by audience members	-12	0.0432839	0.0539033	-0.1010791	0.1876469
Middle	Hateful comments by audience members	-11	0.0090365	0.0432308	-0.1067437	0.1248167
Middle	Hateful comments by audience members	-10	0.0110684	0.0230199	-0.0505831	0.0727199
Middle	Hateful comments by audience members	-9	0.0090397	0.0255550	-0.0594013	0.0774808
Middle	Hateful comments by audience members	-8	-0.0303132	0.0285840	-0.1068664	0.0462401
Middle	Hateful comments by audience members	-7	-0.0925278	0.0572680	-0.2459021	0.0608465
Middle	Hateful comments by audience members	-6	-0.0459081	0.0645296	-0.2187304	0.1269143
Middle	Hateful comments by audience members	-5	-0.0469034	0.0382438	-0.1493273	0.0555204
Middle	Hateful comments by audience members	-4	0.0283829	0.0587575	-0.1289806	0.1857465
Middle	Hateful comments by audience members	-3	-0.0803053	0.0405752	-0.1889731	0.0283625
Middle	Hateful comments by audience members	-2	0.0285244	0.0416609	-0.0830512	0.1401000
Middle	Hateful comments by audience members	-1	0.0586052	0.0845078	-0.1677224	0.2849328
Middle	Hateful comments by audience members	0	0.0041874	0.0401539	-0.1033521	0.1117270
Middle	Hateful comments by audience members	1	0.0392861	0.0614482	-0.1252834	0.2038556
Middle	Hateful comments by audience members	2	0.0496862	0.1140468	-0.2557522	0.3551246
Middle	Hateful comments by audience members	3	0.0623322	0.1128543	-0.2399125	0.3645768
Middle	Hateful comments by audience members	4	0.0504121	0.1003610	-0.2183733	0.3191975
Middle	Hateful comments by audience members	5	0.1045752	0.1031765	-0.1717507	0.3809010
Middle	Hateful comments by audience members	6	0.0459393	0.1200204	-0.2754975	0.3673760
Middle	Hateful comments by audience members	7	0.1705285	0.1093344	-0.1222893	0.4633463
Middle	Hateful comments by audience members	8	0.1077619	0.0832599	-0.1152234	0.3307472
Middle	Hateful comments by audience members	9	0.1018206	0.1045549	-0.1781967	0.3818380
Middle	Hateful comments by audience members	10	0.1130999	0.0661582	-0.0640840	0.2902838
Middle	Hateful comments by audience members	11	0.1504599	0.0751134	-0.0507078	0.3516275
Middle	Hateful comments by audience members	12	0.1482517	0.0768021	-0.0574384	0.3539419
Middle	Hateful comments by audience members	13	0.0848965	0.0979491	-0.1774294	0.3472224
Middle	Hateful comments by audience members	14	0.0921543	0.0794025	-0.1205004	0.3048090
Middle	Hateful comments by audience members	15	0.1577759	0.3715984	-0.8374333	1.1529852
Middle	Hateful comments by audience members	16	0.1136778	0.1962423	-0.4118953	0.6392510
Middle	Hateful comments by audience members	17	0.1172197	0.2270436	-0.4908451	0.7252845
Middle	Hateful comments by audience members	18	0.2360495	0.2352867	-0.3940917	0.8661908
Middle	Hateful comments by audience members	19	0.1518625	0.1715877	-0.3076811	0.6114061
Middle	Hateful comments by audience members	20	0.1678500	0.2270177	-0.4401453	0.7758453
Middle	Hateful comments by audience members	21	0.1843496	0.1910927	-0.3274320	0.6961312
Middle	Hateful comments by audience members	22	0.1661815	0.1146237	-0.1408019	0.4731649
Middle	Hateful comments by audience members	23	0.1666788	0.1969558	-0.3608054	0.6941630
Middle	Hateful comments by audience members	24	0.2216028	0.2921089	-0.5607188	1.0039245
Middle	Hateful comments by audience members	25	0.2171982	0.1892497	-0.2896476	0.7240440
Middle	Hateful comments by audience members	26	0.1538274	0.1466517	-0.2389330	0.5465879

Middle	Hateful comments by audience members	27	-0.1186681	0.1072229	-0.4058307	0.1684946
Middle	Hateful comments by audience members	28	0.0429694	0.3558852	-0.9101571	0.9960958
Middle	Hateful comments by audience members	29	0.0171819	0.6994934	-1.8561908	1.8905546
Middle	Hateful comments by audience members	30	-0.0690752	0.2274385	-0.6781975	0.5400471

sample	outcome	event.time	estimate	std.error	conf.low	conf.high
Least	Hateful comments by audience members	-30	-0.0682579	0.0646271	-0.2330178	0.0965020
Least	Hateful comments by audience members	-29	0.1149054	0.0588567	-0.0351436	0.2649544
Least	Hateful comments by audience members	-28	-0.0861085	0.0953705	-0.3292453	0.1570283
Least	Hateful comments by audience members	-27	0.1474909	0.0414922	0.0417109	0.2532708
Least	Hateful comments by audience members	-26	-0.0247494	0.0839120	-0.2386741	0.1891753
Least	Hateful comments by audience members	-25	0.0236540	0.0606993	-0.1310923	0.1784004
Least	Hateful comments by audience members	-24	-0.1402574	0.1015522	-0.3991539	0.1186391
Least	Hateful comments by audience members	-23	-0.0267994	0.0252072	-0.0910624	0.0374636
Least	Hateful comments by audience members	-22	0.1040053	0.0619038	-0.0538117	0.2618223
Least	Hateful comments by audience members	-21	0.0449858	0.1003842	-0.2109331	0.3009047
Least	Hateful comments by audience members	-20	0.0372477	0.0966326	-0.2091068	0.2836022
Least	Hateful comments by audience members	-19	0.0026906	0.1341889	-0.3394097	0.3447909
Least	Hateful comments by audience members	-18	-0.0301051	0.0873773	-0.2528641	0.1926540
Least	Hateful comments by audience members	-17	0.0363402	0.0476120	-0.0850414	0.1577218
Least	Hateful comments by audience members	-16	-0.0438475	0.0426301	-0.1525283	0.0648334
Least	Hateful comments by audience members	-15	0.0595390	0.0576871	-0.0875280	0.2066061
Least	Hateful comments by audience members	-14	-0.0569095	0.0775993	-0.2547407	0.1409217
Least	Hateful comments by audience members	-13	-0.0198110	0.1003527	-0.2756494	0.2360274
Least	Hateful comments by audience members	-12	-0.0137792	0.0706051	-0.1937794	0.1662211
Least	Hateful comments by audience members	-11	0.0114299	0.0572637	-0.1345576	0.1574175
Least	Hateful comments by audience members	-10	0.0093059	0.0964486	-0.2365795	0.2551913
Least	Hateful comments by audience members	-9	-0.0027020	0.0633167	-0.1641212	0.1587173
Least	Hateful comments by audience members	-8	-0.0102232	0.0868701	-0.2316892	0.2112428
Least	Hateful comments by audience members	-7	-0.1637210	0.0809254	-0.3700318	0.0425897
Least	Hateful comments by audience members	-6	-0.0064121	0.1325821	-0.3444160	0.3315918
Least	Hateful comments by audience members	-5	-0.0739547	0.0507798	-0.2034123	0.0555030
Least	Hateful comments by audience members	-4	0.0302607	0.0869719	-0.1914649	0.2519863
Least	Hateful comments by audience members	-3	-0.1217855	0.1523457	-0.5101747	0.2666036
Least	Hateful comments by audience members	-2	0.0245657	0.2067176	-0.5024386	0.5515700
Least	Hateful comments by audience members	-1	0.0288192	0.0969320	-0.2182986	0.2759370
Least	Hateful comments by audience members	0	0.0610021	0.2072382	-0.4673294	0.5893336
Least	Hateful comments by audience members	1	0.0246700	0.1065393	-0.2469405	0.2962805
Least	Hateful comments by audience members	2	0.0984401	0.1628319	-0.3166826	0.5135627
Least	Hateful comments by audience members	3	0.1583868	0.2384338	-0.4494747	0.7662482
Least	Hateful comments by audience members	4	0.1017113	0.1658208	-0.3210312	0.5244538

Least	Hateful comments by audience members	5	0.0904864	0.1304678	-0.2421272	0.4231000
Least	Hateful comments by audience members	6	0.1675827	0.1893663	-0.3151865	0.6503519
Least	Hateful comments by audience members	7	0.1320110	0.2500595	-0.5054889	0.7695110
Least	Hateful comments by audience members	8	0.1146938	0.1738638	-0.3285534	0.5579410
Least	Hateful comments by audience members	9	0.1167078	0.3738056	-0.8362695	1.0696851
Least	Hateful comments by audience members	10	0.1697073	0.3279484	-0.6663621	1.0057767
Least	Hateful comments by audience members	11	0.2023134	0.1267897	-0.1209235	0.5255502
Least	Hateful comments by audience members	12	0.1994755	0.3036539	-0.5746576	0.9736087
Least	Hateful comments by audience members	13	0.1816195	0.2369923	-0.4225669	0.7858059
Least	Hateful comments by audience members	14	0.1671488	0.3904963	-0.8283797	1.1626773
Least	Hateful comments by audience members	15	0.2253692	0.5213338	-1.1037154	1.5544538
Least	Hateful comments by audience members	16	0.2611294	0.5942170	-1.2537633	1.7760221
Least	Hateful comments by audience members	17	0.2512985	0.5059632	-1.0386006	1.5411976
Least	Hateful comments by audience members	18	0.2549580	0.4821058	-0.9741191	1.4840351
Least	Hateful comments by audience members	19	0.1611083	0.4617998	-1.0162008	1.3384175
Least	Hateful comments by audience members	20	0.1625136	0.5066267	-1.1290770	1.4541041
Least	Hateful comments by audience members	21	0.2081670	0.4681893	-0.9854315	1.4017655
Least	Hateful comments by audience members	22	0.2010400	0.5531928	-1.2092659	1.6113458
Least	Hateful comments by audience members	23	0.2439926	0.5456599	-1.1471090	1.6350941
Least	Hateful comments by audience members	24	0.3672804	0.5571002	-1.0529869	1.7875477
Least	Hateful comments by audience members	25	0.3148917	0.7544200	-1.6084212	2.2382047
Least	Hateful comments by audience members	26	0.3164852	0.6905090	-1.4438935	2.0768640
Least	Hateful comments by audience members	27	-0.1341520	3.4478606	-8.9241036	8.6557995
Least	Hateful comments by audience members	28	-0.0328365	2.6323811	-6.7438103	6.6781374
Least	Hateful comments by audience members	29	-0.0226299	2.5466596	-6.5150661	6.4698062
Least	Hateful comments by audience members	30	-0.1637395	2.6401916	-6.8946253	6.5671464

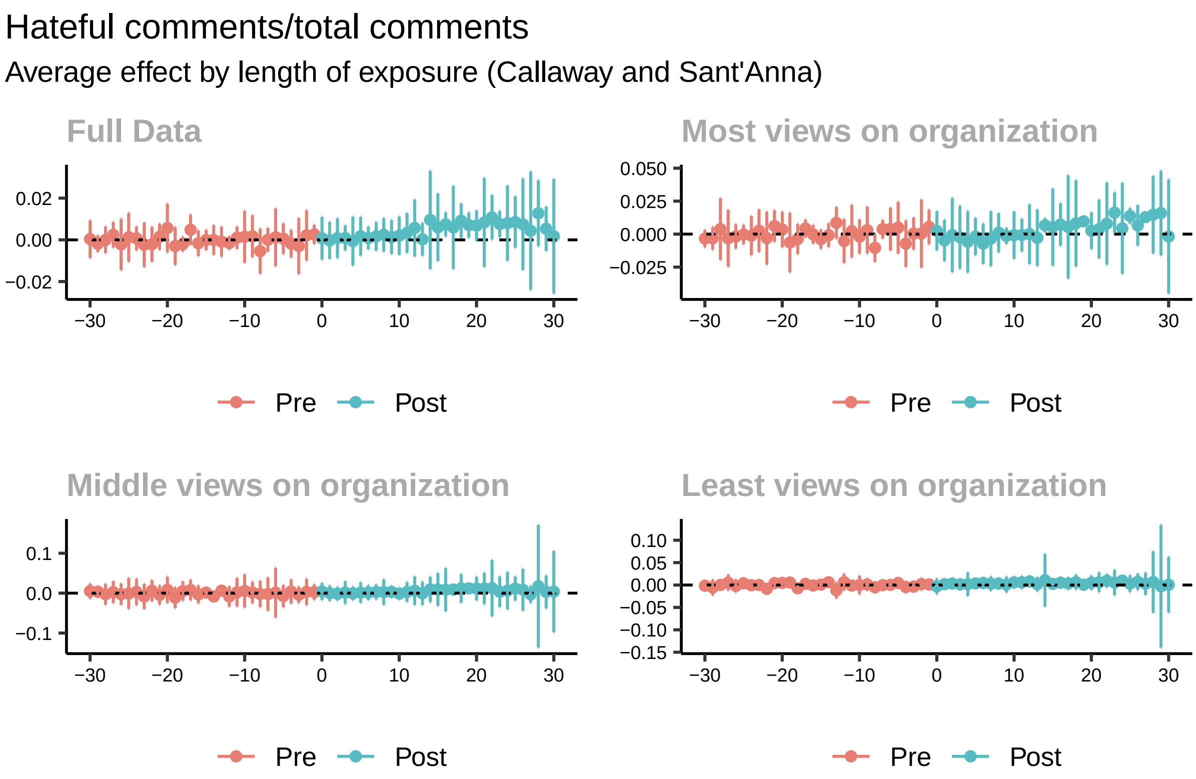

Long timeframe with sampled control groups

sample	outcome	event.time	estimate	std.error	conf.low	conf.high
Full	Hateful comments/total comments	-30	0.0003427	0.0040921	-0.0086376	0.0093230
Full	Hateful comments/total comments	-29	-0.0018602	0.0018098	-0.0058320	0.0021115
Full	Hateful comments/total comments	-28	0.0000053	0.0028866	-0.0063294	0.0063401
Full	Hateful comments/total comments	-27	0.0023820	0.0027672	-0.0036909	0.0084549
Full	Hateful comments/total comments	-26	-0.0022088	0.0055796	-0.0144536	0.0100359
Full	Hateful comments/total comments	-25	0.0012347	0.0053143	-0.0104278	0.0128971
Full	Hateful comments/total comments	-24	0.0006642	0.0025672	-0.0049696	0.0062980
Full	Hateful comments/total comments	-23	-0.0023867	0.0048320	-0.0129909	0.0082175
Full	Hateful comments/total comments	-22	-0.0021216	0.0037967	-0.0104538	0.0062106
Full	Hateful comments/total comments	-21	0.0014993	0.0028280	-0.0047070	0.0077055
Full	Hateful comments/total comments	-20	0.0056503	0.0052858	-0.0059498	0.0172503
Full	Hateful comments/total comments	-19	-0.0029857	0.0040855	-0.0119515	0.0059801
Full	Hateful comments/total comments	-18	-0.0020214	0.0015966	-0.0055253	0.0014825
Full	Hateful comments/total comments	-17	0.0048299	0.0032903	-0.0023909	0.0120507
Full	Hateful comments/total comments	-16	-0.0016476	0.0027814	-0.0077516	0.0044563
Full	Hateful comments/total comments	-15	-0.0000720	0.0021881	-0.0048739	0.0047298
Full	Hateful comments/total comments	-14	-0.0000695	0.0031934	-0.0070776	0.0069387
Full	Hateful comments/total comments	-13	-0.0008524	0.0032409	-0.0079648	0.0062600
Full	Hateful comments/total comments	-12	-0.0014916	0.0014273	-0.0046240	0.0016408

Full	Hateful comments/total comments	-11	0.0009831	0.0024148	-0.0043163	0.0062825
Full	Hateful comments/total comments	-10	0.0014709	0.0056459	-0.0109193	0.0138612
Full	Hateful comments/total comments	-9	0.0017453	0.0045653	-0.0082735	0.0117642
Full	Hateful comments/total comments	-8	-0.0054152	0.0049061	-0.0161820	0.0053515
Full	Hateful comments/total comments	-7	0.0000234	0.0025734	-0.0056241	0.0056708
Full	Hateful comments/total comments	-6	0.0012044	0.0062797	-0.0125768	0.0149855
Full	Hateful comments/total comments	-5	0.0005644	0.0033323	-0.0067484	0.0078773
Full	Hateful comments/total comments	-4	-0.0018181	0.0029926	-0.0083855	0.0047493
Full	Hateful comments/total comments	-3	-0.0030097	0.0060933	-0.0163818	0.0103625
Full	Hateful comments/total comments	-2	0.0021428	0.0054533	-0.0098247	0.0141104
Full	Hateful comments/total comments	-1	0.0025949	0.0020570	-0.0019194	0.0071091
Full	Hateful comments/total comments	0	0.0007445	0.0046584	-0.0094786	0.0109677
Full	Hateful comments/total comments	1	-0.0002601	0.0040213	-0.0090850	0.0085648
Full	Hateful comments/total comments	2	0.0007648	0.0043222	-0.0087206	0.0102502
Full	Hateful comments/total comments	3	0.0008851	0.0027001	-0.0050404	0.0068107
Full	Hateful comments/total comments	4	-0.0006230	0.0052997	-0.0122535	0.0110076
Full	Hateful comments/total comments	5	0.0017403	0.0041934	-0.0074624	0.0109431
Full	Hateful comments/total comments	6	0.0008925	0.0024548	-0.0044946	0.0062797
Full	Hateful comments/total comments	7	0.0016512	0.0031010	-0.0051541	0.0084564
Full	Hateful comments/total comments	8	0.0024246	0.0036257	-0.0055322	0.0103814
Full	Hateful comments/total comments	9	0.0013376	0.0036898	-0.0067599	0.0094351
Full	Hateful comments/total comments	10	0.0019754	0.0041514	-0.0071350	0.0110859
Full	Hateful comments/total comments	11	0.0032995	0.0043279	-0.0061983	0.0127972
Full	Hateful comments/total comments	12	0.0056997	0.0062132	-0.0079355	0.0193349
Full	Hateful comments/total comments	13	0.0002709	0.0035741	-0.0075726	0.0081144
Full	Hateful comments/total comments	14	0.0095882	0.0107006	-0.0138949	0.0330712
Full	Hateful comments/total comments	15	0.0060024	0.0073494	-0.0101262	0.0221311
Full	Hateful comments/total comments	16	0.0074096	0.0025970	0.0017103	0.0131089
Full	Hateful comments/total comments	17	0.0059439	0.0090551	-0.0139280	0.0258158
Full	Hateful comments/total comments	18	0.0091861	0.0037281	0.0010045	0.0173676
Full	Hateful comments/total comments	19	0.0070783	0.0027534	0.0010358	0.0131207
Full	Hateful comments/total comments	20	0.0068367	0.0032905	-0.0003846	0.0140579
Full	Hateful comments/total comments	21	0.0083544	0.0096837	-0.0128971	0.0296059
Full	Hateful comments/total comments	22	0.0107810	0.0048421	0.0001547	0.0214072
Full	Hateful comments/total comments	23	0.0076059	0.0028519	0.0013473	0.0138646
Full	Hateful comments/total comments	24	0.0079898	0.0081906	-0.0099850	0.0259645
Full	Hateful comments/total comments	25	0.0085647	0.0056019	-0.0037291	0.0208584
Full	Hateful comments/total comments	26	0.0074711	0.0099761	-0.0144221	0.0293643
Full	Hateful comments/total comments	27	0.0043665	0.0129199	-0.0239871	0.0327201
Full	Hateful comments/total comments	28	0.0127499	0.0071600	-0.0029631	0.0284629
Full	Hateful comments/total comments	29	0.0053159	0.0048047	-0.0052282	0.0158601

Full	Hateful comments/total comments	30	0.0017499	0.0124779	-0.0256336	0.0291334

sample	outcome	event.time	estimate	std.error	conf.low	conf.high
Most	Hateful comments/total comments	-30	-0.0033851	0.0027334	-0.0098843	0.0031141
Most	Hateful comments/total comments	-29	-0.0032034	0.0036063	-0.0117782	0.0053714
Most	Hateful comments/total comments	-28	0.0037595	0.0098010	-0.0195444	0.0270634
Most	Hateful comments/total comments	-27	-0.0031419	0.0090329	-0.0246194	0.0183357
Most	Hateful comments/total comments	-26	-0.0017346	0.0038469	-0.0108813	0.0074122
Most	Hateful comments/total comments	-25	-0.0003223	0.0030682	-0.0076176	0.0069731
Most	Hateful comments/total comments	-24	-0.0011599	0.0061747	-0.0158416	0.0135218
Most	Hateful comments/total comments	-23	0.0025599	0.0067541	-0.0134993	0.0186191
Most	Hateful comments/total comments	-22	-0.0030478	0.0083691	-0.0229471	0.0168514
Most	Hateful comments/total comments	-21	0.0061379	0.0049811	-0.0057058	0.0179817
Most	Hateful comments/total comments	-20	0.0034716	0.0056314	-0.0099182	0.0168615
Most	Hateful comments/total comments	-19	-0.0063961	0.0094614	-0.0288926	0.0161005
Most	Hateful comments/total comments	-18	-0.0036512	0.0047784	-0.0150130	0.0077106
Most	Hateful comments/total comments	-17	0.0042455	0.0027932	-0.0023959	0.0108870
Most	Hateful comments/total comments	-16	-0.0002031	0.0029382	-0.0071893	0.0067831
Most	Hateful comments/total comments	-15	-0.0038049	0.0030453	-0.0110457	0.0034359
Most	Hateful comments/total comments	-14	-0.0008143	0.0037531	-0.0097381	0.0081094
Most	Hateful comments/total comments	-13	0.0085316	0.0050013	-0.0033601	0.0204234
Most	Hateful comments/total comments	-12	-0.0052838	0.0069623	-0.0218382	0.0112706
Most	Hateful comments/total comments	-11	0.0022225	0.0083693	-0.0176773	0.0221223
Most	Hateful comments/total comments	-10	-0.0018456	0.0053876	-0.0146558	0.0109646
Most	Hateful comments/total comments	-9	0.0030314	0.0074592	-0.0147045	0.0207673
Most	Hateful comments/total comments	-8	-0.0104076	0.0044351	-0.0209531	0.0001379
Most	Hateful comments/total comments	-7	0.0037648	0.0029323	-0.0032072	0.0107369
Most	Hateful comments/total comments	-6	0.0038256	0.0067920	-0.0123238	0.0199750
Most	Hateful comments/total comments	-5	0.0048751	0.0082031	-0.0146296	0.0243799
Most	Hateful comments/total comments	-4	-0.0072300	0.0073869	-0.0247940	0.0103340
Most	Hateful comments/total comments	-3	0.0004246	0.0050654	-0.0116195	0.0124687
Most	Hateful comments/total comments	-2	0.0004273	0.0108317	-0.0253275	0.0261821
Most	Hateful comments/total comments	-1	0.0054383	0.0054763	-0.0075829	0.0184595
Most	Hateful comments/total comments	0	0.0024916	0.0061848	-0.0122140	0.0171972
Most	Hateful comments/total comments	1	-0.0047891	0.0065818	-0.0204388	0.0108607
Most	Hateful comments/total comments	2	-0.0007736	0.0117850	-0.0287950	0.0272478
Most	Hateful comments/total comments	3	-0.0023975	0.0099913	-0.0261540	0.0213590
Most	Hateful comments/total comments	4	-0.0058314	0.0097484	-0.0290102	0.0173474
Most	Hateful comments/total comments	5	-0.0018492	0.0059019	-0.0158822	0.0121838
Most	Hateful comments/total comments	6	-0.0072099	0.0064019	-0.0224319	0.0080121
Most	Hateful comments/total comments	7	-0.0034209	0.0086944	-0.0240936	0.0172518

Most	Hateful comments/total comments	8	0.0010523	0.0061941	-0.0136754	0.0157800
Most	Hateful comments/total comments	9	-0.0013171	0.0025637	-0.0074128	0.0047786
Most	Hateful comments/total comments	10	-0.0007614	0.0075228	-0.0186484	0.0171256
Most	Hateful comments/total comments	11	-0.0007441	0.0051620	-0.0130178	0.0115296
Most	Hateful comments/total comments	12	-0.0000288	0.0094796	-0.0225685	0.0225109
Most	Hateful comments/total comments	13	-0.0027645	0.0089805	-0.0241175	0.0185886
Most	Hateful comments/total comments	14	0.0066573	0.0022288	0.0013578	0.0119567
Most	Hateful comments/total comments	15	0.0053129	0.0122551	-0.0238261	0.0344520
Most	Hateful comments/total comments	16	0.0072735	0.0067130	-0.0086881	0.0232350
Most	Hateful comments/total comments	17	0.0054306	0.0164512	-0.0336857	0.0445469
Most	Hateful comments/total comments	18	0.0081859	0.0136940	-0.0243746	0.0407464
Most	Hateful comments/total comments	19	0.0096725	0.0003959	0.0087313	0.0106138
Most	Hateful comments/total comments	20	0.0025208	0.0058060	-0.0112841	0.0163257
Most	Hateful comments/total comments	21	0.0038371	0.0093285	-0.0183435	0.0260176
Most	Hateful comments/total comments	22	0.0079324	0.0130430	-0.0230801	0.0389449
Most	Hateful comments/total comments	23	0.0162701	0.0064253	0.0009926	0.0315476
Most	Hateful comments/total comments	24	0.0043832	0.0144941	-0.0300797	0.0388461
Most	Hateful comments/total comments	25	0.0137789	0.0024507	0.0079519	0.0196060
Most	Hateful comments/total comments	26	0.0066202	0.0064178	-0.0086395	0.0218800
Most	Hateful comments/total comments	27	0.0128945	0.0006184	0.0114242	0.0143648
Most	Hateful comments/total comments	28	0.0147493	0.0123312	-0.0145707	0.0440693
Most	Hateful comments/total comments	29	0.0159912	0.0134354	-0.0159543	0.0479368
Most	Hateful comments/total comments	30	-0.0017683	0.0181262	-0.0448672	0.0413306

sample	outcome	event.time	estimate	std.error	conf.low	conf.high
Middle	Hateful comments/total comments	-30	0.0049522	0.0045641	-0.0136677	0.0235721
Middle	Hateful comments/total comments	-29	0.0034093	0.0032887	-0.0100076	0.0168262
Middle	Hateful comments/total comments	-28	-0.0027224	0.0063643	-0.0286864	0.0232416
Middle	Hateful comments/total comments	-27	0.0024080	0.0066073	-0.0245475	0.0293635
Middle	Hateful comments/total comments	-26	-0.0026831	0.0064295	-0.0289131	0.0235469
Middle	Hateful comments/total comments	-25	-0.0008526	0.0094040	-0.0392176	0.0375125
Middle	Hateful comments/total comments	-24	0.0031996	0.0079793	-0.0293532	0.0357523
Middle	Hateful comments/total comments	-23	-0.0083884	0.0075845	-0.0393307	0.0225540
Middle	Hateful comments/total comments	-22	0.0060324	0.0064443	-0.0202581	0.0323229
Middle	Hateful comments/total comments	-21	-0.0046154	0.0058593	-0.0285191	0.0192884
Middle	Hateful comments/total comments	-20	0.0082363	0.0079671	-0.0242668	0.0407395
Middle	Hateful comments/total comments	-19	-0.0112183	0.0063889	-0.0372828	0.0148462
Middle	Hateful comments/total comments	-18	0.0051857	0.0058433	-0.0186531	0.0290244
Middle	Hateful comments/total comments	-17	0.0078057	0.0062580	-0.0177247	0.0333361
Middle	Hateful comments/total comments	-16	-0.0030358	0.0054244	-0.0251656	0.0190940
Middle	Hateful comments/total comments	-15	0.0015418	0.0029513	-0.0104986	0.0135822

Middle	Hateful comments/total comments	-14	-0.0083538	0.0029088	-0.0202208	0.0035133
Middle	Hateful comments/total comments	-13	0.0062483	0.0023961	-0.0035271	0.0160238
Middle	Hateful comments/total comments	-12	-0.0083037	0.0061506	-0.0333959	0.0167886
Middle	Hateful comments/total comments	-11	0.0021911	0.0087369	-0.0334523	0.0378346
Middle	Hateful comments/total comments	-10	0.0054397	0.0100634	-0.0356153	0.0464948
Middle	Hateful comments/total comments	-9	0.0015373	0.0065617	-0.0252324	0.0283069
Middle	Hateful comments/total comments	-8	-0.0020319	0.0080082	-0.0347028	0.0306390
Middle	Hateful comments/total comments	-7	-0.0022793	0.0101839	-0.0438262	0.0392677
Middle	Hateful comments/total comments	-6	0.0012747	0.0151774	-0.0606439	0.0631934
Middle	Hateful comments/total comments	-5	-0.0074142	0.0067818	-0.0350818	0.0202534
Middle	Hateful comments/total comments	-4	0.0042114	0.0073386	-0.0257278	0.0341505
Middle	Hateful comments/total comments	-3	-0.0041503	0.0049733	-0.0244397	0.0161391
Middle	Hateful comments/total comments	-2	0.0031671	0.0080376	-0.0296238	0.0359579
Middle	Hateful comments/total comments	-1	0.0026862	0.0046335	-0.0162167	0.0215891
Middle	Hateful comments/total comments	0	0.0040568	0.0052277	-0.0172705	0.0253841
Middle	Hateful comments/total comments	1	0.0000141	0.0047083	-0.0191940	0.0192222
Middle	Hateful comments/total comments	2	-0.0015730	0.0041267	-0.0184085	0.0152625
Middle	Hateful comments/total comments	3	0.0014331	0.0069253	-0.0268199	0.0296860
Middle	Hateful comments/total comments	4	-0.0006683	0.0041127	-0.0174467	0.0161101
Middle	Hateful comments/total comments	5	0.0013886	0.0061743	-0.0238003	0.0265775
Middle	Hateful comments/total comments	6	0.0011987	0.0045297	-0.0172810	0.0196784
Middle	Hateful comments/total comments	7	0.0030320	0.0045154	-0.0153892	0.0214533
Middle	Hateful comments/total comments	8	0.0027227	0.0077709	-0.0289800	0.0344254
Middle	Hateful comments/total comments	9	0.0031434	0.0035253	-0.0112386	0.0175253
Middle	Hateful comments/total comments	10	-0.0011714	0.0035866	-0.0158035	0.0134608
Middle	Hateful comments/total comments	11	0.0030015	0.0060254	-0.0215800	0.0275831
Middle	Hateful comments/total comments	12	0.0060611	0.0087015	-0.0294382	0.0415604
Middle	Hateful comments/total comments	13	-0.0004778	0.0070903	-0.0294037	0.0284481
Middle	Hateful comments/total comments	14	0.0092896	0.0077252	-0.0222266	0.0408059
Middle	Hateful comments/total comments	15	0.0093023	0.0100165	-0.0315614	0.0501661
Middle	Hateful comments/total comments	16	0.0085418	0.0131751	-0.0452082	0.0622918
Middle	Hateful comments/total comments	17	0.0090802	0.0020572	0.0006874	0.0174730
Middle	Hateful comments/total comments	18	0.0119519	0.0087297	-0.0236622	0.0475661
Middle	Hateful comments/total comments	19	0.0119465	0.0024621	0.0019021	0.0219910
Middle	Hateful comments/total comments	20	0.0116193	0.0071902	-0.0177144	0.0409530
Middle	Hateful comments/total comments	21	0.0116328	0.0095905	-0.0274931	0.0507587
Middle	Hateful comments/total comments	22	0.0126215	0.0172578	-0.0577843	0.0830274
Middle	Hateful comments/total comments	23	0.0035519	0.0093389	-0.0345477	0.0416514
Middle	Hateful comments/total comments	24	0.0062593	0.0114568	-0.0404806	0.0529991
Middle	Hateful comments/total comments	25	0.0114549	0.0073159	-0.0183914	0.0413012
Middle	Hateful comments/total comments	26	0.0085121	0.0127882	-0.0436594	0.0606836

Middle	Hateful comments/total comments	27	-0.0030281	0.0051650	-0.0240994	0.0180432
Middle	Hateful comments/total comments	28	0.0171943	0.0375989	-0.1361966	0.1705851
Middle	Hateful comments/total comments	29	0.0032399	0.0100540	-0.0377772	0.0442570
Middle	Hateful comments/total comments	30	0.0038395	0.0248902	-0.0977042	0.1053832

sample	outcome	event.time	estimate	std.error	conf.low	conf.high
Least	Hateful comments/total comments	-30	-0.0015757	0.0050822	-0.0126216	0.0094703
Least	Hateful comments/total comments	-29	-0.0054893	0.0079024	-0.0226649	0.0116864
Least	Hateful comments/total comments	-28	0.0002693	0.0045985	-0.0097253	0.0102640
Least	Hateful comments/total comments	-27	0.0053093	0.0080392	-0.0121636	0.0227822
Least	Hateful comments/total comments	-26	-0.0020607	0.0064256	-0.0160265	0.0119050
Least	Hateful comments/total comments	-25	0.0037846	0.0050099	-0.0071043	0.0146735
Least	Hateful comments/total comments	-24	-0.0004785	0.0013791	-0.0034760	0.0025191
Least	Hateful comments/total comments	-23	-0.0000495	0.0032764	-0.0071706	0.0070717
Least	Hateful comments/total comments	-22	-0.0084360	0.0050907	-0.0195005	0.0026284
Least	Hateful comments/total comments	-21	0.0042227	0.0041965	-0.0048983	0.0133437
Least	Hateful comments/total comments	-20	0.0046591	0.0040885	-0.0042271	0.0135453
Least	Hateful comments/total comments	-19	0.0055506	0.0024286	0.0002722	0.0108290
Least	Hateful comments/total comments	-18	-0.0071532	0.0031576	-0.0140160	-0.0002903
Least	Hateful comments/total comments	-17	0.0025739	0.0045695	-0.0073577	0.0125054
Least	Hateful comments/total comments	-16	-0.0010922	0.0037677	-0.0092811	0.0070968
Least	Hateful comments/total comments	-15	0.0009705	0.0041529	-0.0080557	0.0099967
Least	Hateful comments/total comments	-14	0.0063024	0.0041088	-0.0026279	0.0152326
Least	Hateful comments/total comments	-13	-0.0118589	0.0081090	-0.0294837	0.0057658
Least	Hateful comments/total comments	-12	0.0064170	0.0085367	-0.0121371	0.0249712
Least	Hateful comments/total comments	-11	-0.0012004	0.0043816	-0.0107236	0.0083227
Least	Hateful comments/total comments	-10	0.0000398	0.0093217	-0.0202206	0.0203002
Least	Hateful comments/total comments	-9	0.0011918	0.0062020	-0.0122881	0.0146717
Least	Hateful comments/total comments	-8	-0.0050156	0.0055041	-0.0169786	0.0069474
Least	Hateful comments/total comments	-7	-0.0003983	0.0027012	-0.0062693	0.0054727
Least	Hateful comments/total comments	-6	0.0002735	0.0033040	-0.0069077	0.0074547
Least	Hateful comments/total comments	-5	0.0045944	0.0050747	-0.0064352	0.0156240
Least	Hateful comments/total comments	-4	-0.0044908	0.0057343	-0.0169542	0.0079726
Least	Hateful comments/total comments	-3	-0.0034797	0.0052853	-0.0149672	0.0080078
Least	Hateful comments/total comments	-2	0.0020507	0.0061462	-0.0113078	0.0154092
Least	Hateful comments/total comments	-1	0.0012094	0.0042008	-0.0079209	0.0103397
Least	Hateful comments/total comments	0	-0.0029898	0.0078322	-0.0200128	0.0140332
Least	Hateful comments/total comments	1	0.0018679	0.0046897	-0.0083249	0.0120608
Least	Hateful comments/total comments	2	0.0032377	0.0051363	-0.0079259	0.0144012
Least	Hateful comments/total comments	3	0.0014477	0.0052429	-0.0099476	0.0128430
Least	Hateful comments/total comments	4	0.0017760	0.0118875	-0.0240610	0.0276130

Least	Hateful comments/total comments	5	0.0035555	0.0037724	-0.0046437	0.0117547
Least	Hateful comments/total comments	6	0.0045334	0.0051946	-0.0067569	0.0158237
Least	Hateful comments/total comments	7	0.0029698	0.0072051	-0.0126903	0.0186299
Least	Hateful comments/total comments	8	0.0032334	0.0053237	-0.0083376	0.0148044
Least	Hateful comments/total comments	9	0.0011119	0.0080284	-0.0163375	0.0185612
Least	Hateful comments/total comments	10	0.0059940	0.0059184	-0.0068694	0.0188574
Least	Hateful comments/total comments	11	0.0059786	0.0065100	-0.0081707	0.0201279
Least	Hateful comments/total comments	12	0.0080996	0.0053540	-0.0035372	0.0197364
Least	Hateful comments/total comments	13	0.0018576	0.0072597	-0.0139213	0.0176364
Least	Hateful comments/total comments	14	0.0106515	0.0269170	-0.0478518	0.0691549
Least	Hateful comments/total comments	15	0.0024729	0.0017818	-0.0013997	0.0063456
Least	Hateful comments/total comments	16	0.0051971	0.0053547	-0.0064410	0.0168353
Least	Hateful comments/total comments	17	0.0034721	0.0062262	-0.0100602	0.0170045
Least	Hateful comments/total comments	18	0.0064717	0.0074267	-0.0096701	0.0226135
Least	Hateful comments/total comments	19	0.0006301	0.0046930	-0.0095700	0.0108303
Least	Hateful comments/total comments	20	0.0051260	0.0071590	-0.0104339	0.0206859
Least	Hateful comments/total comments	21	0.0061996	0.0100659	-0.0156783	0.0280776
Least	Hateful comments/total comments	22	0.0095507	0.0067210	-0.0050572	0.0241587
Least	Hateful comments/total comments	23	0.0054806	0.0127925	-0.0223235	0.0332847
Least	Hateful comments/total comments	24	0.0098629	0.0022417	0.0049906	0.0147352
Least	Hateful comments/total comments	25	0.0030753	0.0085848	-0.0155834	0.0217340
Least	Hateful comments/total comments	26	0.0071113	0.0083588	-0.0110563	0.0252788
Least	Hateful comments/total comments	27	0.0028618	0.0113121	-0.0217246	0.0274482
Least	Hateful comments/total comments	28	0.0066529	0.0314476	-0.0616973	0.0750032
Least	Hateful comments/total comments	29	-0.0028230	0.0628612	-0.1394498	0.1338037
Least	Hateful comments/total comments	30	0.0007135	0.0283565	-0.0609184	0.0623455

Audience outdegree

Average effect by length of exposure (Callaway and Sant'Anna)

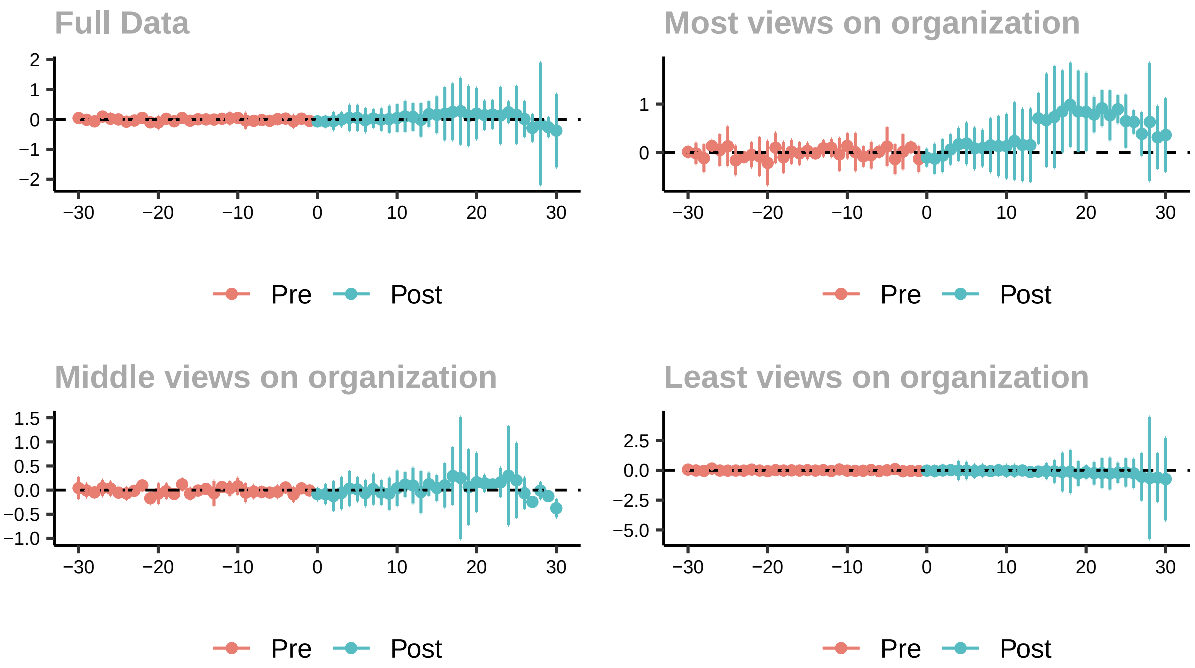

Long timeframe with sampled control groups

sample	outcome	event.time	estimate	std.error	conf.low	conf.high
Full	Audience outdegree	-30	0.0438899	0.0358693	-0.0517469	0.1395267
Full	Audience outdegree	-29	-0.0141968	0.0256821	-0.0826719	0.0542783
Full	Audience outdegree	-28	-0.0659118	0.0485869	-0.1954570	0.0636335
Full	Audience outdegree	-27	0.0941532	0.0328562	0.0065502	0.1817561
Full	Audience outdegree	-26	0.0204704	0.0641604	-0.1505975	0.1915384
Full	Audience outdegree	-25	-0.0013504	0.0573529	-0.1542681	0.1515673
Full	Audience outdegree	-24	-0.0765206	0.0446638	-0.1956058	0.0425645
Full	Audience outdegree	-23	-0.0381033	0.0391900	-0.1425939	0.0663874
Full	Audience outdegree	-22	0.0503281	0.0536819	-0.0928015	0.1934577
Full	Audience outdegree	-21	-0.0989559	0.0396579	-0.2046941	0.0067822
Full	Audience outdegree	-20	-0.1049739	0.0810625	-0.3211072	0.1111595
Full	Audience outdegree	-19	0.0201107	0.0569920	-0.1318447	0.1720661
Full	Audience outdegree	-18	-0.0637051	0.0357388	-0.1589939	0.0315837
Full	Audience outdegree	-17	0.0457460	0.0295783	-0.0331173	0.1246094
Full	Audience outdegree	-16	-0.0445058	0.0526899	-0.1849906	0.0959790
Full	Audience outdegree	-15	0.0018632	0.0260221	-0.0675184	0.0712449
Full	Audience outdegree	-14	-0.0013700	0.0142009	-0.0392332	0.0364932
Full	Audience outdegree	-13	-0.0062287	0.0424505	-0.1194125	0.1069552
Full	Audience outdegree	-12	0.0212628	0.0336919	-0.0685683	0.1110940

Full	Audience outdegree	-11	0.0350824	0.0782648	-0.1735916	0.2437564
Full	Audience outdegree	-10	0.0480232	0.0520078	-0.0906430	0.1866894
Full	Audience outdegree	-9	-0.0419918	0.1000449	-0.3087371	0.2247534
Full	Audience outdegree	-8	-0.0474461	0.0523882	-0.1871265	0.0922344
Full	Audience outdegree	-7	-0.0210695	0.0397289	-0.1269970	0.0848581
Full	Audience outdegree	-6	-0.0479741	0.0456916	-0.1697996	0.0738515
Full	Audience outdegree	-5	0.0089663	0.0621768	-0.1568129	0.1747456
Full	Audience outdegree	-4	0.0259191	0.0392408	-0.0787068	0.1305451
Full	Audience outdegree	-3	-0.0641271	0.0794229	-0.2758889	0.1476346
Full	Audience outdegree	-2	0.0237572	0.0543758	-0.1212227	0.1687371
Full	Audience outdegree	-1	-0.0517425	0.0531708	-0.1935094	0.0900244
Full	Audience outdegree	0	-0.0663858	0.0532836	-0.2084534	0.0756819
Full	Audience outdegree	1	-0.0726803	0.0680215	-0.2540432	0.1086826
Full	Audience outdegree	2	-0.0603097	0.1113394	-0.3571691	0.2365496
Full	Audience outdegree	3	-0.0002476	0.0877233	-0.2341405	0.2336453
Full	Audience outdegree	4	0.0497799	0.1637474	-0.3868126	0.4863724
Full	Audience outdegree	5	0.0514929	0.1608643	-0.3774125	0.4803983
Full	Audience outdegree	6	-0.0265622	0.1513324	-0.4300533	0.3769288
Full	Audience outdegree	7	0.0305780	0.1169492	-0.2812385	0.3423945
Full	Audience outdegree	8	-0.0054540	0.1390255	-0.3761317	0.3652236
Full	Audience outdegree	9	0.0072528	0.1693332	-0.4442329	0.4587385
Full	Audience outdegree	10	0.0430022	0.1728634	-0.4178961	0.5039005
Full	Audience outdegree	11	0.1003363	0.1947955	-0.4190383	0.6197110
Full	Audience outdegree	12	0.0822160	0.1725609	-0.3778757	0.5423078
Full	Audience outdegree	13	-0.0166005	0.2090677	-0.5740285	0.5408276
Full	Audience outdegree	14	0.1788605	0.1633591	-0.2566968	0.6144178
Full	Audience outdegree	15	0.1511442	0.2309699	-0.4646809	0.7669693
Full	Audience outdegree	16	0.1880167	0.3333311	-0.7007295	1.0767630
Full	Audience outdegree	17	0.2500992	0.3565042	-0.7004323	1.2006308
Full	Audience outdegree	18	0.2737884	0.4182286	-0.8413163	1.3888930
Full	Audience outdegree	19	0.1172249	0.3775199	-0.8893399	1.1237897
Full	Audience outdegree	20	0.1949132	0.3228418	-0.6658658	1.0556922
Full	Audience outdegree	21	0.1390172	0.1826872	-0.3480738	0.6261081
Full	Audience outdegree	22	0.1644983	0.1773576	-0.3083825	0.6373790
Full	Audience outdegree	23	0.1296546	0.3577343	-0.8241567	1.0834660
Full	Audience outdegree	24	0.2387979	0.1341090	-0.1187711	0.5963669
Full	Audience outdegree	25	0.1530068	0.3608477	-0.8091057	1.1151193
Full	Audience outdegree	26	0.0118878	0.2250906	-0.5882616	0.6120372
Full	Audience outdegree	27	-0.2848817	0.1703770	-0.7391507	0.1693872
Full	Audience outdegree	28	-0.1491156	0.7678110	-2.1962968	1.8980656
Full	Audience outdegree	29	-0.2619942	0.1231970	-0.5904690	0.0664806

Full	Audience outdegree	30	-0.3765142	0.4603176	-1.6038391	0.8508108

sample	outcome	event.time	estimate	std.error	conf.low	conf.high
Most	Audience outdegree	-30	0.0174907	0.0546461	-0.1037198	0.1387012
Most	Audience outdegree	-29	-0.0148782	0.0995254	-0.2356354	0.2058790
Most	Audience outdegree	-28	-0.1147699	0.1290493	-0.4010141	0.1714742
Most	Audience outdegree	-27	0.1363221	0.0571229	0.0096179	0.2630263
Most	Audience outdegree	-26	0.0538379	0.1464846	-0.2710793	0.3787552
Most	Audience outdegree	-25	0.1316332	0.1830985	-0.2744973	0.5377638
Most	Audience outdegree	-24	-0.1584002	0.1368020	-0.4618405	0.1450402
Most	Audience outdegree	-23	-0.0945687	0.0338001	-0.1695406	-0.0195967
Most	Audience outdegree	-22	-0.0450596	0.1152648	-0.3007284	0.2106092
Most	Audience outdegree	-21	-0.0774013	0.1789313	-0.4742887	0.3194860
Most	Audience outdegree	-20	-0.2114798	0.2049388	-0.6660541	0.2430946
Most	Audience outdegree	-19	0.1007708	0.1398048	-0.2093301	0.4108717
Most	Audience outdegree	-18	-0.0942521	0.1426573	-0.4106800	0.2221758
Most	Audience outdegree	-17	0.0192381	0.1108131	-0.2265564	0.2650325
Most	Audience outdegree	-16	-0.0143244	0.1034094	-0.2436966	0.2150478
Most	Audience outdegree	-15	0.0363165	0.0755547	-0.1312712	0.2039043
Most	Audience outdegree	-14	-0.0154141	0.0419408	-0.1084430	0.0776147
Most	Audience outdegree	-13	0.0905372	0.0752464	-0.0763667	0.2574410
Most	Audience outdegree	-12	0.0987758	0.0842711	-0.0881457	0.2856974
Most	Audience outdegree	-11	-0.0345100	0.1521058	-0.3718957	0.3028757
Most	Audience outdegree	-10	0.1396530	0.1160849	-0.1178349	0.3971408
Most	Audience outdegree	-9	0.0128738	0.1769715	-0.3796664	0.4054141
Most	Audience outdegree	-8	-0.0771166	0.0942776	-0.2862335	0.1320003
Most	Audience outdegree	-7	-0.0507543	0.1233971	-0.3244614	0.2229527
Most	Audience outdegree	-6	0.0202355	0.0555690	-0.1030220	0.1434931
Most	Audience outdegree	-5	0.1251910	0.1798720	-0.2737828	0.5241649
Most	Audience outdegree	-4	-0.1339576	0.1376992	-0.4393881	0.1714728
Most	Audience outdegree	-3	0.0203080	0.1636372	-0.3426555	0.3832715
Most	Audience outdegree	-2	0.1089181	0.0518699	-0.0061345	0.2239708
Most	Audience outdegree	-1	-0.1311130	0.1212596	-0.4000787	0.1378527
Most	Audience outdegree	0	-0.1009127	0.0830013	-0.2850178	0.0831925
Most	Audience outdegree	1	-0.1234186	0.1391163	-0.4319924	0.1851551
Most	Audience outdegree	2	-0.0612361	0.1527531	-0.4000575	0.2775853
Most	Audience outdegree	3	0.0663538	0.1394480	-0.2429556	0.3756632
Most	Audience outdegree	4	0.1729813	0.1530850	-0.1665763	0.5125389
Most	Audience outdegree	5	0.1893905	0.1930813	-0.2388828	0.6176639
Most	Audience outdegree	6	0.0869829	0.1929752	-0.3410552	0.5150210
Most	Audience outdegree	7	0.0938138	0.1689957	-0.2810354	0.4686630

Most	Audience outdegree	8	0.1517515	0.2487084	-0.3999083	0.7034113
Most	Audience outdegree	9	0.1345520	0.2783289	-0.4828090	0.7519130
Most	Audience outdegree	10	0.1328925	0.2984856	-0.5291780	0.7949629
Most	Audience outdegree	11	0.2404556	0.3576198	-0.5527804	1.0336916
Most	Audience outdegree	12	0.1604523	0.3349350	-0.5824667	0.9033712
Most	Audience outdegree	13	0.1546689	0.3388309	-0.5968915	0.9062293
Most	Audience outdegree	14	0.7065818	0.2343291	0.1868167	1.2263470
Most	Audience outdegree	15	0.6692649	0.4314539	-0.2877425	1.6262723
Most	Audience outdegree	16	0.7321760	0.4716442	-0.3139774	1.7783294
Most	Audience outdegree	17	0.8477424	0.3800708	0.0047080	1.6907769
Most	Audience outdegree	18	0.9851677	0.3909239	0.1180599	1.8522755
Most	Audience outdegree	19	0.8475375	0.3790220	0.0068293	1.6882457
Most	Audience outdegree	20	0.8393440	0.3631564	0.0338273	1.6448606
Most	Audience outdegree	21	0.7839165	0.1647834	0.4184107	1.1494223
Most	Audience outdegree	22	0.9144861	0.1673432	0.5433025	1.2856697
Most	Audience outdegree	23	0.7674710	0.2317474	0.2534324	1.2815096
Most	Audience outdegree	24	0.8937456	0.1305828	0.6041001	1.1833911
Most	Audience outdegree	25	0.6496004	0.2462021	0.1034999	1.1957009
Most	Audience outdegree	26	0.6334833	0.1084963	0.3928278	0.8741389
Most	Audience outdegree	27	0.3861669	0.2017884	-0.0614196	0.8337535
Most	Audience outdegree	28	0.6315426	0.5495158	-0.5873376	1.8504229
Most	Audience outdegree	29	0.3153722	0.2917425	-0.3317415	0.9624860
Most	Audience outdegree	30	0.3636353	0.3391053	-0.3885338	1.1158045

sample	outcome	event.time	estimate	std.error	conf.low	conf.high
Middle	Audience outdegree	-30	0.0413650	0.0921108	-0.1812854	0.2640153
Middle	Audience outdegree	-29	-0.0080603	0.0583102	-0.1490078	0.1328873
Middle	Audience outdegree	-28	-0.0481293	0.0404427	-0.1458874	0.0496288
Middle	Audience outdegree	-27	0.0429632	0.0695962	-0.1252648	0.2111911
Middle	Audience outdegree	-26	0.0309916	0.0600097	-0.1140639	0.1760472
Middle	Audience outdegree	-25	-0.0527700	0.0277006	-0.1197279	0.0141880
Middle	Audience outdegree	-24	-0.0694017	0.0542229	-0.2004695	0.0616660
Middle	Audience outdegree	-23	-0.0142454	0.0230238	-0.0698986	0.0414079
Middle	Audience outdegree	-22	0.0964641	0.0418983	-0.0048125	0.1977407
Middle	Audience outdegree	-21	-0.1708694	0.0490128	-0.2893433	-0.0523956
Middle	Audience outdegree	-20	-0.0748897	0.0879276	-0.2874286	0.1376491
Middle	Audience outdegree	-19	-0.0223559	0.0662620	-0.1825245	0.1378127
Middle	Audience outdegree	-18	-0.0810944	0.0159445	-0.1196355	-0.0425533
Middle	Audience outdegree	-17	0.1172559	0.0542183	-0.0138006	0.2483124
Middle	Audience outdegree	-16	-0.0754584	0.0520015	-0.2011566	0.0502398
Middle	Audience outdegree	-15	-0.0098596	0.0236284	-0.0669742	0.0472549

Middle	Audience outdegree	-14	0.0244312	0.0206900	-0.0255806	0.0744431
Middle	Audience outdegree	-13	-0.0649057	0.1066973	-0.3228145	0.1930031
Middle	Audience outdegree	-12	0.0625636	0.0288930	-0.0072765	0.1324038
Middle	Audience outdegree	-11	0.0347941	0.0630372	-0.1175796	0.1871677
Middle	Audience outdegree	-10	0.0785917	0.0736860	-0.0995222	0.2567056
Middle	Audience outdegree	-9	-0.0539890	0.0813472	-0.2506216	0.1426436
Middle	Audience outdegree	-8	-0.0328406	0.0578598	-0.1726995	0.1070182
Middle	Audience outdegree	-7	-0.0384118	0.0438715	-0.1444582	0.0676346
Middle	Audience outdegree	-6	-0.0515810	0.0389131	-0.1456418	0.0424798
Middle	Audience outdegree	-5	-0.0357191	0.0537592	-0.1656658	0.0942277
Middle	Audience outdegree	-4	0.0537450	0.0444606	-0.0537253	0.1612152
Middle	Audience outdegree	-3	-0.0905767	0.0629725	-0.2427939	0.0616406
Middle	Audience outdegree	-2	0.0356377	0.0288829	-0.0341782	0.1054536
Middle	Audience outdegree	-1	-0.0084986	0.0279061	-0.0759533	0.0589561
Middle	Audience outdegree	0	-0.0846806	0.0524963	-0.2115748	0.0422136
Middle	Audience outdegree	1	-0.0848265	0.0841712	-0.2882852	0.1186322
Middle	Audience outdegree	2	-0.1249390	0.1268815	-0.4316373	0.1817592
Middle	Audience outdegree	3	-0.0615961	0.1375258	-0.3940238	0.2708315
Middle	Audience outdegree	4	0.0304082	0.1488251	-0.3293321	0.3901484
Middle	Audience outdegree	5	0.0151216	0.1030178	-0.2338932	0.2641364
Middle	Audience outdegree	6	-0.0642666	0.1124257	-0.3360222	0.2074890
Middle	Audience outdegree	7	0.0205985	0.1325502	-0.2998020	0.3409989
Middle	Audience outdegree	8	-0.0515299	0.1057278	-0.3070953	0.2040355
Middle	Audience outdegree	9	-0.0746816	0.1374568	-0.4069425	0.2575792
Middle	Audience outdegree	10	0.0361883	0.1522132	-0.3317418	0.4041185
Middle	Audience outdegree	11	0.1137518	0.1067375	-0.1442544	0.3717579
Middle	Audience outdegree	12	0.0943344	0.1532265	-0.2760451	0.4647138
Middle	Audience outdegree	13	-0.0422314	0.1812876	-0.4804402	0.3959774
Middle	Audience outdegree	14	0.1212018	0.0998042	-0.1200451	0.3624487
Middle	Audience outdegree	15	0.0411530	0.1118510	-0.2292135	0.3115196
Middle	Audience outdegree	16	0.0990609	0.1900891	-0.3604229	0.5585448
Middle	Audience outdegree	17	0.2919024	0.2468682	-0.3048279	0.8886326
Middle	Audience outdegree	18	0.2497893	0.5254880	-1.0204214	1.5200000
Middle	Audience outdegree	19	0.0612624	0.3236535	-0.7210735	0.8435983
Middle	Audience outdegree	20	0.1623591	0.2526417	-0.4483268	0.7730451
Middle	Audience outdegree	21	0.1407442	0.0719998	-0.0332939	0.3147822
Middle	Audience outdegree	22	0.1169620	0.0309872	0.0420597	0.1918643
Middle	Audience outdegree	23	0.1610047	0.1248063	-0.1406773	0.4626868
Middle	Audience outdegree	24	0.2973103	0.4246143	-0.7290682	1.3236889
Middle	Audience outdegree	25	0.2029908	0.3212082	-0.5734342	0.9794158
Middle	Audience outdegree	26	-0.0638509	0.1330005	-0.3853400	0.2576383

Middle	Audience outdegree	27	-0.2454004	0.0250501	-0.3059515	-0.1848493
Middle	Audience outdegree	28	-0.0109900	0.0722483	-0.1856288	0.1636488
Middle	Audience outdegree	29	-0.1256974	0.0390539	-0.2200986	-0.0312962
Middle	Audience outdegree	30	-0.3767777	0.0780493	-0.5654387	-0.1881168

sample	outcome	event.time	estimate	std.error	conf.low	conf.high
Least	Audience outdegree	-30	0.0536745	0.0487617	-0.0735692	0.1809182
Least	Audience outdegree	-29	-0.0193114	0.1315242	-0.3625240	0.3239013
Least	Audience outdegree	-28	-0.0543199	0.0238856	-0.1166495	0.0080097
Least	Audience outdegree	-27	0.1416154	0.0350426	0.0501716	0.2330592
Least	Audience outdegree	-26	-0.0234376	0.0334722	-0.1107834	0.0639083
Least	Audience outdegree	-25	-0.0332190	0.0433982	-0.1464667	0.0800286
Least	Audience outdegree	-24	-0.0232639	0.0686453	-0.2023940	0.1558661
Least	Audience outdegree	-23	-0.0307412	0.0831113	-0.2476203	0.1861378
Least	Audience outdegree	-22	0.0549361	0.0512389	-0.0787718	0.1886441
Least	Audience outdegree	-21	-0.0234749	0.1340213	-0.3732038	0.3262541
Least	Audience outdegree	-20	-0.0766411	0.0579807	-0.2279417	0.0746596
Least	Audience outdegree	-19	0.0078937	0.0883331	-0.2226116	0.2383990
Least	Audience outdegree	-18	-0.0234383	0.0655769	-0.1945613	0.1476847
Least	Audience outdegree	-17	-0.0098740	0.0320944	-0.0936243	0.0738763
Least	Audience outdegree	-16	-0.0248301	0.0824062	-0.2398692	0.1902091
Least	Audience outdegree	-15	0.0043079	0.0221455	-0.0534807	0.0620965
Least	Audience outdegree	-14	-0.0267858	0.0316006	-0.1092477	0.0556762
Least	Audience outdegree	-13	0.0099015	0.0448408	-0.1071106	0.1269136
Least	Audience outdegree	-12	-0.0603271	0.1251857	-0.3869994	0.2663452
Least	Audience outdegree	-11	0.0651160	0.0527129	-0.0724385	0.2026704
Least	Audience outdegree	-10	-0.0320829	0.0749191	-0.2275845	0.1634188
Least	Audience outdegree	-9	-0.0486520	0.0852812	-0.2711934	0.1738894
Least	Audience outdegree	-8	-0.0396431	0.0828800	-0.2559187	0.1766324
Least	Audience outdegree	-7	0.0194206	0.0401304	-0.0852998	0.1241411
Least	Audience outdegree	-6	-0.0761384	0.0908069	-0.3130993	0.1608225
Least	Audience outdegree	-5	-0.0041181	0.0517393	-0.1391320	0.1308958
Least	Audience outdegree	-4	0.0932403	0.1250007	-0.2329494	0.4194300
Least	Audience outdegree	-3	-0.0823253	0.0961069	-0.3331164	0.1684658
Least	Audience outdegree	-2	-0.0564133	0.1224644	-0.3759845	0.2631580
Least	Audience outdegree	-1	-0.0653866	0.0542194	-0.2068721	0.0760990
Least	Audience outdegree	0	-0.0305738	0.1027701	-0.2987525	0.2376049
Least	Audience outdegree	1	-0.0563444	0.1791453	-0.5238245	0.4111357
Least	Audience outdegree	2	-0.0147934	0.1743493	-0.4697584	0.4401716
Least	Audience outdegree	3	0.0071963	0.1306148	-0.3336433	0.3480359
Least	Audience outdegree	4	-0.0211948	0.3026202	-0.8108829	0.7684933

Least	Audience outdegree	5	-0.0223939	0.2734696	-0.7360133	0.6912256
Least	Audience outdegree	6	-0.0709034	0.2111537	-0.6219094	0.4801026
Least	Audience outdegree	7	-0.0242385	0.1889381	-0.5172728	0.4687959
Least	Audience outdegree	8	-0.0728409	0.1198156	-0.3855000	0.2398182
Least	Audience outdegree	9	-0.0027524	0.1676808	-0.4403158	0.4348111
Least	Audience outdegree	10	-0.0420765	0.1876055	-0.5316335	0.4474805
Least	Audience outdegree	11	-0.0348460	0.1884604	-0.5266338	0.4569417
Least	Audience outdegree	12	-0.0204018	0.1420164	-0.3909940	0.3501904
Least	Audience outdegree	13	-0.1515167	0.0763166	-0.3506651	0.0476317
Least	Audience outdegree	14	-0.1111122	0.1373898	-0.4696312	0.2474067
Least	Audience outdegree	15	-0.0736874	0.2501586	-0.7264768	0.5791021
Least	Audience outdegree	16	-0.0909340	0.3598403	-1.0299380	0.8480701
Least	Audience outdegree	17	-0.1477405	0.6208696	-1.7679012	1.4724201
Least	Audience outdegree	18	-0.1163621	0.6909866	-1.9194932	1.6867690
Least	Audience outdegree	19	-0.2756723	0.3948414	-1.3060118	0.7546671
Least	Audience outdegree	20	-0.1534935	0.2202179	-0.7281527	0.4211657
Least	Audience outdegree	21	-0.2449494	0.3628945	-1.1919235	0.7020247
Least	Audience outdegree	22	-0.2305398	0.4722455	-1.4628654	1.0017859
Least	Audience outdegree	23	-0.2746101	0.5109109	-1.6078331	1.0586129
Least	Audience outdegree	24	-0.2155779	0.3259699	-1.0661971	0.6350414
Least	Audience outdegree	25	-0.1829890	0.4516989	-1.3616983	0.9957203
Least	Audience outdegree	26	-0.2603832	0.4705604	-1.4883116	0.9675452
Least	Audience outdegree	27	-0.5477232	0.7609174	-2.5333387	1.4378924
Least	Audience outdegree	28	-0.6521926	1.9650312	-5.7799460	4.4755607
Least	Audience outdegree	29	-0.6190389	0.7793521	-2.6527599	1.4146820
Least	Audience outdegree	30	-0.7374253	1.3252624	-4.1957004	2.7208498

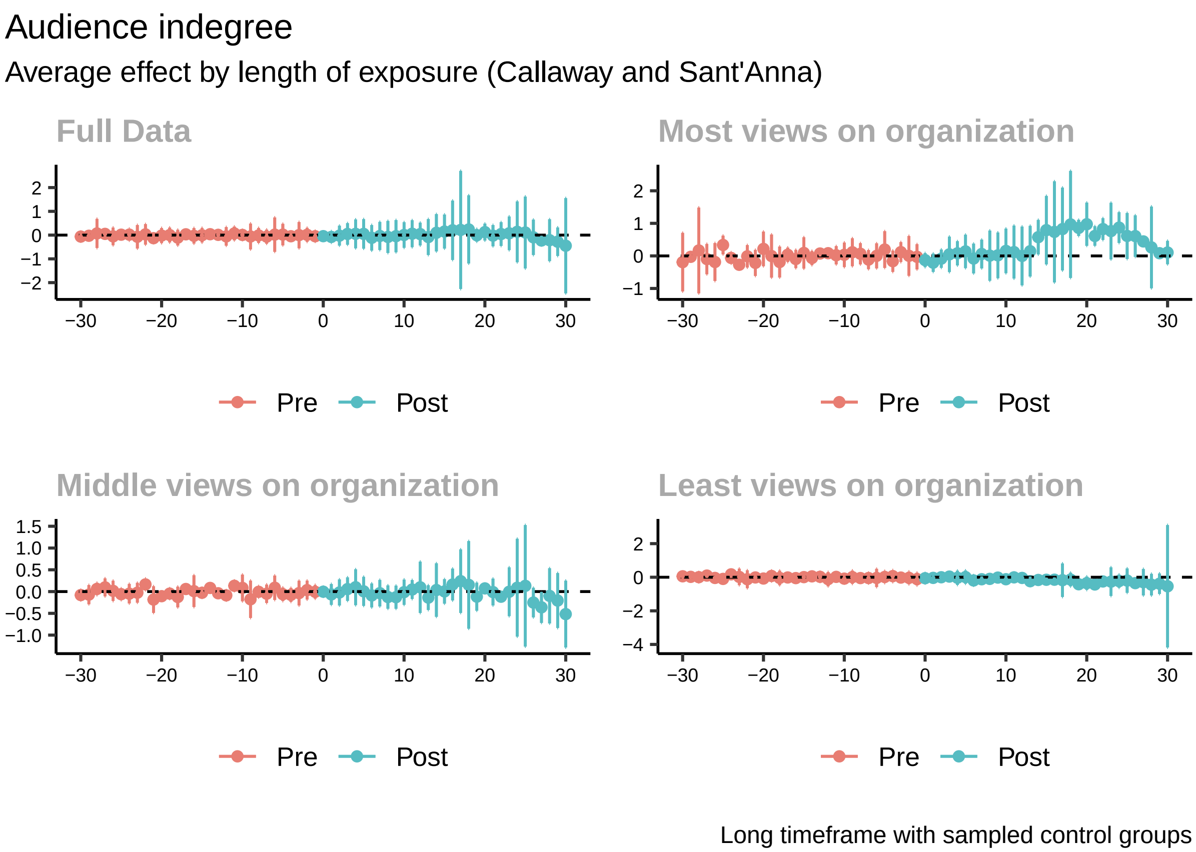

sample	outcome	event.time	estimate	std.error	conf.low	conf.high
Full	Audience indegree	-30	-0.0622278	0.0448286	-0.2669426	0.1424871
Full	Audience indegree	-29	-0.0274293	0.0385288	-0.2033752	0.1485167
Full	Audience indegree	-28	0.0701232	0.1340926	-0.5422257	0.6824721
Full	Audience indegree	-27	0.0508697	0.0352226	-0.1099780	0.2117175
Full	Audience indegree	-26	-0.0532243	0.0813470	-0.4247047	0.3182560
Full	Audience indegree	-25	0.0173417	0.0395845	-0.1634256	0.1981089
Full	Audience indegree	-24	0.0197395	0.0549392	-0.2311467	0.2706257
Full	Audience indegree	-23	-0.0695411	0.1085062	-0.5650467	0.4259645
Full	Audience indegree	-22	0.0308647	0.0943969	-0.4002094	0.4619387
Full	Audience indegree	-21	-0.1294226	0.0444888	-0.3325856	0.0737405
Full	Audience indegree	-20	-0.0190657	0.0696074	-0.3369357	0.2988043
Full	Audience indegree	-19	0.0020387	0.0687930	-0.3121125	0.3161899
Full	Audience indegree	-18	-0.1066368	0.0726226	-0.4382759	0.2250024
Full	Audience indegree	-17	0.0336302	0.0362530	-0.1319233	0.1991837
Full	Audience indegree	-16	-0.0296890	0.0715878	-0.3566028	0.2972248
Full	Audience indegree	-15	-0.0050236	0.0671774	-0.3117970	0.3017497
Full	Audience indegree	-14	0.0377399	0.0171916	-0.0407676	0.1162473
Full	Audience indegree	-13	0.0112261	0.0422424	-0.1816785	0.2041308
Full	Audience indegree	-12	-0.0526948	0.0856392	-0.4437761	0.3383865

Full	Audience indegree	-11	0.0701256	0.0636060	-0.2203382	0.3605894
Full	Audience indegree	-10	0.0079959	0.0467291	-0.2053976	0.2213895
Full	Audience indegree	-9	-0.0572014	0.1192113	-0.6015932	0.4871905
Full	Audience indegree	-8	-0.0134663	0.0688511	-0.3278827	0.3009501
Full	Audience indegree	-7	-0.0640393	0.0651685	-0.3616385	0.2335599
Full	Audience indegree	-6	0.0208338	0.1581460	-0.7013576	0.7430251
Full	Audience indegree	-5	0.0214028	0.0993273	-0.4321864	0.4749919
Full	Audience indegree	-4	-0.0469534	0.0396084	-0.2278297	0.1339229
Full	Audience indegree	-3	-0.0006279	0.1208359	-0.5524387	0.5511828
Full	Audience indegree	-2	0.0120963	0.0643243	-0.2816480	0.3058406
Full	Audience indegree	-1	-0.0472769	0.0502611	-0.2768001	0.1822462
Full	Audience indegree	0	-0.0437712	0.0253896	-0.1597156	0.0721731
Full	Audience indegree	1	-0.0770129	0.0531172	-0.3195785	0.1655526
Full	Audience indegree	2	-0.0232236	0.0916976	-0.4419712	0.3955240
Full	Audience indegree	3	0.0494692	0.0991466	-0.4032950	0.5022334
Full	Audience indegree	4	0.0536112	0.1329129	-0.5533507	0.6605731
Full	Audience indegree	5	0.0507972	0.1356637	-0.5687262	0.6703206
Full	Audience indegree	6	-0.1137061	0.1179539	-0.6523560	0.4249437
Full	Audience indegree	7	-0.0360031	0.1296011	-0.6278414	0.5558351
Full	Audience indegree	8	-0.0772847	0.1477217	-0.7518723	0.5973029
Full	Audience indegree	9	-0.0443424	0.1482925	-0.7215367	0.6328518
Full	Audience indegree	10	0.0047499	0.1174586	-0.5316382	0.5411381
Full	Audience indegree	11	0.0596079	0.1240204	-0.5067450	0.6259609
Full	Audience indegree	12	0.0426347	0.1051083	-0.4373542	0.5226236
Full	Audience indegree	13	-0.0817604	0.1657180	-0.8385303	0.6750096
Full	Audience indegree	14	0.0980124	0.1719591	-0.6872583	0.8832830
Full	Audience indegree	15	0.1505586	0.1569647	-0.5662382	0.8673555
Full	Audience indegree	16	0.2040689	0.2735153	-1.0449696	1.4531073
Full	Audience indegree	17	0.2177983	0.5442800	-2.2677179	2.7033145
Full	Audience indegree	18	0.2386842	0.3141709	-1.1960125	1.6733809
Full	Audience indegree	19	-0.0071712	0.0588241	-0.2757981	0.2614557
Full	Audience indegree	20	0.1263533	0.0780010	-0.2298469	0.4825536
Full	Audience indegree	21	-0.0219131	0.0981015	-0.4699048	0.4260787
Full	Audience indegree	22	0.0445731	0.1093294	-0.4546920	0.5438382
Full	Audience indegree	23	0.0764048	0.1550152	-0.6314897	0.7842994
Full	Audience indegree	24	0.1390048	0.2800718	-1.1399746	1.4179842
Full	Audience indegree	25	0.1094430	0.3313923	-1.4038974	1.6227834
Full	Audience indegree	26	-0.0934665	0.1630050	-0.8378471	0.6509140
Full	Audience indegree	27	-0.2272232	0.0440258	-0.4282719	-0.0261745
Full	Audience indegree	28	-0.2139384	0.1926356	-1.0936308	0.6657540
Full	Audience indegree	29	-0.2771483	0.1312045	-0.8763083	0.3220118

Full	Audience indegree	30	-0.4475367	0.4383839	-2.4494661	1.5543927

sample	outcome	event.time	estimate	std.error	conf.low	conf.high
Most	Audience indegree	-30	-0.1922646	0.3514078	-1.0915499	0.7070207
Most	Audience indegree	-29	-0.0288294	0.0438914	-0.1411517	0.0834929
Most	Audience indegree	-28	0.1673824	0.5143540	-1.1488977	1.4836624
Most	Audience indegree	-27	-0.0976880	0.1819176	-0.5632322	0.3678561
Most	Audience indegree	-26	-0.1876817	0.2274420	-0.7697270	0.3943637
Most	Audience indegree	-25	0.3347890	0.1119138	0.0483913	0.6211868
Most	Audience indegree	-24	-0.0631516	0.0681755	-0.2376191	0.1113158
Most	Audience indegree	-23	-0.2729585	0.0540162	-0.4111911	-0.1347259
Most	Audience indegree	-22	-0.0131073	0.1346812	-0.3577691	0.3315545
Most	Audience indegree	-21	-0.2145582	0.1561597	-0.6141854	0.1850690
Most	Audience indegree	-20	0.2146003	0.2074817	-0.3163649	0.7455655
Most	Audience indegree	-19	-0.0043553	0.2587644	-0.6665575	0.6578469
Most	Audience indegree	-18	-0.1882421	0.1862272	-0.6648149	0.2883306
Most	Audience indegree	-17	0.0341910	0.0870335	-0.1885359	0.2569178
Most	Audience indegree	-16	-0.0949709	0.1096703	-0.3756275	0.1856856
Most	Audience indegree	-15	0.0905028	0.1879472	-0.3904717	0.5714773
Most	Audience indegree	-14	-0.0646046	0.0852439	-0.2827517	0.1535425
Most	Audience indegree	-13	0.0732400	0.0361610	-0.0192993	0.1657793
Most	Audience indegree	-12	0.0832766	0.0365952	-0.0103739	0.1769271
Most	Audience indegree	-11	0.0194496	0.1092661	-0.2601726	0.2990719
Most	Audience indegree	-10	0.0338048	0.1474840	-0.3436205	0.4112301
Most	Audience indegree	-9	0.1127572	0.1672146	-0.3151606	0.5406750
Most	Audience indegree	-8	0.0648583	0.1267442	-0.2594920	0.3892086
Most	Audience indegree	-7	-0.1135799	0.1146850	-0.4070696	0.1799098
Most	Audience indegree	-6	0.0011665	0.1500934	-0.3829365	0.3852694
Most	Audience indegree	-5	0.1948498	0.2199037	-0.3679044	0.7576041
Most	Audience indegree	-4	-0.1624838	0.1278861	-0.4897562	0.1647887
Most	Audience indegree	-3	0.1161736	0.1191858	-0.1888340	0.4211812
Most	Audience indegree	-2	0.0015454	0.2384929	-0.6087801	0.6118710
Most	Audience indegree	-1	-0.0285324	0.1512706	-0.4156480	0.3585833
Most	Audience indegree	0	-0.1290461	0.0839624	-0.3439137	0.0858216
Most	Audience indegree	1	-0.2087676	0.1097943	-0.4897414	0.0722063
Most	Audience indegree	2	-0.0838295	0.1098522	-0.3649516	0.1972926
Most	Audience indegree	3	0.0473639	0.2124086	-0.4962095	0.5909373
Most	Audience indegree	4	0.0785742	0.1451899	-0.2929804	0.4501288
Most	Audience indegree	5	0.1375647	0.2003568	-0.3751670	0.6502963
Most	Audience indegree	6	-0.0809295	0.1786690	-0.5381601	0.3763011
Most	Audience indegree	7	0.0585079	0.1722671	-0.3823398	0.4993556

Most	Audience indegree	8	0.0086195	0.3010271	-0.7617369	0.7789759
Most	Audience indegree	9	0.0173932	0.2743116	-0.6845958	0.7193822
Most	Audience indegree	10	0.1535052	0.2670037	-0.5297823	0.8367926
Most	Audience indegree	11	0.1171867	0.3176692	-0.6957584	0.9301318
Most	Audience indegree	12	-0.0005944	0.3525443	-0.9027882	0.9015993
Most	Audience indegree	13	0.1442174	0.3042420	-0.6343663	0.9228011
Most	Audience indegree	14	0.5747427	0.2072650	0.0443322	1.1051533
Most	Audience indegree	15	0.7914774	0.4103961	-0.2587644	1.8417193
Most	Audience indegree	16	0.7371113	0.6067041	-0.8155012	2.2897238
Most	Audience indegree	17	0.8244744	0.4971446	-0.4477651	2.0967139
Most	Audience indegree	18	0.9669461	0.6424056	-0.6770297	2.6109220
Most	Audience indegree	19	0.8596768	0.0971071	0.6111707	1.1081828
Most	Audience indegree	20	0.9733069	0.2588735	0.3108254	1.6357884
Most	Audience indegree	21	0.6116829	0.1169708	0.3123438	0.9110221
Most	Audience indegree	22	0.8231308	0.1304454	0.4893089	1.1569527
Most	Audience indegree	23	0.7619028	0.3395852	-0.1071274	1.6309329
Most	Audience indegree	24	0.8688190	0.1844479	0.3967996	1.3408384
Most	Audience indegree	25	0.6125684	0.2758546	-0.0933692	1.3185061
Most	Audience indegree	26	0.6082773	0.2488329	-0.0285093	1.2450639
Most	Audience indegree	27	0.4445745	0.0269039	0.3757248	0.5134242
Most	Audience indegree	28	0.2615682	0.4901909	-0.9928761	1.5160124
Most	Audience indegree	29	0.0831006	0.0203969	0.0309030	0.1352983
Most	Audience indegree	30	0.1029947	0.1401557	-0.2556768	0.4616663

sample	outcome	event.time	estimate	std.error	conf.low	conf.high
Middle	Audience indegree	-30	-0.0804420	0.0521806	-0.2010634	0.0401794
Middle	Audience indegree	-29	-0.0699507	0.0953235	-0.2903017	0.1504003
Middle	Audience indegree	-28	0.0601313	0.0613241	-0.0816264	0.2018889
Middle	Audience indegree	-27	0.0966162	0.0891269	-0.1094107	0.3026431
Middle	Audience indegree	-26	0.0161135	0.1004615	-0.2161146	0.2483416
Middle	Audience indegree	-25	-0.0601599	0.0582863	-0.1948952	0.0745754
Middle	Audience indegree	-24	-0.0483457	0.0970213	-0.2726215	0.1759301
Middle	Audience indegree	-23	-0.0264259	0.0985060	-0.2541338	0.2012819
Middle	Audience indegree	-22	0.1641069	0.0582665	0.0294174	0.2987964
Middle	Audience indegree	-21	-0.1835991	0.1318661	-0.4884225	0.1212243
Middle	Audience indegree	-20	-0.1057060	0.0390828	-0.1960503	-0.0153618
Middle	Audience indegree	-19	-0.0470837	0.0528339	-0.1692152	0.0750477
Middle	Audience indegree	-18	-0.1249891	0.1027055	-0.3624045	0.1124263
Middle	Audience indegree	-17	0.0590983	0.0222333	0.0077035	0.1104931
Middle	Audience indegree	-16	0.0095773	0.1576467	-0.3548409	0.3739954
Middle	Audience indegree	-15	-0.0275670	0.0489939	-0.1408219	0.0856879

Middle	Audience indegree	-14	0.0834928	0.0441729	-0.0186179	0.1856035
Middle	Audience indegree	-13	-0.0386815	0.0393056	-0.1295408	0.0521779
Middle	Audience indegree	-12	-0.0868527	0.0510700	-0.2049067	0.0312014
Middle	Audience indegree	-11	0.1329235	0.0518565	0.0130513	0.2527957
Middle	Audience indegree	-10	0.0837065	0.1325179	-0.2226236	0.3900366
Middle	Audience indegree	-9	-0.1772149	0.1853400	-0.6056492	0.2512194
Middle	Audience indegree	-8	-0.0053388	0.0563658	-0.1356346	0.1249571
Middle	Audience indegree	-7	-0.0491660	0.0916572	-0.2610419	0.1627099
Middle	Audience indegree	-6	0.0880532	0.1212832	-0.1923066	0.3684129
Middle	Audience indegree	-5	-0.0614439	0.0673027	-0.2170217	0.0941338
Middle	Audience indegree	-4	-0.0760038	0.0692925	-0.2361812	0.0841735
Middle	Audience indegree	-3	-0.0353082	0.1231040	-0.3198770	0.2492606
Middle	Audience indegree	-2	0.0336349	0.0939635	-0.1835724	0.2508422
Middle	Audience indegree	-1	-0.0054328	0.0680686	-0.1627811	0.1519155
Middle	Audience indegree	0	-0.0028958	0.0493154	-0.1168940	0.1111023
Middle	Audience indegree	1	-0.0647272	0.1032556	-0.3034143	0.1739599
Middle	Audience indegree	2	-0.0206024	0.1297962	-0.3206410	0.2794362
Middle	Audience indegree	3	0.0571300	0.1163934	-0.2119265	0.3261864
Middle	Audience indegree	4	0.0995620	0.1780767	-0.3120825	0.5112065
Middle	Audience indegree	5	0.0058430	0.1439687	-0.3269569	0.3386430
Middle	Audience indegree	6	-0.0886746	0.1190313	-0.3638289	0.1864798
Middle	Audience indegree	7	-0.0369256	0.1319815	-0.3420157	0.2681645
Middle	Audience indegree	8	-0.1257176	0.1156966	-0.3931634	0.1417282
Middle	Audience indegree	9	-0.1294191	0.1146093	-0.3943514	0.1355132
Middle	Audience indegree	10	-0.0131539	0.1255150	-0.3032961	0.2769883
Middle	Audience indegree	11	0.0473707	0.0939780	-0.1698702	0.2646116
Middle	Audience indegree	12	0.0981885	0.2546696	-0.4905092	0.6868861
Middle	Audience indegree	13	-0.1392422	0.1229600	-0.4234782	0.1449939
Middle	Audience indegree	14	0.0352896	0.2655153	-0.5784791	0.6490582
Middle	Audience indegree	15	0.0038198	0.1214768	-0.2769875	0.2846270
Middle	Audience indegree	16	0.1610754	0.1582062	-0.2046362	0.5267870
Middle	Audience indegree	17	0.2393371	0.3153449	-0.4896183	0.9682926
Middle	Audience indegree	18	0.1537273	0.4348015	-0.8513655	1.1588201
Middle	Audience indegree	19	-0.1171012	0.1390862	-0.4386148	0.2044124
Middle	Audience indegree	20	0.0731730	0.0455104	-0.0320296	0.1783755
Middle	Audience indegree	21	-0.0111522	0.1335382	-0.3198409	0.2975365
Middle	Audience indegree	22	-0.1180037	0.0345935	-0.1979705	-0.0380368
Middle	Audience indegree	23	-0.0041750	0.2421226	-0.5638687	0.5555187
Middle	Audience indegree	24	0.0888956	0.4858248	-1.0341434	1.2119346
Middle	Audience indegree	25	0.1318281	0.6035177	-1.2632713	1.5269275
Middle	Audience indegree	26	-0.2524191	0.1465758	-0.5912457	0.0864075

Middle	Audience indegree	27	-0.3620935	0.1541097	-0.7183355	-0.0058515
Middle	Audience indegree	28	-0.0988848	0.2742499	-0.7328444	0.5350748
Middle	Audience indegree	29	-0.2060742	0.2727480	-0.8365621	0.4244137
Middle	Audience indegree	30	-0.5175872	0.3317460	-1.2844555	0.2492812

sample	outcome	event.time	estimate	std.error	conf.low	conf.high
Least	Audience indegree	-30	0.0557925	0.0709010	-0.1159134	0.2274983
Least	Audience indegree	-29	0.0220293	0.0650499	-0.1355067	0.1795652
Least	Audience indegree	-28	0.0040830	0.1245273	-0.2974934	0.3056594
Least	Audience indegree	-27	0.0986068	0.0557569	-0.0364237	0.2336373
Least	Audience indegree	-26	-0.0425734	0.0494291	-0.1622794	0.0771325
Least	Audience indegree	-25	-0.0999663	0.0977158	-0.3366114	0.1366788
Least	Audience indegree	-24	0.1719861	0.0887784	-0.0430146	0.3869869
Least	Audience indegree	-23	0.0196952	0.2018683	-0.4691834	0.5085738
Least	Audience indegree	-22	-0.1192758	0.2189744	-0.6495815	0.4110298
Least	Audience indegree	-21	-0.0133140	0.0567172	-0.1506699	0.1240419
Least	Audience indegree	-20	-0.0782403	0.0441779	-0.1852291	0.0287484
Least	Audience indegree	-19	0.0747850	0.1327896	-0.2468009	0.3963710
Least	Audience indegree	-18	-0.0410285	0.1784189	-0.4731179	0.3910610
Least	Audience indegree	-17	-0.0137984	0.0433575	-0.1188003	0.0912034
Least	Audience indegree	-16	-0.0521503	0.0718622	-0.2261840	0.1218834
Least	Audience indegree	-15	0.0071400	0.0700112	-0.1624111	0.1766911
Least	Audience indegree	-14	0.0530181	0.1014483	-0.1926663	0.2987025
Least	Audience indegree	-13	0.0240005	0.1230305	-0.2739511	0.3219522
Least	Audience indegree	-12	-0.1136830	0.1748477	-0.5371239	0.3097578
Least	Audience indegree	-11	0.0201682	0.0714186	-0.1527911	0.1931276
Least	Audience indegree	-10	-0.0843060	0.1143829	-0.3613151	0.1927031
Least	Audience indegree	-9	0.0045108	0.1632170	-0.3907634	0.3997849
Least	Audience indegree	-8	-0.0499038	0.1011778	-0.2949333	0.1951256
Least	Audience indegree	-7	-0.0486914	0.1326067	-0.3698344	0.2724517
Least	Audience indegree	-6	-0.0421466	0.2237318	-0.5839735	0.4996804
Least	Audience indegree	-5	0.0143358	0.1499461	-0.3487991	0.3774707
Least	Audience indegree	-4	0.0652627	0.1464814	-0.2894815	0.4200069
Least	Audience indegree	-3	-0.0200644	0.0714641	-0.1931341	0.1530052
Least	Audience indegree	-2	-0.0383057	0.1561812	-0.4165406	0.3399291
Least	Audience indegree	-1	-0.1102014	0.1571844	-0.4908659	0.2704631
Least	Audience indegree	0	-0.0590556	0.1269532	-0.3665072	0.2483959
Least	Audience indegree	1	-0.0397038	0.0850707	-0.2457254	0.1663179
Least	Audience indegree	2	-0.0010486	0.0671590	-0.1636923	0.1615951
Least	Audience indegree	3	0.0385662	0.0448376	-0.0700202	0.1471525
Least	Audience indegree	4	-0.0329982	0.1740762	-0.4545707	0.3885742

Least	Audience indegree	5	0.0083491	0.1631735	-0.3868195	0.4035177
Least	Audience indegree	6	-0.1911611	0.1279370	-0.5009950	0.1186729
Least	Audience indegree	7	-0.1199670	0.0707606	-0.2913328	0.0513989
Least	Audience indegree	8	-0.0986102	0.1101138	-0.3652805	0.1680601
Least	Audience indegree	9	-0.0223489	0.0989823	-0.2620613	0.2173635
Least	Audience indegree	10	-0.1182643	0.0937189	-0.3452298	0.1087012
Least	Audience indegree	11	-0.0071019	0.1127966	-0.2802694	0.2660656
Least	Audience indegree	12	-0.0424160	0.1087939	-0.3058898	0.2210577
Least	Audience indegree	13	-0.2358012	0.1143333	-0.5126902	0.0410878
Least	Audience indegree	14	-0.1634707	0.0970215	-0.3984344	0.0714930
Least	Audience indegree	15	-0.1512506	0.0535491	-0.2809342	-0.0215670
Least	Audience indegree	16	-0.1481031	0.0577489	-0.2879576	-0.0082486
Least	Audience indegree	17	-0.1756389	0.4111659	-1.1713881	0.8201103
Least	Audience indegree	18	-0.1418882	0.1693102	-0.5519186	0.2681423
Least	Audience indegree	19	-0.4178008	0.0796862	-0.6107824	-0.2248192
Least	Audience indegree	20	-0.3532199	0.1648361	-0.7524151	0.0459753
Least	Audience indegree	21	-0.4316498	0.0372547	-0.5218721	-0.3414274
Least	Audience indegree	22	-0.2517867	0.1088017	-0.5152794	0.0117060
Least	Audience indegree	23	-0.2628111	0.3515316	-1.1141399	0.5885176
Least	Audience indegree	24	-0.2318117	0.1630629	-0.6267126	0.1630892
Least	Audience indegree	25	-0.2001344	0.2992753	-0.9249104	0.5246416
Least	Audience indegree	26	-0.3502086	0.0918812	-0.5727239	-0.1276934
Least	Audience indegree	27	-0.2962073	0.3253641	-1.0841644	0.4917497
Least	Audience indegree	28	-0.4422725	0.2658966	-1.0862129	0.2016679
Least	Audience indegree	29	-0.3847285	0.2518550	-0.9946635	0.2252065
Least	Audience indegree	30	-0.5396208	1.5045343	-4.1832570	3.1040153

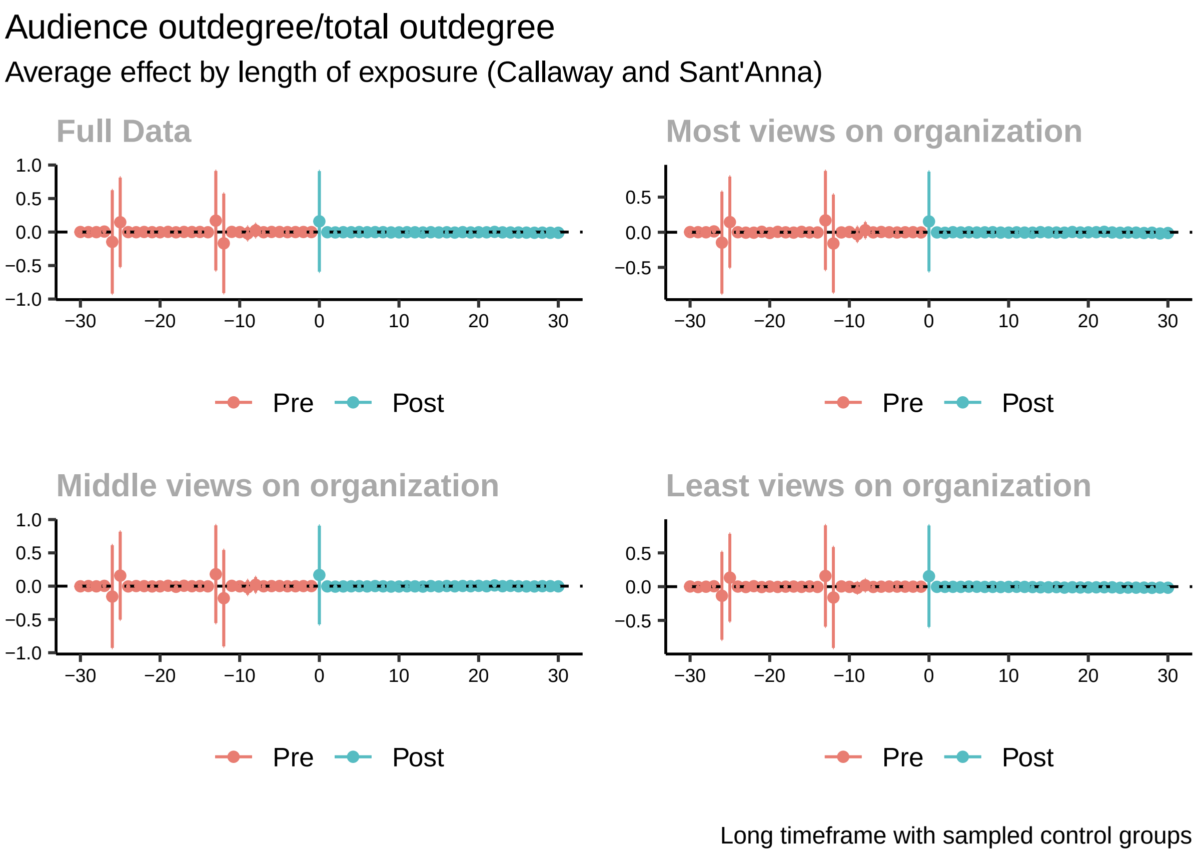

sample	outcome	event.time	estimate	std.error	conf.low	conf.high
Full	Audience outdegree/total outdegree	-30	0.0003838	0.0021176	-0.0045275	0.0052950
Full	Audience outdegree/total outdegree	-29	-0.0013132	0.0032417	-0.0088316	0.0062052
Full	Audience outdegree/total outdegree	-28	-0.0017786	0.0018367	-0.0060384	0.0024811
Full	Audience outdegree/total outdegree	-27	0.0067362	0.0031941	-0.0006718	0.0141443
Full	Audience outdegree/total outdegree	-26	-0.1471103	0.3319086	-0.9169056	0.6226849
Full	Audience outdegree/total outdegree	-25	0.1458985	0.2873663	-0.5205900	0.8123869
Full	Audience outdegree/total outdegree	-24	-0.0009786	0.0030826	-0.0081280	0.0061708
Full	Audience outdegree/total outdegree	-23	-0.0029393	0.0024812	-0.0086939	0.0028154
Full	Audience outdegree/total outdegree	-22	0.0008790	0.0020298	-0.0038288	0.0055868
Full	Audience outdegree/total outdegree	-21	-0.0021262	0.0034277	-0.0100760	0.0058236
Full	Audience outdegree/total outdegree	-20	-0.0036287	0.0048430	-0.0148610	0.0076036
Full	Audience outdegree/total outdegree	-19	0.0024814	0.0014585	-0.0009013	0.0058641
Full	Audience outdegree/total outdegree	-18	-0.0042995	0.0009912	-0.0065985	-0.0020005
Full	Audience outdegree/total outdegree	-17	0.0013790	0.0019300	-0.0030973	0.0058554
Full	Audience outdegree/total outdegree	-16	-0.0000728	0.0021640	-0.0050918	0.0049462
Full	Audience outdegree/total outdegree	-15	0.0006964	0.0014132	-0.0025812	0.0039739
Full	Audience outdegree/total outdegree	-14	-0.0032943	0.0019973	-0.0079266	0.0013380
Full	Audience outdegree/total outdegree	-13	0.1695915	0.3196696	-0.5718180	0.9110009
Full	Audience outdegree/total outdegree	-12	-0.1687070	0.3196066	-0.9099702	0.5725562

Full	Audience outdegree/total outdegree	-11	0.0022697	0.0017547	-0.0017999	0.0063393
Full	Audience outdegree/total outdegree	-10	-0.0013432	0.0037493	-0.0100389	0.0073525
Full	Audience outdegree/total outdegree	-9	-0.0211289	0.0436835	-0.1224440	0.0801863
Full	Audience outdegree/total outdegree	-8	0.0218477	0.0427759	-0.0773624	0.1210579
Full	Audience outdegree/total outdegree	-7	-0.0030881	0.0016715	-0.0069648	0.0007886
Full	Audience outdegree/total outdegree	-6	0.0011012	0.0033054	-0.0065649	0.0087673
Full	Audience outdegree/total outdegree	-5	0.0010849	0.0021430	-0.0038854	0.0060552
Full	Audience outdegree/total outdegree	-4	-0.0015504	0.0014400	-0.0048903	0.0017895
Full	Audience outdegree/total outdegree	-3	-0.0007774	0.0015846	-0.0044526	0.0028979
Full	Audience outdegree/total outdegree	-2	0.0005552	0.0015298	-0.0029928	0.0041032
Full	Audience outdegree/total outdegree	-1	-0.0006343	0.0014974	-0.0041074	0.0028387
Full	Audience outdegree/total outdegree	0	0.1593345	0.3231220	-0.5900820	0.9087510
Full	Audience outdegree/total outdegree	1	-0.0031125	0.0029386	-0.0099280	0.0037030
Full	Audience outdegree/total outdegree	2	-0.0060910	0.0019498	-0.0106132	-0.0015689
Full	Audience outdegree/total outdegree	3	-0.0026385	0.0008972	-0.0047194	-0.0005576
Full	Audience outdegree/total outdegree	4	-0.0030012	0.0007745	-0.0047975	-0.0012049
Full	Audience outdegree/total outdegree	5	-0.0004074	0.0032476	-0.0079395	0.0071246
Full	Audience outdegree/total outdegree	6	-0.0032798	0.0023672	-0.0087700	0.0022104
Full	Audience outdegree/total outdegree	7	-0.0015035	0.0028534	-0.0081214	0.0051143
Full	Audience outdegree/total outdegree	8	-0.0024486	0.0022811	-0.0077391	0.0028419
Full	Audience outdegree/total outdegree	9	-0.0058024	0.0030466	-0.0128684	0.0012636
Full	Audience outdegree/total outdegree	10	-0.0051087	0.0019102	-0.0095389	-0.0006784
Full	Audience outdegree/total outdegree	11	-0.0025480	0.0015127	-0.0060565	0.0009605
Full	Audience outdegree/total outdegree	12	-0.0040675	0.0031240	-0.0113129	0.0031780
Full	Audience outdegree/total outdegree	13	-0.0066351	0.0028005	-0.0131302	-0.0001400
Full	Audience outdegree/total outdegree	14	-0.0027709	0.0015675	-0.0064064	0.0008645
Full	Audience outdegree/total outdegree	15	-0.0061789	0.0057723	-0.0195666	0.0072088
Full	Audience outdegree/total outdegree	16	-0.0034438	0.0101653	-0.0270200	0.0201325
Full	Audience outdegree/total outdegree	17	-0.0082489	0.0111509	-0.0341113	0.0176135
Full	Audience outdegree/total outdegree	18	-0.0018021	0.0152272	-0.0371186	0.0335144
Full	Audience outdegree/total outdegree	19	-0.0061879	0.0070461	-0.0225299	0.0101542
Full	Audience outdegree/total outdegree	20	-0.0033177	0.0058127	-0.0167991	0.0101637
Full	Audience outdegree/total outdegree	21	-0.0044188	0.0018990	-0.0088231	-0.0000145
Full	Audience outdegree/total outdegree	22	0.0021262	0.0077001	-0.0157328	0.0199851
Full	Audience outdegree/total outdegree	23	-0.0051338	0.0116381	-0.0321261	0.0218584
Full	Audience outdegree/total outdegree	24	-0.0066674	0.0064680	-0.0216686	0.0083339
Full	Audience outdegree/total outdegree	25	-0.0069897	0.0118322	-0.0344321	0.0204528
Full	Audience outdegree/total outdegree	26	-0.0087374	0.0047220	-0.0196892	0.0022143
Full	Audience outdegree/total outdegree	27	-0.0104885	0.0008935	-0.0125609	-0.0084161
Full	Audience outdegree/total outdegree	28	-0.0091103	0.0090666	-0.0301384	0.0119178
Full	Audience outdegree/total outdegree	29	-0.0110466	0.0007093	-0.0126917	-0.0094015

Full	Audience outdegree/total outdegree	30	-0.0106543	0.0007939	-0.0124957	-0.0088130

sample	outcome	event.time	estimate	std.error	conf.low	conf.high
Most	Audience outdegree/total outdegree	-30	0.0025590	0.0082190	-0.0166365	0.0217545
Most	Audience outdegree/total outdegree	-29	0.0007498	0.0085137	-0.0191339	0.0206334
Most	Audience outdegree/total outdegree	-28	-0.0007536	0.0047582	-0.0118664	0.0103591
Most	Audience outdegree/total outdegree	-27	0.0119767	0.0044879	0.0014952	0.0224582
Most	Audience outdegree/total outdegree	-26	-0.1478870	0.3096799	-0.8711439	0.5753699
Most	Audience outdegree/total outdegree	-25	0.1440551	0.2774162	-0.5038499	0.7919601
Most	Audience outdegree/total outdegree	-24	0.0006182	0.0084163	-0.0190380	0.0202743
Most	Audience outdegree/total outdegree	-23	-0.0061156	0.0090846	-0.0273327	0.0151015
Most	Audience outdegree/total outdegree	-22	-0.0062575	0.0062459	-0.0208449	0.0083299
Most	Audience outdegree/total outdegree	-21	0.0065023	0.0060926	-0.0077269	0.0207314
Most	Audience outdegree/total outdegree	-20	-0.0102402	0.0130266	-0.0406639	0.0201834
Most	Audience outdegree/total outdegree	-19	0.0059066	0.0075197	-0.0116555	0.0234687
Most	Audience outdegree/total outdegree	-18	-0.0015534	0.0056196	-0.0146780	0.0115712
Most	Audience outdegree/total outdegree	-17	-0.0051435	0.0041872	-0.0149227	0.0046357
Most	Audience outdegree/total outdegree	-16	0.0059113	0.0063163	-0.0088404	0.0206630
Most	Audience outdegree/total outdegree	-15	-0.0022601	0.0045948	-0.0129912	0.0084710
Most	Audience outdegree/total outdegree	-14	-0.0029247	0.0018925	-0.0073447	0.0014952
Most	Audience outdegree/total outdegree	-13	0.1687898	0.3009483	-0.5340744	0.8716540
Most	Audience outdegree/total outdegree	-12	-0.1601663	0.2979475	-0.8560221	0.5356895
Most	Audience outdegree/total outdegree	-11	-0.0026824	0.0086450	-0.0228727	0.0175079
Most	Audience outdegree/total outdegree	-10	0.0034533	0.0076498	-0.0144127	0.0213193
Most	Audience outdegree/total outdegree	-9	-0.0291841	0.0453329	-0.1350590	0.0766908
Most	Audience outdegree/total outdegree	-8	0.0288607	0.0481649	-0.0836282	0.1413497
Most	Audience outdegree/total outdegree	-7	-0.0023450	0.0030162	-0.0093893	0.0046992
Most	Audience outdegree/total outdegree	-6	0.0038990	0.0066049	-0.0115267	0.0193247
Most	Audience outdegree/total outdegree	-5	-0.0004447	0.0051633	-0.0125036	0.0116143
Most	Audience outdegree/total outdegree	-4	-0.0033262	0.0034860	-0.0114678	0.0048154
Most	Audience outdegree/total outdegree	-3	-0.0004626	0.0060020	-0.0144803	0.0135550
Most	Audience outdegree/total outdegree	-2	0.0005710	0.0042400	-0.0093317	0.0104736
Most	Audience outdegree/total outdegree	-1	-0.0024740	0.0038375	-0.0114364	0.0064884
Most	Audience outdegree/total outdegree	0	0.1535207	0.3033706	-0.5550007	0.8620422
Most	Audience outdegree/total outdegree	1	-0.0023983	0.0042390	-0.0122985	0.0075018
Most	Audience outdegree/total outdegree	2	-0.0079860	0.0020390	-0.0127480	-0.0032239
Most	Audience outdegree/total outdegree	3	0.0005692	0.0033415	-0.0072347	0.0083732
Most	Audience outdegree/total outdegree	4	-0.0027731	0.0047039	-0.0137590	0.0082128
Most	Audience outdegree/total outdegree	5	-0.0002737	0.0064988	-0.0154517	0.0149044
Most	Audience outdegree/total outdegree	6	-0.0032057	0.0070041	-0.0195638	0.0131523
Most	Audience outdegree/total outdegree	7	-0.0014980	0.0033453	-0.0093109	0.0063149

Most	Audience outdegree/total outdegree	8	0.0005815	0.0041869	-0.0091969	0.0103600
Most	Audience outdegree/total outdegree	9	-0.0044136	0.0114451	-0.0311435	0.0223164
Most	Audience outdegree/total outdegree	10	-0.0050399	0.0061733	-0.0194576	0.0093778
Most	Audience outdegree/total outdegree	11	-0.0015308	0.0055633	-0.0145239	0.0114623
Most	Audience outdegree/total outdegree	12	-0.0049233	0.0071754	-0.0216816	0.0118349
Most	Audience outdegree/total outdegree	13	-0.0054426	0.0056645	-0.0186721	0.0077869
Most	Audience outdegree/total outdegree	14	0.0014466	0.0049475	-0.0101082	0.0130014
Most	Audience outdegree/total outdegree	15	-0.0018181	0.0081684	-0.0208952	0.0172591
Most	Audience outdegree/total outdegree	16	-0.0038403	0.0220883	-0.0554275	0.0477468
Most	Audience outdegree/total outdegree	17	-0.0058273	0.0248877	-0.0639525	0.0522978
Most	Audience outdegree/total outdegree	18	0.0035007	0.0221708	-0.0482790	0.0552805
Most	Audience outdegree/total outdegree	19	-0.0032928	0.0097493	-0.0260623	0.0194768
Most	Audience outdegree/total outdegree	20	-0.0012484	0.0028760	-0.0079652	0.0054684
Most	Audience outdegree/total outdegree	21	0.0006444	0.0132624	-0.0303300	0.0316188
Most	Audience outdegree/total outdegree	22	0.0063770	0.0084287	-0.0133083	0.0260623
Most	Audience outdegree/total outdegree	23	-0.0027748	0.0165926	-0.0415269	0.0359773
Most	Audience outdegree/total outdegree	24	-0.0071335	0.0172193	-0.0473491	0.0330820
Most	Audience outdegree/total outdegree	25	-0.0034385	0.0082729	-0.0227599	0.0158829
Most	Audience outdegree/total outdegree	26	-0.0051045	0.0064448	-0.0201563	0.0099473
Most	Audience outdegree/total outdegree	27	-0.0110194	0.0127086	-0.0407002	0.0186614
Most	Audience outdegree/total outdegree	28	-0.0063741	0.0173712	-0.0469445	0.0341963
Most	Audience outdegree/total outdegree	29	-0.0175926	0.0088838	-0.0383406	0.0031554
Most	Audience outdegree/total outdegree	30	-0.0109411	0.0120388	-0.0390577	0.0171754

sample	outcome	event.time	estimate	std.error	conf.low	conf.high
Middle	Audience outdegree/total outdegree	-30	-0.0024669	0.0024796	-0.0081727	0.0032390
Middle	Audience outdegree/total outdegree	-29	0.0015260	0.0016536	-0.0022791	0.0053310
Middle	Audience outdegree/total outdegree	-28	-0.0030367	0.0040943	-0.0124582	0.0063847
Middle	Audience outdegree/total outdegree	-27	0.0041904	0.0039562	-0.0049134	0.0132942
Middle	Audience outdegree/total outdegree	-26	-0.1564897	0.3349648	-0.9272850	0.6143056
Middle	Audience outdegree/total outdegree	-25	0.1578039	0.2865441	-0.5015692	0.8171770
Middle	Audience outdegree/total outdegree	-24	-0.0040833	0.0050993	-0.0158173	0.0076508
Middle	Audience outdegree/total outdegree	-23	0.0015864	0.0044656	-0.0086894	0.0118623
Middle	Audience outdegree/total outdegree	-22	0.0003413	0.0033789	-0.0074339	0.0081166
Middle	Audience outdegree/total outdegree	-21	-0.0043900	0.0029804	-0.0112482	0.0024683
Middle	Audience outdegree/total outdegree	-20	-0.0031114	0.0044206	-0.0132838	0.0070610
Middle	Audience outdegree/total outdegree	-19	0.0056737	0.0012958	0.0026920	0.0086555
Middle	Audience outdegree/total outdegree	-18	-0.0097974	0.0026272	-0.0158430	-0.0037518
Middle	Audience outdegree/total outdegree	-17	0.0056496	0.0022921	0.0003752	0.0109240
Middle	Audience outdegree/total outdegree	-16	-0.0013108	0.0036348	-0.0096749	0.0070533
Middle	Audience outdegree/total outdegree	-15	0.0006892	0.0023398	-0.0046949	0.0060734

Middle	Audience outdegree/total outdegree	-14	-0.0028431	0.0020813	-0.0076324	0.0019462
Middle	Audience outdegree/total outdegree	-13	0.1794761	0.3185650	-0.5535811	0.9125334
Middle	Audience outdegree/total outdegree	-12	-0.1809414	0.3143247	-0.9042412	0.5423584
Middle	Audience outdegree/total outdegree	-11	0.0046466	0.0055214	-0.0080588	0.0173520
Middle	Audience outdegree/total outdegree	-10	-0.0030139	0.0058383	-0.0164485	0.0104208
Middle	Audience outdegree/total outdegree	-9	-0.0187737	0.0473024	-0.1276222	0.0900749
Middle	Audience outdegree/total outdegree	-8	0.0197282	0.0501354	-0.0956395	0.1350960
Middle	Audience outdegree/total outdegree	-7	-0.0021412	0.0043604	-0.0121749	0.0078926
Middle	Audience outdegree/total outdegree	-6	0.0005603	0.0040190	-0.0086879	0.0098085
Middle	Audience outdegree/total outdegree	-5	0.0018925	0.0033237	-0.0057558	0.0095408
Middle	Audience outdegree/total outdegree	-4	-0.0018261	0.0023963	-0.0073402	0.0036880
Middle	Audience outdegree/total outdegree	-3	-0.0015774	0.0032968	-0.0091639	0.0060090
Middle	Audience outdegree/total outdegree	-2	0.0011178	0.0030838	-0.0059784	0.0082140
Middle	Audience outdegree/total outdegree	-1	0.0005721	0.0037688	-0.0081004	0.0092446
Middle	Audience outdegree/total outdegree	0	0.1671378	0.3216414	-0.5729987	0.9072743
Middle	Audience outdegree/total outdegree	1	-0.0035689	0.0052789	-0.0157162	0.0085784
Middle	Audience outdegree/total outdegree	2	-0.0073551	0.0049151	-0.0186654	0.0039553
Middle	Audience outdegree/total outdegree	3	-0.0046296	0.0033686	-0.0123812	0.0031220
Middle	Audience outdegree/total outdegree	4	-0.0027014	0.0038445	-0.0115480	0.0061452
Middle	Audience outdegree/total outdegree	5	-0.0010616	0.0015157	-0.0045494	0.0024263
Middle	Audience outdegree/total outdegree	6	-0.0043416	0.0054055	-0.0167803	0.0080971
Middle	Audience outdegree/total outdegree	7	0.0009603	0.0024032	-0.0045697	0.0064902
Middle	Audience outdegree/total outdegree	8	-0.0032443	0.0058492	-0.0167040	0.0102155
Middle	Audience outdegree/total outdegree	9	-0.0065112	0.0039168	-0.0155242	0.0025019
Middle	Audience outdegree/total outdegree	10	-0.0056844	0.0037341	-0.0142771	0.0029083
Middle	Audience outdegree/total outdegree	11	-0.0027144	0.0021630	-0.0076917	0.0022629
Middle	Audience outdegree/total outdegree	12	-0.0034814	0.0050991	-0.0152152	0.0082524
Middle	Audience outdegree/total outdegree	13	-0.0069134	0.0026049	-0.0129075	-0.0009193
Middle	Audience outdegree/total outdegree	14	0.0009918	0.0022605	-0.0042098	0.0061935
Middle	Audience outdegree/total outdegree	15	-0.0051555	0.0024807	-0.0108640	0.0005530
Middle	Audience outdegree/total outdegree	16	0.0013989	0.0076361	-0.0161726	0.0189705
Middle	Audience outdegree/total outdegree	17	-0.0031359	0.0068815	-0.0189711	0.0126994
Middle	Audience outdegree/total outdegree	18	0.0022189	0.0101042	-0.0210320	0.0254699
Middle	Audience outdegree/total outdegree	19	-0.0015078	0.0120266	-0.0291825	0.0261670
Middle	Audience outdegree/total outdegree	20	0.0043541	0.0188856	-0.0391039	0.0478122
Middle	Audience outdegree/total outdegree	21	-0.0016871	0.0061191	-0.0157679	0.0123937
Middle	Audience outdegree/total outdegree	22	0.0104797	0.0101375	-0.0128479	0.0338073
Middle	Audience outdegree/total outdegree	23	-0.0015074	0.0121141	-0.0293834	0.0263686
Middle	Audience outdegree/total outdegree	24	0.0039007	0.0039132	-0.0051040	0.0129054
Middle	Audience outdegree/total outdegree	25	-0.0023433	0.0079907	-0.0207309	0.0160443
Middle	Audience outdegree/total outdegree	26	-0.0042593	0.0072962	-0.0210489	0.0125302

Middle	Audience outdegree/total outdegree	27	-0.0043628	0.0020212	-0.0090139	0.0002883
Middle	Audience outdegree/total outdegree	28	-0.0007359	0.0064706	-0.0156255	0.0141537
Middle	Audience outdegree/total outdegree	29	-0.0000922	0.0012957	-0.0030738	0.0028894
Middle	Audience outdegree/total outdegree	30	-0.0034950	0.0034013	-0.0113219	0.0043318

sample	outcome	event.time	estimate	std.error	conf.low	conf.high
Least	Audience outdegree/total outdegree	-30	0.0020530	0.0036576	-0.0059567	0.0100628
Least	Audience outdegree/total outdegree	-29	-0.0062791	0.0034086	-0.0137436	0.0011853
Least	Audience outdegree/total outdegree	-28	-0.0010444	0.0029620	-0.0075310	0.0054421
Least	Audience outdegree/total outdegree	-27	0.0055595	0.0029514	-0.0009037	0.0120227
Least	Audience outdegree/total outdegree	-26	-0.1354286	0.2962188	-0.7841163	0.5132591
Least	Audience outdegree/total outdegree	-25	0.1333689	0.2964592	-0.5158452	0.7825830
Least	Audience outdegree/total outdegree	-24	0.0013965	0.0029548	-0.0050742	0.0078673
Least	Audience outdegree/total outdegree	-23	-0.0057846	0.0032804	-0.0129684	0.0013992
Least	Audience outdegree/total outdegree	-22	0.0070512	0.0018577	0.0029830	0.0111194
Least	Audience outdegree/total outdegree	-21	-0.0061342	0.0036820	-0.0141974	0.0019290
Least	Audience outdegree/total outdegree	-20	0.0009765	0.0028116	-0.0051805	0.0071336
Least	Audience outdegree/total outdegree	-19	-0.0039795	0.0022029	-0.0088036	0.0008447
Least	Audience outdegree/total outdegree	-18	-0.0000327	0.0024374	-0.0053703	0.0053050
Least	Audience outdegree/total outdegree	-17	0.0012780	0.0040271	-0.0075408	0.0100969
Least	Audience outdegree/total outdegree	-16	-0.0032403	0.0055013	-0.0152876	0.0088070
Least	Audience outdegree/total outdegree	-15	0.0030054	0.0018619	-0.0010720	0.0070829
Least	Audience outdegree/total outdegree	-14	-0.0041390	0.0020174	-0.0085569	0.0002790
Least	Audience outdegree/total outdegree	-13	0.1582270	0.3420605	-0.5908492	0.9073031
Least	Audience outdegree/total outdegree	-12	-0.1608368	0.3406940	-0.9069205	0.5852469
Least	Audience outdegree/total outdegree	-11	0.0033573	0.0017841	-0.0005498	0.0072643
Least	Audience outdegree/total outdegree	-10	-0.0029749	0.0013497	-0.0059307	-0.0000192
Least	Audience outdegree/total outdegree	-9	-0.0176358	0.0375542	-0.0998755	0.0646039
Least	Audience outdegree/total outdegree	-8	0.0186897	0.0407576	-0.0705652	0.1079446
Least	Audience outdegree/total outdegree	-7	-0.0046530	0.0043476	-0.0141737	0.0048677
Least	Audience outdegree/total outdegree	-6	-0.0005833	0.0047486	-0.0109823	0.0098156
Least	Audience outdegree/total outdegree	-5	0.0013205	0.0021485	-0.0033845	0.0060256
Least	Audience outdegree/total outdegree	-4	0.0003080	0.0026068	-0.0054006	0.0060166
Least	Audience outdegree/total outdegree	-3	-0.0001443	0.0026280	-0.0058994	0.0056108
Least	Audience outdegree/total outdegree	-2	-0.0001229	0.0020640	-0.0046428	0.0043969
Least	Audience outdegree/total outdegree	-1	-0.0004221	0.0031773	-0.0073801	0.0065358
Least	Audience outdegree/total outdegree	0	0.1544610	0.3424434	-0.5954536	0.9043756
Least	Audience outdegree/total outdegree	1	-0.0031217	0.0040803	-0.0120571	0.0058138
Least	Audience outdegree/total outdegree	2	-0.0029967	0.0023001	-0.0080336	0.0020403
Least	Audience outdegree/total outdegree	3	-0.0027552	0.0024812	-0.0081887	0.0026783
Least	Audience outdegree/total outdegree	4	-0.0033471	0.0053021	-0.0149583	0.0082640

Least	Audience outdegree/total outdegree	5	0.0005829	0.0032789	-0.0065976	0.0077634
Least	Audience outdegree/total outdegree	6	-0.0018008	0.0044482	-0.0115419	0.0079402
Least	Audience outdegree/total outdegree	7	-0.0041012	0.0041940	-0.0132856	0.0050832
Least	Audience outdegree/total outdegree	8	-0.0036826	0.0024015	-0.0089417	0.0015765
Least	Audience outdegree/total outdegree	9	-0.0055611	0.0019587	-0.0098505	-0.0012717
Least	Audience outdegree/total outdegree	10	-0.0038559	0.0047284	-0.0142105	0.0064987
Least	Audience outdegree/total outdegree	11	-0.0025573	0.0026190	-0.0082928	0.0031781
Least	Audience outdegree/total outdegree	12	-0.0038223	0.0043583	-0.0133666	0.0057220
Least	Audience outdegree/total outdegree	13	-0.0066512	0.0040749	-0.0155749	0.0022724
Least	Audience outdegree/total outdegree	14	-0.0097216	0.0037708	-0.0179793	-0.0014640
Least	Audience outdegree/total outdegree	15	-0.0096953	0.0107987	-0.0333432	0.0139527
Least	Audience outdegree/total outdegree	16	-0.0074973	0.0036070	-0.0153962	0.0004016
Least	Audience outdegree/total outdegree	17	-0.0143603	0.0008312	-0.0161806	-0.0125400
Least	Audience outdegree/total outdegree	18	-0.0089830	0.0058314	-0.0217531	0.0037870
Least	Audience outdegree/total outdegree	19	-0.0125256	0.0064292	-0.0266050	0.0015537
Least	Audience outdegree/total outdegree	20	-0.0117907	0.0011467	-0.0143018	-0.0092796
Least	Audience outdegree/total outdegree	21	-0.0099462	0.0016334	-0.0135232	-0.0063692
Least	Audience outdegree/total outdegree	22	-0.0093064	0.0012356	-0.0120122	-0.0066005
Least	Audience outdegree/total outdegree	23	-0.0095507	0.0041138	-0.0185595	-0.0005419
Least	Audience outdegree/total outdegree	24	-0.0166779	0.0062675	-0.0304031	-0.0029526
Least	Audience outdegree/total outdegree	25	-0.0138849	0.0038800	-0.0223816	-0.0053882
Least	Audience outdegree/total outdegree	26	-0.0152564	0.0131824	-0.0441245	0.0136117
Least	Audience outdegree/total outdegree	27	-0.0142173	0.0006947	-0.0157385	-0.0126960
Least	Audience outdegree/total outdegree	28	-0.0177698	0.0133297	-0.0469605	0.0114210
Least	Audience outdegree/total outdegree	29	-0.0140350	0.0124155	-0.0412236	0.0131536
Least	Audience outdegree/total outdegree	30	-0.0158651	0.0073618	-0.0319867	0.0002566

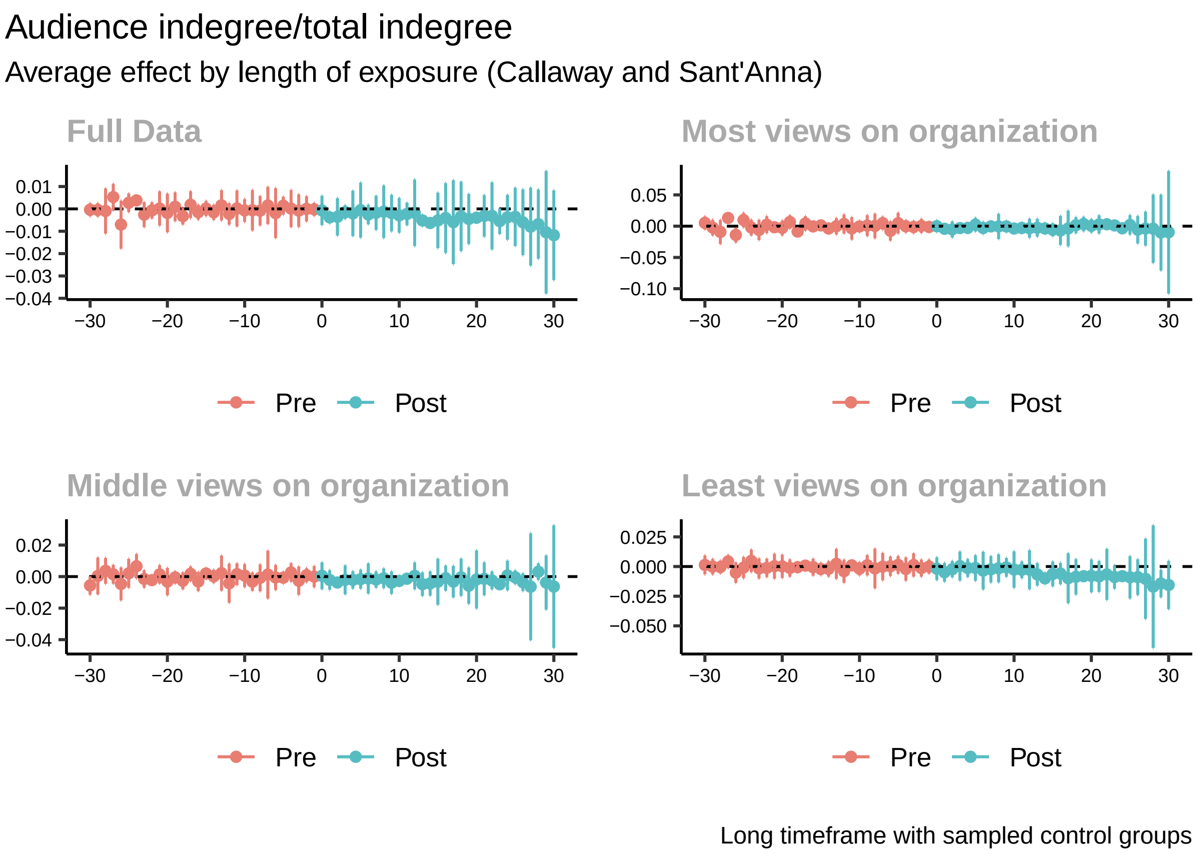

sample	outcome	event.time	estimate	std.error	conf.low	conf.high
Full	Audience indegree/total indegree	-30	-0.0003681	0.0008239	-0.0033028	0.0025666
Full	Audience indegree/total indegree	-29	-0.0004527	0.0008520	-0.0034876	0.0025822
Full	Audience indegree/total indegree	-28	-0.0009543	0.0028259	-0.0110198	0.0091113
Full	Audience indegree/total indegree	-27	0.0052873	0.0016505	-0.0005917	0.0111662
Full	Audience indegree/total indegree	-26	-0.0070249	0.0030036	-0.0177233	0.0036734
Full	Audience indegree/total indegree	-25	0.0027115	0.0011706	-0.0014579	0.0068808
Full	Audience indegree/total indegree	-24	0.0037873	0.0006042	0.0016353	0.0059393
Full	Audience indegree/total indegree	-23	-0.0025761	0.0015564	-0.0081197	0.0029676
Full	Audience indegree/total indegree	-22	-0.0006702	0.0010137	-0.0042811	0.0029406
Full	Audience indegree/total indegree	-21	0.0001796	0.0021464	-0.0074654	0.0078247
Full	Audience indegree/total indegree	-20	-0.0018172	0.0024208	-0.0104397	0.0068053
Full	Audience indegree/total indegree	-19	0.0009468	0.0018036	-0.0054774	0.0073709
Full	Audience indegree/total indegree	-18	-0.0032641	0.0010474	-0.0069949	0.0004667
Full	Audience indegree/total indegree	-17	0.0018602	0.0016815	-0.0041291	0.0078496
Full	Audience indegree/total indegree	-16	-0.0014964	0.0009387	-0.0048400	0.0018472
Full	Audience indegree/total indegree	-15	0.0001338	0.0009525	-0.0032588	0.0035264
Full	Audience indegree/total indegree	-14	-0.0015451	0.0009558	-0.0049495	0.0018593
Full	Audience indegree/total indegree	-13	0.0015767	0.0018924	-0.0051638	0.0083173
Full	Audience indegree/total indegree	-12	-0.0022500	0.0013521	-0.0070661	0.0025662

Full	Audience indegree/total indegree	-11	0.0001911	0.0022523	-0.0078313	0.0082136
Full	Audience indegree/total indegree	-10	-0.0007034	0.0014314	-0.0058019	0.0043952
Full	Audience indegree/total indegree	-9	-0.0006151	0.0025430	-0.0096731	0.0084429
Full	Audience indegree/total indegree	-8	-0.0008189	0.0018404	-0.0073741	0.0057364
Full	Audience indegree/total indegree	-7	0.0015419	0.0023271	-0.0067468	0.0098306
Full	Audience indegree/total indegree	-6	-0.0018988	0.0031160	-0.0129976	0.0092001
Full	Audience indegree/total indegree	-5	0.0014344	0.0011268	-0.0025792	0.0054479
Full	Audience indegree/total indegree	-4	0.0001431	0.0023217	-0.0081265	0.0084126
Full	Audience indegree/total indegree	-3	-0.0007791	0.0020412	-0.0080496	0.0064914
Full	Audience indegree/total indegree	-2	0.0000128	0.0015710	-0.0055828	0.0056084
Full	Audience indegree/total indegree	-1	-0.0002067	0.0008109	-0.0030951	0.0026817
Full	Audience indegree/total indegree	0	-0.0007000	0.0018332	-0.0072295	0.0058296
Full	Audience indegree/total indegree	1	-0.0037806	0.0007491	-0.0064487	-0.0011126
Full	Audience indegree/total indegree	2	-0.0035760	0.0023370	-0.0119001	0.0047481
Full	Audience indegree/total indegree	3	-0.0014606	0.0008625	-0.0045326	0.0016114
Full	Audience indegree/total indegree	4	-0.0020206	0.0028391	-0.0121331	0.0080918
Full	Audience indegree/total indegree	5	-0.0005612	0.0034448	-0.0128311	0.0117087
Full	Audience indegree/total indegree	6	-0.0025138	0.0012230	-0.0068698	0.0018423
Full	Audience indegree/total indegree	7	-0.0017598	0.0021329	-0.0093570	0.0058373
Full	Audience indegree/total indegree	8	-0.0012517	0.0032917	-0.0129763	0.0104729
Full	Audience indegree/total indegree	9	-0.0018655	0.0022875	-0.0100131	0.0062821
Full	Audience indegree/total indegree	10	-0.0029041	0.0021751	-0.0106514	0.0048433
Full	Audience indegree/total indegree	11	-0.0022639	0.0014151	-0.0073042	0.0027764
Full	Audience indegree/total indegree	12	-0.0017343	0.0041655	-0.0165714	0.0131028
Full	Audience indegree/total indegree	13	-0.0051688	0.0007156	-0.0077178	-0.0026199
Full	Audience indegree/total indegree	14	-0.0063035	0.0003004	-0.0073735	-0.0052335
Full	Audience indegree/total indegree	15	-0.0051774	0.0034736	-0.0175499	0.0071950
Full	Audience indegree/total indegree	16	-0.0040882	0.0043761	-0.0196755	0.0114990
Full	Audience indegree/total indegree	17	-0.0059415	0.0052672	-0.0247024	0.0128195
Full	Audience indegree/total indegree	18	-0.0033649	0.0043496	-0.0188576	0.0121278
Full	Audience indegree/total indegree	19	-0.0045048	0.0031350	-0.0156711	0.0066616
Full	Audience indegree/total indegree	20	-0.0039539	0.0006273	-0.0061881	-0.0017197
Full	Audience indegree/total indegree	21	-0.0031319	0.0026089	-0.0124246	0.0061608
Full	Audience indegree/total indegree	22	-0.0031347	0.0042028	-0.0181044	0.0118351
Full	Audience indegree/total indegree	23	-0.0054739	0.0016109	-0.0112119	0.0002641
Full	Audience indegree/total indegree	24	-0.0037512	0.0028106	-0.0137622	0.0062598
Full	Audience indegree/total indegree	25	-0.0035726	0.0036502	-0.0165743	0.0094290
Full	Audience indegree/total indegree	26	-0.0059935	0.0041283	-0.0206981	0.0087111
Full	Audience indegree/total indegree	27	-0.0079268	0.0048892	-0.0253416	0.0094879
Full	Audience indegree/total indegree	28	-0.0068146	0.0043392	-0.0222703	0.0086410
Full	Audience indegree/total indegree	29	-0.0104396	0.0076902	-0.0378311	0.0169518

Full	Audience indegree/total indegree	30	-0.0117755	0.0056167	-0.0317815	0.0082304

sample	outcome	event.time	estimate	std.error	conf.low	conf.high
Most	Audience indegree/total indegree	-30	0.0051805	0.0042408	-0.0052925	0.0156536
Most	Audience indegree/total indegree	-29	-0.0022379	0.0050881	-0.0148032	0.0103274
Most	Audience indegree/total indegree	-28	-0.0092254	0.0077565	-0.0283804	0.0099297
Most	Audience indegree/total indegree	-27	0.0130748	0.0010481	0.0104864	0.0156632
Most	Audience indegree/total indegree	-26	-0.0140279	0.0048561	-0.0260204	-0.0020354
Most	Audience indegree/total indegree	-25	0.0098070	0.0047545	-0.0019345	0.0215485
Most	Audience indegree/total indegree	-24	-0.0021812	0.0051036	-0.0147848	0.0104224
Most	Audience indegree/total indegree	-23	-0.0060283	0.0065405	-0.0221806	0.0101239
Most	Audience indegree/total indegree	-22	0.0023790	0.0055071	-0.0112211	0.0159792
Most	Audience indegree/total indegree	-21	-0.0017367	0.0025159	-0.0079499	0.0044764
Most	Audience indegree/total indegree	-20	-0.0028448	0.0048176	-0.0147421	0.0090525
Most	Audience indegree/total indegree	-19	0.0066849	0.0043506	-0.0040593	0.0174291
Most	Audience indegree/total indegree	-18	-0.0085893	0.0028726	-0.0156833	-0.0014952
Most	Audience indegree/total indegree	-17	0.0043470	0.0047231	-0.0073170	0.0160110
Most	Audience indegree/total indegree	-16	-0.0003747	0.0029448	-0.0076469	0.0068976
Most	Audience indegree/total indegree	-15	0.0012555	0.0025645	-0.0050778	0.0075888
Most	Audience indegree/total indegree	-14	-0.0036994	0.0032366	-0.0116925	0.0042937
Most	Audience indegree/total indegree	-13	-0.0001482	0.0052920	-0.0132170	0.0129206
Most	Audience indegree/total indegree	-12	0.0035067	0.0061359	-0.0116464	0.0186597
Most	Audience indegree/total indegree	-11	-0.0035469	0.0072203	-0.0213777	0.0142840
Most	Audience indegree/total indegree	-10	-0.0004401	0.0036843	-0.0095386	0.0086584
Most	Audience indegree/total indegree	-9	0.0008882	0.0067782	-0.0158510	0.0176273
Most	Audience indegree/total indegree	-8	0.0003479	0.0079524	-0.0192911	0.0199868
Most	Audience indegree/total indegree	-7	0.0048008	0.0042647	-0.0057313	0.0153328
Most	Audience indegree/total indegree	-6	-0.0074352	0.0062927	-0.0229754	0.0081049
Most	Audience indegree/total indegree	-5	0.0047705	0.0067075	-0.0117941	0.0213351
Most	Audience indegree/total indegree	-4	-0.0002223	0.0040640	-0.0102586	0.0098139
Most	Audience indegree/total indegree	-3	-0.0012906	0.0040922	-0.0113964	0.0088152
Most	Audience indegree/total indegree	-2	0.0003816	0.0045362	-0.0108207	0.0115840
Most	Audience indegree/total indegree	-1	-0.0012586	0.0032417	-0.0092641	0.0067469
Most	Audience indegree/total indegree	0	-0.0000640	0.0037920	-0.0094285	0.0093006
Most	Audience indegree/total indegree	1	-0.0042253	0.0017738	-0.0086058	0.0001552
Most	Audience indegree/total indegree	2	-0.0052734	0.0051690	-0.0180385	0.0074918
Most	Audience indegree/total indegree	3	-0.0029969	0.0020155	-0.0079744	0.0019806
Most	Audience indegree/total indegree	4	-0.0028077	0.0032772	-0.0109010	0.0052856
Most	Audience indegree/total indegree	5	0.0023501	0.0044172	-0.0085584	0.0132586
Most	Audience indegree/total indegree	6	-0.0029118	0.0039963	-0.0127808	0.0069572
Most	Audience indegree/total indegree	7	-0.0003672	0.0022627	-0.0059551	0.0052208

Most	Audience indegree/total indegree	8	-0.0004515	0.0081547	-0.0205900	0.0196870
Most	Audience indegree/total indegree	9	-0.0004572	0.0037491	-0.0097158	0.0088014
Most	Audience indegree/total indegree	10	-0.0038198	0.0022786	-0.0094470	0.0018073
Most	Audience indegree/total indegree	11	-0.0028434	0.0034256	-0.0113032	0.0056164
Most	Audience indegree/total indegree	12	-0.0033462	0.0061353	-0.0184977	0.0118053
Most	Audience indegree/total indegree	13	-0.0025776	0.0057376	-0.0167469	0.0115917
Most	Audience indegree/total indegree	14	-0.0036552	0.0034818	-0.0122538	0.0049434
Most	Audience indegree/total indegree	15	-0.0057245	0.0042156	-0.0161351	0.0046862
Most	Audience indegree/total indegree	16	-0.0068326	0.0093602	-0.0299483	0.0162830
Most	Audience indegree/total indegree	17	-0.0036753	0.0115302	-0.0321498	0.0247991
Most	Audience indegree/total indegree	18	0.0015310	0.0052021	-0.0113159	0.0143779
Most	Audience indegree/total indegree	19	0.0033818	0.0046615	-0.0081302	0.0148937
Most	Audience indegree/total indegree	20	0.0005325	0.0044111	-0.0103610	0.0114260
Most	Audience indegree/total indegree	21	0.0028008	0.0059336	-0.0118527	0.0174543
Most	Audience indegree/total indegree	22	0.0030292	0.0013520	-0.0003096	0.0063680
Most	Audience indegree/total indegree	23	0.0011998	0.0015345	-0.0025896	0.0049893
Most	Audience indegree/total indegree	24	-0.0033792	0.0022970	-0.0090518	0.0022935
Most	Audience indegree/total indegree	25	0.0020075	0.0063152	-0.0135882	0.0176033
Most	Audience indegree/total indegree	26	-0.0055947	0.0088740	-0.0275095	0.0163201
Most	Audience indegree/total indegree	27	-0.0038365	0.0109725	-0.0309339	0.0232608
Most	Audience indegree/total indegree	28	-0.0040305	0.0221053	-0.0586210	0.0505600
Most	Audience indegree/total indegree	29	-0.0102020	0.0245388	-0.0708021	0.0503981
Most	Audience indegree/total indegree	30	-0.0096955	0.0396672	-0.1076562	0.0882652

sample	outcome	event.time	estimate	std.error	conf.low	conf.high
Middle	Audience indegree/total indegree	-30	-0.0056055	0.0024771	-0.0114395	0.0002286
Middle	Audience indegree/total indegree	-29	0.0003843	0.0049525	-0.0112798	0.0120484
Middle	Audience indegree/total indegree	-28	0.0036686	0.0034657	-0.0044938	0.0118310
Middle	Audience indegree/total indegree	-27	0.0014467	0.0025267	-0.0045042	0.0073977
Middle	Audience indegree/total indegree	-26	-0.0046767	0.0044232	-0.0150941	0.0057407
Middle	Audience indegree/total indegree	-25	0.0020749	0.0038947	-0.0070977	0.0112476
Middle	Audience indegree/total indegree	-24	0.0065657	0.0032630	-0.0011193	0.0142508
Middle	Audience indegree/total indegree	-23	-0.0014865	0.0023435	-0.0070060	0.0040330
Middle	Audience indegree/total indegree	-22	-0.0022287	0.0009840	-0.0045463	0.0000889
Middle	Audience indegree/total indegree	-21	0.0013890	0.0024867	-0.0044677	0.0072457
Middle	Audience indegree/total indegree	-20	-0.0031457	0.0036898	-0.0118359	0.0055444
Middle	Audience indegree/total indegree	-19	-0.0005300	0.0016766	-0.0044786	0.0034187
Middle	Audience indegree/total indegree	-18	-0.0026825	0.0023012	-0.0081023	0.0027373
Middle	Audience indegree/total indegree	-17	0.0017619	0.0019674	-0.0028717	0.0063955
Middle	Audience indegree/total indegree	-16	-0.0031071	0.0025672	-0.0091533	0.0029390
Middle	Audience indegree/total indegree	-15	0.0019388	0.0014684	-0.0015196	0.0053971

Middle	Audience indegree/total indegree	-14	0.0001660	0.0017997	-0.0040727	0.0044047
Middle	Audience indegree/total indegree	-13	0.0021884	0.0047195	-0.0089271	0.0133038
Middle	Audience indegree/total indegree	-12	-0.0041983	0.0052468	-0.0165556	0.0081590
Middle	Audience indegree/total indegree	-11	0.0015333	0.0029357	-0.0053808	0.0084475
Middle	Audience indegree/total indegree	-10	0.0006349	0.0031004	-0.0066671	0.0079369
Middle	Audience indegree/total indegree	-9	-0.0031857	0.0024717	-0.0090070	0.0026355
Middle	Audience indegree/total indegree	-8	-0.0007881	0.0036098	-0.0092900	0.0077138
Middle	Audience indegree/total indegree	-7	0.0012702	0.0064379	-0.0138922	0.0164326
Middle	Audience indegree/total indegree	-6	-0.0005962	0.0033615	-0.0085132	0.0073208
Middle	Audience indegree/total indegree	-5	-0.0006738	0.0016014	-0.0044455	0.0030978
Middle	Audience indegree/total indegree	-4	0.0026332	0.0024856	-0.0032209	0.0084873
Middle	Audience indegree/total indegree	-3	-0.0026087	0.0038080	-0.0115772	0.0063598
Middle	Audience indegree/total indegree	-2	0.0008425	0.0019043	-0.0036427	0.0053276
Middle	Audience indegree/total indegree	-1	-0.0000608	0.0028327	-0.0067323	0.0066107
Middle	Audience indegree/total indegree	0	0.0004867	0.0035996	-0.0079910	0.0089644
Middle	Audience indegree/total indegree	1	-0.0021184	0.0026141	-0.0082751	0.0040382
Middle	Audience indegree/total indegree	2	-0.0037448	0.0010668	-0.0062574	-0.0012322
Middle	Audience indegree/total indegree	3	-0.0020440	0.0038726	-0.0111648	0.0070768
Middle	Audience indegree/total indegree	4	-0.0020800	0.0023434	-0.0075993	0.0034392
Middle	Audience indegree/total indegree	5	-0.0016358	0.0025437	-0.0076267	0.0043551
Middle	Audience indegree/total indegree	6	-0.0011890	0.0040491	-0.0107255	0.0083474
Middle	Audience indegree/total indegree	7	-0.0018513	0.0021902	-0.0070096	0.0033071
Middle	Audience indegree/total indegree	8	-0.0010660	0.0026311	-0.0072627	0.0051307
Middle	Audience indegree/total indegree	9	-0.0038722	0.0030190	-0.0109826	0.0032381
Middle	Audience indegree/total indegree	10	-0.0027591	0.0013358	-0.0059052	0.0003870
Middle	Audience indegree/total indegree	11	-0.0013366	0.0009298	-0.0035266	0.0008533
Middle	Audience indegree/total indegree	12	0.0004862	0.0035959	-0.0079827	0.0089552
Middle	Audience indegree/total indegree	13	-0.0048003	0.0031661	-0.0122570	0.0026564
Middle	Audience indegree/total indegree	14	-0.0043719	0.0032441	-0.0120124	0.0032687
Middle	Audience indegree/total indegree	15	-0.0032662	0.0061928	-0.0178516	0.0113191
Middle	Audience indegree/total indegree	16	-0.0010117	0.0033888	-0.0089930	0.0069697
Middle	Audience indegree/total indegree	17	-0.0031904	0.0042510	-0.0132024	0.0068216
Middle	Audience indegree/total indegree	18	-0.0004564	0.0050098	-0.0122554	0.0113426
Middle	Audience indegree/total indegree	19	-0.0057876	0.0048800	-0.0172809	0.0057057
Middle	Audience indegree/total indegree	20	-0.0018210	0.0078043	-0.0202017	0.0165596
Middle	Audience indegree/total indegree	21	-0.0014057	0.0044204	-0.0118166	0.0090052
Middle	Audience indegree/total indegree	22	-0.0024404	0.0024039	-0.0081020	0.0032213
Middle	Audience indegree/total indegree	23	-0.0048561	0.0001526	-0.0052154	-0.0044967
Middle	Audience indegree/total indegree	24	0.0007041	0.0039829	-0.0086763	0.0100846
Middle	Audience indegree/total indegree	25	-0.0004728	0.0018515	-0.0048334	0.0038878
Middle	Audience indegree/total indegree	26	-0.0035456	0.0023721	-0.0091324	0.0020412

Middle	Audience indegree/total indegree	27	-0.0064319	0.0143750	-0.0402878	0.0274239
Middle	Audience indegree/total indegree	28	0.0031008	0.0022198	-0.0021272	0.0083288
Middle	Audience indegree/total indegree	29	-0.0037803	0.0072771	-0.0209192	0.0133586
Middle	Audience indegree/total indegree	30	-0.0063684	0.0164986	-0.0452258	0.0324889

sample	outcome	event.time	estimate	std.error	conf.low	conf.high
Least	Audience indegree/total indegree	-30	0.0013530	0.0028099	-0.0065401	0.0092462
Least	Audience indegree/total indegree	-29	-0.0001224	0.0023424	-0.0067024	0.0064577
Least	Audience indegree/total indegree	-28	-0.0004218	0.0019351	-0.0058578	0.0050141
Least	Audience indegree/total indegree	-27	0.0042171	0.0020034	-0.0014107	0.0098449
Least	Audience indegree/total indegree	-26	-0.0051541	0.0030454	-0.0137088	0.0034006
Least	Audience indegree/total indegree	-25	-0.0008948	0.0031963	-0.0098735	0.0080840
Least	Audience indegree/total indegree	-24	0.0047563	0.0033686	-0.0047064	0.0142189
Least	Audience indegree/total indegree	-23	-0.0015924	0.0030294	-0.0101022	0.0069174
Least	Audience indegree/total indegree	-22	-0.0011458	0.0027474	-0.0088636	0.0065720
Least	Audience indegree/total indegree	-21	0.0003083	0.0037309	-0.0101721	0.0107888
Least	Audience indegree/total indegree	-20	0.0001455	0.0034876	-0.0096515	0.0099425
Least	Audience indegree/total indegree	-19	-0.0012477	0.0025926	-0.0085305	0.0060350
Least	Audience indegree/total indegree	-18	-0.0005384	0.0015163	-0.0047977	0.0037209
Least	Audience indegree/total indegree	-17	0.0007564	0.0013193	-0.0029497	0.0044624
Least	Audience indegree/total indegree	-16	-0.0005519	0.0025272	-0.0076512	0.0065473
Least	Audience indegree/total indegree	-15	-0.0020814	0.0018709	-0.0073370	0.0031742
Least	Audience indegree/total indegree	-14	-0.0019544	0.0023159	-0.0084600	0.0045512
Least	Audience indegree/total indegree	-13	0.0022849	0.0045028	-0.0103637	0.0149336
Least	Audience indegree/total indegree	-12	-0.0039381	0.0034604	-0.0136587	0.0057824
Least	Audience indegree/total indegree	-11	0.0009825	0.0014029	-0.0029584	0.0049235
Least	Audience indegree/total indegree	-10	-0.0018211	0.0020130	-0.0074759	0.0038336
Least	Audience indegree/total indegree	-9	0.0010492	0.0030375	-0.0074833	0.0095818
Least	Audience indegree/total indegree	-8	-0.0016280	0.0059416	-0.0183185	0.0150626
Least	Audience indegree/total indegree	-7	-0.0001175	0.0040958	-0.0116230	0.0113880
Least	Audience indegree/total indegree	-6	0.0002219	0.0029053	-0.0079394	0.0083832
Least	Audience indegree/total indegree	-5	0.0013895	0.0026583	-0.0060779	0.0088570
Least	Audience indegree/total indegree	-4	-0.0021054	0.0034926	-0.0119165	0.0077056
Least	Audience indegree/total indegree	-3	0.0013265	0.0034813	-0.0084527	0.0111057
Least	Audience indegree/total indegree	-2	-0.0013180	0.0025484	-0.0084767	0.0058408
Least	Audience indegree/total indegree	-1	-0.0000430	0.0021073	-0.0059625	0.0058765
Least	Audience indegree/total indegree	0	-0.0018081	0.0034169	-0.0114064	0.0077901
Least	Audience indegree/total indegree	1	-0.0048018	0.0028202	-0.0127241	0.0031204
Least	Audience indegree/total indegree	2	-0.0022247	0.0024576	-0.0091284	0.0046790
Least	Audience indegree/total indegree	3	0.0003733	0.0043392	-0.0118158	0.0125624
Least	Audience indegree/total indegree	4	-0.0012694	0.0026845	-0.0088105	0.0062716

Least	Audience indegree/total indegree	5	-0.0012201	0.0038269	-0.0119701	0.0095299
Least	Audience indegree/total indegree	6	-0.0034437	0.0056617	-0.0193479	0.0124605
Least	Audience indegree/total indegree	7	-0.0023227	0.0039499	-0.0134182	0.0087728
Least	Audience indegree/total indegree	8	-0.0014983	0.0042130	-0.0133330	0.0103363
Least	Audience indegree/total indegree	9	-0.0007174	0.0028097	-0.0086100	0.0071752
Least	Audience indegree/total indegree	10	-0.0025462	0.0054617	-0.0178886	0.0127962
Least	Audience indegree/total indegree	11	-0.0027713	0.0024956	-0.0097818	0.0042392
Least	Audience indegree/total indegree	12	-0.0027342	0.0058623	-0.0192017	0.0137333
Least	Audience indegree/total indegree	13	-0.0065955	0.0033704	-0.0160633	0.0028723
Least	Audience indegree/total indegree	14	-0.0099603	0.0016742	-0.0146631	-0.0052574
Least	Audience indegree/total indegree	15	-0.0063101	0.0037126	-0.0167390	0.0041188
Least	Audience indegree/total indegree	16	-0.0059011	0.0032139	-0.0149293	0.0031271
Least	Audience indegree/total indegree	17	-0.0098305	0.0074775	-0.0308352	0.0111742
Least	Audience indegree/total indegree	18	-0.0086665	0.0052953	-0.0235414	0.0062084
Least	Audience indegree/total indegree	19	-0.0081566	0.0009378	-0.0107911	-0.0055222
Least	Audience indegree/total indegree	20	-0.0078851	0.0049284	-0.0217293	0.0059591
Least	Audience indegree/total indegree	21	-0.0083383	0.0046221	-0.0213220	0.0046455
Least	Audience indegree/total indegree	22	-0.0066439	0.0076456	-0.0281209	0.0148331
Least	Audience indegree/total indegree	23	-0.0088541	0.0034865	-0.0186479	0.0009397
Least	Audience indegree/total indegree	24	-0.0081338	0.0000792	-0.0083564	-0.0079112
Least	Audience indegree/total indegree	25	-0.0090907	0.0063393	-0.0268982	0.0087168
Least	Audience indegree/total indegree	26	-0.0089321	0.0053606	-0.0239904	0.0061262
Least	Audience indegree/total indegree	27	-0.0103431	0.0120152	-0.0440947	0.0234085
Least	Audience indegree/total indegree	28	-0.0169456	0.0183825	-0.0685833	0.0346922
Least	Audience indegree/total indegree	29	-0.0143203	0.0041293	-0.0259199	-0.0027208
Least	Audience indegree/total indegree	30	-0.0155379	0.0072252	-0.0358339	0.0047582

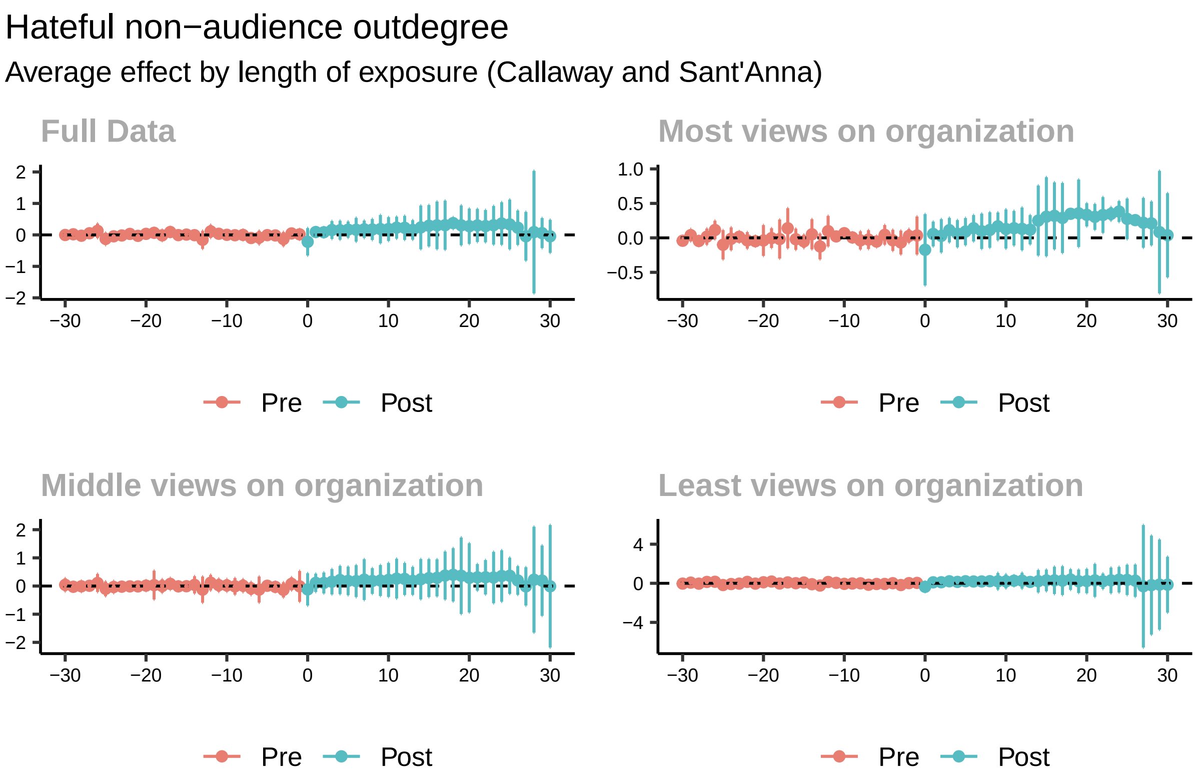

Long timeframe with sampled control groups

sample	outcome	event.time	estimate	std.error	conf.low	conf.high
Full	Hateful non-audience outdegree	-30	-0.0025356	0.0120853	-0.0343268	0.0292556
Full	Hateful non-audience outdegree	-29	0.0190249	0.0238157	-0.0436240	0.0816738
Full	Hateful non-audience outdegree	-28	-0.0268623	0.0215014	-0.0834233	0.0296987
Full	Hateful non-audience outdegree	-27	0.0525694	0.0404958	-0.0539578	0.1590965
Full	Hateful non-audience outdegree	-26	0.1284851	0.0873461	-0.1012849	0.3582550
Full	Hateful non-audience outdegree	-25	-0.1269580	0.0748965	-0.3239784	0.0700624
Full	Hateful non-audience outdegree	-24	-0.0452638	0.0195569	-0.0967096	0.0061821
Full	Hateful non-audience outdegree	-23	-0.0182406	0.0252478	-0.0846566	0.0481755
Full	Hateful non-audience outdegree	-22	0.0311429	0.0270422	-0.0399936	0.1022794
Full	Hateful non-audience outdegree	-21	-0.0300948	0.0159695	-0.0721038	0.0119142
Full	Hateful non-audience outdegree	-20	0.0326665	0.0353569	-0.0603424	0.1256754
Full	Hateful non-audience outdegree	-19	0.0662252	0.0565974	-0.0826583	0.2151087
Full	Hateful non-audience outdegree	-18	-0.0148746	0.0673007	-0.1919137	0.1621644
Full	Hateful non-audience outdegree	-17	0.0923892	0.0592251	-0.0634066	0.2481850
Full	Hateful non-audience outdegree	-16	-0.0067433	0.0511027	-0.1411725	0.1276860
Full	Hateful non-audience outdegree	-15	0.0102366	0.0375540	-0.0885520	0.1090251
Full	Hateful non-audience outdegree	-14	-0.0087841	0.0439073	-0.1242854	0.1067172
Full	Hateful non-audience outdegree	-13	-0.1565588	0.1081887	-0.4411567	0.1280392
Full	Hateful non-audience outdegree	-12	0.1112348	0.0789328	-0.0964033	0.3188730

Full	Hateful non-audience outdegree	-11	0.0358328	0.0363232	-0.0597179	0.1313835
Full	Hateful non-audience outdegree	-10	0.0026193	0.0224605	-0.0564646	0.0617031
Full	Hateful non-audience outdegree	-9	-0.0102480	0.0503041	-0.1425765	0.1220805
Full	Hateful non-audience outdegree	-8	-0.0010834	0.0636062	-0.1684040	0.1662372
Full	Hateful non-audience outdegree	-7	-0.0982595	0.0498876	-0.2294924	0.0329734
Full	Hateful non-audience outdegree	-6	-0.0860158	0.0833049	-0.3051552	0.1331235
Full	Hateful non-audience outdegree	-5	-0.0080983	0.0087402	-0.0310900	0.0148934
Full	Hateful non-audience outdegree	-4	-0.0174380	0.0260386	-0.0859343	0.0510583
Full	Hateful non-audience outdegree	-3	-0.1319034	0.0861524	-0.3585333	0.0947265
Full	Hateful non-audience outdegree	-2	0.0462283	0.0274336	-0.0259378	0.1183944
Full	Hateful non-audience outdegree	-1	0.0174345	0.0627323	-0.1475874	0.1824563
Full	Hateful non-audience outdegree	0	-0.2203527	0.1650969	-0.6546515	0.2139462
Full	Hateful non-audience outdegree	1	0.0911410	0.0568600	-0.0584332	0.2407152
Full	Hateful non-audience outdegree	2	0.0838396	0.0604782	-0.0752525	0.2429318
Full	Hateful non-audience outdegree	3	0.1551098	0.1078954	-0.1287165	0.4389362
Full	Hateful non-audience outdegree	4	0.1482532	0.1142766	-0.1523595	0.4488658
Full	Hateful non-audience outdegree	5	0.1667997	0.0975876	-0.0899114	0.4235107
Full	Hateful non-audience outdegree	6	0.1632808	0.1431651	-0.2133250	0.5398865
Full	Hateful non-audience outdegree	7	0.1772270	0.1048934	-0.0987025	0.4531565
Full	Hateful non-audience outdegree	8	0.1718084	0.1271363	-0.1626325	0.5062493
Full	Hateful non-audience outdegree	9	0.1842139	0.1702295	-0.2635868	0.6320146
Full	Hateful non-audience outdegree	10	0.1855712	0.1427917	-0.1900524	0.5611949
Full	Hateful non-audience outdegree	11	0.2291162	0.1322666	-0.1188202	0.5770527
Full	Hateful non-audience outdegree	12	0.2248257	0.1443825	-0.1549827	0.6046341
Full	Hateful non-audience outdegree	13	0.1573149	0.1172767	-0.1511897	0.4658194
Full	Hateful non-audience outdegree	14	0.2389051	0.2630126	-0.4529679	0.9307781
Full	Hateful non-audience outdegree	15	0.2915456	0.2495471	-0.3649055	0.9479966
Full	Hateful non-audience outdegree	16	0.3042630	0.2875383	-0.4521266	1.0606526
Full	Hateful non-audience outdegree	17	0.3079229	0.2958294	-0.4702770	1.0861228
Full	Hateful non-audience outdegree	18	0.3743777	0.0712893	0.1868462	0.5619093
Full	Hateful non-audience outdegree	19	0.3038739	0.2401002	-0.3277265	0.9354742
Full	Hateful non-audience outdegree	20	0.2757745	0.2122839	-0.2826531	0.8342021
Full	Hateful non-audience outdegree	21	0.3012553	0.1997762	-0.2242700	0.8267805
Full	Hateful non-audience outdegree	22	0.2792576	0.1948376	-0.2332764	0.7917916
Full	Hateful non-audience outdegree	23	0.3062249	0.2333111	-0.3075164	0.9199662
Full	Hateful non-audience outdegree	24	0.3622234	0.2560980	-0.3114602	1.0359071
Full	Hateful non-audience outdegree	25	0.3325520	0.2991789	-0.4544591	1.1195631
Full	Hateful non-audience outdegree	26	0.2286851	0.2071679	-0.3162844	0.7736546
Full	Hateful non-audience outdegree	27	-0.0431845	0.2942588	-0.8172529	0.7308839
Full	Hateful non-audience outdegree	28	0.0890384	0.7395510	-1.8564022	2.0344791
Full	Hateful non-audience outdegree	29	0.0567525	0.1810343	-0.4194708	0.5329758

Full	Hateful non-audience outdegree	30	-0.0442143	0.1985981	-0.5666404	0.4782119

sample	outcome	event.time	estimate	std.error	conf.low	conf.high
Most	Hateful non-audience outdegree	-30	-0.0417056	0.0226159	-0.0971326	0.0137215
Most	Hateful non-audience outdegree	-29	0.0390451	0.0362624	-0.0498269	0.1279172
Most	Hateful non-audience outdegree	-28	-0.0454855	0.0292796	-0.1172441	0.0262732
Most	Hateful non-audience outdegree	-27	0.0178757	0.0478395	-0.0993695	0.1351209
Most	Hateful non-audience outdegree	-26	0.1140230	0.0546078	-0.0198100	0.2478560
Most	Hateful non-audience outdegree	-25	-0.1004727	0.0864321	-0.3123009	0.1113555
Most	Hateful non-audience outdegree	-24	-0.0137461	0.0673469	-0.1788001	0.1513078
Most	Hateful non-audience outdegree	-23	0.0148270	0.0328962	-0.0657950	0.0954491
Most	Hateful non-audience outdegree	-22	-0.0370442	0.0485670	-0.1560725	0.0819841
Most	Hateful non-audience outdegree	-21	-0.0483328	0.0313644	-0.1252008	0.0285353
Most	Hateful non-audience outdegree	-20	-0.0380557	0.0906738	-0.2602794	0.1841681
Most	Hateful non-audience outdegree	-19	-0.0039476	0.0581740	-0.1465208	0.1386256
Most	Hateful non-audience outdegree	-18	-0.0175046	0.1152358	-0.2999250	0.2649159
Most	Hateful non-audience outdegree	-17	0.1391225	0.1180951	-0.1503053	0.4285504
Most	Hateful non-audience outdegree	-16	-0.0200427	0.0621285	-0.1723076	0.1322221
Most	Hateful non-audience outdegree	-15	-0.0508766	0.0397237	-0.1482317	0.0464785
Most	Hateful non-audience outdegree	-14	0.0518450	0.0887379	-0.1656341	0.2693241
Most	Hateful non-audience outdegree	-13	-0.1248612	0.0757433	-0.3104931	0.0607708
Most	Hateful non-audience outdegree	-12	0.0975040	0.0902125	-0.1235891	0.3185972
Most	Hateful non-audience outdegree	-11	0.0211726	0.0185853	-0.0243763	0.0667216
Most	Hateful non-audience outdegree	-10	0.0675271	0.0268938	0.0016157	0.1334385
Most	Hateful non-audience outdegree	-9	0.0060888	0.0298799	-0.0671410	0.0793186
Most	Hateful non-audience outdegree	-8	-0.0363232	0.0534230	-0.1672524	0.0946060
Most	Hateful non-audience outdegree	-7	-0.0253252	0.0554265	-0.1611646	0.1105143
Most	Hateful non-audience outdegree	-6	-0.0536415	0.0343437	-0.1378113	0.0305282
Most	Hateful non-audience outdegree	-5	0.0436556	0.0581368	-0.0988264	0.1861376
Most	Hateful non-audience outdegree	-4	-0.0356462	0.0621897	-0.1880609	0.1167684
Most	Hateful non-audience outdegree	-3	-0.0674955	0.0700606	-0.2392003	0.1042092
Most	Hateful non-audience outdegree	-2	0.0275194	0.0414148	-0.0739801	0.1290190
Most	Hateful non-audience outdegree	-1	0.0343666	0.1121400	-0.2404664	0.3091997
Most	Hateful non-audience outdegree	0	-0.1730112	0.2107698	-0.6895666	0.3435443
Most	Hateful non-audience outdegree	1	0.0558302	0.0729659	-0.1229949	0.2346554
Most	Hateful non-audience outdegree	2	0.0288747	0.0983892	-0.2122580	0.2700074
Most	Hateful non-audience outdegree	3	0.1117462	0.0725742	-0.0661188	0.2896112
Most	Hateful non-audience outdegree	4	0.0603104	0.0800788	-0.1359469	0.2565678
Most	Hateful non-audience outdegree	5	0.0911004	0.0803657	-0.1058601	0.2880608
Most	Hateful non-audience outdegree	6	0.1394383	0.0779844	-0.0516861	0.3305627
Most	Hateful non-audience outdegree	7	0.0956093	0.1040979	-0.1595141	0.3507327

Most	Hateful non-audience outdegree	8	0.1139665	0.1047782	-0.1428244	0.3707573
Most	Hateful non-audience outdegree	9	0.1670719	0.0820640	-0.0340509	0.3681946
Most	Hateful non-audience outdegree	10	0.1284211	0.1157146	-0.1551727	0.4120150
Most	Hateful non-audience outdegree	11	0.1399120	0.1021564	-0.1104534	0.3902773
Most	Hateful non-audience outdegree	12	0.1304338	0.1259368	-0.1782126	0.4390802
Most	Hateful non-audience outdegree	13	0.1178357	0.0863412	-0.0937696	0.3294409
Most	Hateful non-audience outdegree	14	0.2508313	0.2069886	-0.2564571	0.7581198
Most	Hateful non-audience outdegree	15	0.3068650	0.2335176	-0.2654407	0.8791706
Most	Hateful non-audience outdegree	16	0.3196923	0.1976982	-0.1648272	0.8042117
Most	Hateful non-audience outdegree	17	0.2888276	0.2069813	-0.2184429	0.7960980
Most	Hateful non-audience outdegree	18	0.3491799	0.0155794	0.3109978	0.3873620
Most	Hateful non-audience outdegree	19	0.3562098	0.1994440	-0.1325883	0.8450078
Most	Hateful non-audience outdegree	20	0.3342715	0.0683921	0.1666560	0.5018870
Most	Hateful non-audience outdegree	21	0.3046624	0.0764241	0.1173620	0.4919628
Most	Hateful non-audience outdegree	22	0.3316101	0.1053630	0.0733860	0.5898342
Most	Hateful non-audience outdegree	23	0.3477416	0.0399316	0.2498770	0.4456062
Most	Hateful non-audience outdegree	24	0.3821144	0.0613804	0.2316831	0.5325457
Most	Hateful non-audience outdegree	25	0.2755539	0.1201915	-0.0190118	0.5701197
Most	Hateful non-audience outdegree	26	0.2572860	0.0115684	0.2289341	0.2856379
Most	Hateful non-audience outdegree	27	0.2188156	0.1472771	-0.1421315	0.5797628
Most	Hateful non-audience outdegree	28	0.2100965	0.1295246	-0.1073428	0.5275359
Most	Hateful non-audience outdegree	29	0.0836129	0.3623332	-0.8043945	0.9716204
Most	Hateful non-audience outdegree	30	0.0376856	0.2486227	-0.5716399	0.6470110

sample	outcome	event.time	estimate	std.error	conf.low	conf.high
Middle	Hateful non-audience outdegree	-30	0.0422213	0.0503937	-0.1909327	0.2753752
Middle	Hateful non-audience outdegree	-29	-0.0263940	0.0360846	-0.1933444	0.1405565
Middle	Hateful non-audience outdegree	-28	-0.0102339	0.0428061	-0.2082828	0.1878149
Middle	Hateful non-audience outdegree	-27	0.0088414	0.0330597	-0.1441141	0.1617970
Middle	Hateful non-audience outdegree	-26	0.1085593	0.0690653	-0.2109815	0.4281000
Middle	Hateful non-audience outdegree	-25	-0.0983825	0.0565593	-0.3600622	0.1632972
Middle	Hateful non-audience outdegree	-24	-0.0348044	0.0470214	-0.2523559	0.1827470
Middle	Hateful non-audience outdegree	-23	-0.0221608	0.0358817	-0.1881727	0.1438511
Middle	Hateful non-audience outdegree	-22	-0.0116999	0.0186473	-0.0979741	0.0745743
Middle	Hateful non-audience outdegree	-21	-0.0129155	0.0237967	-0.1230145	0.0971835
Middle	Hateful non-audience outdegree	-20	0.0145222	0.0408603	-0.1745239	0.2035684
Middle	Hateful non-audience outdegree	-19	0.0326805	0.1100467	-0.4764666	0.5418275
Middle	Hateful non-audience outdegree	-18	-0.0004542	0.0519439	-0.2407804	0.2398720
Middle	Hateful non-audience outdegree	-17	0.0758968	0.0458421	-0.1361981	0.2879918
Middle	Hateful non-audience outdegree	-16	-0.0113753	0.0370555	-0.1828179	0.1600674
Middle	Hateful non-audience outdegree	-15	-0.0060907	0.0206543	-0.1016509	0.0894695

Middle	Hateful non-audience outdegree	-14	0.0412146	0.0656545	-0.2625454	0.3449747
Middle	Hateful non-audience outdegree	-13	-0.1284184	0.0997189	-0.5897826	0.3329457
Middle	Hateful non-audience outdegree	-12	0.1174549	0.0599300	-0.1598199	0.3947296
Middle	Hateful non-audience outdegree	-11	0.0295259	0.0504054	-0.2036821	0.2627339
Middle	Hateful non-audience outdegree	-10	0.0222968	0.0485485	-0.2023198	0.2469133
Middle	Hateful non-audience outdegree	-9	-0.0119346	0.0632960	-0.3047827	0.2809135
Middle	Hateful non-audience outdegree	-8	0.0128292	0.0497944	-0.2175519	0.2432102
Middle	Hateful non-audience outdegree	-7	-0.0862693	0.0504712	-0.3197819	0.1472433
Middle	Hateful non-audience outdegree	-6	-0.1260136	0.0996707	-0.5871548	0.3351277
Middle	Hateful non-audience outdegree	-5	0.0094614	0.0249137	-0.1058055	0.1247284
Middle	Hateful non-audience outdegree	-4	-0.0300016	0.0391907	-0.2113231	0.1513199
Middle	Hateful non-audience outdegree	-3	-0.1373346	0.0582604	-0.4068847	0.1322155
Middle	Hateful non-audience outdegree	-2	0.0772634	0.0503665	-0.1557644	0.3102912
Middle	Hateful non-audience outdegree	-1	-0.0108975	0.1177251	-0.5555698	0.5337748
Middle	Hateful non-audience outdegree	0	-0.1196964	0.1235820	-0.6914667	0.4520740
Middle	Hateful non-audience outdegree	1	0.1119298	0.0698996	-0.2114709	0.4353305
Middle	Hateful non-audience outdegree	2	0.1094559	0.0801314	-0.2612836	0.4801954
Middle	Hateful non-audience outdegree	3	0.1522364	0.0969141	-0.2961509	0.6006237
Middle	Hateful non-audience outdegree	4	0.2132850	0.1080908	-0.2868129	0.7133830
Middle	Hateful non-audience outdegree	5	0.1814611	0.1090536	-0.3230913	0.6860134
Middle	Hateful non-audience outdegree	6	0.1741327	0.1220036	-0.3903347	0.7386002
Middle	Hateful non-audience outdegree	7	0.2272177	0.1561025	-0.4950135	0.9494489
Middle	Hateful non-audience outdegree	8	0.1780060	0.0980735	-0.2757455	0.6317575
Middle	Hateful non-audience outdegree	9	0.1955872	0.1184442	-0.3524124	0.7435867
Middle	Hateful non-audience outdegree	10	0.2177966	0.1300703	-0.3839926	0.8195858
Middle	Hateful non-audience outdegree	11	0.2612117	0.1521053	-0.4425258	0.9649492
Middle	Hateful non-audience outdegree	12	0.2519019	0.1223590	-0.3142099	0.8180138
Middle	Hateful non-audience outdegree	13	0.1831976	0.1067852	-0.3108598	0.6772550
Middle	Hateful non-audience outdegree	14	0.2418506	0.1539266	-0.4703133	0.9540145
Middle	Hateful non-audience outdegree	15	0.2803982	0.1452519	-0.3916310	0.9524274
Middle	Hateful non-audience outdegree	16	0.2905372	0.1417585	-0.3653290	0.9464035
Middle	Hateful non-audience outdegree	17	0.3712877	0.1838552	-0.4793452	1.2219205
Middle	Hateful non-audience outdegree	18	0.3979701	0.2037982	-0.5449321	1.3408723
Middle	Hateful non-audience outdegree	19	0.3643625	0.2933336	-0.9927884	1.7215133
Middle	Hateful non-audience outdegree	20	0.2919579	0.2648688	-0.9334964	1.5174121
Middle	Hateful non-audience outdegree	21	0.3075964	0.1017996	-0.1633943	0.7785871
Middle	Hateful non-audience outdegree	22	0.3105927	0.1316630	-0.2985656	0.9197510
Middle	Hateful non-audience outdegree	23	0.3016029	0.1968841	-0.6093102	1.2125161
Middle	Hateful non-audience outdegree	24	0.3560087	0.1968309	-0.5546581	1.2666755
Middle	Hateful non-audience outdegree	25	0.3646706	0.1381874	-0.2746736	1.0040149
Middle	Hateful non-audience outdegree	26	0.1970749	0.1094313	-0.3092251	0.7033750

Middle	Hateful non-audience outdegree	27	-0.0134873	0.1467376	-0.6923903	0.6654156
Middle	Hateful non-audience outdegree	28	0.2254217	0.4060629	-1.6532876	2.1041311
Middle	Hateful non-audience outdegree	29	0.1922682	0.2695187	-1.0546994	1.4392358
Middle	Hateful non-audience outdegree	30	-0.0093644	0.4696895	-2.1824516	2.1637227

sample	outcome	event.time	estimate	std.error	conf.low	conf.high
Least	Hateful non-audience outdegree	-30	-0.0330358	0.0615168	-0.1691641	0.1030925
Least	Hateful non-audience outdegree	-29	0.0657930	0.0685085	-0.0858069	0.2173929
Least	Hateful non-audience outdegree	-28	-0.0381813	0.0655847	-0.1833113	0.1069488
Least	Hateful non-audience outdegree	-27	0.1384376	0.0947474	-0.0712254	0.3481005
Least	Hateful non-audience outdegree	-26	0.1551954	0.1716120	-0.2245584	0.5349492
Least	Hateful non-audience outdegree	-25	-0.1779876	0.1383877	-0.4842205	0.1282454
Least	Hateful non-audience outdegree	-24	-0.0779211	0.1644797	-0.4418922	0.2860499
Least	Hateful non-audience outdegree	-23	-0.0372558	0.0420556	-0.1303192	0.0558075
Least	Hateful non-audience outdegree	-22	0.1432941	0.1175270	-0.1167770	0.4033652
Least	Hateful non-audience outdegree	-21	-0.0345412	0.0653966	-0.1792550	0.1101725
Least	Hateful non-audience outdegree	-20	0.1053419	0.0492820	-0.0037124	0.2143962
Least	Hateful non-audience outdegree	-19	0.1785133	0.0988268	-0.0401769	0.3972035
Least	Hateful non-audience outdegree	-18	-0.0229787	0.1105301	-0.2675668	0.2216094
Least	Hateful non-audience outdegree	-17	0.0897866	0.0678671	-0.0603940	0.2399672
Least	Hateful non-audience outdegree	-16	0.0066790	0.0654514	-0.1381560	0.1515140
Least	Hateful non-audience outdegree	-15	0.0763555	0.0812295	-0.1033942	0.2561053
Least	Hateful non-audience outdegree	-14	-0.0983527	0.0307141	-0.1663188	-0.0303866
Least	Hateful non-audience outdegree	-13	-0.2312936	0.1493050	-0.5616851	0.0990979
Least	Hateful non-audience outdegree	-12	0.1317552	0.2045984	-0.3209931	0.5845036
Least	Hateful non-audience outdegree	-11	0.0425993	0.0593971	-0.0888383	0.1740370
Least	Hateful non-audience outdegree	-10	-0.0585137	0.0765981	-0.2280148	0.1109874
Least	Hateful non-audience outdegree	-9	-0.0246959	0.1384039	-0.3309647	0.2815729
Least	Hateful non-audience outdegree	-8	-0.0026815	0.1001635	-0.2243296	0.2189666
Least	Hateful non-audience outdegree	-7	-0.1500119	0.0943674	-0.3588341	0.0588104
Least	Hateful non-audience outdegree	-6	-0.0724054	0.1120938	-0.3204536	0.1756427
Least	Hateful non-audience outdegree	-5	-0.0711291	0.0895734	-0.2693428	0.1270846
Least	Hateful non-audience outdegree	-4	0.0101075	0.0803450	-0.1676851	0.1879000
Least	Hateful non-audience outdegree	-3	-0.1799472	0.1421855	-0.4945843	0.1346899
Least	Hateful non-audience outdegree	-2	0.0148972	0.0916941	-0.1880092	0.2178037
Least	Hateful non-audience outdegree	-1	0.0393893	0.1114156	-0.2071582	0.2859368
Least	Hateful non-audience outdegree	0	-0.3617749	0.2372627	-0.8868049	0.1632550
Least	Hateful non-audience outdegree	1	0.1003425	0.0697662	-0.0540406	0.2547256
Least	Hateful non-audience outdegree	2	0.1050211	0.0785485	-0.0687961	0.2788383
Least	Hateful non-audience outdegree	3	0.2045180	0.1506541	-0.1288590	0.5378949
Least	Hateful non-audience outdegree	4	0.1432382	0.1135664	-0.1080688	0.3945451

Least	Hateful non-audience outdegree	5	0.2084839	0.1163866	-0.0490636	0.4660315
Least	Hateful non-audience outdegree	6	0.1848776	0.1440835	-0.1339595	0.5037148
Least	Hateful non-audience outdegree	7	0.1862668	0.1725595	-0.1955838	0.5681174
Least	Hateful non-audience outdegree	8	0.2147757	0.1253725	-0.0626565	0.4922079
Least	Hateful non-audience outdegree	9	0.2034766	0.3835189	-0.6451982	1.0521514
Least	Hateful non-audience outdegree	10	0.2003189	0.3215475	-0.5112216	0.9118594
Least	Hateful non-audience outdegree	11	0.2629216	0.2479645	-0.2857898	0.8116330
Least	Hateful non-audience outdegree	12	0.2665460	0.3653366	-0.5418938	1.0749859
Least	Hateful non-audience outdegree	13	0.1387656	0.1871899	-0.2754600	0.5529912
Least	Hateful non-audience outdegree	14	0.2137554	0.5160440	-0.9281795	1.3556902
Least	Hateful non-audience outdegree	15	0.2913027	0.4963379	-0.8070251	1.3896306
Least	Hateful non-audience outdegree	16	0.2986126	0.6278673	-1.0907718	1.6879970
Least	Hateful non-audience outdegree	17	0.2698072	0.6627193	-1.1967000	1.7363144
Least	Hateful non-audience outdegree	18	0.3840097	0.4744906	-0.6659731	1.4339926
Least	Hateful non-audience outdegree	19	0.2088558	0.5380792	-0.9818400	1.3995516
Least	Hateful non-audience outdegree	20	0.2315801	0.5597507	-1.0070716	1.4702317
Least	Hateful non-audience outdegree	21	0.2989674	0.7594943	-1.3816896	1.9796245
Least	Hateful non-audience outdegree	22	0.2068198	0.3562151	-0.5814356	0.9950752
Least	Hateful non-audience outdegree	23	0.2956656	0.5873876	-1.0041428	1.5954741
Least	Hateful non-audience outdegree	24	0.3694684	0.5895514	-0.9351282	1.6740651
Least	Hateful non-audience outdegree	25	0.3472106	0.6986645	-1.1988384	1.8932595
Least	Hateful non-audience outdegree	26	0.2660339	0.7432379	-1.3786500	1.9107178
Least	Hateful non-audience outdegree	27	-0.3073884	2.8297095	-6.5691485	5.9543717
Least	Hateful non-audience outdegree	28	-0.1895547	2.2860509	-5.2482727	4.8691632
Least	Hateful non-audience outdegree	29	-0.1348731	2.0786603	-4.7346640	4.4649178
Least	Hateful non-audience outdegree	30	-0.1429421	1.2885888	-2.9944128	2.7085285

Robustness: Dynamic differences-in-differences (Callaway and Sant'Anna 2021) for short time frame with sampled control groups

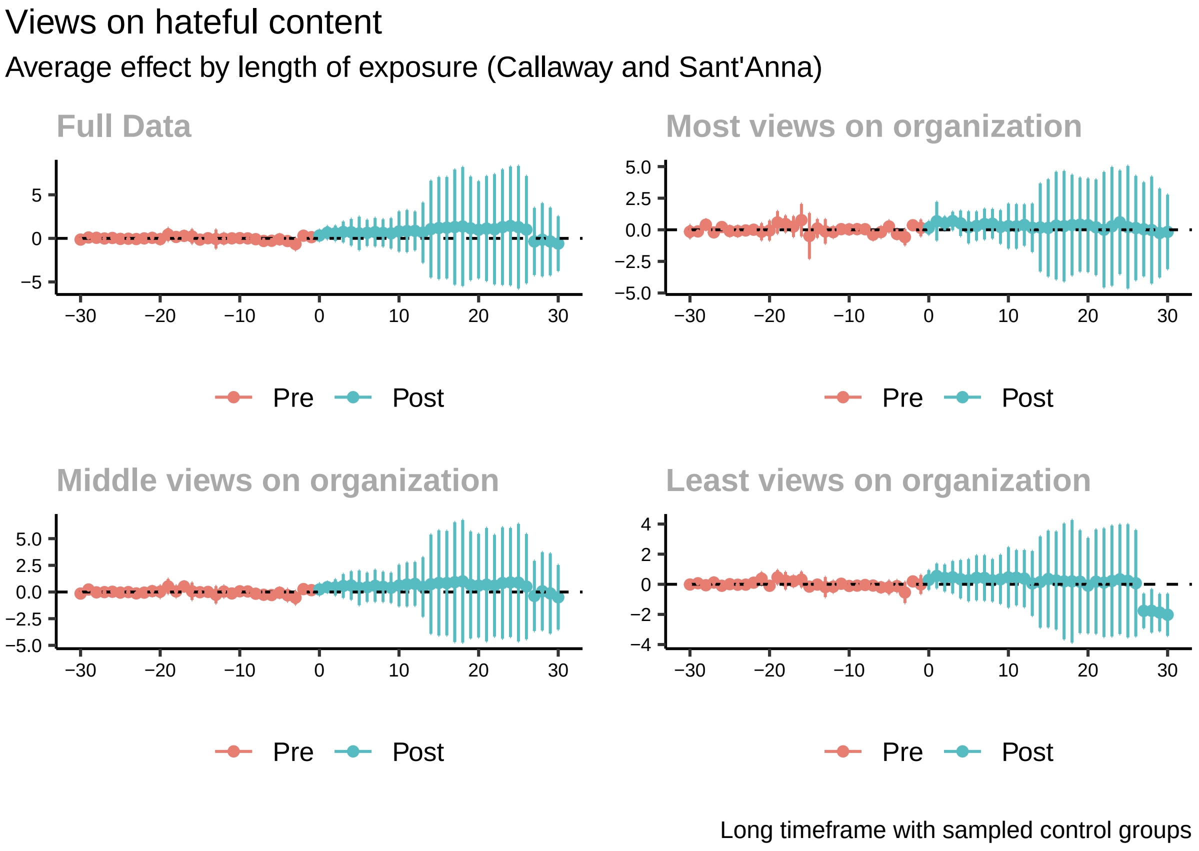

sample	outcome	event.time	estimate	std.error	conf.low	conf.high
Full	Views on hateful content	-30	-0.1415501	0.1199598	-0.4512977	0.1681975
Full	Views on hateful content	-29	0.0965897	0.0424639	-0.0130561	0.2062354
Full	Views on hateful content	-28	0.0638345	0.0701894	-0.1174011	0.2450700
Full	Views on hateful content	-27	-0.0121928	0.0603576	-0.1680418	0.1436561
Full	Views on hateful content	-26	0.0466527	0.0913582	-0.1892429	0.2825482
Full	Views on hateful content	-25	-0.0622994	0.1039609	-0.3307363	0.2061375
Full	Views on hateful content	-24	-0.0435349	0.0596648	-0.1975950	0.1105252
Full	Views on hateful content	-23	-0.0785737	0.0481208	-0.2028260	0.0456787
Full	Views on hateful content	-22	-0.0021421	0.0766115	-0.1999603	0.1956760
Full	Views on hateful content	-21	0.0595144	0.2033999	-0.4656835	0.5847122
Full	Views on hateful content	-20	-0.0892152	0.2227073	-0.6642665	0.4858361
Full	Views on hateful content	-19	0.4382365	0.2909452	-0.3130117	1.1894846
Full	Views on hateful content	-18	0.1626173	0.1965499	-0.3448933	0.6701278
Full	Views on hateful content	-17	0.2860609	0.2081013	-0.2512763	0.8233981
Full	Views on hateful content	-16	0.2242095	0.3194679	-0.6006869	1.0491059

Full	Views on hateful content	-15	-0.1433289	0.2015767	-0.6638189	0.3771612
Full	Views on hateful content	-14	0.0156900	0.0802706	-0.1915762	0.2229563
Full	Views on hateful content	-13	-0.0776200	0.4212762	-1.1653950	1.0101549
Full	Views on hateful content	-12	-0.0261428	0.2462923	-0.6620927	0.6098072
Full	Views on hateful content	-11	0.0042282	0.1335033	-0.3404900	0.3489464
Full	Views on hateful content	-10	0.0264619	0.1608890	-0.3889687	0.4418925
Full	Views on hateful content	-9	-0.0017178	0.0589695	-0.1539826	0.1505470
Full	Views on hateful content	-8	-0.0950574	0.0916079	-0.3315978	0.1414830
Full	Views on hateful content	-7	-0.2969535	0.1433682	-0.6671438	0.0732367
Full	Views on hateful content	-6	-0.2764810	0.1271063	-0.6046816	0.0517196
Full	Views on hateful content	-5	-0.1100236	0.2235523	-0.6872568	0.4672095
Full	Views on hateful content	-4	-0.3098016	0.1970878	-0.8187010	0.1990977
Full	Views on hateful content	-3	-0.6516326	0.2733976	-1.3575710	0.0543058
Full	Views on hateful content	-2	0.3043354	0.0943297	0.0607670	0.5479037
Full	Views on hateful content	-1	0.1390797	0.1556495	-0.2628221	0.5409816
Full	Views on hateful content	0	0.3264885	0.2490622	-0.3166137	0.9695906
Full	Views on hateful content	1	0.6455141	0.2895689	-0.1021804	1.3932085
Full	Views on hateful content	2	0.5808065	0.3403313	-0.2979612	1.4595743
Full	Views on hateful content	3	0.7366433	0.4609544	-0.4535846	1.9268712
Full	Views on hateful content	4	0.7045301	0.5882910	-0.8144930	2.2235531
Full	Views on hateful content	5	0.5683113	0.7378935	-1.3369997	2.4736223
Full	Views on hateful content	6	0.6296860	0.5779697	-0.8626865	2.1220585
Full	Views on hateful content	7	0.7196776	0.6274394	-0.9004303	2.3397856
Full	Views on hateful content	8	0.6227902	0.6029352	-0.9340456	2.1796260
Full	Views on hateful content	9	0.5711187	0.6692642	-1.1569847	2.2992221
Full	Views on hateful content	10	0.7947511	0.8884956	-1.4994286	3.0889309
Full	Views on hateful content	11	0.8394350	0.9290681	-1.5595069	3.2383769
Full	Views on hateful content	12	0.8602878	0.8559729	-1.3499153	3.0704909
Full	Views on hateful content	13	0.6536460	1.3335561	-2.7897223	4.0970143
Full	Views on hateful content	14	1.0633073	2.1480813	-4.4832421	6.6098568
Full	Views on hateful content	15	1.1879318	2.2623929	-4.6537810	7.0296445
Full	Views on hateful content	16	1.2232917	2.2543129	-4.5975577	7.0441412
Full	Views on hateful content	17	1.2938325	2.5565339	-5.3073801	7.8950450
Full	Views on hateful content	18	1.3652559	2.6385695	-5.4477805	8.1782923
Full	Views on hateful content	19	1.1481527	2.2979633	-4.7854063	7.0817116
Full	Views on hateful content	20	0.9836128	2.1570175	-4.5860107	6.5532364
Full	Views on hateful content	21	1.1275317	2.3269465	-4.8808645	7.1359278
Full	Views on hateful content	22	1.0440213	2.4440023	-5.2666242	7.3546668
Full	Views on hateful content	23	1.2922130	2.5634114	-5.3267579	7.9111838
Full	Views on hateful content	24	1.4288045	2.6350441	-5.3751290	8.2327380
Full	Views on hateful content	25	1.2868524	2.7212453	-5.7396606	8.3133653

Full	Views on hateful content	26	1.0040433	2.3800941	-5.1415849	7.1496716
Full	Views on hateful content	27	-0.3569084	1.4913951	-4.2078318	3.4940149
Full	Views on hateful content	28	-0.1597078	1.6222436	-4.3484943	4.0290788
Full	Views on hateful content	29	-0.3603887	1.4996336	-4.2325846	3.5118073
Full	Views on hateful content	30	-0.6005315	1.2121016	-3.7302926	2.5292295

sample	outcome	event.time	estimate	std.error	conf.low	conf.high
Most	Views on hateful content	-30	-0.1491549	0.2046496	-0.6730999	0.3747902
Most	Views on hateful content	-29	-0.1273845	0.0472810	-0.2484335	-0.0063354
Most	Views on hateful content	-28	0.4005425	0.1641627	-0.0197479	0.8208328
Most	Views on hateful content	-27	-0.2051393	0.1226731	-0.5192077	0.1089290
Most	Views on hateful content	-26	0.2186495	0.1044078	-0.0486560	0.4859550
Most	Views on hateful content	-25	-0.1012121	0.1633234	-0.5193536	0.3169294
Most	Views on hateful content	-24	-0.1105709	0.1649158	-0.5327892	0.3116475
Most	Views on hateful content	-23	-0.0516073	0.0900899	-0.2822559	0.1790413
Most	Views on hateful content	-22	0.0058157	0.0920258	-0.2297893	0.2414207
Most	Views on hateful content	-21	-0.1552784	0.2625410	-0.8274373	0.5168806
Most	Views on hateful content	-20	-0.0565413	0.3055023	-0.8386901	0.7256074
Most	Views on hateful content	-19	0.5834529	0.3455374	-0.3011937	1.4680995
Most	Views on hateful content	-18	0.4558088	0.2594907	-0.2085407	1.1201583
Most	Views on hateful content	-17	0.2538064	0.3193146	-0.5637047	1.0713175
Most	Views on hateful content	-16	0.7644327	0.4994807	-0.5143405	2.0432060
Most	Views on hateful content	-15	-0.4897851	0.7044014	-2.2931976	1.3136273
Most	Views on hateful content	-14	0.1050672	0.2877117	-0.6315339	0.8416683
Most	Views on hateful content	-13	-0.1292572	0.3760254	-1.0919594	0.8334449
Most	Views on hateful content	-12	-0.1855650	0.1783897	-0.6422792	0.2711493
Most	Views on hateful content	-11	0.0499861	0.1388022	-0.3053760	0.4053482
Most	Views on hateful content	-10	0.0325999	0.1574934	-0.3706155	0.4358154
Most	Views on hateful content	-9	0.0510342	0.1480586	-0.3280261	0.4300945
Most	Views on hateful content	-8	0.0450813	0.1112398	-0.2397155	0.3298781
Most	Views on hateful content	-7	-0.3898781	0.1674852	-0.8186747	0.0389184
Most	Views on hateful content	-6	-0.1812654	0.1792281	-0.6401262	0.2775954
Most	Views on hateful content	-5	0.2624497	0.1860994	-0.2140030	0.7389024
Most	Views on hateful content	-4	-0.3335519	0.1531933	-0.7257582	0.0586545
Most	Views on hateful content	-3	-0.5796746	0.2491761	-1.2176166	0.0582674
Most	Views on hateful content	-2	0.3579821	0.1186929	0.0541039	0.6618602
Most	Views on hateful content	-1	0.1471289	0.2568017	-0.5103361	0.8045940
Most	Views on hateful content	0	0.1516155	0.2109560	-0.3884751	0.6917062
Most	Views on hateful content	1	0.6786746	0.5956885	-0.8464104	2.2037596
Most	Views on hateful content	2	0.5110269	0.2154628	-0.0406021	1.0626558
Most	Views on hateful content	3	0.6972397	0.2845723	-0.0313239	1.4258032

Most	Views on hateful content	4	0.5100972	0.3915663	-0.4923928	1.5125873
Most	Views on hateful content	5	0.1955168	0.4920032	-1.0641124	1.4551459
Most	Views on hateful content	6	0.3051642	0.4468220	-0.8387920	1.4491204
Most	Views on hateful content	7	0.4541499	0.4714165	-0.7527732	1.6610729
Most	Views on hateful content	8	0.4797969	0.4541085	-0.6828141	1.6424078
Most	Views on hateful content	9	0.2236901	0.5155468	-1.0962157	1.5435959
Most	Views on hateful content	10	0.2924721	0.6897833	-1.4735148	2.0584589
Most	Views on hateful content	11	0.2681321	0.6853146	-1.4864140	2.0226782
Most	Views on hateful content	12	0.3793847	0.6373249	-1.2522980	2.0110674
Most	Views on hateful content	13	0.1606488	0.7453151	-1.7475109	2.0688084
Most	Views on hateful content	14	0.1832502	1.3601422	-3.2989932	3.6654935
Most	Views on hateful content	15	0.1486476	1.5016330	-3.6958414	3.9931367
Most	Views on hateful content	16	0.3315466	1.6600329	-3.9184787	4.5815718
Most	Views on hateful content	17	0.2848673	1.7046056	-4.0792733	4.6490079
Most	Views on hateful content	18	0.3731277	1.5511515	-3.5981388	4.3443942
Most	Views on hateful content	19	0.4082842	1.4491290	-3.3017838	4.1183522
Most	Views on hateful content	20	0.3642263	1.4432872	-3.3308855	4.0593380
Most	Views on hateful content	21	0.1965022	1.4747135	-3.5790674	3.9720718
Most	Views on hateful content	22	0.0055629	1.7807467	-4.5535146	4.5646404
Most	Views on hateful content	23	0.2736996	1.8311265	-4.4143604	4.9617596
Most	Views on hateful content	24	0.5951903	1.6001976	-3.5016444	4.6920250
Most	Views on hateful content	25	0.2084762	1.8912787	-4.6335859	5.0505383
Most	Views on hateful content	26	0.1388170	1.6099155	-3.9828975	4.2605315
Most	Views on hateful content	27	0.0409036	1.4502142	-3.6719426	3.7537498
Most	Views on hateful content	28	-0.0228698	1.6504888	-4.2484602	4.2027207
Most	Views on hateful content	29	-0.2550860	1.3710614	-3.7652850	3.2551130
Most	Views on hateful content	30	-0.1687814	1.1474307	-3.1064398	2.7688770

sample	outcome	event.time	estimate	std.error	conf.low	conf.high
Middle	Views on hateful content	-30	-0.1495147	0.1136939	-0.4435810	0.1445516
Middle	Views on hateful content	-29	0.2324614	0.0427576	0.1218700	0.3430528
Middle	Views on hateful content	-28	-0.0351514	0.0869426	-0.2600261	0.1897234
Middle	Views on hateful content	-27	-0.0122974	0.0828702	-0.2266392	0.2020443
Middle	Views on hateful content	-26	0.0230180	0.0972081	-0.2284081	0.2744441
Middle	Views on hateful content	-25	-0.0562612	0.1122081	-0.3464847	0.2339622
Middle	Views on hateful content	-24	-0.0093383	0.0672063	-0.1831658	0.1644892
Middle	Views on hateful content	-23	-0.1333732	0.0523303	-0.2687243	0.0019778
Middle	Views on hateful content	-22	-0.0588671	0.0966270	-0.3087903	0.1910562
Middle	Views on hateful content	-21	0.0779138	0.1788271	-0.3846177	0.5404454
Middle	Views on hateful content	-20	0.0260150	0.2290507	-0.5664186	0.6184486
Middle	Views on hateful content	-19	0.5248909	0.2696392	-0.1725238	1.2223057

Middle	Views on hateful content	-18	0.0553590	0.2126405	-0.4946301	0.6053482
Middle	Views on hateful content	-17	0.5178001	0.1813014	0.0488686	0.9867315
Middle	Views on hateful content	-16	0.0618830	0.3141430	-0.7506398	0.8744057
Middle	Views on hateful content	-15	-0.0184961	0.1118953	-0.3079104	0.2709182
Middle	Views on hateful content	-14	0.0074757	0.0969890	-0.2433839	0.2583352
Middle	Views on hateful content	-13	-0.2620242	0.3094009	-1.0622817	0.5382332
Middle	Views on hateful content	-12	0.0407714	0.2148190	-0.5148524	0.5963953
Middle	Views on hateful content	-11	-0.1364966	0.1456348	-0.5131771	0.2401840
Middle	Views on hateful content	-10	0.0640705	0.1616417	-0.3540115	0.4821525
Middle	Views on hateful content	-9	0.0505879	0.0620949	-0.1100189	0.2111948
Middle	Views on hateful content	-8	-0.1615039	0.1168985	-0.4638588	0.1408510
Middle	Views on hateful content	-7	-0.2586017	0.1592740	-0.6705598	0.1533564
Middle	Views on hateful content	-6	-0.2876581	0.1518444	-0.6803996	0.1050833
Middle	Views on hateful content	-5	-0.0692669	0.1857900	-0.5498078	0.4112740
Middle	Views on hateful content	-4	-0.3042320	0.2270416	-0.8914693	0.2830053
Middle	Views on hateful content	-3	-0.5362855	0.2449549	-1.1698550	0.0972840
Middle	Views on hateful content	-2	0.2706215	0.0835300	0.0545734	0.4866695
Middle	Views on hateful content	-1	0.1709803	0.1554578	-0.2311073	0.5730679
Middle	Views on hateful content	0	0.2379535	0.2170094	-0.3233358	0.7992427
Middle	Views on hateful content	1	0.4493254	0.1979983	-0.0627922	0.9614430
Middle	Views on hateful content	2	0.4299319	0.2918258	-0.3248680	1.1847318
Middle	Views on hateful content	3	0.5632028	0.4282758	-0.5445216	1.6709271
Middle	Views on hateful content	4	0.6083636	0.5129056	-0.7182534	1.9349805
Middle	Views on hateful content	5	0.3842074	0.6256706	-1.2340734	2.0024883
Middle	Views on hateful content	6	0.4448669	0.5241852	-0.9109246	1.8006585
Middle	Views on hateful content	7	0.5818459	0.5766096	-0.9095399	2.0732317
Middle	Views on hateful content	8	0.4851926	0.5428489	-0.9188720	1.8892571
Middle	Views on hateful content	9	0.3936094	0.5417739	-1.0076748	1.7948935
Middle	Views on hateful content	10	0.6050973	0.7498277	-1.3343126	2.5445073
Middle	Views on hateful content	11	0.7062606	0.7912811	-1.3403674	2.7528885
Middle	Views on hateful content	12	0.7601698	0.7893534	-1.2814724	2.8018120
Middle	Views on hateful content	13	0.4687235	1.0757613	-2.3137052	3.2511522
Middle	Views on hateful content	14	0.7333350	1.7953065	-3.9101789	5.3768488
Middle	Views on hateful content	15	0.8487888	1.9054793	-4.0796841	5.7772618
Middle	Views on hateful content	16	0.8317346	1.8903651	-4.0576456	5.7211149
Middle	Views on hateful content	17	0.9214044	2.1689660	-4.6885701	6.5313789
Middle	Views on hateful content	18	0.9975939	2.2184869	-4.7404650	6.7356528
Middle	Views on hateful content	19	0.6620854	1.9336861	-4.3393437	5.6635145
Middle	Views on hateful content	20	0.5969201	1.8735428	-4.2489497	5.4427899
Middle	Views on hateful content	21	0.6865527	2.0519751	-4.6208276	5.9939331
Middle	Views on hateful content	22	0.5881121	1.8415461	-4.1749992	5.3512235

Middle	Views on hateful content	23	0.8414855	2.0145014	-4.3689702	6.0519411
Middle	Views on hateful content	24	0.8921970	1.9704585	-4.2043427	5.9887367
Middle	Views on hateful content	25	0.8771073	2.1281648	-4.6273358	6.3815504
Middle	Views on hateful content	26	0.5052619	1.9030682	-4.4169747	5.4274984
Middle	Views on hateful content	27	-0.3702813	1.2694580	-3.6537015	2.9131389
Middle	Views on hateful content	28	0.0658173	1.4135234	-3.5902243	3.7218589
Middle	Views on hateful content	29	-0.1354885	1.4499413	-3.8857241	3.6147471
Middle	Views on hateful content	30	-0.4968963	1.1686986	-3.5197050	2.5259124

sample	outcome	event.time	estimate	std.error	conf.low	conf.high
Least	Views on hateful content	-30	-0.0163019	0.0905504	-0.2460845	0.2134807
Least	Views on hateful content	-29	0.0683391	0.0565071	-0.0750544	0.2117326
Least	Views on hateful content	-28	-0.0631227	0.0976100	-0.3108200	0.1845746
Least	Views on hateful content	-27	0.1171065	0.1288606	-0.2098929	0.4441058
Least	Views on hateful content	-26	-0.0990748	0.1067256	-0.3699040	0.1717544
Least	Views on hateful content	-25	0.0074552	0.0877781	-0.2152923	0.2302028
Least	Views on hateful content	-24	-0.0294869	0.0766126	-0.2239006	0.1649268
Least	Views on hateful content	-23	-0.0228799	0.0475984	-0.1436665	0.0979067
Least	Views on hateful content	-22	0.1074203	0.0783967	-0.0915207	0.3063613
Least	Views on hateful content	-21	0.3722235	0.1684273	-0.0551812	0.7996281
Least	Views on hateful content	-20	-0.0987267	0.1244885	-0.4146314	0.2171780
Least	Views on hateful content	-19	0.4639484	0.1892985	-0.0164195	0.9443163
Least	Views on hateful content	-18	0.2388854	0.2270218	-0.3372099	0.8149807
Least	Views on hateful content	-17	0.2028965	0.1475834	-0.1716141	0.5774071
Least	Views on hateful content	-16	0.3156774	0.2047134	-0.2038077	0.8351625
Least	Views on hateful content	-15	-0.1541945	0.1105940	-0.4348401	0.1264511
Least	Views on hateful content	-14	-0.0217327	0.0558855	-0.1635488	0.1200834
Least	Views on hateful content	-13	-0.1865527	0.2605604	-0.8477562	0.4746509
Least	Views on hateful content	-12	-0.1593823	0.1580478	-0.5604477	0.2416831
Least	Views on hateful content	-11	0.0354531	0.0912917	-0.1962106	0.2671168
Least	Views on hateful content	-10	-0.1121691	0.1032132	-0.3740850	0.1497468
Least	Views on hateful content	-9	-0.0906441	0.0896889	-0.3182405	0.1369522
Least	Views on hateful content	-8	-0.0466102	0.1206273	-0.3527166	0.2594962
Least	Views on hateful content	-7	-0.0846656	0.0848814	-0.3000623	0.1307311
Least	Views on hateful content	-6	-0.2009449	0.1324137	-0.5369606	0.1350708
Least	Views on hateful content	-5	-0.2035701	0.1804804	-0.6615610	0.2544208
Least	Views on hateful content	-4	-0.1216974	0.1557584	-0.5169532	0.2735584
Least	Views on hateful content	-3	-0.5433020	0.2754198	-1.2422131	0.1556091
Least	Views on hateful content	-2	0.1890230	0.0757539	-0.0032118	0.3812577
Least	Views on hateful content	-1	-0.0076619	0.2462822	-0.6326327	0.6173089
Least	Views on hateful content	0	0.2882546	0.2563359	-0.3622287	0.9387380

Least	Views on hateful content	1	0.5538792	0.3217729	-0.2626585	1.3704169
Least	Views on hateful content	2	0.4201072	0.3509285	-0.4704161	1.3106306
Least	Views on hateful content	3	0.4659760	0.4218020	-0.6043975	1.5363495
Least	Views on hateful content	4	0.3370378	0.4977211	-0.9259896	1.6000652
Least	Views on hateful content	5	0.2714737	0.5477613	-1.1185368	1.6614843
Least	Views on hateful content	6	0.4294803	0.5849298	-1.0548499	1.9138104
Least	Views on hateful content	7	0.4197672	0.5935900	-1.0865392	1.9260735
Least	Views on hateful content	8	0.2605599	0.5523128	-1.1410004	1.6621203
Least	Views on hateful content	9	0.3294673	0.6393484	-1.2929564	1.9518910
Least	Views on hateful content	10	0.4571572	0.7877782	-1.5419251	2.4562395
Least	Views on hateful content	11	0.4384724	0.7202082	-1.3891430	2.2660879
Least	Views on hateful content	12	0.3799228	0.7462313	-1.5137293	2.2735749
Least	Views on hateful content	13	0.0560394	0.8449672	-2.0881669	2.2002458
Least	Views on hateful content	14	0.1512627	1.1930147	-2.8761563	3.1786817
Least	Views on hateful content	15	0.3523232	1.2634759	-2.8538996	3.5585460
Least	Views on hateful content	16	0.2546532	1.2806843	-2.9952380	3.5045444
Least	Views on hateful content	17	0.1907335	1.5118374	-3.6457365	4.0272035
Least	Views on hateful content	18	0.1949610	1.6017537	-3.8696824	4.2596045
Least	Views on hateful content	19	0.1725696	1.3431605	-3.2358624	3.5810015
Least	Views on hateful content	20	-0.0860957	1.2466295	-3.2495687	3.0773772
Least	Views on hateful content	21	0.1756161	1.3616451	-3.2797229	3.6309550
Least	Views on hateful content	22	0.1104937	1.4182621	-3.4885178	3.7095051
Least	Views on hateful content	23	0.2189236	1.4486435	-3.4571842	3.8950315
Least	Views on hateful content	24	0.3316479	1.4319175	-3.3020156	3.9653115
Least	Views on hateful content	25	0.2307322	1.4778913	-3.5195955	3.9810599
Least	Views on hateful content	26	0.0714056	1.3884692	-3.4520027	3.5948138
Least	Views on hateful content	27	-1.7667599	0.4513306	-2.9120658	-0.6214540
Least	Views on hateful content	28	-1.7634212	0.5649560	-3.1970653	-0.3297772
Least	Views on hateful content	29	-1.8784675	0.4879249	-3.1166359	-0.6402992
Least	Views on hateful content	30	-2.0231656	0.5490991	-3.4165708	-0.6297603

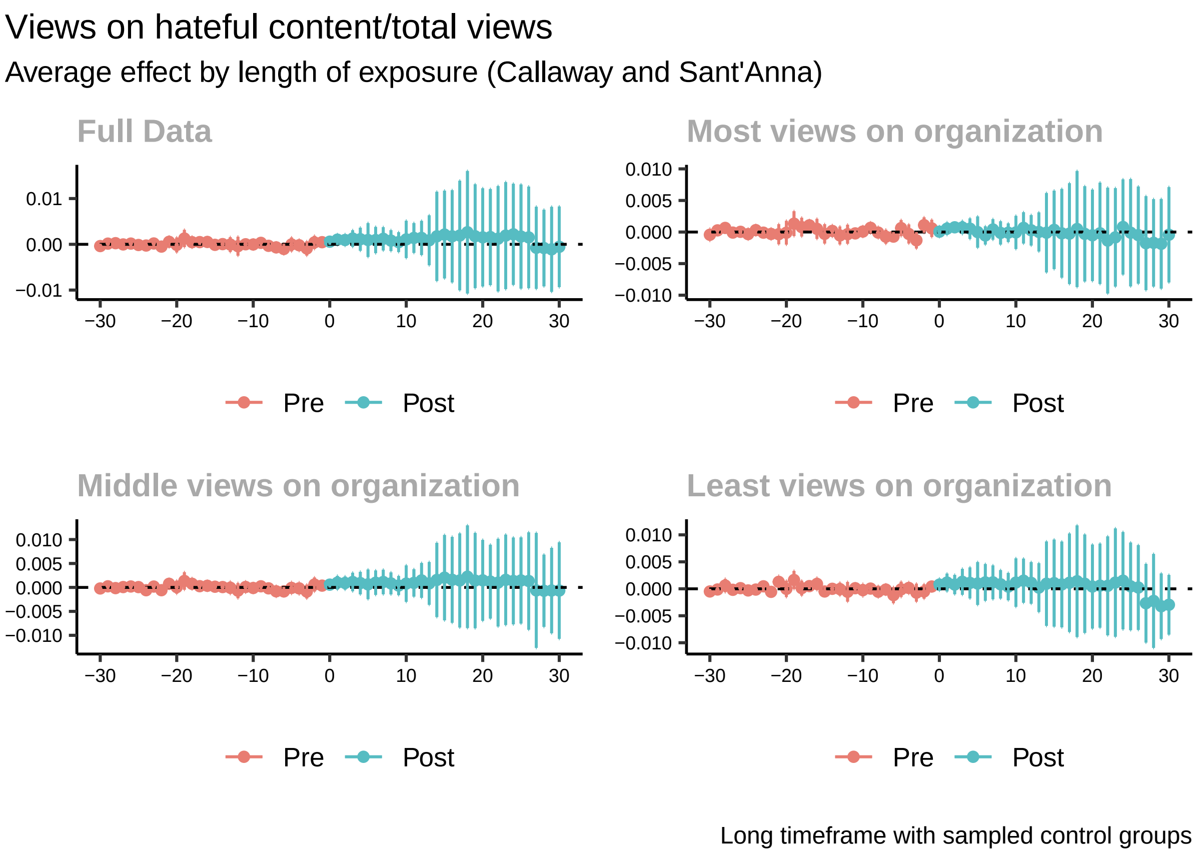

sample	outcome	event.time	estimate	std.error	conf.low	conf.high
Full	Views on hateful content/total views	-30	-0.0003835	0.0002002	-0.0008975	0.0001304
Full	Views on hateful content/total views	-29	0.0001506	0.0001601	-0.0002602	0.0005614
Full	Views on hateful content/total views	-28	0.0002477	0.0002559	-0.0004091	0.0009044
Full	Views on hateful content/total views	-27	-0.0000272	0.0002276	-0.0006113	0.0005569
Full	Views on hateful content/total views	-26	0.0001613	0.0002203	-0.0004043	0.0007268
Full	Views on hateful content/total views	-25	-0.0001468	0.0002485	-0.0007848	0.0004911
Full	Views on hateful content/total views	-24	-0.0002676	0.0002561	-0.0009248	0.0003896
Full	Views on hateful content/total views	-23	0.0001850	0.0002761	-0.0005237	0.0008938
Full	Views on hateful content/total views	-22	-0.0004981	0.0002245	-0.0010741	0.0000780
Full	Views on hateful content/total views	-21	0.0005464	0.0004516	-0.0006126	0.0017053
Full	Views on hateful content/total views	-20	-0.0001295	0.0006356	-0.0017609	0.0015018
Full	Views on hateful content/total views	-19	0.0012700	0.0007352	-0.0006171	0.0031571
Full	Views on hateful content/total views	-18	0.0004713	0.0004709	-0.0007373	0.0016799
Full	Views on hateful content/total views	-17	0.0004835	0.0002437	-0.0001419	0.0011089
Full	Views on hateful content/total views	-16	0.0005136	0.0003949	-0.0005001	0.0015272
Full	Views on hateful content/total views	-15	-0.0001156	0.0003708	-0.0010673	0.0008361
Full	Views on hateful content/total views	-14	0.0000425	0.0002267	-0.0005392	0.0006243
Full	Views on hateful content/total views	-13	-0.0000514	0.0006298	-0.0016679	0.0015651
Full	Views on hateful content/total views	-12	-0.0004130	0.0008090	-0.0024893	0.0016633

Full	Views on hateful content/total views	-11	0.0000083	0.0003459	-0.0008795	0.0008961
Full	Views on hateful content/total views	-10	-0.0000392	0.0004152	-0.0011049	0.0010265
Full	Views on hateful content/total views	-9	0.0003163	0.0002750	-0.0003895	0.0010220
Full	Views on hateful content/total views	-8	-0.0003280	0.0003630	-0.0012596	0.0006036
Full	Views on hateful content/total views	-7	-0.0006675	0.0004144	-0.0017312	0.0003961
Full	Views on hateful content/total views	-6	-0.0010189	0.0004478	-0.0021683	0.0001305
Full	Views on hateful content/total views	-5	-0.0000287	0.0005938	-0.0015528	0.0014955
Full	Views on hateful content/total views	-4	-0.0002082	0.0004804	-0.0014412	0.0010249
Full	Views on hateful content/total views	-3	-0.0009052	0.0006106	-0.0024723	0.0006619
Full	Views on hateful content/total views	-2	0.0004157	0.0005589	-0.0010188	0.0018502
Full	Views on hateful content/total views	-1	0.0004638	0.0003158	-0.0003466	0.0012743
Full	Views on hateful content/total views	0	0.0005800	0.0002713	-0.0001164	0.0012764
Full	Views on hateful content/total views	1	0.0010171	0.0004602	-0.0001639	0.0021982
Full	Views on hateful content/total views	2	0.0009418	0.0004926	-0.0003226	0.0022062
Full	Views on hateful content/total views	3	0.0012604	0.0007074	-0.0005553	0.0030761
Full	Views on hateful content/total views	4	0.0011170	0.0009824	-0.0014043	0.0036383
Full	Views on hateful content/total views	5	0.0009433	0.0014402	-0.0027530	0.0046396
Full	Views on hateful content/total views	6	0.0009296	0.0011161	-0.0019349	0.0037941
Full	Views on hateful content/total views	7	0.0012043	0.0009891	-0.0013344	0.0037430
Full	Views on hateful content/total views	8	0.0008090	0.0008907	-0.0014770	0.0030949
Full	Views on hateful content/total views	9	0.0004799	0.0008446	-0.0016880	0.0026478
Full	Views on hateful content/total views	10	0.0010777	0.0015858	-0.0029925	0.0051479
Full	Views on hateful content/total views	11	0.0013886	0.0012698	-0.0018705	0.0046477
Full	Views on hateful content/total views	12	0.0013966	0.0014519	-0.0023297	0.0051230
Full	Views on hateful content/total views	13	0.0008775	0.0021231	-0.0045717	0.0063267
Full	Views on hateful content/total views	14	0.0017450	0.0037940	-0.0079928	0.0114828
Full	Views on hateful content/total views	15	0.0021376	0.0037329	-0.0074433	0.0117185
Full	Views on hateful content/total views	16	0.0017540	0.0039222	-0.0083127	0.0118206
Full	Views on hateful content/total views	17	0.0019276	0.0046662	-0.0100486	0.0139039
Full	Views on hateful content/total views	18	0.0026411	0.0052152	-0.0107444	0.0160265
Full	Views on hateful content/total views	19	0.0017707	0.0044175	-0.0095672	0.0131086
Full	Views on hateful content/total views	20	0.0015181	0.0041753	-0.0091984	0.0122346
Full	Views on hateful content/total views	21	0.0015761	0.0040904	-0.0089225	0.0120746
Full	Views on hateful content/total views	22	0.0012286	0.0044836	-0.0102790	0.0127363
Full	Views on hateful content/total views	23	0.0019076	0.0045559	-0.0097855	0.0136007
Full	Views on hateful content/total views	24	0.0021841	0.0043011	-0.0088551	0.0132234
Full	Views on hateful content/total views	25	0.0017019	0.0044334	-0.0096770	0.0130808
Full	Views on hateful content/total views	26	0.0015131	0.0043279	-0.0095948	0.0126211
Full	Views on hateful content/total views	27	-0.0007594	0.0034944	-0.0097281	0.0082093
Full	Views on hateful content/total views	28	-0.0008132	0.0032592	-0.0091782	0.0075518
Full	Views on hateful content/total views	29	-0.0010924	0.0036182	-0.0103789	0.0081941

Full	Views on hateful content/total views	30	-0.0005462	0.0034334	-0.0093584	0.0082660

sample	outcome	event.time	estimate	std.error	conf.low	conf.high
Most	Views on hateful content/total views	-30	-0.0004307	0.0003748	-0.0013830	0.0005216
Most	Views on hateful content/total views	-29	0.0002631	0.0002126	-0.0002770	0.0008032
Most	Views on hateful content/total views	-28	0.0006517	0.0002354	0.0000535	0.0012498
Most	Views on hateful content/total views	-27	-0.0000848	0.0002263	-0.0006598	0.0004901
Most	Views on hateful content/total views	-26	0.0000371	0.0002138	-0.0005061	0.0005802
Most	Views on hateful content/total views	-25	-0.0003267	0.0003369	-0.0011826	0.0005292
Most	Views on hateful content/total views	-24	0.0003004	0.0003136	-0.0004962	0.0010970
Most	Views on hateful content/total views	-23	-0.0001008	0.0002667	-0.0007782	0.0005766
Most	Views on hateful content/total views	-22	-0.0003017	0.0003409	-0.0011678	0.0005645
Most	Views on hateful content/total views	-21	-0.0003423	0.0006267	-0.0019344	0.0012498
Most	Views on hateful content/total views	-20	-0.0001062	0.0007413	-0.0019893	0.0017770
Most	Views on hateful content/total views	-19	0.0013260	0.0007743	-0.0006410	0.0032930
Most	Views on hateful content/total views	-18	0.0007805	0.0005836	-0.0007022	0.0022631
Most	Views on hateful content/total views	-17	0.0010634	0.0003255	0.0002365	0.0018903
Most	Views on hateful content/total views	-16	0.0005035	0.0006227	-0.0010785	0.0020855
Most	Views on hateful content/total views	-15	-0.0002865	0.0006021	-0.0018162	0.0012431
Most	Views on hateful content/total views	-14	0.0001738	0.0003613	-0.0007440	0.0010916
Most	Views on hateful content/total views	-13	-0.0005381	0.0005563	-0.0019513	0.0008750
Most	Views on hateful content/total views	-12	-0.0003417	0.0005953	-0.0018540	0.0011705
Most	Views on hateful content/total views	-11	-0.0002128	0.0002723	-0.0009047	0.0004790
Most	Views on hateful content/total views	-10	0.0000792	0.0003296	-0.0007581	0.0009165
Most	Views on hateful content/total views	-9	0.0006699	0.0003389	-0.0001910	0.0015307
Most	Views on hateful content/total views	-8	-0.0000832	0.0003581	-0.0009930	0.0008267
Most	Views on hateful content/total views	-7	-0.0007387	0.0004413	-0.0018597	0.0003822
Most	Views on hateful content/total views	-6	-0.0007236	0.0002868	-0.0014522	0.0000050
Most	Views on hateful content/total views	-5	0.0006223	0.0005021	-0.0006532	0.0018978
Most	Views on hateful content/total views	-4	-0.0003043	0.0005944	-0.0018144	0.0012057
Most	Views on hateful content/total views	-3	-0.0013082	0.0005119	-0.0026086	-0.0000078
Most	Views on hateful content/total views	-2	0.0010927	0.0004831	-0.0001345	0.0023200
Most	Views on hateful content/total views	-1	0.0005812	0.0005374	-0.0007839	0.0019464
Most	Views on hateful content/total views	0	0.0000589	0.0003332	-0.0007875	0.0009053
Most	Views on hateful content/total views	1	0.0005399	0.0003916	-0.0004549	0.0015348
Most	Views on hateful content/total views	2	0.0007514	0.0002419	0.0001368	0.0013660
Most	Views on hateful content/total views	3	0.0007225	0.0004090	-0.0003166	0.0017616
Most	Views on hateful content/total views	4	0.0005328	0.0006385	-0.0010891	0.0021548
Most	Views on hateful content/total views	5	-0.0000075	0.0009746	-0.0024834	0.0024683
Most	Views on hateful content/total views	6	-0.0005384	0.0005654	-0.0019748	0.0008981
Most	Views on hateful content/total views	7	0.0004089	0.0006407	-0.0012187	0.0020365

Most	Views on hateful content/total views	8	-0.0001384	0.0007224	-0.0019736	0.0016969
Most	Views on hateful content/total views	9	-0.0001040	0.0005777	-0.0015715	0.0013636
Most	Views on hateful content/total views	10	-0.0000611	0.0010298	-0.0026772	0.0025550
Most	Views on hateful content/total views	11	0.0006585	0.0009731	-0.0018135	0.0031305
Most	Views on hateful content/total views	12	0.0002748	0.0009489	-0.0021358	0.0026854
Most	Views on hateful content/total views	13	0.0000172	0.0012164	-0.0030729	0.0031072
Most	Views on hateful content/total views	14	-0.0001039	0.0024720	-0.0063838	0.0061761
Most	Views on hateful content/total views	15	0.0003447	0.0024449	-0.0058664	0.0065557
Most	Views on hateful content/total views	16	-0.0002015	0.0027659	-0.0072281	0.0068251
Most	Views on hateful content/total views	17	-0.0002514	0.0031397	-0.0082276	0.0077247
Most	Views on hateful content/total views	18	0.0004776	0.0036178	-0.0087130	0.0096682
Most	Views on hateful content/total views	19	-0.0002849	0.0029658	-0.0078192	0.0072495
Most	Views on hateful content/total views	20	-0.0005163	0.0028490	-0.0077539	0.0067213
Most	Views on hateful content/total views	21	-0.0001947	0.0031582	-0.0082177	0.0078283
Most	Views on hateful content/total views	22	-0.0013519	0.0032974	-0.0097286	0.0070248
Most	Views on hateful content/total views	23	-0.0008374	0.0030677	-0.0086305	0.0069557
Most	Views on hateful content/total views	24	0.0008022	0.0029644	-0.0067285	0.0083328
Most	Views on hateful content/total views	25	-0.0001067	0.0033431	-0.0085995	0.0083861
Most	Views on hateful content/total views	26	-0.0004861	0.0030285	-0.0081797	0.0072075
Most	Views on hateful content/total views	27	-0.0017644	0.0029292	-0.0092056	0.0056768
Most	Views on hateful content/total views	28	-0.0017111	0.0027246	-0.0086327	0.0052105
Most	Views on hateful content/total views	29	-0.0018629	0.0027926	-0.0089572	0.0052314
Most	Views on hateful content/total views	30	-0.0004259	0.0029700	-0.0079708	0.0071191

sample	outcome	event.time	estimate	std.error	conf.low	conf.high
Middle	Views on hateful content/total views	-30	-0.0002154	0.0002192	-0.0007603	0.0003295
Middle	Views on hateful content/total views	-29	0.0002339	0.0002503	-0.0003885	0.0008563
Middle	Views on hateful content/total views	-28	-0.0001423	0.0003646	-0.0010489	0.0007643
Middle	Views on hateful content/total views	-27	0.0000774	0.0002615	-0.0005729	0.0007276
Middle	Views on hateful content/total views	-26	0.0001998	0.0002779	-0.0004911	0.0008908
Middle	Views on hateful content/total views	-25	0.0000740	0.0002200	-0.0004730	0.0006210
Middle	Views on hateful content/total views	-24	-0.0005822	0.0003036	-0.0013370	0.0001726
Middle	Views on hateful content/total views	-23	0.0001852	0.0002516	-0.0004403	0.0008107
Middle	Views on hateful content/total views	-22	-0.0005724	0.0002032	-0.0010776	-0.0000672
Middle	Views on hateful content/total views	-21	0.0007578	0.0004079	-0.0002564	0.0017721
Middle	Views on hateful content/total views	-20	0.0000633	0.0005516	-0.0013084	0.0014349
Middle	Views on hateful content/total views	-19	0.0013521	0.0007337	-0.0004721	0.0031764
Middle	Views on hateful content/total views	-18	0.0007650	0.0004699	-0.0004034	0.0019334
Middle	Views on hateful content/total views	-17	0.0002594	0.0002485	-0.0003586	0.0008773
Middle	Views on hateful content/total views	-16	0.0003472	0.0004076	-0.0006662	0.0013606
Middle	Views on hateful content/total views	-15	0.0001514	0.0003799	-0.0007932	0.0010960

Middle	Views on hateful content/total views	-14	0.0000793	0.0002495	-0.0005412	0.0006998
Middle	Views on hateful content/total views	-13	-0.0000681	0.0005238	-0.0013704	0.0012342
Middle	Views on hateful content/total views	-12	-0.0006757	0.0006390	-0.0022646	0.0009131
Middle	Views on hateful content/total views	-11	0.0000933	0.0004680	-0.0010702	0.0012569
Middle	Views on hateful content/total views	-10	-0.0001153	0.0004068	-0.0011268	0.0008962
Middle	Views on hateful content/total views	-9	0.0002358	0.0002707	-0.0004372	0.0009088
Middle	Views on hateful content/total views	-8	-0.0001905	0.0003720	-0.0011155	0.0007346
Middle	Views on hateful content/total views	-7	-0.0008100	0.0004523	-0.0019345	0.0003146
Middle	Views on hateful content/total views	-6	-0.0008390	0.0003858	-0.0017983	0.0001203
Middle	Views on hateful content/total views	-5	-0.0000990	0.0004947	-0.0013292	0.0011311
Middle	Views on hateful content/total views	-4	-0.0001947	0.0005055	-0.0014515	0.0010622
Middle	Views on hateful content/total views	-3	-0.0008612	0.0005824	-0.0023092	0.0005869
Middle	Views on hateful content/total views	-2	0.0006069	0.0005897	-0.0008592	0.0020731
Middle	Views on hateful content/total views	-1	0.0003778	0.0003284	-0.0004388	0.0011943
Middle	Views on hateful content/total views	0	0.0005838	0.0002494	-0.0000363	0.0012039
Middle	Views on hateful content/total views	1	0.0009885	0.0005750	-0.0004412	0.0024183
Middle	Views on hateful content/total views	2	0.0009212	0.0005547	-0.0004579	0.0023004
Middle	Views on hateful content/total views	3	0.0011354	0.0007775	-0.0007977	0.0030686
Middle	Views on hateful content/total views	4	0.0009053	0.0009481	-0.0014521	0.0032628
Middle	Views on hateful content/total views	5	0.0006444	0.0012424	-0.0024447	0.0037335
Middle	Views on hateful content/total views	6	0.0009881	0.0010244	-0.0015591	0.0035352
Middle	Views on hateful content/total views	7	0.0011388	0.0010285	-0.0014184	0.0036960
Middle	Views on hateful content/total views	8	0.0008405	0.0009244	-0.0014580	0.0031390
Middle	Views on hateful content/total views	9	0.0003947	0.0007994	-0.0015931	0.0023825
Middle	Views on hateful content/total views	10	0.0008023	0.0015298	-0.0030015	0.0046062
Middle	Views on hateful content/total views	11	0.0009462	0.0011484	-0.0019091	0.0038016
Middle	Views on hateful content/total views	12	0.0014658	0.0014618	-0.0021689	0.0051005
Middle	Views on hateful content/total views	13	0.0008423	0.0017937	-0.0036176	0.0053022
Middle	Views on hateful content/total views	14	0.0015667	0.0031170	-0.0061835	0.0093169
Middle	Views on hateful content/total views	15	0.0020392	0.0035818	-0.0068668	0.0109452
Middle	Views on hateful content/total views	16	0.0015990	0.0036075	-0.0073708	0.0105688
Middle	Views on hateful content/total views	17	0.0014520	0.0039503	-0.0083700	0.0112741
Middle	Views on hateful content/total views	18	0.0022396	0.0043102	-0.0084773	0.0129565
Middle	Views on hateful content/total views	19	0.0014335	0.0039972	-0.0085053	0.0113723
Middle	Views on hateful content/total views	20	0.0014800	0.0033937	-0.0069582	0.0099182
Middle	Views on hateful content/total views	21	0.0012123	0.0030993	-0.0064940	0.0089185
Middle	Views on hateful content/total views	22	0.0010004	0.0036835	-0.0081584	0.0101592
Middle	Views on hateful content/total views	23	0.0015875	0.0037920	-0.0078411	0.0110162
Middle	Views on hateful content/total views	24	0.0013489	0.0036444	-0.0077127	0.0104105
Middle	Views on hateful content/total views	25	0.0014597	0.0036191	-0.0075389	0.0104583
Middle	Views on hateful content/total views	26	0.0013483	0.0040874	-0.0088147	0.0115113

Middle	Views on hateful content/total views	27	-0.0006231	0.0048268	-0.0126248	0.0113785
Middle	Views on hateful content/total views	28	-0.0006975	0.0030336	-0.0082404	0.0068455
Middle	Views on hateful content/total views	29	-0.0006444	0.0035858	-0.0095604	0.0082715
Middle	Views on hateful content/total views	30	-0.0006505	0.0040539	-0.0107302	0.0094292

sample	outcome	event.time	estimate	std.error	conf.low	conf.high
Least	Views on hateful content/total views	-30	-0.0004957	0.0001845	-0.0009821	-0.0000093
Least	Views on hateful content/total views	-29	-0.0001269	0.0001306	-0.0004710	0.0002173
Least	Views on hateful content/total views	-28	0.0006112	0.0004690	-0.0006250	0.0018473
Least	Views on hateful content/total views	-27	-0.0001889	0.0003181	-0.0010273	0.0006496
Least	Views on hateful content/total views	-26	0.0001179	0.0002413	-0.0005180	0.0007538
Least	Views on hateful content/total views	-25	-0.0003025	0.0002657	-0.0010028	0.0003977
Least	Views on hateful content/total views	-24	-0.0001412	0.0003063	-0.0009486	0.0006662
Least	Views on hateful content/total views	-23	0.0004620	0.0002892	-0.0003003	0.0012244
Least	Views on hateful content/total views	-22	-0.0005617	0.0002606	-0.0012486	0.0001252
Least	Views on hateful content/total views	-21	0.0012850	0.0004242	0.0001668	0.0024032
Least	Views on hateful content/total views	-20	-0.0000098	0.0005736	-0.0015217	0.0015021
Least	Views on hateful content/total views	-19	0.0016306	0.0006351	-0.0000433	0.0033046
Least	Views on hateful content/total views	-18	0.0001288	0.0005241	-0.0012527	0.0015103
Least	Views on hateful content/total views	-17	0.0004989	0.0002283	-0.0001027	0.0011006
Least	Views on hateful content/total views	-16	0.0009043	0.0004368	-0.0002470	0.0020557
Least	Views on hateful content/total views	-15	-0.0005016	0.0002930	-0.0012739	0.0002706
Least	Views on hateful content/total views	-14	0.0000025	0.0002851	-0.0007488	0.0007539
Least	Views on hateful content/total views	-13	-0.0000606	0.0004578	-0.0012673	0.0011462
Least	Views on hateful content/total views	-12	-0.0005515	0.0006916	-0.0023744	0.0012714
Least	Views on hateful content/total views	-11	0.0001079	0.0003302	-0.0007623	0.0009781
Least	Views on hateful content/total views	-10	-0.0002457	0.0004405	-0.0014068	0.0009153
Least	Views on hateful content/total views	-9	0.0000398	0.0002950	-0.0007378	0.0008174
Least	Views on hateful content/total views	-8	-0.0005390	0.0004032	-0.0016017	0.0005236
Least	Views on hateful content/total views	-7	-0.0001419	0.0003591	-0.0010883	0.0008045
Least	Views on hateful content/total views	-6	-0.0011748	0.0005656	-0.0026655	0.0003159
Least	Views on hateful content/total views	-5	-0.0001041	0.0005373	-0.0015202	0.0013121
Least	Views on hateful content/total views	-4	0.0001543	0.0004112	-0.0009295	0.0012381
Least	Views on hateful content/total views	-3	-0.0007158	0.0006303	-0.0023771	0.0009454
Least	Views on hateful content/total views	-2	-0.0004889	0.0005087	-0.0018298	0.0008519
Least	Views on hateful content/total views	-1	0.0004269	0.0003343	-0.0004543	0.0013081
Least	Views on hateful content/total views	0	0.0007722	0.0004224	-0.0003411	0.0018855
Least	Views on hateful content/total views	1	0.0011583	0.0006277	-0.0004961	0.0028127
Least	Views on hateful content/total views	2	0.0007861	0.0006807	-0.0010080	0.0025801
Least	Views on hateful content/total views	3	0.0012974	0.0009123	-0.0011073	0.0037021
Least	Views on hateful content/total views	4	0.0010874	0.0010918	-0.0017902	0.0039649

Least	Views on hateful content/total views	5	0.0009810	0.0015063	-0.0029891	0.0049512
Least	Views on hateful content/total views	6	0.0011879	0.0012880	-0.0022071	0.0045828
Least	Views on hateful content/total views	7	0.0012025	0.0011894	-0.0019325	0.0043374
Least	Views on hateful content/total views	8	0.0008318	0.0009982	-0.0017991	0.0034626
Least	Views on hateful content/total views	9	0.0004052	0.0009614	-0.0021288	0.0029393
Least	Views on hateful content/total views	10	0.0011585	0.0017049	-0.0033350	0.0056520
Least	Views on hateful content/total views	11	0.0015023	0.0015492	-0.0025809	0.0055854
Least	Views on hateful content/total views	12	0.0010967	0.0014620	-0.0027566	0.0049500
Least	Views on hateful content/total views	13	0.0002234	0.0017158	-0.0042990	0.0047457
Least	Views on hateful content/total views	14	0.0009470	0.0029650	-0.0068678	0.0087618
Least	Views on hateful content/total views	15	0.0010557	0.0030589	-0.0070066	0.0091181
Least	Views on hateful content/total views	16	0.0008019	0.0030159	-0.0071472	0.0087509
Least	Views on hateful content/total views	17	0.0011177	0.0034599	-0.0080016	0.0102370
Least	Views on hateful content/total views	18	0.0014067	0.0039264	-0.0089422	0.0117555
Least	Views on hateful content/total views	19	0.0009380	0.0034600	-0.0081816	0.0100577
Least	Views on hateful content/total views	20	0.0004143	0.0029515	-0.0073651	0.0081936
Least	Views on hateful content/total views	21	0.0005872	0.0029539	-0.0071984	0.0083728
Least	Views on hateful content/total views	22	0.0005506	0.0034775	-0.0086149	0.0097162
Least	Views on hateful content/total views	23	0.0011553	0.0038076	-0.0088804	0.0111910
Least	Views on hateful content/total views	24	0.0015025	0.0034213	-0.0075150	0.0105201
Least	Views on hateful content/total views	25	0.0004547	0.0030784	-0.0076591	0.0085685
Least	Views on hateful content/total views	26	0.0002358	0.0029817	-0.0076231	0.0080946
Least	Views on hateful content/total views	27	-0.0026704	0.0027676	-0.0099649	0.0046242
Least	Views on hateful content/total views	28	-0.0022497	0.0033026	-0.0109544	0.0064550
Least	Views on hateful content/total views	29	-0.0032297	0.0023041	-0.0093026	0.0028432
Least	Views on hateful content/total views	30	-0.0029588	0.0020974	-0.0084869	0.0025693

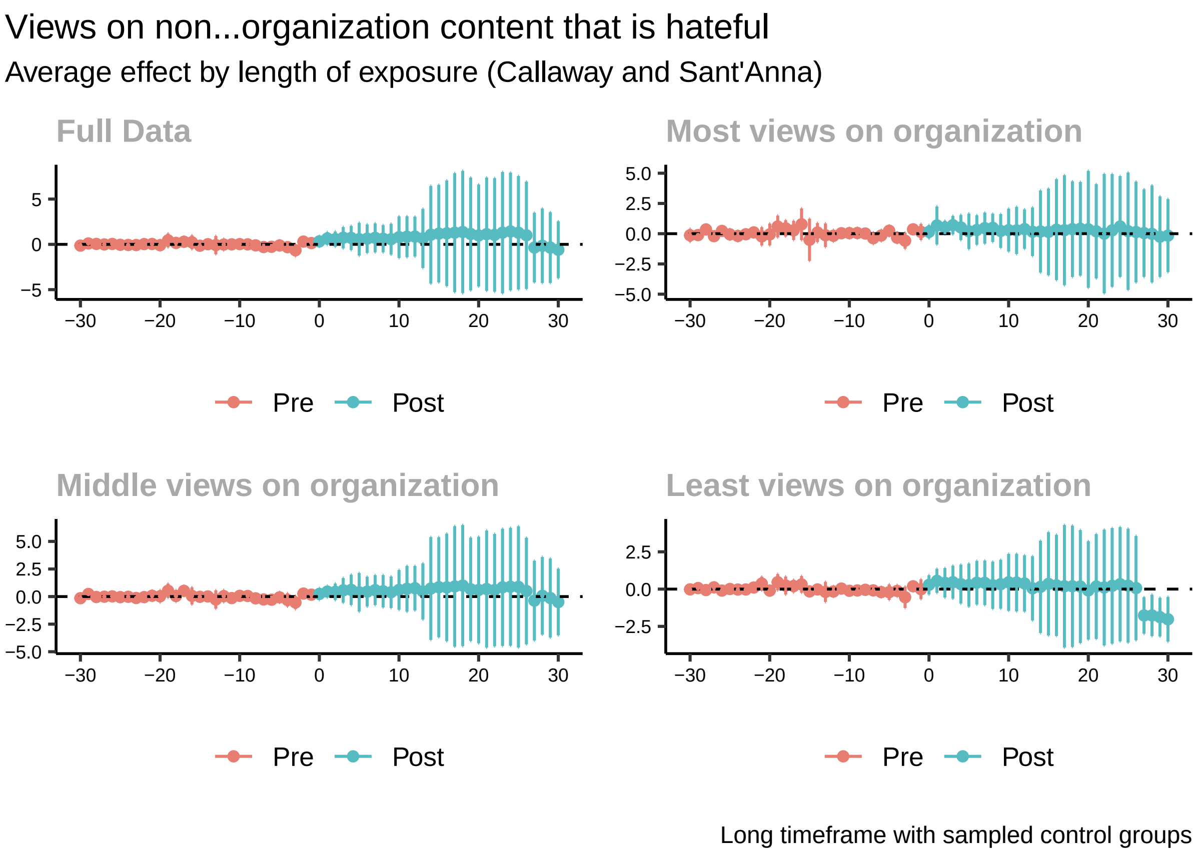

sample	outcome	event.time	estimate	std.error	conf.low	conf.high
Full	Views on nonorganization content that is hateful	-30	-0.1368013	0.1127407	-0.4232723	0.1496697
Full	Views on nonorganization content that is hateful	-29	0.0996605	0.0420512	-0.0071905	0.2065115
Full	Views on nonorganization content that is hateful	-28	0.0491642	0.0638796	-0.1131521	0.2114805
Full	Views on nonorganization content that is hateful	-27	-0.0110696	0.0556182	-0.1523940	0.1302547
Full	Views on nonorganization content that is hateful	-26	0.0463409	0.0864785	-0.1733986	0.2660805
Full	Views on nonorganization content that is hateful	-25	-0.0497420	0.1025122	-0.3102228	0.2107388
Full	Views on nonorganization content that is hateful	-24	-0.0670933	0.0594352	-0.2181165	0.0839299
Full	Views on nonorganization content that is hateful	-23	-0.0753114	0.0478822	-0.1969788	0.0463560
Full	Views on nonorganization content that is hateful	-22	0.0268807	0.0786983	-0.1730897	0.2268510
Full	Views on nonorganization content that is hateful	-21	0.0421100	0.2036506	-0.4753608	0.5595808
Full	Views on nonorganization content that is hateful	-20	-0.0902887	0.2177278	-0.6435292	0.4629518
Full	Views on nonorganization content that is hateful	-19	0.4415628	0.3001775	-0.3211803	1.2043058
Full	Views on nonorganization content that is hateful	-18	0.1568962	0.2070317	-0.3691659	0.6829583
Full	Views on nonorganization content that is hateful	-17	0.2886360	0.2162509	-0.2608519	0.8381238
Full	Views on nonorganization content that is hateful	-16	0.2257722	0.3011985	-0.5395651	0.9911095
Full	Views on nonorganization content that is hateful	-15	-0.1482529	0.1962037	-0.6468013	0.3502955
Full	Views on nonorganization content that is hateful	-14	0.0160463	0.0811834	-0.1902386	0.2223311
Full	Views on nonorganization content that is hateful	-13	-0.0793737	0.3961540	-1.0859904	0.9272430
Full	Views on nonorganization content that is hateful	-12	-0.0209065	0.2508601	-0.6583353	0.6165222

Full	Views on nonorganization content that is hateful	-11	0.0004246	0.1281623	-0.3252325	0.3260816
Full	Views on nonorganization content that is hateful	-10	0.0289888	0.1565311	-0.3687526	0.4267302
Full	Views on nonorganization content that is hateful	-9	0.0015560	0.0580859	-0.1460387	0.1491507
Full	Views on nonorganization content that is hateful	-8	-0.1071805	0.0904873	-0.3371063	0.1227453
Full	Views on nonorganization content that is hateful	-7	-0.2944566	0.1452534	-0.6635415	0.0746284
Full	Views on nonorganization content that is hateful	-6	-0.2662268	0.1253164	-0.5846523	0.0521987
Full	Views on nonorganization content that is hateful	-5	-0.1191918	0.2110431	-0.6554467	0.4170631
Full	Views on nonorganization content that is hateful	-4	-0.3086458	0.2045277	-0.8283452	0.2110537
Full	Views on nonorganization content that is hateful	-3	-0.6512990	0.2583300	-1.3077086	0.0051106
Full	Views on nonorganization content that is hateful	-2	0.3047755	0.0895023	0.0773526	0.5321984
Full	Views on nonorganization content that is hateful	-1	0.1388601	0.1517495	-0.2467313	0.5244514
Full	Views on nonorganization content that is hateful	0	0.3281120	0.2356267	-0.2706092	0.9268333
Full	Views on nonorganization content that is hateful	1	0.6453049	0.2777486	-0.0604469	1.3510567
Full	Views on nonorganization content that is hateful	2	0.5803866	0.3305927	-0.2596407	1.4204139
Full	Views on nonorganization content that is hateful	3	0.7378207	0.4622795	-0.4368191	1.9124606
Full	Views on nonorganization content that is hateful	4	0.7054097	0.5245446	-0.6274442	2.0382637
Full	Views on nonorganization content that is hateful	5	0.5687543	0.7112497	-1.2385122	2.3760208
Full	Views on nonorganization content that is hateful	6	0.6291479	0.6180477	-0.9412949	2.1995907
Full	Views on nonorganization content that is hateful	7	0.7220098	0.6267548	-0.8705574	2.3145770
Full	Views on nonorganization content that is hateful	8	0.6228803	0.5885312	-0.8725618	2.1183225
Full	Views on nonorganization content that is hateful	9	0.5709800	0.6682409	-1.1270022	2.2689623
Full	Views on nonorganization content that is hateful	10	0.7948332	0.9032289	-1.5002473	3.0899137
Full	Views on nonorganization content that is hateful	11	0.8395056	0.8874403	-1.4154565	3.0944677
Full	Views on nonorganization content that is hateful	12	0.8594319	0.8651583	-1.3389123	3.0577761
Full	Views on nonorganization content that is hateful	13	0.6524903	1.2828580	-2.6072175	3.9121981
Full	Views on nonorganization content that is hateful	14	1.0609339	2.1216798	-4.3301978	6.4520656
Full	Views on nonorganization content that is hateful	15	1.1853000	2.1163703	-4.1923404	6.5629404
Full	Views on nonorganization content that is hateful	16	1.2219468	2.2931972	-4.6050061	7.0488996
Full	Views on nonorganization content that is hateful	17	1.2915406	2.5823567	-5.2701588	7.8532400
Full	Views on nonorganization content that is hateful	18	1.3641489	2.6560462	-5.3847936	8.1130914
Full	Views on nonorganization content that is hateful	19	1.1456690	2.4480502	-5.0747613	7.3660993
Full	Views on nonorganization content that is hateful	20	0.9815924	2.2133387	-4.6424421	6.6056269
Full	Views on nonorganization content that is hateful	21	1.1247623	2.4580006	-5.1209516	7.3704763
Full	Views on nonorganization content that is hateful	22	1.0414546	2.4635688	-5.2184079	7.3013170
Full	Views on nonorganization content that is hateful	23	1.2897772	2.6346106	-5.4046979	7.9842524
Full	Views on nonorganization content that is hateful	24	1.4261540	2.5548809	-5.0657302	7.9180382
Full	Views on nonorganization content that is hateful	25	1.2838520	2.4657212	-4.9814799	7.5491838
Full	Views on nonorganization content that is hateful	26	1.0012413	2.3282966	-4.9148982	6.9173809
Full	Views on nonorganization content that is hateful	27	-0.3531270	1.5120232	-4.1951376	3.4888837
Full	Views on nonorganization content that is hateful	28	-0.1562369	1.6155508	-4.2613083	3.9488344
Full	Views on nonorganization content that is hateful	29	-0.3571249	1.5338220	-4.2545256	3.5402757

Full	Views on nonorganization content that is hateful	30	-0.5971643	1.2336759	-3.7319017	2.5375731

sample	outcome	event.time	estimate	std.error	conf.low	conf.high
Most	Views on nonorganization content that is hateful	-30	-0.1329868	0.2084557	-0.6719859	0.4060122
Most	Views on nonorganization content that is hateful	-29	-0.1167159	0.0567958	-0.2635713	0.0301395
Most	Views on nonorganization content that is hateful	-28	0.3507185	0.1516617	-0.0414295	0.7428666
Most	Views on nonorganization content that is hateful	-27	-0.2050320	0.1254172	-0.5293202	0.1192563
Most	Views on nonorganization content that is hateful	-26	0.2280637	0.0993726	-0.0288817	0.4850092
Most	Views on nonorganization content that is hateful	-25	-0.0587840	0.1669108	-0.4903613	0.3727933
Most	Views on nonorganization content that is hateful	-24	-0.1883830	0.1657982	-0.6170834	0.2403174
Most	Views on nonorganization content that is hateful	-23	-0.0445463	0.0842178	-0.2623064	0.1732138
Most	Views on nonorganization content that is hateful	-22	0.1057343	0.0903264	-0.1278205	0.3392892
Most	Views on nonorganization content that is hateful	-21	-0.2191514	0.2899441	-0.9688530	0.5305503
Most	Views on nonorganization content that is hateful	-20	-0.0589928	0.3479434	-0.9586619	0.8406763
Most	Views on nonorganization content that is hateful	-19	0.5915535	0.3470693	-0.3058555	1.4889625
Most	Views on nonorganization content that is hateful	-18	0.4404884	0.2584833	-0.2278657	1.1088426
Most	Views on nonorganization content that is hateful	-17	0.2694016	0.3061055	-0.5220882	1.0608914
Most	Views on nonorganization content that is hateful	-16	0.7743452	0.5058842	-0.5337078	2.0823982
Most	Views on nonorganization content that is hateful	-15	-0.5061720	0.6706896	-2.2403583	1.2280143
Most	Views on nonorganization content that is hateful	-14	0.0941421	0.3040536	-0.6920421	0.8803262
Most	Views on nonorganization content that is hateful	-13	-0.1279382	0.3768956	-1.1024684	0.8465920
Most	Views on nonorganization content that is hateful	-12	-0.1725431	0.1855330	-0.6522713	0.3071851
Most	Views on nonorganization content that is hateful	-11	0.0393975	0.1344097	-0.3081425	0.3869374
Most	Views on nonorganization content that is hateful	-10	0.0479369	0.1633496	-0.3744322	0.4703061
Most	Views on nonorganization content that is hateful	-9	0.0542722	0.1642256	-0.3703621	0.4789064
Most	Views on nonorganization content that is hateful	-8	0.0141978	0.1182240	-0.2914911	0.3198868
Most	Views on nonorganization content that is hateful	-7	-0.3772058	0.1825200	-0.8491435	0.0947319
Most	Views on nonorganization content that is hateful	-6	-0.1518922	0.1810742	-0.6200915	0.3163070
Most	Views on nonorganization content that is hateful	-5	0.2421932	0.1728182	-0.2046587	0.6890451
Most	Views on nonorganization content that is hateful	-4	-0.3325710	0.1694293	-0.7706603	0.1055184
Most	Views on nonorganization content that is hateful	-3	-0.5789446	0.2547948	-1.2377615	0.0798723
Most	Views on nonorganization content that is hateful	-2	0.3600380	0.1166887	0.0583187	0.6617572
Most	Views on nonorganization content that is hateful	-1	0.1446676	0.2561998	-0.5177823	0.8071175
Most	Views on nonorganization content that is hateful	0	0.1552602	0.2144906	-0.3993432	0.7098636
Most	Views on nonorganization content that is hateful	1	0.6800633	0.6072512	-0.8900919	2.2502184
Most	Views on nonorganization content that is hateful	2	0.5120349	0.2158266	-0.0460229	1.0700928
Most	Views on nonorganization content that is hateful	3	0.7012034	0.2963783	-0.0651349	1.4675417
Most	Views on nonorganization content that is hateful	4	0.5136892	0.4024382	-0.5268859	1.5542643
Most	Views on nonorganization content that is hateful	5	0.1982461	0.5688138	-1.2725225	1.6690146
Most	Views on nonorganization content that is hateful	6	0.3060592	0.4694385	-0.9077568	1.5198753
Most	Views on nonorganization content that is hateful	7	0.4590890	0.4946751	-0.8199807	1.7381586

Most	Views on nonorganization content that is hateful	8	0.4818897	0.4514241	-0.6853469	1.6491263
Most	Views on nonorganization content that is hateful	9	0.2257626	0.5382349	-1.1659387	1.6174638
Most	Views on nonorganization content that is hateful	10	0.2939964	0.6849467	-1.4770541	2.0650470
Most	Views on nonorganization content that is hateful	11	0.2710432	0.7525306	-1.6747573	2.2168437
Most	Views on nonorganization content that is hateful	12	0.3792710	0.6280074	-1.2445528	2.0030949
Most	Views on nonorganization content that is hateful	13	0.1606355	0.7755518	-1.8446904	2.1659614
Most	Views on nonorganization content that is hateful	14	0.1822092	1.3115221	-3.2089627	3.5733811
Most	Views on nonorganization content that is hateful	15	0.1478798	1.3830817	-3.4283220	3.7240817
Most	Views on nonorganization content that is hateful	16	0.3332364	1.6118573	-3.8345051	4.5009778
Most	Views on nonorganization content that is hateful	17	0.2848870	1.7568783	-4.2578320	4.8276059
Most	Views on nonorganization content that is hateful	18	0.3747267	1.5289242	-3.5785766	4.3280301
Most	Views on nonorganization content that is hateful	19	0.4072208	1.4966667	-3.4626749	4.2771166
Most	Views on nonorganization content that is hateful	20	0.3645688	1.8669942	-4.4628742	5.1920118
Most	Views on nonorganization content that is hateful	21	0.1954683	1.5063877	-3.6995630	4.0904996
Most	Views on nonorganization content that is hateful	22	0.0049046	1.9080069	-4.9285840	4.9383932
Most	Views on nonorganization content that is hateful	23	0.2731335	1.8022449	-4.3868888	4.9331558
Most	Views on nonorganization content that is hateful	24	0.5940098	1.6090667	-3.5665162	4.7545358
Most	Views on nonorganization content that is hateful	25	0.2065036	1.8739618	-4.6389552	5.0519624
Most	Views on nonorganization content that is hateful	26	0.1376660	1.6089245	-4.0224921	4.2978241
Most	Views on nonorganization content that is hateful	27	0.0489858	1.3987990	-3.5678559	3.6658275
Most	Views on nonorganization content that is hateful	28	-0.0157535	1.5489783	-4.0209102	3.9894033
Most	Views on nonorganization content that is hateful	29	-0.2482916	1.2868481	-3.5756645	3.0790813
Most	Views on nonorganization content that is hateful	30	-0.1619871	1.1655847	-3.1758121	2.8518380

sample	outcome	event.time	estimate	std.error	conf.low	conf.high
Middle	Views on nonorganization content that is hateful	-30	-0.1475377	0.1109559	-0.4297490	0.1346736
Middle	Views on nonorganization content that is hateful	-29	0.2333778	0.0420269	0.1264843	0.3402712
Middle	Views on nonorganization content that is hateful	-28	-0.0399658	0.0828631	-0.2507243	0.1707927
Middle	Views on nonorganization content that is hateful	-27	-0.0101336	0.0718722	-0.1929373	0.1726702
Middle	Views on nonorganization content that is hateful	-26	0.0179485	0.0870961	-0.2035764	0.2394735
Middle	Views on nonorganization content that is hateful	-25	-0.0532235	0.1066183	-0.3244024	0.2179554
Middle	Views on nonorganization content that is hateful	-24	-0.0182858	0.0679272	-0.1910557	0.1544841
Middle	Views on nonorganization content that is hateful	-23	-0.1297985	0.0492353	-0.2550262	-0.0045708
Middle	Views on nonorganization content that is hateful	-22	-0.0506312	0.0908485	-0.2817003	0.1804380
Middle	Views on nonorganization content that is hateful	-21	0.0770366	0.1814232	-0.3844051	0.5384783
Middle	Views on nonorganization content that is hateful	-20	0.0248391	0.2196523	-0.5338367	0.5835149
Middle	Views on nonorganization content that is hateful	-19	0.5275706	0.2520973	-0.1136277	1.1687689
Middle	Views on nonorganization content that is hateful	-18	0.0516400	0.2056769	-0.4714901	0.5747700
Middle	Views on nonorganization content that is hateful	-17	0.5171435	0.1808180	0.0572409	0.9770461
Middle	Views on nonorganization content that is hateful	-16	0.0603252	0.3038849	-0.7125925	0.8332430
Middle	Views on nonorganization content that is hateful	-15	-0.0200755	0.1195904	-0.3242484	0.2840974

Middle	Views on nonorganization content that is hateful	-14	0.0123148	0.0906336	-0.2182078	0.2428374
Middle	Views on nonorganization content that is hateful	-13	-0.2628014	0.3258414	-1.0915646	0.5659618
Middle	Views on nonorganization content that is hateful	-12	0.0404527	0.2196421	-0.5181971	0.5991025
Middle	Views on nonorganization content that is hateful	-11	-0.1379486	0.1408647	-0.4962318	0.2203346
Middle	Views on nonorganization content that is hateful	-10	0.0629276	0.1644099	-0.3552417	0.4810969
Middle	Views on nonorganization content that is hateful	-9	0.0505991	0.0684859	-0.1235919	0.2247900
Middle	Views on nonorganization content that is hateful	-8	-0.1611536	0.1123564	-0.4469271	0.1246198
Middle	Views on nonorganization content that is hateful	-7	-0.2601859	0.1574949	-0.6607671	0.1403952
Middle	Views on nonorganization content that is hateful	-6	-0.2819594	0.1473424	-0.6567183	0.0927995
Middle	Views on nonorganization content that is hateful	-5	-0.0746925	0.1906844	-0.5596897	0.4103047
Middle	Views on nonorganization content that is hateful	-4	-0.3038959	0.2366365	-0.9057701	0.2979784
Middle	Views on nonorganization content that is hateful	-3	-0.5360246	0.2347253	-1.1330379	0.0609886
Middle	Views on nonorganization content that is hateful	-2	0.2712117	0.0807751	0.0657637	0.4766597
Middle	Views on nonorganization content that is hateful	-1	0.1704004	0.1481535	-0.2064215	0.5472222
Middle	Views on nonorganization content that is hateful	0	0.2396460	0.2112927	-0.2977677	0.7770596
Middle	Views on nonorganization content that is hateful	1	0.4489066	0.2070265	-0.0776560	0.9754691
Middle	Views on nonorganization content that is hateful	2	0.4296075	0.2951722	-0.3211499	1.1803649
Middle	Views on nonorganization content that is hateful	3	0.5641591	0.4401667	-0.5553853	1.6837035
Middle	Views on nonorganization content that is hateful	4	0.6086777	0.5390093	-0.7622685	1.9796240
Middle	Views on nonorganization content that is hateful	5	0.3842555	0.6852172	-1.3585638	2.1270748
Middle	Views on nonorganization content that is hateful	6	0.4441644	0.5295246	-0.9026578	1.7909865
Middle	Views on nonorganization content that is hateful	7	0.5840610	0.5317626	-0.7684535	1.9365756
Middle	Views on nonorganization content that is hateful	8	0.4849032	0.5750820	-0.9777923	1.9475988
Middle	Views on nonorganization content that is hateful	9	0.3930701	0.5601057	-1.0315338	1.8176741
Middle	Views on nonorganization content that is hateful	10	0.6050713	0.7064541	-1.1917632	2.4019059
Middle	Views on nonorganization content that is hateful	11	0.7061871	0.8149021	-1.3664800	2.7788542
Middle	Views on nonorganization content that is hateful	12	0.7594794	0.7924808	-1.2561603	2.7751190
Middle	Views on nonorganization content that is hateful	13	0.4677287	0.9983816	-2.0716104	3.0070678
Middle	Views on nonorganization content that is hateful	14	0.7318661	1.8290554	-3.9202548	5.3839870
Middle	Views on nonorganization content that is hateful	15	0.8468000	1.7816078	-3.6846399	5.3782399
Middle	Views on nonorganization content that is hateful	16	0.8300915	1.9148517	-4.0402484	5.7004314
Middle	Views on nonorganization content that is hateful	17	0.9195231	2.1453258	-4.5370176	6.3760637
Middle	Views on nonorganization content that is hateful	18	0.9967497	2.1558957	-4.4866750	6.4801744
Middle	Views on nonorganization content that is hateful	19	0.6603273	1.8368479	-4.0116135	5.3322681
Middle	Views on nonorganization content that is hateful	20	0.5956069	1.8979183	-4.2316637	5.4228775
Middle	Views on nonorganization content that is hateful	21	0.6844012	2.0823967	-4.6120819	5.9808842
Middle	Views on nonorganization content that is hateful	22	0.5860854	1.9989179	-4.4980732	5.6702441
Middle	Views on nonorganization content that is hateful	23	0.8396041	2.0847222	-4.4627938	6.1420020
Middle	Views on nonorganization content that is hateful	24	0.8904149	2.0985733	-4.4472127	6.2280425
Middle	Views on nonorganization content that is hateful	25	0.8750035	2.1593428	-4.6171887	6.3671957
Middle	Views on nonorganization content that is hateful	26	0.5031085	1.8971718	-4.3222634	5.3284803

Middle	Views on nonorganization content that is hateful	27	-0.3673139	1.4186739	-3.9756478	3.2410200
Middle	Views on nonorganization content that is hateful	28	0.0687846	1.3831692	-3.4492446	3.5868139
Middle	Views on nonorganization content that is hateful	29	-0.1327436	1.4055627	-3.7077298	3.4422426
Middle	Views on nonorganization content that is hateful	30	-0.4941514	1.1846465	-3.5072470	2.5189441

sample	outcome	event.time	estimate	std.error	conf.low	conf.high
Least	Views on nonorganization content that is hateful	-30	-0.0163019	0.0954733	-0.2667615	0.2341577
Least	Views on nonorganization content that is hateful	-29	0.0683391	0.0572021	-0.0817220	0.2184002
Least	Views on nonorganization content that is hateful	-28	-0.0631227	0.0878593	-0.2936082	0.1673628
Least	Views on nonorganization content that is hateful	-27	0.1171065	0.1260115	-0.2134657	0.4476786
Least	Views on nonorganization content that is hateful	-26	-0.0990748	0.1079120	-0.3821656	0.1840160
Least	Views on nonorganization content that is hateful	-25	0.0074552	0.0896105	-0.2276244	0.2425349
Least	Views on nonorganization content that is hateful	-24	-0.0294869	0.0785997	-0.2356814	0.1767076
Least	Views on nonorganization content that is hateful	-23	-0.0228799	0.0461011	-0.1438191	0.0980593
Least	Views on nonorganization content that is hateful	-22	0.1074203	0.0724278	-0.0825832	0.2974237
Least	Views on nonorganization content that is hateful	-21	0.3722235	0.1744306	-0.0853688	0.8298158
Least	Views on nonorganization content that is hateful	-20	-0.0987267	0.1234757	-0.4226465	0.2251931
Least	Views on nonorganization content that is hateful	-19	0.4639484	0.2061863	-0.0769502	1.0048470
Least	Views on nonorganization content that is hateful	-18	0.2388854	0.2301525	-0.3648847	0.8426555
Least	Views on nonorganization content that is hateful	-17	0.2029513	0.1605880	-0.2183270	0.6242296
Least	Views on nonorganization content that is hateful	-16	0.3154414	0.2118166	-0.2402273	0.8711100
Least	Views on nonorganization content that is hateful	-15	-0.1539584	0.1221628	-0.4744339	0.1665172
Least	Views on nonorganization content that is hateful	-14	-0.0212942	0.0564020	-0.1692564	0.1266680
Least	Views on nonorganization content that is hateful	-13	-0.1868938	0.2574792	-0.8623514	0.4885639
Least	Views on nonorganization content that is hateful	-12	-0.1597148	0.1349996	-0.5138659	0.1944362
Least	Views on nonorganization content that is hateful	-11	0.0353593	0.0865651	-0.1917312	0.2624498
Least	Views on nonorganization content that is hateful	-10	-0.1121691	0.1044568	-0.3861957	0.1618575
Least	Views on nonorganization content that is hateful	-9	-0.0901325	0.0918399	-0.3310606	0.1507957
Least	Views on nonorganization content that is hateful	-8	-0.0471133	0.1166615	-0.3531571	0.2589305
Least	Views on nonorganization content that is hateful	-7	-0.0845889	0.0878756	-0.3151172	0.1459394
Least	Views on nonorganization content that is hateful	-6	-0.2006891	0.1321901	-0.5474698	0.1460917
Least	Views on nonorganization content that is hateful	-5	-0.2039879	0.1978317	-0.7229694	0.3149935
Least	Views on nonorganization content that is hateful	-4	-0.1213715	0.1495249	-0.5136273	0.2708843
Least	Views on nonorganization content that is hateful	-3	-0.5432081	0.2728512	-1.2589919	0.1725757
Least	Views on nonorganization content that is hateful	-2	0.1885180	0.0754022	-0.0092884	0.3863243
Least	Views on nonorganization content that is hateful	-1	-0.0067072	0.2519400	-0.6676336	0.6542193
Least	Views on nonorganization content that is hateful	0	0.2876355	0.2444438	-0.3536258	0.9288969
Least	Views on nonorganization content that is hateful	1	0.5534117	0.3064164	-0.2504252	1.3572486
Least	Views on nonorganization content that is hateful	2	0.4193252	0.3752193	-0.5650056	1.4036560
Least	Views on nonorganization content that is hateful	3	0.4654507	0.4206150	-0.6379690	1.5688704
Least	Views on nonorganization content that is hateful	4	0.3367966	0.4967445	-0.9663373	1.6399306

Least	Views on nonorganization content that is hateful	5	0.2709040	0.5505789	-1.1734563	1.7152644
Least	Views on nonorganization content that is hateful	6	0.4289367	0.5568152	-1.0317836	1.8896570
Least	Views on nonorganization content that is hateful	7	0.4193741	0.5688017	-1.0727909	1.9115392
Least	Views on nonorganization content that is hateful	8	0.2601056	0.6019520	-1.3190242	1.8392354
Least	Views on nonorganization content that is hateful	9	0.3288797	0.6231463	-1.3058502	1.9636096
Least	Views on nonorganization content that is hateful	10	0.4568521	0.7257441	-1.4470274	2.3607315
Least	Views on nonorganization content that is hateful	11	0.4377436	0.7350607	-1.4905766	2.3660638
Least	Views on nonorganization content that is hateful	12	0.3792407	0.7184475	-1.5054974	2.2639787
Least	Views on nonorganization content that is hateful	13	0.0553515	0.8194697	-2.0944031	2.2051060
Least	Views on nonorganization content that is hateful	14	0.1496976	1.1789982	-2.9432252	3.2426204
Least	Views on nonorganization content that is hateful	15	0.3506055	1.3184080	-3.1080378	3.8092488
Least	Views on nonorganization content that is hateful	16	0.2533427	1.2906286	-3.1324256	3.6391109
Least	Views on nonorganization content that is hateful	17	0.1891323	1.5646967	-3.9156122	4.2938768
Least	Views on nonorganization content that is hateful	18	0.1934342	1.5495117	-3.8714748	4.2583432
Least	Views on nonorganization content that is hateful	19	0.1712336	1.4420034	-3.6116435	3.9541107
Least	Views on nonorganization content that is hateful	20	-0.0875449	1.2561667	-3.3829077	3.2078178
Least	Views on nonorganization content that is hateful	21	0.1740892	1.3382288	-3.3365510	3.6847295
Least	Views on nonorganization content that is hateful	22	0.1088148	1.4802049	-3.7742781	3.9919077
Least	Views on nonorganization content that is hateful	23	0.2172836	1.4758299	-3.6543323	4.0888995
Least	Views on nonorganization content that is hateful	24	0.3299691	1.4587600	-3.4968665	4.1568046
Least	Views on nonorganization content that is hateful	25	0.2290534	1.4547275	-3.5872037	4.0453104
Least	Views on nonorganization content that is hateful	26	0.0698787	1.3324157	-3.4255116	3.5652691
Least	Views on nonorganization content that is hateful	27	-1.7667599	0.4651217	-2.9869361	-0.5465837
Least	Views on nonorganization content that is hateful	28	-1.7634212	0.5280105	-3.1485768	-0.3782656
Least	Views on nonorganization content that is hateful	29	-1.8784675	0.4924543	-3.1703468	-0.5865883
Least	Views on nonorganization content that is hateful	30	-2.0229359	0.5699227	-3.5180417	-0.5278301

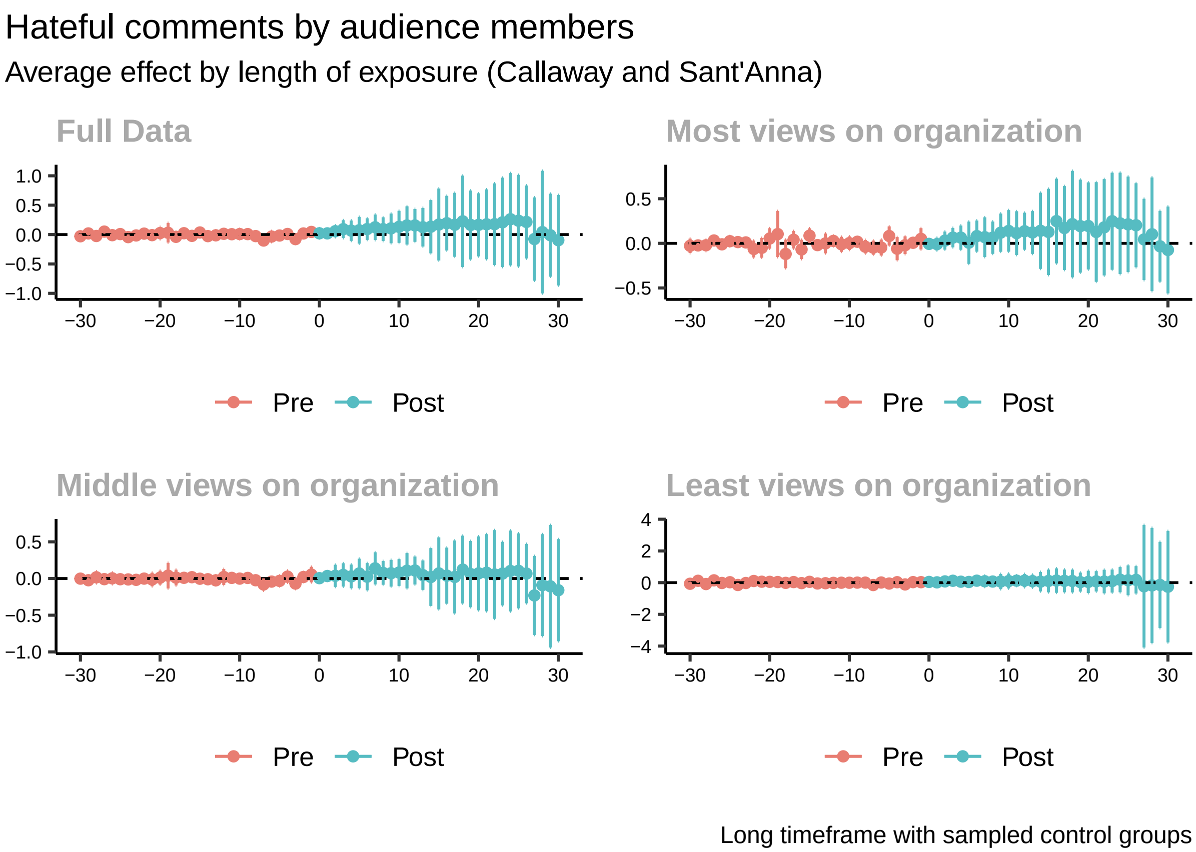

sample	outcome	event.time	estimate	std.error	conf.low	conf.high
Full	Hateful comments by audience members	-30	-0.0287071	0.0177685	-0.0735590	0.0161449
Full	Hateful comments by audience members	-29	0.0191003	0.0124536	-0.0123357	0.0505362
Full	Hateful comments by audience members	-28	-0.0244917	0.0271534	-0.0930334	0.0440501
Full	Hateful comments by audience members	-27	0.0507824	0.0098183	0.0259986	0.0755663
Full	Hateful comments by audience members	-26	-0.0101861	0.0087635	-0.0323074	0.0119352
Full	Hateful comments by audience members	-25	0.0082859	0.0067391	-0.0087253	0.0252971
Full	Hateful comments by audience members	-24	-0.0433262	0.0184741	-0.0899594	0.0033069
Full	Hateful comments by audience members	-23	-0.0148243	0.0086045	-0.0365441	0.0068954
Full	Hateful comments by audience members	-22	0.0162492	0.0180494	-0.0293119	0.0618103
Full	Hateful comments by audience members	-21	-0.0098308	0.0298719	-0.0852346	0.0655730
Full	Hateful comments by audience members	-20	0.0252539	0.0408921	-0.0779677	0.1284755
Full	Hateful comments by audience members	-19	0.0307480	0.0658380	-0.1354430	0.1969391
Full	Hateful comments by audience members	-18	-0.0383322	0.0322754	-0.1198032	0.0431388
Full	Hateful comments by audience members	-17	0.0229209	0.0178181	-0.0220563	0.0678982
Full	Hateful comments by audience members	-16	-0.0221968	0.0272702	-0.0910335	0.0466398
Full	Hateful comments by audience members	-15	0.0353950	0.0169097	-0.0072892	0.0780792
Full	Hateful comments by audience members	-14	-0.0265218	0.0189956	-0.0744713	0.0214276
Full	Hateful comments by audience members	-13	-0.0123253	0.0324016	-0.0941147	0.0694641
Full	Hateful comments by audience members	-12	0.0128530	0.0216121	-0.0417012	0.0674073

Full	Hateful comments by audience members	-11	0.0061927	0.0265581	-0.0608463	0.0732317
Full	Hateful comments by audience members	-10	0.0086169	0.0343728	-0.0781484	0.0953821
Full	Hateful comments by audience members	-9	0.0081232	0.0091479	-0.0149684	0.0312147
Full	Hateful comments by audience members	-8	-0.0262207	0.0236449	-0.0859060	0.0334647
Full	Hateful comments by audience members	-7	-0.1015662	0.0271194	-0.1700220	-0.0331104
Full	Hateful comments by audience members	-6	-0.0336556	0.0366254	-0.1261070	0.0587958
Full	Hateful comments by audience members	-5	-0.0162566	0.0228490	-0.0739331	0.0414198
Full	Hateful comments by audience members	-4	0.0099047	0.0135312	-0.0242514	0.0440607
Full	Hateful comments by audience members	-3	-0.0768241	0.0312112	-0.1556087	0.0019605
Full	Hateful comments by audience members	-2	0.0186861	0.0210323	-0.0344045	0.0717767
Full	Hateful comments by audience members	-1	0.0448886	0.0200579	-0.0057424	0.0955196
Full	Hateful comments by audience members	0	0.0216419	0.0333211	-0.0624687	0.1057525
Full	Hateful comments by audience members	1	0.0207812	0.0271434	-0.0477352	0.0892976
Full	Hateful comments by audience members	2	0.0568348	0.0392141	-0.0421510	0.1558206
Full	Hateful comments by audience members	3	0.0919254	0.0597034	-0.0587804	0.2426312
Full	Hateful comments by audience members	4	0.0667304	0.0683525	-0.1058078	0.2392687
Full	Hateful comments by audience members	5	0.0731291	0.0898149	-0.1535855	0.2998436
Full	Hateful comments by audience members	6	0.0931439	0.0723881	-0.0895812	0.2758690
Full	Hateful comments by audience members	7	0.1243033	0.0859355	-0.0926187	0.3412254
Full	Hateful comments by audience members	8	0.0923749	0.0795050	-0.1083149	0.2930647
Full	Hateful comments by audience members	9	0.1052779	0.0999041	-0.1469043	0.3574600
Full	Hateful comments by audience members	10	0.1318769	0.1076883	-0.1399545	0.4037083
Full	Hateful comments by audience members	11	0.1552190	0.1288725	-0.1700864	0.4805244
Full	Hateful comments by audience members	12	0.1560208	0.1103089	-0.1224256	0.4344671
Full	Hateful comments by audience members	13	0.1246673	0.1300628	-0.2036426	0.4529772
Full	Hateful comments by audience members	14	0.1336572	0.1790704	-0.3183599	0.5856743
Full	Hateful comments by audience members	15	0.1709521	0.2426256	-0.4414936	0.7833978
Full	Hateful comments by audience members	16	0.1936685	0.1842912	-0.2715272	0.6588642
Full	Hateful comments by audience members	17	0.1666569	0.2151474	-0.3764271	0.7097410
Full	Hateful comments by audience members	18	0.2254817	0.3077332	-0.5513114	1.0022747
Full	Hateful comments by audience members	19	0.1634360	0.2322584	-0.4228405	0.7497125
Full	Hateful comments by audience members	20	0.1657864	0.2121142	-0.3696412	0.7012141
Full	Hateful comments by audience members	21	0.1747246	0.2350423	-0.4185790	0.7680283
Full	Hateful comments by audience members	22	0.1770877	0.2745385	-0.5159140	0.8700893
Full	Hateful comments by audience members	23	0.2083545	0.3003999	-0.5499276	0.9666365
Full	Hateful comments by audience members	24	0.2612068	0.3103064	-0.5220817	1.0444954
Full	Hateful comments by audience members	25	0.2358657	0.3086806	-0.5433188	1.0150501
Full	Hateful comments by audience members	26	0.2147203	0.2456823	-0.4054414	0.8348820
Full	Hateful comments by audience members	27	-0.0760719	0.2803238	-0.7836768	0.6315331
Full	Hateful comments by audience members	28	0.0421154	0.4126047	-0.9993986	1.0836294
Full	Hateful comments by audience members	29	-0.0109849	0.2791030	-0.7155083	0.6935386

Full	Hateful comments by audience members	30	-0.0961189	0.3046898	-0.8652296	0.6729917

sample	outcome	event.time	estimate	std.error	conf.low	conf.high
Most	Hateful comments by audience members	-30	-0.0277083	0.0324745	-0.1081090	0.0526923
Most	Hateful comments by audience members	-29	-0.0232667	0.0111034	-0.0507565	0.0042231
Most	Hateful comments by audience members	-28	-0.0214586	0.0257991	-0.0853323	0.0424151
Most	Hateful comments by audience members	-27	0.0320385	0.0198281	-0.0170522	0.0811292
Most	Hateful comments by audience members	-26	-0.0108178	0.0110900	-0.0382746	0.0166390
Most	Hateful comments by audience members	-25	0.0240867	0.0228612	-0.0325133	0.0806867
Most	Hateful comments by audience members	-24	0.0142579	0.0186104	-0.0318179	0.0603337
Most	Hateful comments by audience members	-23	0.0103328	0.0114217	-0.0179452	0.0386108
Most	Hateful comments by audience members	-22	-0.0635733	0.0381642	-0.1580605	0.0309140
Most	Hateful comments by audience members	-21	-0.0502033	0.0443844	-0.1600906	0.0596840
Most	Hateful comments by audience members	-20	0.0546651	0.0459011	-0.0589771	0.1683073
Most	Hateful comments by audience members	-19	0.1040016	0.1039190	-0.1532820	0.3612851
Most	Hateful comments by audience members	-18	-0.1188594	0.0642690	-0.2779772	0.0402584
Most	Hateful comments by audience members	-17	0.0398566	0.0393554	-0.0575798	0.1372930
Most	Hateful comments by audience members	-16	-0.0682166	0.0433840	-0.1756270	0.0391938
Most	Hateful comments by audience members	-15	0.0860026	0.0330165	0.0042601	0.1677450
Most	Hateful comments by audience members	-14	-0.0195148	0.0131358	-0.0520364	0.0130068
Most	Hateful comments by audience members	-13	-0.0006550	0.0445050	-0.1108408	0.1095308
Most	Hateful comments by audience members	-12	0.0271365	0.0235906	-0.0312692	0.0855423
Most	Hateful comments by audience members	-11	-0.0106581	0.0336610	-0.0939963	0.0726800
Most	Hateful comments by audience members	-10	0.0024472	0.0304329	-0.0728988	0.0777932
Most	Hateful comments by audience members	-9	0.0179341	0.0207499	-0.0334388	0.0693069
Most	Hateful comments by audience members	-8	-0.0379326	0.0306238	-0.1137512	0.0378860
Most	Hateful comments by audience members	-7	-0.0480131	0.0319373	-0.1270837	0.0310574
Most	Hateful comments by audience members	-6	-0.0472155	0.0361904	-0.1368160	0.0423849
Most	Hateful comments by audience members	-5	0.0824528	0.0434192	-0.0250448	0.1899503
Most	Hateful comments by audience members	-4	-0.0598006	0.0521476	-0.1889082	0.0693069
Most	Hateful comments by audience members	-3	-0.0208892	0.0401156	-0.1202077	0.0784293
Most	Hateful comments by audience members	-2	0.0063276	0.0214369	-0.0467461	0.0594013
Most	Hateful comments by audience members	-1	0.0500950	0.0480981	-0.0689867	0.1691767
Most	Hateful comments by audience members	0	-0.0061065	0.0196684	-0.0548016	0.0425885
Most	Hateful comments by audience members	1	-0.0089125	0.0289916	-0.0806901	0.0628651
Most	Hateful comments by audience members	2	0.0305133	0.0407559	-0.0703904	0.1314169
Most	Hateful comments by audience members	3	0.0642492	0.0435815	-0.0436503	0.1721487
Most	Hateful comments by audience members	4	0.0639637	0.0546323	-0.0712955	0.1992228
Most	Hateful comments by audience members	5	0.0069177	0.0951830	-0.2287372	0.2425726
Most	Hateful comments by audience members	6	0.0815194	0.0702968	-0.0925220	0.2555607
Most	Hateful comments by audience members	7	0.0693564	0.0896293	-0.1525485	0.2912613

Most	Hateful comments by audience members	8	0.0637484	0.0728950	-0.1167256	0.2442223
Most	Hateful comments by audience members	9	0.1198647	0.0861656	-0.0934647	0.3331941
Most	Hateful comments by audience members	10	0.1392694	0.0938068	-0.0929782	0.3715169
Most	Hateful comments by audience members	11	0.1148299	0.0986074	-0.1293030	0.3589629
Most	Hateful comments by audience members	12	0.1360046	0.0833809	-0.0704305	0.3424396
Most	Hateful comments by audience members	13	0.1215969	0.0964950	-0.1173062	0.3604999
Most	Hateful comments by audience members	14	0.1408256	0.1719983	-0.2850092	0.5666603
Most	Hateful comments by audience members	15	0.1279253	0.1950414	-0.3549597	0.6108103
Most	Hateful comments by audience members	16	0.2495502	0.1916517	-0.2249426	0.7240431
Most	Hateful comments by audience members	17	0.1717438	0.1896366	-0.2977599	0.6412476
Most	Hateful comments by audience members	18	0.2158114	0.2409320	-0.3806900	0.8123129
Most	Hateful comments by audience members	19	0.1928495	0.2103578	-0.3279561	0.7136551
Most	Hateful comments by audience members	20	0.1947501	0.1971712	-0.2934081	0.6829083
Most	Hateful comments by audience members	21	0.1275167	0.2252439	-0.4301439	0.6851773
Most	Hateful comments by audience members	22	0.1794800	0.2180273	-0.3603137	0.7192738
Most	Hateful comments by audience members	23	0.2479975	0.2194720	-0.2953730	0.7913679
Most	Hateful comments by audience members	24	0.2237337	0.2293134	-0.3440023	0.7914697
Most	Hateful comments by audience members	25	0.2129161	0.2159251	-0.3216729	0.7475051
Most	Hateful comments by audience members	26	0.2029611	0.1899667	-0.2673600	0.6732823
Most	Hateful comments by audience members	27	0.0438719	0.1836919	-0.4109140	0.4986578
Most	Hateful comments by audience members	28	0.1018700	0.2571102	-0.5346856	0.7384257
Most	Hateful comments by audience members	29	-0.0329120	0.1600888	-0.4292611	0.3634370
Most	Hateful comments by audience members	30	-0.0744030	0.1961695	-0.5600809	0.4112750

sample	outcome	event.time	estimate	std.error	conf.low	conf.high
Middle	Hateful comments by audience members	-30	-0.0017320	0.0193146	-0.0500812	0.0466173
Middle	Hateful comments by audience members	-29	-0.0236604	0.0162583	-0.0643590	0.0170382
Middle	Hateful comments by audience members	-28	0.0162837	0.0320624	-0.0639764	0.0965439
Middle	Hateful comments by audience members	-27	-0.0087147	0.0147006	-0.0455139	0.0280845
Middle	Hateful comments by audience members	-26	0.0026047	0.0315410	-0.0763501	0.0815595
Middle	Hateful comments by audience members	-25	-0.0096602	0.0100669	-0.0348601	0.0155397
Middle	Hateful comments by audience members	-24	-0.0125167	0.0139906	-0.0475385	0.0225051
Middle	Hateful comments by audience members	-23	-0.0179543	0.0086233	-0.0395405	0.0036320
Middle	Hateful comments by audience members	-22	-0.0013404	0.0136949	-0.0356221	0.0329413
Middle	Hateful comments by audience members	-21	-0.0133287	0.0366063	-0.1049634	0.0783059
Middle	Hateful comments by audience members	-20	0.0110668	0.0377081	-0.0833257	0.1054594
Middle	Hateful comments by audience members	-19	0.0384241	0.0694105	-0.1353276	0.2121757
Middle	Hateful comments by audience members	-18	0.0122371	0.0428002	-0.0949024	0.1193765
Middle	Hateful comments by audience members	-17	0.0094934	0.0145322	-0.0268841	0.0458710
Middle	Hateful comments by audience members	-16	0.0179194	0.0226790	-0.0388516	0.0746904
Middle	Hateful comments by audience members	-15	-0.0028817	0.0242783	-0.0636562	0.0578928

Middle	Hateful comments by audience members	-14	-0.0102001	0.0143903	-0.0462226	0.0258225
Middle	Hateful comments by audience members	-13	-0.0247847	0.0264920	-0.0911007	0.0415313
Middle	Hateful comments by audience members	-12	0.0208939	0.0430311	-0.0868235	0.1286114
Middle	Hateful comments by audience members	-11	0.0085118	0.0240720	-0.0517464	0.0687700
Middle	Hateful comments by audience members	-10	-0.0015088	0.0243162	-0.0623781	0.0593606
Middle	Hateful comments by audience members	-9	0.0075804	0.0130032	-0.0249697	0.0401306
Middle	Hateful comments by audience members	-8	-0.0235523	0.0165073	-0.0648741	0.0177695
Middle	Hateful comments by audience members	-7	-0.0811083	0.0334308	-0.1647937	0.0025771
Middle	Hateful comments by audience members	-6	-0.0420818	0.0244717	-0.1033404	0.0191768
Middle	Hateful comments by audience members	-5	-0.0326073	0.0288640	-0.1048609	0.0396462
Middle	Hateful comments by audience members	-4	0.0255481	0.0324768	-0.0557492	0.1068455
Middle	Hateful comments by audience members	-3	-0.0688348	0.0292038	-0.1419392	0.0042696
Middle	Hateful comments by audience members	-2	0.0205864	0.0263793	-0.0454474	0.0866203
Middle	Hateful comments by audience members	-1	0.0536105	0.0417884	-0.0509961	0.1582171
Middle	Hateful comments by audience members	0	0.0048145	0.0224616	-0.0514124	0.0610415
Middle	Hateful comments by audience members	1	0.0339032	0.0252092	-0.0292016	0.0970081
Middle	Hateful comments by audience members	2	0.0366104	0.0597768	-0.1130257	0.1862465
Middle	Hateful comments by audience members	3	0.0485992	0.0630755	-0.1092943	0.2064927
Middle	Hateful comments by audience members	4	0.0290935	0.0640459	-0.1312292	0.1894162
Middle	Hateful comments by audience members	5	0.0687521	0.0798769	-0.1311993	0.2687036
Middle	Hateful comments by audience members	6	0.0248647	0.0736430	-0.1594818	0.2092111
Middle	Hateful comments by audience members	7	0.1384139	0.0872484	-0.0799904	0.3568181
Middle	Hateful comments by audience members	8	0.0764310	0.0642465	-0.0843939	0.2372558
Middle	Hateful comments by audience members	9	0.0715747	0.0726181	-0.1102063	0.2533557
Middle	Hateful comments by audience members	10	0.0812976	0.0725956	-0.1004272	0.2630223
Middle	Hateful comments by audience members	11	0.1056563	0.0959234	-0.1344636	0.3457762
Middle	Hateful comments by audience members	12	0.1090075	0.0762789	-0.0819373	0.2999523
Middle	Hateful comments by audience members	13	0.0460447	0.0799639	-0.1541246	0.2462140
Middle	Hateful comments by audience members	14	0.0204340	0.1569259	-0.3723902	0.4132583
Middle	Hateful comments by audience members	15	0.0698950	0.1959046	-0.4205026	0.5602925
Middle	Hateful comments by audience members	16	0.0390811	0.1525876	-0.3428833	0.4210455
Middle	Hateful comments by audience members	17	0.0212998	0.1985546	-0.4757312	0.5183307
Middle	Hateful comments by audience members	18	0.1211909	0.1844440	-0.3405180	0.5828997
Middle	Hateful comments by audience members	19	0.0602951	0.1799557	-0.3901783	0.5107686
Middle	Hateful comments by audience members	20	0.0706683	0.2007932	-0.4319665	0.5733030
Middle	Hateful comments by audience members	21	0.0789000	0.2094563	-0.4454206	0.6032207
Middle	Hateful comments by audience members	22	0.0550132	0.2408026	-0.5477751	0.6578014
Middle	Hateful comments by audience members	23	0.0669339	0.1725669	-0.3650435	0.4989114
Middle	Hateful comments by audience members	24	0.1027115	0.2197312	-0.4473299	0.6527529
Middle	Hateful comments by audience members	25	0.1048040	0.2035764	-0.4047979	0.6144059
Middle	Hateful comments by audience members	26	0.0671337	0.1613004	-0.3366408	0.4709083

Middle	Hateful comments by audience members	27	-0.2307822	0.2143952	-0.7674662	0.3059019
Middle	Hateful comments by audience members	28	-0.0903263	0.2768552	-0.7833630	0.6027104
Middle	Hateful comments by audience members	29	-0.1060431	0.3331775	-0.9400683	0.7279822
Middle	Hateful comments by audience members	30	-0.1592480	0.2774428	-0.8537557	0.5352596

sample	outcome	event.time	estimate	std.error	conf.low	conf.high
Least	Hateful comments by audience members	-30	-0.0699349	0.0356687	-0.1573901	0.0175204
Least	Hateful comments by audience members	-29	0.1168941	0.0318554	0.0387886	0.1949997
Least	Hateful comments by audience members	-28	-0.0883992	0.0460658	-0.2013467	0.0245483
Least	Hateful comments by audience members	-27	0.1456597	0.0347701	0.0604078	0.2309116
Least	Hateful comments by audience members	-26	-0.0220879	0.0467593	-0.1367358	0.0925600
Least	Hateful comments by audience members	-25	0.0263188	0.0304066	-0.0482342	0.1008719
Least	Hateful comments by audience members	-24	-0.1479596	0.0513044	-0.2737517	-0.0221676
Least	Hateful comments by audience members	-23	-0.0229368	0.0129618	-0.0547175	0.0088438
Least	Hateful comments by audience members	-22	0.1036772	0.0295926	0.0311197	0.1762346
Least	Hateful comments by audience members	-21	0.0583097	0.0584618	-0.0850312	0.2016507
Least	Hateful comments by audience members	-20	0.0499480	0.0564601	-0.0884850	0.1883811
Least	Hateful comments by audience members	-19	0.0379298	0.0822543	-0.1637475	0.2396072
Least	Hateful comments by audience members	-18	-0.0145824	0.0464351	-0.1284355	0.0992706
Least	Hateful comments by audience members	-17	0.0355714	0.0292094	-0.0360463	0.1071892
Least	Hateful comments by audience members	-16	-0.0296612	0.0337477	-0.1124065	0.0530841
Least	Hateful comments by audience members	-15	0.0505695	0.0303465	-0.0238364	0.1249754
Least	Hateful comments by audience members	-14	-0.0517874	0.0428458	-0.1568401	0.0532652
Least	Hateful comments by audience members	-13	-0.0358861	0.0643034	-0.1935501	0.1217778
Least	Hateful comments by audience members	-12	-0.0152889	0.0399522	-0.1132466	0.0826689
Least	Hateful comments by audience members	-11	-0.0050195	0.0363034	-0.0940308	0.0839918
Least	Hateful comments by audience members	-10	-0.0061476	0.0552292	-0.1415627	0.1292675
Least	Hateful comments by audience members	-9	0.0021631	0.0347286	-0.0829870	0.0873133
Least	Hateful comments by audience members	-8	0.0023923	0.0437104	-0.1047801	0.1095647
Least	Hateful comments by audience members	-7	-0.1518512	0.0507530	-0.2762912	-0.0274113
Least	Hateful comments by audience members	-6	0.0086645	0.0629532	-0.1456889	0.1630179
Least	Hateful comments by audience members	-5	-0.0565600	0.0378009	-0.1492430	0.0361230
Least	Hateful comments by audience members	-4	0.0276373	0.0555376	-0.1085340	0.1638086
Least	Hateful comments by audience members	-3	-0.1126025	0.0792720	-0.3069675	0.0817625
Least	Hateful comments by audience members	-2	0.0319188	0.0851180	-0.1767798	0.2406174
Least	Hateful comments by audience members	-1	0.0294951	0.0581077	-0.1129776	0.1719678
Least	Hateful comments by audience members	0	0.0440451	0.1096082	-0.2247004	0.3127907
Least	Hateful comments by audience members	1	0.0188539	0.0520661	-0.1088057	0.1465135
Least	Hateful comments by audience members	2	0.0771801	0.0841559	-0.1291596	0.2835198
Least	Hateful comments by audience members	3	0.1184143	0.1167900	-0.1679403	0.4047689
Least	Hateful comments by audience members	4	0.0596463	0.1060214	-0.2003050	0.3195975

Least	Hateful comments by audience members	5	0.0422227	0.1074315	-0.2211860	0.3056314
Least	Hateful comments by audience members	6	0.1236375	0.1129729	-0.1533579	0.4006329
Least	Hateful comments by audience members	7	0.0888043	0.1425686	-0.2607562	0.4383648
Least	Hateful comments by audience members	8	0.0692034	0.1083298	-0.1964079	0.3348146
Least	Hateful comments by audience members	9	0.0674819	0.1936533	-0.4073318	0.5422955
Least	Hateful comments by audience members	10	0.1000250	0.1907511	-0.3676729	0.5677229
Least	Hateful comments by audience members	11	0.1239944	0.1339904	-0.2045333	0.4525220
Least	Hateful comments by audience members	12	0.1290091	0.1689422	-0.2852160	0.5432342
Least	Hateful comments by audience members	13	0.0952686	0.1647930	-0.3087832	0.4993204
Least	Hateful comments by audience members	14	0.0619542	0.2488448	-0.5481821	0.6720906
Least	Hateful comments by audience members	15	0.1064031	0.2904604	-0.6057694	0.8185757
Least	Hateful comments by audience members	16	0.1318744	0.3123090	-0.6338683	0.8976172
Least	Hateful comments by audience members	17	0.1108376	0.2918728	-0.6047980	0.8264731
Least	Hateful comments by audience members	18	0.1024873	0.2915173	-0.6122768	0.8172513
Least	Hateful comments by audience members	19	0.0336135	0.2430936	-0.5624217	0.6296487
Least	Hateful comments by audience members	20	0.0452523	0.2859456	-0.6558507	0.7463552
Least	Hateful comments by audience members	21	0.0812413	0.2609017	-0.5584570	0.7209397
Least	Hateful comments by audience members	22	0.0688333	0.3015822	-0.6706087	0.8082752
Least	Hateful comments by audience members	23	0.0996324	0.2957916	-0.6256118	0.8248765
Least	Hateful comments by audience members	24	0.1870722	0.3224217	-0.6034654	0.9776099
Least	Hateful comments by audience members	25	0.1495315	0.3828962	-0.7892823	1.0883453
Least	Hateful comments by audience members	26	0.1745092	0.3511203	-0.6863941	1.0354125
Least	Hateful comments by audience members	27	-0.2302368	1.5747329	-4.0912853	3.6308116
Least	Hateful comments by audience members	28	-0.1750829	1.4781781	-3.7993913	3.4492255
Least	Hateful comments by audience members	29	-0.1449851	1.1063871	-2.8577082	2.5677380
Least	Hateful comments by audience members	30	-0.2575925	1.4306289	-3.7653160	3.2501309

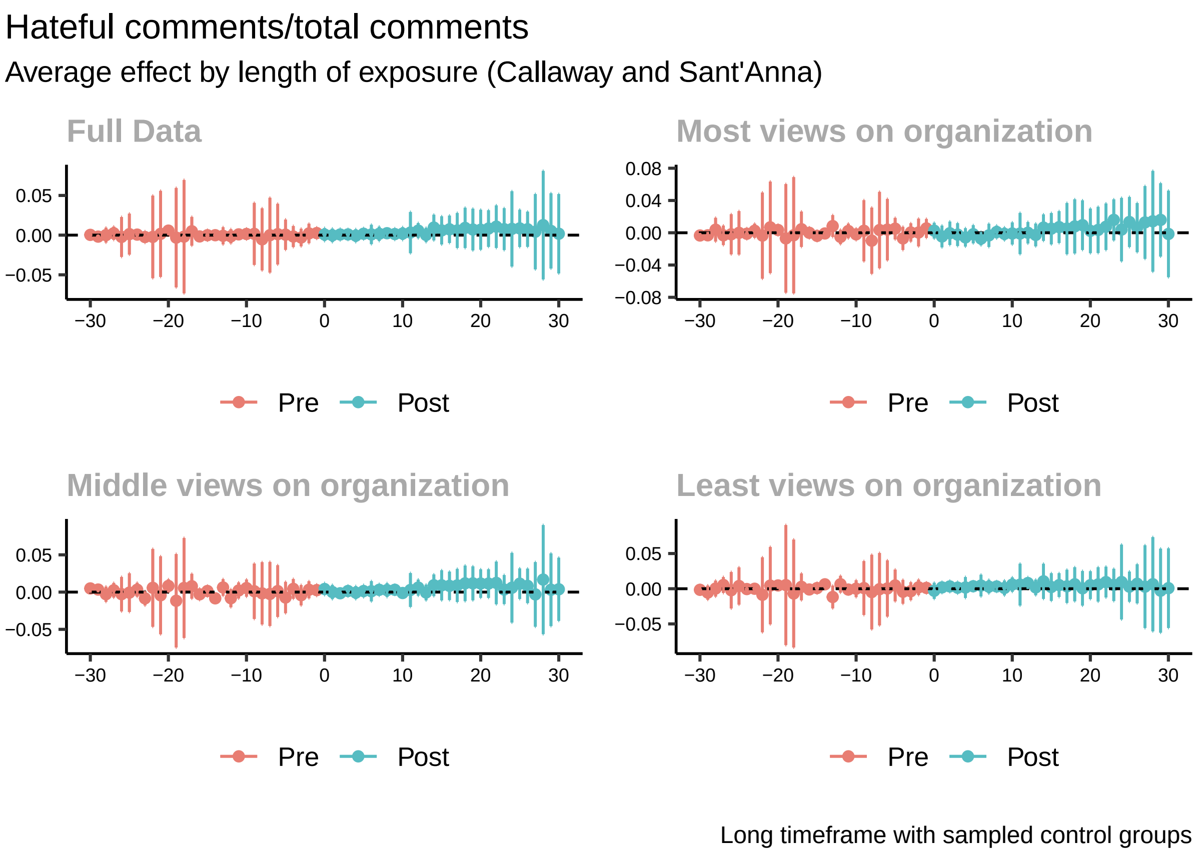

sample	outcome	event.time	estimate	std.error	conf.low	conf.high
Full	Hateful comments/total comments	-30	0.0003424	0.0020553	-0.0045954	0.0052801
Full	Hateful comments/total comments	-29	-0.0018527	0.0010953	-0.0044840	0.0007786
Full	Hateful comments/total comments	-28	-0.0000078	0.0039156	-0.0094148	0.0093992
Full	Hateful comments/total comments	-27	0.0023865	0.0034898	-0.0059976	0.0107707
Full	Hateful comments/total comments	-26	-0.0022388	0.0104475	-0.0273384	0.0228607
Full	Hateful comments/total comments	-25	0.0012780	0.0107344	-0.0245109	0.0270669
Full	Hateful comments/total comments	-24	0.0006589	0.0014413	-0.0028037	0.0041216
Full	Hateful comments/total comments	-23	-0.0023817	0.0030220	-0.0096418	0.0048784
Full	Hateful comments/total comments	-22	-0.0022161	0.0216471	-0.0542222	0.0497899
Full	Hateful comments/total comments	-21	0.0016137	0.0225585	-0.0525819	0.0558093
Full	Hateful comments/total comments	-20	0.0056549	0.0024769	-0.0002957	0.0116055
Full	Hateful comments/total comments	-19	-0.0031333	0.0260716	-0.0657689	0.0595022
Full	Hateful comments/total comments	-18	-0.0018800	0.0297309	-0.0733068	0.0695469
Full	Hateful comments/total comments	-17	0.0048652	0.0075405	-0.0132505	0.0229809
Full	Hateful comments/total comments	-16	-0.0016198	0.0023217	-0.0071977	0.0039580
Full	Hateful comments/total comments	-15	-0.0000961	0.0028079	-0.0068420	0.0066498
Full	Hateful comments/total comments	-14	-0.0000824	0.0016746	-0.0041055	0.0039407
Full	Hateful comments/total comments	-13	-0.0008633	0.0045058	-0.0116882	0.0099616
Full	Hateful comments/total comments	-12	-0.0014771	0.0037466	-0.0104782	0.0075240

Full	Hateful comments/total comments	-11	0.0009781	0.0012179	-0.0019478	0.0039039
Full	Hateful comments/total comments	-10	0.0014610	0.0028574	-0.0054037	0.0083257
Full	Hateful comments/total comments	-9	0.0016213	0.0162551	-0.0374306	0.0406733
Full	Hateful comments/total comments	-8	-0.0052898	0.0162686	-0.0443743	0.0337946
Full	Hateful comments/total comments	-7	-0.0000661	0.0196283	-0.0472221	0.0470900
Full	Hateful comments/total comments	-6	0.0012301	0.0159204	-0.0370179	0.0394781
Full	Hateful comments/total comments	-5	0.0006052	0.0079449	-0.0184820	0.0196923
Full	Hateful comments/total comments	-4	-0.0017670	0.0055343	-0.0150628	0.0115288
Full	Hateful comments/total comments	-3	-0.0030160	0.0045853	-0.0140321	0.0080000
Full	Hateful comments/total comments	-2	0.0021292	0.0051092	-0.0101453	0.0144037
Full	Hateful comments/total comments	-1	0.0025844	0.0029808	-0.0045767	0.0097455
Full	Hateful comments/total comments	0	0.0007669	0.0034398	-0.0074971	0.0090309
Full	Hateful comments/total comments	1	-0.0002335	0.0035465	-0.0087537	0.0082867
Full	Hateful comments/total comments	2	0.0007830	0.0030406	-0.0065218	0.0080879
Full	Hateful comments/total comments	3	0.0009026	0.0028507	-0.0059461	0.0077513
Full	Hateful comments/total comments	4	-0.0006324	0.0033899	-0.0087764	0.0075115
Full	Hateful comments/total comments	5	0.0017353	0.0029753	-0.0054127	0.0088833
Full	Hateful comments/total comments	6	0.0008713	0.0050677	-0.0113035	0.0130462
Full	Hateful comments/total comments	7	0.0016649	0.0037279	-0.0072913	0.0106210
Full	Hateful comments/total comments	8	0.0024062	0.0023041	-0.0031293	0.0079417
Full	Hateful comments/total comments	9	0.0013252	0.0030286	-0.0059509	0.0086014
Full	Hateful comments/total comments	10	0.0019476	0.0033597	-0.0061239	0.0100191
Full	Hateful comments/total comments	11	0.0032422	0.0108104	-0.0227292	0.0292136
Full	Hateful comments/total comments	12	0.0056949	0.0039727	-0.0038494	0.0152391
Full	Hateful comments/total comments	13	0.0002702	0.0037561	-0.0087536	0.0092940
Full	Hateful comments/total comments	14	0.0095352	0.0067334	-0.0066414	0.0257118
Full	Hateful comments/total comments	15	0.0059506	0.0073833	-0.0117874	0.0236886
Full	Hateful comments/total comments	16	0.0073616	0.0071899	-0.0099117	0.0246349
Full	Hateful comments/total comments	17	0.0059086	0.0091894	-0.0161684	0.0279856
Full	Hateful comments/total comments	18	0.0091144	0.0105640	-0.0162651	0.0344938
Full	Hateful comments/total comments	19	0.0070402	0.0109154	-0.0191834	0.0332639
Full	Hateful comments/total comments	20	0.0067979	0.0104566	-0.0183235	0.0319194
Full	Hateful comments/total comments	21	0.0082926	0.0095820	-0.0147276	0.0313128
Full	Hateful comments/total comments	22	0.0106925	0.0111520	-0.0160995	0.0374846
Full	Hateful comments/total comments	23	0.0075548	0.0109796	-0.0188232	0.0339327
Full	Hateful comments/total comments	24	0.0078519	0.0197403	-0.0395731	0.0552769
Full	Hateful comments/total comments	25	0.0084992	0.0097674	-0.0149666	0.0319649
Full	Hateful comments/total comments	26	0.0074213	0.0092047	-0.0146926	0.0295352
Full	Hateful comments/total comments	27	0.0043628	0.0197348	-0.0430490	0.0517747
Full	Hateful comments/total comments	28	0.0126260	0.0284505	-0.0557249	0.0809769
Full	Hateful comments/total comments	29	0.0053105	0.0197396	-0.0421128	0.0527337

Full	Hateful comments/total comments	30	0.0017730	0.0208079	-0.0482168	0.0517628

sample	outcome	event.time	estimate	std.error	conf.low	conf.high
Most	Hateful comments/total comments	-30	-0.0033783	0.0012696	-0.0065020	-0.0002546
Most	Hateful comments/total comments	-29	-0.0031475	0.0017361	-0.0074190	0.0011239
Most	Hateful comments/total comments	-28	0.0037974	0.0059796	-0.0109148	0.0185095
Most	Hateful comments/total comments	-27	-0.0031909	0.0049101	-0.0152719	0.0088900
Most	Hateful comments/total comments	-26	-0.0018828	0.0100885	-0.0267046	0.0229389
Most	Hateful comments/total comments	-25	-0.0000975	0.0109424	-0.0270203	0.0268253
Most	Hateful comments/total comments	-24	-0.0011811	0.0027494	-0.0079458	0.0055835
Most	Hateful comments/total comments	-23	0.0025002	0.0035727	-0.0062902	0.0112906
Most	Hateful comments/total comments	-22	-0.0034734	0.0217066	-0.0568804	0.0499336
Most	Hateful comments/total comments	-21	0.0067260	0.0230377	-0.0499561	0.0634080
Most	Hateful comments/total comments	-20	0.0034360	0.0026550	-0.0030964	0.0099684
Most	Hateful comments/total comments	-19	-0.0069707	0.0273373	-0.0742314	0.0602901
Most	Hateful comments/total comments	-18	-0.0030768	0.0292498	-0.0750432	0.0688896
Most	Hateful comments/total comments	-17	0.0044034	0.0088932	-0.0174774	0.0262842
Most	Hateful comments/total comments	-16	-0.0000398	0.0027273	-0.0067500	0.0066704
Most	Hateful comments/total comments	-15	-0.0038524	0.0025927	-0.0102315	0.0025268
Most	Hateful comments/total comments	-14	-0.0008650	0.0020557	-0.0059228	0.0041927
Most	Hateful comments/total comments	-13	0.0082772	0.0055005	-0.0052562	0.0218105
Most	Hateful comments/total comments	-12	-0.0051419	0.0036515	-0.0141262	0.0038423
Most	Hateful comments/total comments	-11	0.0021955	0.0037172	-0.0069502	0.0113413
Most	Hateful comments/total comments	-10	-0.0019334	0.0029604	-0.0092171	0.0053503
Most	Hateful comments/total comments	-9	0.0024682	0.0153789	-0.0353701	0.0403065
Most	Hateful comments/total comments	-8	-0.0097924	0.0166472	-0.0507513	0.0311664
Most	Hateful comments/total comments	-7	0.0033814	0.0192221	-0.0439128	0.0506755
Most	Hateful comments/total comments	-6	0.0038899	0.0154608	-0.0341499	0.0419296
Most	Hateful comments/total comments	-5	0.0050687	0.0054341	-0.0083013	0.0184386
Most	Hateful comments/total comments	-4	-0.0070129	0.0058401	-0.0213818	0.0073560
Most	Hateful comments/total comments	-3	0.0003938	0.0046138	-0.0109580	0.0117455
Most	Hateful comments/total comments	-2	0.0003367	0.0069798	-0.0168364	0.0175098
Most	Hateful comments/total comments	-1	0.0054283	0.0047733	-0.0063159	0.0171725
Most	Hateful comments/total comments	0	0.0025770	0.0039745	-0.0072019	0.0123559
Most	Hateful comments/total comments	1	-0.0045891	0.0054642	-0.0180332	0.0088549
Most	Hateful comments/total comments	2	-0.0006084	0.0059038	-0.0151341	0.0139174
Most	Hateful comments/total comments	3	-0.0022961	0.0057507	-0.0164450	0.0118529
Most	Hateful comments/total comments	4	-0.0057536	0.0046200	-0.0171206	0.0056134
Most	Hateful comments/total comments	5	-0.0018293	0.0045611	-0.0130513	0.0093927
Most	Hateful comments/total comments	6	-0.0072071	0.0033907	-0.0155497	0.0011355
Most	Hateful comments/total comments	7	-0.0032522	0.0058402	-0.0176215	0.0111172

Most	Hateful comments/total comments	8	0.0009397	0.0032772	-0.0071235	0.0090029
Most	Hateful comments/total comments	9	-0.0013675	0.0032730	-0.0094205	0.0066856
Most	Hateful comments/total comments	10	-0.0008859	0.0055788	-0.0146119	0.0128402
Most	Hateful comments/total comments	11	-0.0009358	0.0103865	-0.0264908	0.0246193
Most	Hateful comments/total comments	12	0.0000231	0.0054283	-0.0133327	0.0133790
Most	Hateful comments/total comments	13	-0.0026792	0.0057230	-0.0167601	0.0114018
Most	Hateful comments/total comments	14	0.0064005	0.0066519	-0.0099658	0.0227669
Most	Hateful comments/total comments	15	0.0050974	0.0078922	-0.0143206	0.0245155
Most	Hateful comments/total comments	16	0.0070180	0.0080158	-0.0127041	0.0267402
Most	Hateful comments/total comments	17	0.0052202	0.0128903	-0.0264952	0.0369356
Most	Hateful comments/total comments	18	0.0078678	0.0136408	-0.0256941	0.0414297
Most	Hateful comments/total comments	19	0.0094459	0.0124796	-0.0212588	0.0401507
Most	Hateful comments/total comments	20	0.0023873	0.0112320	-0.0252480	0.0300225
Most	Hateful comments/total comments	21	0.0035172	0.0116530	-0.0251538	0.0321883
Most	Hateful comments/total comments	22	0.0074736	0.0117281	-0.0213823	0.0363296
Most	Hateful comments/total comments	23	0.0159693	0.0103381	-0.0094665	0.0414051
Most	Hateful comments/total comments	24	0.0039498	0.0159578	-0.0353127	0.0432124
Most	Hateful comments/total comments	25	0.0133387	0.0126209	-0.0177139	0.0443913
Most	Hateful comments/total comments	26	0.0063980	0.0123825	-0.0240680	0.0368641
Most	Hateful comments/total comments	27	0.0128037	0.0183171	-0.0322637	0.0578712
Most	Hateful comments/total comments	28	0.0142458	0.0253868	-0.0482159	0.0767075
Most	Hateful comments/total comments	29	0.0159176	0.0184671	-0.0295190	0.0613542
Most	Hateful comments/total comments	30	-0.0014651	0.0217720	-0.0550331	0.0521029

sample	outcome	event.time	estimate	std.error	conf.low	conf.high
Middle	Hateful comments/total comments	-30	0.0049144	0.0025562	-0.0009791	0.0108079
Middle	Hateful comments/total comments	-29	0.0034186	0.0016545	-0.0003960	0.0072331
Middle	Hateful comments/total comments	-28	-0.0027860	0.0044421	-0.0130274	0.0074553
Middle	Hateful comments/total comments	-27	0.0024673	0.0042742	-0.0073870	0.0123216
Middle	Hateful comments/total comments	-26	-0.0027605	0.0100166	-0.0258541	0.0203332
Middle	Hateful comments/total comments	-25	-0.0007252	0.0112281	-0.0266120	0.0251615
Middle	Hateful comments/total comments	-24	0.0031775	0.0040573	-0.0061767	0.0125317
Middle	Hateful comments/total comments	-23	-0.0083575	0.0042001	-0.0180409	0.0013260
Middle	Hateful comments/total comments	-22	0.0057189	0.0226483	-0.0464973	0.0579351
Middle	Hateful comments/total comments	-21	-0.0042598	0.0226768	-0.0565419	0.0480223
Middle	Hateful comments/total comments	-20	0.0082579	0.0038551	-0.0006301	0.0171459
Middle	Hateful comments/total comments	-19	-0.0116338	0.0272130	-0.0743743	0.0511067
Middle	Hateful comments/total comments	-18	0.0055557	0.0291079	-0.0615534	0.0726648
Middle	Hateful comments/total comments	-17	0.0078817	0.0072330	-0.0087943	0.0245576
Middle	Hateful comments/total comments	-16	-0.0029543	0.0033183	-0.0106048	0.0046962
Middle	Hateful comments/total comments	-15	0.0014511	0.0030965	-0.0056880	0.0085902

Middle	Hateful comments/total comments	-14	-0.0083631	0.0015630	-0.0119666	-0.0047597
Middle	Hateful comments/total comments	-13	0.0061826	0.0048366	-0.0049684	0.0173336
Middle	Hateful comments/total comments	-12	-0.0081997	0.0052563	-0.0203183	0.0039189
Middle	Hateful comments/total comments	-11	0.0021745	0.0047005	-0.0086626	0.0130116
Middle	Hateful comments/total comments	-10	0.0054016	0.0050134	-0.0061568	0.0169601
Middle	Hateful comments/total comments	-9	0.0011664	0.0161245	-0.0360092	0.0383420
Middle	Hateful comments/total comments	-8	-0.0016846	0.0181267	-0.0434761	0.0401070
Middle	Hateful comments/total comments	-7	-0.0024989	0.0186504	-0.0454981	0.0405002
Middle	Hateful comments/total comments	-6	0.0013417	0.0150332	-0.0333179	0.0360013
Middle	Hateful comments/total comments	-5	-0.0072764	0.0091231	-0.0283100	0.0137573
Middle	Hateful comments/total comments	-4	0.0043262	0.0057634	-0.0089616	0.0176140
Middle	Hateful comments/total comments	-3	-0.0041490	0.0059252	-0.0178097	0.0095118
Middle	Hateful comments/total comments	-2	0.0031212	0.0049543	-0.0083011	0.0145435
Middle	Hateful comments/total comments	-1	0.0026250	0.0033364	-0.0050671	0.0103171
Middle	Hateful comments/total comments	0	0.0041099	0.0035094	-0.0039812	0.0122010
Middle	Hateful comments/total comments	1	0.0001100	0.0041350	-0.0094233	0.0096433
Middle	Hateful comments/total comments	2	-0.0015597	0.0025077	-0.0073414	0.0042220
Middle	Hateful comments/total comments	3	0.0014670	0.0031411	-0.0057749	0.0087090
Middle	Hateful comments/total comments	4	-0.0007175	0.0037879	-0.0094506	0.0080156
Middle	Hateful comments/total comments	5	0.0013737	0.0035536	-0.0068191	0.0095665
Middle	Hateful comments/total comments	6	0.0011229	0.0058861	-0.0124477	0.0146935
Middle	Hateful comments/total comments	7	0.0030636	0.0034094	-0.0047970	0.0109242
Middle	Hateful comments/total comments	8	0.0026775	0.0040314	-0.0066170	0.0119719
Middle	Hateful comments/total comments	9	0.0031089	0.0029132	-0.0036075	0.0098254
Middle	Hateful comments/total comments	10	-0.0012128	0.0024660	-0.0068981	0.0044726
Middle	Hateful comments/total comments	11	0.0028598	0.0098386	-0.0198233	0.0255429
Middle	Hateful comments/total comments	12	0.0060595	0.0047071	-0.0047928	0.0169118
Middle	Hateful comments/total comments	13	-0.0005038	0.0045681	-0.0110358	0.0100281
Middle	Hateful comments/total comments	14	0.0091818	0.0063194	-0.0053879	0.0237514
Middle	Hateful comments/total comments	15	0.0090943	0.0087360	-0.0110469	0.0292355
Middle	Hateful comments/total comments	16	0.0083777	0.0082801	-0.0107124	0.0274678
Middle	Hateful comments/total comments	17	0.0089581	0.0096212	-0.0132239	0.0311401
Middle	Hateful comments/total comments	18	0.0117397	0.0103528	-0.0121289	0.0356083
Middle	Hateful comments/total comments	19	0.0117839	0.0098741	-0.0109810	0.0345489
Middle	Hateful comments/total comments	20	0.0114712	0.0082758	-0.0076089	0.0305512
Middle	Hateful comments/total comments	21	0.0114440	0.0083051	-0.0077036	0.0305917
Middle	Hateful comments/total comments	22	0.0123520	0.0123954	-0.0162260	0.0409300
Middle	Hateful comments/total comments	23	0.0034222	0.0084599	-0.0160824	0.0229268
Middle	Hateful comments/total comments	24	0.0058546	0.0202830	-0.0409084	0.0526176
Middle	Hateful comments/total comments	25	0.0112842	0.0088911	-0.0092146	0.0317830
Middle	Hateful comments/total comments	26	0.0083498	0.0100345	-0.0147851	0.0314848

Middle	Hateful comments/total comments	27	-0.0030809	0.0187913	-0.0464048	0.0402429
Middle	Hateful comments/total comments	28	0.0167782	0.0317398	-0.0563989	0.0899553
Middle	Hateful comments/total comments	29	0.0031599	0.0211621	-0.0456299	0.0519498
Middle	Hateful comments/total comments	30	0.0037906	0.0183496	-0.0385149	0.0460960

sample	outcome	event.time	estimate	std.error	conf.low	conf.high
Least	Hateful comments/total comments	-30	-0.0015522	0.0026674	-0.0082563	0.0051520
Least	Hateful comments/total comments	-29	-0.0054694	0.0040325	-0.0156047	0.0046659
Least	Hateful comments/total comments	-28	0.0002307	0.0045402	-0.0111806	0.0116419
Least	Hateful comments/total comments	-27	0.0053059	0.0045258	-0.0060692	0.0166810
Least	Hateful comments/total comments	-26	-0.0021316	0.0103083	-0.0280405	0.0237774
Least	Hateful comments/total comments	-25	0.0038728	0.0105259	-0.0225829	0.0303285
Least	Hateful comments/total comments	-24	-0.0004862	0.0006681	-0.0021653	0.0011929
Least	Hateful comments/total comments	-23	-0.0000268	0.0016710	-0.0042267	0.0041732
Least	Hateful comments/total comments	-22	-0.0086211	0.0211676	-0.0618237	0.0445815
Least	Hateful comments/total comments	-21	0.0044431	0.0218472	-0.0504675	0.0593537
Least	Hateful comments/total comments	-20	0.0046798	0.0019700	-0.0002716	0.0096311
Least	Hateful comments/total comments	-19	0.0051828	0.0339167	-0.0800634	0.0904290
Least	Hateful comments/total comments	-18	-0.0067876	0.0305284	-0.0835176	0.0699424
Least	Hateful comments/total comments	-17	0.0026729	0.0075866	-0.0163952	0.0217411
Least	Hateful comments/total comments	-16	-0.0010394	0.0023895	-0.0070452	0.0049664
Least	Hateful comments/total comments	-15	0.0009084	0.0030039	-0.0066416	0.0084584
Least	Hateful comments/total comments	-14	0.0062608	0.0023499	0.0003546	0.0121671
Least	Hateful comments/total comments	-13	-0.0117908	0.0063997	-0.0278757	0.0042942
Least	Hateful comments/total comments	-12	0.0063750	0.0047593	-0.0055870	0.0183369
Least	Hateful comments/total comments	-11	-0.0012030	0.0026549	-0.0078757	0.0054697
Least	Hateful comments/total comments	-10	0.0000367	0.0047736	-0.0119613	0.0120346
Least	Hateful comments/total comments	-9	0.0009258	0.0152258	-0.0373428	0.0391945
Least	Hateful comments/total comments	-8	-0.0047434	0.0210672	-0.0576936	0.0482068
Least	Hateful comments/total comments	-7	-0.0006271	0.0204571	-0.0520440	0.0507899
Least	Hateful comments/total comments	-6	0.0003417	0.0158870	-0.0395888	0.0402721
Least	Hateful comments/total comments	-5	0.0046705	0.0089949	-0.0179374	0.0272784
Least	Hateful comments/total comments	-4	-0.0043404	0.0067318	-0.0212600	0.0125792
Least	Hateful comments/total comments	-3	-0.0035113	0.0050407	-0.0161805	0.0091580
Least	Hateful comments/total comments	-2	0.0020329	0.0043947	-0.0090127	0.0130785
Least	Hateful comments/total comments	-1	0.0011980	0.0030593	-0.0064913	0.0088873
Least	Hateful comments/total comments	0	-0.0029234	0.0045476	-0.0143534	0.0085066
Least	Hateful comments/total comments	1	0.0018903	0.0031847	-0.0061142	0.0098947
Least	Hateful comments/total comments	2	0.0032874	0.0032037	-0.0047646	0.0113395
Least	Hateful comments/total comments	3	0.0015005	0.0033563	-0.0069353	0.0099363
Least	Hateful comments/total comments	4	0.0017373	0.0059157	-0.0131313	0.0166060

Least	Hateful comments/total comments	5	0.0035360	0.0027980	-0.0034966	0.0105685
Least	Hateful comments/total comments	6	0.0044708	0.0061440	-0.0109717	0.0199132
Least	Hateful comments/total comments	7	0.0029757	0.0038523	-0.0067065	0.0126580
Least	Hateful comments/total comments	8	0.0031916	0.0029935	-0.0043322	0.0107154
Least	Hateful comments/total comments	9	0.0010836	0.0042584	-0.0096193	0.0117866
Least	Hateful comments/total comments	10	0.0059012	0.0040659	-0.0043180	0.0161203
Least	Hateful comments/total comments	11	0.0058102	0.0118267	-0.0239149	0.0355353
Least	Hateful comments/total comments	12	0.0080608	0.0033393	-0.0003323	0.0164538
Least	Hateful comments/total comments	13	0.0018597	0.0044235	-0.0092583	0.0129776
Least	Hateful comments/total comments	14	0.0105173	0.0099083	-0.0143863	0.0354210
Least	Hateful comments/total comments	15	0.0024181	0.0077938	-0.0171707	0.0220069
Least	Hateful comments/total comments	16	0.0051456	0.0067206	-0.0117459	0.0220370
Least	Hateful comments/total comments	17	0.0034278	0.0093532	-0.0200804	0.0269361
Least	Hateful comments/total comments	18	0.0063356	0.0096046	-0.0178045	0.0304758
Least	Hateful comments/total comments	19	0.0006260	0.0097816	-0.0239592	0.0252112
Least	Hateful comments/total comments	20	0.0050559	0.0078619	-0.0147042	0.0248159
Least	Hateful comments/total comments	21	0.0061184	0.0096975	-0.0182553	0.0304921
Least	Hateful comments/total comments	22	0.0094057	0.0086989	-0.0124582	0.0312697
Least	Hateful comments/total comments	23	0.0053943	0.0091167	-0.0175197	0.0283083
Least	Hateful comments/total comments	24	0.0095337	0.0211305	-0.0435758	0.0626432
Least	Hateful comments/total comments	25	0.0029702	0.0085182	-0.0184396	0.0243799
Least	Hateful comments/total comments	26	0.0070124	0.0110228	-0.0206923	0.0347172
Least	Hateful comments/total comments	27	0.0029123	0.0233858	-0.0558656	0.0616902
Least	Hateful comments/total comments	28	0.0064112	0.0265969	-0.0604375	0.0732599
Least	Hateful comments/total comments	29	-0.0027665	0.0237356	-0.0624235	0.0568905
Least	Hateful comments/total comments	30	0.0007955	0.0224925	-0.0557371	0.0573281

Audience outdegree

Average effect by length of exposure (Callaway and Sant'Anna)

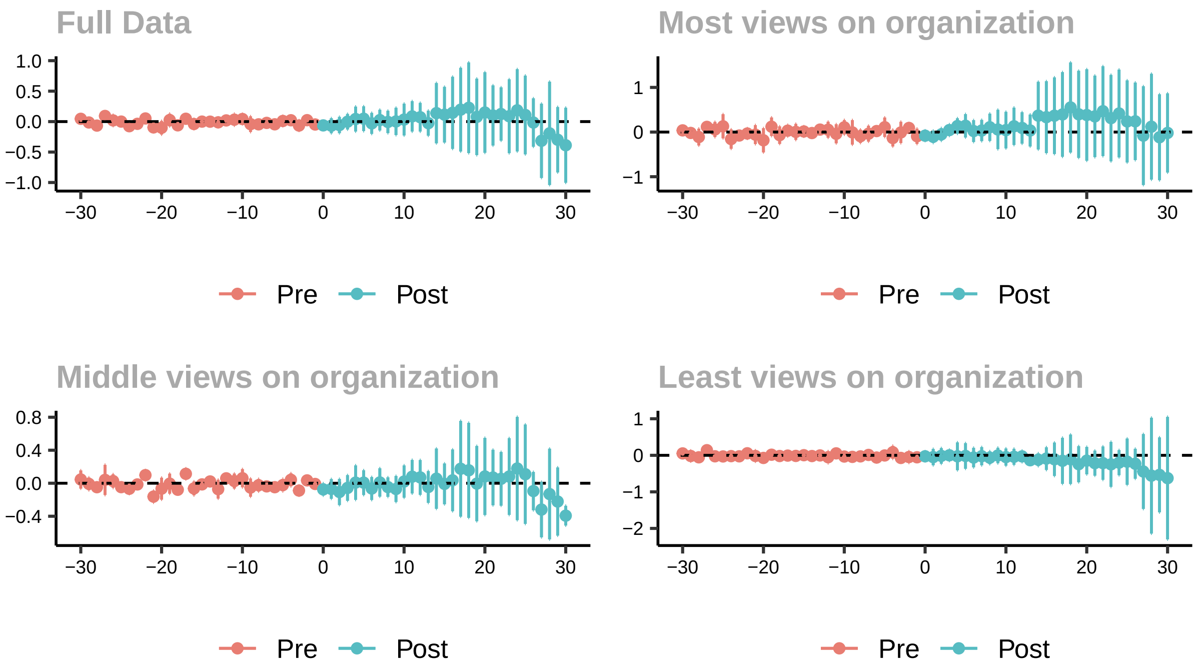

Long timeframe with sampled control groups

sample	outcome	event.time	estimate	std.error	conf.low	conf.high
Full	Audience outdegree	-30	0.0454893	0.0198107	-0.0054302	0.0964088
Full	Audience outdegree	-29	-0.0142792	0.0121135	-0.0454144	0.0168560
Full	Audience outdegree	-28	-0.0657547	0.0233416	-0.1257495	-0.0057599
Full	Audience outdegree	-27	0.0923012	0.0265647	0.0240221	0.1605802
Full	Audience outdegree	-26	0.0193271	0.0354152	-0.0717004	0.1103547
Full	Audience outdegree	-25	-0.0001636	0.0285131	-0.0734507	0.0731236
Full	Audience outdegree	-24	-0.0759758	0.0204334	-0.1284956	-0.0234560
Full	Audience outdegree	-23	-0.0367684	0.0185293	-0.0843942	0.0108574
Full	Audience outdegree	-22	0.0514007	0.0236711	-0.0094411	0.1122424
Full	Audience outdegree	-21	-0.0954589	0.0306626	-0.1742709	-0.0166469
Full	Audience outdegree	-20	-0.1011317	0.0432966	-0.2124167	0.0101534
Full	Audience outdegree	-19	0.0255991	0.0404962	-0.0784881	0.1296863
Full	Audience outdegree	-18	-0.0613446	0.0219741	-0.1178245	-0.0048647
Full	Audience outdegree	-17	0.0463794	0.0156846	0.0060654	0.0866934
Full	Audience outdegree	-16	-0.0399039	0.0322672	-0.1228403	0.0430324
Full	Audience outdegree	-15	-0.0001980	0.0165811	-0.0428162	0.0424203
Full	Audience outdegree	-14	-0.0014071	0.0083108	-0.0227684	0.0199542
Full	Audience outdegree	-13	-0.0121237	0.0308543	-0.0914284	0.0671810
Full	Audience outdegree	-12	0.0193877	0.0154464	-0.0203142	0.0590897

Full	Audience outdegree	-11	0.0310062	0.0380175	-0.0667100	0.1287223
Full	Audience outdegree	-10	0.0422564	0.0328900	-0.0422807	0.1267935
Full	Audience outdegree	-9	-0.0420161	0.0499040	-0.1702842	0.0862520
Full	Audience outdegree	-8	-0.0452061	0.0248394	-0.1090508	0.0186385
Full	Audience outdegree	-7	-0.0215958	0.0187905	-0.0698930	0.0267014
Full	Audience outdegree	-6	-0.0447400	0.0248005	-0.1084846	0.0190046
Full	Audience outdegree	-5	0.0102945	0.0326377	-0.0735940	0.0941829
Full	Audience outdegree	-4	0.0217811	0.0245954	-0.0414364	0.0849985
Full	Audience outdegree	-3	-0.0646509	0.0334383	-0.1505973	0.0212955
Full	Audience outdegree	-2	0.0234508	0.0136630	-0.0116673	0.0585688
Full	Audience outdegree	-1	-0.0477172	0.0295512	-0.1236726	0.0282382
Full	Audience outdegree	0	-0.0624038	0.0265619	-0.1306757	0.0058682
Full	Audience outdegree	1	-0.0677017	0.0478300	-0.1906390	0.0552356
Full	Audience outdegree	2	-0.0567903	0.0520347	-0.1905350	0.0769543
Full	Audience outdegree	3	-0.0021213	0.0497313	-0.1299454	0.1257028
Full	Audience outdegree	4	0.0420534	0.0797840	-0.1630151	0.2471220
Full	Audience outdegree	5	0.0472792	0.0789258	-0.1555833	0.2501418
Full	Audience outdegree	6	-0.0290921	0.0667681	-0.2007060	0.1425218
Full	Audience outdegree	7	0.0244027	0.0659399	-0.1450823	0.1938878
Full	Audience outdegree	8	-0.0059371	0.0714399	-0.1895587	0.1776846
Full	Audience outdegree	9	0.0028585	0.0871811	-0.2212227	0.2269397
Full	Audience outdegree	10	0.0332059	0.1013539	-0.2273036	0.2937153
Full	Audience outdegree	11	0.0855531	0.0969402	-0.1636120	0.3347182
Full	Audience outdegree	12	0.0715055	0.0916798	-0.1641386	0.3071497
Full	Audience outdegree	13	-0.0258076	0.0800727	-0.2316181	0.1800030
Full	Audience outdegree	14	0.1389174	0.1924979	-0.3558592	0.6336939
Full	Audience outdegree	15	0.1133797	0.1778244	-0.3436816	0.5704411
Full	Audience outdegree	16	0.1444892	0.2301507	-0.4470663	0.7360447
Full	Audience outdegree	17	0.1959869	0.2669644	-0.4901907	0.8821646
Full	Audience outdegree	18	0.2269513	0.2896449	-0.5175218	0.9714244
Full	Audience outdegree	19	0.0777383	0.2442162	-0.5499697	0.7054464
Full	Audience outdegree	20	0.1490290	0.2572504	-0.5121806	0.8102387
Full	Audience outdegree	21	0.1004270	0.1921785	-0.3935286	0.5943826
Full	Audience outdegree	22	0.1238578	0.1702188	-0.3136549	0.5613704
Full	Audience outdegree	23	0.0877876	0.2358256	-0.5183541	0.6939293
Full	Audience outdegree	24	0.1852983	0.2623522	-0.4890247	0.8596212
Full	Audience outdegree	25	0.1087450	0.2512588	-0.5370646	0.7545547
Full	Audience outdegree	26	-0.0157994	0.1545046	-0.4129220	0.3813232
Full	Audience outdegree	27	-0.3151135	0.2371603	-0.9246856	0.2944586
Full	Audience outdegree	28	-0.1926860	0.3297605	-1.0402683	0.6548963
Full	Audience outdegree	29	-0.2973863	0.2086363	-0.8336433	0.2388708

Full	Audience outdegree	30	-0.3887757	0.2394701	-1.0042849	0.2267335

sample	outcome	event.time	estimate	std.error	conf.low	conf.high
Most	Audience outdegree	-30	0.0374666	0.0350777	-0.0481753	0.1231085
Most	Audience outdegree	-29	-0.0199617	0.0415948	-0.1215149	0.0815914
Most	Audience outdegree	-28	-0.1112993	0.0770717	-0.2994691	0.0768705
Most	Audience outdegree	-27	0.1159938	0.0330296	0.0353524	0.1966352
Most	Audience outdegree	-26	0.0574997	0.0695826	-0.1123855	0.2273849
Most	Audience outdegree	-25	0.1294529	0.1103560	-0.1399800	0.3988858
Most	Audience outdegree	-24	-0.1573009	0.0885997	-0.3736162	0.0590143
Most	Audience outdegree	-23	-0.0775796	0.0278092	-0.1454753	-0.0096838
Most	Audience outdegree	-22	-0.0329570	0.0476648	-0.1493302	0.0834161
Most	Audience outdegree	-21	-0.0572402	0.0868299	-0.2692345	0.1547541
Most	Audience outdegree	-20	-0.1844609	0.1106836	-0.4546937	0.0857719
Most	Audience outdegree	-19	0.1189369	0.0847671	-0.0880211	0.3258948
Most	Audience outdegree	-18	-0.0691208	0.0800447	-0.2645491	0.1263075
Most	Audience outdegree	-17	0.0308013	0.0529295	-0.0984255	0.1600281
Most	Audience outdegree	-16	0.0038292	0.0730882	-0.1746147	0.1822731
Most	Audience outdegree	-15	0.0133364	0.0468480	-0.1010424	0.1277151
Most	Audience outdegree	-14	-0.0221902	0.0277570	-0.0899586	0.0455782
Most	Audience outdegree	-13	0.0551327	0.0384804	-0.0388167	0.1490822
Most	Audience outdegree	-12	0.0522570	0.0733108	-0.1267305	0.2312446
Most	Audience outdegree	-11	-0.0385962	0.0870713	-0.2511798	0.1739873
Most	Audience outdegree	-10	0.1059086	0.0667287	-0.0570089	0.2688260
Most	Audience outdegree	-9	-0.0074360	0.1122745	-0.2815530	0.2666810
Most	Audience outdegree	-8	-0.0931081	0.0613215	-0.2428239	0.0566076
Most	Audience outdegree	-7	-0.0370810	0.0728424	-0.2149248	0.1407628
Most	Audience outdegree	-6	0.0219846	0.0231949	-0.0346455	0.0786146
Most	Audience outdegree	-5	0.1082253	0.0889239	-0.1088814	0.3253319
Most	Audience outdegree	-4	-0.1342821	0.0768733	-0.3219675	0.0534032
Most	Audience outdegree	-3	-0.0088757	0.0974805	-0.2468733	0.2291219
Most	Audience outdegree	-2	0.0917177	0.0433972	-0.0142359	0.1976714
Most	Audience outdegree	-1	-0.0937300	0.0702139	-0.2651564	0.0776965
Most	Audience outdegree	0	-0.0809421	0.0479636	-0.1980446	0.0361605
Most	Audience outdegree	1	-0.1055935	0.0563340	-0.2431323	0.0319454
Most	Audience outdegree	2	-0.0524753	0.0583986	-0.1950547	0.0901042
Most	Audience outdegree	3	0.0443072	0.0526727	-0.0842925	0.1729069
Most	Audience outdegree	4	0.1345987	0.0708906	-0.0384800	0.3076773
Most	Audience outdegree	5	0.1405000	0.1071038	-0.1209928	0.4019928
Most	Audience outdegree	6	0.0222440	0.1000731	-0.2220834	0.2665713
Most	Audience outdegree	7	0.0341691	0.0976627	-0.2042734	0.2726116

Most	Audience outdegree	8	0.1074787	0.1250214	-0.1977596	0.4127169
Most	Audience outdegree	9	0.0576665	0.1803372	-0.3826249	0.4979578
Most	Audience outdegree	10	0.0439843	0.1671203	-0.3640381	0.4520066
Most	Audience outdegree	11	0.1293982	0.1711318	-0.2884182	0.5472147
Most	Audience outdegree	12	0.0915993	0.1440093	-0.2599979	0.4431965
Most	Audience outdegree	13	0.0350636	0.1464160	-0.3224095	0.3925366
Most	Audience outdegree	14	0.3677709	0.3099829	-0.3890489	1.1245907
Most	Audience outdegree	15	0.3329108	0.3296273	-0.4718706	1.1376922
Most	Audience outdegree	16	0.3664336	0.3508957	-0.4902743	1.2231415
Most	Audience outdegree	17	0.3943415	0.3879518	-0.5528385	1.3415214
Most	Audience outdegree	18	0.5507317	0.4131166	-0.4578880	1.5593514
Most	Audience outdegree	19	0.3972862	0.3993116	-0.5776287	1.3722011
Most	Audience outdegree	20	0.3814577	0.4193658	-0.6424193	1.4053348
Most	Audience outdegree	21	0.3510564	0.3740078	-0.5620794	1.2641923
Most	Audience outdegree	22	0.4670342	0.4120654	-0.5390188	1.4730873
Most	Audience outdegree	23	0.3123455	0.3961544	-0.6548610	1.2795520
Most	Audience outdegree	24	0.4163685	0.4023558	-0.5659788	1.3987159
Most	Audience outdegree	25	0.2380787	0.3763102	-0.6806785	1.1568360
Most	Audience outdegree	26	0.2405029	0.3556708	-0.6278635	1.1088693
Most	Audience outdegree	27	-0.0797681	0.4525214	-1.1845940	1.0250578
Most	Audience outdegree	28	0.1195968	0.4854414	-1.0656030	1.3047967
Most	Audience outdegree	29	-0.1167266	0.3939125	-1.0784598	0.8450065
Most	Audience outdegree	30	-0.0198832	0.3627148	-0.9054472	0.8656809

sample	outcome	event.time	estimate	std.error	conf.low	conf.high
Middle	Audience outdegree	-30	0.0455140	0.0433439	-0.0646295	0.1556575
Middle	Audience outdegree	-29	-0.0075411	0.0303535	-0.0846739	0.0695917
Middle	Audience outdegree	-28	-0.0481477	0.0184742	-0.0950935	-0.0012019
Middle	Audience outdegree	-27	0.0404802	0.0719361	-0.1423203	0.2232807
Middle	Audience outdegree	-26	0.0263188	0.0324713	-0.0561958	0.1088334
Middle	Audience outdegree	-25	-0.0475502	0.0183920	-0.0942870	-0.0008134
Middle	Audience outdegree	-24	-0.0672510	0.0248027	-0.1302785	-0.0042236
Middle	Audience outdegree	-23	-0.0146492	0.0157142	-0.0545813	0.0252829
Middle	Audience outdegree	-22	0.0980264	0.0237898	0.0375728	0.1584799
Middle	Audience outdegree	-21	-0.1621467	0.0284420	-0.2344222	-0.0898713
Middle	Audience outdegree	-20	-0.0656040	0.0520646	-0.1979081	0.0667001
Middle	Audience outdegree	-19	-0.0051644	0.0476588	-0.1262726	0.1159439
Middle	Audience outdegree	-18	-0.0789599	0.0119454	-0.1093149	-0.0486048
Middle	Audience outdegree	-17	0.1139553	0.0258763	0.0481997	0.1797108
Middle	Audience outdegree	-16	-0.0631111	0.0331409	-0.1473272	0.0211049
Middle	Audience outdegree	-15	-0.0140530	0.0142719	-0.0503202	0.0222142

Middle	Audience outdegree	-14	0.0216367	0.0124546	-0.0100123	0.0532857
Middle	Audience outdegree	-13	-0.0718232	0.0447545	-0.1855512	0.0419048
Middle	Audience outdegree	-12	0.0592659	0.0165336	0.0172514	0.1012804
Middle	Audience outdegree	-11	0.0264644	0.0355916	-0.0639791	0.1169080
Middle	Audience outdegree	-10	0.0606023	0.0434056	-0.0496977	0.1709024
Middle	Audience outdegree	-9	-0.0528041	0.0432297	-0.1626573	0.0570491
Middle	Audience outdegree	-8	-0.0245315	0.0299855	-0.1007293	0.0516662
Middle	Audience outdegree	-7	-0.0380233	0.0191827	-0.0867694	0.0107228
Middle	Audience outdegree	-6	-0.0479809	0.0176474	-0.0928258	-0.0031361
Middle	Audience outdegree	-5	-0.0225566	0.0297930	-0.0982652	0.0531519
Middle	Audience outdegree	-4	0.0415555	0.0312286	-0.0378010	0.1209120
Middle	Audience outdegree	-3	-0.0889519	0.0237447	-0.1492907	-0.0286131
Middle	Audience outdegree	-2	0.0343189	0.0187514	-0.0133314	0.0819691
Middle	Audience outdegree	-1	-0.0089071	0.0140324	-0.0445656	0.0267514
Middle	Audience outdegree	0	-0.0749303	0.0293079	-0.1494061	-0.0004545
Middle	Audience outdegree	1	-0.0690060	0.0468588	-0.1880813	0.0500693
Middle	Audience outdegree	2	-0.1063507	0.0622668	-0.2645800	0.0518786
Middle	Audience outdegree	3	-0.0571145	0.0610373	-0.2122195	0.0979905
Middle	Audience outdegree	4	0.0075682	0.0822452	-0.2014293	0.2165657
Middle	Audience outdegree	5	0.0073067	0.0590082	-0.1426422	0.1572556
Middle	Audience outdegree	6	-0.0640521	0.0545907	-0.2027753	0.0746710
Middle	Audience outdegree	7	0.0096751	0.0675520	-0.1619846	0.1813349
Middle	Audience outdegree	8	-0.0467266	0.0466153	-0.1651831	0.0717299
Middle	Audience outdegree	9	-0.0698553	0.0620182	-0.2274528	0.0877422
Middle	Audience outdegree	10	0.0204894	0.0768135	-0.1747055	0.2156842
Middle	Audience outdegree	11	0.0790583	0.0798770	-0.1239213	0.2820380
Middle	Audience outdegree	12	0.0703321	0.0827832	-0.1400325	0.2806968
Middle	Audience outdegree	13	-0.0456160	0.0757388	-0.2380798	0.1468478
Middle	Audience outdegree	14	0.0552945	0.1435661	-0.3095286	0.4201176
Middle	Audience outdegree	15	-0.0078969	0.0972219	-0.2549525	0.2391587
Middle	Audience outdegree	16	0.0352311	0.1470545	-0.3384566	0.4089188
Middle	Audience outdegree	17	0.1755061	0.2281907	-0.4043609	0.7553731
Middle	Audience outdegree	18	0.1565502	0.2265636	-0.4191821	0.7322825
Middle	Audience outdegree	19	-0.0045308	0.1792947	-0.4601456	0.4510840
Middle	Audience outdegree	20	0.0823335	0.1838426	-0.3848383	0.5495052
Middle	Audience outdegree	21	0.0692887	0.1325244	-0.2674757	0.4060532
Middle	Audience outdegree	22	0.0536121	0.1280044	-0.2716666	0.3788908
Middle	Audience outdegree	23	0.0810433	0.1829698	-0.3839108	0.5459974
Middle	Audience outdegree	24	0.1785521	0.2461416	-0.4469308	0.8040351
Middle	Audience outdegree	25	0.1093083	0.2369019	-0.4926952	0.7113118
Middle	Audience outdegree	26	-0.0950690	0.0912582	-0.3269700	0.1368319

Middle	Audience outdegree	27	-0.3192944	0.1321228	-0.6550385	0.0164497
Middle	Audience outdegree	28	-0.1310895	0.2163468	-0.6808595	0.4186805
Middle	Audience outdegree	29	-0.2215932	0.1623479	-0.6341437	0.1909574
Middle	Audience outdegree	30	-0.3930700	0.0469743	-0.5124388	-0.2737012

sample	outcome	event.time	estimate	std.error	conf.low	conf.high
Least	Audience outdegree	-30	0.0518466	0.0248609	-0.0062203	0.1099135
Least	Audience outdegree	-29	-0.0186255	0.0634214	-0.1667569	0.1295059
Least	Audience outdegree	-28	-0.0545332	0.0140011	-0.0872352	-0.0218312
Least	Audience outdegree	-27	0.1398761	0.0188616	0.0958217	0.1839305
Least	Audience outdegree	-26	-0.0266208	0.0201583	-0.0737039	0.0204622
Least	Audience outdegree	-25	-0.0309579	0.0247201	-0.0886959	0.0267800
Least	Audience outdegree	-24	-0.0226962	0.0384983	-0.1126155	0.0672231
Least	Audience outdegree	-23	-0.0276695	0.0412778	-0.1240808	0.0687418
Least	Audience outdegree	-22	0.0556713	0.0240091	-0.0004062	0.1117487
Least	Audience outdegree	-21	-0.0184341	0.0652570	-0.1708529	0.1339847
Least	Audience outdegree	-20	-0.0725225	0.0274091	-0.1365410	-0.0085039
Least	Audience outdegree	-19	0.0164721	0.0485243	-0.0968647	0.1298089
Least	Audience outdegree	-18	-0.0196807	0.0250792	-0.0782575	0.0388961
Least	Audience outdegree	-17	-0.0082041	0.0152446	-0.0438105	0.0274024
Least	Audience outdegree	-16	-0.0166925	0.0462732	-0.1247713	0.0913864
Least	Audience outdegree	-15	0.0027593	0.0130639	-0.0277537	0.0332724
Least	Audience outdegree	-14	-0.0202818	0.0201113	-0.0672553	0.0266917
Least	Audience outdegree	-13	-0.0032338	0.0288684	-0.0706609	0.0641932
Least	Audience outdegree	-12	-0.0517073	0.0703109	-0.2159303	0.1125157
Least	Audience outdegree	-11	0.0535701	0.0316461	-0.0203448	0.1274849
Least	Audience outdegree	-10	-0.0335366	0.0381971	-0.1227523	0.0556791
Least	Audience outdegree	-9	-0.0431417	0.0454763	-0.1493594	0.0630760
Least	Audience outdegree	-8	-0.0289893	0.0408600	-0.1244248	0.0664462
Least	Audience outdegree	-7	0.0105317	0.0218085	-0.0404059	0.0614692
Least	Audience outdegree	-6	-0.0612439	0.0537204	-0.1867170	0.0642292
Least	Audience outdegree	-5	-0.0041179	0.0254058	-0.0634576	0.0552218
Least	Audience outdegree	-4	0.0830970	0.0758907	-0.0941586	0.2603527
Least	Audience outdegree	-3	-0.0723214	0.0397419	-0.1651453	0.0205026
Least	Audience outdegree	-2	-0.0475149	0.0677475	-0.2057507	0.1107208
Least	Audience outdegree	-1	-0.0537088	0.0293306	-0.1222154	0.0147978
Least	Audience outdegree	0	-0.0267368	0.0575889	-0.1612456	0.1077720
Least	Audience outdegree	1	-0.0492741	0.0952747	-0.2718045	0.1732562
Least	Audience outdegree	2	-0.0162690	0.0913147	-0.2295500	0.1970120
Least	Audience outdegree	3	0.0003421	0.0654425	-0.1525101	0.1531942
Least	Audience outdegree	4	-0.0268886	0.1626715	-0.4068355	0.3530583

Least	Audience outdegree	5	-0.0175894	0.1495347	-0.3668532	0.3316744
Least	Audience outdegree	6	-0.0621990	0.1148241	-0.3303901	0.2059920
Least	Audience outdegree	7	-0.0250436	0.1065996	-0.2740252	0.2239379
Least	Audience outdegree	8	-0.0649096	0.0754519	-0.2411403	0.1113211
Least	Audience outdegree	9	-0.0048829	0.0904290	-0.2160951	0.2063293
Least	Audience outdegree	10	-0.0487233	0.1001145	-0.2825577	0.1851112
Least	Audience outdegree	11	-0.0405186	0.0936936	-0.2593561	0.1783188
Least	Audience outdegree	12	-0.0298133	0.0672712	-0.1869367	0.1273100
Least	Audience outdegree	13	-0.1352923	0.0441398	-0.2383882	-0.0321963
Least	Audience outdegree	14	-0.1200665	0.0749556	-0.2951379	0.0550049
Least	Audience outdegree	15	-0.0900541	0.1328294	-0.4002996	0.2201914
Least	Audience outdegree	16	-0.1087071	0.1952551	-0.5647585	0.3473444
Least	Audience outdegree	17	-0.1504351	0.2696243	-0.7801884	0.4793182
Least	Audience outdegree	18	-0.1087645	0.2892128	-0.7842700	0.5667410
Least	Audience outdegree	19	-0.2428675	0.2111186	-0.7359710	0.2502359
Least	Audience outdegree	20	-0.1456167	0.1608346	-0.5212733	0.2300399
Least	Audience outdegree	21	-0.2145017	0.1463014	-0.5562134	0.1272099
Least	Audience outdegree	22	-0.2136051	0.1949522	-0.6689492	0.2417390
Least	Audience outdegree	23	-0.2476906	0.2633820	-0.8628639	0.3674828
Least	Audience outdegree	24	-0.2008785	0.1468205	-0.5438027	0.1420456
Least	Audience outdegree	25	-0.1743321	0.2706978	-0.8065928	0.4579286
Least	Audience outdegree	26	-0.2305932	0.1751587	-0.6397060	0.1785196
Least	Audience outdegree	27	-0.4431228	0.4383555	-1.4669766	0.5807309
Least	Audience outdegree	28	-0.5562855	0.6782105	-2.1403612	1.0277902
Least	Audience outdegree	29	-0.5358466	0.4390698	-1.5613687	0.4896755
Least	Audience outdegree	30	-0.6250028	0.7176262	-2.3011407	1.0511351

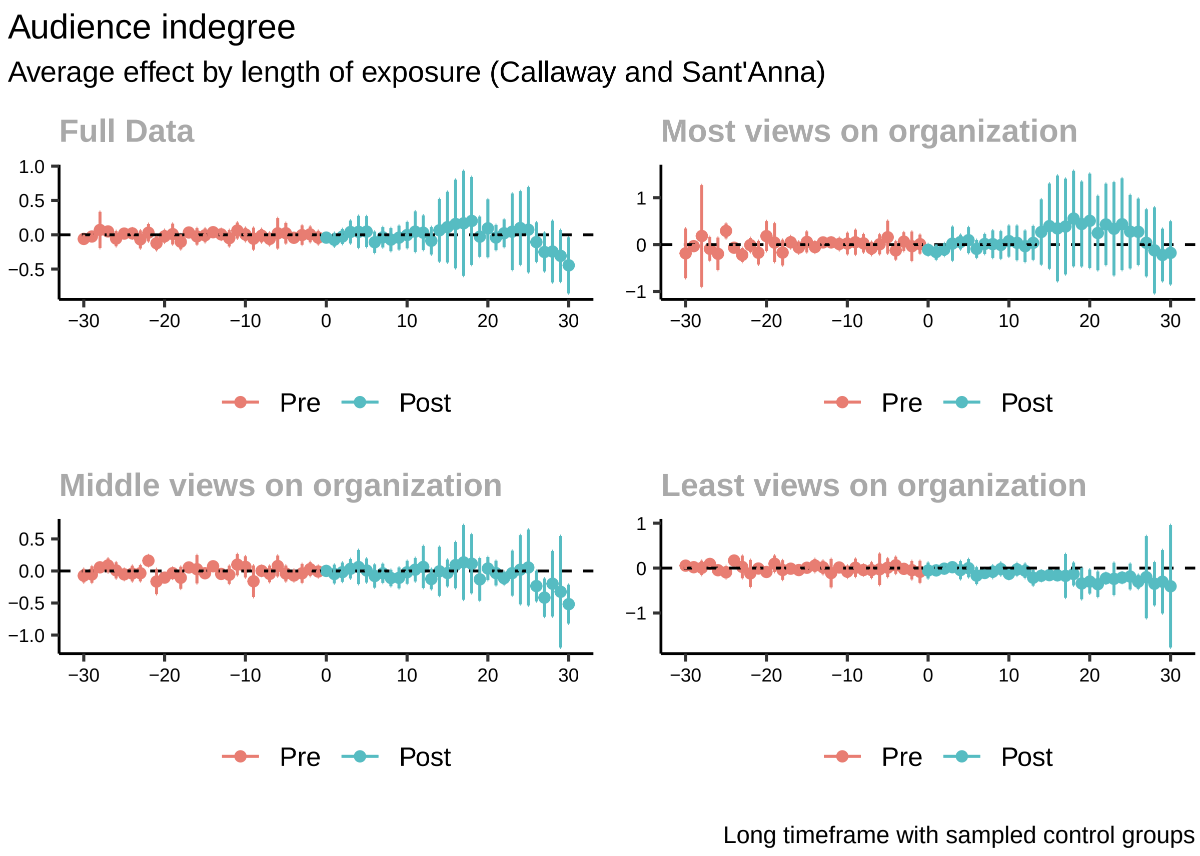

sample	outcome	event.time	estimate	std.error	conf.low	conf.high
Full	Audience indegree	-30	-0.0607239	0.0231598	-0.1238096	0.0023618
Full	Audience indegree	-29	-0.0252569	0.0204178	-0.0808734	0.0303596
Full	Audience indegree	-28	0.0706010	0.0966644	-0.1927059	0.3339079
Full	Audience indegree	-27	0.0484789	0.0235522	-0.0156755	0.1126334
Full	Audience indegree	-26	-0.0557473	0.0403667	-0.1657034	0.0542088
Full	Audience indegree	-25	0.0172862	0.0160629	-0.0264681	0.0610405
Full	Audience indegree	-24	0.0204425	0.0268811	-0.0527797	0.0936648
Full	Audience indegree	-23	-0.0672894	0.0480989	-0.1983074	0.0637287
Full	Audience indegree	-22	0.0304530	0.0464591	-0.0960983	0.1570043
Full	Audience indegree	-21	-0.1230796	0.0374045	-0.2249667	-0.0211925
Full	Audience indegree	-20	-0.0218771	0.0340459	-0.1146156	0.0708614
Full	Audience indegree	-19	0.0103373	0.0552643	-0.1401986	0.1608731
Full	Audience indegree	-18	-0.1023710	0.0401499	-0.2117366	0.0069946
Full	Audience indegree	-17	0.0332864	0.0173088	-0.0138616	0.0804344
Full	Audience indegree	-16	-0.0234777	0.0440312	-0.1434155	0.0964601
Full	Audience indegree	-15	-0.0071779	0.0370437	-0.1080822	0.0937263
Full	Audience indegree	-14	0.0373111	0.0101101	0.0097719	0.0648504
Full	Audience indegree	-13	0.0064488	0.0286753	-0.0716607	0.0845583
Full	Audience indegree	-12	-0.0497349	0.0434987	-0.1682222	0.0687524

Full	Audience indegree	-11	0.0617848	0.0428426	-0.0549153	0.1784849
Full	Audience indegree	-10	0.0044249	0.0355958	-0.0925354	0.1013853
Full	Audience indegree	-9	-0.0571453	0.0584674	-0.2164063	0.1021158
Full	Audience indegree	-8	-0.0131077	0.0360128	-0.1112039	0.0849885
Full	Audience indegree	-7	-0.0601662	0.0341246	-0.1531191	0.0327866
Full	Audience indegree	-6	0.0210402	0.0814367	-0.2007874	0.2428678
Full	Audience indegree	-5	0.0222288	0.0551303	-0.1279422	0.1723997
Full	Audience indegree	-4	-0.0438509	0.0249517	-0.1118175	0.0241158
Full	Audience indegree	-3	-0.0041962	0.0518678	-0.1454804	0.1370881
Full	Audience indegree	-2	0.0063861	0.0419304	-0.1078292	0.1206014
Full	Audience indegree	-1	-0.0411140	0.0360715	-0.1393703	0.0571423
Full	Audience indegree	0	-0.0415524	0.0134643	-0.0782281	-0.0048766
Full	Audience indegree	1	-0.0724016	0.0366089	-0.1721216	0.0273184
Full	Audience indegree	2	-0.0259295	0.0396759	-0.1340038	0.0821449
Full	Audience indegree	3	0.0405616	0.0608462	-0.1251792	0.2063024
Full	Audience indegree	4	0.0428551	0.0852946	-0.1894812	0.2751913
Full	Audience indegree	5	0.0462647	0.0824383	-0.1782912	0.2708207
Full	Audience indegree	6	-0.1085658	0.0571999	-0.2643742	0.0472427
Full	Audience indegree	7	-0.0370959	0.0545226	-0.1856115	0.1114197
Full	Audience indegree	8	-0.0708971	0.0617076	-0.2389843	0.0971900
Full	Audience indegree	9	-0.0419012	0.0638038	-0.2156981	0.1318958
Full	Audience indegree	10	-0.0041876	0.0702373	-0.1955091	0.1871338
Full	Audience indegree	11	0.0453624	0.1084315	-0.2499970	0.3407219
Full	Audience indegree	12	0.0305234	0.0922239	-0.2206878	0.2817347
Full	Audience indegree	13	-0.0872225	0.0730239	-0.2861344	0.1116894
Full	Audience indegree	14	0.0650433	0.1672748	-0.3906012	0.5206879
Full	Audience indegree	15	0.1110425	0.1887503	-0.4030998	0.6251848
Full	Audience indegree	16	0.1574964	0.2363271	-0.4862417	0.8012345
Full	Audience indegree	17	0.1670325	0.2805687	-0.5972164	0.9312815
Full	Audience indegree	18	0.2019498	0.2351079	-0.4384673	0.8423668
Full	Audience indegree	19	-0.0275791	0.1076584	-0.3208328	0.2656747
Full	Audience indegree	20	0.0946083	0.1548300	-0.3271375	0.5163541
Full	Audience indegree	21	-0.0401479	0.0674677	-0.2239251	0.1436293
Full	Audience indegree	22	0.0218585	0.0743179	-0.1805782	0.2242952
Full	Audience indegree	23	0.0441581	0.2047050	-0.5134435	0.6017598
Full	Audience indegree	24	0.0987656	0.1968549	-0.4374530	0.6349842
Full	Audience indegree	25	0.0737282	0.2276588	-0.5463980	0.6938543
Full	Audience indegree	26	-0.1066883	0.1054945	-0.3940477	0.1806710
Full	Audience indegree	27	-0.2511059	0.1033401	-0.5325970	0.0303851
Full	Audience indegree	28	-0.2447269	0.1643793	-0.6924843	0.2030306
Full	Audience indegree	29	-0.3076177	0.1384006	-0.6846109	0.0693754

Full	Audience indegree	30	-0.4454500	0.1496703	-0.8531411	-0.0377590

sample	outcome	event.time	estimate	std.error	conf.low	conf.high
Most	Audience indegree	-30	-0.1857832	0.2122527	-0.7134741	0.3419077
Most	Audience indegree	-29	-0.0326764	0.0204494	-0.0835167	0.0181639
Most	Audience indegree	-28	0.1837263	0.4355218	-0.8990437	1.2664963
Most	Audience indegree	-27	-0.0901910	0.1018797	-0.3434786	0.1630966
Most	Audience indegree	-26	-0.1963854	0.1388206	-0.5415135	0.1487427
Most	Audience indegree	-25	0.2915338	0.0640909	0.1321946	0.4508729
Most	Audience indegree	-24	-0.0668911	0.0353123	-0.1546825	0.0209003
Most	Audience indegree	-23	-0.2211847	0.0592494	-0.3684874	-0.0738821
Most	Audience indegree	-22	-0.0100907	0.0605883	-0.1607219	0.1405404
Most	Audience indegree	-21	-0.1735370	0.1010344	-0.4247232	0.0776492
Most	Audience indegree	-20	0.1811895	0.1277492	-0.1364134	0.4987924
Most	Audience indegree	-19	0.0422514	0.1661022	-0.3707026	0.4552054
Most	Audience indegree	-18	-0.1695087	0.1094899	-0.4417163	0.1026990
Most	Audience indegree	-17	0.0503794	0.0515992	-0.0779038	0.1786625
Most	Audience indegree	-16	-0.0631083	0.0512188	-0.1904455	0.0642290
Most	Audience indegree	-15	0.0580828	0.0896129	-0.1647078	0.2808734
Most	Audience indegree	-14	-0.0527875	0.0482424	-0.1727251	0.0671500
Most	Audience indegree	-13	0.0464448	0.0360297	-0.0431303	0.1360199
Most	Audience indegree	-12	0.0471615	0.0415566	-0.0561541	0.1504772
Most	Audience indegree	-11	0.0122176	0.0536393	-0.1211375	0.1455727
Most	Audience indegree	-10	0.0226298	0.0920182	-0.2061409	0.2514004
Most	Audience indegree	-9	0.0506694	0.1064937	-0.2140893	0.3154280
Most	Audience indegree	-8	0.0272208	0.0789201	-0.1689858	0.2234275
Most	Audience indegree	-7	-0.0810896	0.0563488	-0.2211809	0.0590016
Most	Audience indegree	-6	0.0071971	0.0850873	-0.2043421	0.2187363
Most	Audience indegree	-5	0.1572602	0.1402190	-0.1913446	0.5058650
Most	Audience indegree	-4	-0.1247552	0.0785943	-0.3201520	0.0706416
Most	Audience indegree	-3	0.0634063	0.0801659	-0.1358978	0.2627103
Most	Audience indegree	-2	-0.0347557	0.1253634	-0.3464272	0.2769157
Most	Audience indegree	-1	0.0096275	0.0814829	-0.1929508	0.2122058
Most	Audience indegree	0	-0.1121748	0.0489874	-0.2339645	0.0096149
Most	Audience indegree	1	-0.1589796	0.0659967	-0.3230569	0.0050977
Most	Audience indegree	2	-0.1045848	0.0547444	-0.2406872	0.0315176
Most	Audience indegree	3	0.0203812	0.1457249	-0.3419118	0.3826742
Most	Audience indegree	4	0.0542175	0.0624208	-0.1009696	0.2094047
Most	Audience indegree	5	0.0948393	0.1108455	-0.1807386	0.3704173
Most	Audience indegree	6	-0.0912604	0.0761033	-0.2804642	0.0979433
Most	Audience indegree	7	0.0137441	0.0854253	-0.1986356	0.2261237

Most	Audience indegree	8	0.0099822	0.1176101	-0.2824134	0.3023777
Most	Audience indegree	9	-0.0078376	0.1178313	-0.3007832	0.2851081
Most	Audience indegree	10	0.0750111	0.1331444	-0.2560052	0.4060274
Most	Audience indegree	11	0.0380788	0.1480910	-0.3300968	0.4062544
Most	Audience indegree	12	-0.0343814	0.1349251	-0.3698248	0.3010619
Most	Audience indegree	13	0.0370799	0.1449589	-0.3233088	0.3974687
Most	Audience indegree	14	0.2687876	0.2798241	-0.4268954	0.9644706
Most	Audience indegree	15	0.3942345	0.3651533	-0.5135893	1.3020583
Most	Audience indegree	16	0.3452279	0.4527051	-0.7802623	1.4707181
Most	Audience indegree	17	0.3855943	0.4094473	-0.6323508	1.4035393
Most	Audience indegree	18	0.5550776	0.4089163	-0.4615473	1.5717025
Most	Audience indegree	19	0.4385014	0.3642007	-0.4669540	1.3439568
Most	Audience indegree	20	0.5070522	0.4037486	-0.4967251	1.5108295
Most	Audience indegree	21	0.2465373	0.3184477	-0.5451697	1.0382442
Most	Audience indegree	22	0.4305183	0.3483098	-0.4354301	1.2964668
Most	Audience indegree	23	0.3375341	0.3994682	-0.6556015	1.3306696
Most	Audience indegree	24	0.4349821	0.3930997	-0.5423205	1.4122848
Most	Audience indegree	25	0.2761399	0.3148650	-0.5066599	1.0589398
Most	Audience indegree	26	0.2727973	0.2839255	-0.4330825	0.9786771
Most	Audience indegree	27	0.0378055	0.2871135	-0.6760001	0.7516110
Most	Audience indegree	28	-0.1225943	0.3683233	-1.0382992	0.7931106
Most	Audience indegree	29	-0.2219710	0.2248092	-0.7808790	0.3369370
Most	Audience indegree	30	-0.1769360	0.2707927	-0.8501658	0.4962938

sample	outcome	event.time	estimate	std.error	conf.low	conf.high
Middle	Audience indegree	-30	-0.0730623	0.0416698	-0.1791876	0.0330629
Middle	Audience indegree	-29	-0.0550229	0.0503944	-0.1833680	0.0733222
Middle	Audience indegree	-28	0.0566745	0.0287661	-0.0165874	0.1299363
Middle	Audience indegree	-27	0.0864259	0.0430435	-0.0231979	0.1960497
Middle	Audience indegree	-26	0.0090984	0.0493240	-0.1165207	0.1347175
Middle	Audience indegree	-25	-0.0526141	0.0339117	-0.1389808	0.0337527
Middle	Audience indegree	-24	-0.0403637	0.0474845	-0.1612980	0.0805706
Middle	Audience indegree	-23	-0.0349386	0.0489734	-0.1596649	0.0897876
Middle	Audience indegree	-22	0.1603063	0.0333512	0.0753670	0.2452456
Middle	Audience indegree	-21	-0.1645868	0.0767839	-0.3601411	0.0309675
Middle	Audience indegree	-20	-0.1038992	0.0232581	-0.1631333	-0.0446651
Middle	Audience indegree	-19	-0.0341081	0.0338336	-0.1202760	0.0520597
Middle	Audience indegree	-18	-0.1057484	0.0670311	-0.2764641	0.0649674
Middle	Audience indegree	-17	0.0551521	0.0144537	0.0183411	0.0919630
Middle	Audience indegree	-16	0.0276575	0.0862292	-0.1919522	0.2472673
Middle	Audience indegree	-15	-0.0349632	0.0280670	-0.1064446	0.0365182

Middle	Audience indegree	-14	0.0736154	0.0257277	0.0080917	0.1391392
Middle	Audience indegree	-13	-0.0464342	0.0267145	-0.1144710	0.0216026
Middle	Audience indegree	-12	-0.0591921	0.0551794	-0.1997239	0.0813397
Middle	Audience indegree	-11	0.0988175	0.0643684	-0.0651170	0.2627520
Middle	Audience indegree	-10	0.0640545	0.0644053	-0.0999739	0.2280830
Middle	Audience indegree	-9	-0.1605358	0.0964034	-0.4060574	0.0849858
Middle	Audience indegree	-8	0.0021450	0.0304755	-0.0754705	0.0797605
Middle	Audience indegree	-7	-0.0459234	0.0503289	-0.1741017	0.0822548
Middle	Audience indegree	-6	0.0772934	0.0640850	-0.0859194	0.2405061
Middle	Audience indegree	-5	-0.0401296	0.0487611	-0.1643152	0.0840560
Middle	Audience indegree	-4	-0.0707827	0.0361638	-0.1628853	0.0213199
Middle	Audience indegree	-3	-0.0371909	0.0577032	-0.1841504	0.1097686
Middle	Audience indegree	-2	0.0289211	0.0425984	-0.0795692	0.1374115
Middle	Audience indegree	-1	-0.0084627	0.0352315	-0.0981907	0.0812653
Middle	Audience indegree	0	0.0007485	0.0283796	-0.0715291	0.0730262
Middle	Audience indegree	1	-0.0473542	0.0567494	-0.1918844	0.0971760
Middle	Audience indegree	2	-0.0183844	0.0586685	-0.1678022	0.1310333
Middle	Audience indegree	3	0.0342347	0.0595415	-0.1174065	0.1858759
Middle	Audience indegree	4	0.0619110	0.1042573	-0.2036130	0.3274350
Middle	Audience indegree	5	0.0073765	0.0735484	-0.1799377	0.1946907
Middle	Audience indegree	6	-0.0786664	0.0710602	-0.2596437	0.1023108
Middle	Audience indegree	7	-0.0364267	0.0583842	-0.1851204	0.1122669
Middle	Audience indegree	8	-0.1098233	0.0499507	-0.2370386	0.0173920
Middle	Audience indegree	9	-0.1073357	0.0637489	-0.2696923	0.0550210
Middle	Audience indegree	10	-0.0204657	0.0700472	-0.1988629	0.1579316
Middle	Audience indegree	11	0.0197413	0.0718691	-0.1632960	0.2027786
Middle	Audience indegree	12	0.0630997	0.1282127	-0.2634344	0.3896337
Middle	Audience indegree	13	-0.1257181	0.0619095	-0.2833900	0.0319539
Middle	Audience indegree	14	-0.0051694	0.1496822	-0.3863822	0.3760434
Middle	Audience indegree	15	-0.0315509	0.0815586	-0.2392657	0.1761638
Middle	Audience indegree	16	0.0920292	0.1411157	-0.2673664	0.4514248
Middle	Audience indegree	17	0.1342346	0.2283686	-0.4473780	0.7158472
Middle	Audience indegree	18	0.1129565	0.1805802	-0.3469479	0.5728609
Middle	Audience indegree	19	-0.1308731	0.1304843	-0.4631925	0.2014463
Middle	Audience indegree	20	0.0382717	0.0706200	-0.1415843	0.2181277
Middle	Audience indegree	21	-0.0330661	0.0755796	-0.2255533	0.1594210
Middle	Audience indegree	22	-0.1118187	0.0402286	-0.2142736	-0.0093639
Middle	Audience indegree	23	-0.0340754	0.1375063	-0.3842785	0.3161278
Middle	Audience indegree	24	0.0193585	0.2112042	-0.5185395	0.5572565
Middle	Audience indegree	25	0.0541932	0.2319372	-0.5365080	0.6448944
Middle	Audience indegree	26	-0.2351540	0.0932033	-0.4725256	0.0022176

Middle	Audience indegree	27	-0.4155137	0.1168526	-0.7131156	-0.1179117
Middle	Audience indegree	28	-0.1990452	0.1998695	-0.7080759	0.3099855
Middle	Audience indegree	29	-0.3246592	0.3412080	-1.1936529	0.5443345
Middle	Audience indegree	30	-0.5176073	0.1185880	-0.8196289	-0.2155857

sample	outcome	event.time	estimate	std.error	conf.low	conf.high
Least	Audience indegree	-30	0.0543387	0.0310720	-0.0212604	0.1299378
Least	Audience indegree	-29	0.0195647	0.0399346	-0.0775973	0.1167266
Least	Audience indegree	-28	0.0054613	0.0648329	-0.1522791	0.1632017
Least	Audience indegree	-27	0.0925192	0.0284276	0.0233541	0.1616843
Least	Audience indegree	-26	-0.0474698	0.0244840	-0.1070402	0.0121006
Least	Audience indegree	-25	-0.0936883	0.0565811	-0.2313518	0.0439751
Least	Audience indegree	-24	0.1682613	0.0422410	0.0654876	0.2710351
Least	Audience indegree	-23	0.0259568	0.1029515	-0.2245272	0.2764408
Least	Audience indegree	-22	-0.1184619	0.1233978	-0.4186923	0.1817685
Least	Audience indegree	-21	-0.0096910	0.0311939	-0.0855866	0.0662047
Least	Audience indegree	-20	-0.0867433	0.0277937	-0.1543662	-0.0191204
Least	Audience indegree	-19	0.0966151	0.0751853	-0.0863130	0.2795433
Least	Audience indegree	-18	-0.0432174	0.0892069	-0.2602605	0.1738257
Least	Audience indegree	-17	-0.0136880	0.0208370	-0.0643850	0.0370090
Least	Audience indegree	-16	-0.0438003	0.0386369	-0.1378050	0.0502045
Least	Audience indegree	-15	0.0072087	0.0337447	-0.0748930	0.0893105
Least	Audience indegree	-14	0.0568703	0.0601830	-0.0895568	0.2032973
Least	Audience indegree	-13	0.0160695	0.0627006	-0.1364829	0.1686218
Least	Audience indegree	-12	-0.1115107	0.1295689	-0.4267557	0.2037342
Least	Audience indegree	-11	0.0134423	0.0417483	-0.0881326	0.1150173
Least	Audience indegree	-10	-0.0788075	0.0563101	-0.2158117	0.0581967
Least	Audience indegree	-9	0.0070743	0.0826719	-0.1940690	0.2082176
Least	Audience indegree	-8	-0.0430712	0.0488298	-0.1618756	0.0757332
Least	Audience indegree	-7	-0.0450164	0.0713010	-0.2184938	0.1284609
Least	Audience indegree	-6	-0.0269816	0.1431371	-0.3752385	0.3212753
Least	Audience indegree	-5	0.0089137	0.0854872	-0.1990793	0.2169067
Least	Audience indegree	-4	0.0627195	0.0772092	-0.1251328	0.2505717
Least	Audience indegree	-3	-0.0169411	0.0333848	-0.0981674	0.0642851
Least	Audience indegree	-2	-0.0469622	0.0841454	-0.2516904	0.1577661
Least	Audience indegree	-1	-0.0826413	0.1002231	-0.3264872	0.1612045
Least	Audience indegree	0	-0.0545332	0.0731592	-0.2325318	0.1234653
Least	Audience indegree	1	-0.0510883	0.0441579	-0.1585259	0.0563493
Least	Audience indegree	2	-0.0127632	0.0428722	-0.1170724	0.0915461
Least	Audience indegree	3	0.0196215	0.0334128	-0.0616728	0.1009158
Least	Audience indegree	4	-0.0455469	0.0828971	-0.2472379	0.1561441

Least	Audience indegree	5	-0.0012778	0.0829255	-0.2030380	0.2004825
Least	Audience indegree	6	-0.1678614	0.0766211	-0.3542828	0.0185601
Least	Audience indegree	7	-0.1095546	0.0333141	-0.1906087	-0.0285005
Least	Audience indegree	8	-0.0805787	0.0576513	-0.2208460	0.0596886
Least	Audience indegree	9	-0.0204064	0.0589300	-0.1637847	0.1229720
Least	Audience indegree	10	-0.1292107	0.0463113	-0.2418875	-0.0165338
Least	Audience indegree	11	-0.0292968	0.0578569	-0.1700643	0.1114706
Least	Audience indegree	12	-0.0601675	0.0661161	-0.2210299	0.1006950
Least	Audience indegree	13	-0.2160860	0.0715147	-0.3900835	-0.0420886
Least	Audience indegree	14	-0.1715493	0.0475842	-0.2873231	-0.0557755
Least	Audience indegree	15	-0.1572559	0.0333526	-0.2384037	-0.0761081
Least	Audience indegree	16	-0.1605253	0.0442805	-0.2682611	-0.0527894
Least	Audience indegree	17	-0.1736911	0.1996159	-0.6593625	0.3119804
Least	Audience indegree	18	-0.1317022	0.1048923	-0.3869082	0.1235038
Least	Audience indegree	19	-0.3425932	0.1437965	-0.6924543	0.0072678
Least	Audience indegree	20	-0.2996267	0.1099166	-0.5670572	-0.0321963
Least	Audience indegree	21	-0.3591404	0.1159233	-0.6411851	-0.0770957
Least	Audience indegree	22	-0.2268652	0.0483255	-0.3444426	-0.1092877
Least	Audience indegree	23	-0.2398892	0.1472256	-0.5980935	0.1183150
Least	Audience indegree	24	-0.2130031	0.0476242	-0.3288743	-0.0971319
Least	Audience indegree	25	-0.1881089	0.1171978	-0.4732547	0.0970369
Least	Audience indegree	26	-0.3042618	0.0642984	-0.4607017	-0.1478218
Least	Audience indegree	27	-0.2020640	0.3753659	-1.1153403	0.7112124
Least	Audience indegree	28	-0.3482591	0.1974151	-0.8285760	0.1320578
Least	Audience indegree	29	-0.3034300	0.2893649	-1.0074633	0.4006033
Least	Audience indegree	30	-0.4053786	0.5601749	-1.7683006	0.9575435

Audience outdegree/total outdegree

Average effect by length of exposure (Callaway and Sant'Anna)

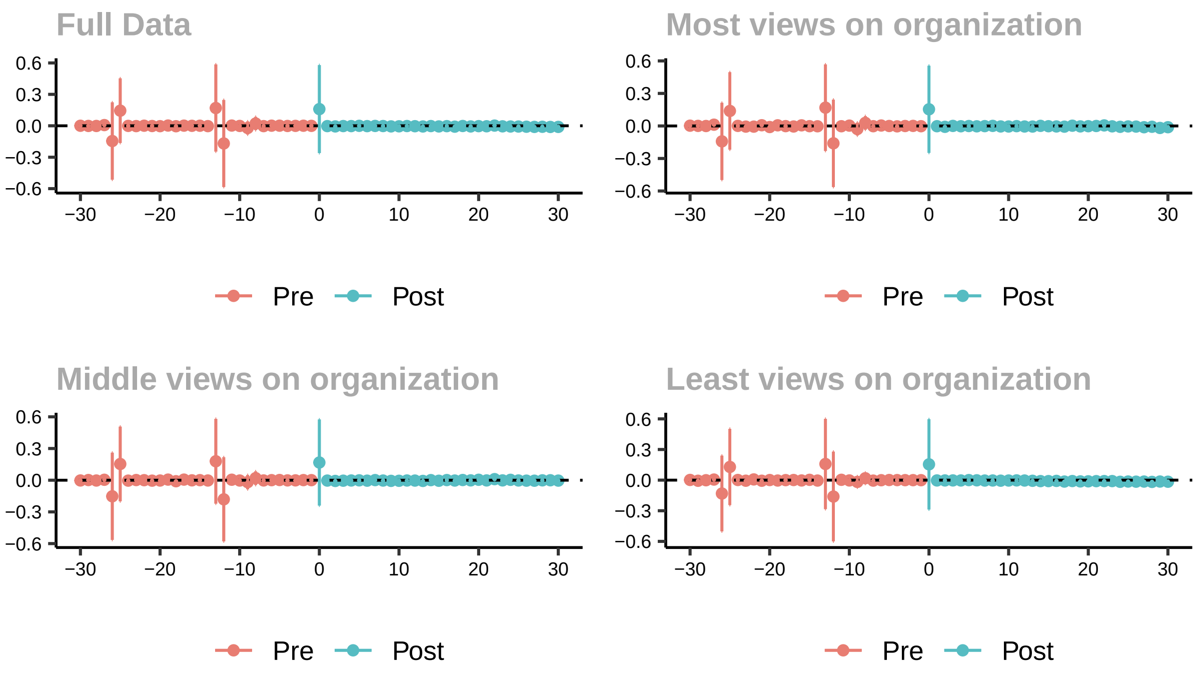

Long timeframe with sampled control groups

sample	outcome	event.time	estimate	std.error	conf.low	conf.high
Full	Audience outdegree/total outdegree	-30	0.0003922	0.0011097	-0.0024768	0.0032612
Full	Audience outdegree/total outdegree	-29	-0.0013186	0.0015954	-0.0054434	0.0028063
Full	Audience outdegree/total outdegree	-28	-0.0017472	0.0012419	-0.0049582	0.0014637
Full	Audience outdegree/total outdegree	-27	0.0067019	0.0018176	0.0020026	0.0114012
Full	Audience outdegree/total outdegree	-26	-0.1455334	0.1426846	-0.5144450	0.2233783
Full	Audience outdegree/total outdegree	-25	0.1443141	0.1191481	-0.1637438	0.4523720
Full	Audience outdegree/total outdegree	-24	-0.0009487	0.0017238	-0.0054057	0.0035082
Full	Audience outdegree/total outdegree	-23	-0.0029552	0.0015987	-0.0070886	0.0011782
Full	Audience outdegree/total outdegree	-22	0.0008853	0.0010684	-0.0018771	0.0036477
Full	Audience outdegree/total outdegree	-21	-0.0020904	0.0019915	-0.0072393	0.0030585
Full	Audience outdegree/total outdegree	-20	-0.0035976	0.0025294	-0.0101373	0.0029421
Full	Audience outdegree/total outdegree	-19	0.0024774	0.0006763	0.0007289	0.0042259
Full	Audience outdegree/total outdegree	-18	-0.0042713	0.0009866	-0.0068222	-0.0017203
Full	Audience outdegree/total outdegree	-17	0.0013545	0.0010493	-0.0013585	0.0040674
Full	Audience outdegree/total outdegree	-16	-0.0000772	0.0011067	-0.0029386	0.0027842
Full	Audience outdegree/total outdegree	-15	0.0006997	0.0009379	-0.0017251	0.0031246
Full	Audience outdegree/total outdegree	-14	-0.0032848	0.0010065	-0.0058870	-0.0006826
Full	Audience outdegree/total outdegree	-13	0.1695991	0.1609444	-0.2465232	0.5857213
Full	Audience outdegree/total outdegree	-12	-0.1687158	0.1605432	-0.5838008	0.2463693

Full	Audience outdegree/total outdegree	-11	0.0022633	0.0009280	-0.0001361	0.0046628
Full	Audience outdegree/total outdegree	-10	-0.0013628	0.0018796	-0.0062226	0.0034969
Full	Audience outdegree/total outdegree	-9	-0.0209682	0.0242413	-0.0836440	0.0417077
Full	Audience outdegree/total outdegree	-8	0.0216953	0.0244401	-0.0414946	0.0848852
Full	Audience outdegree/total outdegree	-7	-0.0030947	0.0008273	-0.0052337	-0.0009556
Full	Audience outdegree/total outdegree	-6	0.0011023	0.0015290	-0.0028509	0.0050555
Full	Audience outdegree/total outdegree	-5	0.0010896	0.0010818	-0.0017075	0.0038867
Full	Audience outdegree/total outdegree	-4	-0.0015652	0.0008526	-0.0037697	0.0006393
Full	Audience outdegree/total outdegree	-3	-0.0007891	0.0008783	-0.0030599	0.0014817
Full	Audience outdegree/total outdegree	-2	0.0005511	0.0007232	-0.0013189	0.0024210
Full	Audience outdegree/total outdegree	-1	-0.0006246	0.0009328	-0.0030363	0.0017870
Full	Audience outdegree/total outdegree	0	0.1594231	0.1623170	-0.2602481	0.5790943
Full	Audience outdegree/total outdegree	1	-0.0030804	0.0014717	-0.0068854	0.0007246
Full	Audience outdegree/total outdegree	2	-0.0060452	0.0015379	-0.0100214	-0.0020691
Full	Audience outdegree/total outdegree	3	-0.0026119	0.0009859	-0.0051609	-0.0000628
Full	Audience outdegree/total outdegree	4	-0.0029998	0.0004659	-0.0042044	-0.0017953
Full	Audience outdegree/total outdegree	5	-0.0004737	0.0025315	-0.0070190	0.0060716
Full	Audience outdegree/total outdegree	6	-0.0032654	0.0010729	-0.0060394	-0.0004914
Full	Audience outdegree/total outdegree	7	-0.0014917	0.0014850	-0.0053311	0.0023478
Full	Audience outdegree/total outdegree	8	-0.0024236	0.0013891	-0.0060150	0.0011679
Full	Audience outdegree/total outdegree	9	-0.0057651	0.0018113	-0.0104482	-0.0010821
Full	Audience outdegree/total outdegree	10	-0.0050961	0.0012181	-0.0082455	-0.0019467
Full	Audience outdegree/total outdegree	11	-0.0025571	0.0008328	-0.0047102	-0.0004040
Full	Audience outdegree/total outdegree	12	-0.0040642	0.0015614	-0.0081011	-0.0000272
Full	Audience outdegree/total outdegree	13	-0.0065827	0.0020768	-0.0119523	-0.0012132
Full	Audience outdegree/total outdegree	14	-0.0028223	0.0013145	-0.0062208	0.0005763
Full	Audience outdegree/total outdegree	15	-0.0062197	0.0030829	-0.0141906	0.0017512
Full	Audience outdegree/total outdegree	16	-0.0034971	0.0051139	-0.0167191	0.0097249
Full	Audience outdegree/total outdegree	17	-0.0082728	0.0060012	-0.0237888	0.0072433
Full	Audience outdegree/total outdegree	18	-0.0019266	0.0058218	-0.0169789	0.0131257
Full	Audience outdegree/total outdegree	19	-0.0062322	0.0035711	-0.0154654	0.0030009
Full	Audience outdegree/total outdegree	20	-0.0033773	0.0047540	-0.0156689	0.0089143
Full	Audience outdegree/total outdegree	21	-0.0044884	0.0023907	-0.0106695	0.0016926
Full	Audience outdegree/total outdegree	22	0.0020053	0.0048698	-0.0105856	0.0145962
Full	Audience outdegree/total outdegree	23	-0.0051944	0.0057709	-0.0201150	0.0097262
Full	Audience outdegree/total outdegree	24	-0.0067463	0.0041816	-0.0175579	0.0040654
Full	Audience outdegree/total outdegree	25	-0.0070469	0.0052582	-0.0206419	0.0065481
Full	Audience outdegree/total outdegree	26	-0.0087591	0.0025332	-0.0153086	-0.0022095
Full	Audience outdegree/total outdegree	27	-0.0105519	0.0017793	-0.0151524	-0.0059515
Full	Audience outdegree/total outdegree	28	-0.0092107	0.0053403	-0.0230180	0.0045965
Full	Audience outdegree/total outdegree	29	-0.0111293	0.0025305	-0.0176718	-0.0045868

Full	Audience outdegree/total outdegree	30	-0.0106818	0.0013495	-0.0141710	-0.0071925

sample	outcome	event.time	estimate	std.error	conf.low	conf.high
Most	Audience outdegree/total outdegree	-30	0.0025575	0.0041176	-0.0081029	0.0132179
Most	Audience outdegree/total outdegree	-29	0.0007283	0.0045729	-0.0111106	0.0125672
Most	Audience outdegree/total outdegree	-28	-0.0005844	0.0028089	-0.0078566	0.0066877
Most	Audience outdegree/total outdegree	-27	0.0116565	0.0032362	0.0032782	0.0200348
Most	Audience outdegree/total outdegree	-26	-0.1421223	0.1373195	-0.4976357	0.2133910
Most	Audience outdegree/total outdegree	-25	0.1384309	0.1378856	-0.2185480	0.4954098
Most	Audience outdegree/total outdegree	-24	0.0006362	0.0049733	-0.0122395	0.0135119
Most	Audience outdegree/total outdegree	-23	-0.0060986	0.0044333	-0.0175762	0.0053790
Most	Audience outdegree/total outdegree	-22	-0.0062116	0.0030359	-0.0140713	0.0016481
Most	Audience outdegree/total outdegree	-21	0.0066102	0.0033215	-0.0019889	0.0152094
Most	Audience outdegree/total outdegree	-20	-0.0099834	0.0071223	-0.0284227	0.0084559
Most	Audience outdegree/total outdegree	-19	0.0058652	0.0037071	-0.0037324	0.0154628
Most	Audience outdegree/total outdegree	-18	-0.0016229	0.0028228	-0.0089311	0.0056852
Most	Audience outdegree/total outdegree	-17	-0.0050567	0.0019940	-0.0102189	0.0001056
Most	Audience outdegree/total outdegree	-16	0.0056672	0.0035446	-0.0035097	0.0148440
Most	Audience outdegree/total outdegree	-15	-0.0021138	0.0023300	-0.0081462	0.0039186
Most	Audience outdegree/total outdegree	-14	-0.0029489	0.0011278	-0.0058686	-0.0000291
Most	Audience outdegree/total outdegree	-13	0.1686854	0.1540416	-0.2301205	0.5674914
Most	Audience outdegree/total outdegree	-12	-0.1602306	0.1554253	-0.5626189	0.2421577
Most	Audience outdegree/total outdegree	-11	-0.0025619	0.0039017	-0.0126632	0.0075395
Most	Audience outdegree/total outdegree	-10	0.0031290	0.0046116	-0.0088102	0.0150683
Most	Audience outdegree/total outdegree	-9	-0.0283888	0.0230937	-0.0881773	0.0313997
Most	Audience outdegree/total outdegree	-8	0.0280744	0.0243278	-0.0349091	0.0910580
Most	Audience outdegree/total outdegree	-7	-0.0024269	0.0018247	-0.0071510	0.0022973
Most	Audience outdegree/total outdegree	-6	0.0039777	0.0032057	-0.0043217	0.0122772
Most	Audience outdegree/total outdegree	-5	-0.0003449	0.0026580	-0.0072263	0.0065365
Most	Audience outdegree/total outdegree	-4	-0.0035645	0.0025966	-0.0102870	0.0031581
Most	Audience outdegree/total outdegree	-3	-0.0006056	0.0032147	-0.0089283	0.0077172
Most	Audience outdegree/total outdegree	-2	0.0006066	0.0021876	-0.0050570	0.0062701
Most	Audience outdegree/total outdegree	-1	-0.0022786	0.0024219	-0.0085489	0.0039917
Most	Audience outdegree/total outdegree	0	0.1537576	0.1551317	-0.2478705	0.5553858
Most	Audience outdegree/total outdegree	1	-0.0024191	0.0021172	-0.0079005	0.0030622
Most	Audience outdegree/total outdegree	2	-0.0079421	0.0010685	-0.0107085	-0.0051758
Most	Audience outdegree/total outdegree	3	0.0004098	0.0022777	-0.0054870	0.0063067
Most	Audience outdegree/total outdegree	4	-0.0029054	0.0024168	-0.0091625	0.0033517
Most	Audience outdegree/total outdegree	5	-0.0007835	0.0052063	-0.0142623	0.0126953
Most	Audience outdegree/total outdegree	6	-0.0032847	0.0033869	-0.0120534	0.0054839
Most	Audience outdegree/total outdegree	7	-0.0016200	0.0021858	-0.0072788	0.0040388

Most	Audience outdegree/total outdegree	8	0.0005365	0.0021815	-0.0051113	0.0061843
Most	Audience outdegree/total outdegree	9	-0.0045106	0.0059626	-0.0199474	0.0109261
Most	Audience outdegree/total outdegree	10	-0.0051205	0.0033552	-0.0138070	0.0035659
Most	Audience outdegree/total outdegree	11	-0.0017932	0.0031088	-0.0098419	0.0062554
Most	Audience outdegree/total outdegree	12	-0.0050200	0.0034154	-0.0138622	0.0038222
Most	Audience outdegree/total outdegree	13	-0.0054739	0.0030218	-0.0132973	0.0023495
Most	Audience outdegree/total outdegree	14	0.0007766	0.0060084	-0.0147788	0.0163321
Most	Audience outdegree/total outdegree	15	-0.0024589	0.0063097	-0.0187945	0.0138766
Most	Audience outdegree/total outdegree	16	-0.0045522	0.0116321	-0.0346670	0.0255627
Most	Audience outdegree/total outdegree	17	-0.0065359	0.0126259	-0.0392237	0.0261518
Most	Audience outdegree/total outdegree	18	0.0022829	0.0110025	-0.0262019	0.0307678
Most	Audience outdegree/total outdegree	19	-0.0040982	0.0077598	-0.0241879	0.0159915
Most	Audience outdegree/total outdegree	20	-0.0022589	0.0079859	-0.0229339	0.0184161
Most	Audience outdegree/total outdegree	21	-0.0002643	0.0095263	-0.0249275	0.0243989
Most	Audience outdegree/total outdegree	22	0.0050829	0.0094429	-0.0193643	0.0295302
Most	Audience outdegree/total outdegree	23	-0.0035568	0.0094919	-0.0281307	0.0210172
Most	Audience outdegree/total outdegree	24	-0.0081048	0.0108435	-0.0361780	0.0199684
Most	Audience outdegree/total outdegree	25	-0.0042449	0.0102473	-0.0307747	0.0222850
Most	Audience outdegree/total outdegree	26	-0.0058179	0.0063023	-0.0221344	0.0104985
Most	Audience outdegree/total outdegree	27	-0.0117307	0.0065219	-0.0286154	0.0051541
Most	Audience outdegree/total outdegree	28	-0.0074364	0.0092239	-0.0313167	0.0164439
Most	Audience outdegree/total outdegree	29	-0.0184597	0.0075513	-0.0380095	0.0010901
Most	Audience outdegree/total outdegree	30	-0.0115604	0.0078794	-0.0319598	0.0088391

sample	outcome	event.time	estimate	std.error	conf.low	conf.high
Middle	Audience outdegree/total outdegree	-30	-0.0024058	0.0014596	-0.0060418	0.0012301
Middle	Audience outdegree/total outdegree	-29	0.0014826	0.0009819	-0.0009634	0.0039285
Middle	Audience outdegree/total outdegree	-28	-0.0029614	0.0021090	-0.0082148	0.0022919
Middle	Audience outdegree/total outdegree	-27	0.0041751	0.0021477	-0.0011746	0.0095249
Middle	Audience outdegree/total outdegree	-26	-0.1522880	0.1656282	-0.5648603	0.2602843
Middle	Audience outdegree/total outdegree	-25	0.1535116	0.1419124	-0.1999858	0.5070090
Middle	Audience outdegree/total outdegree	-24	-0.0039384	0.0028002	-0.0109135	0.0030367
Middle	Audience outdegree/total outdegree	-23	0.0014665	0.0025064	-0.0047767	0.0077098
Middle	Audience outdegree/total outdegree	-22	0.0004005	0.0017722	-0.0040141	0.0048151
Middle	Audience outdegree/total outdegree	-21	-0.0043158	0.0016593	-0.0084491	-0.0001825
Middle	Audience outdegree/total outdegree	-20	-0.0030580	0.0023532	-0.0089197	0.0028038
Middle	Audience outdegree/total outdegree	-19	0.0056457	0.0006457	0.0040373	0.0072541
Middle	Audience outdegree/total outdegree	-18	-0.0096154	0.0026148	-0.0161287	-0.0031022
Middle	Audience outdegree/total outdegree	-17	0.0055048	0.0018492	0.0008985	0.0101110
Middle	Audience outdegree/total outdegree	-16	-0.0012738	0.0018706	-0.0059334	0.0033857
Middle	Audience outdegree/total outdegree	-15	0.0006477	0.0012384	-0.0024371	0.0037325

Middle	Audience outdegree/total outdegree	-14	-0.0028126	0.0015901	-0.0067734	0.0011482
Middle	Audience outdegree/total outdegree	-13	0.1795036	0.1609401	-0.2213908	0.5803981
Middle	Audience outdegree/total outdegree	-12	-0.1809584	0.1599275	-0.5793307	0.2174138
Middle	Audience outdegree/total outdegree	-11	0.0045831	0.0024813	-0.0015977	0.0107640
Middle	Audience outdegree/total outdegree	-10	-0.0030260	0.0031766	-0.0109388	0.0048868
Middle	Audience outdegree/total outdegree	-9	-0.0183572	0.0270048	-0.0856249	0.0489105
Middle	Audience outdegree/total outdegree	-8	0.0193961	0.0259459	-0.0452340	0.0840262
Middle	Audience outdegree/total outdegree	-7	-0.0022085	0.0021561	-0.0075792	0.0031622
Middle	Audience outdegree/total outdegree	-6	0.0005097	0.0025512	-0.0058452	0.0068646
Middle	Audience outdegree/total outdegree	-5	0.0019154	0.0015867	-0.0020369	0.0058678
Middle	Audience outdegree/total outdegree	-4	-0.0018353	0.0012193	-0.0048725	0.0012018
Middle	Audience outdegree/total outdegree	-3	-0.0015500	0.0017569	-0.0059264	0.0028263
Middle	Audience outdegree/total outdegree	-2	0.0010818	0.0016463	-0.0030191	0.0051827
Middle	Audience outdegree/total outdegree	-1	0.0005258	0.0018692	-0.0041302	0.0051819
Middle	Audience outdegree/total outdegree	0	0.1674511	0.1634939	-0.2398047	0.5747069
Middle	Audience outdegree/total outdegree	1	-0.0033867	0.0032802	-0.0115574	0.0047841
Middle	Audience outdegree/total outdegree	2	-0.0071224	0.0033597	-0.0154912	0.0012464
Middle	Audience outdegree/total outdegree	3	-0.0044427	0.0026973	-0.0111617	0.0022763
Middle	Audience outdegree/total outdegree	4	-0.0026761	0.0016133	-0.0066946	0.0013425
Middle	Audience outdegree/total outdegree	5	-0.0011758	0.0016443	-0.0052717	0.0029201
Middle	Audience outdegree/total outdegree	6	-0.0042308	0.0027773	-0.0111490	0.0026874
Middle	Audience outdegree/total outdegree	7	0.0010055	0.0016615	-0.0031332	0.0051442
Middle	Audience outdegree/total outdegree	8	-0.0031238	0.0028137	-0.0101326	0.0038849
Middle	Audience outdegree/total outdegree	9	-0.0063456	0.0026572	-0.0129646	0.0002733
Middle	Audience outdegree/total outdegree	10	-0.0056173	0.0019782	-0.0105449	-0.0006897
Middle	Audience outdegree/total outdegree	11	-0.0027328	0.0011496	-0.0055964	0.0001308
Middle	Audience outdegree/total outdegree	12	-0.0034191	0.0027750	-0.0103314	0.0034932
Middle	Audience outdegree/total outdegree	13	-0.0067031	0.0027147	-0.0134652	0.0000591
Middle	Audience outdegree/total outdegree	14	0.0009141	0.0014649	-0.0027349	0.0045631
Middle	Audience outdegree/total outdegree	15	-0.0051826	0.0020216	-0.0102183	-0.0001469
Middle	Audience outdegree/total outdegree	16	0.0013230	0.0033671	-0.0070643	0.0097102
Middle	Audience outdegree/total outdegree	17	-0.0032383	0.0047006	-0.0149472	0.0084706
Middle	Audience outdegree/total outdegree	18	0.0018935	0.0066903	-0.0147716	0.0185587
Middle	Audience outdegree/total outdegree	19	-0.0015816	0.0064288	-0.0175956	0.0144324
Middle	Audience outdegree/total outdegree	20	0.0042035	0.0093004	-0.0189634	0.0273705
Middle	Audience outdegree/total outdegree	21	-0.0019179	0.0042962	-0.0126195	0.0087837
Middle	Audience outdegree/total outdegree	22	0.0101584	0.0063637	-0.0056934	0.0260101
Middle	Audience outdegree/total outdegree	23	-0.0016946	0.0061496	-0.0170131	0.0136238
Middle	Audience outdegree/total outdegree	24	0.0036004	0.0038290	-0.0059374	0.0131383
Middle	Audience outdegree/total outdegree	25	-0.0025140	0.0064375	-0.0185496	0.0135215
Middle	Audience outdegree/total outdegree	26	-0.0043367	0.0025882	-0.0107839	0.0021104

Middle	Audience outdegree/total outdegree	27	-0.0046634	0.0037263	-0.0139455	0.0046186
Middle	Audience outdegree/total outdegree	28	-0.0011730	0.0052114	-0.0141544	0.0118084
Middle	Audience outdegree/total outdegree	29	-0.0003892	0.0034956	-0.0090966	0.0083181
Middle	Audience outdegree/total outdegree	30	-0.0036597	0.0027513	-0.0105131	0.0031937

sample	outcome	event.time	estimate	std.error	conf.low	conf.high
Least	Audience outdegree/total outdegree	-30	0.0020425	0.0017174	-0.0022997	0.0063847
Least	Audience outdegree/total outdegree	-29	-0.0062517	0.0016869	-0.0105169	-0.0019865
Least	Audience outdegree/total outdegree	-28	-0.0009848	0.0016250	-0.0050934	0.0031238
Least	Audience outdegree/total outdegree	-27	0.0054880	0.0016055	0.0014287	0.0095473
Least	Audience outdegree/total outdegree	-26	-0.1312537	0.1467143	-0.5022057	0.2396982
Least	Audience outdegree/total outdegree	-25	0.1291651	0.1475898	-0.2440005	0.5023306
Least	Audience outdegree/total outdegree	-24	0.0014435	0.0016338	-0.0026874	0.0055744
Least	Audience outdegree/total outdegree	-23	-0.0057674	0.0016207	-0.0098651	-0.0016697
Least	Audience outdegree/total outdegree	-22	0.0069945	0.0011663	0.0040457	0.0099433
Least	Audience outdegree/total outdegree	-21	-0.0059957	0.0022923	-0.0117915	-0.0001998
Least	Audience outdegree/total outdegree	-20	0.0010077	0.0014104	-0.0025583	0.0045737
Least	Audience outdegree/total outdegree	-19	-0.0039491	0.0010369	-0.0065708	-0.0013274
Least	Audience outdegree/total outdegree	-18	0.0000050	0.0012530	-0.0031630	0.0031730
Least	Audience outdegree/total outdegree	-17	0.0012229	0.0020239	-0.0038943	0.0063400
Least	Audience outdegree/total outdegree	-16	-0.0031879	0.0027494	-0.0101394	0.0037636
Least	Audience outdegree/total outdegree	-15	0.0030077	0.0013861	-0.0004969	0.0065122
Least	Audience outdegree/total outdegree	-14	-0.0040859	0.0010303	-0.0066909	-0.0014809
Least	Audience outdegree/total outdegree	-13	0.1583110	0.1746688	-0.2833211	0.5999430
Least	Audience outdegree/total outdegree	-12	-0.1608497	0.1737992	-0.6002828	0.2785835
Least	Audience outdegree/total outdegree	-11	0.0033221	0.0010214	0.0007397	0.0059045
Least	Audience outdegree/total outdegree	-10	-0.0029374	0.0008134	-0.0049940	-0.0008809
Least	Audience outdegree/total outdegree	-9	-0.0173053	0.0218536	-0.0725600	0.0379493
Least	Audience outdegree/total outdegree	-8	0.0183159	0.0217766	-0.0367441	0.0733759
Least	Audience outdegree/total outdegree	-7	-0.0045738	0.0022581	-0.0102832	0.0011356
Least	Audience outdegree/total outdegree	-6	-0.0005546	0.0024546	-0.0067608	0.0056516
Least	Audience outdegree/total outdegree	-5	0.0012719	0.0011001	-0.0015096	0.0040534
Least	Audience outdegree/total outdegree	-4	0.0003391	0.0014292	-0.0032745	0.0039526
Least	Audience outdegree/total outdegree	-3	-0.0001908	0.0013488	-0.0036011	0.0032194
Least	Audience outdegree/total outdegree	-2	-0.0001313	0.0010680	-0.0028315	0.0025690
Least	Audience outdegree/total outdegree	-1	-0.0003986	0.0015673	-0.0043613	0.0035641
Least	Audience outdegree/total outdegree	0	0.1546419	0.1748957	-0.2875638	0.5968475
Least	Audience outdegree/total outdegree	1	-0.0030864	0.0020667	-0.0083120	0.0021391
Least	Audience outdegree/total outdegree	2	-0.0029502	0.0013065	-0.0062535	0.0003531
Least	Audience outdegree/total outdegree	3	-0.0026904	0.0015252	-0.0065467	0.0011658
Least	Audience outdegree/total outdegree	4	-0.0032953	0.0023479	-0.0092317	0.0026411

Least	Audience outdegree/total outdegree	5	0.0004825	0.0018032	-0.0040768	0.0050418
Least	Audience outdegree/total outdegree	6	-0.0017860	0.0023957	-0.0078432	0.0042712
Least	Audience outdegree/total outdegree	7	-0.0039994	0.0024116	-0.0100968	0.0020980
Least	Audience outdegree/total outdegree	8	-0.0036165	0.0015092	-0.0074323	0.0001992
Least	Audience outdegree/total outdegree	9	-0.0054258	0.0015042	-0.0092291	-0.0016224
Least	Audience outdegree/total outdegree	10	-0.0038057	0.0022443	-0.0094802	0.0018688
Least	Audience outdegree/total outdegree	11	-0.0024657	0.0014209	-0.0060584	0.0011269
Least	Audience outdegree/total outdegree	12	-0.0038252	0.0021466	-0.0092526	0.0016022
Least	Audience outdegree/total outdegree	13	-0.0064945	0.0028571	-0.0137183	0.0007293
Least	Audience outdegree/total outdegree	14	-0.0096609	0.0018019	-0.0142168	-0.0051050
Least	Audience outdegree/total outdegree	15	-0.0096299	0.0053974	-0.0232767	0.0040168
Least	Audience outdegree/total outdegree	16	-0.0074237	0.0017976	-0.0119688	-0.0028787
Least	Audience outdegree/total outdegree	17	-0.0140518	0.0033702	-0.0225731	-0.0055305
Least	Audience outdegree/total outdegree	18	-0.0089118	0.0028974	-0.0162376	-0.0015859
Least	Audience outdegree/total outdegree	19	-0.0123616	0.0033288	-0.0207782	-0.0039451
Least	Audience outdegree/total outdegree	20	-0.0115493	0.0027318	-0.0184564	-0.0046422
Least	Audience outdegree/total outdegree	21	-0.0097239	0.0021028	-0.0150406	-0.0044072
Least	Audience outdegree/total outdegree	22	-0.0091731	0.0013949	-0.0126998	-0.0056463
Least	Audience outdegree/total outdegree	23	-0.0093840	0.0041844	-0.0199639	0.0011959
Least	Audience outdegree/total outdegree	24	-0.0164109	0.0038047	-0.0260308	-0.0067910
Least	Audience outdegree/total outdegree	25	-0.0136969	0.0030737	-0.0214685	-0.0059254
Least	Audience outdegree/total outdegree	26	-0.0149588	0.0046483	-0.0267115	-0.0032060
Least	Audience outdegree/total outdegree	27	-0.0139688	0.0025862	-0.0205078	-0.0074299
Least	Audience outdegree/total outdegree	28	-0.0174576	0.0068731	-0.0348355	-0.0000797
Least	Audience outdegree/total outdegree	29	-0.0138599	0.0068048	-0.0310653	0.0033454
Least	Audience outdegree/total outdegree	30	-0.0155527	0.0084120	-0.0368215	0.0057161

Audience indegree/total indegree

Average effect by length of exposure (Callaway and Sant'Anna)

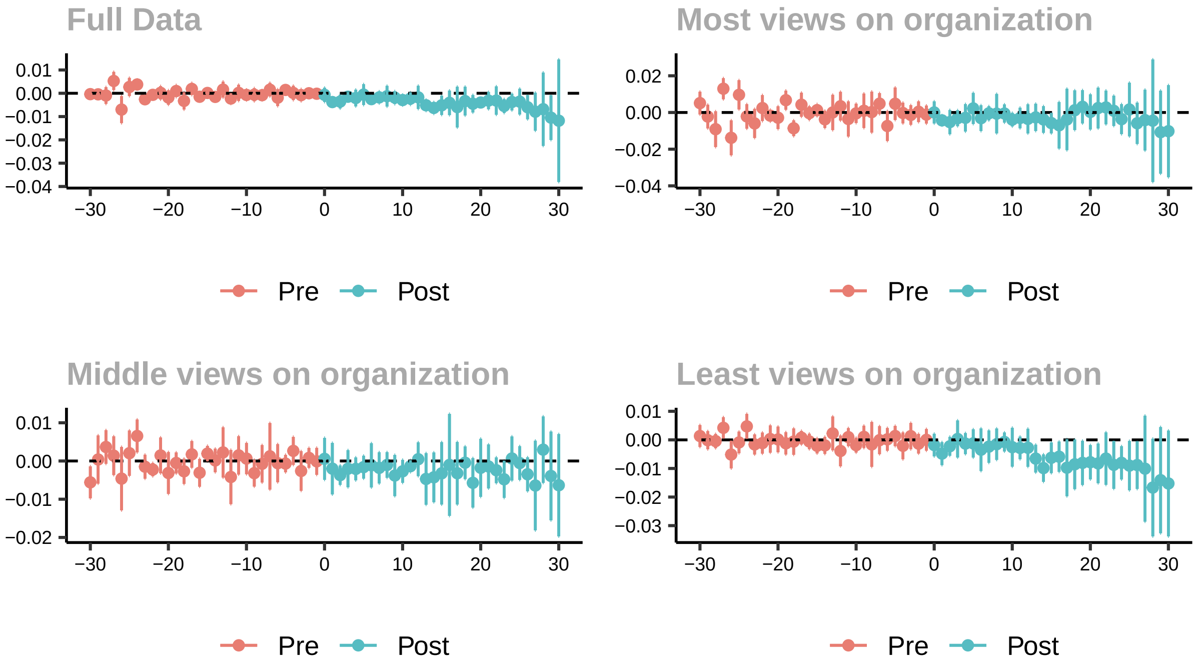

Long timeframe with sampled control groups

sample	outcome	event.time	estimate	std.error	conf.low	conf.high
Full	Audience indegree/total indegree	-30	-0.0003695	0.0005335	-0.0016935	0.0009545
Full	Audience indegree/total indegree	-29	-0.0004493	0.0005217	-0.0017441	0.0008455
Full	Audience indegree/total indegree	-28	-0.0009482	0.0014429	-0.0045292	0.0026328
Full	Audience indegree/total indegree	-27	0.0052742	0.0015710	0.0013752	0.0091731
Full	Audience indegree/total indegree	-26	-0.0070005	0.0023434	-0.0128164	-0.0011845
Full	Audience indegree/total indegree	-25	0.0026972	0.0015765	-0.0012156	0.0066099
Full	Audience indegree/total indegree	-24	0.0037815	0.0008342	0.0017110	0.0058519
Full	Audience indegree/total indegree	-23	-0.0025734	0.0007643	-0.0044704	-0.0006763
Full	Audience indegree/total indegree	-22	-0.0006681	0.0005198	-0.0019583	0.0006221
Full	Audience indegree/total indegree	-21	0.0001885	0.0011658	-0.0027049	0.0030819
Full	Audience indegree/total indegree	-20	-0.0018150	0.0012532	-0.0049253	0.0012952
Full	Audience indegree/total indegree	-19	0.0009568	0.0010419	-0.0016291	0.0035427
Full	Audience indegree/total indegree	-18	-0.0032768	0.0014976	-0.0069936	0.0004399
Full	Audience indegree/total indegree	-17	0.0018522	0.0010492	-0.0007518	0.0044562
Full	Audience indegree/total indegree	-16	-0.0014890	0.0005626	-0.0028854	-0.0000927
Full	Audience indegree/total indegree	-15	0.0001358	0.0004667	-0.0010226	0.0012942
Full	Audience indegree/total indegree	-14	-0.0015438	0.0006297	-0.0031066	0.0000191
Full	Audience indegree/total indegree	-13	0.0015915	0.0013854	-0.0018469	0.0050299
Full	Audience indegree/total indegree	-12	-0.0022448	0.0009239	-0.0045378	0.0000482

Full	Audience indegree/total indegree	-11	0.0001791	0.0013976	-0.0032895	0.0036477
Full	Audience indegree/total indegree	-10	-0.0007089	0.0009329	-0.0030243	0.0016065
Full	Audience indegree/total indegree	-9	-0.0006105	0.0011341	-0.0034252	0.0022043
Full	Audience indegree/total indegree	-8	-0.0008131	0.0008737	-0.0029816	0.0013555
Full	Audience indegree/total indegree	-7	0.0015400	0.0011852	-0.0014016	0.0044816
Full	Audience indegree/total indegree	-6	-0.0018961	0.0014558	-0.0055091	0.0017170
Full	Audience indegree/total indegree	-5	0.0014285	0.0007297	-0.0003826	0.0032397
Full	Audience indegree/total indegree	-4	0.0001444	0.0012394	-0.0029316	0.0032204
Full	Audience indegree/total indegree	-3	-0.0007844	0.0010521	-0.0033956	0.0018267
Full	Audience indegree/total indegree	-2	0.0000066	0.0008132	-0.0020116	0.0020249
Full	Audience indegree/total indegree	-1	-0.0001948	0.0007620	-0.0020859	0.0016963
Full	Audience indegree/total indegree	0	-0.0006832	0.0012346	-0.0037475	0.0023810
Full	Audience indegree/total indegree	1	-0.0037674	0.0007961	-0.0057432	-0.0017917
Full	Audience indegree/total indegree	2	-0.0035694	0.0011875	-0.0065166	-0.0006222
Full	Audience indegree/total indegree	3	-0.0014714	0.0007985	-0.0034533	0.0005104
Full	Audience indegree/total indegree	4	-0.0020294	0.0014421	-0.0056084	0.0015496
Full	Audience indegree/total indegree	5	-0.0005667	0.0017981	-0.0050294	0.0038959
Full	Audience indegree/total indegree	6	-0.0025156	0.0006593	-0.0041518	-0.0008793
Full	Audience indegree/total indegree	7	-0.0017621	0.0010348	-0.0043303	0.0008061
Full	Audience indegree/total indegree	8	-0.0012607	0.0017964	-0.0057192	0.0031977
Full	Audience indegree/total indegree	9	-0.0018637	0.0011207	-0.0046450	0.0009177
Full	Audience indegree/total indegree	10	-0.0029021	0.0009313	-0.0052134	-0.0005907
Full	Audience indegree/total indegree	11	-0.0022807	0.0010834	-0.0049697	0.0004082
Full	Audience indegree/total indegree	12	-0.0017487	0.0020045	-0.0067236	0.0032262
Full	Audience indegree/total indegree	13	-0.0051501	0.0009714	-0.0075611	-0.0027392
Full	Audience indegree/total indegree	14	-0.0062847	0.0010200	-0.0088163	-0.0037531
Full	Audience indegree/total indegree	15	-0.0051656	0.0016560	-0.0092756	-0.0010556
Full	Audience indegree/total indegree	16	-0.0040962	0.0021281	-0.0093779	0.0011855
Full	Audience indegree/total indegree	17	-0.0059358	0.0035249	-0.0146841	0.0028126
Full	Audience indegree/total indegree	18	-0.0033609	0.0024903	-0.0095414	0.0028196
Full	Audience indegree/total indegree	19	-0.0044947	0.0015171	-0.0082600	-0.0007294
Full	Audience indegree/total indegree	20	-0.0039487	0.0007667	-0.0058516	-0.0020459
Full	Audience indegree/total indegree	21	-0.0031397	0.0014917	-0.0068418	0.0005625
Full	Audience indegree/total indegree	22	-0.0031263	0.0024502	-0.0092073	0.0029547
Full	Audience indegree/total indegree	23	-0.0054561	0.0012108	-0.0084612	-0.0024510
Full	Audience indegree/total indegree	24	-0.0037539	0.0014465	-0.0073440	-0.0001637
Full	Audience indegree/total indegree	25	-0.0035876	0.0022306	-0.0091238	0.0019485
Full	Audience indegree/total indegree	26	-0.0059789	0.0020835	-0.0111498	-0.0008080
Full	Audience indegree/total indegree	27	-0.0079053	0.0032875	-0.0160644	0.0002538
Full	Audience indegree/total indegree	28	-0.0068234	0.0063388	-0.0225556	0.0089088
Full	Audience indegree/total indegree	29	-0.0104583	0.0038604	-0.0200395	-0.0008772

Full	Audience indegree/total indegree	30	-0.0117614	0.0105934	-0.0380530	0.0145301

sample	outcome	event.time	estimate	std.error	conf.low	conf.high
Most	Audience indegree/total indegree	-30	0.0051061	0.0024623	-0.0011087	0.0113209
Most	Audience indegree/total indegree	-29	-0.0022227	0.0025584	-0.0086800	0.0042347
Most	Audience indegree/total indegree	-28	-0.0091023	0.0038574	-0.0188382	0.0006336
Most	Audience indegree/total indegree	-27	0.0129531	0.0022757	0.0072093	0.0186968
Most	Audience indegree/total indegree	-26	-0.0138073	0.0037653	-0.0233108	-0.0043039
Most	Audience indegree/total indegree	-25	0.0096368	0.0031571	0.0016682	0.0176054
Most	Audience indegree/total indegree	-24	-0.0021528	0.0026456	-0.0088304	0.0045247
Most	Audience indegree/total indegree	-23	-0.0059585	0.0031382	-0.0138793	0.0019624
Most	Audience indegree/total indegree	-22	0.0024013	0.0028092	-0.0046891	0.0094918
Most	Audience indegree/total indegree	-21	-0.0017251	0.0012882	-0.0049764	0.0015263
Most	Audience indegree/total indegree	-20	-0.0028415	0.0024598	-0.0090501	0.0033671
Most	Audience indegree/total indegree	-19	0.0067133	0.0020673	0.0014956	0.0119311
Most	Audience indegree/total indegree	-18	-0.0086060	0.0017061	-0.0129123	-0.0042998
Most	Audience indegree/total indegree	-17	0.0042448	0.0025967	-0.0023092	0.0107988
Most	Audience indegree/total indegree	-16	-0.0003576	0.0014704	-0.0040688	0.0033537
Most	Audience indegree/total indegree	-15	0.0012618	0.0013192	-0.0020679	0.0045915
Most	Audience indegree/total indegree	-14	-0.0037223	0.0018729	-0.0084495	0.0010049
Most	Audience indegree/total indegree	-13	0.0000102	0.0038159	-0.0096212	0.0096416
Most	Audience indegree/total indegree	-12	0.0034154	0.0031078	-0.0044286	0.0112593
Most	Audience indegree/total indegree	-11	-0.0035499	0.0037996	-0.0131401	0.0060403
Most	Audience indegree/total indegree	-10	-0.0004834	0.0020931	-0.0057664	0.0047995
Most	Audience indegree/total indegree	-9	0.0009407	0.0037245	-0.0084599	0.0103413
Most	Audience indegree/total indegree	-8	0.0002076	0.0044377	-0.0109930	0.0114082
Most	Audience indegree/total indegree	-7	0.0048206	0.0022779	-0.0009287	0.0105698
Most	Audience indegree/total indegree	-6	-0.0073662	0.0032330	-0.0155263	0.0007939
Most	Audience indegree/total indegree	-5	0.0047680	0.0035097	-0.0040904	0.0136263
Most	Audience indegree/total indegree	-4	-0.0003029	0.0022324	-0.0059373	0.0053315
Most	Audience indegree/total indegree	-3	-0.0013527	0.0021162	-0.0066939	0.0039886
Most	Audience indegree/total indegree	-2	0.0004036	0.0022390	-0.0052476	0.0060548
Most	Audience indegree/total indegree	-1	-0.0011416	0.0019529	-0.0060706	0.0037874
Most	Audience indegree/total indegree	0	0.0000297	0.0023864	-0.0059936	0.0060530
Most	Audience indegree/total indegree	1	-0.0042820	0.0011059	-0.0070733	-0.0014906
Most	Audience indegree/total indegree	2	-0.0052479	0.0026116	-0.0118396	0.0013438
Most	Audience indegree/total indegree	3	-0.0031108	0.0017300	-0.0074773	0.0012558
Most	Audience indegree/total indegree	4	-0.0029043	0.0029699	-0.0104004	0.0045918
Most	Audience indegree/total indegree	5	0.0022114	0.0033566	-0.0062605	0.0106833
Most	Audience indegree/total indegree	6	-0.0030977	0.0027858	-0.0101290	0.0039335
Most	Audience indegree/total indegree	7	-0.0004435	0.0015284	-0.0043012	0.0034142

Most	Audience indegree/total indegree	8	-0.0006308	0.0042594	-0.0113814	0.0101197
Most	Audience indegree/total indegree	9	-0.0005680	0.0019726	-0.0055469	0.0044109
Most	Audience indegree/total indegree	10	-0.0038992	0.0014652	-0.0075974	-0.0002010
Most	Audience indegree/total indegree	11	-0.0029937	0.0022293	-0.0086203	0.0026330
Most	Audience indegree/total indegree	12	-0.0034813	0.0031406	-0.0114082	0.0044457
Most	Audience indegree/total indegree	13	-0.0026731	0.0029425	-0.0100998	0.0047536
Most	Audience indegree/total indegree	14	-0.0038122	0.0028773	-0.0110744	0.0034501
Most	Audience indegree/total indegree	15	-0.0057754	0.0022217	-0.0113829	-0.0001679
Most	Audience indegree/total indegree	16	-0.0069720	0.0050263	-0.0196582	0.0057142
Most	Audience indegree/total indegree	17	-0.0038157	0.0066339	-0.0205595	0.0129281
Most	Audience indegree/total indegree	18	0.0012263	0.0042933	-0.0096099	0.0120626
Most	Audience indegree/total indegree	19	0.0031064	0.0035895	-0.0059535	0.0121662
Most	Audience indegree/total indegree	20	0.0002960	0.0037965	-0.0092862	0.0098782
Most	Audience indegree/total indegree	21	0.0024229	0.0044025	-0.0086890	0.0135349
Most	Audience indegree/total indegree	22	0.0027593	0.0036427	-0.0064349	0.0119535
Most	Audience indegree/total indegree	23	0.0009237	0.0032663	-0.0073203	0.0091678
Most	Audience indegree/total indegree	24	-0.0036129	0.0032465	-0.0118071	0.0045813
Most	Audience indegree/total indegree	25	0.0016192	0.0057867	-0.0129864	0.0162248
Most	Audience indegree/total indegree	26	-0.0058745	0.0044632	-0.0171395	0.0053904
Most	Audience indegree/total indegree	27	-0.0041605	0.0065660	-0.0207330	0.0124120
Most	Audience indegree/total indegree	28	-0.0044707	0.0132311	-0.0378657	0.0289243
Most	Audience indegree/total indegree	29	-0.0107222	0.0089740	-0.0333724	0.0119279
Most	Audience indegree/total indegree	30	-0.0101957	0.0099398	-0.0352834	0.0148921

sample	outcome	event.time	estimate	std.error	conf.low	conf.high
Middle	Audience indegree/total indegree	-30	-0.0055885	0.0015895	-0.0097835	-0.0013935
Middle	Audience indegree/total indegree	-29	0.0003933	0.0023888	-0.0059112	0.0066978
Middle	Audience indegree/total indegree	-28	0.0036654	0.0016659	-0.0007312	0.0080620
Middle	Audience indegree/total indegree	-27	0.0013965	0.0019218	-0.0036755	0.0064685
Middle	Audience indegree/total indegree	-26	-0.0046130	0.0031406	-0.0129018	0.0036757
Middle	Audience indegree/total indegree	-25	0.0020461	0.0022454	-0.0038800	0.0079721
Middle	Audience indegree/total indegree	-24	0.0065454	0.0016276	0.0022497	0.0108411
Middle	Audience indegree/total indegree	-23	-0.0014896	0.0011863	-0.0046204	0.0016413
Middle	Audience indegree/total indegree	-22	-0.0022471	0.0006590	-0.0039864	-0.0005077
Middle	Audience indegree/total indegree	-21	0.0014518	0.0017774	-0.0032392	0.0061428
Middle	Audience indegree/total indegree	-20	-0.0031422	0.0020732	-0.0086138	0.0023294
Middle	Audience indegree/total indegree	-19	-0.0005100	0.0010461	-0.0032708	0.0022508
Middle	Audience indegree/total indegree	-18	-0.0027009	0.0012526	-0.0060067	0.0006049
Middle	Audience indegree/total indegree	-17	0.0017301	0.0013201	-0.0017540	0.0052141
Middle	Audience indegree/total indegree	-16	-0.0030764	0.0013784	-0.0067143	0.0005615
Middle	Audience indegree/total indegree	-15	0.0019334	0.0007691	-0.0000965	0.0039632

Middle	Audience indegree/total indegree	-14	0.0001420	0.0011697	-0.0029450	0.0032290
Middle	Audience indegree/total indegree	-13	0.0022434	0.0024903	-0.0043292	0.0088159
Middle	Audience indegree/total indegree	-12	-0.0041856	0.0026895	-0.0112836	0.0029124
Middle	Audience indegree/total indegree	-11	0.0014691	0.0018979	-0.0035399	0.0064782
Middle	Audience indegree/total indegree	-10	0.0006329	0.0015491	-0.0034556	0.0047214
Middle	Audience indegree/total indegree	-9	-0.0031778	0.0013300	-0.0066880	0.0003324
Middle	Audience indegree/total indegree	-8	-0.0007355	0.0018544	-0.0056296	0.0041587
Middle	Audience indegree/total indegree	-7	0.0012317	0.0032993	-0.0074759	0.0099393
Middle	Audience indegree/total indegree	-6	-0.0005761	0.0018865	-0.0055550	0.0044029
Middle	Audience indegree/total indegree	-5	-0.0006788	0.0008691	-0.0029725	0.0016148
Middle	Audience indegree/total indegree	-4	0.0026417	0.0013935	-0.0010360	0.0063193
Middle	Audience indegree/total indegree	-3	-0.0026085	0.0019649	-0.0077943	0.0025772
Middle	Audience indegree/total indegree	-2	0.0008116	0.0009908	-0.0018034	0.0034267
Middle	Audience indegree/total indegree	-1	-0.0000842	0.0013391	-0.0036184	0.0034501
Middle	Audience indegree/total indegree	0	0.0005645	0.0020793	-0.0049231	0.0060522
Middle	Audience indegree/total indegree	1	-0.0020077	0.0025539	-0.0087479	0.0047324
Middle	Audience indegree/total indegree	2	-0.0036983	0.0009688	-0.0062550	-0.0011415
Middle	Audience indegree/total indegree	3	-0.0020212	0.0018407	-0.0068791	0.0028368
Middle	Audience indegree/total indegree	4	-0.0020708	0.0011389	-0.0050766	0.0009350
Middle	Audience indegree/total indegree	5	-0.0016114	0.0011327	-0.0046007	0.0013779
Middle	Audience indegree/total indegree	6	-0.0011368	0.0021925	-0.0069233	0.0046496
Middle	Audience indegree/total indegree	7	-0.0017970	0.0015490	-0.0058851	0.0022911
Middle	Audience indegree/total indegree	8	-0.0010461	0.0012733	-0.0044067	0.0023145
Middle	Audience indegree/total indegree	9	-0.0038034	0.0020429	-0.0091951	0.0015882
Middle	Audience indegree/total indegree	10	-0.0027038	0.0011424	-0.0057189	0.0003113
Middle	Audience indegree/total indegree	11	-0.0013550	0.0005984	-0.0029342	0.0002242
Middle	Audience indegree/total indegree	12	0.0004647	0.0016775	-0.0039625	0.0048920
Middle	Audience indegree/total indegree	13	-0.0046915	0.0025554	-0.0114358	0.0020527
Middle	Audience indegree/total indegree	14	-0.0042688	0.0024666	-0.0107787	0.0022411
Middle	Audience indegree/total indegree	15	-0.0032293	0.0030907	-0.0113864	0.0049277
Middle	Audience indegree/total indegree	16	-0.0010202	0.0050556	-0.0143630	0.0123227
Middle	Audience indegree/total indegree	17	-0.0032034	0.0030970	-0.0113770	0.0049703
Middle	Audience indegree/total indegree	18	-0.0004498	0.0016212	-0.0047284	0.0038289
Middle	Audience indegree/total indegree	19	-0.0057148	0.0024515	-0.0121849	0.0007553
Middle	Audience indegree/total indegree	20	-0.0017821	0.0028796	-0.0093821	0.0058179
Middle	Audience indegree/total indegree	21	-0.0014262	0.0021808	-0.0071817	0.0043293
Middle	Audience indegree/total indegree	22	-0.0024165	0.0012795	-0.0057934	0.0009605
Middle	Audience indegree/total indegree	23	-0.0047858	0.0018457	-0.0096569	0.0000853
Middle	Audience indegree/total indegree	24	0.0006554	0.0021854	-0.0051123	0.0064232
Middle	Audience indegree/total indegree	25	-0.0005380	0.0016726	-0.0049524	0.0038763
Middle	Audience indegree/total indegree	26	-0.0034751	0.0016853	-0.0079230	0.0009729

Middle	Audience indegree/total indegree	27	-0.0064209	0.0044436	-0.0181486	0.0053067
Middle	Audience indegree/total indegree	28	0.0029613	0.0032963	-0.0057383	0.0116608
Middle	Audience indegree/total indegree	29	-0.0039188	0.0044009	-0.0155336	0.0076960
Middle	Audience indegree/total indegree	30	-0.0063445	0.0050717	-0.0197297	0.0070406

sample	outcome	event.time	estimate	std.error	conf.low	conf.high
Least	Audience indegree/total indegree	-30	0.0013526	0.0014967	-0.0024833	0.0051884
Least	Audience indegree/total indegree	-29	-0.0001141	0.0012399	-0.0032918	0.0030636
Least	Audience indegree/total indegree	-28	-0.0004201	0.0009676	-0.0028999	0.0020597
Least	Audience indegree/total indegree	-27	0.0042203	0.0014517	0.0004997	0.0079408
Least	Audience indegree/total indegree	-26	-0.0051298	0.0018916	-0.0099777	-0.0002819
Least	Audience indegree/total indegree	-25	-0.0009033	0.0014812	-0.0046995	0.0028928
Least	Audience indegree/total indegree	-24	0.0047289	0.0017027	0.0003650	0.0090927
Least	Audience indegree/total indegree	-23	-0.0015951	0.0013741	-0.0051167	0.0019266
Least	Audience indegree/total indegree	-22	-0.0011221	0.0014358	-0.0048018	0.0025577
Least	Audience indegree/total indegree	-21	0.0003021	0.0018604	-0.0044659	0.0050702
Least	Audience indegree/total indegree	-20	0.0001551	0.0017556	-0.0043444	0.0046546
Least	Audience indegree/total indegree	-19	-0.0012120	0.0015205	-0.0051090	0.0026850
Least	Audience indegree/total indegree	-18	-0.0005970	0.0017994	-0.0052088	0.0040147
Least	Audience indegree/total indegree	-17	0.0007629	0.0009338	-0.0016303	0.0031560
Least	Audience indegree/total indegree	-16	-0.0005399	0.0010546	-0.0032429	0.0021630
Least	Audience indegree/total indegree	-15	-0.0020667	0.0010484	-0.0047537	0.0006202
Least	Audience indegree/total indegree	-14	-0.0019211	0.0011678	-0.0049141	0.0010719
Least	Audience indegree/total indegree	-13	0.0022658	0.0023162	-0.0036704	0.0082020
Least	Audience indegree/total indegree	-12	-0.0038777	0.0020989	-0.0092571	0.0015017
Least	Audience indegree/total indegree	-11	0.0009667	0.0012309	-0.0021880	0.0041215
Least	Audience indegree/total indegree	-10	-0.0018441	0.0010110	-0.0044351	0.0007469
Least	Audience indegree/total indegree	-9	0.0010523	0.0015236	-0.0028525	0.0049570
Least	Audience indegree/total indegree	-8	-0.0015863	0.0030641	-0.0094394	0.0062668
Least	Audience indegree/total indegree	-7	-0.0000989	0.0019101	-0.0049944	0.0047965
Least	Audience indegree/total indegree	-6	0.0001914	0.0015465	-0.0037722	0.0041550
Least	Audience indegree/total indegree	-5	0.0013593	0.0013837	-0.0021869	0.0049055
Least	Audience indegree/total indegree	-4	-0.0020741	0.0018386	-0.0067863	0.0026380
Least	Audience indegree/total indegree	-3	0.0013135	0.0018002	-0.0033002	0.0059273
Least	Audience indegree/total indegree	-2	-0.0013314	0.0013452	-0.0047791	0.0021162
Least	Audience indegree/total indegree	-1	0.0000185	0.0014743	-0.0037600	0.0037971
Least	Audience indegree/total indegree	0	-0.0018135	0.0015469	-0.0057780	0.0021511
Least	Audience indegree/total indegree	1	-0.0048050	0.0015694	-0.0088271	-0.0007829
Least	Audience indegree/total indegree	2	-0.0022392	0.0012959	-0.0055605	0.0010822
Least	Audience indegree/total indegree	3	0.0003159	0.0025319	-0.0061730	0.0068048
Least	Audience indegree/total indegree	4	-0.0013042	0.0014365	-0.0049858	0.0023774

Least	Audience indegree/total indegree	5	-0.0012275	0.0019273	-0.0061670	0.0037120
Least	Audience indegree/total indegree	6	-0.0034393	0.0029099	-0.0108971	0.0040185
Least	Audience indegree/total indegree	7	-0.0023635	0.0022098	-0.0080270	0.0033000
Least	Audience indegree/total indegree	8	-0.0015165	0.0021426	-0.0070077	0.0039748
Least	Audience indegree/total indegree	9	-0.0007305	0.0012976	-0.0040562	0.0025951
Least	Audience indegree/total indegree	10	-0.0025570	0.0026531	-0.0093566	0.0042426
Least	Audience indegree/total indegree	11	-0.0028097	0.0015429	-0.0067640	0.0011445
Least	Audience indegree/total indegree	12	-0.0027621	0.0025965	-0.0094166	0.0038924
Least	Audience indegree/total indegree	13	-0.0065489	0.0019007	-0.0114203	-0.0016776
Least	Audience indegree/total indegree	14	-0.0098658	0.0018782	-0.0146795	-0.0050522
Least	Audience indegree/total indegree	15	-0.0062595	0.0020979	-0.0116361	-0.0008828
Least	Audience indegree/total indegree	16	-0.0058868	0.0020703	-0.0111929	-0.0005808
Least	Audience indegree/total indegree	17	-0.0097315	0.0038909	-0.0197036	0.0002406
Least	Audience indegree/total indegree	18	-0.0085443	0.0033877	-0.0172267	0.0001382
Least	Audience indegree/total indegree	19	-0.0080437	0.0030179	-0.0157783	-0.0003091
Least	Audience indegree/total indegree	20	-0.0078204	0.0023416	-0.0138218	-0.0018190
Least	Audience indegree/total indegree	21	-0.0082264	0.0027049	-0.0151587	-0.0012940
Least	Audience indegree/total indegree	22	-0.0065314	0.0035848	-0.0157190	0.0026562
Least	Audience indegree/total indegree	23	-0.0087323	0.0032719	-0.0171180	-0.0003466
Least	Audience indegree/total indegree	24	-0.0080291	0.0022704	-0.0138480	-0.0022103
Least	Audience indegree/total indegree	25	-0.0090028	0.0033657	-0.0176286	-0.0003769
Least	Audience indegree/total indegree	26	-0.0088023	0.0032644	-0.0171686	-0.0004360
Least	Audience indegree/total indegree	27	-0.0100687	0.0072276	-0.0285924	0.0084550
Least	Audience indegree/total indegree	28	-0.0166807	0.0066669	-0.0337673	0.0004060
Least	Audience indegree/total indegree	29	-0.0140903	0.0072449	-0.0326582	0.0044777
Least	Audience indegree/total indegree	30	-0.0152519	0.0072205	-0.0337573	0.0032534

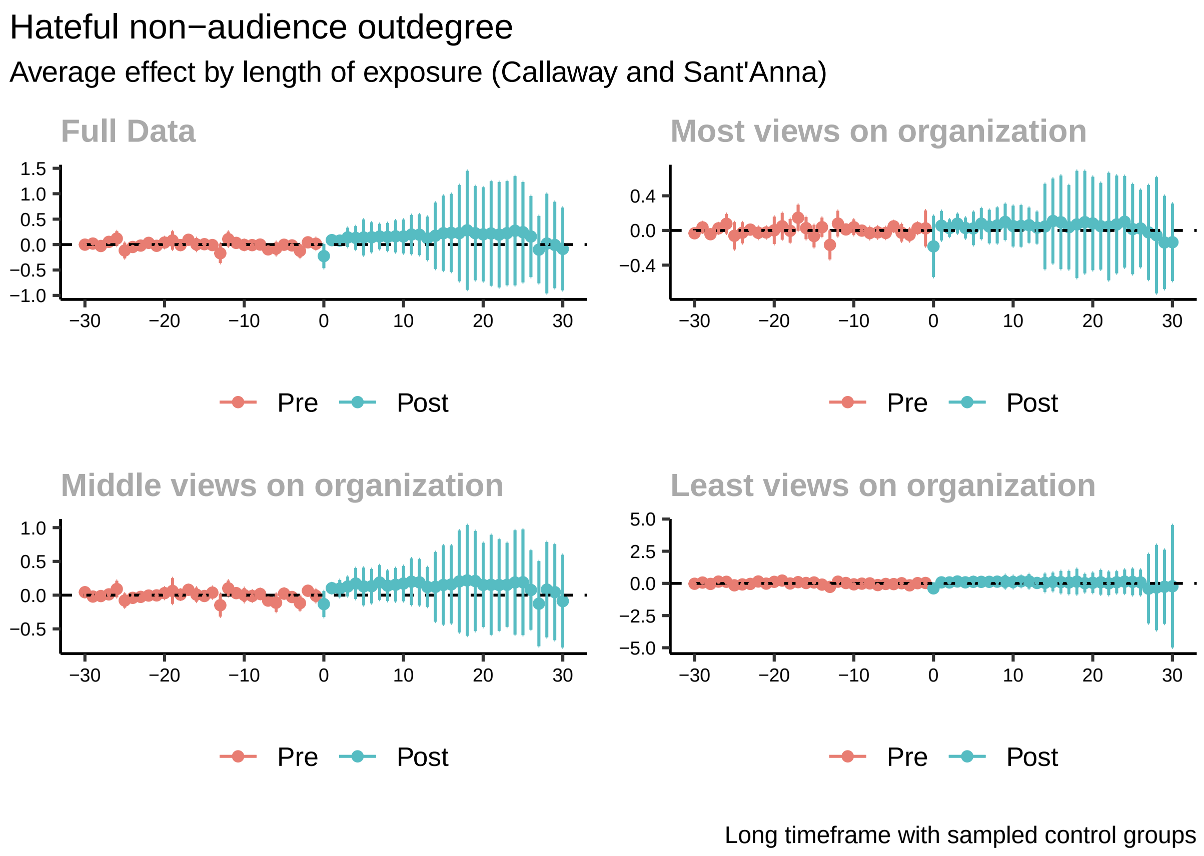

sample	outcome	event.time	estimate	std.error	conf.low	conf.high
Full	Hateful non-audience outdegree	-30	-0.0010026	0.0090371	-0.0241285	0.0221232
Full	Hateful non-audience outdegree	-29	0.0198691	0.0114786	-0.0095045	0.0492428
Full	Hateful non-audience outdegree	-28	-0.0277488	0.0146235	-0.0651704	0.0096728
Full	Hateful non-audience outdegree	-27	0.0536390	0.0181989	0.0070680	0.1002099
Full	Hateful non-audience outdegree	-26	0.1183808	0.0563204	-0.0257428	0.2625045
Full	Hateful non-audience outdegree	-25	-0.1167249	0.0600307	-0.2703432	0.0368933
Full	Hateful non-audience outdegree	-24	-0.0473248	0.0119598	-0.0779300	-0.0167196
Full	Hateful non-audience outdegree	-23	-0.0195043	0.0135616	-0.0542083	0.0151997
Full	Hateful non-audience outdegree	-22	0.0335431	0.0198120	-0.0171557	0.0842420
Full	Hateful non-audience outdegree	-21	-0.0243496	0.0304191	-0.1021920	0.0534928
Full	Hateful non-audience outdegree	-20	0.0399735	0.0414927	-0.0662062	0.1461532
Full	Hateful non-audience outdegree	-19	0.0806629	0.0703370	-0.0993294	0.2606552
Full	Hateful non-audience outdegree	-18	-0.0101040	0.0356213	-0.1012589	0.0810508
Full	Hateful non-audience outdegree	-17	0.0944849	0.0328102	0.0105236	0.1784461
Full	Hateful non-audience outdegree	-16	0.0029871	0.0489000	-0.1221478	0.1281219
Full	Hateful non-audience outdegree	-15	0.0077367	0.0248304	-0.0558043	0.0712777
Full	Hateful non-audience outdegree	-14	-0.0095350	0.0241436	-0.0713184	0.0522483
Full	Hateful non-audience outdegree	-13	-0.1705611	0.0747404	-0.3618216	0.0206994
Full	Hateful non-audience outdegree	-12	0.1074015	0.0573370	-0.0393236	0.2541267

Full	Hateful non-audience outdegree	-11	0.0330295	0.0207357	-0.0200332	0.0860922
Full	Hateful non-audience outdegree	-10	-0.0056527	0.0382161	-0.1034475	0.0921422
Full	Hateful non-audience outdegree	-9	-0.0103942	0.0251406	-0.0747288	0.0539403
Full	Hateful non-audience outdegree	-8	0.0001629	0.0324980	-0.0829994	0.0833253
Full	Hateful non-audience outdegree	-7	-0.0937808	0.0311036	-0.1733747	-0.0141868
Full	Hateful non-audience outdegree	-6	-0.0783988	0.0518488	-0.2110798	0.0542821
Full	Hateful non-audience outdegree	-5	-0.0032879	0.0221634	-0.0600040	0.0534281
Full	Hateful non-audience outdegree	-4	-0.0161436	0.0142462	-0.0525997	0.0203125
Full	Hateful non-audience outdegree	-3	-0.1240112	0.0527211	-0.2589243	0.0109018
Full	Hateful non-audience outdegree	-2	0.0443274	0.0162339	0.0027849	0.0858698
Full	Hateful non-audience outdegree	-1	0.0159246	0.0455678	-0.1006832	0.1325323
Full	Hateful non-audience outdegree	0	-0.2258392	0.0925410	-0.4626512	0.0109728
Full	Hateful non-audience outdegree	1	0.0875227	0.0290446	0.0131977	0.1618477
Full	Hateful non-audience outdegree	2	0.0783229	0.0360829	-0.0140130	0.1706588
Full	Hateful non-audience outdegree	3	0.1412158	0.0742533	-0.0487981	0.3312297
Full	Hateful non-audience outdegree	4	0.1304009	0.0904738	-0.1011212	0.3619230
Full	Hateful non-audience outdegree	5	0.1400046	0.1384409	-0.2142651	0.4942743
Full	Hateful non-audience outdegree	6	0.1432880	0.1148160	-0.1505258	0.4371019
Full	Hateful non-audience outdegree	7	0.1584017	0.0971625	-0.0902368	0.4070401
Full	Hateful non-audience outdegree	8	0.1505413	0.1066130	-0.1222811	0.4233637
Full	Hateful non-audience outdegree	9	0.1624769	0.1225313	-0.1510804	0.4760342
Full	Hateful non-audience outdegree	10	0.1592269	0.1294671	-0.1720790	0.4905327
Full	Hateful non-audience outdegree	11	0.1972649	0.1500986	-0.1868368	0.5813667
Full	Hateful non-audience outdegree	12	0.1941477	0.1564363	-0.2061724	0.5944678
Full	Hateful non-audience outdegree	13	0.1221645	0.1663188	-0.3034447	0.5477737
Full	Hateful non-audience outdegree	14	0.1736281	0.2543224	-0.4771821	0.8244384
Full	Hateful non-audience outdegree	15	0.2235521	0.2881849	-0.5139121	0.9610162
Full	Hateful non-audience outdegree	16	0.2302570	0.2993288	-0.5357245	0.9962384
Full	Hateful non-audience outdegree	17	0.2255165	0.3694399	-0.7198792	1.1709122
Full	Hateful non-audience outdegree	18	0.2805921	0.4567048	-0.8881140	1.4492983
Full	Hateful non-audience outdegree	19	0.2246571	0.3615287	-0.7004938	1.1498080
Full	Hateful non-audience outdegree	20	0.2009118	0.3614791	-0.7241120	1.1259356
Full	Hateful non-audience outdegree	21	0.2187991	0.4013798	-0.8083305	1.2459287
Full	Hateful non-audience outdegree	22	0.1945264	0.4053063	-0.8426512	1.2317040
Full	Hateful non-audience outdegree	23	0.2241848	0.3997401	-0.7987488	1.2471184
Full	Hateful non-audience outdegree	24	0.2723007	0.4194627	-0.8011030	1.3457044
Full	Hateful non-audience outdegree	25	0.2434047	0.3858144	-0.7438932	1.2307026
Full	Hateful non-audience outdegree	26	0.1582290	0.3115745	-0.6390892	0.9555472
Full	Hateful non-audience outdegree	27	-0.0989934	0.2588636	-0.7614245	0.5634376
Full	Hateful non-audience outdegree	28	0.0205928	0.3824831	-0.9581804	0.9993660
Full	Hateful non-audience outdegree	29	-0.0081501	0.3318237	-0.8572860	0.8409858

Full	Hateful non-audience outdegree	30	-0.0855155	0.3178017	-0.8987691	0.7277381

sample	outcome	event.time	estimate	std.error	conf.low	conf.high
Most	Hateful non-audience outdegree	-30	-0.0323929	0.0133021	-0.0663054	0.0015196
Most	Hateful non-audience outdegree	-29	0.0354313	0.0236749	-0.0249257	0.0957883
Most	Hateful non-audience outdegree	-28	-0.0432753	0.0138371	-0.0785517	-0.0079989
Most	Hateful non-audience outdegree	-27	0.0244407	0.0229715	-0.0341230	0.0830043
Most	Hateful non-audience outdegree	-26	0.0771240	0.0443447	-0.0359286	0.1901767
Most	Hateful non-audience outdegree	-25	-0.0614174	0.0613217	-0.2177514	0.0949167
Most	Hateful non-audience outdegree	-24	-0.0263325	0.0473146	-0.1469569	0.0942918
Most	Hateful non-audience outdegree	-23	0.0096062	0.0132046	-0.0240578	0.0432701
Most	Hateful non-audience outdegree	-22	-0.0266323	0.0275296	-0.0968164	0.0435518
Most	Hateful non-audience outdegree	-21	-0.0231238	0.0274059	-0.0929925	0.0467450
Most	Hateful non-audience outdegree	-20	0.0014607	0.0619135	-0.1563819	0.1593034
Most	Hateful non-audience outdegree	-19	0.0485011	0.0604851	-0.1057001	0.2027024
Most	Hateful non-audience outdegree	-18	-0.0039674	0.0536063	-0.1406317	0.1326968
Most	Hateful non-audience outdegree	-17	0.1468570	0.0586914	-0.0027713	0.2964853
Most	Hateful non-audience outdegree	-16	0.0269011	0.0500480	-0.1006918	0.1544939
Most	Hateful non-audience outdegree	-15	-0.0648394	0.0509488	-0.1947285	0.0650498
Most	Hateful non-audience outdegree	-14	0.0374254	0.0434017	-0.0732234	0.1480741
Most	Hateful non-audience outdegree	-13	-0.1662488	0.0654896	-0.3332085	0.0007108
Most	Hateful non-audience outdegree	-12	0.0791672	0.0579009	-0.0684459	0.2267803
Most	Hateful non-audience outdegree	-11	0.0118216	0.0144247	-0.0249528	0.0485960
Most	Hateful non-audience outdegree	-10	0.0372168	0.0354964	-0.0532780	0.1277115
Most	Hateful non-audience outdegree	-9	-0.0017540	0.0170133	-0.0451278	0.0416197
Most	Hateful non-audience outdegree	-8	-0.0295971	0.0274832	-0.0996631	0.0404689
Most	Hateful non-audience outdegree	-7	-0.0221689	0.0274332	-0.0921072	0.0477694
Most	Hateful non-audience outdegree	-6	-0.0285526	0.0262808	-0.0955530	0.0384478
Most	Hateful non-audience outdegree	-5	0.0477936	0.0236507	-0.0125017	0.1080888
Most	Hateful non-audience outdegree	-4	-0.0264788	0.0396680	-0.1276088	0.0746511
Most	Hateful non-audience outdegree	-3	-0.0511938	0.0291511	-0.1255119	0.0231243
Most	Hateful non-audience outdegree	-2	0.0265405	0.0252561	-0.0378477	0.0909287
Most	Hateful non-audience outdegree	-1	0.0241340	0.0810860	-0.1825872	0.2308553
Most	Hateful non-audience outdegree	0	-0.1836069	0.1382050	-0.5359477	0.1687339
Most	Hateful non-audience outdegree	1	0.0548523	0.0668910	-0.1156802	0.2253848
Most	Hateful non-audience outdegree	2	0.0276342	0.0393993	-0.0728108	0.1280792
Most	Hateful non-audience outdegree	3	0.0786916	0.0448164	-0.0355638	0.1929469
Most	Hateful non-audience outdegree	4	0.0283842	0.0475593	-0.0928639	0.1496323
Most	Hateful non-audience outdegree	5	0.0236355	0.0756736	-0.1692874	0.2165584
Most	Hateful non-audience outdegree	6	0.0787735	0.0701176	-0.0999848	0.2575319
Most	Hateful non-audience outdegree	7	0.0453940	0.0756881	-0.1475659	0.2383539

Most	Hateful non-audience outdegree	8	0.0581986	0.0815635	-0.1497399	0.2661372
Most	Hateful non-audience outdegree	9	0.0987840	0.0820197	-0.1103178	0.3078858
Most	Hateful non-audience outdegree	10	0.0502558	0.0923778	-0.1852528	0.2857645
Most	Hateful non-audience outdegree	11	0.0523576	0.0940373	-0.1873818	0.2920970
Most	Hateful non-audience outdegree	12	0.0609096	0.0793150	-0.1412966	0.2631159
Most	Hateful non-audience outdegree	13	0.0330793	0.0725253	-0.1518173	0.2179759
Most	Hateful non-audience outdegree	14	0.0459140	0.1929996	-0.4461208	0.5379489
Most	Hateful non-audience outdegree	15	0.1090560	0.1922400	-0.3810422	0.5991542
Most	Hateful non-audience outdegree	16	0.0944968	0.2115831	-0.4449148	0.6339084
Most	Hateful non-audience outdegree	17	0.0372803	0.1905665	-0.4485515	0.5231121
Most	Hateful non-audience outdegree	18	0.0697638	0.2422716	-0.5478853	0.6874129
Most	Hateful non-audience outdegree	19	0.0958487	0.2318347	-0.4951925	0.6868900
Most	Hateful non-audience outdegree	20	0.0807864	0.2110821	-0.4573481	0.6189208
Most	Hateful non-audience outdegree	21	0.0490860	0.1960786	-0.4507983	0.5489704
Most	Hateful non-audience outdegree	22	0.0451282	0.2429886	-0.5743488	0.6646052
Most	Hateful non-audience outdegree	23	0.0696945	0.2210476	-0.4938461	0.6332351
Most	Hateful non-audience outdegree	24	0.1003893	0.2072604	-0.4280021	0.6287807
Most	Hateful non-audience outdegree	25	0.0157948	0.2038728	-0.5039600	0.5355497
Most	Hateful non-audience outdegree	26	0.0225282	0.1751158	-0.4239133	0.4689697
Most	Hateful non-audience outdegree	27	-0.0223582	0.2143457	-0.5688128	0.5240965
Most	Hateful non-audience outdegree	28	-0.0554266	0.2626039	-0.7249110	0.6140578
Most	Hateful non-audience outdegree	29	-0.1384958	0.2109348	-0.6762548	0.3992632
Most	Hateful non-audience outdegree	30	-0.1356830	0.1750237	-0.5818899	0.3105238

sample	outcome	event.time	estimate	std.error	conf.low	conf.high
Middle	Hateful non-audience outdegree	-30	0.0435851	0.0275389	-0.0272843	0.1144544
Middle	Hateful non-audience outdegree	-29	-0.0211027	0.0233046	-0.0810755	0.0388702
Middle	Hateful non-audience outdegree	-28	-0.0134207	0.0191566	-0.0627189	0.0358774
Middle	Hateful non-audience outdegree	-27	0.0114213	0.0159956	-0.0297421	0.0525847
Middle	Hateful non-audience outdegree	-26	0.0905946	0.0472753	-0.0310650	0.2122543
Middle	Hateful non-audience outdegree	-25	-0.0796310	0.0410396	-0.1852434	0.0259815
Middle	Hateful non-audience outdegree	-24	-0.0404534	0.0244914	-0.1034803	0.0225734
Middle	Hateful non-audience outdegree	-23	-0.0254999	0.0148976	-0.0638378	0.0128380
Middle	Hateful non-audience outdegree	-22	-0.0061178	0.0139292	-0.0419637	0.0297281
Middle	Hateful non-audience outdegree	-21	-0.0036552	0.0224686	-0.0614765	0.0541662
Middle	Hateful non-audience outdegree	-20	0.0298077	0.0326883	-0.0543133	0.1139287
Middle	Hateful non-audience outdegree	-19	0.0619638	0.0735165	-0.1272258	0.2511534
Middle	Hateful non-audience outdegree	-18	0.0078624	0.0287291	-0.0660699	0.0817946
Middle	Hateful non-audience outdegree	-17	0.0792870	0.0269444	0.0099475	0.1486265
Middle	Hateful non-audience outdegree	-16	0.0079871	0.0405906	-0.0964700	0.1124442
Middle	Hateful non-audience outdegree	-15	-0.0127780	0.0181855	-0.0595772	0.0340211

Middle	Hateful non-audience outdegree	-14	0.0370671	0.0335613	-0.0493004	0.1234347
Middle	Hateful non-audience outdegree	-13	-0.1494311	0.0653340	-0.3175634	0.0187012
Middle	Hateful non-audience outdegree	-12	0.1011186	0.0447088	-0.0139365	0.2161737
Middle	Hateful non-audience outdegree	-11	0.0298975	0.0238139	-0.0313860	0.0911810
Middle	Hateful non-audience outdegree	-10	0.0015962	0.0405225	-0.1026856	0.1058780
Middle	Hateful non-audience outdegree	-9	-0.0127230	0.0318384	-0.0946569	0.0692110
Middle	Hateful non-audience outdegree	-8	0.0174206	0.0292667	-0.0578952	0.0927363
Middle	Hateful non-audience outdegree	-7	-0.0805622	0.0243554	-0.1432392	-0.0178852
Middle	Hateful non-audience outdegree	-6	-0.1121884	0.0524022	-0.2470418	0.0226651
Middle	Hateful non-audience outdegree	-5	0.0226678	0.0294769	-0.0531889	0.0985245
Middle	Hateful non-audience outdegree	-4	-0.0276745	0.0216024	-0.0832668	0.0279179
Middle	Hateful non-audience outdegree	-3	-0.1187258	0.0431647	-0.2298070	-0.0076446
Middle	Hateful non-audience outdegree	-2	0.0650255	0.0272190	-0.0050208	0.1350718
Middle	Hateful non-audience outdegree	-1	-0.0090682	0.0373915	-0.1052925	0.0871562
Middle	Hateful non-audience outdegree	0	-0.1325504	0.0743650	-0.3239235	0.0588226
Middle	Hateful non-audience outdegree	1	0.1029768	0.0286600	0.0292222	0.1767314
Middle	Hateful non-audience outdegree	2	0.0969492	0.0496892	-0.0309224	0.2248209
Middle	Hateful non-audience outdegree	3	0.1283399	0.0572809	-0.0190684	0.2757482
Middle	Hateful non-audience outdegree	4	0.1718435	0.0890987	-0.0574458	0.4011328
Middle	Hateful non-audience outdegree	5	0.1290005	0.1083512	-0.1498338	0.4078348
Middle	Hateful non-audience outdegree	6	0.1328352	0.0985702	-0.1208283	0.3864988
Middle	Hateful non-audience outdegree	7	0.1871163	0.0995055	-0.0689542	0.4431868
Middle	Hateful non-audience outdegree	8	0.1398365	0.0879357	-0.0864599	0.3661328
Middle	Hateful non-audience outdegree	9	0.1538865	0.0960491	-0.0932892	0.4010621
Middle	Hateful non-audience outdegree	10	0.1716626	0.1012623	-0.0889288	0.4322541
Middle	Hateful non-audience outdegree	11	0.1989973	0.1340128	-0.1458752	0.5438698
Middle	Hateful non-audience outdegree	12	0.1895986	0.1327992	-0.1521507	0.5313478
Middle	Hateful non-audience outdegree	13	0.1211740	0.1141142	-0.1724909	0.4148388
Middle	Hateful non-audience outdegree	14	0.1223100	0.2001932	-0.3928732	0.6374932
Middle	Hateful non-audience outdegree	15	0.1505638	0.2279905	-0.4361537	0.7372814
Middle	Hateful non-audience outdegree	16	0.1588939	0.2244209	-0.4186375	0.7364253
Middle	Hateful non-audience outdegree	17	0.2032567	0.2933240	-0.5515920	0.9581054
Middle	Hateful non-audience outdegree	18	0.2183315	0.3184535	-0.6011861	1.0378492
Middle	Hateful non-audience outdegree	19	0.2092033	0.2880973	-0.5321947	0.9506013
Middle	Hateful non-audience outdegree	20	0.1513358	0.2422022	-0.4719545	0.7746261
Middle	Hateful non-audience outdegree	21	0.1533133	0.2875918	-0.5867838	0.8934104
Middle	Hateful non-audience outdegree	22	0.1497627	0.2634919	-0.5283151	0.8278406
Middle	Hateful non-audience outdegree	23	0.1530752	0.2417142	-0.4689592	0.7751095
Middle	Hateful non-audience outdegree	24	0.1880261	0.3003092	-0.5847984	0.9608506
Middle	Hateful non-audience outdegree	25	0.1909198	0.3043140	-0.5922107	0.9740504
Middle	Hateful non-audience outdegree	26	0.0763482	0.2286378	-0.5120349	0.6647313

Middle	Hateful non-audience outdegree	27	-0.1264863	0.2448145	-0.7564991	0.5035265
Middle	Hateful non-audience outdegree	28	0.0818153	0.2743892	-0.6243060	0.7879366
Middle	Hateful non-audience outdegree	29	0.0437565	0.2762287	-0.6670986	0.7546116
Middle	Hateful non-audience outdegree	30	-0.0890619	0.2667809	-0.7756036	0.5974797

sample	outcome	event.time	estimate	std.error	conf.low	conf.high
Least	Hateful non-audience outdegree	-30	-0.0280016	0.0340577	-0.1116411	0.0556379
Least	Hateful non-audience outdegree	-29	0.0662328	0.0261231	0.0020792	0.1303863
Least	Hateful non-audience outdegree	-28	-0.0409510	0.0291405	-0.1125148	0.0306128
Least	Hateful non-audience outdegree	-27	0.1399406	0.0548563	0.0052233	0.2746579
Least	Hateful non-audience outdegree	-26	0.1239860	0.0887331	-0.0939267	0.3418987
Least	Hateful non-audience outdegree	-25	-0.1476774	0.0770175	-0.3368185	0.0414638
Least	Hateful non-audience outdegree	-24	-0.0800149	0.1380689	-0.4190873	0.2590575
Least	Hateful non-audience outdegree	-23	-0.0396056	0.0238875	-0.0982690	0.0190578
Least	Hateful non-audience outdegree	-22	0.1485023	0.0600078	0.0011339	0.2958707
Least	Hateful non-audience outdegree	-21	-0.0171808	0.0380760	-0.1106887	0.0763270
Least	Hateful non-audience outdegree	-20	0.1204149	0.0352777	0.0337793	0.2070505
Least	Hateful non-audience outdegree	-19	0.2188032	0.0851386	0.0097180	0.4278884
Least	Hateful non-audience outdegree	-18	-0.0066194	0.0658740	-0.1683940	0.1551553
Least	Hateful non-audience outdegree	-17	0.0960429	0.0344964	0.0113259	0.1807598
Least	Hateful non-audience outdegree	-16	0.0297424	0.0504533	-0.0941618	0.1536466
Least	Hateful non-audience outdegree	-15	0.0712291	0.0489612	-0.0490109	0.1914691
Least	Hateful non-audience outdegree	-14	-0.0934226	0.0214082	-0.1459973	-0.0408478
Least	Hateful non-audience outdegree	-13	-0.2712302	0.1018682	-0.5214003	-0.0210602
Least	Hateful non-audience outdegree	-12	0.1331470	0.1171596	-0.1545761	0.4208701
Least	Hateful non-audience outdegree	-11	0.0297992	0.0373222	-0.0618574	0.1214558
Least	Hateful non-audience outdegree	-10	-0.0726277	0.0521344	-0.2006605	0.0554051
Least	Hateful non-audience outdegree	-9	-0.0197536	0.0748952	-0.2036829	0.1641757
Least	Hateful non-audience outdegree	-8	0.0014680	0.0505757	-0.1227369	0.1256728
Least	Hateful non-audience outdegree	-7	-0.1292740	0.0507247	-0.2538447	-0.0047033
Least	Hateful non-audience outdegree	-6	-0.0434021	0.0679483	-0.2102710	0.1234668
Least	Hateful non-audience outdegree	-5	-0.0555735	0.0562444	-0.1936995	0.0825526
Least	Hateful non-audience outdegree	-4	0.0146028	0.0424050	-0.0895364	0.1187419
Least	Hateful non-audience outdegree	-3	-0.1496072	0.0725690	-0.3278237	0.0286093
Least	Hateful non-audience outdegree	-2	0.0204713	0.0597111	-0.1261684	0.1671109
Least	Hateful non-audience outdegree	-1	0.0315535	0.0726052	-0.1467518	0.2098589
Least	Hateful non-audience outdegree	0	-0.3839855	0.1340982	-0.7133067	-0.0546642
Least	Hateful non-audience outdegree	1	0.0810002	0.0416994	-0.0214060	0.1834063
Least	Hateful non-audience outdegree	2	0.0767688	0.0538576	-0.0554957	0.2090333
Least	Hateful non-audience outdegree	3	0.1487303	0.1009666	-0.0992257	0.3966863
Least	Hateful non-audience outdegree	4	0.0815758	0.1061637	-0.1791433	0.3422949

Least	Hateful non-audience outdegree	5	0.1209467	0.1522734	-0.2530096	0.4949029
Least	Hateful non-audience outdegree	6	0.1202738	0.1230938	-0.1820225	0.4225700
Least	Hateful non-audience outdegree	7	0.1223047	0.1286643	-0.1936717	0.4382812
Least	Hateful non-audience outdegree	8	0.1368535	0.1285433	-0.1788258	0.4525328
Least	Hateful non-audience outdegree	9	0.1296817	0.2160961	-0.4010115	0.6603749
Least	Hateful non-audience outdegree	10	0.1063036	0.1969184	-0.3772928	0.5899000
Least	Hateful non-audience outdegree	11	0.1592157	0.1797123	-0.2821256	0.6005569
Least	Hateful non-audience outdegree	12	0.1619607	0.2303757	-0.4038008	0.7277222
Least	Hateful non-audience outdegree	13	0.0334877	0.1404977	-0.3115494	0.3785248
Least	Hateful non-audience outdegree	14	0.0450346	0.2917349	-0.6714140	0.7614832
Least	Hateful non-audience outdegree	15	0.1132660	0.2932854	-0.6069904	0.8335223
Least	Hateful non-audience outdegree	16	0.0991955	0.3516981	-0.7645121	0.9629031
Least	Hateful non-audience outdegree	17	0.0676339	0.3661225	-0.8314973	0.9667651
Least	Hateful non-audience outdegree	18	0.1387205	0.4038282	-0.8530092	1.1304503
Least	Hateful non-audience outdegree	19	0.0193937	0.2913172	-0.6960291	0.7348166
Least	Hateful non-audience outdegree	20	0.0474828	0.3198350	-0.7379746	0.8329402
Least	Hateful non-audience outdegree	21	0.0863590	0.3892331	-0.8695277	1.0422457
Least	Hateful non-audience outdegree	22	0.0042133	0.3715071	-0.9081415	0.9165681
Least	Hateful non-audience outdegree	23	0.0874593	0.3485293	-0.7684663	0.9433849
Least	Hateful non-audience outdegree	24	0.1375935	0.3817269	-0.7998594	1.0750463
Least	Hateful non-audience outdegree	25	0.1157231	0.4203633	-0.9166137	1.1480600
Least	Hateful non-audience outdegree	26	0.0747069	0.4047695	-0.9193346	1.0687483
Least	Hateful non-audience outdegree	27	-0.4091374	1.0963064	-3.1014694	2.2831946
Least	Hateful non-audience outdegree	28	-0.3221113	1.3486315	-3.6341086	2.9898860
Least	Hateful non-audience outdegree	29	-0.2601925	1.1699331	-3.1333387	2.6129537
Least	Hateful non-audience outdegree	30	-0.2247002	1.9367368	-4.9809788	4.5315784

Robustness: Dynamic differences-in-differences (Callaway and Sant'Anna 2021) for long time frame with sampled control groups

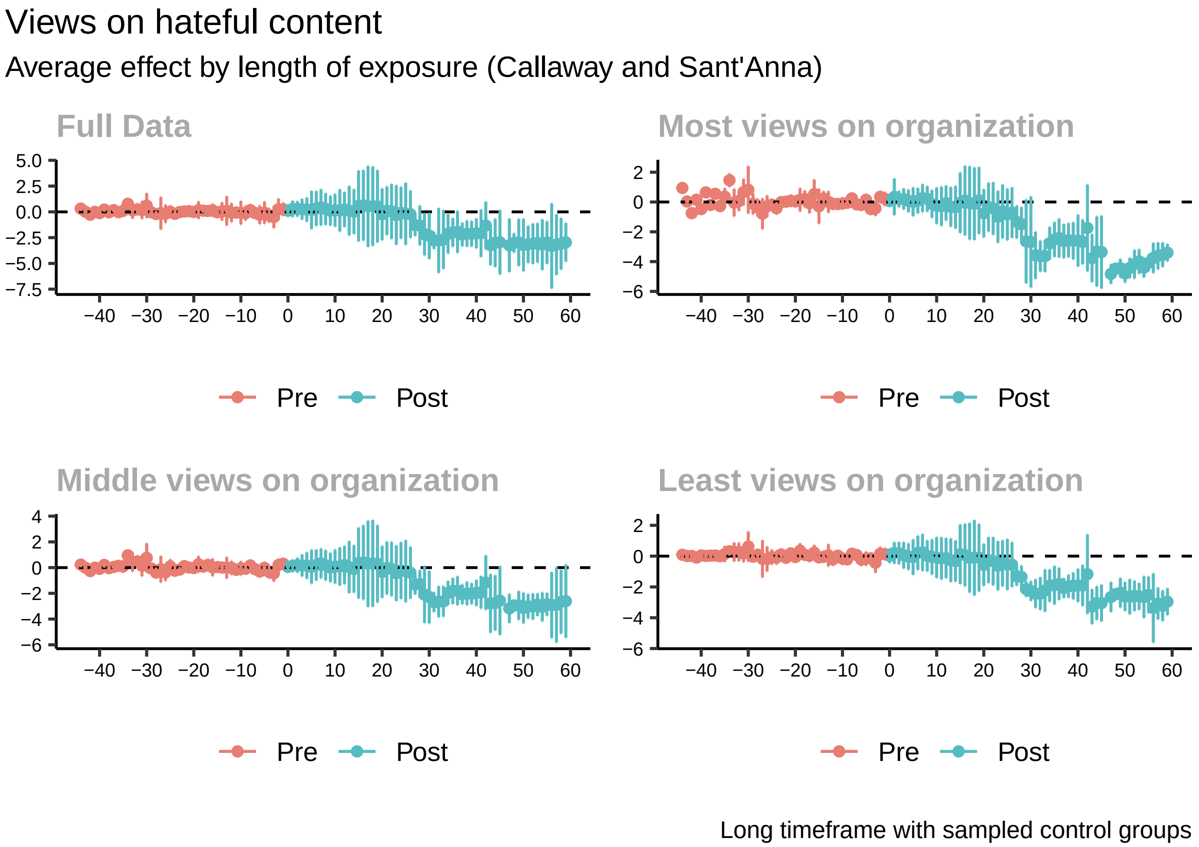

sample	outcome	event.time	estimate	std.error	conf.low	conf.high
Full	Views on hateful content	-44	0.3156433	0.0896840	0.0793014	0.5519852
Full	Views on hateful content	-43	0.0098167	0.0428141	-0.1030100	0.1226434
Full	Views on hateful content	-42	-0.2722284	0.0496683	-0.4031181	-0.1413388
Full	Views on hateful content	-41	-0.0015550	0.0580121	-0.1544325	0.1513226
Full	Views on hateful content	-40	-0.1222139	0.0808314	-0.3352265	0.0907987
Full	Views on hateful content	-39	0.2243700	0.0874472	-0.0060772	0.4548172
Full	Views on hateful content	-38	-0.0406787	0.0676672	-0.2190003	0.1376429
Full	Views on hateful content	-37	0.1660275	0.0362568	0.0704809	0.2615742
Full	Views on hateful content	-36	-0.0284254	0.0750141	-0.2261081	0.1692572
Full	Views on hateful content	-35	0.1033568	0.1980841	-0.4186487	0.6253624
Full	Views on hateful content	-34	0.7833448	0.1263958	0.4502575	1.1164322
Full	Views on hateful content	-33	0.1470407	0.3026320	-0.6504770	0.9445584
Full	Views on hateful content	-32	0.2460091	0.2026576	-0.2880489	0.7800671
Full	Views on hateful content	-31	0.1699635	0.3262618	-0.6898253	1.0297522
Full	Views on hateful content	-30	0.6064396	0.4521275	-0.5850396	1.7979189

Full	Views on hateful content	-29	-0.0453440	0.1387699	-0.4110405	0.3203526
Full	Views on hateful content	-28	-0.2037504	0.1115552	-0.4977287	0.0902280
Full	Views on hateful content	-27	-0.1256826	0.5964614	-1.6975210	1.4461557
Full	Views on hateful content	-26	-0.1843393	0.3316563	-1.0583440	0.6896655
Full	Views on hateful content	-25	0.0148706	0.2214793	-0.5687878	0.5985290
Full	Views on hateful content	-24	-0.1942747	0.1639694	-0.6263788	0.2378294
Full	Views on hateful content	-23	-0.0192825	0.0956843	-0.2714367	0.2328717
Full	Views on hateful content	-22	0.0337911	0.0665426	-0.1415669	0.2091491
Full	Views on hateful content	-21	0.0663085	0.0906191	-0.1724975	0.3051146
Full	Views on hateful content	-20	-0.0075830	0.0823797	-0.2246760	0.2095100
Full	Views on hateful content	-19	0.1701168	0.3190595	-0.6706919	1.0109256
Full	Views on hateful content	-18	0.1123242	0.0680309	-0.0669558	0.2916041
Full	Views on hateful content	-17	0.0830975	0.1507207	-0.3140927	0.4802877
Full	Views on hateful content	-16	0.1511782	0.2357415	-0.4700648	0.7724213
Full	Views on hateful content	-15	-0.0390828	0.1551686	-0.4479944	0.3698288
Full	Views on hateful content	-14	0.0352045	0.3341775	-0.8454442	0.9158531
Full	Views on hateful content	-13	0.1030491	0.5373676	-1.3130609	1.5191591
Full	Views on hateful content	-12	-0.0722805	0.3474257	-0.9878418	0.8432807
Full	Views on hateful content	-11	-0.0649783	0.1922002	-0.5714782	0.4415217
Full	Views on hateful content	-10	-0.0800036	0.4312828	-1.2165513	1.0565441
Full	Views on hateful content	-9	-0.1261442	0.2662209	-0.8277088	0.5754204
Full	Views on hateful content	-8	0.1672190	0.2113572	-0.3897650	0.7242029
Full	Views on hateful content	-7	-0.1053956	0.2079351	-0.6533612	0.4425700
Full	Views on hateful content	-6	-0.2792895	0.3428432	-1.1827747	0.6241957
Full	Views on hateful content	-5	-0.0799297	0.4026130	-1.1409248	0.9810654
Full	Views on hateful content	-4	-0.3428322	0.2609958	-1.0306274	0.3449629
Full	Views on hateful content	-3	-0.5043008	0.4041097	-1.5692400	0.5606384
Full	Views on hateful content	-2	0.2446016	0.3931174	-0.7913697	1.2805730
Full	Views on hateful content	-1	0.2441213	0.2417247	-0.3928891	0.8811318
Full	Views on hateful content	0	0.1096822	0.2046712	-0.4296823	0.6490467
Full	Views on hateful content	1	0.2851654	0.2981566	-0.5005585	1.0708893
Full	Views on hateful content	2	0.2841049	0.2819190	-0.4588284	1.0270382
Full	Views on hateful content	3	0.2516541	0.3782031	-0.7450142	1.2483224
Full	Views on hateful content	4	0.2175571	0.4411927	-0.9451060	1.3802202
Full	Views on hateful content	5	0.1848732	0.6960058	-1.6492919	2.0190382
Full	Views on hateful content	6	0.3277355	0.6350641	-1.3458314	2.0013024
Full	Views on hateful content	7	0.4307426	0.6645116	-1.3204265	2.1819118
Full	Views on hateful content	8	0.2752623	0.5813478	-1.2567476	1.8072722
Full	Views on hateful content	9	0.1148196	0.5435269	-1.3175219	1.5471611
Full	Views on hateful content	10	0.1563428	0.6008443	-1.4270457	1.7397313
Full	Views on hateful content	11	0.1373902	0.7742561	-1.9029857	2.1777661

Full	Views on hateful content	12	0.2343488	0.6406048	-1.4538193	1.9225170
Full	Views on hateful content	13	0.1161630	0.9055389	-2.2701789	2.5025049
Full	Views on hateful content	14	0.0251924	0.8174872	-2.1291091	2.1794940
Full	Views on hateful content	15	0.5775605	1.3003897	-2.8493208	4.0044418
Full	Views on hateful content	16	0.6356107	1.2980115	-2.7850034	4.0562248
Full	Views on hateful content	17	0.5306960	1.4890748	-3.3934220	4.4548140
Full	Views on hateful content	18	0.5451105	1.4578706	-3.2967759	4.3869969
Full	Views on hateful content	19	0.5359974	1.3270228	-2.9610694	4.0330641
Full	Views on hateful content	20	-0.2360786	0.9497051	-2.7388105	2.2666533
Full	Views on hateful content	21	0.1061530	0.8921260	-2.2448421	2.4571481
Full	Views on hateful content	22	0.0372203	1.0093437	-2.6226754	2.6971160
Full	Views on hateful content	23	-0.3095275	1.0896934	-3.1811667	2.5621117
Full	Views on hateful content	24	-0.0845696	0.9518067	-2.5928397	2.4237006
Full	Views on hateful content	25	-0.1852299	1.1363262	-3.1797591	2.8092992
Full	Views on hateful content	26	-0.2305488	0.8715341	-2.5272787	2.0661810
Full	Views on hateful content	27	-1.2906243	0.3984067	-2.3405344	-0.2407141
Full	Views on hateful content	28	-1.3227860	0.7256583	-3.2350933	0.5895213
Full	Views on hateful content	29	-2.1594439	0.7787158	-4.2115722	-0.1073156
Full	Views on hateful content	30	-2.3154557	0.8498791	-4.5551186	-0.0757928
Full	Views on hateful content	31	-2.7748390	0.3095152	-3.5904959	-1.9591821
Full	Views on hateful content	32	-2.7681846	1.1892466	-5.9021735	0.3658043
Full	Views on hateful content	33	-2.6818647	1.0815206	-5.5319661	0.1682367
Full	Views on hateful content	34	-2.1289127	0.7187588	-4.0230380	-0.2347875
Full	Views on hateful content	35	-1.9959694	0.5215185	-3.3703127	-0.6216260
Full	Views on hateful content	36	-1.9356938	0.7694879	-3.9635040	0.0921165
Full	Views on hateful content	37	-2.1968792	0.4302405	-3.3306802	-1.0630782
Full	Views on hateful content	38	-2.1109639	0.4771362	-3.3683477	-0.8535801
Full	Views on hateful content	39	-2.1223115	0.4677403	-3.3549347	-0.8896882
Full	Views on hateful content	40	-2.0834797	0.5450001	-3.5197035	-0.6472559
Full	Views on hateful content	41	-2.0803262	0.8667380	-4.3644170	0.2037646
Full	Views on hateful content	42	-1.3187876	0.8705918	-3.6130341	0.9754589
Full	Views on hateful content	43	-3.2284992	0.7351854	-5.1659130	-1.2910855
Full	Views on hateful content	44	-3.0072223	0.8812027	-5.3294317	-0.6850129
Full	Views on hateful content	45	-2.9464396	1.1783395	-6.0516853	0.1588060
Full	Views on hateful content	47	-3.2436477	0.9807965	-5.8283137	-0.6589816
Full	Views on hateful content	48	-3.0117672	0.3514167	-3.9378460	-2.0856885
Full	Views on hateful content	49	-2.9666189	0.8660325	-5.2488505	-0.6843873
Full	Views on hateful content	50	-3.2132847	0.9635389	-5.7524721	-0.6740972
Full	Views on hateful content	51	-3.1533865	0.6790064	-4.9427534	-1.3640196
Full	Views on hateful content	52	-3.0945418	0.7415655	-5.0487691	-1.1403146
Full	Views on hateful content	53	-3.0069565	0.7241553	-4.9153030	-1.0986099

Full	Views on hateful content	54	-3.1801691	0.9276341	-5.6247378	-0.7356004
Full	Views on hateful content	55	-2.9751485	0.7789214	-5.0278186	-0.9224784
Full	Views on hateful content	56	-3.3046670	1.5617246	-7.4202369	0.8109029
Full	Views on hateful content	57	-3.1749514	1.1091288	-6.0978080	-0.2520948
Full	Views on hateful content	58	-3.0869328	0.9446566	-5.5763603	-0.5975053
Full	Views on hateful content	59	-2.9530223	0.7089435	-4.8212815	-1.0847630

sample	outcome	event.time	estimate	std.error	conf.low	conf.high
Most	Views on hateful content	-44	0.9393102	0.1015181	0.6733859	1.2052346
Most	Views on hateful content	-43	0.0350929	0.0495727	-0.0947617	0.1649475
Most	Views on hateful content	-42	-0.7489948	0.0821332	-0.9641409	-0.5338486
Most	Views on hateful content	-41	0.1518887	0.0794382	-0.0561978	0.3599752
Most	Views on hateful content	-40	-0.4772743	0.0684625	-0.6566103	-0.2979382
Most	Views on hateful content	-39	0.6432520	0.1097963	0.3556431	0.9308608
Most	Views on hateful content	-38	-0.1956736	0.1016504	-0.4619445	0.0705974
Most	Views on hateful content	-37	0.5496962	0.0478340	0.4243960	0.6749964
Most	Views on hateful content	-36	-0.2744977	0.0857430	-0.4990996	-0.0498957
Most	Views on hateful content	-35	0.3581583	0.2145387	-0.2038211	0.9201377
Most	Views on hateful content	-34	1.4588292	0.1716785	1.0091213	1.9085372
Most	Views on hateful content	-33	-0.0432946	0.3522738	-0.9660679	0.8794786
Most	Views on hateful content	-32	0.0619567	0.2481068	-0.5879534	0.7118668
Most	Views on hateful content	-31	0.6686911	0.3361047	-0.2117276	1.5491099
Most	Views on hateful content	-30	0.8195811	0.6027858	-0.7594027	2.3985650
Most	Views on hateful content	-29	-0.1182749	0.2679785	-0.8202386	0.5836889
Most	Views on hateful content	-28	-0.3810084	0.2261749	-0.9734685	0.2114517
Most	Views on hateful content	-27	-0.7864696	0.3930074	-1.8159438	0.2430046
Most	Views on hateful content	-26	-0.2828792	0.2728074	-0.9974920	0.4317337
Most	Views on hateful content	-25	-0.1809940	0.1267050	-0.5128950	0.1509069
Most	Views on hateful content	-24	-0.4163606	0.1578480	-0.8298400	-0.0028811
Most	Views on hateful content	-23	-0.0091329	0.1005405	-0.2724964	0.2542307
Most	Views on hateful content	-22	0.0203649	0.0886512	-0.2118549	0.2525847
Most	Views on hateful content	-21	0.0843136	0.1534627	-0.3176786	0.4863059
Most	Views on hateful content	-20	0.0387463	0.1288721	-0.2988313	0.3763239
Most	Views on hateful content	-19	0.1475477	0.2969965	-0.6304281	0.9255235
Most	Views on hateful content	-18	0.2004929	0.2149820	-0.3626476	0.7636333
Most	Views on hateful content	-17	-0.0851015	0.2526059	-0.7467970	0.5765940
Most	Views on hateful content	-16	0.5315703	0.3699311	-0.4374559	1.5005965
Most	Views on hateful content	-15	-0.2869391	0.4451353	-1.4529611	0.8790829
Most	Views on hateful content	-14	0.2265054	0.1872096	-0.2638859	0.7168967
Most	Views on hateful content	-13	0.0025300	0.2740358	-0.7153007	0.7203607
Most	Views on hateful content	-12	-0.1255525	0.1456530	-0.5070874	0.2559824

Most	Views on hateful content	-11	-0.1344193	0.0858176	-0.3592166	0.0903781
Most	Views on hateful content	-10	-0.1000943	0.1502297	-0.4936177	0.2934292
Most	Views on hateful content	-9	-0.0533085	0.1093674	-0.3397939	0.2331770
Most	Views on hateful content	-8	0.2482779	0.1105762	-0.0413739	0.5379298
Most	Views on hateful content	-7	-0.1584468	0.1181925	-0.4680494	0.1511558
Most	Views on hateful content	-6	-0.1984426	0.1268792	-0.5307998	0.1339145
Most	Views on hateful content	-5	0.1467778	0.1424838	-0.2264554	0.5200110
Most	Views on hateful content	-4	-0.4770792	0.1326300	-0.8245005	-0.1296580
Most	Views on hateful content	-3	-0.4270639	0.1954497	-0.9390400	0.0849122
Most	Views on hateful content	-2	0.3486728	0.1360721	-0.0077651	0.7051106
Most	Views on hateful content	-1	0.2077832	0.1752041	-0.2511600	0.6667265
Most	Views on hateful content	0	0.0664171	0.1479930	-0.3212472	0.4540815
Most	Views on hateful content	1	0.3457311	0.4680217	-0.8802411	1.5717033
Most	Views on hateful content	2	0.1977803	0.2064839	-0.3430996	0.7386601
Most	Views on hateful content	3	0.1965730	0.2668551	-0.5024481	0.8955940
Most	Views on hateful content	4	0.0733454	0.2868514	-0.6780553	0.8247461
Most	Views on hateful content	5	0.0137337	0.3670898	-0.9478499	0.9753173
Most	Views on hateful content	6	0.0893636	0.3226401	-0.7557848	0.9345120
Most	Views on hateful content	7	0.2562238	0.3565090	-0.6776436	1.1900913
Most	Views on hateful content	8	0.2084901	0.3258356	-0.6450290	1.0620091
Most	Views on hateful content	9	-0.1306538	0.3515163	-1.0514429	0.7901353
Most	Views on hateful content	10	-0.2460658	0.4620107	-1.4562925	0.9641609
Most	Views on hateful content	11	-0.2854031	0.4991743	-1.5929789	1.0221726
Most	Views on hateful content	12	-0.0733669	0.4458060	-1.2411458	1.0944119
Most	Views on hateful content	13	-0.3284799	0.5046191	-1.6503183	0.9933586
Most	Views on hateful content	14	-0.3516812	0.5419247	-1.7712409	1.0678785
Most	Views on hateful content	15	-0.0497281	0.7726844	-2.0737577	1.9743015
Most	Views on hateful content	16	0.0958211	0.8876079	-2.2292479	2.4208901
Most	Views on hateful content	17	-0.0638335	0.9372172	-2.5188529	2.3911858
Most	Views on hateful content	18	-0.1167245	0.9275595	-2.5464458	2.3129969
Most	Views on hateful content	19	0.1045884	0.8549128	-2.1348365	2.3440133
Most	Views on hateful content	20	-0.7572477	0.6140260	-2.3656750	0.8511797
Most	Views on hateful content	21	-0.3463150	0.6229290	-1.9780635	1.2854334
Most	Views on hateful content	22	-0.4367937	0.6704712	-2.1930779	1.3194905
Most	Views on hateful content	23	-0.9908333	0.6628945	-2.7272704	0.7456039
Most	Views on hateful content	24	-0.6301505	0.6869761	-2.4296691	1.1693680
Most	Views on hateful content	25	-0.8373686	0.6581797	-2.5614556	0.8867184
Most	Views on hateful content	26	-0.7164043	0.6421171	-2.3984157	0.9656071
Most	Views on hateful content	27	-1.3421539	0.4166955	-2.4336783	-0.2506295
Most	Views on hateful content	28	-1.4876971	0.4400454	-2.6403861	-0.3350082
Most	Views on hateful content	29	-2.6434995	1.0700751	-5.4465372	0.1595382

Most	Views on hateful content	30	-2.6822537	1.1656064	-5.7355336	0.3710262
Most	Views on hateful content	31	-3.5773546	0.6016566	-5.1533807	-2.0013285
Most	Views on hateful content	32	-3.6347937	0.3943610	-4.6678136	-2.6017738
Most	Views on hateful content	33	-3.6243867	0.4093361	-4.6966334	-2.5521400
Most	Views on hateful content	34	-2.7705891	0.4181059	-3.8658080	-1.6753701
Most	Views on hateful content	35	-2.5164099	0.4477630	-3.6893150	-1.3435049
Most	Views on hateful content	36	-2.4184639	0.4939829	-3.7124409	-1.1244869
Most	Views on hateful content	37	-2.6296106	0.4336753	-3.7656133	-1.4936080
Most	Views on hateful content	38	-2.5666191	0.4359625	-3.7086131	-1.4246250
Most	Views on hateful content	39	-2.6039770	0.4743086	-3.8464178	-1.3615363
Most	Views on hateful content	40	-2.5884324	0.6610843	-4.3201278	-0.8567369
Most	Views on hateful content	41	-2.6735809	0.5711572	-4.1697144	-1.1774473
Most	Views on hateful content	42	-1.7417606	1.1076846	-4.6433156	1.1597943
Most	Views on hateful content	43	-3.7676931	0.6125404	-5.3722290	-2.1631573
Most	Views on hateful content	44	-3.3195563	0.8967497	-5.6685722	-0.9705405
Most	Views on hateful content	45	-3.3580426	0.9304783	-5.7954096	-0.9206756
Most	Views on hateful content	47	-4.8277071	0.2507334	-5.4844977	-4.1709165
Most	Views on hateful content	48	-4.4753683	0.0851427	-4.6983977	-4.2523388
Most	Views on hateful content	49	-4.4418444	0.2347772	-5.0568381	-3.8268507
Most	Views on hateful content	50	-4.7941180	0.2342438	-5.4077144	-4.1805216
Most	Views on hateful content	51	-4.4262938	0.2587038	-5.1039626	-3.7486250
Most	Views on hateful content	52	-4.1736391	0.3654987	-5.1310546	-3.2162235
Most	Views on hateful content	53	-3.9917520	0.3108299	-4.8059640	-3.1775401
Most	Views on hateful content	54	-4.4079971	0.2498288	-5.0624180	-3.7535762
Most	Views on hateful content	55	-4.0830322	0.2087497	-4.6298473	-3.5362172
Most	Views on hateful content	56	-3.7557110	0.3868926	-4.7691676	-2.7422544
Most	Views on hateful content	57	-3.6160394	0.3422347	-4.5125155	-2.7195632
Most	Views on hateful content	58	-3.5379205	0.3108274	-4.3521259	-2.7237151
Most	Views on hateful content	59	-3.4031829	0.2193710	-3.9778203	-2.8285454

sample	outcome	event.time	estimate	std.error	conf.low	conf.high
Middle	Views on hateful content	-44	0.2317459	0.0786647	0.0241140	0.4393777
Middle	Views on hateful content	-43	-0.0057926	0.0515453	-0.1418439	0.1302587
Middle	Views on hateful content	-42	-0.2662493	0.0678558	-0.4453514	-0.0871471
Middle	Views on hateful content	-41	-0.0089105	0.0602659	-0.1679794	0.1501585
Middle	Views on hateful content	-40	-0.0972873	0.0706165	-0.2836763	0.0891016
Middle	Views on hateful content	-39	0.2124450	0.0856512	-0.0136274	0.4385174
Middle	Views on hateful content	-38	-0.0409854	0.0710608	-0.2285471	0.1465764
Middle	Views on hateful content	-37	0.0492996	0.0458950	-0.0718380	0.1704372
Middle	Views on hateful content	-36	0.1392956	0.0800060	-0.0718766	0.3504677
Middle	Views on hateful content	-35	0.0927139	0.1824220	-0.3887802	0.5742081

Middle	Views on hateful content	-34	0.9530565	0.1125180	0.6560705	1.2500425
Middle	Views on hateful content	-33	0.4383089	0.2813412	-0.3042780	1.1808957
Middle	Views on hateful content	-32	0.4794356	0.1642520	0.0459002	0.9129709
Middle	Views on hateful content	-31	0.1417520	0.3061643	-0.6663541	0.9498581
Middle	Views on hateful content	-30	0.7604249	0.4260326	-0.3640681	1.8849179
Middle	Views on hateful content	-29	-0.0737939	0.1302668	-0.4176271	0.2700392
Middle	Views on hateful content	-28	-0.3870626	0.1533358	-0.7917853	0.0176602
Middle	Views on hateful content	-27	-0.1262934	0.3852750	-1.1432085	0.8906216
Middle	Views on hateful content	-26	-0.3677564	0.2503694	-1.0285946	0.2930817
Middle	Views on hateful content	-25	0.0969332	0.2038551	-0.4411327	0.6349992
Middle	Views on hateful content	-24	-0.2365654	0.1471621	-0.6249929	0.1518620
Middle	Views on hateful content	-23	-0.1556950	0.0817054	-0.3713525	0.0599625
Middle	Views on hateful content	-22	0.1150736	0.0739687	-0.0801633	0.3103105
Middle	Views on hateful content	-21	0.0015890	0.0893021	-0.2341198	0.2372977
Middle	Views on hateful content	-20	-0.0299485	0.0904678	-0.2687339	0.2088369
Middle	Views on hateful content	-19	0.2616419	0.2387696	-0.3685791	0.8918629
Middle	Views on hateful content	-18	0.0771198	0.0731447	-0.1159423	0.2701818
Middle	Views on hateful content	-17	0.2084923	0.1658406	-0.2292361	0.6462208
Middle	Views on hateful content	-16	0.0170330	0.2561157	-0.6589722	0.6930383
Middle	Views on hateful content	-15	0.0447389	0.1299662	-0.2983008	0.3877785
Middle	Views on hateful content	-14	0.0070432	0.1440683	-0.3732181	0.3873046
Middle	Views on hateful content	-13	-0.0074468	0.3131476	-0.8339851	0.8190916
Middle	Views on hateful content	-12	-0.0153485	0.2017201	-0.5477790	0.5170820
Middle	Views on hateful content	-11	-0.1717293	0.1072122	-0.4547107	0.1112522
Middle	Views on hateful content	-10	-0.0511079	0.2062623	-0.5955276	0.4933117
Middle	Views on hateful content	-9	-0.0885459	0.1218063	-0.4100480	0.2329562
Middle	Views on hateful content	-8	0.1687556	0.1528227	-0.2346128	0.5721240
Middle	Views on hateful content	-7	-0.1202255	0.1245299	-0.4489163	0.2084653
Middle	Views on hateful content	-6	-0.3021597	0.1610870	-0.7273411	0.1230218
Middle	Views on hateful content	-5	-0.0243968	0.1666028	-0.4641370	0.4153434
Middle	Views on hateful content	-4	-0.3399322	0.1894715	-0.8400333	0.1601689
Middle	Views on hateful content	-3	-0.4457945	0.2358741	-1.0683730	0.1767840
Middle	Views on hateful content	-2	0.2004403	0.1846164	-0.2868458	0.6877265
Middle	Views on hateful content	-1	0.3023985	0.1429190	-0.0748295	0.6796265
Middle	Views on hateful content	0	0.0589648	0.1495213	-0.3356895	0.4536192
Middle	Views on hateful content	1	0.1428803	0.1813170	-0.3356972	0.6214579
Middle	Views on hateful content	2	0.1882383	0.2166169	-0.3835117	0.7599883
Middle	Views on hateful content	3	0.1662815	0.3218482	-0.6832215	1.0157845
Middle	Views on hateful content	4	0.1626392	0.3887027	-0.8633231	1.1886015
Middle	Views on hateful content	5	0.0675574	0.4955514	-1.2404270	1.3755418
Middle	Views on hateful content	6	0.1997705	0.4502581	-0.9886645	1.3882056

Middle	Views on hateful content	7	0.3294263	0.4370655	-0.8241874	1.4830400
Middle	Views on hateful content	8	0.1867906	0.4455619	-0.9892489	1.3628301
Middle	Views on hateful content	9	0.0089206	0.4289836	-1.1233613	1.1412026
Middle	Views on hateful content	10	0.0872879	0.4964491	-1.2230660	1.3976418
Middle	Views on hateful content	11	0.0865160	0.5592585	-1.3896203	1.5626523
Middle	Views on hateful content	12	0.2007273	0.5628541	-1.2848995	1.6863542
Middle	Views on hateful content	13	0.0397407	0.7635188	-1.9755310	2.0550123
Middle	Views on hateful content	14	-0.1015847	0.6976468	-1.9429904	1.7398211
Middle	Views on hateful content	15	0.3596814	1.0408856	-2.3876868	3.1070495
Middle	Views on hateful content	16	0.4034497	1.0921214	-2.4791530	3.2860523
Middle	Views on hateful content	17	0.2958199	1.2676436	-3.0500654	3.6417052
Middle	Views on hateful content	18	0.3285400	1.2738632	-3.0337616	3.6908416
Middle	Views on hateful content	19	0.2910259	1.1377641	-2.7120486	3.2941004
Middle	Views on hateful content	20	-0.3314131	0.7639706	-2.3478773	1.6850512
Middle	Views on hateful content	21	-0.0319482	0.7749530	-2.0774000	2.0135035
Middle	Views on hateful content	22	-0.1085816	0.7982679	-2.2155718	1.9984087
Middle	Views on hateful content	23	-0.4346352	0.8152592	-2.5864734	1.7172030
Middle	Views on hateful content	24	-0.2319190	0.8336837	-2.4323878	1.9685498
Middle	Views on hateful content	25	-0.2737800	0.9125964	-2.6825350	2.1349751
Middle	Views on hateful content	26	-0.3984898	0.7630182	-2.4124401	1.6154606
Middle	Views on hateful content	27	-1.3081898	0.2917844	-2.0783408	-0.5380387
Middle	Views on hateful content	28	-1.2805779	0.4074316	-2.3559744	-0.2051815
Middle	Views on hateful content	29	-2.0992370	0.8323879	-4.2962854	0.0978114
Middle	Views on hateful content	30	-2.2931306	0.7724301	-4.3319232	-0.2543380
Middle	Views on hateful content	31	-2.6864325	0.2897918	-3.4513242	-1.9215409
Middle	Views on hateful content	32	-2.6482354	0.4552459	-3.8498354	-1.4466355
Middle	Views on hateful content	33	-2.6244476	0.4482132	-3.8074852	-1.4414100
Middle	Views on hateful content	34	-1.9723274	0.3499134	-2.8959073	-1.0487476
Middle	Views on hateful content	35	-1.8199780	0.3830339	-2.8309779	-0.8089780
Middle	Views on hateful content	36	-1.7973997	0.4229762	-2.9138253	-0.6809740
Middle	Views on hateful content	37	-2.0728129	0.2894146	-2.8367090	-1.3089168
Middle	Views on hateful content	38	-2.0057922	0.3429368	-2.9109576	-1.1006269
Middle	Views on hateful content	39	-2.0276596	0.3068425	-2.8375560	-1.2177632
Middle	Views on hateful content	40	-1.9712223	0.3822671	-2.9801982	-0.9622464
Middle	Views on hateful content	41	-1.9193559	0.4794289	-3.1847858	-0.6539260
Middle	Views on hateful content	42	-1.1636609	0.7988959	-3.2723089	0.9449872
Middle	Views on hateful content	43	-2.7846582	0.8615671	-5.0587239	-0.5105926
Middle	Views on hateful content	44	-2.7433546	0.8106882	-4.8831277	-0.6035815
Middle	Views on hateful content	45	-2.5643090	1.0117001	-5.2346436	0.1060257
Middle	Views on hateful content	47	-3.1723006	0.4266919	-4.2985338	-2.0460674
Middle	Views on hateful content	48	-2.9428380	0.1511076	-3.3416792	-2.5439968

Middle	Views on hateful content	49	-2.9402324	0.4208919	-4.0511567	-1.8293081
Middle	Views on hateful content	50	-3.1450143	0.4502365	-4.3333922	-1.9566365
Middle	Views on hateful content	51	-2.9989901	0.3602775	-3.9499254	-2.0480548
Middle	Views on hateful content	52	-3.0261504	0.3838122	-4.0392046	-2.0130962
Middle	Views on hateful content	53	-2.8809918	0.3309439	-3.7545026	-2.0074810
Middle	Views on hateful content	54	-3.0509953	0.4237859	-4.1695581	-1.9324325
Middle	Views on hateful content	55	-2.8476772	0.3364297	-3.7356675	-1.9596870
Middle	Views on hateful content	56	-2.9187644	0.9685861	-5.4753016	-0.3622272
Middle	Views on hateful content	57	-2.9029090	1.1073810	-5.8257886	0.0199706
Middle	Views on hateful content	58	-2.6559013	0.9366146	-5.1280512	-0.1837514
Middle	Views on hateful content	59	-2.6071689	1.0748480	-5.4441793	0.2298414

sample	outcome	event.time	estimate	std.error	conf.low	conf.high
Least	Views on hateful content	-44	0.0889137	0.0982886	-0.1711300	0.3489574
Least	Views on hateful content	-43	0.0083944	0.0683671	-0.1724856	0.1892744
Least	Views on hateful content	-42	0.0103021	0.0695645	-0.1737458	0.1943501
Least	Views on hateful content	-41	-0.0918864	0.0834235	-0.3126015	0.1288286
Least	Views on hateful content	-40	0.0455610	0.0868850	-0.1843121	0.2754340
Least	Views on hateful content	-39	0.0147353	0.0947776	-0.2360192	0.2654899
Least	Views on hateful content	-38	0.0305449	0.0597769	-0.1276077	0.1886975
Least	Views on hateful content	-37	0.0420841	0.0473364	-0.0831547	0.1673228
Least	Views on hateful content	-36	-0.0057757	0.0790869	-0.2150173	0.2034659
Least	Views on hateful content	-35	0.1355632	0.1945627	-0.3791944	0.6503208
Least	Views on hateful content	-34	0.3130993	0.1061994	0.0321259	0.5940727
Least	Views on hateful content	-33	0.2655132	0.2299671	-0.3429144	0.8739409
Least	Views on hateful content	-32	0.2732977	0.2247190	-0.3212450	0.8678404
Least	Views on hateful content	-31	0.1691174	0.2093695	-0.3848149	0.7230497
Least	Views on hateful content	-30	0.6212510	0.3630068	-0.3391621	1.5816640
Least	Views on hateful content	-29	-0.0349158	0.0997780	-0.2989002	0.2290686
Least	Views on hateful content	-28	0.0946242	0.0764753	-0.1077078	0.2969561
Least	Views on hateful content	-27	-0.1792049	0.4545103	-1.3817104	1.0233005
Least	Views on hateful content	-26	-0.1851533	0.3025307	-0.9855636	0.6152570
Least	Views on hateful content	-25	-0.0643641	0.1829204	-0.5483195	0.4195913
Least	Views on hateful content	-24	-0.0946536	0.1657010	-0.5330514	0.3437442
Least	Views on hateful content	-23	0.1069538	0.1304316	-0.2381313	0.4520388
Least	Views on hateful content	-22	-0.1047055	0.1039015	-0.3795993	0.1701883
Least	Views on hateful content	-21	0.1820615	0.0994899	-0.0811606	0.4452836
Least	Views on hateful content	-20	-0.0583404	0.0840548	-0.2807257	0.1640449
Least	Views on hateful content	-19	0.3520287	0.1793789	-0.1225569	0.8266142
Least	Views on hateful content	-18	0.0816233	0.1161882	-0.2257777	0.3890242
Least	Views on hateful content	-17	0.0245445	0.1486435	-0.3687240	0.4178129

Least	Views on hateful content	-16	0.2289855	0.1863598	-0.2640696	0.7220406
Least	Views on hateful content	-15	-0.0789690	0.0892418	-0.3150773	0.1571394
Least	Views on hateful content	-14	-0.0427898	0.1332080	-0.3952204	0.3096408
Least	Views on hateful content	-13	0.0512470	0.2703222	-0.6639488	0.7664428
Least	Views on hateful content	-12	-0.2057818	0.1548039	-0.6153489	0.2037853
Least	Views on hateful content	-11	0.0298321	0.0817866	-0.1865520	0.2462163
Least	Views on hateful content	-10	-0.1590357	0.1569375	-0.5742477	0.2561763
Least	Views on hateful content	-9	-0.2096353	0.1144540	-0.5124481	0.0931774
Least	Views on hateful content	-8	0.1553296	0.1105263	-0.1370917	0.4477510
Least	Views on hateful content	-7	0.0730612	0.1051557	-0.2051510	0.3512733
Least	Views on hateful content	-6	-0.2218845	0.1537672	-0.6287088	0.1849399
Least	Views on hateful content	-5	-0.1615371	0.1728109	-0.6187458	0.2956716
Least	Views on hateful content	-4	-0.1426440	0.1314477	-0.4904174	0.2051295
Least	Views on hateful content	-3	-0.4279461	0.2441699	-1.0739504	0.2180582
Least	Views on hateful content	-2	0.1525317	0.1678388	-0.2915222	0.5965856
Least	Views on hateful content	-1	0.1285934	0.1441512	-0.2527899	0.5099766
Least	Views on hateful content	0	0.0262655	0.1726825	-0.4306035	0.4831345
Least	Views on hateful content	1	0.2089800	0.2624194	-0.4853072	0.9032672
Least	Views on hateful content	2	0.1939742	0.2698228	-0.5199005	0.9078489
Least	Views on hateful content	3	0.0513960	0.3222289	-0.8011303	0.9039223
Least	Views on hateful content	4	-0.0263594	0.3223937	-0.8793218	0.8266029
Least	Views on hateful content	5	-0.0669016	0.4316295	-1.2088708	1.0750677
Least	Views on hateful content	6	0.2127194	0.4178293	-0.8927383	1.3181771
Least	Views on hateful content	7	0.2407987	0.4526453	-0.9567723	1.4383698
Least	Views on hateful content	8	0.0164170	0.3781824	-0.9841464	1.0169805
Least	Views on hateful content	9	-0.0526242	0.4311821	-1.1934097	1.0881614
Least	Views on hateful content	10	-0.0938476	0.5011016	-1.4196203	1.2319250
Least	Views on hateful content	11	-0.1457281	0.5170873	-1.5137943	1.2223382
Least	Views on hateful content	12	-0.1306611	0.4911208	-1.4300276	1.1687053
Least	Views on hateful content	13	-0.2651119	0.5322619	-1.6733260	1.1431022
Least	Views on hateful content	14	-0.3211173	0.5012923	-1.6473945	1.0051598
Least	Views on hateful content	15	0.1239036	0.7281551	-1.8025882	2.0503954
Least	Views on hateful content	16	0.0709878	0.7644338	-1.9514873	2.0934629
Least	Views on hateful content	17	-0.1063238	0.8531420	-2.3634956	2.1508479
Least	Views on hateful content	18	-0.1028395	0.9226402	-2.5438837	2.3382047
Least	Views on hateful content	19	-0.1014069	0.8281769	-2.2925282	2.0897144
Least	Views on hateful content	20	-0.5613616	0.5140619	-1.9214236	0.7987004
Least	Views on hateful content	21	-0.2573461	0.5606792	-1.7407443	1.2260520
Least	Views on hateful content	22	-0.3304276	0.5894752	-1.8900118	1.2291565
Least	Views on hateful content	23	-0.6218938	0.6011894	-2.2124704	0.9686828
Least	Views on hateful content	24	-0.4656685	0.5621187	-1.9528752	1.0215383

Least	Views on hateful content	25	-0.5455824	0.6226677	-2.1929844	1.1018196
Least	Views on hateful content	26	-0.5488905	0.5483844	-1.9997600	0.9019789
Least	Views on hateful content	27	-1.3045764	0.2285805	-1.9093355	-0.6998172
Least	Views on hateful content	28	-1.3460545	0.2746644	-2.0727386	-0.6193705
Least	Views on hateful content	29	-2.1419102	0.1878192	-2.6388264	-1.6449940
Least	Views on hateful content	30	-2.2687899	0.2427248	-2.9109710	-1.6266088
Least	Views on hateful content	31	-2.4379800	0.3520898	-3.3695098	-1.5064502
Least	Views on hateful content	32	-2.4330890	0.3988325	-3.4882865	-1.3778914
Least	Views on hateful content	33	-2.2318913	0.5174738	-3.6009803	-0.8628023
Least	Views on hateful content	34	-1.9165989	0.3947231	-2.9609244	-0.8722734
Least	Views on hateful content	35	-1.8853222	0.4706696	-3.1305806	-0.6400639
Least	Views on hateful content	36	-1.8016969	0.3936133	-2.8430859	-0.7603078
Least	Views on hateful content	37	-2.0769044	0.2404335	-2.7130233	-1.4407854
Least	Views on hateful content	38	-1.9526363	0.2808579	-2.6957066	-1.2095661
Least	Views on hateful content	39	-1.9341857	0.3266302	-2.7983565	-1.0700150
Least	Views on hateful content	40	-1.9026289	0.4067139	-2.9786785	-0.8265794
Least	Views on hateful content	41	-1.9043893	0.5036954	-3.2370246	-0.5717541
Least	Views on hateful content	42	-1.1710631	0.9723195	-3.7435448	1.4014186
Least	Views on hateful content	43	-3.2773874	0.4284099	-4.4108384	-2.1439364
Least	Views on hateful content	44	-3.0459772	0.4110862	-4.1335948	-1.9583596
Least	Views on hateful content	45	-3.0505534	0.4500816	-4.2413416	-1.8597651
Least	Views on hateful content	47	-2.6560134	0.3664745	-3.6256009	-1.6864260
Least	Views on hateful content	48	-2.4721133	0.1352251	-2.8298807	-2.1143460
Least	Views on hateful content	49	-2.3754836	0.3614929	-3.3318914	-1.4190757
Least	Views on hateful content	50	-2.6236365	0.3524881	-3.5562199	-1.6910530
Least	Views on hateful content	51	-2.6356718	0.4278929	-3.7677552	-1.5035885
Least	Views on hateful content	52	-2.5824736	0.3714773	-3.5652972	-1.5996500
Least	Views on hateful content	53	-2.6140503	0.3295871	-3.4860443	-1.7420564
Least	Views on hateful content	54	-2.6576577	0.5116213	-4.0112625	-1.3040528
Least	Views on hateful content	55	-2.5164623	0.4449708	-3.6937289	-1.3391957
Least	Views on hateful content	56	-3.3681106	0.8478323	-5.6112345	-1.1249868
Least	Views on hateful content	57	-3.0745589	0.3872878	-4.0992126	-2.0499052
Least	Views on hateful content	58	-3.2200310	0.3751821	-4.2126564	-2.2274056
Least	Views on hateful content	59	-2.9557518	0.3264679	-3.8194934	-2.0920102

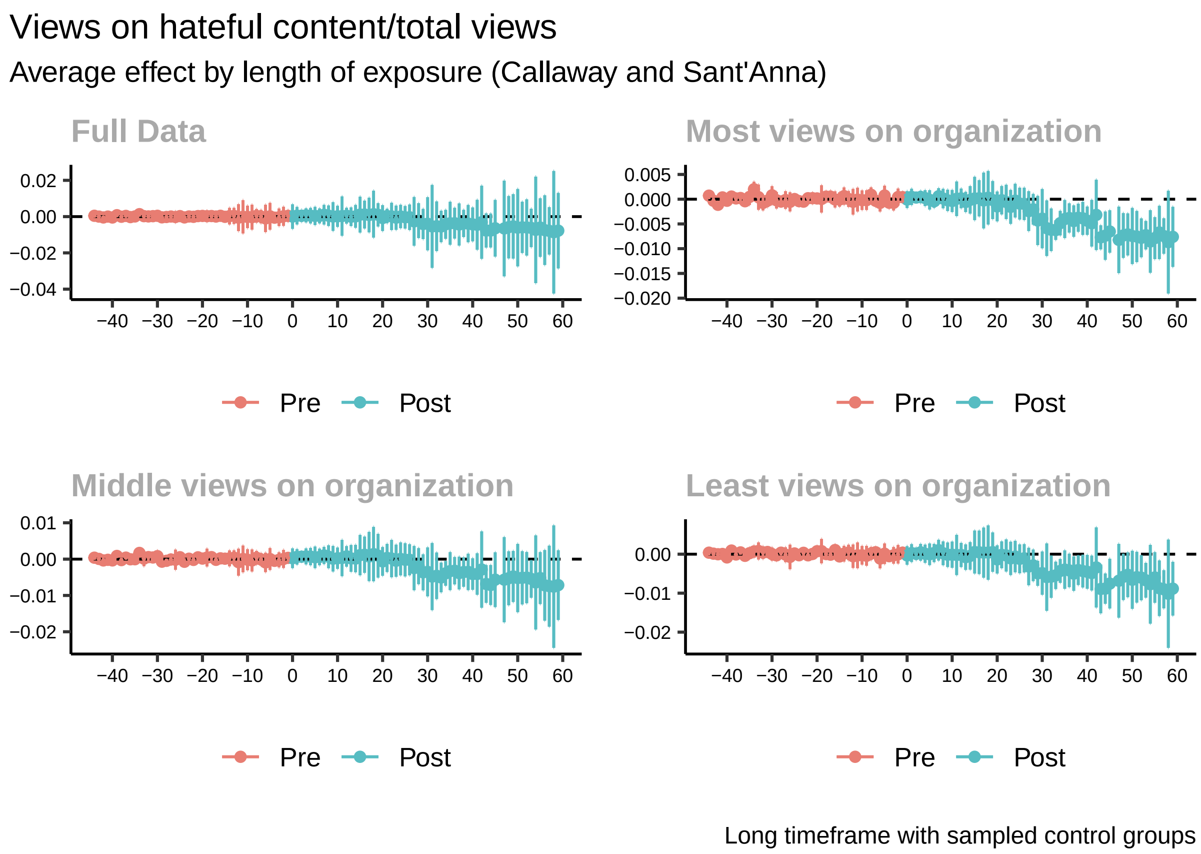

sample	outcome	event.time	estimate	std.error	conf.low	conf.high
Full	Views on hateful content/total views	-44	0.0004586	0.0002344	-0.0001289	0.0010462
Full	Views on hateful content/total views	-43	0.0000182	0.0002482	-0.0006039	0.0006402
Full	Views on hateful content/total views	-42	-0.0004571	0.0002539	-0.0010934	0.0001791
Full	Views on hateful content/total views	-41	0.0000145	0.0002571	-0.0006298	0.0006588
Full	Views on hateful content/total views	-40	-0.0004790	0.0002293	-0.0010538	0.0000957
Full	Views on hateful content/total views	-39	0.0007738	0.0002218	0.0002181	0.0013296
Full	Views on hateful content/total views	-38	-0.0000702	0.0002211	-0.0006243	0.0004839
Full	Views on hateful content/total views	-37	0.0003822	0.0002195	-0.0001680	0.0009324
Full	Views on hateful content/total views	-36	-0.0002577	0.0002701	-0.0009347	0.0004193
Full	Views on hateful content/total views	-35	0.0000872	0.0005461	-0.0012814	0.0014558
Full	Views on hateful content/total views	-34	0.0013370	0.0005567	-0.0000582	0.0027322
Full	Views on hateful content/total views	-33	0.0002605	0.0008689	-0.0019172	0.0024381
Full	Views on hateful content/total views	-32	0.0001014	0.0005083	-0.0011725	0.0013752
Full	Views on hateful content/total views	-31	0.0002374	0.0004091	-0.0007878	0.0012626
Full	Views on hateful content/total views	-30	0.0004423	0.0006756	-0.0012508	0.0021354
Full	Views on hateful content/total views	-29	-0.0003957	0.0005831	-0.0018571	0.0010656
Full	Views on hateful content/total views	-28	-0.0002090	0.0002486	-0.0008321	0.0004141
Full	Views on hateful content/total views	-27	0.0000552	0.0007930	-0.0019323	0.0020426
Full	Views on hateful content/total views	-26	-0.0002054	0.0012504	-0.0033390	0.0029283

Full	Views on hateful content/total views	-25	0.0002613	0.0004112	-0.0007691	0.0012917
Full	Views on hateful content/total views	-24	-0.0004329	0.0004221	-0.0014907	0.0006250
Full	Views on hateful content/total views	-23	0.0000700	0.0003317	-0.0007612	0.0009012
Full	Views on hateful content/total views	-22	-0.0001198	0.0002691	-0.0007942	0.0005545
Full	Views on hateful content/total views	-21	0.0002815	0.0004476	-0.0008401	0.0014032
Full	Views on hateful content/total views	-20	0.0003651	0.0004208	-0.0006895	0.0014198
Full	Views on hateful content/total views	-19	0.0002334	0.0012401	-0.0028745	0.0033412
Full	Views on hateful content/total views	-18	0.0003181	0.0005126	-0.0009665	0.0016027
Full	Views on hateful content/total views	-17	0.0000486	0.0004043	-0.0009646	0.0010618
Full	Views on hateful content/total views	-16	0.0004430	0.0004551	-0.0006977	0.0015836
Full	Views on hateful content/total views	-15	-0.0001078	0.0005284	-0.0014321	0.0012165
Full	Views on hateful content/total views	-14	0.0002595	0.0018142	-0.0042871	0.0048062
Full	Views on hateful content/total views	-13	0.0003887	0.0017681	-0.0040425	0.0048199
Full	Views on hateful content/total views	-12	-0.0005816	0.0029631	-0.0080076	0.0068444
Full	Views on hateful content/total views	-11	-0.0001019	0.0036538	-0.0092590	0.0090553
Full	Views on hateful content/total views	-10	-0.0001643	0.0024740	-0.0063645	0.0060359
Full	Views on hateful content/total views	-9	-0.0003096	0.0027519	-0.0072063	0.0065871
Full	Views on hateful content/total views	-8	0.0004066	0.0015098	-0.0033772	0.0041904
Full	Views on hateful content/total views	-7	0.0000079	0.0014755	-0.0036899	0.0037058
Full	Views on hateful content/total views	-6	-0.0010000	0.0030013	-0.0085219	0.0065218
Full	Views on hateful content/total views	-5	0.0001628	0.0029505	-0.0072317	0.0075572
Full	Views on hateful content/total views	-4	-0.0005907	0.0010137	-0.0031311	0.0019497
Full	Views on hateful content/total views	-3	-0.0003718	0.0019314	-0.0052121	0.0044686
Full	Views on hateful content/total views	-2	0.0001740	0.0020952	-0.0050768	0.0054248
Full	Views on hateful content/total views	-1	0.0003962	0.0012846	-0.0028231	0.0036155
Full	Views on hateful content/total views	0	0.0000134	0.0026929	-0.0067355	0.0067623
Full	Views on hateful content/total views	1	0.0005136	0.0019243	-0.0043089	0.0053362
Full	Views on hateful content/total views	2	0.0004591	0.0013417	-0.0029035	0.0038217
Full	Views on hateful content/total views	3	0.0006886	0.0015399	-0.0031706	0.0045479
Full	Views on hateful content/total views	4	0.0005230	0.0017035	-0.0037462	0.0047922
Full	Views on hateful content/total views	5	0.0003642	0.0020016	-0.0046522	0.0053807
Full	Views on hateful content/total views	6	0.0003822	0.0016398	-0.0037272	0.0044917
Full	Views on hateful content/total views	7	0.0010368	0.0021609	-0.0043787	0.0064524
Full	Views on hateful content/total views	8	0.0005027	0.0022495	-0.0051348	0.0061403
Full	Views on hateful content/total views	9	0.0000501	0.0031502	-0.0078447	0.0079450
Full	Views on hateful content/total views	10	0.0001603	0.0023144	-0.0056400	0.0059605
Full	Views on hateful content/total views	11	0.0002875	0.0043234	-0.0105476	0.0111225
Full	Views on hateful content/total views	12	0.0004852	0.0017439	-0.0038852	0.0048557
Full	Views on hateful content/total views	13	-0.0000654	0.0020514	-0.0052066	0.0050759
Full	Views on hateful content/total views	14	0.0001332	0.0025084	-0.0061534	0.0064198
Full	Views on hateful content/total views	15	0.0011027	0.0039509	-0.0087988	0.0110042

Full	Views on hateful content/total views	16	0.0011481	0.0030627	-0.0065275	0.0088238
Full	Views on hateful content/total views	17	0.0008659	0.0038009	-0.0086598	0.0103916
Full	Views on hateful content/total views	18	0.0013453	0.0051638	-0.0115960	0.0142866
Full	Views on hateful content/total views	19	0.0009786	0.0025635	-0.0054458	0.0074031
Full	Views on hateful content/total views	20	-0.0007883	0.0028442	-0.0079162	0.0063396
Full	Views on hateful content/total views	21	0.0003340	0.0015832	-0.0036338	0.0043017
Full	Views on hateful content/total views	22	0.0001106	0.0030007	-0.0074097	0.0076310
Full	Views on hateful content/total views	23	-0.0005920	0.0026651	-0.0072711	0.0060871
Full	Views on hateful content/total views	24	0.0000564	0.0025720	-0.0063894	0.0065022
Full	Views on hateful content/total views	25	-0.0003724	0.0024851	-0.0066006	0.0058558
Full	Views on hateful content/total views	26	-0.0003455	0.0028028	-0.0073698	0.0066787
Full	Views on hateful content/total views	27	-0.0026236	0.0053584	-0.0160528	0.0108055
Full	Views on hateful content/total views	28	-0.0022140	0.0038337	-0.0118219	0.0073938
Full	Views on hateful content/total views	29	-0.0040883	0.0033250	-0.0124213	0.0042448
Full	Views on hateful content/total views	30	-0.0039381	0.0058369	-0.0185665	0.0106902
Full	Views on hateful content/total views	31	-0.0053913	0.0091188	-0.0282445	0.0174619
Full	Views on hateful content/total views	32	-0.0053296	0.0054600	-0.0190132	0.0083540
Full	Views on hateful content/total views	33	-0.0054398	0.0034588	-0.0141080	0.0032284
Full	Views on hateful content/total views	34	-0.0042554	0.0031808	-0.0122270	0.0037162
Full	Views on hateful content/total views	35	-0.0036719	0.0047266	-0.0155176	0.0081738
Full	Views on hateful content/total views	36	-0.0036428	0.0033895	-0.0121374	0.0048519
Full	Views on hateful content/total views	37	-0.0043430	0.0046346	-0.0159580	0.0072720
Full	Views on hateful content/total views	38	-0.0037615	0.0029927	-0.0112616	0.0037386
Full	Views on hateful content/total views	39	-0.0038549	0.0041005	-0.0141313	0.0064215
Full	Views on hateful content/total views	40	-0.0042889	0.0037216	-0.0136158	0.0050380
Full	Views on hateful content/total views	41	-0.0045079	0.0054917	-0.0182711	0.0092553
Full	Views on hateful content/total views	42	-0.0031023	0.0080416	-0.0232557	0.0170511
Full	Views on hateful content/total views	43	-0.0076718	0.0037639	-0.0171048	0.0017611
Full	Views on hateful content/total views	44	-0.0076562	0.0037279	-0.0169990	0.0016866
Full	Views on hateful content/total views	45	-0.0064404	0.0062366	-0.0220703	0.0091896
Full	Views on hateful content/total views	47	-0.0065352	0.0105283	-0.0329208	0.0198504
Full	Views on hateful content/total views	48	-0.0058301	0.0068552	-0.0230103	0.0113501
Full	Views on hateful content/total views	49	-0.0053818	0.0070871	-0.0231432	0.0123796
Full	Views on hateful content/total views	50	-0.0060948	0.0085091	-0.0274199	0.0152304
Full	Views on hateful content/total views	51	-0.0059163	0.0056378	-0.0200455	0.0082128
Full	Views on hateful content/total views	52	-0.0059924	0.0061824	-0.0214864	0.0095016
Full	Views on hateful content/total views	53	-0.0062546	0.0042347	-0.0168674	0.0043583
Full	Views on hateful content/total views	54	-0.0073092	0.0117220	-0.0366864	0.0220680
Full	Views on hateful content/total views	55	-0.0060441	0.0064367	-0.0221754	0.0100872
Full	Views on hateful content/total views	56	-0.0074521	0.0076597	-0.0266485	0.0117443
Full	Views on hateful content/total views	57	-0.0078546	0.0052217	-0.0209410	0.0052318

Full	Views on hateful content/total views	58	-0.0086134	0.0134767	-0.0423881	0.0251612
Full	Views on hateful content/total views	59	-0.0077475	0.0082999	-0.0285483	0.0130533

sample	outcome	event.time	estimate	std.error	conf.low	conf.high
Most	Views on hateful content/total views	-44	0.0007178	0.0002727	0.0000054	0.0014303
Most	Views on hateful content/total views	-43	-0.0003128	0.0002361	-0.0009297	0.0003040
Most	Views on hateful content/total views	-42	-0.0011298	0.0002664	-0.0018257	-0.0004338
Most	Views on hateful content/total views	-41	0.0003595	0.0002494	-0.0002921	0.0010111
Most	Views on hateful content/total views	-40	-0.0003148	0.0002264	-0.0009064	0.0002768
Most	Views on hateful content/total views	-39	0.0005339	0.0003532	-0.0003890	0.0014568
Most	Views on hateful content/total views	-38	0.0001621	0.0002831	-0.0005777	0.0009019
Most	Views on hateful content/total views	-37	0.0002371	0.0002070	-0.0003038	0.0007780
Most	Views on hateful content/total views	-36	-0.0004139	0.0003060	-0.0012135	0.0003857
Most	Views on hateful content/total views	-35	0.0004568	0.0006198	-0.0011625	0.0020761
Most	Views on hateful content/total views	-34	0.0020139	0.0005786	0.0005022	0.0035256
Most	Views on hateful content/total views	-33	0.0004128	0.0009764	-0.0021383	0.0029640
Most	Views on hateful content/total views	-32	-0.0007489	0.0005767	-0.0022558	0.0007579
Most	Views on hateful content/total views	-31	-0.0002990	0.0005014	-0.0016090	0.0010109
Most	Views on hateful content/total views	-30	0.0007841	0.0007060	-0.0010606	0.0026288
Most	Views on hateful content/total views	-29	-0.0002171	0.0006392	-0.0018872	0.0014531
Most	Views on hateful content/total views	-28	-0.0004740	0.0003852	-0.0014803	0.0005324
Most	Views on hateful content/total views	-27	-0.0001638	0.0006831	-0.0019487	0.0016211
Most	Views on hateful content/total views	-26	-0.0005375	0.0007359	-0.0024602	0.0013853
Most	Views on hateful content/total views	-25	0.0000278	0.0003608	-0.0009149	0.0009705
Most	Views on hateful content/total views	-24	-0.0003950	0.0003941	-0.0014247	0.0006348
Most	Views on hateful content/total views	-23	-0.0004903	0.0003132	-0.0013085	0.0003280
Most	Views on hateful content/total views	-22	0.0002008	0.0002486	-0.0004488	0.0008504
Most	Views on hateful content/total views	-21	0.0002055	0.0004379	-0.0009385	0.0013495
Most	Views on hateful content/total views	-20	0.0001529	0.0003336	-0.0007188	0.0010245
Most	Views on hateful content/total views	-19	0.0000514	0.0010543	-0.0027031	0.0028060
Most	Views on hateful content/total views	-18	0.0005196	0.0004361	-0.0006197	0.0016589
Most	Views on hateful content/total views	-17	0.0005041	0.0004787	-0.0007466	0.0017548
Most	Views on hateful content/total views	-16	-0.0000528	0.0006248	-0.0016853	0.0015797
Most	Views on hateful content/total views	-15	-0.0000701	0.0005544	-0.0015186	0.0013784
Most	Views on hateful content/total views	-14	0.0006264	0.0006703	-0.0011248	0.0023777
Most	Views on hateful content/total views	-13	0.0000462	0.0006167	-0.0015651	0.0016576
Most	Views on hateful content/total views	-12	-0.0005223	0.0009946	-0.0031209	0.0020763
Most	Views on hateful content/total views	-11	-0.0000825	0.0009182	-0.0024817	0.0023166
Most	Views on hateful content/total views	-10	-0.0001977	0.0007546	-0.0021692	0.0017738
Most	Views on hateful content/total views	-9	-0.0001876	0.0007573	-0.0021662	0.0017911
Most	Views on hateful content/total views	-8	0.0008905	0.0005722	-0.0006045	0.0023856

Most	Views on hateful content/total views	-7	-0.0003122	0.0004668	-0.0015318	0.0009075
Most	Views on hateful content/total views	-6	-0.0007468	0.0006680	-0.0024920	0.0009984
Most	Views on hateful content/total views	-5	0.0007436	0.0007577	-0.0012361	0.0027233
Most	Views on hateful content/total views	-4	-0.0007068	0.0004636	-0.0019180	0.0005044
Most	Views on hateful content/total views	-3	-0.0007347	0.0005894	-0.0022745	0.0008052
Most	Views on hateful content/total views	-2	0.0004408	0.0006584	-0.0012794	0.0021611
Most	Views on hateful content/total views	-1	0.0004136	0.0004149	-0.0006703	0.0014975
Most	Views on hateful content/total views	0	-0.0000703	0.0006543	-0.0017798	0.0016392
Most	Views on hateful content/total views	1	0.0004660	0.0006291	-0.0011777	0.0021097
Most	Views on hateful content/total views	2	0.0003330	0.0002908	-0.0004269	0.0010929
Most	Views on hateful content/total views	3	0.0004502	0.0004824	-0.0008102	0.0017107
Most	Views on hateful content/total views	4	0.0002210	0.0006175	-0.0013923	0.0018344
Most	Views on hateful content/total views	5	-0.0001314	0.0007449	-0.0020775	0.0018148
Most	Views on hateful content/total views	6	-0.0004226	0.0005044	-0.0017406	0.0008953
Most	Views on hateful content/total views	7	0.0006032	0.0006221	-0.0010222	0.0022287
Most	Views on hateful content/total views	8	0.0001268	0.0007506	-0.0018344	0.0020880
Most	Views on hateful content/total views	9	-0.0002250	0.0008285	-0.0023896	0.0019396
Most	Views on hateful content/total views	10	-0.0003622	0.0008541	-0.0025939	0.0018695
Most	Views on hateful content/total views	11	0.0000831	0.0013370	-0.0034100	0.0035763
Most	Views on hateful content/total views	12	0.0001635	0.0007609	-0.0018244	0.0021515
Most	Views on hateful content/total views	13	-0.0005648	0.0007821	-0.0026081	0.0014785
Most	Views on hateful content/total views	14	-0.0001326	0.0010674	-0.0029215	0.0026562
Most	Views on hateful content/total views	15	0.0001432	0.0016765	-0.0042370	0.0045235
Most	Views on hateful content/total views	16	0.0001860	0.0013980	-0.0034667	0.0038387
Most	Views on hateful content/total views	17	-0.0002566	0.0021580	-0.0058950	0.0053817
Most	Views on hateful content/total views	18	0.0002970	0.0020694	-0.0051098	0.0057038
Most	Views on hateful content/total views	19	-0.0001728	0.0014721	-0.0040191	0.0036735
Most	Views on hateful content/total views	20	-0.0014552	0.0011515	-0.0044637	0.0015533
Most	Views on hateful content/total views	21	-0.0002014	0.0010975	-0.0030689	0.0026662
Most	Views on hateful content/total views	22	-0.0005561	0.0013279	-0.0040255	0.0029133
Most	Views on hateful content/total views	23	-0.0015510	0.0013104	-0.0049748	0.0018727
Most	Views on hateful content/total views	24	-0.0002862	0.0013011	-0.0036855	0.0031131
Most	Views on hateful content/total views	25	-0.0008879	0.0012309	-0.0041038	0.0023280
Most	Views on hateful content/total views	26	-0.0009488	0.0012512	-0.0042179	0.0023204
Most	Views on hateful content/total views	27	-0.0024109	0.0015410	-0.0064371	0.0016153
Most	Views on hateful content/total views	28	-0.0020437	0.0011888	-0.0051497	0.0010622
Most	Views on hateful content/total views	29	-0.0043079	0.0019052	-0.0092858	0.0006700
Most	Views on hateful content/total views	30	-0.0039307	0.0022918	-0.0099185	0.0020570
Most	Views on hateful content/total views	31	-0.0058595	0.0021478	-0.0114711	-0.0002479
Most	Views on hateful content/total views	32	-0.0061824	0.0016490	-0.0104908	-0.0018740
Most	Views on hateful content/total views	33	-0.0062474	0.0007554	-0.0082211	-0.0042736

Most	Views on hateful content/total views	34	-0.0048375	0.0009460	-0.0073092	-0.0023657
Most	Views on hateful content/total views	35	-0.0040139	0.0014757	-0.0078696	-0.0001581
Most	Views on hateful content/total views	36	-0.0038012	0.0011332	-0.0067619	-0.0008406
Most	Views on hateful content/total views	37	-0.0044247	0.0012151	-0.0075994	-0.0012500
Most	Views on hateful content/total views	38	-0.0037494	0.0010848	-0.0065836	-0.0009151
Most	Views on hateful content/total views	39	-0.0038697	0.0012777	-0.0072079	-0.0005314
Most	Views on hateful content/total views	40	-0.0043427	0.0010952	-0.0072043	-0.0014812
Most	Views on hateful content/total views	41	-0.0048584	0.0018034	-0.0095701	-0.0001467
Most	Views on hateful content/total views	42	-0.0031728	0.0027190	-0.0102769	0.0039312
Most	Views on hateful content/total views	43	-0.0077053	0.0009281	-0.0101303	-0.0052804
Most	Views on hateful content/total views	44	-0.0073749	0.0018695	-0.0122595	-0.0024904
Most	Views on hateful content/total views	45	-0.0065283	0.0016424	-0.0108195	-0.0022371
Most	Views on hateful content/total views	47	-0.0082218	0.0025728	-0.0149439	-0.0014996
Most	Views on hateful content/total views	48	-0.0073306	0.0017338	-0.0118606	-0.0028006
Most	Views on hateful content/total views	49	-0.0071176	0.0016235	-0.0113592	-0.0028759
Most	Views on hateful content/total views	50	-0.0074358	0.0021701	-0.0131056	-0.0017660
Most	Views on hateful content/total views	51	-0.0075460	0.0019782	-0.0127144	-0.0023776
Most	Views on hateful content/total views	52	-0.0078103	0.0015018	-0.0117339	-0.0038866
Most	Views on hateful content/total views	53	-0.0072716	0.0010986	-0.0101419	-0.0044013
Most	Views on hateful content/total views	54	-0.0085443	0.0024193	-0.0148653	-0.0022234
Most	Views on hateful content/total views	55	-0.0078698	0.0016255	-0.0121167	-0.0036229
Most	Views on hateful content/total views	56	-0.0067271	0.0020609	-0.0121118	-0.0013425
Most	Views on hateful content/total views	57	-0.0074477	0.0013712	-0.0110303	-0.0038651
Most	Views on hateful content/total views	58	-0.0086765	0.0039727	-0.0190560	0.0017030
Most	Views on hateful content/total views	59	-0.0076100	0.0023219	-0.0136765	-0.0015435

sample	outcome	event.time	estimate	std.error	conf.low	conf.high
Middle	Views on hateful content/total views	-44	0.0003968	0.0002535	-0.0002342	0.0010278
Middle	Views on hateful content/total views	-43	0.0000586	0.0002981	-0.0006834	0.0008006
Middle	Views on hateful content/total views	-42	-0.0003584	0.0003378	-0.0011992	0.0004823
Middle	Views on hateful content/total views	-41	-0.0001884	0.0002907	-0.0009119	0.0005352
Middle	Views on hateful content/total views	-40	-0.0003494	0.0002304	-0.0009227	0.0002239
Middle	Views on hateful content/total views	-39	0.0008733	0.0002210	0.0003234	0.0014232
Middle	Views on hateful content/total views	-38	-0.0002628	0.0002528	-0.0008920	0.0003663
Middle	Views on hateful content/total views	-37	0.0004128	0.0002191	-0.0001325	0.0009582
Middle	Views on hateful content/total views	-36	-0.0000459	0.0002594	-0.0006916	0.0005998
Middle	Views on hateful content/total views	-35	-0.0000281	0.0004976	-0.0012665	0.0012103
Middle	Views on hateful content/total views	-34	0.0017092	0.0004468	0.0005973	0.0028210
Middle	Views on hateful content/total views	-33	0.0001305	0.0008392	-0.0019581	0.0022190
Middle	Views on hateful content/total views	-32	0.0005621	0.0004588	-0.0005798	0.0017040
Middle	Views on hateful content/total views	-31	0.0004516	0.0003234	-0.0003533	0.0012564

Middle	Views on hateful content/total views	-30	0.0008165	0.0006278	-0.0007458	0.0023788
Middle	Views on hateful content/total views	-29	-0.0006988	0.0005314	-0.0020213	0.0006237
Middle	Views on hateful content/total views	-28	-0.0004615	0.0002327	-0.0010407	0.0001177
Middle	Views on hateful content/total views	-27	-0.0001045	0.0005527	-0.0014800	0.0012709
Middle	Views on hateful content/total views	-26	-0.0001676	0.0011134	-0.0029385	0.0026033
Middle	Views on hateful content/total views	-25	0.0005239	0.0003779	-0.0004166	0.0014645
Middle	Views on hateful content/total views	-24	-0.0007224	0.0003905	-0.0016943	0.0002495
Middle	Views on hateful content/total views	-23	0.0001134	0.0003777	-0.0008264	0.0010533
Middle	Views on hateful content/total views	-22	-0.0002101	0.0002817	-0.0009111	0.0004908
Middle	Views on hateful content/total views	-21	0.0004950	0.0003591	-0.0003987	0.0013886
Middle	Views on hateful content/total views	-20	0.0001508	0.0003677	-0.0007643	0.0010659
Middle	Views on hateful content/total views	-19	0.0004292	0.0009850	-0.0020221	0.0028805
Middle	Views on hateful content/total views	-18	0.0005497	0.0004749	-0.0006323	0.0017316
Middle	Views on hateful content/total views	-17	-0.0001899	0.0002832	-0.0008946	0.0005148
Middle	Views on hateful content/total views	-16	0.0002144	0.0004253	-0.0008441	0.0012729
Middle	Views on hateful content/total views	-15	0.0001156	0.0004819	-0.0010838	0.0013149
Middle	Views on hateful content/total views	-14	0.0001995	0.0008798	-0.0019901	0.0023891
Middle	Views on hateful content/total views	-13	0.0003920	0.0008219	-0.0016534	0.0024374
Middle	Views on hateful content/total views	-12	-0.0008379	0.0014891	-0.0045438	0.0028681
Middle	Views on hateful content/total views	-11	0.0000453	0.0014774	-0.0036314	0.0037220
Middle	Views on hateful content/total views	-10	-0.0001575	0.0011612	-0.0030471	0.0027322
Middle	Views on hateful content/total views	-9	-0.0003660	0.0012204	-0.0034032	0.0026713
Middle	Views on hateful content/total views	-8	0.0003808	0.0007570	-0.0015032	0.0022647
Middle	Views on hateful content/total views	-7	-0.0000765	0.0007032	-0.0018264	0.0016734
Middle	Views on hateful content/total views	-6	-0.0009002	0.0010695	-0.0035618	0.0017615
Middle	Views on hateful content/total views	-5	0.0000230	0.0012078	-0.0029829	0.0030288
Middle	Views on hateful content/total views	-4	-0.0004696	0.0004903	-0.0016897	0.0007505
Middle	Views on hateful content/total views	-3	-0.0001885	0.0008358	-0.0022685	0.0018915
Middle	Views on hateful content/total views	-2	0.0000565	0.0010014	-0.0024358	0.0025487
Middle	Views on hateful content/total views	-1	0.0003481	0.0004924	-0.0008773	0.0015736
Middle	Views on hateful content/total views	0	0.0002467	0.0010899	-0.0024658	0.0029592
Middle	Views on hateful content/total views	1	0.0006259	0.0008540	-0.0014993	0.0027512
Middle	Views on hateful content/total views	2	0.0006680	0.0005687	-0.0007473	0.0020833
Middle	Views on hateful content/total views	3	0.0007540	0.0008895	-0.0014596	0.0029675
Middle	Views on hateful content/total views	4	0.0006942	0.0009573	-0.0016883	0.0030767
Middle	Views on hateful content/total views	5	0.0004885	0.0011920	-0.0024779	0.0034550
Middle	Views on hateful content/total views	6	0.0005954	0.0009196	-0.0016932	0.0028841
Middle	Views on hateful content/total views	7	0.0010210	0.0009506	-0.0013446	0.0033866
Middle	Views on hateful content/total views	8	0.0006527	0.0012705	-0.0025092	0.0038146
Middle	Views on hateful content/total views	9	0.0001086	0.0014102	-0.0034010	0.0036182
Middle	Views on hateful content/total views	10	0.0002443	0.0011776	-0.0026863	0.0031750

Middle	Views on hateful content/total views	11	0.0003198	0.0020053	-0.0046707	0.0053102
Middle	Views on hateful content/total views	12	0.0007577	0.0011351	-0.0020671	0.0035824
Middle	Views on hateful content/total views	13	0.0001945	0.0014820	-0.0034937	0.0038827
Middle	Views on hateful content/total views	14	0.0001565	0.0015202	-0.0036269	0.0039398
Middle	Views on hateful content/total views	15	0.0011065	0.0022355	-0.0044570	0.0066699
Middle	Views on hateful content/total views	16	0.0012052	0.0020022	-0.0037775	0.0061880
Middle	Views on hateful content/total views	17	0.0007305	0.0027205	-0.0060400	0.0075010
Middle	Views on hateful content/total views	18	0.0013817	0.0030056	-0.0060981	0.0088615
Middle	Views on hateful content/total views	19	0.0009429	0.0024196	-0.0050787	0.0069645
Middle	Views on hateful content/total views	20	-0.0006255	0.0015855	-0.0045714	0.0033203
Middle	Views on hateful content/total views	21	0.0003299	0.0015561	-0.0035428	0.0042025
Middle	Views on hateful content/total views	22	0.0001478	0.0020684	-0.0049997	0.0052954
Middle	Views on hateful content/total views	23	-0.0004394	0.0017753	-0.0048574	0.0039786
Middle	Views on hateful content/total views	24	0.0000557	0.0018247	-0.0044852	0.0045966
Middle	Views on hateful content/total views	25	-0.0001876	0.0018371	-0.0047595	0.0043842
Middle	Views on hateful content/total views	26	-0.0001792	0.0017225	-0.0044660	0.0041076
Middle	Views on hateful content/total views	27	-0.0024710	0.0024439	-0.0085528	0.0036109
Middle	Views on hateful content/total views	28	-0.0019341	0.0019921	-0.0068916	0.0030235
Middle	Views on hateful content/total views	29	-0.0036331	0.0019967	-0.0086022	0.0013360
Middle	Views on hateful content/total views	30	-0.0035127	0.0027104	-0.0102578	0.0032323
Middle	Views on hateful content/total views	31	-0.0048021	0.0037072	-0.0140279	0.0044237
Middle	Views on hateful content/total views	32	-0.0045918	0.0025707	-0.0109893	0.0018057
Middle	Views on hateful content/total views	33	-0.0050524	0.0016428	-0.0091407	-0.0009642
Middle	Views on hateful content/total views	34	-0.0039171	0.0013667	-0.0073182	-0.0005160
Middle	Views on hateful content/total views	35	-0.0032951	0.0020971	-0.0085142	0.0019239
Middle	Views on hateful content/total views	36	-0.0031920	0.0015129	-0.0069571	0.0005731
Middle	Views on hateful content/total views	37	-0.0038391	0.0018091	-0.0083414	0.0006632
Middle	Views on hateful content/total views	38	-0.0035474	0.0016296	-0.0076028	0.0005080
Middle	Views on hateful content/total views	39	-0.0035138	0.0020091	-0.0085138	0.0014862
Middle	Views on hateful content/total views	40	-0.0040963	0.0017428	-0.0084335	0.0002409
Middle	Views on hateful content/total views	41	-0.0040861	0.0022930	-0.0097925	0.0016202
Middle	Views on hateful content/total views	42	-0.0028619	0.0042186	-0.0133605	0.0076367
Middle	Views on hateful content/total views	43	-0.0068438	0.0020837	-0.0120294	-0.0016582
Middle	Views on hateful content/total views	44	-0.0071273	0.0022078	-0.0126217	-0.0016329
Middle	Views on hateful content/total views	45	-0.0056548	0.0030289	-0.0131928	0.0018831
Middle	Views on hateful content/total views	47	-0.0056575	0.0047078	-0.0173736	0.0060586
Middle	Views on hateful content/total views	48	-0.0052618	0.0029832	-0.0126859	0.0021622
Middle	Views on hateful content/total views	49	-0.0047767	0.0028231	-0.0118024	0.0022490
Middle	Views on hateful content/total views	50	-0.0052005	0.0037736	-0.0145917	0.0041907
Middle	Views on hateful content/total views	51	-0.0051430	0.0029465	-0.0124758	0.0021898
Middle	Views on hateful content/total views	52	-0.0050904	0.0028297	-0.0121324	0.0019516

Middle	Views on hateful content/total views	53	-0.0054387	0.0020803	-0.0106158	-0.0002617
Middle	Views on hateful content/total views	54	-0.0064163	0.0052038	-0.0193667	0.0065341
Middle	Views on hateful content/total views	55	-0.0052175	0.0028721	-0.0123652	0.0019302
Middle	Views on hateful content/total views	56	-0.0072150	0.0039012	-0.0169237	0.0024936
Middle	Views on hateful content/total views	57	-0.0074431	0.0044778	-0.0185868	0.0037005
Middle	Views on hateful content/total views	58	-0.0075724	0.0067755	-0.0244342	0.0092893
Middle	Views on hateful content/total views	59	-0.0071525	0.0038556	-0.0167477	0.0024426

sample	outcome	event.time	estimate	std.error	conf.low	conf.high
Least	Views on hateful content/total views	-44	0.0003872	0.0002681	-0.0003108	0.0010853
Least	Views on hateful content/total views	-43	0.0001377	0.0002766	-0.0005825	0.0008578
Least	Views on hateful content/total views	-42	-0.0000115	0.0003316	-0.0008747	0.0008518
Least	Views on hateful content/total views	-41	0.0000508	0.0002712	-0.0006553	0.0007568
Least	Views on hateful content/total views	-40	-0.0008281	0.0002149	-0.0013875	-0.0002686
Least	Views on hateful content/total views	-39	0.0009310	0.0002007	0.0004083	0.0014537
Least	Views on hateful content/total views	-38	-0.0000782	0.0002062	-0.0006150	0.0004587
Least	Views on hateful content/total views	-37	0.0004594	0.0002367	-0.0001570	0.0010758
Least	Views on hateful content/total views	-36	-0.0004165	0.0003271	-0.0012680	0.0004350
Least	Views on hateful content/total views	-35	0.0002520	0.0005541	-0.0011907	0.0016947
Least	Views on hateful content/total views	-34	0.0006869	0.0005973	-0.0008682	0.0022420
Least	Views on hateful content/total views	-33	0.0008255	0.0008407	-0.0013634	0.0030144
Least	Views on hateful content/total views	-32	0.0004078	0.0006282	-0.0012279	0.0020436
Least	Views on hateful content/total views	-31	0.0005634	0.0004515	-0.0006121	0.0017389
Least	Views on hateful content/total views	-30	0.0000332	0.0006533	-0.0016679	0.0017342
Least	Views on hateful content/total views	-29	-0.0003033	0.0005346	-0.0016952	0.0010885
Least	Views on hateful content/total views	-28	0.0003807	0.0002824	-0.0003545	0.0011159
Least	Views on hateful content/total views	-27	-0.0001187	0.0007311	-0.0020223	0.0017849
Least	Views on hateful content/total views	-26	-0.0006940	0.0012046	-0.0038303	0.0024422
Least	Views on hateful content/total views	-25	0.0001824	0.0004011	-0.0008619	0.0012266
Least	Views on hateful content/total views	-24	-0.0002758	0.0003883	-0.0012867	0.0007351
Least	Views on hateful content/total views	-23	0.0003459	0.0003289	-0.0005105	0.0012024
Least	Views on hateful content/total views	-22	-0.0002901	0.0003184	-0.0011191	0.0005389
Least	Views on hateful content/total views	-21	0.0000602	0.0004912	-0.0012188	0.0013391
Least	Views on hateful content/total views	-20	0.0007555	0.0005171	-0.0005908	0.0021019
Least	Views on hateful content/total views	-19	0.0007756	0.0012017	-0.0023532	0.0039045
Least	Views on hateful content/total views	-18	0.0000355	0.0004776	-0.0012081	0.0012791
Least	Views on hateful content/total views	-17	-0.0001211	0.0003952	-0.0011500	0.0009078
Least	Views on hateful content/total views	-16	0.0010608	0.0005505	-0.0003724	0.0024940
Least	Views on hateful content/total views	-15	-0.0005773	0.0003541	-0.0014993	0.0003448
Least	Views on hateful content/total views	-14	0.0001345	0.0007751	-0.0018834	0.0021525
Least	Views on hateful content/total views	-13	0.0004336	0.0007639	-0.0015553	0.0024224

Least	Views on hateful content/total views	-12	-0.0005265	0.0011534	-0.0035295	0.0024764
Least	Views on hateful content/total views	-11	-0.0002974	0.0012672	-0.0035969	0.0030021
Least	Views on hateful content/total views	-10	-0.0002799	0.0009164	-0.0026660	0.0021061
Least	Views on hateful content/total views	-9	-0.0004022	0.0009774	-0.0029470	0.0021425
Least	Views on hateful content/total views	-8	0.0001632	0.0006436	-0.0015126	0.0018390
Least	Views on hateful content/total views	-7	0.0004969	0.0005201	-0.0008571	0.0018510
Least	Views on hateful content/total views	-6	-0.0011257	0.0009664	-0.0036419	0.0013906
Least	Views on hateful content/total views	-5	0.0001127	0.0010085	-0.0025130	0.0027385
Least	Views on hateful content/total views	-4	-0.0005213	0.0004264	-0.0016316	0.0005889
Least	Views on hateful content/total views	-3	-0.0002953	0.0008159	-0.0024197	0.0018291
Least	Views on hateful content/total views	-2	-0.0000231	0.0009107	-0.0023941	0.0023480
Least	Views on hateful content/total views	-1	0.0003946	0.0004809	-0.0008575	0.0016468
Least	Views on hateful content/total views	0	-0.0003282	0.0009118	-0.0027022	0.0020458
Least	Views on hateful content/total views	1	0.0002875	0.0008786	-0.0020001	0.0025752
Least	Views on hateful content/total views	2	0.0000953	0.0006144	-0.0015043	0.0016949
Least	Views on hateful content/total views	3	0.0004818	0.0008247	-0.0016656	0.0026291
Least	Views on hateful content/total views	4	0.0001090	0.0008983	-0.0022298	0.0024478
Least	Views on hateful content/total views	5	-0.0000305	0.0010615	-0.0027943	0.0027333
Least	Views on hateful content/total views	6	0.0003039	0.0008956	-0.0020279	0.0026357
Least	Views on hateful content/total views	7	0.0010332	0.0010228	-0.0016297	0.0036961
Least	Views on hateful content/total views	8	0.0002312	0.0011718	-0.0028199	0.0032823
Least	Views on hateful content/total views	9	-0.0001476	0.0012036	-0.0032815	0.0029863
Least	Views on hateful content/total views	10	-0.0001348	0.0012897	-0.0034928	0.0032233
Least	Views on hateful content/total views	11	-0.0001905	0.0019823	-0.0053516	0.0049706
Least	Views on hateful content/total views	12	-0.0001927	0.0011912	-0.0032941	0.0029087
Least	Views on hateful content/total views	13	-0.0007643	0.0012575	-0.0040383	0.0025097
Least	Views on hateful content/total views	14	-0.0004297	0.0013540	-0.0039550	0.0030955
Least	Views on hateful content/total views	15	0.0005504	0.0021148	-0.0049557	0.0060566
Least	Views on hateful content/total views	16	0.0004753	0.0021377	-0.0050906	0.0060412
Least	Views on hateful content/total views	17	0.0004006	0.0024664	-0.0060209	0.0068222
Least	Views on hateful content/total views	18	0.0004092	0.0026801	-0.0065688	0.0073872
Least	Views on hateful content/total views	19	0.0005302	0.0019405	-0.0045222	0.0055826
Least	Views on hateful content/total views	20	-0.0013379	0.0013890	-0.0049543	0.0022785
Least	Views on hateful content/total views	21	-0.0001622	0.0013253	-0.0036128	0.0032884
Least	Views on hateful content/total views	22	-0.0003215	0.0015846	-0.0044473	0.0038042
Least	Views on hateful content/total views	23	-0.0010752	0.0016408	-0.0053473	0.0031969
Least	Views on hateful content/total views	24	-0.0006096	0.0015539	-0.0046554	0.0034362
Least	Views on hateful content/total views	25	-0.0011946	0.0014266	-0.0049088	0.0025197
Least	Views on hateful content/total views	26	-0.0010562	0.0013944	-0.0046868	0.0025743
Least	Views on hateful content/total views	27	-0.0031581	0.0018465	-0.0079658	0.0016496
Least	Views on hateful content/total views	28	-0.0028663	0.0015528	-0.0069093	0.0011767

Least	Views on hateful content/total views	29	-0.0049114	0.0012329	-0.0081214	-0.0017013
Least	Views on hateful content/total views	30	-0.0048594	0.0021254	-0.0103933	0.0006744
Least	Views on hateful content/total views	31	-0.0058766	0.0033153	-0.0145083	0.0027552
Least	Views on hateful content/total views	32	-0.0057651	0.0020868	-0.0111984	-0.0003318
Least	Views on hateful content/total views	33	-0.0054360	0.0013463	-0.0089412	-0.0019307
Least	Views on hateful content/total views	34	-0.0042977	0.0011741	-0.0073546	-0.0012409
Least	Views on hateful content/total views	35	-0.0039475	0.0018921	-0.0088739	0.0009790
Least	Views on hateful content/total views	36	-0.0041594	0.0016452	-0.0084429	0.0001241
Least	Views on hateful content/total views	37	-0.0049918	0.0016966	-0.0094091	-0.0005745
Least	Views on hateful content/total views	38	-0.0040722	0.0014723	-0.0079055	-0.0002390
Least	Views on hateful content/total views	39	-0.0043244	0.0016178	-0.0085366	-0.0001122
Least	Views on hateful content/total views	40	-0.0045202	0.0016668	-0.0088600	-0.0001804
Least	Views on hateful content/total views	41	-0.0048405	0.0017151	-0.0093061	-0.0003749
Least	Views on hateful content/total views	42	-0.0034006	0.0039453	-0.0136726	0.0068715
Least	Views on hateful content/total views	43	-0.0089176	0.0024062	-0.0151825	-0.0026528
Least	Views on hateful content/total views	44	-0.0088348	0.0014878	-0.0127085	-0.0049610
Least	Views on hateful content/total views	45	-0.0075514	0.0024492	-0.0139281	-0.0011747
Least	Views on hateful content/total views	47	-0.0068123	0.0036330	-0.0162713	0.0026466
Least	Views on hateful content/total views	48	-0.0058028	0.0022855	-0.0117533	0.0001477
Least	Views on hateful content/total views	49	-0.0052830	0.0022078	-0.0110313	0.0004653
Least	Views on hateful content/total views	50	-0.0065680	0.0028605	-0.0140157	0.0008797
Least	Views on hateful content/total views	51	-0.0058974	0.0025039	-0.0124167	0.0006219
Least	Views on hateful content/total views	52	-0.0060239	0.0022238	-0.0118138	-0.0002340
Least	Views on hateful content/total views	53	-0.0066883	0.0017138	-0.0111505	-0.0022260
Least	Views on hateful content/total views	54	-0.0077052	0.0038792	-0.0178053	0.0023949
Least	Views on hateful content/total views	55	-0.0059697	0.0024904	-0.0124539	0.0005145
Least	Views on hateful content/total views	56	-0.0087377	0.0027352	-0.0158591	-0.0016164
Least	Views on hateful content/total views	57	-0.0090093	0.0018800	-0.0139042	-0.0041144
Least	Views on hateful content/total views	58	-0.0101533	0.0053321	-0.0240362	0.0037297
Least	Views on hateful content/total views	59	-0.0088470	0.0026480	-0.0157414	-0.0019525

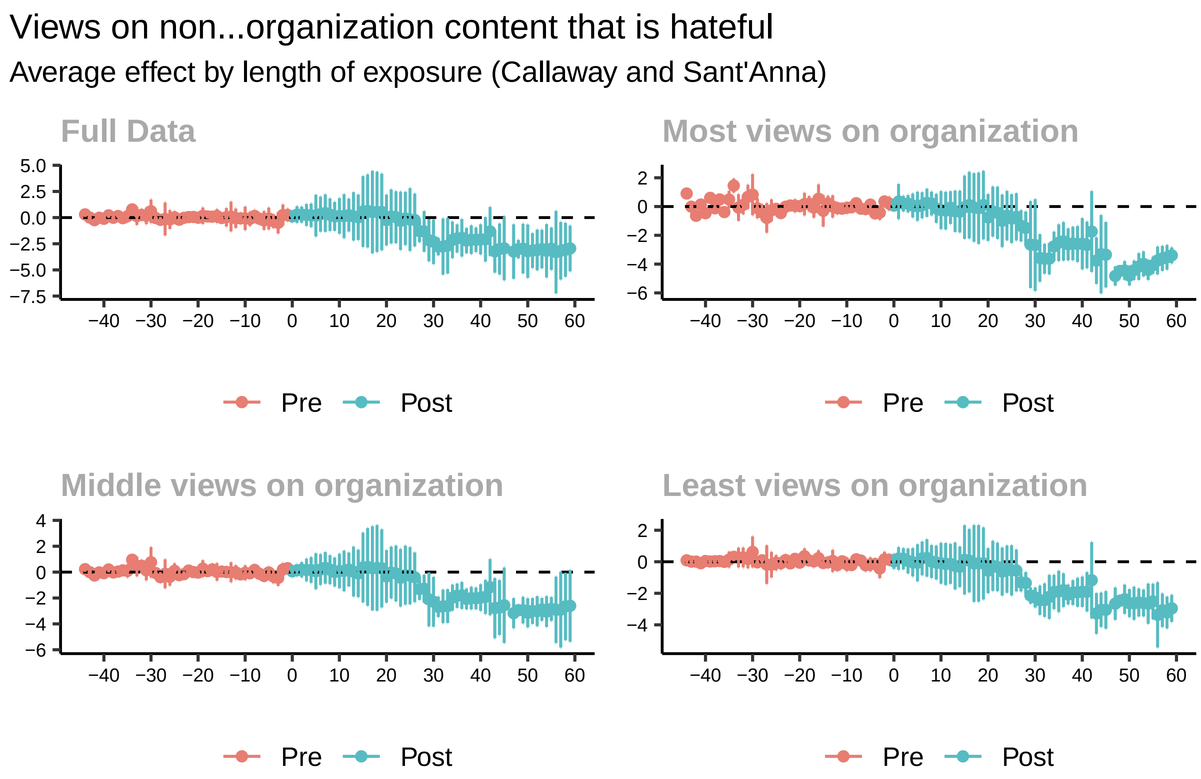

Long timeframe with sampled control groups

sample	outcome	event.time	estimate	std.error	conf.low	conf.high
Full	Views on nonorganization content that is hateful	-44	0.3049943	0.0944609	0.0614106	0.5485779
Full	Views on nonorganization content that is hateful	-43	0.0000492	0.0434559	-0.1120093	0.1121078
Full	Views on nonorganization content that is hateful	-42	-0.2447495	0.0513552	-0.3771777	-0.1123213
Full	Views on nonorganization content that is hateful	-41	-0.0101672	0.0547488	-0.1513464	0.1310119
Full	Views on nonorganization content that is hateful	-40	-0.1127269	0.0760267	-0.3087749	0.0833211
Full	Views on nonorganization content that is hateful	-39	0.2127826	0.0935392	-0.0284243	0.4539894
Full	Views on nonorganization content that is hateful	-38	-0.0175684	0.0683602	-0.1938468	0.1587100
Full	Views on nonorganization content that is hateful	-37	0.1489747	0.0396944	0.0466159	0.2513335
Full	Views on nonorganization content that is hateful	-36	-0.0553597	0.0880132	-0.2823167	0.1715973
Full	Views on nonorganization content that is hateful	-35	0.1316237	0.2179460	-0.4303873	0.6936348
Full	Views on nonorganization content that is hateful	-34	0.7831011	0.1217539	0.4691378	1.0970643
Full	Views on nonorganization content that is hateful	-33	0.1367278	0.3265131	-0.7052423	0.9786979
Full	Views on nonorganization content that is hateful	-32	0.2642559	0.2173873	-0.2963146	0.8248264
Full	Views on nonorganization content that is hateful	-31	0.1706719	0.3076104	-0.6225543	0.9638982
Full	Views on nonorganization content that is hateful	-30	0.6085684	0.4341927	-0.5110721	1.7282088
Full	Views on nonorganization content that is hateful	-29	-0.0442637	0.1436279	-0.4146328	0.3261054
Full	Views on nonorganization content that is hateful	-28	-0.2130210	0.1105577	-0.4981131	0.0720711
Full	Views on nonorganization content that is hateful	-27	-0.1274677	0.6078701	-1.6949652	1.4400298
Full	Views on nonorganization content that is hateful	-26	-0.1791103	0.3511394	-1.0845834	0.7263628

Full	Views on nonorganization content that is hateful	-25	0.0199371	0.2331908	-0.5813853	0.6212595
Full	Views on nonorganization content that is hateful	-24	-0.2030403	0.1571679	-0.6083248	0.2022442
Full	Views on nonorganization content that is hateful	-23	-0.0206124	0.1004252	-0.2795759	0.2383512
Full	Views on nonorganization content that is hateful	-22	0.0484472	0.0707565	-0.1340106	0.2309051
Full	Views on nonorganization content that is hateful	-21	0.0581422	0.0864382	-0.1647535	0.2810379
Full	Views on nonorganization content that is hateful	-20	-0.0130278	0.0911712	-0.2481283	0.2220728
Full	Views on nonorganization content that is hateful	-19	0.1769232	0.3042747	-0.6077013	0.9615478
Full	Views on nonorganization content that is hateful	-18	0.1089728	0.0667699	-0.0632049	0.2811506
Full	Views on nonorganization content that is hateful	-17	0.0843771	0.1530663	-0.3103308	0.4790849
Full	Views on nonorganization content that is hateful	-16	0.1528727	0.2547448	-0.5040305	0.8097759
Full	Views on nonorganization content that is hateful	-15	-0.0422316	0.1552600	-0.4425961	0.3581329
Full	Views on nonorganization content that is hateful	-14	0.0349643	0.3400182	-0.8418309	0.9117596
Full	Views on nonorganization content that is hateful	-13	0.1023261	0.5484787	-1.3120203	1.5166725
Full	Views on nonorganization content that is hateful	-12	-0.0688643	0.3560039	-0.9868815	0.8491529
Full	Views on nonorganization content that is hateful	-11	-0.0681430	0.1991430	-0.5816674	0.4453813
Full	Views on nonorganization content that is hateful	-10	-0.0778151	0.4332934	-1.1951364	1.0395062
Full	Views on nonorganization content that is hateful	-9	-0.1246665	0.2641382	-0.8057923	0.5564592
Full	Views on nonorganization content that is hateful	-8	0.1598385	0.2215740	-0.4115281	0.7312051
Full	Views on nonorganization content that is hateful	-7	-0.1037414	0.2059056	-0.6347044	0.4272216
Full	Views on nonorganization content that is hateful	-6	-0.2723072	0.3480065	-1.1697017	0.6250872
Full	Views on nonorganization content that is hateful	-5	-0.0861382	0.4027046	-1.1245812	0.9523048
Full	Views on nonorganization content that is hateful	-4	-0.3421069	0.2561159	-1.0025459	0.3183320
Full	Views on nonorganization content that is hateful	-3	-0.5039304	0.3908378	-1.5117727	0.5039119
Full	Views on nonorganization content that is hateful	-2	0.2449653	0.3908460	-0.7628982	1.2528288
Full	Views on nonorganization content that is hateful	-1	0.2439827	0.2348011	-0.3614923	0.8494576
Full	Views on nonorganization content that is hateful	0	0.1107680	0.2038931	-0.4150053	0.6365412
Full	Views on nonorganization content that is hateful	1	0.2849938	0.3079697	-0.5091589	1.0791465
Full	Views on nonorganization content that is hateful	2	0.2838190	0.2956499	-0.4785649	1.0462029
Full	Views on nonorganization content that is hateful	3	0.2524511	0.4052336	-0.7925133	1.2974154
Full	Views on nonorganization content that is hateful	4	0.2181117	0.4363407	-0.9070677	1.3432911
Full	Views on nonorganization content that is hateful	5	0.1851456	0.7717918	-1.8050519	2.1753432
Full	Views on nonorganization content that is hateful	6	0.3273691	0.6456313	-1.3375020	1.9922403
Full	Views on nonorganization content that is hateful	7	0.4322736	0.6922110	-1.3527112	2.2172584
Full	Views on nonorganization content that is hateful	8	0.2750661	0.6018709	-1.2769612	1.8270933
Full	Views on nonorganization content that is hateful	9	0.1146686	0.5343398	-1.2632183	1.4925554
Full	Views on nonorganization content that is hateful	10	0.1563981	0.6576292	-1.5394116	1.8522079
Full	Views on nonorganization content that is hateful	11	0.1373836	0.8134958	-1.9603550	2.2351222
Full	Views on nonorganization content that is hateful	12	0.2333098	0.6082763	-1.3352350	1.8018546
Full	Views on nonorganization content that is hateful	13	0.1147994	0.8952398	-2.1937300	2.4233288
Full	Views on nonorganization content that is hateful	14	0.0242060	0.8398286	-2.1414361	2.1898481
Full	Views on nonorganization content that is hateful	15	0.5759987	1.3116874	-2.8064123	3.9584097

Full	Views on nonorganization content that is hateful	16	0.6348119	1.3537575	-2.8560841	4.1257079
Full	Views on nonorganization content that is hateful	17	0.5293357	1.5262126	-3.4062653	4.4649368
Full	Views on nonorganization content that is hateful	18	0.5443913	1.4864509	-3.2886775	4.3774601
Full	Views on nonorganization content that is hateful	19	0.5345232	1.4181236	-3.1223517	4.1913982
Full	Views on nonorganization content that is hateful	20	-0.2368533	0.9369428	-2.6529212	2.1792146
Full	Views on nonorganization content that is hateful	21	0.1050453	1.0012324	-2.4768042	2.6868948
Full	Views on nonorganization content that is hateful	22	0.0362168	0.9604159	-2.4403803	2.5128138
Full	Views on nonorganization content that is hateful	23	-0.3104692	1.0800321	-3.0955172	2.4745788
Full	Views on nonorganization content that is hateful	24	-0.0856356	0.9858516	-2.6278230	2.4565518
Full	Views on nonorganization content that is hateful	25	-0.1864081	1.1644258	-3.1890797	2.8162635
Full	Views on nonorganization content that is hateful	26	-0.2316377	0.9762439	-2.7490501	2.2857746
Full	Views on nonorganization content that is hateful	27	-1.2901971	0.4109907	-2.3500071	-0.2303870
Full	Views on nonorganization content that is hateful	28	-1.3223497	0.7500078	-3.2563735	0.6116742
Full	Views on nonorganization content that is hateful	29	-2.1585921	0.7808503	-4.1721485	-0.1450356
Full	Views on nonorganization content that is hateful	30	-2.3145798	0.8263468	-4.4454568	-0.1837027
Full	Views on nonorganization content that is hateful	31	-2.7736980	0.3216843	-3.6032162	-1.9441797
Full	Views on nonorganization content that is hateful	32	-2.7670436	1.0408450	-5.4510411	-0.0830460
Full	Views on nonorganization content that is hateful	33	-2.6807237	1.0322857	-5.3426496	-0.0187978
Full	Views on nonorganization content that is hateful	34	-2.1281827	0.6770458	-3.8740615	-0.3823040
Full	Views on nonorganization content that is hateful	35	-1.9952394	0.5209196	-3.3385200	-0.6519589
Full	Views on nonorganization content that is hateful	36	-1.9349638	0.6946635	-3.7262728	-0.1436548
Full	Views on nonorganization content that is hateful	37	-2.1961492	0.4689425	-3.4053978	-0.9869006
Full	Views on nonorganization content that is hateful	38	-2.1102339	0.5291026	-3.4746159	-0.7458520
Full	Views on nonorganization content that is hateful	39	-2.1215815	0.4530648	-3.2898867	-0.9532763
Full	Views on nonorganization content that is hateful	40	-2.0827497	0.5580394	-3.5217500	-0.6437494
Full	Views on nonorganization content that is hateful	41	-2.0795962	0.8225640	-4.2007185	0.0415261
Full	Views on nonorganization content that is hateful	42	-1.3175542	0.9044855	-3.6499253	1.0148169
Full	Views on nonorganization content that is hateful	43	-3.2239855	0.7873094	-5.2541979	-1.1937731
Full	Views on nonorganization content that is hateful	44	-3.0027085	0.9440989	-5.4372294	-0.5681876
Full	Views on nonorganization content that is hateful	45	-2.9419259	1.1809727	-5.9872666	0.1034149
Full	Views on nonorganization content that is hateful	47	-3.2441427	1.0076698	-5.8425923	-0.6456931
Full	Views on nonorganization content that is hateful	48	-3.0120148	0.3392374	-3.8867967	-2.1372328
Full	Views on nonorganization content that is hateful	49	-2.9664951	0.9183805	-5.3346969	-0.5982934
Full	Views on nonorganization content that is hateful	50	-3.2132847	0.9881381	-5.7613682	-0.6652011
Full	Views on nonorganization content that is hateful	51	-3.1522455	0.6444254	-4.8140069	-1.4904840
Full	Views on nonorganization content that is hateful	52	-3.0934008	0.7512236	-5.0305597	-1.1562419
Full	Views on nonorganization content that is hateful	53	-3.0058155	0.7128731	-4.8440810	-1.1675499
Full	Views on nonorganization content that is hateful	54	-3.1790281	0.9872223	-5.7247501	-0.6333061
Full	Views on nonorganization content that is hateful	55	-2.9740075	0.7851513	-4.9986547	-0.9493602
Full	Views on nonorganization content that is hateful	56	-3.3001532	1.5265668	-7.2366677	0.6363613
Full	Views on nonorganization content that is hateful	57	-3.1704377	1.0651521	-5.9171152	-0.4237601

Full	Views on nonorganization content that is hateful	58	-3.0824191	0.9990788	-5.6587153	-0.5061229
Full	Views on nonorganization content that is hateful	59	-2.9485085	0.8457044	-5.1293025	-0.7677145

sample	outcome	event.time	estimate	std.error	conf.low	conf.high
Most	Views on nonorganization content that is hateful	-44	0.9028382	0.1015294	0.6352310	1.1704453
Most	Views on nonorganization content that is hateful	-43	-0.0117749	0.0497893	-0.1430077	0.1194580
Most	Views on nonorganization content that is hateful	-42	-0.6443516	0.0825870	-0.8620311	-0.4266721
Most	Views on nonorganization content that is hateful	-41	0.1356200	0.0848400	-0.0879980	0.3592381
Most	Views on nonorganization content that is hateful	-40	-0.4618525	0.0644747	-0.6317924	-0.2919125
Most	Views on nonorganization content that is hateful	-39	0.6033268	0.0967860	0.3482222	0.8584315
Most	Views on nonorganization content that is hateful	-38	-0.1227041	0.1071530	-0.4051338	0.1597257
Most	Views on nonorganization content that is hateful	-37	0.4936024	0.0423369	0.3820125	0.6051923
Most	Views on nonorganization content that is hateful	-36	-0.3839289	0.0840511	-0.6054676	-0.1623901
Most	Views on nonorganization content that is hateful	-35	0.4856263	0.2284212	-0.1164375	1.0876901
Most	Views on nonorganization content that is hateful	-34	1.4527115	0.1832317	0.9697565	1.9356665
Most	Views on nonorganization content that is hateful	-33	-0.0721458	0.3525604	-1.0014108	0.8571193
Most	Views on nonorganization content that is hateful	-32	0.1170948	0.2481730	-0.5370301	0.7712198
Most	Views on nonorganization content that is hateful	-31	0.6735606	0.3173346	-0.1628577	1.5099789
Most	Views on nonorganization content that is hateful	-30	0.8258057	0.5409862	-0.6001048	2.2517161
Most	Views on nonorganization content that is hateful	-29	-0.1151429	0.2652055	-0.8141612	0.5838755
Most	Views on nonorganization content that is hateful	-28	-0.4081475	0.2132946	-0.9703411	0.1540461
Most	Views on nonorganization content that is hateful	-27	-0.7907718	0.3883987	-1.8144982	0.2329546
Most	Views on nonorganization content that is hateful	-26	-0.2663744	0.2910995	-1.0336431	0.5008943
Most	Views on nonorganization content that is hateful	-25	-0.1629327	0.1211337	-0.4822124	0.1563469
Most	Views on nonorganization content that is hateful	-24	-0.4512599	0.1403720	-0.8212469	-0.0812729
Most	Views on nonorganization content that is hateful	-23	-0.0112543	0.1012966	-0.2782478	0.2557393
Most	Views on nonorganization content that is hateful	-22	0.0718648	0.0919060	-0.1703774	0.3141070
Most	Views on nonorganization content that is hateful	-21	0.0539749	0.1715521	-0.3981956	0.5061453
Most	Views on nonorganization content that is hateful	-20	0.0247378	0.1328056	-0.3253061	0.3747817
Most	Views on nonorganization content that is hateful	-19	0.1649830	0.2983086	-0.6212872	0.9512532
Most	Views on nonorganization content that is hateful	-18	0.1918533	0.2031516	-0.3436059	0.7273125
Most	Views on nonorganization content that is hateful	-17	-0.0760451	0.2647824	-0.7739483	0.6218580
Most	Views on nonorganization content that is hateful	-16	0.5388691	0.3788889	-0.4597916	1.5375297
Most	Views on nonorganization content that is hateful	-15	-0.2969028	0.4127760	-1.3848818	0.7910762
Most	Views on nonorganization content that is hateful	-14	0.2183861	0.1999556	-0.3086493	0.7454214
Most	Views on nonorganization content that is hateful	-13	0.0047638	0.2930063	-0.7675309	0.7770585
Most	Views on nonorganization content that is hateful	-12	-0.1174354	0.1558243	-0.5281511	0.2932802
Most	Views on nonorganization content that is hateful	-11	-0.1434382	0.0911548	-0.3837004	0.0968240
Most	Views on nonorganization content that is hateful	-10	-0.0883631	0.1483531	-0.4793865	0.3026603
Most	Views on nonorganization content that is hateful	-9	-0.0529817	0.1135047	-0.3521530	0.2461895
Most	Views on nonorganization content that is hateful	-8	0.2303131	0.1172241	-0.0786618	0.5392879

Most	Views on nonorganization content that is hateful	-7	-0.1499754	0.1117308	-0.4444710	0.1445203
Most	Views on nonorganization content that is hateful	-6	-0.1790506	0.1225996	-0.5021939	0.1440927
Most	Views on nonorganization content that is hateful	-5	0.1327544	0.1325244	-0.2165484	0.4820572
Most	Views on nonorganization content that is hateful	-4	-0.4764298	0.1300277	-0.8191516	-0.1337079
Most	Views on nonorganization content that is hateful	-3	-0.4259620	0.1927013	-0.9338766	0.0819527
Most	Views on nonorganization content that is hateful	-2	0.3501776	0.1296567	0.0084335	0.6919217
Most	Views on nonorganization content that is hateful	-1	0.2063441	0.1815089	-0.2720701	0.6847582
Most	Views on nonorganization content that is hateful	0	0.0686928	0.1380299	-0.2951211	0.4325066
Most	Views on nonorganization content that is hateful	1	0.3463907	0.4616481	-0.8704036	1.5631851
Most	Views on nonorganization content that is hateful	2	0.1982843	0.2062376	-0.3453090	0.7418775
Most	Views on nonorganization content that is hateful	3	0.1990434	0.2273297	-0.4001435	0.7982302
Most	Views on nonorganization content that is hateful	4	0.0755847	0.3139971	-0.7520366	0.9032061
Most	Views on nonorganization content that is hateful	5	0.0152978	0.3829505	-0.9940683	1.0246639
Most	Views on nonorganization content that is hateful	6	0.0897906	0.3418740	-0.8113076	0.9908888
Most	Views on nonorganization content that is hateful	7	0.2592722	0.3697992	-0.7154303	1.2339748
Most	Views on nonorganization content that is hateful	8	0.2095509	0.3122456	-0.6134540	1.0325558
Most	Views on nonorganization content that is hateful	9	-0.1296534	0.3820663	-1.1366891	0.8773824
Most	Views on nonorganization content that is hateful	10	-0.2452150	0.5011378	-1.5660948	1.0756647
Most	Views on nonorganization content that is hateful	11	-0.2838473	0.4938528	-1.5855256	1.0178309
Most	Views on nonorganization content that is hateful	12	-0.0753213	0.4303613	-1.2096509	1.0590083
Most	Views on nonorganization content that is hateful	13	-0.3307381	0.5437371	-1.7638992	1.1024231
Most	Views on nonorganization content that is hateful	14	-0.3523099	0.5494372	-1.8004953	1.0958755
Most	Views on nonorganization content that is hateful	15	-0.0501539	0.8348700	-2.2506720	2.1503641
Most	Views on nonorganization content that is hateful	16	0.0967325	0.8777559	-2.2168227	2.4102877
Most	Views on nonorganization content that is hateful	17	-0.0638319	0.9024189	-2.4423930	2.3147292
Most	Views on nonorganization content that is hateful	18	-0.1160385	0.9419004	-2.5986633	2.3665864
Most	Views on nonorganization content that is hateful	19	0.1040009	0.9006737	-2.2699601	2.4779618
Most	Views on nonorganization content that is hateful	20	-0.7571291	0.6169370	-2.3832279	0.8689696
Most	Views on nonorganization content that is hateful	21	-0.3466974	0.6597686	-2.0856899	1.3922951
Most	Views on nonorganization content that is hateful	22	-0.4370803	0.6875525	-2.2493046	1.3751439
Most	Views on nonorganization content that is hateful	23	-0.9910453	0.6924380	-2.8161467	0.8340561
Most	Views on nonorganization content that is hateful	24	-0.6305863	0.6503824	-2.3448392	1.0836666
Most	Views on nonorganization content that is hateful	25	-0.8380921	0.6435025	-2.5342112	0.8580270
Most	Views on nonorganization content that is hateful	26	-0.7168293	0.6211986	-2.3541606	0.9205020
Most	Views on nonorganization content that is hateful	27	-1.3407470	0.4114608	-2.4252596	-0.2562344
Most	Views on nonorganization content that is hateful	28	-1.4864181	0.4328412	-2.6272844	-0.3455519
Most	Views on nonorganization content that is hateful	29	-2.6405541	1.1455542	-5.6599616	0.3788533
Most	Views on nonorganization content that is hateful	30	-2.6793084	1.2088512	-5.8655517	0.5069350
Most	Views on nonorganization content that is hateful	31	-3.5731133	0.6237860	-5.2172643	-1.9289623
Most	Views on nonorganization content that is hateful	32	-3.6305524	0.3928294	-4.6659571	-2.5951477
Most	Views on nonorganization content that is hateful	33	-3.6201454	0.4123280	-4.7069436	-2.5333472

Most	Views on nonorganization content that is hateful	34	-2.7680400	0.3867708	-3.7874755	-1.7486045
Most	Views on nonorganization content that is hateful	35	-2.5138609	0.4843114	-3.7903901	-1.2373316
Most	Views on nonorganization content that is hateful	36	-2.4159148	0.5084711	-3.7561234	-1.0757062
Most	Views on nonorganization content that is hateful	37	-2.6270616	0.4196506	-3.7331605	-1.5209626
Most	Views on nonorganization content that is hateful	38	-2.5640700	0.4398226	-3.7233376	-1.4048024
Most	Views on nonorganization content that is hateful	39	-2.6014280	0.4744996	-3.8520958	-1.3507602
Most	Views on nonorganization content that is hateful	40	-2.5858833	0.6739363	-4.3622187	-0.8095479
Most	Views on nonorganization content that is hateful	41	-2.6710318	0.6123281	-4.2849825	-1.0570811
Most	Views on nonorganization content that is hateful	42	-1.7382628	1.0656586	-4.5470846	1.0705590
Most	Views on nonorganization content that is hateful	43	-3.7573305	0.6130533	-5.3731927	-2.1414682
Most	Views on nonorganization content that is hateful	44	-3.3091936	1.0319054	-6.0290501	-0.5893371
Most	Views on nonorganization content that is hateful	45	-3.3476799	0.8631929	-5.6228505	-1.0725094
Most	Views on nonorganization content that is hateful	47	-4.8269663	0.2507334	-5.4878398	-4.1660928
Most	Views on nonorganization content that is hateful	48	-4.4768498	0.0852371	-4.7015145	-4.2521850
Most	Views on nonorganization content that is hateful	49	-4.4411037	0.2481596	-5.0951933	-3.7870141
Most	Views on nonorganization content that is hateful	50	-4.7941180	0.2583995	-5.4751973	-4.1130386
Most	Views on nonorganization content that is hateful	51	-4.4220525	0.2535913	-5.0904587	-3.7536463
Most	Views on nonorganization content that is hateful	52	-4.1693978	0.3531648	-5.1002559	-3.2385396
Most	Views on nonorganization content that is hateful	53	-3.9875108	0.3292649	-4.8553744	-3.1196471
Most	Views on nonorganization content that is hateful	54	-4.4037558	0.2639296	-5.0994112	-3.7081004
Most	Views on nonorganization content that is hateful	55	-4.0787910	0.2219136	-4.6637024	-3.4938795
Most	Views on nonorganization content that is hateful	56	-3.7453483	0.3667219	-4.7119397	-2.7787569
Most	Views on nonorganization content that is hateful	57	-3.6056767	0.3302972	-4.4762612	-2.7350921
Most	Views on nonorganization content that is hateful	58	-3.5275578	0.3258986	-4.3865486	-2.6685670
Most	Views on nonorganization content that is hateful	59	-3.3928202	0.2094091	-3.9447725	-2.8408679

sample	outcome	event.time	estimate	std.error	conf.low	conf.high
Middle	Views on nonorganization content that is hateful	-44	0.2284093	0.0779858	0.0211300	0.4356886
Middle	Views on nonorganization content that is hateful	-43	-0.0019370	0.0488578	-0.1317965	0.1279225
Middle	Views on nonorganization content that is hateful	-42	-0.2596545	0.0632184	-0.4276834	-0.0916257
Middle	Views on nonorganization content that is hateful	-41	-0.0218325	0.0609110	-0.1837283	0.1400633
Middle	Views on nonorganization content that is hateful	-40	-0.0795324	0.0695809	-0.2644720	0.1054072
Middle	Views on nonorganization content that is hateful	-39	0.2028153	0.0860455	-0.0258858	0.4315164
Middle	Views on nonorganization content that is hateful	-38	-0.0262172	0.0692910	-0.2103863	0.1579520
Middle	Views on nonorganization content that is hateful	-37	0.0412408	0.0456235	-0.0800224	0.1625039
Middle	Views on nonorganization content that is hateful	-36	0.1345733	0.0816084	-0.0823344	0.3514809
Middle	Views on nonorganization content that is hateful	-35	0.0953668	0.1920889	-0.4151880	0.6059216
Middle	Views on nonorganization content that is hateful	-34	0.9556047	0.1186744	0.6401789	1.2710305
Middle	Views on nonorganization content that is hateful	-33	0.4289809	0.2824322	-0.3216983	1.1796600
Middle	Views on nonorganization content that is hateful	-32	0.4938579	0.1669932	0.0500052	0.9377107
Middle	Views on nonorganization content that is hateful	-31	0.1410256	0.2904208	-0.6308863	0.9129375

Middle	Views on nonorganization content that is hateful	-30	0.7618863	0.4406131	-0.4092232	1.9329959
Middle	Views on nonorganization content that is hateful	-29	-0.0731730	0.1262891	-0.4088379	0.2624920
Middle	Views on nonorganization content that is hateful	-28	-0.3919541	0.1485944	-0.7869044	0.0029962
Middle	Views on nonorganization content that is hateful	-27	-0.1288315	0.4189871	-1.2424610	0.9847980
Middle	Views on nonorganization content that is hateful	-26	-0.3649738	0.2566909	-1.0472349	0.3172873
Middle	Views on nonorganization content that is hateful	-25	0.0981188	0.2177237	-0.4805710	0.6768086
Middle	Views on nonorganization content that is hateful	-24	-0.2385809	0.1379407	-0.6052147	0.1280530
Middle	Views on nonorganization content that is hateful	-23	-0.1558146	0.0867146	-0.3862941	0.0746649
Middle	Views on nonorganization content that is hateful	-22	0.1193083	0.0639203	-0.0505859	0.2892025
Middle	Views on nonorganization content that is hateful	-21	0.0010794	0.0883847	-0.2338391	0.2359979
Middle	Views on nonorganization content that is hateful	-20	-0.0349848	0.0873421	-0.2671322	0.1971626
Middle	Views on nonorganization content that is hateful	-19	0.2669484	0.2447742	-0.3836390	0.9175358
Middle	Views on nonorganization content that is hateful	-18	0.0751510	0.0747133	-0.1234302	0.2737322
Middle	Views on nonorganization content that is hateful	-17	0.2076485	0.1611369	-0.2206387	0.6359357
Middle	Views on nonorganization content that is hateful	-16	0.0167497	0.2541332	-0.6587131	0.6922124
Middle	Views on nonorganization content that is hateful	-15	0.0437607	0.1290885	-0.2993449	0.3868662
Middle	Views on nonorganization content that is hateful	-14	0.0100876	0.1414127	-0.3657744	0.3859496
Middle	Views on nonorganization content that is hateful	-13	-0.0079337	0.2887635	-0.7754407	0.7595733
Middle	Views on nonorganization content that is hateful	-12	-0.0155480	0.2065861	-0.5646349	0.5335390
Middle	Views on nonorganization content that is hateful	-11	-0.1729105	0.0972461	-0.4313818	0.0855607
Middle	Views on nonorganization content that is hateful	-10	-0.0517266	0.2151716	-0.6236329	0.5201798
Middle	Views on nonorganization content that is hateful	-9	-0.0890893	0.1274864	-0.4279365	0.2497579
Middle	Views on nonorganization content that is hateful	-8	0.1694593	0.1549110	-0.2422800	0.5811985
Middle	Views on nonorganization content that is hateful	-7	-0.1215154	0.1180736	-0.4353443	0.1923135
Middle	Views on nonorganization content that is hateful	-6	-0.2980012	0.1513817	-0.7003598	0.1043575
Middle	Views on nonorganization content that is hateful	-5	-0.0280039	0.1630706	-0.4614306	0.4054228
Middle	Views on nonorganization content that is hateful	-4	-0.3397874	0.1960039	-0.8607480	0.1811731
Middle	Views on nonorganization content that is hateful	-3	-0.4456123	0.2316705	-1.0613713	0.1701467
Middle	Views on nonorganization content that is hateful	-2	0.2009374	0.1881422	-0.2991274	0.7010022
Middle	Views on nonorganization content that is hateful	-1	0.3020005	0.1349295	-0.0566298	0.6606308
Middle	Views on nonorganization content that is hateful	0	0.0601096	0.1510140	-0.3412718	0.4614910
Middle	Views on nonorganization content that is hateful	1	0.1425858	0.1707422	-0.3112315	0.5964031
Middle	Views on nonorganization content that is hateful	2	0.1880102	0.2214574	-0.4006034	0.7766237
Middle	Views on nonorganization content that is hateful	3	0.1669510	0.3263596	-0.7004829	1.0343849
Middle	Views on nonorganization content that is hateful	4	0.1628470	0.3729734	-0.8284822	1.1541762
Middle	Views on nonorganization content that is hateful	5	0.0675894	0.5203738	-1.3155167	1.4506954
Middle	Views on nonorganization content that is hateful	6	0.1992772	0.4473188	-0.9896554	1.3882098
Middle	Views on nonorganization content that is hateful	7	0.3309370	0.4593355	-0.8899348	1.5518088
Middle	Views on nonorganization content that is hateful	8	0.1862259	0.4196670	-0.9292108	1.3016625
Middle	Views on nonorganization content that is hateful	9	0.0085421	0.4214609	-1.1116624	1.1287466
Middle	Views on nonorganization content that is hateful	10	0.0872690	0.4991651	-1.2394664	1.4140044

Middle	Views on nonorganization content that is hateful	11	0.0864520	0.5884469	-1.4775861	1.6504901
Middle	Views on nonorganization content that is hateful	12	0.2001524	0.4932522	-1.1108670	1.5111718
Middle	Views on nonorganization content that is hateful	13	0.0389528	0.7233942	-1.8837631	1.9616686
Middle	Views on nonorganization content that is hateful	14	-0.1021873	0.6879804	-1.9307763	1.7264018
Middle	Views on nonorganization content that is hateful	15	0.3585133	1.0115591	-2.3301184	3.0471451
Middle	Views on nonorganization content that is hateful	16	0.4024846	1.1283369	-2.5965318	3.4015010
Middle	Views on nonorganization content that is hateful	17	0.2947150	1.2225681	-2.9547594	3.5441893
Middle	Views on nonorganization content that is hateful	18	0.3280439	1.2445498	-2.9798558	3.6359435
Middle	Views on nonorganization content that is hateful	19	0.2899933	1.1345053	-2.7254182	3.3054048
Middle	Views on nonorganization content that is hateful	20	-0.3319519	0.7618131	-2.3567816	1.6928778
Middle	Views on nonorganization content that is hateful	21	-0.0328307	0.7519531	-2.0314534	1.9657919
Middle	Views on nonorganization content that is hateful	22	-0.1094129	0.8118593	-2.2672608	2.0484351
Middle	Views on nonorganization content that is hateful	23	-0.4354070	0.8376703	-2.6618582	1.7910441
Middle	Views on nonorganization content that is hateful	24	-0.2326501	0.8576938	-2.5123219	2.0470216
Middle	Views on nonorganization content that is hateful	25	-0.2746430	0.8299943	-2.4806922	1.9314061
Middle	Views on nonorganization content that is hateful	26	-0.3993732	0.7223164	-2.3192242	1.5204779
Middle	Views on nonorganization content that is hateful	27	-1.3080091	0.3125083	-2.1386276	-0.4773906
Middle	Views on nonorganization content that is hateful	28	-1.2803069	0.4044905	-2.3554056	-0.2052081
Middle	Views on nonorganization content that is hateful	29	-2.0986242	0.7850482	-4.1852107	-0.0120377
Middle	Views on nonorganization content that is hateful	30	-2.2925178	0.7232753	-4.2149176	-0.3701181
Middle	Views on nonorganization content that is hateful	31	-2.6857700	0.2954126	-3.4709498	-1.9005902
Middle	Views on nonorganization content that is hateful	32	-2.6475729	0.4861277	-3.9396559	-1.3554899
Middle	Views on nonorganization content that is hateful	33	-2.6237850	0.4879960	-3.9208339	-1.3267362
Middle	Views on nonorganization content that is hateful	34	-1.9719394	0.3781587	-2.9770508	-0.9668281
Middle	Views on nonorganization content that is hateful	35	-1.8195900	0.3528119	-2.7573319	-0.8818480
Middle	Views on nonorganization content that is hateful	36	-1.7970117	0.3905274	-2.8349979	-0.7590255
Middle	Views on nonorganization content that is hateful	37	-2.0724249	0.3005161	-2.8711694	-1.2736805
Middle	Views on nonorganization content that is hateful	38	-2.0054042	0.3268014	-2.8740125	-1.1367959
Middle	Views on nonorganization content that is hateful	39	-2.0272716	0.3153210	-2.8653661	-1.1891771
Middle	Views on nonorganization content that is hateful	40	-1.9708343	0.3876326	-3.0011264	-0.9405422
Middle	Views on nonorganization content that is hateful	41	-1.9189679	0.4942646	-3.2326780	-0.6052578
Middle	Views on nonorganization content that is hateful	42	-1.1629490	0.8150788	-3.3293540	1.0034561
Middle	Views on nonorganization content that is hateful	43	-2.7816879	0.8747568	-5.1067116	-0.4566642
Middle	Views on nonorganization content that is hateful	44	-2.7403843	0.7976359	-4.8604278	-0.6203408
Middle	Views on nonorganization content that is hateful	45	-2.5613387	1.0963231	-5.4752654	0.3525880
Middle	Views on nonorganization content that is hateful	47	-3.1734376	0.4368791	-4.3346224	-2.0122527
Middle	Views on nonorganization content that is hateful	48	-2.9428380	0.1538620	-3.3517892	-2.5338868
Middle	Views on nonorganization content that is hateful	49	-2.9402324	0.3857594	-3.9655458	-1.9149190
Middle	Views on nonorganization content that is hateful	50	-3.1450143	0.4213647	-4.2649633	-2.0250654
Middle	Views on nonorganization content that is hateful	51	-2.9983276	0.3751305	-3.9953902	-2.0012649
Middle	Views on nonorganization content that is hateful	52	-3.0254878	0.4350215	-4.1817352	-1.8692404

Middle	Views on nonorganization content that is hateful	53	-2.8803292	0.3301755	-3.7579056	-2.0027529
Middle	Views on nonorganization content that is hateful	54	-3.0503328	0.4475762	-4.2399494	-1.8607161
Middle	Views on nonorganization content that is hateful	55	-2.8470147	0.3455749	-3.7655212	-1.9285081
Middle	Views on nonorganization content that is hateful	56	-2.9157941	0.9629571	-5.4752461	-0.3563421
Middle	Views on nonorganization content that is hateful	57	-2.8999387	1.1009376	-5.8261304	0.0262530
Middle	Views on nonorganization content that is hateful	58	-2.6529310	0.9799655	-5.2575900	-0.0482721
Middle	Views on nonorganization content that is hateful	59	-2.6041986	1.0420355	-5.3738339	0.1654366

sample	outcome	event.time	estimate	std.error	conf.low	conf.high
Least	Views on nonorganization content that is hateful	-44	0.0889137	0.0985427	-0.1724643	0.3502917
Least	Views on nonorganization content that is hateful	-43	0.0083944	0.0723673	-0.1835552	0.2003441
Least	Views on nonorganization content that is hateful	-42	0.0103021	0.0704042	-0.1764403	0.1970446
Least	Views on nonorganization content that is hateful	-41	-0.0918864	0.0848789	-0.3170222	0.1332494
Least	Views on nonorganization content that is hateful	-40	0.0455610	0.0873866	-0.1862262	0.2773482
Least	Views on nonorganization content that is hateful	-39	0.0147353	0.0962962	-0.2406840	0.2701546
Least	Views on nonorganization content that is hateful	-38	0.0305449	0.0619490	-0.1337708	0.1948606
Least	Views on nonorganization content that is hateful	-37	0.0420841	0.0461702	-0.0803795	0.1645476
Least	Views on nonorganization content that is hateful	-36	-0.0057757	0.0760561	-0.2075096	0.1959582
Least	Views on nonorganization content that is hateful	-35	0.1355632	0.1896383	-0.3674401	0.6385665
Least	Views on nonorganization content that is hateful	-34	0.3130993	0.1038157	0.0377349	0.5884637
Least	Views on nonorganization content that is hateful	-33	0.2655132	0.2364112	-0.3615520	0.8925785
Least	Views on nonorganization content that is hateful	-32	0.2732977	0.2358829	-0.3523663	0.8989618
Least	Views on nonorganization content that is hateful	-31	0.1688040	0.2190543	-0.4122232	0.7498313
Least	Views on nonorganization content that is hateful	-30	0.6215212	0.3704018	-0.3609453	1.6039877
Least	Views on nonorganization content that is hateful	-29	-0.0349158	0.1025608	-0.3069518	0.2371201
Least	Views on nonorganization content that is hateful	-28	0.0928936	0.0707013	-0.0946369	0.2804241
Least	Views on nonorganization content that is hateful	-27	-0.1784747	0.4596573	-1.3976856	1.0407362
Least	Views on nonorganization content that is hateful	-26	-0.1846186	0.3053230	-0.9944678	0.6252307
Least	Views on nonorganization content that is hateful	-25	-0.0639861	0.1830946	-0.5496325	0.4216603
Least	Views on nonorganization content that is hateful	-24	-0.0946536	0.1502114	-0.4930795	0.3037723
Least	Views on nonorganization content that is hateful	-23	0.1058585	0.1362423	-0.2555153	0.4672322
Least	Views on nonorganization content that is hateful	-22	-0.1038056	0.1111418	-0.3986021	0.1909908
Least	Views on nonorganization content that is hateful	-21	0.1820744	0.1002080	-0.0837207	0.4478696
Least	Views on nonorganization content that is hateful	-20	-0.0588880	0.0842998	-0.2824876	0.1647115
Least	Views on nonorganization content that is hateful	-19	0.3527459	0.1875407	-0.1446936	0.8501854
Least	Views on nonorganization content that is hateful	-18	0.0812711	0.1041530	-0.1949879	0.3575300
Least	Views on nonorganization content that is hateful	-17	0.0243410	0.1462008	-0.3634471	0.4121292
Least	Views on nonorganization content that is hateful	-16	0.2293041	0.1864181	-0.2651578	0.7237659
Least	Views on nonorganization content that is hateful	-15	-0.0789341	0.0881019	-0.3126186	0.1547504
Least	Views on nonorganization content that is hateful	-14	-0.0427411	0.1318600	-0.3924912	0.3070090
Least	Views on nonorganization content that is hateful	-13	0.0513424	0.2655144	-0.6529172	0.7556020

Least	Views on nonorganization content that is hateful	-12	-0.2060017	0.1454642	-0.5918360	0.1798327
Least	Views on nonorganization content that is hateful	-11	0.0295559	0.0820131	-0.1879785	0.2470902
Least	Views on nonorganization content that is hateful	-10	-0.1588217	0.1651669	-0.5969161	0.2792728
Least	Views on nonorganization content that is hateful	-9	-0.2094038	0.1172664	-0.5204453	0.1016376
Least	Views on nonorganization content that is hateful	-8	0.1548899	0.1098382	-0.1364487	0.4462286
Least	Views on nonorganization content that is hateful	-7	0.0732188	0.1034479	-0.2011701	0.3476076
Least	Views on nonorganization content that is hateful	-6	-0.2216082	0.1482215	-0.6147559	0.1715395
Least	Views on nonorganization content that is hateful	-5	-0.1618133	0.1660335	-0.6022065	0.2785798
Least	Views on nonorganization content that is hateful	-4	-0.1424287	0.1387879	-0.5105545	0.2256972
Least	Views on nonorganization content that is hateful	-3	-0.4278839	0.2298952	-1.0376658	0.1818980
Least	Views on nonorganization content that is hateful	-2	0.1521978	0.1799179	-0.3250226	0.6294182
Least	Views on nonorganization content that is hateful	-1	0.1291167	0.1315970	-0.2199358	0.4781692
Least	Views on nonorganization content that is hateful	0	0.0259639	0.1795197	-0.4502004	0.5021281
Least	Views on nonorganization content that is hateful	1	0.2087783	0.2715990	-0.5116202	0.9291768
Least	Views on nonorganization content that is hateful	2	0.1935650	0.2521310	-0.4751960	0.8623259
Least	Views on nonorganization content that is hateful	3	0.0511566	0.2971881	-0.7371154	0.8394286
Least	Views on nonorganization content that is hateful	4	-0.0265186	0.3387464	-0.9250213	0.8719842
Least	Views on nonorganization content that is hateful	5	-0.0671706	0.4418752	-1.2392155	1.1048744
Least	Views on nonorganization content that is hateful	6	0.2124678	0.4013117	-0.8519852	1.2769207
Least	Views on nonorganization content that is hateful	7	0.2406463	0.4458975	-0.9420677	1.4233603
Least	Views on nonorganization content that is hateful	8	0.0160609	0.3943279	-1.0298680	1.0619898
Least	Views on nonorganization content that is hateful	9	-0.0529050	0.4033166	-1.1226758	1.0168658
Least	Views on nonorganization content that is hateful	10	-0.0939421	0.4873507	-1.3866079	1.1987237
Least	Views on nonorganization content that is hateful	11	-0.1461021	0.5022594	-1.4783122	1.1861080
Least	Views on nonorganization content that is hateful	12	-0.1311114	0.4764401	-1.3948378	1.1326149
Least	Views on nonorganization content that is hateful	13	-0.2656731	0.5603868	-1.7520626	1.2207163
Least	Views on nonorganization content that is hateful	14	-0.3216599	0.4840058	-1.6054537	0.9621339
Least	Views on nonorganization content that is hateful	15	0.1228207	0.8259644	-2.0679959	2.3136373
Least	Views on nonorganization content that is hateful	16	0.0701616	0.7572375	-1.9383614	2.0786846
Least	Views on nonorganization content that is hateful	17	-0.1073331	0.9172309	-2.5402282	2.3255621
Least	Views on nonorganization content that is hateful	18	-0.1038020	0.9149025	-2.5305211	2.3229170
Least	Views on nonorganization content that is hateful	19	-0.1022491	0.8590371	-2.3807889	2.1762906
Least	Views on nonorganization content that is hateful	20	-0.5618561	0.5379586	-1.9887562	0.8650440
Least	Views on nonorganization content that is hateful	21	-0.2579522	0.5966898	-1.8406328	1.3247284
Least	Views on nonorganization content that is hateful	22	-0.3310175	0.5775762	-1.8630005	1.2009655
Least	Views on nonorganization content that is hateful	23	-0.6224676	0.5726528	-2.1413916	0.8964565
Least	Views on nonorganization content that is hateful	24	-0.4663654	0.5641250	-1.9626701	1.0299393
Least	Views on nonorganization content that is hateful	25	-0.5461885	0.6012600	-2.1409915	1.0486145
Least	Views on nonorganization content that is hateful	26	-0.5494174	0.4988450	-1.8725712	0.7737364
Least	Views on nonorganization content that is hateful	27	-1.3044693	0.2187797	-1.8847681	-0.7241705
Least	Views on nonorganization content that is hateful	28	-1.3459475	0.2600375	-2.0356801	-0.6562150

Least	Views on nonorganization content that is hateful	29	-2.1419102	0.1948250	-2.6586709	-1.6251495
Least	Views on nonorganization content that is hateful	30	-2.2687508	0.2389761	-2.9026193	-1.6348824
Least	Views on nonorganization content that is hateful	31	-2.4379800	0.3488322	-3.3632345	-1.5127255
Least	Views on nonorganization content that is hateful	32	-2.4330890	0.4000567	-3.4942131	-1.3719648
Least	Views on nonorganization content that is hateful	33	-2.2318913	0.5267880	-3.6291621	-0.8346204
Least	Views on nonorganization content that is hateful	34	-1.9165989	0.4090598	-3.0016033	-0.8315945
Least	Views on nonorganization content that is hateful	35	-1.8853222	0.4935855	-3.1945254	-0.5761190
Least	Views on nonorganization content that is hateful	36	-1.8016969	0.4027256	-2.8699002	-0.7334936
Least	Views on nonorganization content that is hateful	37	-2.0769044	0.2387597	-2.7101988	-1.4436099
Least	Views on nonorganization content that is hateful	38	-1.9526363	0.2956384	-2.7367978	-1.1684749
Least	Views on nonorganization content that is hateful	39	-1.9341857	0.3443722	-2.8476104	-1.0207611
Least	Views on nonorganization content that is hateful	40	-1.9026289	0.3643726	-2.8691035	-0.9361544
Least	Views on nonorganization content that is hateful	41	-1.9043893	0.4687280	-3.1476598	-0.6611189
Least	Views on nonorganization content that is hateful	42	-1.1710631	0.9109077	-3.5871862	1.2450599
Least	Views on nonorganization content that is hateful	43	-3.2773874	0.4878172	-4.5712908	-1.9834841
Least	Views on nonorganization content that is hateful	44	-3.0459772	0.4127658	-4.1408115	-1.9511430
Least	Views on nonorganization content that is hateful	45	-3.0505534	0.4486457	-4.2405567	-1.8605501
Least	Views on nonorganization content that is hateful	47	-2.6563247	0.3855049	-3.6788513	-1.6337982
Least	Views on nonorganization content that is hateful	48	-2.4721133	0.1274166	-2.8100774	-2.1341493
Least	Views on nonorganization content that is hateful	49	-2.3754836	0.3491094	-3.3014734	-1.4494938
Least	Views on nonorganization content that is hateful	50	-2.6236365	0.3344135	-3.5106465	-1.7366264
Least	Views on nonorganization content that is hateful	51	-2.6356718	0.3958637	-3.6856743	-1.5856694
Least	Views on nonorganization content that is hateful	52	-2.5824736	0.3354963	-3.4723557	-1.6925916
Least	Views on nonorganization content that is hateful	53	-2.6140503	0.3215157	-3.4668496	-1.7612511
Least	Views on nonorganization content that is hateful	54	-2.6576577	0.4786964	-3.9273685	-1.3879468
Least	Views on nonorganization content that is hateful	55	-2.5164623	0.4235551	-3.6399144	-1.3930101
Least	Views on nonorganization content that is hateful	56	-3.3681106	0.7799967	-5.4370009	-1.2992203
Least	Views on nonorganization content that is hateful	57	-3.0745589	0.4016030	-4.1397847	-2.0093332
Least	Views on nonorganization content that is hateful	58	-3.2200310	0.3802412	-4.2285958	-2.2114662
Least	Views on nonorganization content that is hateful	59	-2.9557518	0.3226675	-3.8116062	-2.0998974

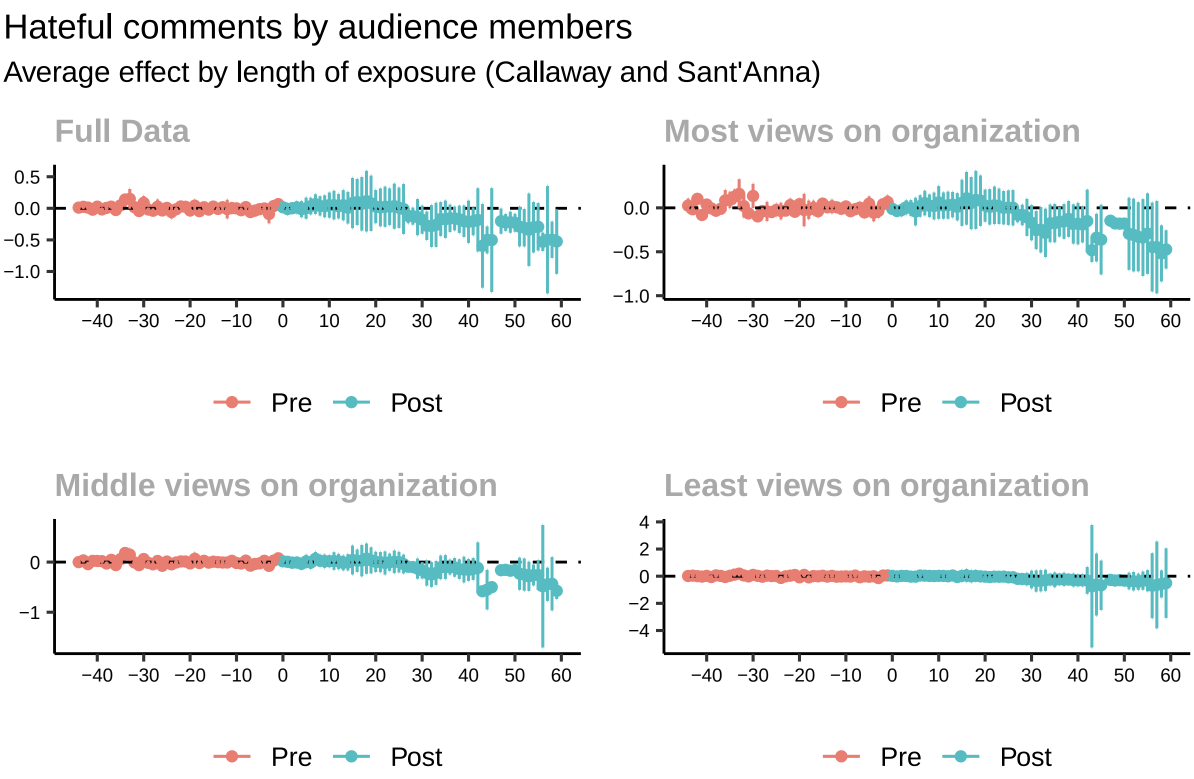

Long timeframe with sampled control groups

sample	outcome	event.time	estimate	std.error	conf.low	conf.high
Full	Hateful comments by audience members	-44	0.0107923	0.0199071	-0.0417581	0.0633426
Full	Hateful comments by audience members	-43	0.0234892	0.0105282	-0.0043028	0.0512813
Full	Hateful comments by audience members	-42	0.0113307	0.0272665	-0.0606471	0.0833085
Full	Hateful comments by audience members	-41	-0.0216093	0.0142449	-0.0592127	0.0159942
Full	Hateful comments by audience members	-40	0.0258166	0.0108401	-0.0027989	0.0544320
Full	Hateful comments by audience members	-39	-0.0174291	0.0063979	-0.0343181	-0.0005402
Full	Hateful comments by audience members	-38	0.0067941	0.0199865	-0.0459660	0.0595543
Full	Hateful comments by audience members	-37	0.0257411	0.0069464	0.0074042	0.0440780
Full	Hateful comments by audience members	-36	-0.0267375	0.0161411	-0.0693464	0.0158715
Full	Hateful comments by audience members	-35	0.0467707	0.0213962	-0.0097107	0.1032522
Full	Hateful comments by audience members	-34	0.1390296	0.0262619	0.0697038	0.2083554
Full	Hateful comments by audience members	-33	0.1493068	0.0591530	-0.0068442	0.3054579
Full	Hateful comments by audience members	-32	0.0183494	0.0252383	-0.0482741	0.0849730
Full	Hateful comments by audience members	-31	-0.0433660	0.0111039	-0.0726778	-0.0140542
Full	Hateful comments by audience members	-30	0.0862547	0.0409004	-0.0217135	0.1942229
Full	Hateful comments by audience members	-29	-0.0195284	0.0119690	-0.0511239	0.0120671
Full	Hateful comments by audience members	-28	-0.0379703	0.0148995	-0.0773019	0.0013612
Full	Hateful comments by audience members	-27	0.0252935	0.0433997	-0.0892722	0.1398593
Full	Hateful comments by audience members	-26	-0.0337272	0.0234808	-0.0957113	0.0282570

Full	Hateful comments by audience members	-25	0.0062279	0.0160075	-0.0360285	0.0484844
Full	Hateful comments by audience members	-24	-0.0593262	0.0388529	-0.1618895	0.0432371
Full	Hateful comments by audience members	-23	-0.0148077	0.0104805	-0.0424740	0.0128587
Full	Hateful comments by audience members	-22	0.0289788	0.0106778	0.0007916	0.0571660
Full	Hateful comments by audience members	-21	0.0240205	0.0123026	-0.0084556	0.0564966
Full	Hateful comments by audience members	-20	-0.0342794	0.0304859	-0.1147556	0.0461968
Full	Hateful comments by audience members	-19	0.0414865	0.0358440	-0.0531340	0.1361070
Full	Hateful comments by audience members	-18	-0.0450310	0.0165151	-0.0886272	-0.0014348
Full	Hateful comments by audience members	-17	0.0163982	0.0217626	-0.0410503	0.0738467
Full	Hateful comments by audience members	-16	-0.0204791	0.0255702	-0.0879790	0.0470208
Full	Hateful comments by audience members	-15	0.0262818	0.0126462	-0.0071015	0.0596652
Full	Hateful comments by audience members	-14	-0.0097267	0.0162759	-0.0526915	0.0332381
Full	Hateful comments by audience members	-13	0.0173445	0.0235329	-0.0447774	0.0794663
Full	Hateful comments by audience members	-12	-0.0129765	0.0557192	-0.1600631	0.1341102
Full	Hateful comments by audience members	-11	0.0040451	0.0307833	-0.0772162	0.0853063
Full	Hateful comments by audience members	-10	-0.0009871	0.0278726	-0.0745647	0.0725905
Full	Hateful comments by audience members	-9	-0.0301872	0.0166397	-0.0741125	0.0137381
Full	Hateful comments by audience members	-8	0.0190458	0.0249090	-0.0467086	0.0848002
Full	Hateful comments by audience members	-7	-0.0592462	0.0209873	-0.1146481	-0.0038442
Full	Hateful comments by audience members	-6	-0.0361012	0.0311141	-0.1182357	0.0460333
Full	Hateful comments by audience members	-5	-0.0134603	0.0169375	-0.0581715	0.0312510
Full	Hateful comments by audience members	-4	-0.0025236	0.0258004	-0.0706311	0.0655838
Full	Hateful comments by audience members	-3	-0.0900614	0.0553602	-0.2362005	0.0560776
Full	Hateful comments by audience members	-2	0.0354936	0.0220134	-0.0226171	0.0936042
Full	Hateful comments by audience members	-1	0.0661769	0.0134412	0.0306950	0.1016589
Full	Hateful comments by audience members	0	0.0162914	0.0251996	-0.0502301	0.0828129
Full	Hateful comments by audience members	1	-0.0106535	0.0340204	-0.1004599	0.0791530
Full	Hateful comments by audience members	2	0.0017372	0.0286852	-0.0739855	0.0774598
Full	Hateful comments by audience members	3	0.0143443	0.0370786	-0.0835353	0.1122238
Full	Hateful comments by audience members	4	-0.0136085	0.0478662	-0.1399650	0.1127479
Full	Hateful comments by audience members	5	0.0024861	0.0632129	-0.1643822	0.1693544
Full	Hateful comments by audience members	6	0.0343085	0.0502380	-0.0983089	0.1669258
Full	Hateful comments by audience members	7	0.0654678	0.0588420	-0.0898623	0.2207979
Full	Hateful comments by audience members	8	0.0375570	0.0578364	-0.1151185	0.1902325
Full	Hateful comments by audience members	9	0.0322091	0.0655851	-0.1409214	0.2053396
Full	Hateful comments by audience members	10	0.0504057	0.0753155	-0.1484111	0.2492224
Full	Hateful comments by audience members	11	0.0502084	0.0856187	-0.1758065	0.2762234
Full	Hateful comments by audience members	12	0.0328037	0.0734968	-0.1612120	0.2268195
Full	Hateful comments by audience members	13	0.0494769	0.0902976	-0.1888894	0.2878433
Full	Hateful comments by audience members	14	0.0088661	0.0947173	-0.2411672	0.2588994
Full	Hateful comments by audience members	15	0.0833789	0.1502567	-0.3132665	0.4800242

Full	Hateful comments by audience members	16	0.0998287	0.1380260	-0.2645302	0.4641876
Full	Hateful comments by audience members	17	0.0705463	0.1602287	-0.3524230	0.4935156
Full	Hateful comments by audience members	18	0.1128811	0.1816424	-0.3666158	0.5923779
Full	Hateful comments by audience members	19	0.0798956	0.1653868	-0.3566899	0.5164810
Full	Hateful comments by audience members	20	0.0272490	0.0991360	-0.2344488	0.2889468
Full	Hateful comments by audience members	21	0.0148190	0.1133256	-0.2843363	0.3139743
Full	Hateful comments by audience members	22	0.0246225	0.1204369	-0.2933051	0.3425500
Full	Hateful comments by audience members	23	0.0249335	0.1100105	-0.2654706	0.3153377
Full	Hateful comments by audience members	24	0.0331024	0.1348728	-0.3229328	0.3891375
Full	Hateful comments by audience members	25	0.0094066	0.1212039	-0.3105457	0.3293588
Full	Hateful comments by audience members	26	-0.0112037	0.1493094	-0.4053483	0.3829409
Full	Hateful comments by audience members	27	-0.1198417	0.0440153	-0.2360326	-0.0036507
Full	Hateful comments by audience members	28	-0.1238937	0.0508635	-0.2581624	0.0103751
Full	Hateful comments by audience members	29	-0.1393241	0.1081745	-0.4248815	0.1462333
Full	Hateful comments by audience members	30	-0.1801308	0.0838176	-0.4013911	0.0411295
Full	Hateful comments by audience members	31	-0.2752484	0.0851717	-0.5000832	-0.0504135
Full	Hateful comments by audience members	32	-0.2809500	0.1254654	-0.6121517	0.0502516
Full	Hateful comments by audience members	33	-0.2616922	0.1318727	-0.6098078	0.0864233
Full	Hateful comments by audience members	34	-0.1700056	0.1017325	-0.4385574	0.0985462
Full	Hateful comments by audience members	35	-0.1730453	0.1118454	-0.4682931	0.1222026
Full	Hateful comments by audience members	36	-0.1579059	0.0821135	-0.3746678	0.0588561
Full	Hateful comments by audience members	37	-0.1606739	0.0712056	-0.3486412	0.0272935
Full	Hateful comments by audience members	38	-0.1734523	0.0813608	-0.3882272	0.0413226
Full	Hateful comments by audience members	39	-0.2035068	0.0941167	-0.4519545	0.0449409
Full	Hateful comments by audience members	40	-0.2134549	0.1263964	-0.5471142	0.1202043
Full	Hateful comments by audience members	41	-0.2050517	0.0836439	-0.4258536	0.0157502
Full	Hateful comments by audience members	42	-0.1836797	0.1895377	-0.6840185	0.3166590
Full	Hateful comments by audience members	43	-0.5935582	0.2505702	-1.2550095	0.0678931
Full	Hateful comments by audience members	44	-0.5000005	0.0806940	-0.7130153	-0.2869857
Full	Hateful comments by audience members	45	-0.5023091	0.3105522	-1.3221000	0.3174818
Full	Hateful comments by audience members	47	-0.2027695	0.0751855	-0.4012430	-0.0042960
Full	Hateful comments by audience members	48	-0.2234378	0.0504649	-0.3566543	-0.0902213
Full	Hateful comments by audience members	49	-0.2204675	0.0628252	-0.3863125	-0.0546225
Full	Hateful comments by audience members	50	-0.2281463	0.0535596	-0.3695320	-0.0867606
Full	Hateful comments by audience members	51	-0.2902804	0.1182973	-0.6025598	0.0219991
Full	Hateful comments by audience members	52	-0.3073004	0.1121148	-0.6032595	-0.0113414
Full	Hateful comments by audience members	53	-0.3390438	0.2173228	-0.9127292	0.2346416
Full	Hateful comments by audience members	54	-0.3009670	0.1511025	-0.6998452	0.0979112
Full	Hateful comments by audience members	55	-0.2924927	0.1422043	-0.6678815	0.0828961
Full	Hateful comments by audience members	56	-0.5295451	0.0552206	-0.6753156	-0.3837745
Full	Hateful comments by audience members	57	-0.4972795	0.3212537	-1.3453202	0.3507612

Full	Hateful comments by audience members	58	-0.4970248	0.1089082	-0.7845189	-0.2095307
Full	Hateful comments by audience members	59	-0.5249279	0.1935813	-1.0359409	-0.0139149

sample	outcome	event.time	estimate	std.error	conf.low	conf.high
Most	Hateful comments by audience members	-44	0.0250883	0.0311055	-0.0562021	0.1063787
Most	Hateful comments by audience members	-43	-0.0160001	0.0120189	-0.0474101	0.0154099
Most	Hateful comments by audience members	-42	0.1026742	0.0196655	0.0512808	0.1540675
Most	Hateful comments by audience members	-41	-0.0808828	0.0086588	-0.1035114	-0.0582542
Most	Hateful comments by audience members	-40	0.0375092	0.0127986	0.0040616	0.0709568
Most	Hateful comments by audience members	-39	-0.0063729	0.0151514	-0.0459692	0.0332234
Most	Hateful comments by audience members	-38	-0.0325968	0.0207708	-0.0868787	0.0216851
Most	Hateful comments by audience members	-37	-0.0113633	0.0118318	-0.0422842	0.0195575
Most	Hateful comments by audience members	-36	0.0844535	0.0455979	-0.0347109	0.2036180
Most	Hateful comments by audience members	-35	0.0903191	0.0356882	-0.0029473	0.1835856
Most	Hateful comments by audience members	-34	0.1278353	0.0311964	0.0463074	0.2093633
Most	Hateful comments by audience members	-33	0.1586207	0.0636189	-0.0076391	0.3248805
Most	Hateful comments by audience members	-32	0.0199806	0.0488773	-0.1077541	0.1477152
Most	Hateful comments by audience members	-31	-0.0622387	0.0234966	-0.1236440	-0.0008333
Most	Hateful comments by audience members	-30	0.1356279	0.0524565	-0.0014606	0.2727164
Most	Hateful comments by audience members	-29	-0.0968641	0.0169266	-0.1410997	-0.0526284
Most	Hateful comments by audience members	-28	-0.0362648	0.0148163	-0.0749855	0.0024558
Most	Hateful comments by audience members	-27	-0.0406273	0.0416349	-0.1494349	0.0681803
Most	Hateful comments by audience members	-26	-0.0478820	0.0161417	-0.0900663	-0.0056977
Most	Hateful comments by audience members	-25	-0.0238690	0.0268618	-0.0940688	0.0463308
Most	Hateful comments by audience members	-24	-0.0361553	0.0359309	-0.1300562	0.0577457
Most	Hateful comments by audience members	-23	-0.0317922	0.0143582	-0.0693154	0.0057311
Most	Hateful comments by audience members	-22	0.0309910	0.0283967	-0.0432201	0.1052021
Most	Hateful comments by audience members	-21	-0.0443101	0.0205701	-0.0980675	0.0094473
Most	Hateful comments by audience members	-20	0.0425109	0.0230041	-0.0176073	0.1026291
Most	Hateful comments by audience members	-19	-0.0245461	0.0703882	-0.2084967	0.1594046
Most	Hateful comments by audience members	-18	-0.0218051	0.0404864	-0.1276113	0.0840010
Most	Hateful comments by audience members	-17	0.0000094	0.0311569	-0.0814150	0.0814339
Most	Hateful comments by audience members	-16	-0.0387840	0.0256121	-0.1057179	0.0281499
Most	Hateful comments by audience members	-15	0.0477632	0.0220058	-0.0097461	0.1052725
Most	Hateful comments by audience members	-14	0.0059000	0.0128923	-0.0277923	0.0395923
Most	Hateful comments by audience members	-13	0.0151205	0.0298434	-0.0628713	0.0931123
Most	Hateful comments by audience members	-12	0.0067122	0.0176212	-0.0393386	0.0527630
Most	Hateful comments by audience members	-11	-0.0107359	0.0221589	-0.0686454	0.0471736
Most	Hateful comments by audience members	-10	0.0156810	0.0216536	-0.0409078	0.0722698
Most	Hateful comments by audience members	-9	-0.0377002	0.0215845	-0.0941087	0.0187082
Most	Hateful comments by audience members	-8	-0.0149224	0.0219843	-0.0723754	0.0425307

Most	Hateful comments by audience members	-7	0.0021329	0.0240005	-0.0605895	0.0648552
Most	Hateful comments by audience members	-6	-0.0520261	0.0224514	-0.1107000	0.0066478
Most	Hateful comments by audience members	-5	0.0474771	0.0316549	-0.0352491	0.1302032
Most	Hateful comments by audience members	-4	-0.0554731	0.0371439	-0.1525440	0.0415978
Most	Hateful comments by audience members	-3	-0.0358980	0.0299421	-0.1141479	0.0423519
Most	Hateful comments by audience members	-2	0.0410507	0.0199111	-0.0109846	0.0930859
Most	Hateful comments by audience members	-1	0.0663288	0.0288965	-0.0091886	0.1418463
Most	Hateful comments by audience members	0	-0.0114617	0.0136782	-0.0472080	0.0242845
Most	Hateful comments by audience members	1	-0.0356416	0.0220815	-0.0933488	0.0220656
Most	Hateful comments by audience members	2	-0.0255516	0.0273958	-0.0971471	0.0460439
Most	Hateful comments by audience members	3	0.0062244	0.0304934	-0.0734664	0.0859151
Most	Hateful comments by audience members	4	-0.0017493	0.0347117	-0.0924640	0.0889655
Most	Hateful comments by audience members	5	-0.0420040	0.0599796	-0.1987532	0.1147451
Most	Hateful comments by audience members	6	0.0214053	0.0466675	-0.1005543	0.1433648
Most	Hateful comments by audience members	7	0.0546723	0.0533612	-0.0847804	0.1941251
Most	Hateful comments by audience members	8	0.0239478	0.0493155	-0.1049320	0.1528277
Most	Hateful comments by audience members	9	0.0211940	0.0588327	-0.1325578	0.1749458
Most	Hateful comments by audience members	10	0.0617594	0.0710976	-0.1240451	0.2475639
Most	Hateful comments by audience members	11	0.0260549	0.0570939	-0.1231529	0.1752627
Most	Hateful comments by audience members	12	0.0306149	0.0588841	-0.1232713	0.1845011
Most	Hateful comments by audience members	13	0.0363679	0.0552537	-0.1080307	0.1807666
Most	Hateful comments by audience members	14	0.0119134	0.0594684	-0.1434998	0.1673266
Most	Hateful comments by audience members	15	0.0573137	0.1003097	-0.2048330	0.3194603
Most	Hateful comments by audience members	16	0.1083429	0.1145877	-0.1911176	0.4078034
Most	Hateful comments by audience members	17	0.0542516	0.1134935	-0.2423492	0.3508523
Most	Hateful comments by audience members	18	0.0909360	0.1262687	-0.2390513	0.4209233
Most	Hateful comments by audience members	19	0.0820625	0.1096730	-0.2045540	0.3686791
Most	Hateful comments by audience members	20	0.0254264	0.0700255	-0.1575764	0.2084293
Most	Hateful comments by audience members	21	0.0108856	0.0771077	-0.1906256	0.2123967
Most	Hateful comments by audience members	22	0.0292323	0.0816630	-0.1841835	0.2426481
Most	Hateful comments by audience members	23	0.0184579	0.0768072	-0.1822680	0.2191838
Most	Hateful comments by audience members	24	0.0010986	0.0726286	-0.1887071	0.1909042
Most	Hateful comments by audience members	25	0.0023952	0.0750659	-0.1937801	0.1985704
Most	Hateful comments by audience members	26	0.0012311	0.0769876	-0.1999663	0.2024285
Most	Hateful comments by audience members	27	-0.0785030	0.0324234	-0.1632373	0.0062313
Most	Hateful comments by audience members	28	-0.0811228	0.0445276	-0.1974900	0.0352444
Most	Hateful comments by audience members	29	-0.1086834	0.0805853	-0.3192828	0.1019160
Most	Hateful comments by audience members	30	-0.1692593	0.0764796	-0.3691290	0.0306104
Most	Hateful comments by audience members	31	-0.2529974	0.0828850	-0.4696069	-0.0363880
Most	Hateful comments by audience members	32	-0.2495483	0.0991767	-0.5087340	0.0096374
Most	Hateful comments by audience members	33	-0.2848869	0.1050232	-0.5593519	-0.0104220

Most	Hateful comments by audience members	34	-0.1760091	0.0821271	-0.3906380	0.0386197
Most	Hateful comments by audience members	35	-0.1735954	0.0834298	-0.3916287	0.0444379
Most	Hateful comments by audience members	36	-0.1587795	0.0644653	-0.3272514	0.0096925
Most	Hateful comments by audience members	37	-0.1535991	0.0756806	-0.3513809	0.0441826
Most	Hateful comments by audience members	38	-0.1260160	0.0779347	-0.3296884	0.0776563
Most	Hateful comments by audience members	39	-0.1837495	0.0854100	-0.4069577	0.0394587
Most	Hateful comments by audience members	40	-0.1796334	0.0902324	-0.4154444	0.0561776
Most	Hateful comments by audience members	41	-0.1899177	0.0779923	-0.3937406	0.0139052
Most	Hateful comments by audience members	42	-0.1503102	0.1364104	-0.5068014	0.2061811
Most	Hateful comments by audience members	43	-0.4822639	0.0511136	-0.6158429	-0.3486850
Most	Hateful comments by audience members	44	-0.3397769	0.1036635	-0.6106883	-0.0688654
Most	Hateful comments by audience members	45	-0.3617728	0.1514628	-0.7576016	0.0340560
Most	Hateful comments by audience members	47	-0.1463128	0.0192206	-0.1965434	-0.0960821
Most	Hateful comments by audience members	48	-0.1774239	0.0129010	-0.2111390	-0.1437088
Most	Hateful comments by audience members	49	-0.1796461	0.0152438	-0.2194839	-0.1398083
Most	Hateful comments by audience members	50	-0.1790365	0.0136921	-0.2148191	-0.1432539
Most	Hateful comments by audience members	51	-0.2953958	0.1568385	-0.7052734	0.1144817
Most	Hateful comments by audience members	52	-0.3095335	0.1581378	-0.7228065	0.1037396
Most	Hateful comments by audience members	53	-0.3290258	0.1502351	-0.7216461	0.0635946
Most	Hateful comments by audience members	54	-0.3381550	0.1670494	-0.7747174	0.0984074
Most	Hateful comments by audience members	55	-0.2930688	0.1751692	-0.7508512	0.1647136
Most	Hateful comments by audience members	56	-0.4459945	0.1937411	-0.9523122	0.0603232
Most	Hateful comments by audience members	57	-0.4470154	0.2011543	-0.9727067	0.0786758
Most	Hateful comments by audience members	58	-0.5185831	0.1220185	-0.8374629	-0.1997032
Most	Hateful comments by audience members	59	-0.4732463	0.0833358	-0.6910338	-0.2554588

sample	outcome	event.time	estimate	std.error	conf.low	conf.high
Middle	Hateful comments by audience members	-44	-0.0001807	0.0147256	-0.0372710	0.0369096
Middle	Hateful comments by audience members	-43	0.0330385	0.0159566	-0.0071524	0.0732295
Middle	Hateful comments by audience members	-42	-0.0409649	0.0192547	-0.0894629	0.0075332
Middle	Hateful comments by audience members	-41	0.0237969	0.0105481	-0.0027713	0.0503651
Middle	Hateful comments by audience members	-40	0.0218640	0.0135716	-0.0123195	0.0560475
Middle	Hateful comments by audience members	-39	0.0185350	0.0085804	-0.0030769	0.0401469
Middle	Hateful comments by audience members	-38	-0.0284368	0.0095141	-0.0524005	-0.0044731
Middle	Hateful comments by audience members	-37	0.0446024	0.0061400	0.0291371	0.0600676
Middle	Hateful comments by audience members	-36	-0.0597088	0.0081739	-0.0802969	-0.0391207
Middle	Hateful comments by audience members	-35	0.0591149	0.0346912	-0.0282639	0.1464938
Middle	Hateful comments by audience members	-34	0.1812253	0.0228600	0.1236463	0.2388042
Middle	Hateful comments by audience members	-33	0.1483295	0.0485509	0.0260413	0.2706176
Middle	Hateful comments by audience members	-32	-0.0065431	0.0428759	-0.1145372	0.1014510
Middle	Hateful comments by audience members	-31	-0.0599881	0.0094997	-0.0839157	-0.0360606

Middle	Hateful comments by audience members	-30	0.0606312	0.0299170	-0.0147225	0.1359849
Middle	Hateful comments by audience members	-29	-0.0155152	0.0137903	-0.0502498	0.0192194
Middle	Hateful comments by audience members	-28	-0.0445731	0.0299546	-0.1200214	0.0308753
Middle	Hateful comments by audience members	-27	0.0213776	0.0169288	-0.0212620	0.0640171
Middle	Hateful comments by audience members	-26	-0.0733735	0.0366815	-0.1657655	0.0190184
Middle	Hateful comments by audience members	-25	0.0106646	0.0124518	-0.0206985	0.0420277
Middle	Hateful comments by audience members	-24	-0.0445655	0.0203882	-0.0959184	0.0067874
Middle	Hateful comments by audience members	-23	-0.0078273	0.0084429	-0.0290930	0.0134384
Middle	Hateful comments by audience members	-22	0.0129282	0.0109864	-0.0147439	0.0406004
Middle	Hateful comments by audience members	-21	0.0112550	0.0203586	-0.0400234	0.0625333
Middle	Hateful comments by audience members	-20	-0.0255289	0.0189979	-0.0733801	0.0223223
Middle	Hateful comments by audience members	-19	0.0652590	0.0485837	-0.0571117	0.1876296
Middle	Hateful comments by audience members	-18	-0.0184540	0.0294019	-0.0925103	0.0556023
Middle	Hateful comments by audience members	-17	0.0247499	0.0183616	-0.0214986	0.0709983
Middle	Hateful comments by audience members	-16	-0.0101177	0.0189738	-0.0579082	0.0376728
Middle	Hateful comments by audience members	-15	0.0078634	0.0260844	-0.0578370	0.0735638
Middle	Hateful comments by audience members	-14	-0.0011105	0.0115002	-0.0300768	0.0278557
Middle	Hateful comments by audience members	-13	-0.0083598	0.0197614	-0.0581340	0.0414145
Middle	Hateful comments by audience members	-12	-0.0084054	0.0362942	-0.0998217	0.0830110
Middle	Hateful comments by audience members	-11	0.0249789	0.0191362	-0.0232207	0.0731784
Middle	Hateful comments by audience members	-10	-0.0181414	0.0185900	-0.0649652	0.0286823
Middle	Hateful comments by audience members	-9	-0.0283298	0.0112661	-0.0567063	0.0000467
Middle	Hateful comments by audience members	-8	0.0286497	0.0177550	-0.0160708	0.0733702
Middle	Hateful comments by audience members	-7	-0.0709128	0.0233475	-0.1297195	-0.0121061
Middle	Hateful comments by audience members	-6	-0.0373925	0.0178751	-0.0824155	0.0076305
Middle	Hateful comments by audience members	-5	-0.0240664	0.0217379	-0.0788190	0.0306862
Middle	Hateful comments by audience members	-4	0.0252766	0.0222332	-0.0307235	0.0812766
Middle	Hateful comments by audience members	-3	-0.0749457	0.0297339	-0.1498382	-0.0000532
Middle	Hateful comments by audience members	-2	0.0284187	0.0178792	-0.0166148	0.0734522
Middle	Hateful comments by audience members	-1	0.0751852	0.0305704	-0.0018144	0.1521847
Middle	Hateful comments by audience members	0	0.0150908	0.0153391	-0.0235447	0.0537263
Middle	Hateful comments by audience members	1	0.0056819	0.0206121	-0.0462349	0.0575987
Middle	Hateful comments by audience members	2	-0.0122609	0.0419128	-0.1178290	0.0933073
Middle	Hateful comments by audience members	3	-0.0083142	0.0393309	-0.1073793	0.0907509
Middle	Hateful comments by audience members	4	-0.0345676	0.0435983	-0.1443814	0.0752461
Middle	Hateful comments by audience members	5	0.0140266	0.0519962	-0.1169394	0.1449926
Middle	Hateful comments by audience members	6	-0.0089321	0.0511400	-0.1377414	0.1198772
Middle	Hateful comments by audience members	7	0.0667489	0.0535127	-0.0680367	0.2015345
Middle	Hateful comments by audience members	8	0.0263769	0.0474819	-0.0932187	0.1459725
Middle	Hateful comments by audience members	9	0.0178979	0.0525569	-0.1144802	0.1502761
Middle	Hateful comments by audience members	10	0.0182327	0.0476506	-0.1017878	0.1382532

Middle	Hateful comments by audience members	11	0.0213746	0.0694744	-0.1536147	0.1963639
Middle	Hateful comments by audience members	12	0.0115245	0.0602880	-0.1403266	0.1633755
Middle	Hateful comments by audience members	13	-0.0156960	0.0585153	-0.1630819	0.1316899
Middle	Hateful comments by audience members	14	-0.0049195	0.0644540	-0.1672637	0.1574247
Middle	Hateful comments by audience members	15	0.0412233	0.1140167	-0.2459573	0.3284039
Middle	Hateful comments by audience members	16	0.0272209	0.0891758	-0.1973916	0.2518334
Middle	Hateful comments by audience members	17	0.0289073	0.1237899	-0.2828897	0.3407043
Middle	Hateful comments by audience members	18	0.0697929	0.1195362	-0.2312902	0.3708759
Middle	Hateful comments by audience members	19	0.0349560	0.1026048	-0.2234808	0.2933927
Middle	Hateful comments by audience members	20	0.0044446	0.0795115	-0.1958257	0.2047149
Middle	Hateful comments by audience members	21	-0.0007059	0.0792690	-0.2003655	0.1989536
Middle	Hateful comments by audience members	22	-0.0176232	0.0915488	-0.2482125	0.2129661
Middle	Hateful comments by audience members	23	-0.0031363	0.0680686	-0.1745849	0.1683122
Middle	Hateful comments by audience members	24	0.0061132	0.0895336	-0.2194005	0.2316269
Middle	Hateful comments by audience members	25	-0.0007297	0.0812660	-0.2054192	0.2039598
Middle	Hateful comments by audience members	26	-0.0389385	0.0740169	-0.2253692	0.1474922
Middle	Hateful comments by audience members	27	-0.0926731	0.0353098	-0.1816100	-0.0037363
Middle	Hateful comments by audience members	28	-0.0997090	0.0330351	-0.1829166	-0.0165015
Middle	Hateful comments by audience members	29	-0.1296378	0.0796306	-0.3302080	0.0709324
Middle	Hateful comments by audience members	30	-0.1640863	0.0652958	-0.3285508	0.0003781
Middle	Hateful comments by audience members	31	-0.2167509	0.1001962	-0.4691210	0.0356191
Middle	Hateful comments by audience members	32	-0.2475048	0.0983791	-0.4952980	0.0002884
Middle	Hateful comments by audience members	33	-0.2169685	0.0940374	-0.4538262	0.0198892
Middle	Hateful comments by audience members	34	-0.1046012	0.0933632	-0.3397608	0.1305583
Middle	Hateful comments by audience members	35	-0.0991953	0.0943010	-0.3367169	0.1383263
Middle	Hateful comments by audience members	36	-0.0991905	0.0597881	-0.2497823	0.0514014
Middle	Hateful comments by audience members	37	-0.1054094	0.0736432	-0.2908988	0.0800801
Middle	Hateful comments by audience members	38	-0.1405509	0.0774711	-0.3356819	0.0545802
Middle	Hateful comments by audience members	39	-0.1503078	0.1016883	-0.4064363	0.1058207
Middle	Hateful comments by audience members	40	-0.1555736	0.0862686	-0.3728636	0.0617163
Middle	Hateful comments by audience members	41	-0.1362152	0.0863629	-0.3537426	0.0813121
Middle	Hateful comments by audience members	42	-0.1169624	0.2024877	-0.6269803	0.3930554
Middle	Hateful comments by audience members	43	-0.5816612	0.0283589	-0.6530905	-0.5102319
Middle	Hateful comments by audience members	44	-0.5499780	0.1562455	-0.9435230	-0.1564330
Middle	Hateful comments by audience members	45	-0.4998377	0.0205498	-0.5515976	-0.4480778
Middle	Hateful comments by audience members	47	-0.1617567	0.0342386	-0.2479956	-0.0755178
Middle	Hateful comments by audience members	48	-0.1549347	0.0224787	-0.2115532	-0.0983161
Middle	Hateful comments by audience members	49	-0.1719898	0.0264632	-0.2386444	-0.1053353
Middle	Hateful comments by audience members	50	-0.1589333	0.0238472	-0.2189988	-0.0988678
Middle	Hateful comments by audience members	51	-0.2328927	0.1266573	-0.5519119	0.0861266
Middle	Hateful comments by audience members	52	-0.2487937	0.1272806	-0.5693830	0.0717956

Middle	Hateful comments by audience members	53	-0.2835277	0.1150222	-0.5732409	0.0061855
Middle	Hateful comments by audience members	54	-0.2581319	0.0824225	-0.4657344	-0.0505294
Middle	Hateful comments by audience members	55	-0.2590417	0.1184830	-0.5574719	0.0393886
Middle	Hateful comments by audience members	56	-0.4826513	0.4846808	-1.7034457	0.7381432
Middle	Hateful comments by audience members	57	-0.4427806	0.1308736	-0.7724196	-0.1131415
Middle	Hateful comments by audience members	58	-0.4324367	0.2109955	-0.9638838	0.0990103
Middle	Hateful comments by audience members	59	-0.5730308	0.0645516	-0.7356208	-0.4104408

sample	outcome	event.time	estimate	std.error	conf.low	conf.high
Least	Hateful comments by audience members	-44	0.0146029	0.0249282	-0.0480020	0.0772079
Least	Hateful comments by audience members	-43	0.0409653	0.0283331	-0.0301907	0.1121212
Least	Hateful comments by audience members	-42	0.0034144	0.0503574	-0.1230538	0.1298825
Least	Hateful comments by audience members	-41	-0.0274954	0.0417911	-0.1324500	0.0774592
Least	Hateful comments by audience members	-40	0.0238214	0.0334402	-0.0601608	0.1078035
Least	Hateful comments by audience members	-39	-0.0611489	0.0168030	-0.1033481	-0.0189496
Least	Hateful comments by audience members	-38	0.0606789	0.0540075	-0.0749562	0.1963139
Least	Hateful comments by audience members	-37	0.0257126	0.0136385	-0.0085392	0.0599644
Least	Hateful comments by audience members	-36	-0.0602690	0.0275034	-0.1293414	0.0088034
Least	Hateful comments by audience members	-35	0.0310871	0.0403448	-0.0702352	0.1324094
Least	Hateful comments by audience members	-34	0.1161614	0.0498924	-0.0091390	0.2414618
Least	Hateful comments by audience members	-33	0.1872824	0.0819560	-0.0185427	0.3931076
Least	Hateful comments by audience members	-32	0.0571887	0.0542591	-0.0790783	0.1934557
Least	Hateful comments by audience members	-31	-0.0132882	0.0215147	-0.0673204	0.0407441
Least	Hateful comments by audience members	-30	0.0957489	0.0701157	-0.0803406	0.2718384
Least	Hateful comments by audience members	-29	0.0259938	0.0277199	-0.0436222	0.0956098
Least	Hateful comments by audience members	-28	-0.0383603	0.0382242	-0.1343570	0.0576364
Least	Hateful comments by audience members	-27	0.0428855	0.0902410	-0.1837468	0.2695177
Least	Hateful comments by audience members	-26	0.0071172	0.0224991	-0.0493873	0.0636218
Least	Hateful comments by audience members	-25	0.0165187	0.0301868	-0.0592928	0.0923302
Least	Hateful comments by audience members	-24	-0.1255719	0.0372098	-0.2190210	-0.0321227
Least	Hateful comments by audience members	-23	-0.0080872	0.0357094	-0.0977683	0.0815939
Least	Hateful comments by audience members	-22	0.0388190	0.0332650	-0.0447231	0.1223612
Least	Hateful comments by audience members	-21	0.0901986	0.0594991	-0.0592282	0.2396253
Least	Hateful comments by audience members	-20	-0.0815107	0.0634082	-0.2407549	0.0777335
Least	Hateful comments by audience members	-19	0.0936553	0.0297737	0.0188813	0.1684294
Least	Hateful comments by audience members	-18	-0.0855986	0.0337384	-0.1703295	-0.0008677
Least	Hateful comments by audience members	-17	0.0096125	0.0294211	-0.0642761	0.0835011
Least	Hateful comments by audience members	-16	-0.0091719	0.0341515	-0.0949403	0.0765965
Least	Hateful comments by audience members	-15	0.0339423	0.0211908	-0.0192765	0.0871610
Least	Hateful comments by audience members	-14	-0.0300622	0.0300359	-0.1054947	0.0453702
Least	Hateful comments by audience members	-13	0.0277895	0.0471949	-0.0907364	0.1463154

Least	Hateful comments by audience members	-12	-0.0362631	0.0253172	-0.0998450	0.0273187
Least	Hateful comments by audience members	-11	-0.0233620	0.0255195	-0.0874520	0.0407281
Least	Hateful comments by audience members	-10	-0.0124127	0.0367429	-0.1046892	0.0798638
Least	Hateful comments by audience members	-9	-0.0271065	0.0227943	-0.0843524	0.0301394
Least	Hateful comments by audience members	-8	0.0448944	0.0266805	-0.0221115	0.1119002
Least	Hateful comments by audience members	-7	-0.0784656	0.0412420	-0.1820413	0.0251102
Least	Hateful comments by audience members	-6	-0.0090592	0.0442221	-0.1201192	0.1020007
Least	Hateful comments by audience members	-5	-0.0378847	0.0250385	-0.1007666	0.0249972
Least	Hateful comments by audience members	-4	-0.0066742	0.0315166	-0.0858255	0.0724770
Least	Hateful comments by audience members	-3	-0.1356724	0.0584558	-0.2824790	0.0111343
Least	Hateful comments by audience members	-2	0.0458654	0.0486973	-0.0764336	0.1681644
Least	Hateful comments by audience members	-1	0.0539775	0.0336661	-0.0305719	0.1385269
Least	Hateful comments by audience members	0	0.0246166	0.0706082	-0.1527097	0.2019429
Least	Hateful comments by audience members	1	-0.0162422	0.0319375	-0.0964505	0.0639660
Least	Hateful comments by audience members	2	0.0213235	0.0545681	-0.1157195	0.1583665
Least	Hateful comments by audience members	3	0.0168240	0.0702632	-0.1596359	0.1932839
Least	Hateful comments by audience members	4	-0.0327274	0.0605483	-0.1847891	0.1193343
Least	Hateful comments by audience members	5	-0.0342468	0.0620110	-0.1899820	0.1214885
Least	Hateful comments by audience members	6	0.0526516	0.0811664	-0.1511907	0.2564938
Least	Hateful comments by audience members	7	0.0368708	0.0986283	-0.2108254	0.2845671
Least	Hateful comments by audience members	8	0.0186113	0.0741933	-0.1677185	0.2049412
Least	Hateful comments by audience members	9	0.0097930	0.1171873	-0.2845126	0.3040987
Least	Hateful comments by audience members	10	0.0226939	0.1172625	-0.2718005	0.3171882
Least	Hateful comments by audience members	11	0.0219906	0.0819427	-0.1838013	0.2277824
Least	Hateful comments by audience members	12	-0.0049196	0.1112803	-0.2843902	0.2745510
Least	Hateful comments by audience members	13	0.0499827	0.0988566	-0.1982869	0.2982524
Least	Hateful comments by audience members	14	-0.0585465	0.0931247	-0.2924210	0.1753279
Least	Hateful comments by audience members	15	0.0232744	0.1711814	-0.4066327	0.4531814
Least	Hateful comments by audience members	16	0.0536859	0.1872023	-0.4164561	0.5238279
Least	Hateful comments by audience members	17	-0.0058981	0.1754311	-0.4464779	0.4346816
Least	Hateful comments by audience members	18	0.0247562	0.1626061	-0.3836146	0.4331271
Least	Hateful comments by audience members	19	-0.0110728	0.1484535	-0.3839008	0.3617551
Least	Hateful comments by audience members	20	-0.0330608	0.1135000	-0.3181061	0.2519844
Least	Hateful comments by audience members	21	-0.0609651	0.1066035	-0.3286903	0.2067601
Least	Hateful comments by audience members	22	-0.0314759	0.1215706	-0.3367898	0.2738380
Least	Hateful comments by audience members	23	-0.0415586	0.1135122	-0.3266346	0.2435174
Least	Hateful comments by audience members	24	-0.0299897	0.1249896	-0.3438901	0.2839106
Least	Hateful comments by audience members	25	-0.0778398	0.1462784	-0.4452050	0.2895254
Least	Hateful comments by audience members	26	-0.0763170	0.1279645	-0.3976886	0.2450546
Least	Hateful comments by audience members	27	-0.1956232	0.0931600	-0.4295863	0.0383398
Least	Hateful comments by audience members	28	-0.2041519	0.1026114	-0.4618514	0.0535476

Least	Hateful comments by audience members	29	-0.2195950	0.1847227	-0.6835096	0.2443197
Least	Hateful comments by audience members	30	-0.2449919	0.2569857	-0.8903887	0.4004049
Least	Hateful comments by audience members	31	-0.3559552	0.3106833	-1.1362089	0.4242985
Least	Hateful comments by audience members	32	-0.3373438	0.3102749	-1.1165718	0.4418843
Least	Hateful comments by audience members	33	-0.3013327	0.3068768	-1.0720266	0.4693612
Least	Hateful comments by audience members	34	-0.2483537	0.1690121	-0.6728128	0.1761054
Least	Hateful comments by audience members	35	-0.2654810	0.2169692	-0.8103800	0.2794181
Least	Hateful comments by audience members	36	-0.2311201	0.1596474	-0.6320604	0.1698202
Least	Hateful comments by audience members	37	-0.2346192	0.1921551	-0.7171999	0.2479615
Least	Hateful comments by audience members	38	-0.2450296	0.1517717	-0.6261908	0.1361317
Least	Hateful comments by audience members	39	-0.2829418	0.1910731	-0.7628051	0.1969214
Least	Hateful comments by audience members	40	-0.3077383	0.1679075	-0.7294231	0.1139465
Least	Hateful comments by audience members	41	-0.3011866	0.1787304	-0.7500521	0.1476789
Least	Hateful comments by audience members	42	-0.3084270	0.3917224	-1.2922033	0.6753492
Least	Hateful comments by audience members	43	-0.7430775	1.7954937	-5.2523014	3.7661463
Least	Hateful comments by audience members	44	-0.6117798	0.9091085	-2.8949256	1.6713660
Least	Hateful comments by audience members	45	-0.6717597	0.7234499	-2.4886404	1.1451210
Least	Hateful comments by audience members	47	-0.2714182	0.0300703	-0.3469369	-0.1958994
Least	Hateful comments by audience members	48	-0.3178067	0.0193064	-0.3662930	-0.2693204
Least	Hateful comments by audience members	49	-0.2907208	0.0240351	-0.3510827	-0.2303588
Least	Hateful comments by audience members	50	-0.3245939	0.0204818	-0.3760320	-0.2731557
Least	Hateful comments by audience members	51	-0.3546705	0.2423370	-0.9632784	0.2539374
Least	Hateful comments by audience members	52	-0.3745826	0.2713272	-1.0559968	0.3068316
Least	Hateful comments by audience members	53	-0.4095468	0.2376176	-1.0063025	0.1872089
Least	Hateful comments by audience members	54	-0.3307174	0.2669276	-1.0010825	0.3396477
Least	Hateful comments by audience members	55	-0.3313455	0.3099197	-1.1096816	0.4469906
Least	Hateful comments by audience members	56	-0.7003294	0.9516695	-3.0903634	1.6897046
Least	Hateful comments by audience members	57	-0.6405585	1.2726655	-3.8367456	2.5556286
Least	Hateful comments by audience members	58	-0.5712093	0.3977025	-1.5700040	0.4275853
Least	Hateful comments by audience members	59	-0.5116673	1.0194407	-3.0719026	2.0485679

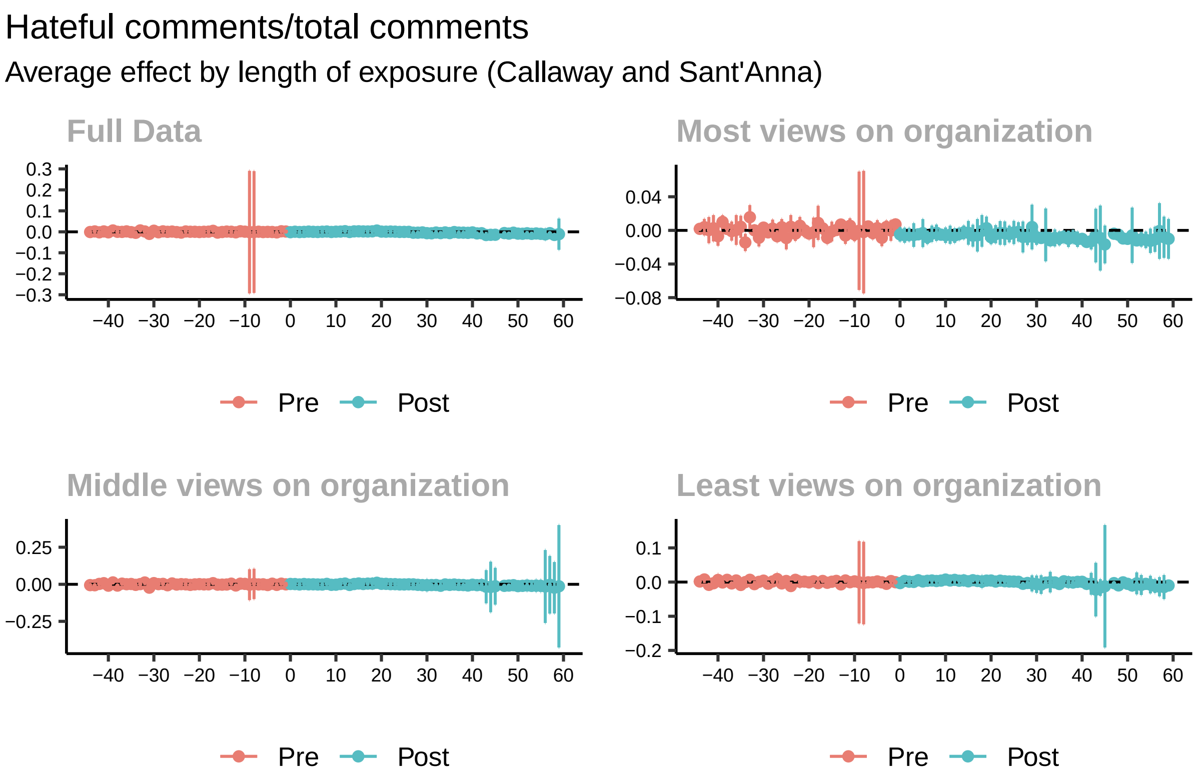

Long timeframe with sampled control groups

sample	outcome	event.time	estimate	std.error	conf.low	conf.high
Full	Hateful comments/total comments	-44	-0.0009911	0.0013172	-0.0043239	0.0023418
Full	Hateful comments/total comments	-43	0.0017685	0.0011435	-0.0011248	0.0046618
Full	Hateful comments/total comments	-42	-0.0023581	0.0021639	-0.0078333	0.0031171
Full	Hateful comments/total comments	-41	0.0018433	0.0025980	-0.0047304	0.0084170
Full	Hateful comments/total comments	-40	-0.0024293	0.0047824	-0.0145303	0.0096716
Full	Hateful comments/total comments	-39	0.0064123	0.0035782	-0.0026416	0.0154663
Full	Hateful comments/total comments	-38	-0.0000009	0.0009310	-0.0023567	0.0023548
Full	Hateful comments/total comments	-37	-0.0003392	0.0030230	-0.0079882	0.0073098
Full	Hateful comments/total comments	-36	0.0021237	0.0014839	-0.0016311	0.0058784
Full	Hateful comments/total comments	-35	-0.0015847	0.0019727	-0.0065762	0.0034068
Full	Hateful comments/total comments	-34	-0.0047118	0.0037188	-0.0141212	0.0046977
Full	Hateful comments/total comments	-33	0.0066978	0.0028477	-0.0005077	0.0139032
Full	Hateful comments/total comments	-32	0.0016662	0.0019677	-0.0033127	0.0066450
Full	Hateful comments/total comments	-31	-0.0098742	0.0029812	-0.0174174	-0.0023310
Full	Hateful comments/total comments	-30	0.0051843	0.0029125	-0.0021851	0.0125538
Full	Hateful comments/total comments	-29	-0.0020647	0.0013028	-0.0053611	0.0012318
Full	Hateful comments/total comments	-28	0.0021444	0.0010179	-0.0004312	0.0047201
Full	Hateful comments/total comments	-27	-0.0000453	0.0026182	-0.0066700	0.0065793
Full	Hateful comments/total comments	-26	0.0011844	0.0021627	-0.0042878	0.0066567

Full	Hateful comments/total comments	-25	-0.0014192	0.0020127	-0.0065120	0.0036736
Full	Hateful comments/total comments	-24	-0.0035958	0.0009522	-0.0060051	-0.0011865
Full	Hateful comments/total comments	-23	0.0016587	0.0024008	-0.0044160	0.0077335
Full	Hateful comments/total comments	-22	0.0003503	0.0033941	-0.0082378	0.0089384
Full	Hateful comments/total comments	-21	0.0002220	0.0029376	-0.0072108	0.0076549
Full	Hateful comments/total comments	-20	-0.0009215	0.0022830	-0.0066981	0.0048550
Full	Hateful comments/total comments	-19	0.0001890	0.0014258	-0.0034187	0.0037966
Full	Hateful comments/total comments	-18	0.0007121	0.0019595	-0.0042461	0.0056703
Full	Hateful comments/total comments	-17	0.0046215	0.0023139	-0.0012332	0.0104763
Full	Hateful comments/total comments	-16	-0.0036867	0.0021987	-0.0092499	0.0018765
Full	Hateful comments/total comments	-15	-0.0008870	0.0019937	-0.0059317	0.0041578
Full	Hateful comments/total comments	-14	0.0011388	0.0013595	-0.0023011	0.0045788
Full	Hateful comments/total comments	-13	0.0003100	0.0012252	-0.0027900	0.0034101
Full	Hateful comments/total comments	-12	-0.0024041	0.0007447	-0.0042883	-0.0005199
Full	Hateful comments/total comments	-11	0.0026959	0.0007547	0.0007862	0.0046056
Full	Hateful comments/total comments	-10	0.0006527	0.0021705	-0.0048393	0.0061447
Full	Hateful comments/total comments	-9	-0.0013126	0.1153300	-0.2931299	0.2905046
Full	Hateful comments/total comments	-8	-0.0009732	0.1145743	-0.2908784	0.2889321
Full	Hateful comments/total comments	-7	0.0005221	0.0011725	-0.0024446	0.0034889
Full	Hateful comments/total comments	-6	-0.0011703	0.0017372	-0.0055658	0.0032252
Full	Hateful comments/total comments	-5	-0.0003223	0.0009435	-0.0027095	0.0020649
Full	Hateful comments/total comments	-4	-0.0008225	0.0014905	-0.0045938	0.0029488
Full	Hateful comments/total comments	-3	-0.0026484	0.0026689	-0.0094014	0.0041047
Full	Hateful comments/total comments	-2	0.0025920	0.0030424	-0.0051061	0.0102902
Full	Hateful comments/total comments	-1	0.0014154	0.0012342	-0.0017074	0.0045382
Full	Hateful comments/total comments	0	-0.0016867	0.0027537	-0.0086543	0.0052810
Full	Hateful comments/total comments	1	0.0003726	0.0014366	-0.0032623	0.0040075
Full	Hateful comments/total comments	2	-0.0011146	0.0017013	-0.0054195	0.0031903
Full	Hateful comments/total comments	3	-0.0003421	0.0020650	-0.0055671	0.0048829
Full	Hateful comments/total comments	4	0.0010788	0.0015686	-0.0028901	0.0050477
Full	Hateful comments/total comments	5	-0.0000886	0.0026356	-0.0067574	0.0065803
Full	Hateful comments/total comments	6	-0.0007524	0.0014991	-0.0045456	0.0030409
Full	Hateful comments/total comments	7	0.0003842	0.0017399	-0.0040182	0.0047865
Full	Hateful comments/total comments	8	0.0014699	0.0025623	-0.0050135	0.0079533
Full	Hateful comments/total comments	9	-0.0006827	0.0018301	-0.0053133	0.0039478
Full	Hateful comments/total comments	10	0.0006649	0.0021658	-0.0048152	0.0061449
Full	Hateful comments/total comments	11	0.0011178	0.0025787	-0.0054069	0.0076426
Full	Hateful comments/total comments	12	0.0029496	0.0032851	-0.0053627	0.0112618
Full	Hateful comments/total comments	13	-0.0007715	0.0016108	-0.0048473	0.0033043
Full	Hateful comments/total comments	14	0.0021333	0.0028132	-0.0049849	0.0092515
Full	Hateful comments/total comments	15	0.0024010	0.0050587	-0.0103990	0.0152009

Full	Hateful comments/total comments	16	0.0017846	0.0036019	-0.0073293	0.0108985
Full	Hateful comments/total comments	17	0.0015649	0.0044072	-0.0095866	0.0127165
Full	Hateful comments/total comments	18	0.0023076	0.0061895	-0.0133536	0.0179689
Full	Hateful comments/total comments	19	0.0059923	0.0043068	-0.0049051	0.0168896
Full	Hateful comments/total comments	20	0.0010669	0.0023969	-0.0049979	0.0071316
Full	Hateful comments/total comments	21	0.0006917	0.0037250	-0.0087337	0.0101170
Full	Hateful comments/total comments	22	0.0013667	0.0038461	-0.0083651	0.0110984
Full	Hateful comments/total comments	23	0.0006551	0.0034792	-0.0081482	0.0094584
Full	Hateful comments/total comments	24	-0.0005155	0.0034388	-0.0092165	0.0081855
Full	Hateful comments/total comments	25	-0.0003562	0.0037061	-0.0097337	0.0090214
Full	Hateful comments/total comments	26	-0.0002088	0.0034760	-0.0090042	0.0085865
Full	Hateful comments/total comments	27	-0.0041512	0.0028333	-0.0113202	0.0030178
Full	Hateful comments/total comments	28	-0.0041956	0.0022389	-0.0098607	0.0014695
Full	Hateful comments/total comments	29	-0.0029241	0.0064669	-0.0192873	0.0134390
Full	Hateful comments/total comments	30	-0.0064666	0.0051628	-0.0195299	0.0065967
Full	Hateful comments/total comments	31	-0.0069909	0.0048109	-0.0191639	0.0051821
Full	Hateful comments/total comments	32	-0.0035628	0.0080565	-0.0239480	0.0168225
Full	Hateful comments/total comments	33	-0.0060150	0.0037592	-0.0155268	0.0034969
Full	Hateful comments/total comments	34	-0.0028905	0.0008341	-0.0050009	-0.0007800
Full	Hateful comments/total comments	35	-0.0059325	0.0031292	-0.0138503	0.0019853
Full	Hateful comments/total comments	36	-0.0012776	0.0023230	-0.0071555	0.0046003
Full	Hateful comments/total comments	37	-0.0037984	0.0030377	-0.0114847	0.0038878
Full	Hateful comments/total comments	38	-0.0040332	0.0015220	-0.0078843	-0.0001821
Full	Hateful comments/total comments	39	-0.0048904	0.0032112	-0.0130157	0.0032348
Full	Hateful comments/total comments	40	-0.0029065	0.0027514	-0.0098684	0.0040553
Full	Hateful comments/total comments	41	-0.0074019	0.0025570	-0.0138718	-0.0009321
Full	Hateful comments/total comments	42	-0.0067046	0.0072860	-0.0251403	0.0117310
Full	Hateful comments/total comments	43	-0.0152626	0.0061282	-0.0307687	0.0002435
Full	Hateful comments/total comments	44	-0.0142777	0.0077484	-0.0338835	0.0053280
Full	Hateful comments/total comments	45	-0.0140120	0.0006978	-0.0157775	-0.0122465
Full	Hateful comments/total comments	47	-0.0059615	NA	NA	NA
Full	Hateful comments/total comments	48	-0.0084276	NA	NA	NA
Full	Hateful comments/total comments	49	-0.0042865	NA	NA	NA
Full	Hateful comments/total comments	50	-0.0086599	NA	NA	NA
Full	Hateful comments/total comments	51	-0.0092292	0.0065405	-0.0257785	0.0073200
Full	Hateful comments/total comments	52	-0.0069031	0.0043928	-0.0180180	0.0042118
Full	Hateful comments/total comments	53	-0.0096346	0.0041104	-0.0200352	0.0007659
Full	Hateful comments/total comments	54	-0.0078700	0.0042465	-0.0186148	0.0028749
Full	Hateful comments/total comments	55	-0.0095707	0.0016726	-0.0138029	-0.0053386
Full	Hateful comments/total comments	56	-0.0121540	0.0105518	-0.0388531	0.0145451
Full	Hateful comments/total comments	57	-0.0064352	0.0000813	-0.0066409	-0.0062296

Full	Hateful comments/total comments	58	-0.0148221	0.0019057	-0.0196441	-0.0100001
Full	Hateful comments/total comments	59	-0.0109943	0.0293981	-0.0853797	0.0633911

sample	outcome	event.time	estimate	std.error	conf.low	conf.high
Most	Hateful comments/total comments	-44	0.0018165	0.0007319	-0.0000320	0.0036651
Most	Hateful comments/total comments	-43	0.0038260	0.0038688	-0.0059452	0.0135971
Most	Hateful comments/total comments	-42	0.0003445	0.0061465	-0.0151793	0.0158682
Most	Hateful comments/total comments	-41	0.0025496	0.0061676	-0.0130275	0.0181267
Most	Hateful comments/total comments	-40	-0.0069224	0.0044752	-0.0182252	0.0043803
Most	Hateful comments/total comments	-39	0.0100279	0.0031663	0.0020310	0.0180249
Most	Hateful comments/total comments	-38	0.0017284	0.0018246	-0.0028800	0.0063368
Most	Hateful comments/total comments	-37	-0.0008351	0.0044936	-0.0121844	0.0105141
Most	Hateful comments/total comments	-36	0.0004998	0.0069924	-0.0171604	0.0181600
Most	Hateful comments/total comments	-35	0.0042741	0.0051809	-0.0088109	0.0173592
Most	Hateful comments/total comments	-34	-0.0143492	0.0040454	-0.0245665	-0.0041320
Most	Hateful comments/total comments	-33	0.0156880	0.0056639	0.0013831	0.0299929
Most	Hateful comments/total comments	-32	0.0000758	0.0023120	-0.0057636	0.0059152
Most	Hateful comments/total comments	-31	-0.0087237	0.0041224	-0.0191354	0.0016880
Most	Hateful comments/total comments	-30	0.0032724	0.0016972	-0.0010141	0.0075588
Most	Hateful comments/total comments	-29	-0.0023623	0.0018939	-0.0071456	0.0024210
Most	Hateful comments/total comments	-28	0.0020801	0.0042352	-0.0086164	0.0127766
Most	Hateful comments/total comments	-27	-0.0069140	0.0028106	-0.0140126	0.0001847
Most	Hateful comments/total comments	-26	0.0037193	0.0038743	-0.0060659	0.0135044
Most	Hateful comments/total comments	-25	-0.0090826	0.0053019	-0.0224733	0.0043082
Most	Hateful comments/total comments	-24	0.0042494	0.0055159	-0.0096818	0.0181807
Most	Hateful comments/total comments	-23	-0.0038852	0.0034274	-0.0125415	0.0047711
Most	Hateful comments/total comments	-22	0.0055349	0.0041374	-0.0049147	0.0159844
Most	Hateful comments/total comments	-21	0.0000523	0.0032103	-0.0080557	0.0081603
Most	Hateful comments/total comments	-20	-0.0036870	0.0028655	-0.0109242	0.0035501
Most	Hateful comments/total comments	-19	-0.0023962	0.0069456	-0.0199383	0.0151460
Most	Hateful comments/total comments	-18	0.0088797	0.0080500	-0.0114517	0.0292112
Most	Hateful comments/total comments	-17	0.0024562	0.0031684	-0.0055461	0.0104585
Most	Hateful comments/total comments	-16	-0.0087385	0.0029968	-0.0163074	-0.0011695
Most	Hateful comments/total comments	-15	-0.0013655	0.0046330	-0.0130667	0.0103357
Most	Hateful comments/total comments	-14	0.0004166	0.0014937	-0.0033559	0.0041891
Most	Hateful comments/total comments	-13	0.0069362	0.0022015	0.0013760	0.0124963
Most	Hateful comments/total comments	-12	-0.0060530	0.0039467	-0.0160209	0.0039149
Most	Hateful comments/total comments	-11	0.0054841	0.0035031	-0.0033634	0.0143316
Most	Hateful comments/total comments	-10	-0.0047507	0.0031995	-0.0128314	0.0033301
Most	Hateful comments/total comments	-9	-0.0004816	0.0278498	-0.0708202	0.0698569
Most	Hateful comments/total comments	-8	-0.0020301	0.0288306	-0.0748457	0.0707855

Most	Hateful comments/total comments	-7	0.0047527	0.0014436	0.0011067	0.0083988
Most	Hateful comments/total comments	-6	-0.0031109	0.0030582	-0.0108348	0.0046131
Most	Hateful comments/total comments	-5	0.0026958	0.0037178	-0.0066941	0.0120856
Most	Hateful comments/total comments	-4	-0.0088253	0.0039315	-0.0187549	0.0011042
Most	Hateful comments/total comments	-3	0.0040160	0.0031015	-0.0038173	0.0118494
Most	Hateful comments/total comments	-2	-0.0001525	0.0048407	-0.0123783	0.0120734
Most	Hateful comments/total comments	-1	0.0071402	0.0022090	0.0015610	0.0127193
Most	Hateful comments/total comments	0	-0.0047466	0.0033844	-0.0132943	0.0038011
Most	Hateful comments/total comments	1	-0.0057745	0.0035253	-0.0146781	0.0031290
Most	Hateful comments/total comments	2	-0.0050282	0.0031789	-0.0130569	0.0030005
Most	Hateful comments/total comments	3	-0.0055267	0.0055569	-0.0195614	0.0085080
Most	Hateful comments/total comments	4	-0.0046773	0.0020018	-0.0097332	0.0003785
Most	Hateful comments/total comments	5	-0.0032490	0.0065693	-0.0198408	0.0133427
Most	Hateful comments/total comments	6	-0.0081836	0.0032119	-0.0162957	-0.0000715
Most	Hateful comments/total comments	7	-0.0039397	0.0038532	-0.0136714	0.0057920
Most	Hateful comments/total comments	8	-0.0028387	0.0038664	-0.0126037	0.0069264
Most	Hateful comments/total comments	9	-0.0050963	0.0021979	-0.0106474	0.0004548
Most	Hateful comments/total comments	10	-0.0057506	0.0036657	-0.0150088	0.0035075
Most	Hateful comments/total comments	11	-0.0052008	0.0042555	-0.0159487	0.0055471
Most	Hateful comments/total comments	12	-0.0061094	0.0034748	-0.0148855	0.0026667
Most	Hateful comments/total comments	13	-0.0042043	0.0029652	-0.0116934	0.0032848
Most	Hateful comments/total comments	14	-0.0023959	0.0032766	-0.0106715	0.0058797
Most	Hateful comments/total comments	15	-0.0028748	0.0055873	-0.0169863	0.0112366
Most	Hateful comments/total comments	16	-0.0062977	0.0051492	-0.0193028	0.0067074
Most	Hateful comments/total comments	17	-0.0058531	0.0076596	-0.0251985	0.0134922
Most	Hateful comments/total comments	18	-0.0005535	0.0074620	-0.0193998	0.0182928
Most	Hateful comments/total comments	19	0.0026141	0.0054429	-0.0111326	0.0163608
Most	Hateful comments/total comments	20	-0.0081642	0.0032718	-0.0164275	0.0000990
Most	Hateful comments/total comments	21	-0.0045278	0.0042634	-0.0152956	0.0062400
Most	Hateful comments/total comments	22	-0.0030126	0.0055583	-0.0170509	0.0110258
Most	Hateful comments/total comments	23	-0.0034040	0.0055743	-0.0174827	0.0106748
Most	Hateful comments/total comments	24	-0.0043131	0.0039120	-0.0141935	0.0055672
Most	Hateful comments/total comments	25	-0.0024952	0.0054627	-0.0162919	0.0113015
Most	Hateful comments/total comments	26	-0.0017926	0.0045283	-0.0132294	0.0096441
Most	Hateful comments/total comments	27	-0.0078221	0.0073086	-0.0262810	0.0106368
Most	Hateful comments/total comments	28	-0.0077335	0.0038471	-0.0174498	0.0019828
Most	Hateful comments/total comments	29	0.0039855	0.0105052	-0.0225469	0.0305178
Most	Hateful comments/total comments	30	-0.0080639	0.0035981	-0.0171514	0.0010237
Most	Hateful comments/total comments	31	-0.0090271	0.0015174	-0.0128594	-0.0051947
Most	Hateful comments/total comments	32	-0.0053069	0.0124292	-0.0366985	0.0260847
Most	Hateful comments/total comments	33	-0.0119065	0.0027366	-0.0188182	-0.0049947

Most	Hateful comments/total comments	34	-0.0089495	0.0040081	-0.0190726	0.0011735
Most	Hateful comments/total comments	35	-0.0095032	0.0033081	-0.0178582	-0.0011481
Most	Hateful comments/total comments	36	-0.0075706	0.0030028	-0.0151546	0.0000135
Most	Hateful comments/total comments	37	-0.0099975	0.0038776	-0.0197908	-0.0002041
Most	Hateful comments/total comments	38	-0.0074461	0.0032346	-0.0156155	0.0007232
Most	Hateful comments/total comments	39	-0.0104857	0.0036334	-0.0196625	-0.0013090
Most	Hateful comments/total comments	40	-0.0101037	0.0020228	-0.0152125	-0.0049948
Most	Hateful comments/total comments	41	-0.0137409	0.0011914	-0.0167501	-0.0107318
Most	Hateful comments/total comments	42	-0.0127529	0.0039087	-0.0226249	-0.0028809
Most	Hateful comments/total comments	43	-0.0061619	0.0126120	-0.0380151	0.0256914
Most	Hateful comments/total comments	44	-0.0091202	0.0153208	-0.0478149	0.0295746
Most	Hateful comments/total comments	45	-0.0166877	0.0089059	-0.0391809	0.0058055
Most	Hateful comments/total comments	47	-0.0039748	NA	NA	NA
Most	Hateful comments/total comments	48	-0.0051514	NA	NA	NA
Most	Hateful comments/total comments	49	-0.0096247	NA	NA	NA
Most	Hateful comments/total comments	50	-0.0100726	NA	NA	NA
Most	Hateful comments/total comments	51	-0.0058654	0.0130456	-0.0388139	0.0270831
Most	Hateful comments/total comments	52	-0.0119447	0.0018319	-0.0165713	-0.0073180
Most	Hateful comments/total comments	53	-0.0105777	0.0034780	-0.0193619	-0.0017934
Most	Hateful comments/total comments	54	-0.0112590	0.0038910	-0.0210863	-0.0014316
Most	Hateful comments/total comments	55	-0.0124244	0.0057394	-0.0269201	0.0020713
Most	Hateful comments/total comments	56	-0.0106549	0.0058874	-0.0255243	0.0042145
Most	Hateful comments/total comments	57	-0.0007309	0.0130953	-0.0338049	0.0323431
Most	Hateful comments/total comments	58	-0.0081446	0.0096779	-0.0325874	0.0162982
Most	Hateful comments/total comments	59	-0.0100447	0.0093701	-0.0337101	0.0136207

sample	outcome	event.time	estimate	std.error	conf.low	conf.high
Middle	Hateful comments/total comments	-44	-0.0055871	0.0023060	-0.0112716	0.0000974
Middle	Hateful comments/total comments	-43	-0.0063544	0.0033980	-0.0147307	0.0020219
Middle	Hateful comments/total comments	-42	0.0025903	0.0051892	-0.0102016	0.0153823
Middle	Hateful comments/total comments	-41	0.0073766	0.0079872	-0.0123124	0.0270657
Middle	Hateful comments/total comments	-40	-0.0092246	0.0044985	-0.0203138	0.0018647
Middle	Hateful comments/total comments	-39	0.0126738	0.0038867	0.0030928	0.0222547
Middle	Hateful comments/total comments	-38	-0.0085277	0.0035933	-0.0173854	0.0003300
Middle	Hateful comments/total comments	-37	0.0042975	0.0041615	-0.0059610	0.0145559
Middle	Hateful comments/total comments	-36	0.0004817	0.0026110	-0.0059548	0.0069181
Middle	Hateful comments/total comments	-35	0.0020373	0.0025676	-0.0042921	0.0083667
Middle	Hateful comments/total comments	-34	-0.0038068	0.0077638	-0.0229454	0.0153318
Middle	Hateful comments/total comments	-33	0.0013122	0.0046734	-0.0102082	0.0128326
Middle	Hateful comments/total comments	-32	0.0120763	0.0075867	-0.0066257	0.0307782
Middle	Hateful comments/total comments	-31	-0.0226016	0.0051380	-0.0352672	-0.0099359

Middle	Hateful comments/total comments	-30	0.0077800	0.0025176	0.0015740	0.0139861
Middle	Hateful comments/total comments	-29	0.0011875	0.0045830	-0.0101100	0.0124849
Middle	Hateful comments/total comments	-28	0.0028105	0.0025716	-0.0035288	0.0091498
Middle	Hateful comments/total comments	-27	-0.0062849	0.0034389	-0.0147622	0.0021923
Middle	Hateful comments/total comments	-26	0.0055756	0.0034947	-0.0030391	0.0141903
Middle	Hateful comments/total comments	-25	-0.0017971	0.0028576	-0.0088413	0.0052472
Middle	Hateful comments/total comments	-24	0.0009129	0.0030890	-0.0067018	0.0085277
Middle	Hateful comments/total comments	-23	-0.0003382	0.0051274	-0.0129776	0.0123012
Middle	Hateful comments/total comments	-22	-0.0038722	0.0056718	-0.0178538	0.0101094
Middle	Hateful comments/total comments	-21	-0.0007669	0.0039121	-0.0104106	0.0088769
Middle	Hateful comments/total comments	-20	0.0008934	0.0032027	-0.0070016	0.0087884
Middle	Hateful comments/total comments	-19	-0.0006582	0.0032475	-0.0086637	0.0073473
Middle	Hateful comments/total comments	-18	-0.0002763	0.0039756	-0.0100765	0.0095239
Middle	Hateful comments/total comments	-17	0.0075167	0.0031369	-0.0002161	0.0152495
Middle	Hateful comments/total comments	-16	-0.0024827	0.0026834	-0.0090975	0.0041320
Middle	Hateful comments/total comments	-15	-0.0022260	0.0031898	-0.0100890	0.0056371
Middle	Hateful comments/total comments	-14	-0.0013928	0.0029749	-0.0087262	0.0059405
Middle	Hateful comments/total comments	-13	0.0040902	0.0014703	0.0004656	0.0077147
Middle	Hateful comments/total comments	-12	-0.0079273	0.0011138	-0.0106730	-0.0051816
Middle	Hateful comments/total comments	-11	0.0042463	0.0030475	-0.0032660	0.0117586
Middle	Hateful comments/total comments	-10	0.0028102	0.0044319	-0.0081148	0.0137352
Middle	Hateful comments/total comments	-9	-0.0027033	0.0421289	-0.1065548	0.1011482
Middle	Hateful comments/total comments	-8	0.0022179	0.0413855	-0.0998011	0.1042369
Middle	Hateful comments/total comments	-7	-0.0008909	0.0021594	-0.0062139	0.0044321
Middle	Hateful comments/total comments	-6	-0.0002194	0.0029361	-0.0074572	0.0070184
Middle	Hateful comments/total comments	-5	-0.0048195	0.0020812	-0.0099498	0.0003109
Middle	Hateful comments/total comments	-4	0.0040625	0.0024336	-0.0019364	0.0100615
Middle	Hateful comments/total comments	-3	-0.0038443	0.0051484	-0.0165356	0.0088471
Middle	Hateful comments/total comments	-2	0.0040989	0.0042602	-0.0064029	0.0146008
Middle	Hateful comments/total comments	-1	-0.0003004	0.0064600	-0.0162249	0.0156241
Middle	Hateful comments/total comments	0	0.0021317	0.0048851	-0.0099106	0.0141739
Middle	Hateful comments/total comments	1	0.0014463	0.0038632	-0.0080768	0.0109694
Middle	Hateful comments/total comments	2	-0.0010980	0.0036210	-0.0100242	0.0078281
Middle	Hateful comments/total comments	3	0.0018218	0.0064689	-0.0141247	0.0177682
Middle	Hateful comments/total comments	4	-0.0002774	0.0039916	-0.0101172	0.0095623
Middle	Hateful comments/total comments	5	-0.0002244	0.0034585	-0.0087500	0.0083012
Middle	Hateful comments/total comments	6	-0.0012593	0.0035022	-0.0098924	0.0073738
Middle	Hateful comments/total comments	7	-0.0016547	0.0032606	-0.0096924	0.0063831
Middle	Hateful comments/total comments	8	0.0027764	0.0031810	-0.0050650	0.0106178
Middle	Hateful comments/total comments	9	-0.0036034	0.0047006	-0.0151909	0.0079841
Middle	Hateful comments/total comments	10	-0.0025962	0.0023787	-0.0084598	0.0032675

Middle	Hateful comments/total comments	11	0.0016969	0.0037038	-0.0074334	0.0108272
Middle	Hateful comments/total comments	12	0.0049038	0.0086506	-0.0164206	0.0262282
Middle	Hateful comments/total comments	13	-0.0042310	0.0054693	-0.0177132	0.0092512
Middle	Hateful comments/total comments	14	0.0018463	0.0029345	-0.0053876	0.0090802
Middle	Hateful comments/total comments	15	0.0054985	0.0068009	-0.0112664	0.0222633
Middle	Hateful comments/total comments	16	0.0020362	0.0054625	-0.0114293	0.0155017
Middle	Hateful comments/total comments	17	0.0045409	0.0058633	-0.0099127	0.0189946
Middle	Hateful comments/total comments	18	0.0046033	0.0139929	-0.0298904	0.0390969
Middle	Hateful comments/total comments	19	0.0102355	0.0056272	-0.0036360	0.0241070
Middle	Hateful comments/total comments	20	0.0032301	0.0039910	-0.0066080	0.0130682
Middle	Hateful comments/total comments	21	0.0028562	0.0047829	-0.0089341	0.0146466
Middle	Hateful comments/total comments	22	0.0009921	0.0052892	-0.0120462	0.0140304
Middle	Hateful comments/total comments	23	0.0007943	0.0075968	-0.0179326	0.0195211
Middle	Hateful comments/total comments	24	-0.0009613	0.0038064	-0.0103445	0.0084219
Middle	Hateful comments/total comments	25	-0.0010912	0.0061067	-0.0161448	0.0139624
Middle	Hateful comments/total comments	26	-0.0001932	0.0036156	-0.0091061	0.0087197
Middle	Hateful comments/total comments	27	-0.0005062	0.0025811	-0.0068689	0.0058564
Middle	Hateful comments/total comments	28	-0.0035538	0.0088057	-0.0252605	0.0181529
Middle	Hateful comments/total comments	29	-0.0055830	0.0129971	-0.0376221	0.0264561
Middle	Hateful comments/total comments	30	-0.0061995	0.0163636	-0.0465373	0.0341382
Middle	Hateful comments/total comments	31	-0.0051500	0.0016291	-0.0091658	-0.0011342
Middle	Hateful comments/total comments	32	-0.0048544	0.0122412	-0.0350301	0.0253214
Middle	Hateful comments/total comments	33	-0.0097937	0.0090205	-0.0320301	0.0124427
Middle	Hateful comments/total comments	34	-0.0011229	0.0045322	-0.0122953	0.0100494
Middle	Hateful comments/total comments	35	-0.0039689	0.0059628	-0.0186678	0.0107300
Middle	Hateful comments/total comments	36	-0.0015828	0.0053842	-0.0148555	0.0116898
Middle	Hateful comments/total comments	37	-0.0042889	0.0075043	-0.0227875	0.0142098
Middle	Hateful comments/total comments	38	-0.0052281	0.0038463	-0.0147097	0.0042535
Middle	Hateful comments/total comments	39	-0.0081501	0.0083268	-0.0286764	0.0123762
Middle	Hateful comments/total comments	40	-0.0028022	0.0080881	-0.0227400	0.0171357
Middle	Hateful comments/total comments	41	-0.0059429	0.0085351	-0.0269828	0.0150969
Middle	Hateful comments/total comments	42	-0.0042038	0.0147122	-0.0404707	0.0320630
Middle	Hateful comments/total comments	43	-0.0164304	0.0451836	-0.1278119	0.0949511
Middle	Hateful comments/total comments	44	-0.0179230	0.0690367	-0.1881046	0.1522585
Middle	Hateful comments/total comments	45	-0.0131031	0.0502927	-0.1370792	0.1108729
Middle	Hateful comments/total comments	47	-0.0104615	NA	NA	NA
Middle	Hateful comments/total comments	48	-0.0083482	NA	NA	NA
Middle	Hateful comments/total comments	49	-0.0060225	NA	NA	NA
Middle	Hateful comments/total comments	50	-0.0122857	NA	NA	NA
Middle	Hateful comments/total comments	51	-0.0103581	0.0125591	-0.0413175	0.0206013
Middle	Hateful comments/total comments	52	-0.0078265	0.0154214	-0.0458417	0.0301888

Middle	Hateful comments/total comments	53	-0.0105937	0.0100955	-0.0354800	0.0142926
Middle	Hateful comments/total comments	54	-0.0090493	0.0166889	-0.0501888	0.0320903
Middle	Hateful comments/total comments	55	-0.0104985	0.0165003	-0.0511732	0.0301761
Middle	Hateful comments/total comments	56	-0.0148592	0.0998430	-0.2609811	0.2312627
Middle	Hateful comments/total comments	57	-0.0032262	0.0788755	-0.1976613	0.1912090
Middle	Hateful comments/total comments	58	-0.0230779	0.0702148	-0.1961638	0.1500080
Middle	Hateful comments/total comments	59	-0.0134770	0.1675665	-0.4265434	0.3995893

sample	outcome	event.time	estimate	std.error	conf.low	conf.high
Least	Hateful comments/total comments	-44	0.0016433	0.0023551	-0.0044840	0.0077706
Least	Hateful comments/total comments	-43	0.0076231	0.0040530	-0.0029218	0.0181680
Least	Hateful comments/total comments	-42	-0.0081635	0.0011930	-0.0112674	-0.0050597
Least	Hateful comments/total comments	-41	-0.0034984	0.0050272	-0.0165780	0.0095812
Least	Hateful comments/total comments	-40	0.0060993	0.0066711	-0.0112572	0.0234559
Least	Hateful comments/total comments	-39	-0.0012090	0.0041207	-0.0119302	0.0095122
Least	Hateful comments/total comments	-38	0.0064751	0.0012665	0.0031798	0.0097703
Least	Hateful comments/total comments	-37	-0.0039860	0.0018547	-0.0088115	0.0008395
Least	Hateful comments/total comments	-36	0.0046485	0.0041897	-0.0062520	0.0155489
Least	Hateful comments/total comments	-35	-0.0083737	0.0025913	-0.0151156	-0.0016318
Least	Hateful comments/total comments	-34	-0.0001105	0.0024666	-0.0065280	0.0063070
Least	Hateful comments/total comments	-33	0.0065464	0.0031145	-0.0015568	0.0146496
Least	Hateful comments/total comments	-32	-0.0060878	0.0036365	-0.0155493	0.0033736
Least	Hateful comments/total comments	-31	0.0005515	0.0037231	-0.0091351	0.0102380
Least	Hateful comments/total comments	-30	0.0040716	0.0039577	-0.0062254	0.0143685
Least	Hateful comments/total comments	-29	-0.0048520	0.0024696	-0.0112773	0.0015733
Least	Hateful comments/total comments	-28	0.0017342	0.0009980	-0.0008624	0.0043307
Least	Hateful comments/total comments	-27	0.0087420	0.0067185	-0.0087378	0.0262218
Least	Hateful comments/total comments	-26	-0.0039354	0.0027671	-0.0111347	0.0032639
Least	Hateful comments/total comments	-25	0.0030986	0.0017574	-0.0014737	0.0076709
Least	Hateful comments/total comments	-24	-0.0115750	0.0045847	-0.0235033	0.0003533
Least	Hateful comments/total comments	-23	0.0064591	0.0023277	0.0004030	0.0125153
Least	Hateful comments/total comments	-22	0.0007290	0.0063934	-0.0159051	0.0173632
Least	Hateful comments/total comments	-21	0.0012411	0.0025536	-0.0054029	0.0078851
Least	Hateful comments/total comments	-20	-0.0008064	0.0034043	-0.0096636	0.0080508
Least	Hateful comments/total comments	-19	0.0023785	0.0015874	-0.0017516	0.0065086
Least	Hateful comments/total comments	-18	-0.0029697	0.0042026	-0.0139038	0.0079644
Least	Hateful comments/total comments	-17	0.0031421	0.0022941	-0.0028265	0.0091107
Least	Hateful comments/total comments	-16	-0.0021073	0.0034302	-0.0110317	0.0068172
Least	Hateful comments/total comments	-15	0.0008648	0.0017628	-0.0037216	0.0054513
Least	Hateful comments/total comments	-14	0.0033661	0.0012626	0.0000811	0.0066512
Least	Hateful comments/total comments	-13	-0.0067462	0.0025899	-0.0134846	-0.0000078

Least	Hateful comments/total comments	-12	0.0045906	0.0028588	-0.0028473	0.0120285
Least	Hateful comments/total comments	-11	-0.0004639	0.0017238	-0.0049489	0.0040211
Least	Hateful comments/total comments	-10	0.0019296	0.0036496	-0.0075658	0.0114249
Least	Hateful comments/total comments	-9	-0.0007204	0.0461726	-0.1208505	0.1194098
Least	Hateful comments/total comments	-8	-0.0028637	0.0463476	-0.1234492	0.1177217
Least	Hateful comments/total comments	-7	-0.0007971	0.0015916	-0.0049379	0.0033438
Least	Hateful comments/total comments	-6	-0.0006817	0.0013599	-0.0042198	0.0028565
Least	Hateful comments/total comments	-5	0.0018412	0.0022852	-0.0041044	0.0077867
Least	Hateful comments/total comments	-4	-0.0008573	0.0022052	-0.0065947	0.0048802
Least	Hateful comments/total comments	-3	-0.0052072	0.0016222	-0.0094278	-0.0009867
Least	Hateful comments/total comments	-2	0.0029030	0.0033939	-0.0059270	0.0117330
Least	Hateful comments/total comments	-1	-0.0004592	0.0021171	-0.0059673	0.0050489
Least	Hateful comments/total comments	0	-0.0030365	0.0024974	-0.0095341	0.0034611
Least	Hateful comments/total comments	1	0.0031782	0.0020754	-0.0022216	0.0085780
Least	Hateful comments/total comments	2	0.0009340	0.0022067	-0.0048074	0.0066754
Least	Hateful comments/total comments	3	0.0004135	0.0020383	-0.0048896	0.0057166
Least	Hateful comments/total comments	4	0.0052684	0.0053566	-0.0086682	0.0192051
Least	Hateful comments/total comments	5	0.0017831	0.0032019	-0.0065475	0.0101138
Least	Hateful comments/total comments	6	0.0036444	0.0035855	-0.0056841	0.0129729
Least	Hateful comments/total comments	7	0.0045585	0.0051511	-0.0088434	0.0179605
Least	Hateful comments/total comments	8	0.0030907	0.0028634	-0.0043591	0.0105406
Least	Hateful comments/total comments	9	0.0042087	0.0038358	-0.0057713	0.0141886
Least	Hateful comments/total comments	10	0.0072498	0.0036355	-0.0022089	0.0167085
Least	Hateful comments/total comments	11	0.0045711	0.0034521	-0.0044105	0.0135526
Least	Hateful comments/total comments	12	0.0063063	0.0043622	-0.0050431	0.0176557
Least	Hateful comments/total comments	13	0.0039459	0.0041339	-0.0068094	0.0147013
Least	Hateful comments/total comments	14	0.0049670	0.0057373	-0.0099600	0.0198941
Least	Hateful comments/total comments	15	0.0022258	0.0042079	-0.0087220	0.0131737
Least	Hateful comments/total comments	16	0.0053138	0.0026420	-0.0015600	0.0121876
Least	Hateful comments/total comments	17	0.0032195	0.0052582	-0.0104610	0.0169001
Least	Hateful comments/total comments	18	0.0013150	0.0070570	-0.0170456	0.0196756
Least	Hateful comments/total comments	19	0.0041784	0.0040311	-0.0063096	0.0146664
Least	Hateful comments/total comments	20	0.0044430	0.0036228	-0.0049826	0.0138686
Least	Hateful comments/total comments	21	0.0014694	0.0035678	-0.0078131	0.0107519
Least	Hateful comments/total comments	22	0.0040697	0.0040231	-0.0063974	0.0145368
Least	Hateful comments/total comments	23	0.0024706	0.0038943	-0.0076615	0.0126027
Least	Hateful comments/total comments	24	0.0016454	0.0036964	-0.0079717	0.0112626
Least	Hateful comments/total comments	25	0.0013865	0.0043434	-0.0099140	0.0126871
Least	Hateful comments/total comments	26	0.0007811	0.0041914	-0.0101240	0.0116862
Least	Hateful comments/total comments	27	-0.0050702	0.0010903	-0.0079070	-0.0022334
Least	Hateful comments/total comments	28	-0.0027222	0.0026623	-0.0096490	0.0042045

Least	Hateful comments/total comments	29	-0.0040066	0.0090821	-0.0276361	0.0196229
Least	Hateful comments/total comments	30	-0.0061315	0.0097369	-0.0314645	0.0192015
Least	Hateful comments/total comments	31	-0.0074175	0.0104698	-0.0346575	0.0198225
Least	Hateful comments/total comments	32	-0.0016932	0.0042851	-0.0128420	0.0094556
Least	Hateful comments/total comments	33	-0.0001548	0.0115484	-0.0302011	0.0298915
Least	Hateful comments/total comments	34	-0.0011871	0.0044309	-0.0127152	0.0103410
Least	Hateful comments/total comments	35	-0.0056645	0.0039114	-0.0158410	0.0045120
Least	Hateful comments/total comments	36	0.0022002	0.0031227	-0.0059243	0.0103247
Least	Hateful comments/total comments	37	-0.0002212	0.0061875	-0.0163196	0.0158773
Least	Hateful comments/total comments	38	-0.0013256	0.0054374	-0.0154724	0.0128211
Least	Hateful comments/total comments	39	0.0005865	0.0047663	-0.0118143	0.0129873
Least	Hateful comments/total comments	40	0.0007090	0.0047656	-0.0116900	0.0131079
Least	Hateful comments/total comments	41	-0.0053085	0.0055013	-0.0196217	0.0090047
Least	Hateful comments/total comments	42	-0.0050036	0.0122739	-0.0369374	0.0269301
Least	Hateful comments/total comments	43	-0.0221574	0.0301286	-0.1005449	0.0562300
Least	Hateful comments/total comments	44	-0.0160371	0.0093204	-0.0402866	0.0082124
Least	Hateful comments/total comments	45	-0.0123920	0.0688259	-0.1914607	0.1666767
Least	Hateful comments/total comments	47	-0.0031077	NA	NA	NA
Least	Hateful comments/total comments	48	-0.0097169	NA	NA	NA
Least	Hateful comments/total comments	49	-0.0009016	NA	NA	NA
Least	Hateful comments/total comments	50	-0.0052331	NA	NA	NA
Least	Hateful comments/total comments	51	-0.0100145	0.0057663	-0.0250171	0.0049882
Least	Hateful comments/total comments	52	-0.0036885	0.0122602	-0.0355866	0.0282096
Least	Hateful comments/total comments	53	-0.0084206	0.0112094	-0.0375849	0.0207436
Least	Hateful comments/total comments	54	-0.0052738	0.0014943	-0.0091617	-0.0013858
Least	Hateful comments/total comments	55	-0.0074354	0.0098384	-0.0330325	0.0181617
Least	Hateful comments/total comments	56	-0.0114804	0.0078942	-0.0320193	0.0090584
Least	Hateful comments/total comments	57	-0.0135944	0.0107291	-0.0415089	0.0143200
Least	Hateful comments/total comments	58	-0.0145538	0.0135107	-0.0497053	0.0205978
Least	Hateful comments/total comments	59	-0.0100138	0.0020356	-0.0153099	-0.0047177

Audience outdegree

Average effect by length of exposure (Callaway and Sant'Anna)

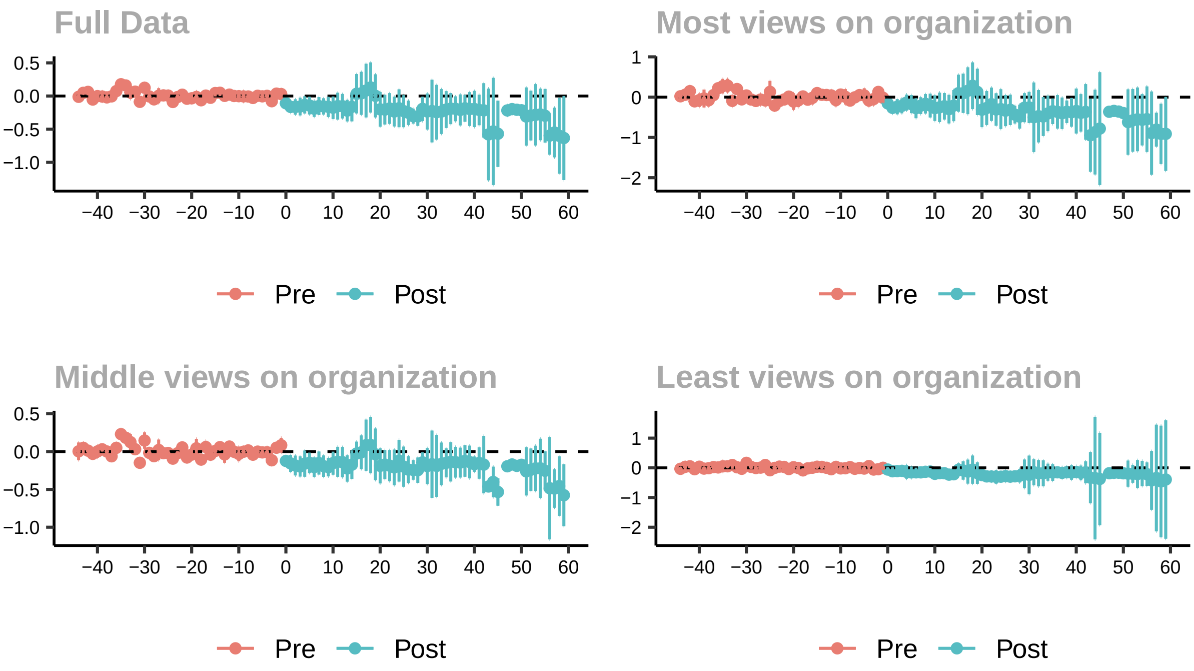

Long timeframe with sampled control groups

sample	outcome	event.time	estimate	std.error	conf.low	conf.high
Full	Audience outdegree	-44	-0.0122413	0.0184907	-0.0592672	0.0347845
Full	Audience outdegree	-43	0.0481967	0.0086925	0.0260897	0.0703037
Full	Audience outdegree	-42	0.0622542	0.0202495	0.0107553	0.1137530
Full	Audience outdegree	-41	-0.0534704	0.0188878	-0.1015063	-0.0054345
Full	Audience outdegree	-40	-0.0017574	0.0256819	-0.0670721	0.0635574
Full	Audience outdegree	-39	-0.0079069	0.0306436	-0.0858405	0.0700267
Full	Audience outdegree	-38	-0.0207668	0.0217921	-0.0761890	0.0346553
Full	Audience outdegree	-37	-0.0052974	0.0170293	-0.0486066	0.0380118
Full	Audience outdegree	-36	0.0742614	0.0221514	0.0179253	0.1305974
Full	Audience outdegree	-35	0.1772251	0.0144274	0.1405331	0.2139172
Full	Audience outdegree	-34	0.1591147	0.0204709	0.1070527	0.2111768
Full	Audience outdegree	-33	0.0529281	0.0391570	-0.0466570	0.1525131
Full	Audience outdegree	-32	0.0657546	0.0141360	0.0298036	0.1017056
Full	Audience outdegree	-31	-0.0878373	0.0156336	-0.1275969	-0.0480776
Full	Audience outdegree	-30	0.1244937	0.0288425	0.0511408	0.1978466
Full	Audience outdegree	-29	-0.0068463	0.0091765	-0.0301842	0.0164917
Full	Audience outdegree	-28	-0.0506058	0.0111754	-0.0790275	-0.0221842
Full	Audience outdegree	-27	0.0037524	0.0442295	-0.1087329	0.1162378
Full	Audience outdegree	-26	0.0095456	0.0260384	-0.0566760	0.0757671

Full	Audience outdegree	-25	0.0060423	0.0288003	-0.0672031	0.0792878
Full	Audience outdegree	-24	-0.0900859	0.0109742	-0.1179956	-0.0621761
Full	Audience outdegree	-23	-0.0157221	0.0138733	-0.0510051	0.0195609
Full	Audience outdegree	-22	0.0193892	0.0167555	-0.0232236	0.0620021
Full	Audience outdegree	-21	-0.0406875	0.0166314	-0.0829849	0.0016099
Full	Audience outdegree	-20	-0.0349794	0.0288260	-0.1082903	0.0383315
Full	Audience outdegree	-19	-0.0141819	0.0330304	-0.0981856	0.0698217
Full	Audience outdegree	-18	-0.0643430	0.0081535	-0.0850790	-0.0436069
Full	Audience outdegree	-17	0.0060454	0.0200634	-0.0449803	0.0570712
Full	Audience outdegree	-16	-0.0265947	0.0240363	-0.0877244	0.0345351
Full	Audience outdegree	-15	0.0445788	0.0198555	-0.0059182	0.0950758
Full	Audience outdegree	-14	0.0493511	0.0194416	-0.0000932	0.0987953
Full	Audience outdegree	-13	0.0020822	0.0231802	-0.0568702	0.0610345
Full	Audience outdegree	-12	0.0212453	0.0119164	-0.0090607	0.0515513
Full	Audience outdegree	-11	0.0002697	0.0246000	-0.0622935	0.0628329
Full	Audience outdegree	-10	-0.0025539	0.0225007	-0.0597783	0.0546705
Full	Audience outdegree	-9	-0.0057960	0.0326766	-0.0889000	0.0773079
Full	Audience outdegree	-8	-0.0069608	0.0182188	-0.0532952	0.0393736
Full	Audience outdegree	-7	-0.0293415	0.0150073	-0.0675083	0.0088253
Full	Audience outdegree	-6	0.0033663	0.0178959	-0.0421470	0.0488796
Full	Audience outdegree	-5	-0.0050950	0.0209062	-0.0582640	0.0480740
Full	Audience outdegree	-4	0.0025426	0.0148795	-0.0352994	0.0403845
Full	Audience outdegree	-3	-0.0788616	0.0241160	-0.1401938	-0.0175294
Full	Audience outdegree	-2	0.0360242	0.0141588	0.0000152	0.0720332
Full	Audience outdegree	-1	0.0293343	0.0290260	-0.0444854	0.1031540
Full	Audience outdegree	0	-0.1094732	0.0197401	-0.1596768	-0.0592697
Full	Audience outdegree	1	-0.1643267	0.0351806	-0.2537988	-0.0748546
Full	Audience outdegree	2	-0.1658208	0.0452533	-0.2809101	-0.0507316
Full	Audience outdegree	3	-0.1586341	0.0522895	-0.2916179	-0.0256503
Full	Audience outdegree	4	-0.1339740	0.0506754	-0.2628528	-0.0050952
Full	Audience outdegree	5	-0.1435545	0.0522630	-0.2764709	-0.0106381
Full	Audience outdegree	6	-0.1915435	0.0481844	-0.3140872	-0.0689998
Full	Audience outdegree	7	-0.1514718	0.0528489	-0.2858784	-0.0170653
Full	Audience outdegree	8	-0.1570254	0.0478061	-0.2786069	-0.0354439
Full	Audience outdegree	9	-0.1633546	0.0594755	-0.3146140	-0.0120952
Full	Audience outdegree	10	-0.1764919	0.0666738	-0.3460582	-0.0069256
Full	Audience outdegree	11	-0.1528224	0.0797128	-0.3555496	0.0499049
Full	Audience outdegree	12	-0.1534686	0.0722159	-0.3371297	0.0301925
Full	Audience outdegree	13	-0.2205641	0.0634412	-0.3819092	-0.0592190
Full	Audience outdegree	14	-0.1638233	0.0855909	-0.3815000	0.0538533
Full	Audience outdegree	15	0.0341751	0.1161485	-0.2612163	0.3295665

Full	Audience outdegree	16	0.0407094	0.1270788	-0.2824802	0.3638990
Full	Audience outdegree	17	0.0822033	0.1589854	-0.3221317	0.4865384
Full	Audience outdegree	18	0.1290361	0.1483938	-0.2483622	0.5064343
Full	Audience outdegree	19	0.0024392	0.1272764	-0.3212530	0.3261314
Full	Audience outdegree	20	-0.2009980	0.1027718	-0.4623695	0.0603735
Full	Audience outdegree	21	-0.2049518	0.0881576	-0.4291563	0.0192526
Full	Audience outdegree	22	-0.1882781	0.0905136	-0.4184743	0.0419182
Full	Audience outdegree	23	-0.2340852	0.0878303	-0.4574573	-0.0107132
Full	Audience outdegree	24	-0.1850335	0.1120677	-0.4700466	0.0999796
Full	Audience outdegree	25	-0.2285362	0.0949954	-0.4701305	0.0130581
Full	Audience outdegree	26	-0.2531146	0.0716728	-0.4353944	-0.0708348
Full	Audience outdegree	27	-0.3037247	0.0442656	-0.4163018	-0.1911475
Full	Audience outdegree	28	-0.3078686	0.0556114	-0.4493007	-0.1664366
Full	Audience outdegree	29	-0.1941456	0.0724026	-0.3782814	-0.0100097
Full	Audience outdegree	30	-0.2331406	0.1065436	-0.5041047	0.0378235
Full	Audience outdegree	31	-0.2264422	0.1857537	-0.6988552	0.2459708
Full	Audience outdegree	32	-0.2439495	0.1617232	-0.6552476	0.1673485
Full	Audience outdegree	33	-0.2341482	0.1325358	-0.5712163	0.1029198
Full	Audience outdegree	34	-0.2047786	0.1089683	-0.4819092	0.0723520
Full	Audience outdegree	35	-0.1902525	0.0921743	-0.4246721	0.0441671
Full	Audience outdegree	36	-0.1931683	0.0734350	-0.3799298	-0.0064069
Full	Audience outdegree	37	-0.2095179	0.0915469	-0.4423420	0.0233062
Full	Audience outdegree	38	-0.1848018	0.0798968	-0.3879970	0.0183934
Full	Audience outdegree	39	-0.1966933	0.0995730	-0.4499295	0.0565429
Full	Audience outdegree	40	-0.1957924	0.1070247	-0.4679800	0.0763953
Full	Audience outdegree	41	-0.2095590	0.0906963	-0.4402198	0.0211019
Full	Audience outdegree	42	-0.2170078	0.1608709	-0.6261382	0.1921226
Full	Audience outdegree	43	-0.5794009	0.2718770	-1.2708446	0.1120427
Full	Audience outdegree	44	-0.5342123	0.3173646	-1.3413409	0.2729164
Full	Audience outdegree	45	-0.5691424	0.1965184	-1.0689323	-0.0693525
Full	Audience outdegree	47	-0.2176815	0.0313104	-0.2973109	-0.1380521
Full	Audience outdegree	48	-0.1977778	0.0272640	-0.2671161	-0.1284394
Full	Audience outdegree	49	-0.2074147	0.0274911	-0.2773306	-0.1374988
Full	Audience outdegree	50	-0.2149642	0.0274911	-0.2848801	-0.1450483
Full	Audience outdegree	51	-0.3093598	0.1723315	-0.7476370	0.1289174
Full	Audience outdegree	52	-0.2937141	0.1476884	-0.6693184	0.0818902
Full	Audience outdegree	53	-0.2833144	0.1811969	-0.7441384	0.1775096
Full	Audience outdegree	54	-0.2782749	0.1524368	-0.6659554	0.1094056
Full	Audience outdegree	55	-0.2976763	0.1589785	-0.7019939	0.1066413
Full	Audience outdegree	56	-0.5968921	0.1129892	-0.8842486	-0.3095356
Full	Audience outdegree	57	-0.5505236	0.1467899	-0.9238428	-0.1772044

Full	Audience outdegree	58	-0.6001748	0.2245363	-1.1712203	-0.0291293
Full	Audience outdegree	59	-0.6334635	0.2494381	-1.2678399	0.0009128

sample	outcome	event.time	estimate	std.error	conf.low	conf.high
Most	Audience outdegree	-44	0.0214033	0.0330872	-0.0652096	0.1080162
Most	Audience outdegree	-43	0.0588841	0.0469153	-0.0639269	0.1816952
Most	Audience outdegree	-42	0.1489487	0.0486196	0.0216764	0.2762210
Most	Audience outdegree	-41	-0.1047654	0.0328448	-0.1907437	-0.0187871
Most	Audience outdegree	-40	-0.0740188	0.0636302	-0.2405847	0.0925472
Most	Audience outdegree	-39	-0.0334541	0.0847387	-0.2552762	0.1883681
Most	Audience outdegree	-38	-0.0764618	0.0610147	-0.2361811	0.0832575
Most	Audience outdegree	-37	0.0503992	0.0215442	-0.0059975	0.1067959
Most	Audience outdegree	-36	0.2207567	0.0450665	0.1027853	0.3387281
Most	Audience outdegree	-35	0.2732780	0.0682971	0.0944953	0.4520606
Most	Audience outdegree	-34	0.2854065	0.0638813	0.1181833	0.4526297
Most	Audience outdegree	-33	-0.0922653	0.0558892	-0.2385675	0.0540368
Most	Audience outdegree	-32	0.2007368	0.0376151	0.1022710	0.2992026
Most	Audience outdegree	-31	-0.0556239	0.0359900	-0.1498355	0.0385876
Most	Audience outdegree	-30	0.0409458	0.0407633	-0.0657610	0.1476526
Most	Audience outdegree	-29	-0.0589282	0.0301319	-0.1378049	0.0199485
Most	Audience outdegree	-28	-0.0939244	0.0294648	-0.1710548	-0.0167940
Most	Audience outdegree	-27	-0.0596302	0.0543457	-0.2018920	0.0826316
Most	Audience outdegree	-26	-0.0814074	0.0584297	-0.2343599	0.0715451
Most	Audience outdegree	-25	0.1317862	0.1036681	-0.1395875	0.4031600
Most	Audience outdegree	-24	-0.2096512	0.0481740	-0.3357570	-0.0835453
Most	Audience outdegree	-23	-0.0959286	0.0118800	-0.1270270	-0.0648302
Most	Audience outdegree	-22	-0.0584272	0.0270253	-0.1291717	0.0123174
Most	Audience outdegree	-21	0.0122195	0.0448237	-0.1051164	0.1295554
Most	Audience outdegree	-20	-0.1094779	0.0780983	-0.3139172	0.0949615
Most	Audience outdegree	-19	-0.0820432	0.0634040	-0.2480170	0.0839307
Most	Audience outdegree	-18	0.0170189	0.0303468	-0.0624205	0.0964582
Most	Audience outdegree	-17	-0.0627322	0.0393745	-0.1658036	0.0403392
Most	Audience outdegree	-16	-0.0184743	0.0620325	-0.1808579	0.1439093
Most	Audience outdegree	-15	0.1003707	0.0415950	-0.0085133	0.2092547
Most	Audience outdegree	-14	0.0584477	0.0304217	-0.0211876	0.1380830
Most	Audience outdegree	-13	0.0529031	0.0252707	-0.0132484	0.1190546
Most	Audience outdegree	-12	0.0432295	0.0502042	-0.0881911	0.1746500
Most	Audience outdegree	-11	-0.0732992	0.0601498	-0.2307545	0.0841560
Most	Audience outdegree	-10	0.0641663	0.0410885	-0.0433917	0.1717244
Most	Audience outdegree	-9	0.0077301	0.0721490	-0.1811356	0.1965958
Most	Audience outdegree	-8	-0.0856950	0.0424675	-0.1968629	0.0254729

Most	Audience outdegree	-7	-0.0051455	0.0421190	-0.1154011	0.1051101
Most	Audience outdegree	-6	0.0499754	0.0192996	-0.0005456	0.1004963
Most	Audience outdegree	-5	0.0433694	0.0667650	-0.1314025	0.2181414
Most	Audience outdegree	-4	-0.0832664	0.0589233	-0.2375111	0.0709783
Most	Audience outdegree	-3	-0.0477276	0.0599588	-0.2046829	0.1092276
Most	Audience outdegree	-2	0.1287551	0.0325169	0.0436351	0.2138752
Most	Audience outdegree	-1	-0.0168192	0.0458405	-0.1368166	0.1031783
Most	Audience outdegree	0	-0.1557379	0.0461691	-0.2765955	-0.0348803
Most	Audience outdegree	1	-0.2652987	0.0560245	-0.4119551	-0.1186423
Most	Audience outdegree	2	-0.2382637	0.0715889	-0.4256631	-0.0508642
Most	Audience outdegree	3	-0.2163456	0.0701873	-0.4000763	-0.0326150
Most	Audience outdegree	4	-0.1346206	0.0755217	-0.3323151	0.0630740
Most	Audience outdegree	5	-0.1727828	0.0856957	-0.3971099	0.0515443
Most	Audience outdegree	6	-0.2746161	0.0946244	-0.5223161	-0.0269160
Most	Audience outdegree	7	-0.2117922	0.0793395	-0.4194806	-0.0041038
Most	Audience outdegree	8	-0.1654229	0.0913477	-0.4045453	0.0736996
Most	Audience outdegree	9	-0.2082876	0.1128421	-0.5036764	0.0871013
Most	Audience outdegree	10	-0.2721658	0.1210431	-0.5890224	0.0446908
Most	Audience outdegree	11	-0.2525912	0.1393112	-0.6172686	0.1120862
Most	Audience outdegree	12	-0.2268520	0.1249904	-0.5540416	0.1003377
Most	Audience outdegree	13	-0.2972194	0.1350210	-0.6506664	0.0562275
Most	Audience outdegree	14	-0.2104804	0.1472066	-0.5958256	0.1748649
Most	Audience outdegree	15	0.0981109	0.1752093	-0.3605377	0.5567596
Most	Audience outdegree	16	0.0970999	0.1864527	-0.3909806	0.5851804
Most	Audience outdegree	17	0.1583478	0.2216345	-0.4218287	0.7385243
Most	Audience outdegree	18	0.2822430	0.2212461	-0.2969169	0.8614030
Most	Audience outdegree	19	0.1297965	0.2195298	-0.4448707	0.7044637
Most	Audience outdegree	20	-0.3114513	0.1652590	-0.7440528	0.1211503
Most	Audience outdegree	21	-0.2693623	0.1600848	-0.6884193	0.1496947
Most	Audience outdegree	22	-0.1748192	0.1576552	-0.5875160	0.2378776
Most	Audience outdegree	23	-0.3070124	0.1535960	-0.7090833	0.0950586
Most	Audience outdegree	24	-0.2967735	0.1877105	-0.7881467	0.1945996
Most	Audience outdegree	25	-0.3351606	0.1469817	-0.7199172	0.0495960
Most	Audience outdegree	26	-0.3143886	0.1442798	-0.6920724	0.0632952
Most	Audience outdegree	27	-0.4343825	0.0828517	-0.6512648	-0.2175002
Most	Audience outdegree	28	-0.4833575	0.1128738	-0.7788292	-0.1878858
Most	Audience outdegree	29	-0.2624736	0.1371338	-0.6214512	0.0965040
Most	Audience outdegree	30	-0.2558417	0.1472564	-0.6413174	0.1296341
Most	Audience outdegree	31	-0.4987982	0.3291899	-1.3605245	0.3629280
Most	Audience outdegree	32	-0.4731965	0.2474611	-1.1209798	0.1745869
Most	Audience outdegree	33	-0.4805028	0.1840670	-0.9623381	0.0013326

Most	Audience outdegree	34	-0.4040231	0.1679111	-0.8435669	0.0355208
Most	Audience outdegree	35	-0.3498828	0.1213127	-0.6674453	-0.0323203
Most	Audience outdegree	36	-0.3647412	0.1587185	-0.7802215	0.0507391
Most	Audience outdegree	37	-0.3996252	0.1490572	-0.7898149	-0.0094355
Most	Audience outdegree	38	-0.3637216	0.1069621	-0.6437183	-0.0837248
Most	Audience outdegree	39	-0.3759471	0.1360239	-0.7320193	-0.0198749
Most	Audience outdegree	40	-0.3437837	0.2129657	-0.9012679	0.2137005
Most	Audience outdegree	41	-0.3893738	0.1767902	-0.8521606	0.0734130
Most	Audience outdegree	42	-0.3666578	0.2629385	-1.0549567	0.3216410
Most	Audience outdegree	43	-0.9455959	0.3456394	-1.8503822	-0.0408095
Most	Audience outdegree	44	-0.8670038	0.4011008	-1.9169726	0.1829650
Most	Audience outdegree	45	-0.7810881	0.5340368	-2.1790459	0.6168697
Most	Audience outdegree	47	-0.3633104	0.0063695	-0.3799839	-0.3466369
Most	Audience outdegree	48	-0.3416126	0.0069698	-0.3598576	-0.3233675
Most	Audience outdegree	49	-0.3575156	0.0070279	-0.3759126	-0.3391185
Most	Audience outdegree	50	-0.3952934	0.0070279	-0.4136904	-0.3768963
Most	Audience outdegree	51	-0.6185800	0.3098625	-1.4297127	0.1925526
Most	Audience outdegree	52	-0.5768813	0.2967146	-1.3535964	0.1998338
Most	Audience outdegree	53	-0.5509533	0.3020069	-1.3415222	0.2396156
Most	Audience outdegree	54	-0.5617921	0.2423790	-1.1962721	0.0726878
Most	Audience outdegree	55	-0.5463242	0.3100545	-1.3579594	0.2653110
Most	Audience outdegree	56	-0.8924622	0.3942841	-1.9245868	0.1396624
Most	Audience outdegree	57	-0.8069700	0.1604520	-1.2269880	-0.3869520
Most	Audience outdegree	58	-0.9105969	0.2852645	-1.6573389	-0.1638549
Most	Audience outdegree	59	-0.9114688	0.3502314	-1.8282757	0.0053382

sample	outcome	event.time	estimate	std.error	conf.low	conf.high
Middle	Audience outdegree	-44	0.0034340	0.0403007	-0.1126345	0.1195025
Middle	Audience outdegree	-43	0.0495479	0.0251891	-0.0229983	0.1220941
Middle	Audience outdegree	-42	0.0125536	0.0182191	-0.0399186	0.0650257
Middle	Audience outdegree	-41	-0.0300232	0.0220683	-0.0935811	0.0335348
Middle	Audience outdegree	-40	0.0016707	0.0286784	-0.0809248	0.0842663
Middle	Audience outdegree	-39	0.0304581	0.0106139	-0.0001105	0.0610267
Middle	Audience outdegree	-38	0.0016150	0.0220355	-0.0618487	0.0650786
Middle	Audience outdegree	-37	-0.0620497	0.0097906	-0.0902471	-0.0338522
Middle	Audience outdegree	-36	0.0497699	0.0222179	-0.0142189	0.1137588
Middle	Audience outdegree	-35	0.2307009	0.0231850	0.1639265	0.2974753
Middle	Audience outdegree	-34	0.1829215	0.0233930	0.1155480	0.2502950
Middle	Audience outdegree	-33	0.1251996	0.0366176	0.0197386	0.2306606
Middle	Audience outdegree	-32	0.0315275	0.0158349	-0.0140780	0.0771330
Middle	Audience outdegree	-31	-0.1480363	0.0195217	-0.2042601	-0.0918125

Middle	Audience outdegree	-30	0.1459144	0.0371721	0.0388565	0.2529722
Middle	Audience outdegree	-29	-0.0164830	0.0140833	-0.0570437	0.0240777
Middle	Audience outdegree	-28	-0.0605208	0.0106551	-0.0912080	-0.0298336
Middle	Audience outdegree	-27	0.0214531	0.0474873	-0.1153133	0.1582194
Middle	Audience outdegree	-26	-0.0212454	0.0216115	-0.0834877	0.0409970
Middle	Audience outdegree	-25	-0.0184557	0.0164952	-0.0659628	0.0290514
Middle	Audience outdegree	-24	-0.0930738	0.0175049	-0.1434889	-0.0426586
Middle	Audience outdegree	-23	-0.0215394	0.0080436	-0.0447055	0.0016268
Middle	Audience outdegree	-22	0.0553035	0.0220099	-0.0080866	0.1186935
Middle	Audience outdegree	-21	-0.0778225	0.0223699	-0.1422492	-0.0133957
Middle	Audience outdegree	-20	-0.0396715	0.0247683	-0.1110058	0.0316627
Middle	Audience outdegree	-19	0.0435123	0.0433863	-0.0814429	0.1684676
Middle	Audience outdegree	-18	-0.1057212	0.0223206	-0.1700060	-0.0414364
Middle	Audience outdegree	-17	0.0603393	0.0287861	-0.0225666	0.1432452
Middle	Audience outdegree	-16	-0.0409991	0.0230183	-0.1072933	0.0252951
Middle	Audience outdegree	-15	0.0088273	0.0131945	-0.0291736	0.0468283
Middle	Audience outdegree	-14	0.0588312	0.0163694	0.0116864	0.1059760
Middle	Audience outdegree	-13	-0.0372577	0.0380566	-0.1468631	0.0723476
Middle	Audience outdegree	-12	0.0668321	0.0101256	0.0376696	0.0959945
Middle	Audience outdegree	-11	-0.0023734	0.0242688	-0.0722690	0.0675222
Middle	Audience outdegree	-10	-0.0309415	0.0337248	-0.1280712	0.0661881
Middle	Audience outdegree	-9	-0.0016128	0.0227605	-0.0671643	0.0639388
Middle	Audience outdegree	-8	0.0154194	0.0172528	-0.0342697	0.0651084
Middle	Audience outdegree	-7	-0.0408267	0.0144348	-0.0823999	0.0007465
Middle	Audience outdegree	-6	-0.0002231	0.0138743	-0.0401819	0.0397358
Middle	Audience outdegree	-5	-0.0104241	0.0207159	-0.0700873	0.0492390
Middle	Audience outdegree	-4	-0.0085813	0.0239586	-0.0775835	0.0604209
Middle	Audience outdegree	-3	-0.1145114	0.0224013	-0.1790286	-0.0499943
Middle	Audience outdegree	-2	0.0510308	0.0117270	0.0172562	0.0848055
Middle	Audience outdegree	-1	0.0841631	0.0339780	-0.0136958	0.1820220
Middle	Audience outdegree	0	-0.1234671	0.0222260	-0.1874795	-0.0594548
Middle	Audience outdegree	1	-0.1563880	0.0387955	-0.2681214	-0.0446546
Middle	Audience outdegree	2	-0.1842387	0.0453505	-0.3148511	-0.0536264
Middle	Audience outdegree	3	-0.1966503	0.0462266	-0.3297858	-0.0635147
Middle	Audience outdegree	4	-0.1591381	0.0600098	-0.3319701	0.0136939
Middle	Audience outdegree	5	-0.1484171	0.0468797	-0.2834337	-0.0134005
Middle	Audience outdegree	6	-0.2050620	0.0446526	-0.3336645	-0.0764596
Middle	Audience outdegree	7	-0.1510970	0.0527825	-0.3031141	0.0009200
Middle	Audience outdegree	8	-0.1926442	0.0490143	-0.3338086	-0.0514798
Middle	Audience outdegree	9	-0.2058352	0.0422658	-0.3275633	-0.0841070
Middle	Audience outdegree	10	-0.1525955	0.0529513	-0.3050987	-0.0000922

Middle	Audience outdegree	11	-0.1346710	0.0687293	-0.3326158	0.0632738
Middle	Audience outdegree	12	-0.1394051	0.0692949	-0.3389788	0.0601686
Middle	Audience outdegree	13	-0.2229638	0.0603087	-0.3966569	-0.0492708
Middle	Audience outdegree	14	-0.1729979	0.0653539	-0.3612213	0.0152256
Middle	Audience outdegree	15	-0.0253619	0.0542647	-0.1816476	0.1309239
Middle	Audience outdegree	16	-0.0091878	0.0769653	-0.2308527	0.2124771
Middle	Audience outdegree	17	0.0843951	0.1173292	-0.2535206	0.4223107
Middle	Audience outdegree	18	0.0870168	0.1290746	-0.2847262	0.4587598
Middle	Audience outdegree	19	-0.0358493	0.1180162	-0.3757436	0.3040449
Middle	Audience outdegree	20	-0.1886439	0.0805685	-0.4206864	0.0433986
Middle	Audience outdegree	21	-0.1717975	0.0668416	-0.3643055	0.0207105
Middle	Audience outdegree	22	-0.1856617	0.0711794	-0.3906628	0.0193393
Middle	Audience outdegree	23	-0.2089089	0.0815273	-0.4437129	0.0258950
Middle	Audience outdegree	24	-0.1202723	0.0956724	-0.3958149	0.1552703
Middle	Audience outdegree	25	-0.1989995	0.0918454	-0.4635200	0.0655209
Middle	Audience outdegree	26	-0.2382473	0.0612661	-0.4146975	-0.0617971
Middle	Audience outdegree	27	-0.2420757	0.0448211	-0.3711632	-0.1129882
Middle	Audience outdegree	28	-0.2455992	0.0580397	-0.4127573	-0.0784410
Middle	Audience outdegree	29	-0.1423015	0.0459405	-0.2746131	-0.0099898
Middle	Audience outdegree	30	-0.1911183	0.0823017	-0.4281524	0.0459158
Middle	Audience outdegree	31	-0.1669958	0.1543191	-0.6114446	0.2774530
Middle	Audience outdegree	32	-0.1890542	0.1421787	-0.5985380	0.2204296
Middle	Audience outdegree	33	-0.1617103	0.0972449	-0.4417817	0.1183612
Middle	Audience outdegree	34	-0.1495668	0.0664060	-0.3408203	0.0416867
Middle	Audience outdegree	35	-0.1351195	0.0901549	-0.3947714	0.1245325
Middle	Audience outdegree	36	-0.1402436	0.0702827	-0.3426622	0.0621750
Middle	Audience outdegree	37	-0.1454101	0.0683530	-0.3422711	0.0514508
Middle	Audience outdegree	38	-0.1234304	0.0724255	-0.3320203	0.0851596
Middle	Audience outdegree	39	-0.1378391	0.0750360	-0.3539474	0.0782693
Middle	Audience outdegree	40	-0.1661542	0.0347325	-0.2661860	-0.0661224
Middle	Audience outdegree	41	-0.1507569	0.0718890	-0.3578018	0.0562880
Middle	Audience outdegree	42	-0.1719423	0.1319192	-0.5518781	0.2079935
Middle	Audience outdegree	43	-0.4613861	0.0109963	-0.4930561	-0.4297162
Middle	Audience outdegree	44	-0.4011084	0.0698758	-0.6023551	-0.1998616
Middle	Audience outdegree	45	-0.5336634	0.0630305	-0.7151952	-0.3521315
Middle	Audience outdegree	47	-0.1940872	0.0110982	-0.2260507	-0.1621236
Middle	Audience outdegree	48	-0.1700020	0.0071688	-0.1906486	-0.1493555
Middle	Audience outdegree	49	-0.1887054	0.0122454	-0.2239731	-0.1534378
Middle	Audience outdegree	50	-0.1744928	0.0122454	-0.2097604	-0.1392252
Middle	Audience outdegree	51	-0.2613127	0.1105990	-0.5798448	0.0572193
Middle	Audience outdegree	52	-0.2399910	0.0974348	-0.5206095	0.0406275

Middle	Audience outdegree	53	-0.2217397	0.1037787	-0.5206290	0.0771495
Middle	Audience outdegree	54	-0.2212980	0.1356965	-0.6121127	0.1695166
Middle	Audience outdegree	55	-0.2537775	0.0892485	-0.5108189	0.0032639
Middle	Audience outdegree	56	-0.4845132	0.2347893	-1.1607212	0.1916948
Middle	Audience outdegree	57	-0.4884736	0.0877965	-0.7413332	-0.2356140
Middle	Audience outdegree	58	-0.4558003	0.1363511	-0.8485003	-0.0631004
Middle	Audience outdegree	59	-0.5764741	0.1420701	-0.9856449	-0.1673034

sample	outcome	event.time	estimate	std.error	conf.low	conf.high
Least	Audience outdegree	-44	-0.0329544	0.0221672	-0.0873662	0.0214575
Least	Audience outdegree	-43	0.0337181	0.0681691	-0.1336110	0.2010471
Least	Audience outdegree	-42	0.0516192	0.0204537	0.0014132	0.1018252
Least	Audience outdegree	-41	-0.0462271	0.0255230	-0.1088762	0.0164220
Least	Audience outdegree	-40	0.0375477	0.0170343	-0.0042649	0.0793604
Least	Audience outdegree	-39	-0.0260929	0.0147195	-0.0622235	0.0100378
Least	Audience outdegree	-38	-0.0120619	0.0174260	-0.0548361	0.0307123
Least	Audience outdegree	-37	0.0242531	0.0490147	-0.0960591	0.1445653
Least	Audience outdegree	-36	0.0128990	0.0302829	-0.0614338	0.0872318
Least	Audience outdegree	-35	0.0517189	0.0772320	-0.1378559	0.2412937
Least	Audience outdegree	-34	0.0528365	0.0234779	-0.0047928	0.1104659
Least	Audience outdegree	-33	0.0934369	0.0510332	-0.0318300	0.2187039
Least	Audience outdegree	-32	0.0191897	0.0268069	-0.0466111	0.0849904
Least	Audience outdegree	-31	-0.0322350	0.0121734	-0.0621160	-0.0023540
Least	Audience outdegree	-30	0.1673039	0.0321986	0.0882686	0.2463392
Least	Audience outdegree	-29	0.0312288	0.0264510	-0.0336982	0.0961559
Least	Audience outdegree	-28	-0.0070629	0.0189236	-0.0535130	0.0393872
Least	Audience outdegree	-27	0.0067926	0.0534759	-0.1244702	0.1380553
Least	Audience outdegree	-26	0.0943564	0.0406911	-0.0055245	0.1942374
Least	Audience outdegree	-25	-0.0812926	0.0133883	-0.1141558	-0.0484294
Least	Audience outdegree	-24	-0.0018218	0.0304395	-0.0765390	0.0728955
Least	Audience outdegree	-23	0.0408943	0.0381343	-0.0527108	0.1344994
Least	Audience outdegree	-22	0.0297020	0.0259433	-0.0339787	0.0933827
Least	Audience outdegree	-21	-0.0407506	0.0290903	-0.1121561	0.0306549
Least	Audience outdegree	-20	0.0214937	0.0398628	-0.0763541	0.1193416
Least	Audience outdegree	-19	-0.0049434	0.0259598	-0.0686649	0.0587780
Least	Audience outdegree	-18	-0.0854563	0.0285674	-0.1555783	-0.0153343
Least	Audience outdegree	-17	-0.0188613	0.0115622	-0.0472421	0.0095195
Least	Audience outdegree	-16	0.0033128	0.0275234	-0.0642465	0.0708721
Least	Audience outdegree	-15	0.0376301	0.0145779	0.0018470	0.0734133
Least	Audience outdegree	-14	0.0312757	0.0183200	-0.0136929	0.0762443
Least	Audience outdegree	-13	0.0011723	0.0177032	-0.0422821	0.0446268

Least	Audience outdegree	-12	-0.0471102	0.0477733	-0.1643754	0.0701549
Least	Audience outdegree	-11	0.0317042	0.0204647	-0.0185287	0.0819371
Least	Audience outdegree	-10	-0.0189848	0.0263854	-0.0837509	0.0457812
Least	Audience outdegree	-9	-0.0135871	0.0251797	-0.0753936	0.0482195
Least	Audience outdegree	-8	0.0236206	0.0376372	-0.0687641	0.1160054
Least	Audience outdegree	-7	-0.0315101	0.0208122	-0.0825961	0.0195759
Least	Audience outdegree	-6	-0.0068466	0.0293254	-0.0788292	0.0651360
Least	Audience outdegree	-5	-0.0245257	0.0172259	-0.0668088	0.0177573
Least	Audience outdegree	-4	0.0608182	0.0519233	-0.0666334	0.1882699
Least	Audience outdegree	-3	-0.0581352	0.0247532	-0.1188949	0.0026245
Least	Audience outdegree	-2	-0.0472552	0.0445847	-0.1566935	0.0621832
Least	Audience outdegree	-1	0.0009638	0.0271642	-0.0657139	0.0676415
Least	Audience outdegree	0	-0.0581891	0.0334165	-0.1402137	0.0238355
Least	Audience outdegree	1	-0.1153064	0.0546166	-0.2493693	0.0187565
Least	Audience outdegree	2	-0.1105226	0.0456901	-0.2226743	0.0016291
Least	Audience outdegree	3	-0.1011615	0.0385115	-0.1956923	-0.0066306
Least	Audience outdegree	4	-0.1370070	0.0927677	-0.3647160	0.0907019
Least	Audience outdegree	5	-0.1540200	0.0801203	-0.3506847	0.0426446
Least	Audience outdegree	6	-0.1511239	0.0638770	-0.3079174	0.0056696
Least	Audience outdegree	7	-0.1525251	0.0586228	-0.2964215	-0.0086287
Least	Audience outdegree	8	-0.1333319	0.0409790	-0.2339195	-0.0327443
Least	Audience outdegree	9	-0.1248231	0.0485905	-0.2440942	-0.0055520
Least	Audience outdegree	10	-0.2020073	0.0541210	-0.3348535	-0.0691610
Least	Audience outdegree	11	-0.1785757	0.0507319	-0.3031031	-0.0540483
Least	Audience outdegree	12	-0.1812980	0.0532704	-0.3120565	-0.0505396
Least	Audience outdegree	13	-0.2287495	0.0347877	-0.3141399	-0.1433591
Least	Audience outdegree	14	-0.2164904	0.0382632	-0.3104117	-0.1225690
Least	Audience outdegree	15	-0.0668754	0.0892307	-0.2859025	0.1521517
Least	Audience outdegree	16	-0.0892397	0.1254982	-0.3972896	0.2188102
Least	Audience outdegree	17	-0.1274751	0.1639451	-0.5298974	0.2749472
Least	Audience outdegree	18	-0.0609519	0.1924239	-0.5332786	0.4113748
Least	Audience outdegree	19	-0.1762018	0.1469428	-0.5368900	0.1844864
Least	Audience outdegree	20	-0.2444299	0.0599668	-0.3916252	-0.0972345
Least	Audience outdegree	21	-0.2889714	0.0614644	-0.4398429	-0.1380999
Least	Audience outdegree	22	-0.2913568	0.0697864	-0.4626555	-0.1200580
Least	Audience outdegree	23	-0.3049841	0.0927805	-0.5327246	-0.0772437
Least	Audience outdegree	24	-0.2978936	0.0592007	-0.4432084	-0.1525787
Least	Audience outdegree	25	-0.2797228	0.0778234	-0.4707492	-0.0886963
Least	Audience outdegree	26	-0.2970896	0.0676851	-0.4632304	-0.1309488
Least	Audience outdegree	27	-0.2847651	0.0594155	-0.4306074	-0.1389229
Least	Audience outdegree	28	-0.2644482	0.0926264	-0.4918105	-0.0370858

Least	Audience outdegree	29	-0.2016641	0.1951102	-0.6805847	0.2772564
Least	Audience outdegree	30	-0.2371571	0.2636609	-0.8843433	0.4100291
Least	Audience outdegree	31	-0.1465958	0.1774621	-0.5821970	0.2890055
Least	Audience outdegree	32	-0.1824300	0.1799058	-0.6240296	0.2591696
Least	Audience outdegree	33	-0.1837823	0.1773707	-0.6191591	0.2515945
Least	Audience outdegree	34	-0.1471350	0.1161214	-0.4321683	0.1378982
Least	Audience outdegree	35	-0.1577630	0.1136796	-0.4368029	0.1212768
Least	Audience outdegree	36	-0.1502912	0.0286783	-0.2206855	-0.0798968
Least	Audience outdegree	37	-0.1688761	0.0798423	-0.3648581	0.0271060
Least	Audience outdegree	38	-0.1478537	0.0791560	-0.3421512	0.0464438
Least	Audience outdegree	39	-0.1563697	0.0989846	-0.3993389	0.0865996
Least	Audience outdegree	40	-0.1386901	0.0825939	-0.3414263	0.0640461
Least	Audience outdegree	41	-0.1688122	0.0985987	-0.4108341	0.0732098
Least	Audience outdegree	42	-0.1506774	0.1503448	-0.5197160	0.2183612
Least	Audience outdegree	43	-0.3297710	0.3499782	-1.1888331	0.5292911
Least	Audience outdegree	44	-0.3472198	0.8398868	-2.4088192	1.7143796
Least	Audience outdegree	45	-0.3740458	0.6326044	-1.9268465	1.1787549
Least	Audience outdegree	47	-0.1823160	0.0095320	-0.2057133	-0.1589187
Least	Audience outdegree	48	-0.1677461	0.0114157	-0.1957673	-0.1397249
Least	Audience outdegree	49	-0.1648192	0.0105173	-0.1906351	-0.1390033
Least	Audience outdegree	50	-0.1834992	0.0085467	-0.2044779	-0.1625204
Least	Audience outdegree	51	-0.1959364	0.1785930	-0.6343137	0.2424409
Least	Audience outdegree	52	-0.2012335	0.1221273	-0.5010090	0.0985420
Least	Audience outdegree	53	-0.2085484	0.1908828	-0.6770923	0.2599954
Least	Audience outdegree	54	-0.1894122	0.1729898	-0.6140356	0.2352113
Least	Audience outdegree	55	-0.2126344	0.1602698	-0.6060352	0.1807664
Least	Audience outdegree	56	-0.4218120	0.4021310	-1.4088889	0.5652650
Least	Audience outdegree	57	-0.3439494	0.7314483	-2.1393739	1.4514752
Least	Audience outdegree	58	-0.4569265	0.7636623	-2.3314240	1.4175711
Least	Audience outdegree	59	-0.3936762	0.8112010	-2.3848628	1.5975105

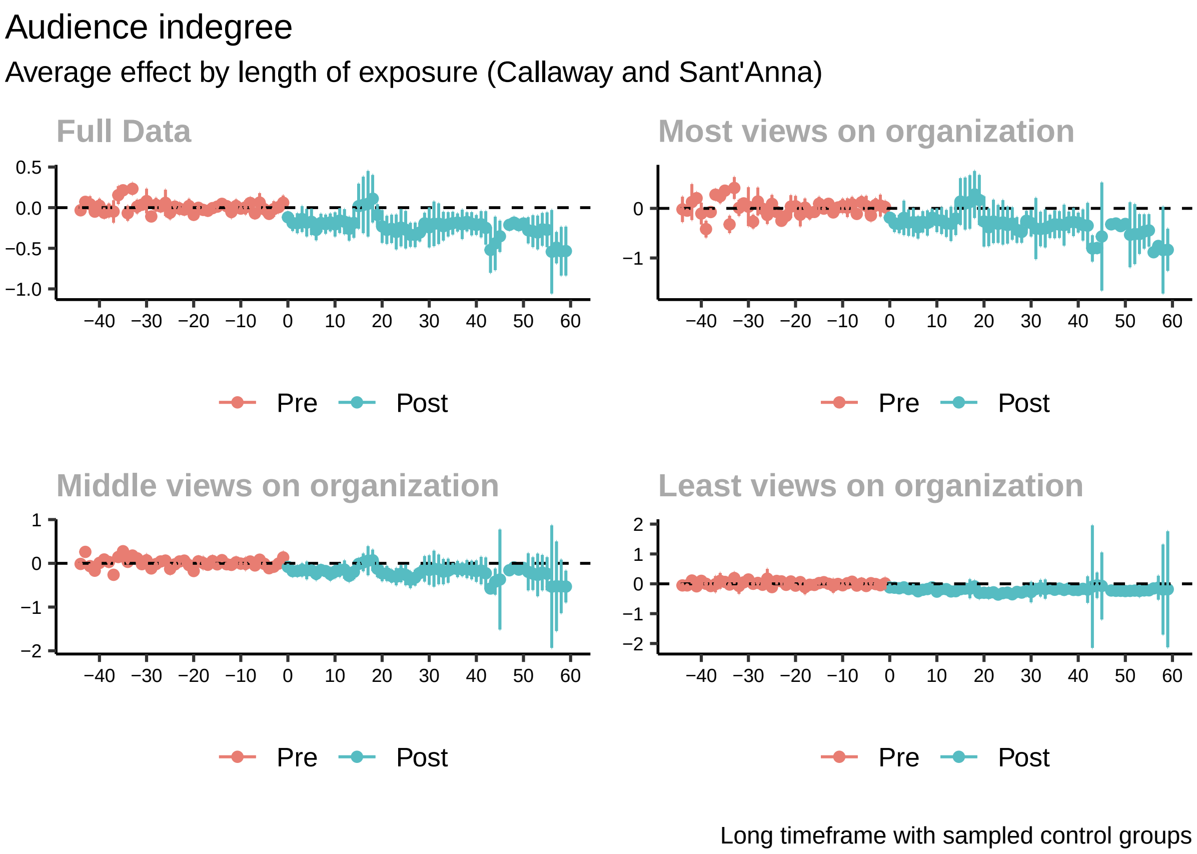

sample	outcome	event.time	estimate	std.error	conf.low	conf.high
Full	Audience indegree	-44	-0.0324798	0.0215034	-0.0881118	0.0231521
Full	Audience indegree	-43	0.0716486	0.0083079	0.0501552	0.0931421
Full	Audience indegree	-42	0.0423734	0.0378072	-0.0554384	0.1401852
Full	Audience indegree	-41	-0.0491086	0.0138367	-0.0849058	-0.0133114
Full	Audience indegree	-40	0.0236452	0.0334994	-0.0630217	0.1103121
Full	Audience indegree	-39	-0.0632950	0.0258717	-0.1302281	0.0036381
Full	Audience indegree	-38	-0.0353906	0.0335366	-0.1221537	0.0513726
Full	Audience indegree	-37	-0.0475422	0.0532245	-0.1852405	0.0901560
Full	Audience indegree	-36	0.1544341	0.0428385	0.0436058	0.2652624
Full	Audience indegree	-35	0.2139094	0.0089992	0.1906274	0.2371913
Full	Audience indegree	-34	-0.0681966	0.0359422	-0.1611832	0.0247901
Full	Audience indegree	-33	0.2320958	0.0282076	0.1591194	0.3050723
Full	Audience indegree	-32	0.0124474	0.0333433	-0.0738158	0.0987106
Full	Audience indegree	-31	0.0358306	0.0151278	-0.0033068	0.0749681
Full	Audience indegree	-30	0.0812175	0.0582499	-0.0694820	0.2319169
Full	Audience indegree	-29	-0.1085703	0.0218910	-0.1652049	-0.0519356
Full	Audience indegree	-28	0.0351700	0.0328244	-0.0497506	0.1200905
Full	Audience indegree	-27	0.0147432	0.0259449	-0.0523793	0.0818657
Full	Audience indegree	-26	0.0589859	0.0624173	-0.1024952	0.2204670

Full	Audience indegree	-25	-0.0624964	0.0314750	-0.1439260	0.0189332
Full	Audience indegree	-24	0.0122259	0.0284279	-0.0613205	0.0857724
Full	Audience indegree	-23	-0.0112699	0.0299606	-0.0887815	0.0662417
Full	Audience indegree	-22	-0.0246214	0.0251132	-0.0895923	0.0403495
Full	Audience indegree	-21	0.0206589	0.0340862	-0.0675263	0.1088440
Full	Audience indegree	-20	-0.0887990	0.0219557	-0.1456011	-0.0319969
Full	Audience indegree	-19	-0.0015556	0.0223540	-0.0593880	0.0562768
Full	Audience indegree	-18	-0.0273295	0.0220470	-0.0843678	0.0297088
Full	Audience indegree	-17	-0.0384657	0.0232397	-0.0985897	0.0216583
Full	Audience indegree	-16	-0.0056012	0.0140263	-0.0418889	0.0306864
Full	Audience indegree	-15	0.0125425	0.0190837	-0.0368294	0.0619143
Full	Audience indegree	-14	0.0469261	0.0076082	0.0272429	0.0666093
Full	Audience indegree	-13	0.0186623	0.0173859	-0.0263172	0.0636418
Full	Audience indegree	-12	-0.0538473	0.0294533	-0.1300466	0.0223520
Full	Audience indegree	-11	0.0206333	0.0313942	-0.0605874	0.1018539
Full	Audience indegree	-10	-0.0114412	0.0203941	-0.0642033	0.0413208
Full	Audience indegree	-9	0.0040622	0.0343051	-0.0846892	0.0928137
Full	Audience indegree	-8	0.0581380	0.0267368	-0.0110333	0.1273094
Full	Audience indegree	-7	-0.0702532	0.0243635	-0.1332845	-0.0072219
Full	Audience indegree	-6	0.0626174	0.0445271	-0.0525796	0.1778144
Full	Audience indegree	-5	-0.0197625	0.0323290	-0.1034016	0.0638765
Full	Audience indegree	-4	-0.0766300	0.0207005	-0.1301847	-0.0230754
Full	Audience indegree	-3	-0.0171233	0.0371514	-0.1132384	0.0789919
Full	Audience indegree	-2	0.0054733	0.0274613	-0.0655725	0.0765190
Full	Audience indegree	-1	0.0607836	0.0341876	-0.0276639	0.1492311
Full	Audience indegree	0	-0.1182874	0.0256523	-0.1846530	-0.0519217
Full	Audience indegree	1	-0.1892322	0.0376177	-0.2865536	-0.0919107
Full	Audience indegree	2	-0.1920021	0.0474017	-0.3146360	-0.0693683
Full	Audience indegree	3	-0.1400836	0.0613947	-0.2989190	0.0187519
Full	Audience indegree	4	-0.1844171	0.0655162	-0.3539154	-0.0149187
Full	Audience indegree	5	-0.1739802	0.0626033	-0.3359424	-0.0120181
Full	Audience indegree	6	-0.2742395	0.0489720	-0.4009360	-0.1475429
Full	Audience indegree	7	-0.1886257	0.0438144	-0.3019788	-0.0752725
Full	Audience indegree	8	-0.2032833	0.0455505	-0.3211278	-0.0854388
Full	Audience indegree	9	-0.1817816	0.0423674	-0.2913911	-0.0721722
Full	Audience indegree	10	-0.2064078	0.0573816	-0.3548608	-0.0579549
Full	Audience indegree	11	-0.1589210	0.0590136	-0.3115962	-0.0062458
Full	Audience indegree	12	-0.1729824	0.0595525	-0.3270518	-0.0189129
Full	Audience indegree	13	-0.2601435	0.0565303	-0.4063941	-0.1138929
Full	Audience indegree	14	-0.1883072	0.0705668	-0.3708719	-0.0057425
Full	Audience indegree	15	0.0179019	0.1070266	-0.2589888	0.2947926

Full	Audience indegree	16	0.0330656	0.1342778	-0.3143272	0.3804584
Full	Audience indegree	17	0.0476413	0.1561006	-0.3562096	0.4514922
Full	Audience indegree	18	0.1102219	0.1128205	-0.1816583	0.4021022
Full	Audience indegree	19	-0.0894817	0.0505789	-0.2203354	0.0413720
Full	Audience indegree	20	-0.2314152	0.0779545	-0.4330930	-0.0297375
Full	Audience indegree	21	-0.2735102	0.0667174	-0.4461161	-0.1009042
Full	Audience indegree	22	-0.2607702	0.0674719	-0.4353282	-0.0862122
Full	Audience indegree	23	-0.2989746	0.0827244	-0.5129925	-0.0849568
Full	Audience indegree	24	-0.2458176	0.0888095	-0.4755786	-0.0160567
Full	Audience indegree	25	-0.2711985	0.0890794	-0.5016576	-0.0407394
Full	Audience indegree	26	-0.3338755	0.0571546	-0.4817412	-0.1860097
Full	Audience indegree	27	-0.3350226	0.0586283	-0.4867010	-0.1833442
Full	Audience indegree	28	-0.3064144	0.0428241	-0.4172055	-0.1956234
Full	Audience indegree	29	-0.1939035	0.0504972	-0.3245457	-0.0632612
Full	Audience indegree	30	-0.2421885	0.0954035	-0.4890089	0.0046318
Full	Audience indegree	31	-0.1991530	0.1060532	-0.4735254	0.0752193
Full	Audience indegree	32	-0.1974534	0.0963923	-0.4468318	0.0519250
Full	Audience indegree	33	-0.2286334	0.0682662	-0.4052461	-0.0520206
Full	Audience indegree	34	-0.2052840	0.0579007	-0.3550801	-0.0554878
Full	Audience indegree	35	-0.1910387	0.0540134	-0.3307779	-0.0512994
Full	Audience indegree	36	-0.1835103	0.0431763	-0.2952126	-0.0718081
Full	Audience indegree	37	-0.2049696	0.0697511	-0.3854241	-0.0245151
Full	Audience indegree	38	-0.1747507	0.0453029	-0.2919548	-0.0575466
Full	Audience indegree	39	-0.1977040	0.0550571	-0.3401434	-0.0552646
Full	Audience indegree	40	-0.2126378	0.0472333	-0.3348361	-0.0904395
Full	Audience indegree	41	-0.2039438	0.0623255	-0.3651874	-0.0427002
Full	Audience indegree	42	-0.2493852	0.0792905	-0.4545193	-0.0442510
Full	Audience indegree	43	-0.5203119	0.1086746	-0.8014661	-0.2391576
Full	Audience indegree	44	-0.4402443	0.1270344	-0.7688976	-0.1115910
Full	Audience indegree	45	-0.3524826	0.0744019	-0.5449692	-0.1599959
Full	Audience indegree	47	-0.2119884	0.0249156	-0.2764480	-0.1475289
Full	Audience indegree	48	-0.1845352	0.0272640	-0.2550704	-0.1140000
Full	Audience indegree	49	-0.2153355	0.0274911	-0.2864582	-0.1442128
Full	Audience indegree	50	-0.1950385	0.0274911	-0.2661612	-0.1239158
Full	Audience indegree	51	-0.2783624	0.0620997	-0.4390218	-0.1177030
Full	Audience indegree	52	-0.2897206	0.0756634	-0.4854708	-0.0939703
Full	Audience indegree	53	-0.3054690	0.0802454	-0.5130735	-0.0978645
Full	Audience indegree	54	-0.2673402	0.0779302	-0.4689550	-0.0657255
Full	Audience indegree	55	-0.2726692	0.0825718	-0.4862923	-0.0590460
Full	Audience indegree	56	-0.5427271	0.1985334	-1.0563569	-0.0290973
Full	Audience indegree	57	-0.4922552	0.0744917	-0.6849741	-0.2995363

Full	Audience indegree	58	-0.5365720	0.1154945	-0.8353700	-0.2377740
Full	Audience indegree	59	-0.5341611	0.1162148	-0.8348229	-0.2334994

sample	outcome	event.time	estimate	std.error	conf.low	conf.high
Most	Audience indegree	-44	-0.0198376	0.1004787	-0.2743222	0.2346471
Most	Audience indegree	-43	-0.0456996	0.0166221	-0.0877988	-0.0036004
Most	Audience indegree	-42	0.1283435	0.1427994	-0.2333276	0.4900147
Most	Audience indegree	-41	0.2053995	0.0468688	0.0866938	0.3241052
Most	Audience indegree	-40	-0.1042518	0.0863809	-0.3230306	0.1145271
Most	Audience indegree	-39	-0.4177036	0.0668629	-0.5870489	-0.2483584
Most	Audience indegree	-38	-0.0738060	0.0304664	-0.1509689	0.0033570
Most	Audience indegree	-37	0.2767905	0.0435109	0.1665895	0.3869914
Most	Audience indegree	-36	0.2445104	0.0556033	0.1036827	0.3853381
Most	Audience indegree	-35	0.3575681	0.0336119	0.2724384	0.4426978
Most	Audience indegree	-34	-0.3236738	0.0688078	-0.4979448	-0.1494029
Most	Audience indegree	-33	0.4116313	0.0869547	0.1913992	0.6318633
Most	Audience indegree	-32	0.0103939	0.0635222	-0.1504902	0.1712780
Most	Audience indegree	-31	0.1008803	0.0297152	0.0256199	0.1761408
Most	Audience indegree	-30	0.0333651	0.1544784	-0.3578856	0.4246158
Most	Audience indegree	-29	-0.2670053	0.0552659	-0.4069784	-0.1270322
Most	Audience indegree	-28	0.1388498	0.1110424	-0.1423897	0.4200892
Most	Audience indegree	-27	-0.0070565	0.0361480	-0.0986092	0.0844963
Most	Audience indegree	-26	-0.1327648	0.0712682	-0.3132674	0.0477378
Most	Audience indegree	-25	0.0904170	0.0711823	-0.0898679	0.2707019
Most	Audience indegree	-24	-0.0720039	0.0144015	-0.1084788	-0.0355290
Most	Audience indegree	-23	-0.2508696	0.0214702	-0.3052476	-0.1964917
Most	Audience indegree	-22	-0.1521743	0.0549087	-0.2912428	-0.0131058
Most	Audience indegree	-21	0.0359176	0.0905699	-0.1934708	0.2653060
Most	Audience indegree	-20	0.0431560	0.0823611	-0.1654419	0.2517539
Most	Audience indegree	-19	-0.1282408	0.0922308	-0.3618358	0.1053543
Most	Audience indegree	-18	0.0152715	0.0726069	-0.1686216	0.1991646
Most	Audience indegree	-17	-0.0842053	0.0557244	-0.2253397	0.0569290
Most	Audience indegree	-16	-0.0723532	0.0335525	-0.1573325	0.0126260
Most	Audience indegree	-15	0.0940594	0.0544963	-0.0439645	0.2320833
Most	Audience indegree	-14	-0.0052421	0.0223910	-0.0619523	0.0514682
Most	Audience indegree	-13	0.0924518	0.0327219	0.0095763	0.1753272
Most	Audience indegree	-12	-0.0820784	0.0325166	-0.1644340	0.0002772
Most	Audience indegree	-11	0.0176407	0.0372762	-0.0767695	0.1120508
Most	Audience indegree	-10	0.0503409	0.0581416	-0.0969156	0.1975973
Most	Audience indegree	-9	0.0146537	0.0743888	-0.1737523	0.2030598
Most	Audience indegree	-8	0.0842183	0.0581945	-0.0631722	0.2316087

Most	Audience indegree	-7	-0.1115425	0.0357893	-0.2021867	-0.0208982
Most	Audience indegree	-6	0.1221484	0.0516993	-0.0087916	0.2530885
Most	Audience indegree	-5	0.0501263	0.0798252	-0.1520487	0.2523012
Most	Audience indegree	-4	-0.1434927	0.0475276	-0.2638667	-0.0231186
Most	Audience indegree	-3	0.0688481	0.0545683	-0.0693581	0.2070544
Most	Audience indegree	-2	0.0566043	0.0871833	-0.1642068	0.2774153
Most	Audience indegree	-1	0.0269392	0.0500571	-0.0998416	0.1537199
Most	Audience indegree	0	-0.1877036	0.0374876	-0.2826493	-0.0927579
Most	Audience indegree	1	-0.3016741	0.0696332	-0.4780357	-0.1253125
Most	Audience indegree	2	-0.3159395	0.0731009	-0.5010839	-0.1307951
Most	Audience indegree	3	-0.1907735	0.1369582	-0.5376505	0.1561036
Most	Audience indegree	4	-0.2874998	0.1048448	-0.5530424	-0.0219572
Most	Audience indegree	5	-0.2772932	0.1138631	-0.5656767	0.0110902
Most	Audience indegree	6	-0.3813091	0.0900593	-0.6094043	-0.1532139
Most	Audience indegree	7	-0.2717154	0.0848152	-0.4865287	-0.0569020
Most	Audience indegree	8	-0.2954697	0.1001905	-0.5492244	-0.0417151
Most	Audience indegree	9	-0.1883225	0.0730054	-0.3732249	-0.0034200
Most	Audience indegree	10	-0.2370824	0.0951379	-0.4780402	0.0038755
Most	Audience indegree	11	-0.2461933	0.1066500	-0.5163082	0.0239216
Most	Audience indegree	12	-0.3031728	0.1070588	-0.5743230	-0.0320226
Most	Audience indegree	13	-0.3123875	0.1361823	-0.6572994	0.0325243
Most	Audience indegree	14	-0.2053290	0.1315060	-0.5383972	0.1277391
Most	Audience indegree	15	0.1377660	0.1855665	-0.3322222	0.6077543
Most	Audience indegree	16	0.0969084	0.2073177	-0.4281696	0.6219864
Most	Audience indegree	17	0.1290130	0.2129044	-0.4102146	0.6682406
Most	Audience indegree	18	0.2809332	0.1869372	-0.1925267	0.7543930
Most	Audience indegree	19	0.1695829	0.1990697	-0.3346053	0.6737712
Most	Audience indegree	20	-0.2641083	0.1997788	-0.7700924	0.2418758
Most	Audience indegree	21	-0.3818657	0.1502526	-0.7624138	-0.0013177
Most	Audience indegree	22	-0.2646548	0.1725295	-0.7016241	0.1723144
Most	Audience indegree	23	-0.3122681	0.1508142	-0.6942386	0.0697024
Most	Audience indegree	24	-0.2825256	0.1772082	-0.7313447	0.1662935
Most	Audience indegree	25	-0.3363985	0.1473146	-0.7095054	0.0367084
Most	Audience indegree	26	-0.3052742	0.1289617	-0.6318984	0.0213501
Most	Audience indegree	27	-0.4350646	0.1024408	-0.6945186	-0.1756107
Most	Audience indegree	28	-0.4744898	0.0864875	-0.6935386	-0.2554411
Most	Audience indegree	29	-0.2496320	0.0784091	-0.4482204	-0.0510436
Most	Audience indegree	30	-0.2957804	0.1070184	-0.5668281	-0.0247327
Most	Audience indegree	31	-0.4073751	0.2435026	-1.0240994	0.2093493
Most	Audience indegree	32	-0.4161748	0.1370443	-0.7632699	-0.0690797
Most	Audience indegree	33	-0.4046309	0.1512936	-0.7878157	-0.0214462

Most	Audience indegree	34	-0.3629831	0.0919780	-0.5959378	-0.1300283
Most	Audience indegree	35	-0.3215881	0.1107152	-0.6019991	-0.0411772
Most	Audience indegree	36	-0.3257404	0.1060054	-0.5942227	-0.0572581
Most	Audience indegree	37	-0.3379377	0.1626241	-0.7498193	0.0739438
Most	Audience indegree	38	-0.2811317	0.0856549	-0.4980718	-0.0641916
Most	Audience indegree	39	-0.2734745	0.1122876	-0.5578678	0.0109188
Most	Audience indegree	40	-0.2869395	0.0795194	-0.4883399	-0.0855390
Most	Audience indegree	41	-0.3350786	0.1238201	-0.6486806	-0.0214767
Most	Audience indegree	42	-0.3483912	0.1812093	-0.8073440	0.1105616
Most	Audience indegree	43	-0.8082902	0.1047789	-1.0736659	-0.5429144
Most	Audience indegree	44	-0.7996463	0.0410865	-0.9037070	-0.6955856
Most	Audience indegree	45	-0.5673575	0.4301281	-1.6567524	0.5220373
Most	Audience indegree	47	-0.3218289	0.0063695	-0.3379611	-0.3056968
Most	Audience indegree	48	-0.3053163	0.0091670	-0.3285337	-0.2820989
Most	Audience indegree	49	-0.3575156	0.0070279	-0.3753153	-0.3397159
Most	Audience indegree	50	-0.3138119	0.0070279	-0.3316116	-0.2960122
Most	Audience indegree	51	-0.5323406	0.2585615	-1.1872051	0.1225239
Most	Audience indegree	52	-0.5184459	0.2387631	-1.1231664	0.0862747
Most	Audience indegree	53	-0.5170231	0.1580309	-0.9172714	-0.1167747
Most	Audience indegree	54	-0.4661277	0.1366682	-0.8122701	-0.1199852
Most	Audience indegree	55	-0.4440622	0.1293228	-0.7716009	-0.1165234
Most	Audience indegree	56	-0.8846902	0.0039464	-0.8946854	-0.8746949
Most	Audience indegree	57	-0.7577472	0.0103488	-0.7839579	-0.7315364
Most	Audience indegree	58	-0.8406487	0.3457483	-1.7163330	0.0350356
Most	Audience indegree	59	-0.8350439	0.1673266	-1.2588357	-0.4112521

sample	outcome	event.time	estimate	std.error	conf.low	conf.high
Middle	Audience indegree	-44	-0.0137921	0.0200134	-0.0652725	0.0376884
Middle	Audience indegree	-43	0.2616608	0.0167800	0.2184976	0.3048239
Middle	Audience indegree	-42	-0.0646723	0.0241559	-0.1268084	-0.0025362
Middle	Audience indegree	-41	-0.1685011	0.0377457	-0.2655942	-0.0714080
Middle	Audience indegree	-40	0.0092986	0.0462567	-0.1096872	0.1282844
Middle	Audience indegree	-39	0.0884314	0.0108335	0.0605644	0.1162984
Middle	Audience indegree	-38	0.0317196	0.0431750	-0.0793391	0.1427784
Middle	Audience indegree	-37	-0.2625574	0.0343010	-0.3507897	-0.1743252
Middle	Audience indegree	-36	0.1449072	0.0369864	0.0497672	0.2400471
Middle	Audience indegree	-35	0.2769097	0.0312422	0.1965457	0.3572738
Middle	Audience indegree	-34	0.0338461	0.0239859	-0.0278528	0.0955450
Middle	Audience indegree	-33	0.1780249	0.0299110	0.1010848	0.2549649
Middle	Audience indegree	-32	0.1141078	0.0371232	0.0186161	0.2095995
Middle	Audience indegree	-31	-0.0195163	0.0222874	-0.0768461	0.0378135

Middle	Audience indegree	-30	0.0679256	0.0549683	-0.0734690	0.2093203
Middle	Audience indegree	-29	-0.1166892	0.0313316	-0.1972833	-0.0360951
Middle	Audience indegree	-28	-0.0168516	0.0183787	-0.0641271	0.0304238
Middle	Audience indegree	-27	0.0427156	0.0364556	-0.0510590	0.1364902
Middle	Audience indegree	-26	0.0627644	0.0413129	-0.0435046	0.1690335
Middle	Audience indegree	-25	-0.1280220	0.0467282	-0.2482208	-0.0078231
Middle	Audience indegree	-24	-0.0236156	0.0300446	-0.1008992	0.0536681
Middle	Audience indegree	-23	0.0428158	0.0402548	-0.0607315	0.1463632
Middle	Audience indegree	-22	0.0619900	0.0372386	-0.0337986	0.1577786
Middle	Audience indegree	-21	-0.0414755	0.0580452	-0.1907848	0.1078337
Middle	Audience indegree	-20	-0.1758662	0.0341015	-0.2635852	-0.0881471
Middle	Audience indegree	-19	0.0455578	0.0430553	-0.0651932	0.1563089
Middle	Audience indegree	-18	0.0074536	0.0575715	-0.1406373	0.1555444
Middle	Audience indegree	-17	-0.0331188	0.0241732	-0.0952995	0.0290619
Middle	Audience indegree	-16	0.0530325	0.0476040	-0.0694189	0.1754840
Middle	Audience indegree	-15	-0.0238221	0.0191895	-0.0731832	0.0255390
Middle	Audience indegree	-14	0.0727884	0.0190988	0.0236606	0.1219161
Middle	Audience indegree	-13	-0.0208885	0.0186113	-0.0687622	0.0269852
Middle	Audience indegree	-12	-0.0354039	0.0399185	-0.1380860	0.0672782
Middle	Audience indegree	-11	0.0230304	0.0476604	-0.0995661	0.1456269
Middle	Audience indegree	-10	-0.0070393	0.0445915	-0.1217417	0.1076631
Middle	Audience indegree	-9	-0.0109666	0.0570019	-0.1575922	0.1356590
Middle	Audience indegree	-8	0.0415063	0.0141900	0.0050053	0.0780072
Middle	Audience indegree	-7	-0.0486972	0.0336421	-0.1352345	0.0378401
Middle	Audience indegree	-6	0.0832942	0.0406249	-0.0212050	0.1877935
Middle	Audience indegree	-5	-0.0122724	0.0306762	-0.0911806	0.0666357
Middle	Audience indegree	-4	-0.1121159	0.0242694	-0.1745440	-0.0496877
Middle	Audience indegree	-3	-0.0773615	0.0455351	-0.1944913	0.0397683
Middle	Audience indegree	-2	-0.0065090	0.0303633	-0.0846123	0.0715944
Middle	Audience indegree	-1	0.1343797	0.0515927	0.0016681	0.2670912
Middle	Audience indegree	0	-0.0813348	0.0295385	-0.1573165	-0.0053531
Middle	Audience indegree	1	-0.1784768	0.0487485	-0.3038724	-0.0530812
Middle	Audience indegree	2	-0.1767095	0.0485834	-0.3016803	-0.0517386
Middle	Audience indegree	3	-0.1512735	0.0595938	-0.3045662	0.0020193
Middle	Audience indegree	4	-0.1624586	0.0782655	-0.3637806	0.0388635
Middle	Audience indegree	5	-0.1610253	0.0531052	-0.2976276	-0.0244230
Middle	Audience indegree	6	-0.2392369	0.0571174	-0.3861597	-0.0923141
Middle	Audience indegree	7	-0.1522110	0.0458017	-0.2700266	-0.0343955
Middle	Audience indegree	8	-0.1771964	0.0402374	-0.2806989	-0.0736940
Middle	Audience indegree	9	-0.2472760	0.0587052	-0.3982831	-0.0962688
Middle	Audience indegree	10	-0.1895101	0.0586715	-0.3404305	-0.0385897

Middle	Audience indegree	11	-0.1531721	0.0618687	-0.3123167	0.0059725
Middle	Audience indegree	12	-0.1435969	0.0815862	-0.3534607	0.0662668
Middle	Audience indegree	13	-0.2858463	0.0542700	-0.4254447	-0.1462480
Middle	Audience indegree	14	-0.1964702	0.0657648	-0.3656366	-0.0273038
Middle	Audience indegree	15	-0.0139935	0.0509378	-0.1450205	0.1170335
Middle	Audience indegree	16	0.0211997	0.0787629	-0.1814017	0.2238012
Middle	Audience indegree	17	0.0597755	0.1290221	-0.2721074	0.3916585
Middle	Audience indegree	18	0.0694080	0.0943788	-0.1733622	0.3121783
Middle	Audience indegree	19	-0.1249871	0.0740819	-0.3155476	0.0655735
Middle	Audience indegree	20	-0.2293516	0.0700925	-0.4096503	-0.0490529
Middle	Audience indegree	21	-0.2319483	0.0671675	-0.4047228	-0.0591738
Middle	Audience indegree	22	-0.2916457	0.0593254	-0.4442482	-0.1390432
Middle	Audience indegree	23	-0.3052626	0.0765182	-0.5020901	-0.1084351
Middle	Audience indegree	24	-0.2372036	0.0761975	-0.4332061	-0.0412012
Middle	Audience indegree	25	-0.2653302	0.0914878	-0.5006639	-0.0299965
Middle	Audience indegree	26	-0.3736499	0.0748310	-0.5661373	-0.1811625
Middle	Audience indegree	27	-0.3281261	0.0708206	-0.5102976	-0.1459546
Middle	Audience indegree	28	-0.2215962	0.0528557	-0.3575567	-0.0856358
Middle	Audience indegree	29	-0.1260031	0.1130094	-0.4166966	0.1646903
Middle	Audience indegree	30	-0.1525305	0.1309744	-0.4894353	0.1843744
Middle	Audience indegree	31	-0.1228262	0.1599137	-0.5341713	0.2885189
Middle	Audience indegree	32	-0.1384800	0.1316443	-0.4771080	0.2001480
Middle	Audience indegree	33	-0.1904205	0.1127723	-0.4805041	0.0996631
Middle	Audience indegree	34	-0.1690961	0.1067541	-0.4436990	0.1055068
Middle	Audience indegree	35	-0.1183062	0.0493327	-0.2452046	0.0085921
Middle	Audience indegree	36	-0.1302850	0.0718279	-0.3150474	0.0544774
Middle	Audience indegree	37	-0.1376502	0.0612804	-0.2952814	0.0199810
Middle	Audience indegree	38	-0.1313197	0.0798479	-0.3367120	0.0740727
Middle	Audience indegree	39	-0.1536176	0.0828744	-0.3667952	0.0595599
Middle	Audience indegree	40	-0.1786995	0.0956543	-0.4247506	0.0673516
Middle	Audience indegree	41	-0.1652421	0.1220768	-0.4792596	0.1487753
Middle	Audience indegree	42	-0.2279461	0.1405297	-0.5894299	0.1335377
Middle	Audience indegree	43	-0.5762376	0.0383686	-0.6749329	-0.4775424
Middle	Audience indegree	44	-0.4189301	0.1156460	-0.7164056	-0.1214546
Middle	Audience indegree	45	-0.3702970	0.4442141	-1.5129468	0.7723527
Middle	Audience indegree	47	-0.1588399	0.0110982	-0.1873877	-0.1302920
Middle	Audience indegree	48	-0.0926854	0.0159726	-0.1337715	-0.0515992
Middle	Audience indegree	49	-0.1440778	0.0122454	-0.1755767	-0.1125789
Middle	Audience indegree	50	-0.1054195	0.0122454	-0.1369183	-0.0739206
Middle	Audience indegree	51	-0.1948375	0.1648693	-0.6189300	0.2292550
Middle	Audience indegree	52	-0.2355740	0.1457308	-0.6104366	0.1392886

Middle	Audience indegree	53	-0.2650259	0.1891541	-0.7515860	0.2215341
Middle	Audience indegree	54	-0.2137892	0.1574449	-0.6187839	0.1912055
Middle	Audience indegree	55	-0.2389807	0.1475173	-0.6184386	0.1404772
Middle	Audience indegree	56	-0.5330280	0.5444852	-1.9336046	0.8675485
Middle	Audience indegree	57	-0.5241172	0.3977320	-1.5472011	0.4989668
Middle	Audience indegree	58	-0.5280776	0.2374402	-1.1388438	0.0826887
Middle	Audience indegree	59	-0.5319197	0.1409872	-0.8945804	-0.1692589

sample	outcome	event.time	estimate	std.error	conf.low	conf.high
Least	Audience indegree	-44	-0.0538819	0.0361801	-0.1448014	0.0370376
Least	Audience indegree	-43	-0.0536025	0.0194688	-0.1025271	-0.0046779
Least	Audience indegree	-42	0.1104604	0.0430875	0.0021828	0.2187380
Least	Audience indegree	-41	-0.0842886	0.0265482	-0.1510034	-0.0175739
Least	Audience indegree	-40	0.0997200	0.0307669	0.0224038	0.1770363
Least	Audience indegree	-39	0.0014288	0.0336958	-0.0832477	0.0861053
Least	Audience indegree	-38	-0.0745267	0.0595876	-0.2242686	0.0752151
Least	Audience indegree	-37	-0.0127030	0.1073542	-0.2824807	0.2570747
Least	Audience indegree	-36	0.0978594	0.1047778	-0.1654439	0.3611627
Least	Audience indegree	-35	0.0623048	0.0355348	-0.0269930	0.1516027
Least	Audience indegree	-34	-0.0252129	0.0271274	-0.0933833	0.0429575
Least	Audience indegree	-33	0.1916907	0.0787808	-0.0062830	0.3896644
Least	Audience indegree	-32	-0.0731526	0.1028812	-0.3316899	0.1853846
Least	Audience indegree	-31	0.0483825	0.0108634	0.0210832	0.0756819
Least	Audience indegree	-30	0.1452770	0.0256158	0.0809052	0.2096487
Least	Audience indegree	-29	0.0001041	0.0133262	-0.0333843	0.0335926
Least	Audience indegree	-28	0.0300547	0.0577504	-0.1150703	0.1751797
Least	Audience indegree	-27	-0.0316822	0.0480107	-0.1523315	0.0889672
Least	Audience indegree	-26	0.1722973	0.1294709	-0.1530589	0.4976536
Least	Audience indegree	-25	-0.1107461	0.0275781	-0.1800491	-0.0414431
Least	Audience indegree	-24	0.0956404	0.0490100	-0.0275201	0.2188008
Least	Audience indegree	-23	0.0813908	0.0516799	-0.0484792	0.2112607
Least	Audience indegree	-22	-0.0338464	0.0436068	-0.1434290	0.0757361
Least	Audience indegree	-21	0.0775133	0.0246359	0.0156040	0.1394226
Least	Audience indegree	-20	-0.0615718	0.0149310	-0.0990930	-0.0240505
Least	Audience indegree	-19	0.0569429	0.0438769	-0.0533184	0.1672043
Least	Audience indegree	-18	-0.1126744	0.0936511	-0.3480167	0.1226680
Least	Audience indegree	-17	-0.0306899	0.0166075	-0.0724241	0.0110443
Least	Audience indegree	-16	-0.0370988	0.0269203	-0.1047487	0.0305511
Least	Audience indegree	-15	0.0252466	0.0228182	-0.0320949	0.0825881
Least	Audience indegree	-14	0.0540535	0.0388578	-0.0435949	0.1517019
Least	Audience indegree	-13	0.0017395	0.0418073	-0.1033208	0.1067998

Least	Audience indegree	-12	-0.0646472	0.0941288	-0.3011899	0.1718954
Least	Audience indegree	-11	-0.0055716	0.0265727	-0.0723481	0.0612048
Least	Audience indegree	-10	-0.0493074	0.0419006	-0.1546024	0.0559876
Least	Audience indegree	-9	0.0189739	0.0514608	-0.1103454	0.1482931
Least	Audience indegree	-8	0.0697442	0.0387164	-0.0275488	0.1670373
Least	Audience indegree	-7	-0.0597026	0.0450174	-0.1728299	0.0534248
Least	Audience indegree	-6	0.0103905	0.0698172	-0.1650579	0.1858389
Least	Audience indegree	-5	-0.0735722	0.0530057	-0.2067739	0.0596295
Least	Audience indegree	-4	0.0207163	0.0551383	-0.1178445	0.1592771
Least	Audience indegree	-3	-0.0089046	0.0194801	-0.0578575	0.0400482
Least	Audience indegree	-2	-0.0480618	0.0554925	-0.1875128	0.0913891
Least	Audience indegree	-1	0.0138831	0.0787818	-0.1840930	0.2118592
Least	Audience indegree	0	-0.1211202	0.0533521	-0.2551922	0.0129518
Least	Audience indegree	1	-0.1301494	0.0377624	-0.2250452	-0.0352537
Least	Audience indegree	2	-0.1496915	0.0537295	-0.2847122	-0.0146708
Least	Audience indegree	3	-0.1185585	0.0514215	-0.2477791	0.0106621
Least	Audience indegree	4	-0.1763072	0.0535315	-0.3108302	-0.0417842
Least	Audience indegree	5	-0.1618967	0.0650709	-0.3254178	0.0016244
Least	Audience indegree	6	-0.2508443	0.0550428	-0.3891651	-0.1125236
Least	Audience indegree	7	-0.1998665	0.0472771	-0.3186725	-0.0810605
Least	Audience indegree	8	-0.1762472	0.0600521	-0.3271563	-0.0253381
Least	Audience indegree	9	-0.1195909	0.0540243	-0.2553522	0.0161703
Least	Audience indegree	10	-0.2624256	0.0521864	-0.3935685	-0.1312827
Least	Audience indegree	11	-0.1770897	0.0552494	-0.3159296	-0.0382497
Least	Audience indegree	12	-0.1796285	0.0563677	-0.3212788	-0.0379782
Least	Audience indegree	13	-0.2555506	0.0534222	-0.3897990	-0.1213022
Least	Audience indegree	14	-0.2474955	0.0236226	-0.3068584	-0.1881327
Least	Audience indegree	15	-0.1801952	0.0244848	-0.2417247	-0.1186656
Least	Audience indegree	16	-0.1620775	0.0268975	-0.2296701	-0.0944848
Least	Audience indegree	17	-0.1646539	0.1262793	-0.4819898	0.1526820
Least	Audience indegree	18	-0.0864700	0.0792173	-0.2855405	0.1126005
Least	Audience indegree	19	-0.3062846	0.0788024	-0.5043125	-0.1082567
Least	Audience indegree	20	-0.3005557	0.0587267	-0.4481341	-0.1529772
Least	Audience indegree	21	-0.3071955	0.0754126	-0.4967051	-0.1176859
Least	Audience indegree	22	-0.2852202	0.0489051	-0.4081170	-0.1623233
Least	Audience indegree	23	-0.3602539	0.0487757	-0.4828257	-0.2376821
Least	Audience indegree	24	-0.3119484	0.0475711	-0.4314932	-0.1924036
Least	Audience indegree	25	-0.2961627	0.0418955	-0.4014447	-0.1908808
Least	Audience indegree	26	-0.3507560	0.0487531	-0.4732711	-0.2282409
Least	Audience indegree	27	-0.2711379	0.0583366	-0.4177360	-0.1245397
Least	Audience indegree	28	-0.2937120	0.0338316	-0.3787298	-0.2086941

Least	Audience indegree	29	-0.2294315	0.0768520	-0.4225581	-0.0363048
Least	Audience indegree	30	-0.2764769	0.1370915	-0.6209836	0.0680297
Least	Audience indegree	31	-0.1742658	0.0668056	-0.3421463	-0.0063853
Least	Audience indegree	32	-0.1464848	0.1112577	-0.4260719	0.1331022
Least	Audience indegree	33	-0.1768001	0.1281443	-0.4988227	0.1452224
Least	Audience indegree	34	-0.1502225	0.0475063	-0.2696043	-0.0308406
Least	Audience indegree	35	-0.1991995	0.0318117	-0.2791413	-0.1192577
Least	Audience indegree	36	-0.1597159	0.0653340	-0.3238983	0.0044664
Least	Audience indegree	37	-0.2047877	0.0629555	-0.3629928	-0.0465826
Least	Audience indegree	38	-0.1615034	0.0566507	-0.3038649	-0.0191419
Least	Audience indegree	39	-0.2047935	0.0322788	-0.2859092	-0.1236778
Least	Audience indegree	40	-0.2079133	0.0347681	-0.2952844	-0.1205423
Least	Audience indegree	41	-0.1689747	0.0488224	-0.2916637	-0.0462856
Least	Audience indegree	42	-0.1935054	0.1772545	-0.6389403	0.2519295
Least	Audience indegree	43	-0.0946565	0.8165338	-2.1465803	1.9572674
Least	Audience indegree	44	-0.0495098	0.1697027	-0.4759674	0.3769478
Least	Audience indegree	45	-0.0717557	0.4463618	-1.1934489	1.0499374
Least	Audience indegree	47	-0.2240345	0.0095320	-0.2479881	-0.2000810
Least	Audience indegree	48	-0.2343712	0.0104304	-0.2605825	-0.2081600
Least	Audience indegree	49	-0.2336237	0.0117744	-0.2632125	-0.2040349
Least	Audience indegree	50	-0.2432750	0.0105173	-0.2697046	-0.2168454
Least	Audience indegree	51	-0.2367950	0.0244844	-0.2983234	-0.1752665
Least	Audience indegree	52	-0.2276106	0.0414849	-0.3318607	-0.1233604
Least	Audience indegree	53	-0.2367357	0.0807537	-0.4396672	-0.0338042
Least	Audience indegree	54	-0.2209612	0.0635223	-0.3805908	-0.0613315
Least	Audience indegree	55	-0.2180650	0.0180423	-0.2634048	-0.1727251
Least	Audience indegree	56	-0.1546364	0.0093286	-0.1780789	-0.1311939
Least	Audience indegree	57	-0.1302089	0.1589629	-0.5296777	0.2692599
Least	Audience indegree	58	-0.1912776	0.6001791	-1.6995090	1.3169538
Least	Audience indegree	59	-0.1829891	0.7735142	-2.1268061	1.7608278

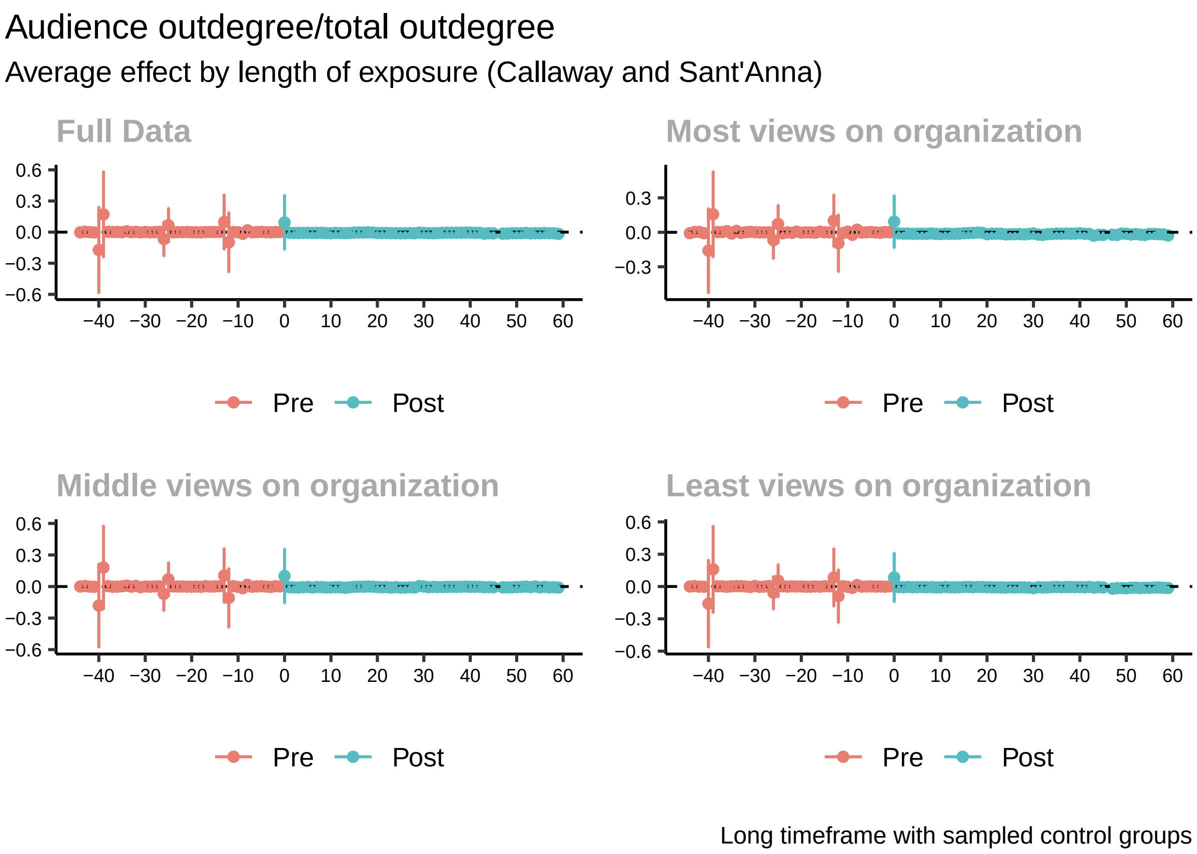

sample	outcome	event.time	estimate	std.error	conf.low	conf.high
Full	Audience outdegree/total outdegree	-44	-0.0021582	0.0006738	-0.0039503	-0.0003660
Full	Audience outdegree/total outdegree	-43	0.0038909	0.0015415	-0.0002093	0.0079910
Full	Audience outdegree/total outdegree	-42	-0.0000145	0.0011658	-0.0031155	0.0030864
Full	Audience outdegree/total outdegree	-41	-0.0035383	0.0020305	-0.0089390	0.0018625
Full	Audience outdegree/total outdegree	-40	-0.1719313	0.1577679	-0.5915672	0.2477046
Full	Audience outdegree/total outdegree	-39	0.1710736	0.1572409	-0.2471607	0.5893080
Full	Audience outdegree/total outdegree	-38	0.0030601	0.0013468	-0.0005221	0.0066422
Full	Audience outdegree/total outdegree	-37	0.0004131	0.0020369	-0.0050047	0.0058310
Full	Audience outdegree/total outdegree	-36	0.0014396	0.0011779	-0.0016934	0.0045726
Full	Audience outdegree/total outdegree	-35	-0.0012854	0.0016640	-0.0057113	0.0031404
Full	Audience outdegree/total outdegree	-34	0.0077786	0.0017608	0.0030951	0.0124621
Full	Audience outdegree/total outdegree	-33	-0.0011370	0.0009832	-0.0037521	0.0014781
Full	Audience outdegree/total outdegree	-32	0.0029735	0.0007250	0.0010451	0.0049019
Full	Audience outdegree/total outdegree	-31	-0.0027763	0.0005850	-0.0043321	-0.0012204
Full	Audience outdegree/total outdegree	-30	0.0019194	0.0006139	0.0002865	0.0035523
Full	Audience outdegree/total outdegree	-29	-0.0021376	0.0005266	-0.0035383	-0.0007370
Full	Audience outdegree/total outdegree	-28	-0.0002060	0.0007363	-0.0021645	0.0017525
Full	Audience outdegree/total outdegree	-27	0.0025938	0.0012847	-0.0008234	0.0060109
Full	Audience outdegree/total outdegree	-26	-0.0667651	0.0637433	-0.2363116	0.1027814

Full	Audience outdegree/total outdegree	-25	0.0664847	0.0637531	-0.1030879	0.2360573
Full	Audience outdegree/total outdegree	-24	-0.0015942	0.0008249	-0.0037882	0.0005998
Full	Audience outdegree/total outdegree	-23	-0.0001931	0.0015216	-0.0042403	0.0038542
Full	Audience outdegree/total outdegree	-22	-0.0006777	0.0015281	-0.0047422	0.0033867
Full	Audience outdegree/total outdegree	-21	0.0011904	0.0013347	-0.0023595	0.0047404
Full	Audience outdegree/total outdegree	-20	-0.0014435	0.0012917	-0.0048791	0.0019921
Full	Audience outdegree/total outdegree	-19	-0.0008852	0.0012902	-0.0043169	0.0025465
Full	Audience outdegree/total outdegree	-18	-0.0019262	0.0004193	-0.0030415	-0.0008110
Full	Audience outdegree/total outdegree	-17	0.0006737	0.0006914	-0.0011653	0.0025127
Full	Audience outdegree/total outdegree	-16	0.0008204	0.0007609	-0.0012035	0.0028442
Full	Audience outdegree/total outdegree	-15	0.0014616	0.0003058	0.0006482	0.0022750
Full	Audience outdegree/total outdegree	-14	0.0016880	0.0013562	-0.0019193	0.0052954
Full	Audience outdegree/total outdegree	-13	0.0970803	0.1010369	-0.1716608	0.3658214
Full	Audience outdegree/total outdegree	-12	-0.0982477	0.1099677	-0.3907433	0.1942480
Full	Audience outdegree/total outdegree	-11	0.0006214	0.0006163	-0.0010178	0.0022607
Full	Audience outdegree/total outdegree	-10	-0.0011914	0.0007127	-0.0030871	0.0007044
Full	Audience outdegree/total outdegree	-9	-0.0163181	0.0144346	-0.0547118	0.0220757
Full	Audience outdegree/total outdegree	-8	0.0170341	0.0147748	-0.0222643	0.0563325
Full	Audience outdegree/total outdegree	-7	-0.0017148	0.0004412	-0.0028884	-0.0005412
Full	Audience outdegree/total outdegree	-6	0.0003699	0.0008727	-0.0019513	0.0026911
Full	Audience outdegree/total outdegree	-5	0.0020807	0.0005329	0.0006632	0.0034981
Full	Audience outdegree/total outdegree	-4	-0.0005443	0.0007227	-0.0024666	0.0013780
Full	Audience outdegree/total outdegree	-3	-0.0025242	0.0007769	-0.0045905	-0.0004578
Full	Audience outdegree/total outdegree	-2	0.0008604	0.0005070	-0.0004882	0.0022090
Full	Audience outdegree/total outdegree	-1	0.0010530	0.0006317	-0.0006272	0.0027332
Full	Audience outdegree/total outdegree	0	0.0929773	0.1003595	-0.1739620	0.3599166
Full	Audience outdegree/total outdegree	1	-0.0062166	0.0010138	-0.0089131	-0.0035201
Full	Audience outdegree/total outdegree	2	-0.0086581	0.0011766	-0.0117876	-0.0055286
Full	Audience outdegree/total outdegree	3	-0.0078807	0.0012424	-0.0111852	-0.0045761
Full	Audience outdegree/total outdegree	4	-0.0082523	0.0011989	-0.0114411	-0.0050636
Full	Audience outdegree/total outdegree	5	-0.0068606	0.0018887	-0.0118843	-0.0018368
Full	Audience outdegree/total outdegree	6	-0.0085327	0.0015459	-0.0126444	-0.0044209
Full	Audience outdegree/total outdegree	7	-0.0075743	0.0015624	-0.0117301	-0.0034186
Full	Audience outdegree/total outdegree	8	-0.0066536	0.0014418	-0.0104886	-0.0028186
Full	Audience outdegree/total outdegree	9	-0.0100514	0.0013498	-0.0136415	-0.0064612
Full	Audience outdegree/total outdegree	10	-0.0103154	0.0019808	-0.0155839	-0.0050469
Full	Audience outdegree/total outdegree	11	-0.0072294	0.0015894	-0.0114570	-0.0030018
Full	Audience outdegree/total outdegree	12	-0.0088099	0.0018179	-0.0136452	-0.0039746
Full	Audience outdegree/total outdegree	13	-0.0108362	0.0013823	-0.0145129	-0.0071595
Full	Audience outdegree/total outdegree	14	-0.0084894	0.0017429	-0.0131251	-0.0038536
Full	Audience outdegree/total outdegree	15	-0.0051920	0.0012219	-0.0084422	-0.0019419

Full	Audience outdegree/total outdegree	16	-0.0038010	0.0010764	-0.0066639	-0.0009381
Full	Audience outdegree/total outdegree	17	-0.0044567	0.0017698	-0.0091642	0.0002507
Full	Audience outdegree/total outdegree	18	-0.0018899	0.0018181	-0.0067256	0.0029459
Full	Audience outdegree/total outdegree	19	-0.0035883	0.0015896	-0.0078164	0.0006399
Full	Audience outdegree/total outdegree	20	-0.0088558	0.0014376	-0.0126796	-0.0050320
Full	Audience outdegree/total outdegree	21	-0.0086476	0.0015952	-0.0128906	-0.0044046
Full	Audience outdegree/total outdegree	22	-0.0084862	0.0019242	-0.0136042	-0.0033683
Full	Audience outdegree/total outdegree	23	-0.0102554	0.0015631	-0.0144129	-0.0060979
Full	Audience outdegree/total outdegree	24	-0.0104765	0.0015252	-0.0145332	-0.0064198
Full	Audience outdegree/total outdegree	25	-0.0115819	0.0015371	-0.0156703	-0.0074935
Full	Audience outdegree/total outdegree	26	-0.0111782	0.0011268	-0.0141752	-0.0081812
Full	Audience outdegree/total outdegree	27	-0.0096393	0.0013481	-0.0132251	-0.0060535
Full	Audience outdegree/total outdegree	28	-0.0111426	0.0017030	-0.0156723	-0.0066129
Full	Audience outdegree/total outdegree	29	-0.0056124	0.0042693	-0.0169681	0.0057433
Full	Audience outdegree/total outdegree	30	-0.0078383	0.0012276	-0.0111035	-0.0045730
Full	Audience outdegree/total outdegree	31	-0.0100850	0.0036006	-0.0196621	-0.0005079
Full	Audience outdegree/total outdegree	32	-0.0108919	0.0028002	-0.0183400	-0.0034439
Full	Audience outdegree/total outdegree	33	-0.0095709	0.0011807	-0.0127112	-0.0064305
Full	Audience outdegree/total outdegree	34	-0.0077134	0.0012701	-0.0110915	-0.0043352
Full	Audience outdegree/total outdegree	35	-0.0059315	0.0017273	-0.0105258	-0.0013372
Full	Audience outdegree/total outdegree	36	-0.0070000	0.0020249	-0.0123858	-0.0016142
Full	Audience outdegree/total outdegree	37	-0.0062902	0.0021488	-0.0120055	-0.0005748
Full	Audience outdegree/total outdegree	38	-0.0058091	0.0021596	-0.0115534	-0.0000649
Full	Audience outdegree/total outdegree	39	-0.0049661	0.0017154	-0.0095288	-0.0004034
Full	Audience outdegree/total outdegree	40	-0.0050225	0.0020641	-0.0105126	0.0004677
Full	Audience outdegree/total outdegree	41	-0.0069630	0.0027250	-0.0142111	0.0002852
Full	Audience outdegree/total outdegree	42	-0.0060874	0.0020241	-0.0114712	-0.0007036
Full	Audience outdegree/total outdegree	43	-0.0148907	0.0016361	-0.0192425	-0.0105390
Full	Audience outdegree/total outdegree	44	-0.0104537	0.0010553	-0.0132605	-0.0076469
Full	Audience outdegree/total outdegree	45	-0.0125015	0.0013469	-0.0160840	-0.0089190
Full	Audience outdegree/total outdegree	47	-0.0150011	NA	NA	NA
Full	Audience outdegree/total outdegree	48	-0.0141784	NA	NA	NA
Full	Audience outdegree/total outdegree	49	-0.0105545	NA	NA	NA
Full	Audience outdegree/total outdegree	50	-0.0105175	NA	NA	NA
Full	Audience outdegree/total outdegree	51	-0.0105107	0.0010288	-0.0132470	-0.0077743
Full	Audience outdegree/total outdegree	52	-0.0078507	0.0012904	-0.0112831	-0.0044184
Full	Audience outdegree/total outdegree	53	-0.0126344	0.0003513	-0.0135688	-0.0117000
Full	Audience outdegree/total outdegree	54	-0.0099338	0.0016829	-0.0144100	-0.0054575
Full	Audience outdegree/total outdegree	55	-0.0109817	0.0007635	-0.0130125	-0.0089509
Full	Audience outdegree/total outdegree	56	-0.0088739	0.0042389	-0.0201488	0.0024009
Full	Audience outdegree/total outdegree	57	-0.0107010	0.0099587	-0.0371895	0.0157875

Full	Audience outdegree/total outdegree	58	-0.0118786	0.0027189	-0.0191103	-0.0046469
Full	Audience outdegree/total outdegree	59	-0.0166248	0.0018630	-0.0215800	-0.0116696

sample	outcome	event.time	estimate	std.error	conf.low	conf.high
Most	Audience outdegree/total outdegree	-44	-0.0079560	0.0023925	-0.0140389	-0.0018732
Most	Audience outdegree/total outdegree	-43	0.0029655	0.0027794	-0.0041010	0.0100321
Most	Audience outdegree/total outdegree	-42	0.0031202	0.0015185	-0.0007404	0.0069808
Most	Audience outdegree/total outdegree	-41	-0.0078134	0.0030848	-0.0156563	0.0000296
Most	Audience outdegree/total outdegree	-40	-0.1594866	0.1465021	-0.5319578	0.2129845
Most	Audience outdegree/total outdegree	-39	0.1571000	0.1480225	-0.2192366	0.5334366
Most	Audience outdegree/total outdegree	-38	0.0021921	0.0030322	-0.0055169	0.0099012
Most	Audience outdegree/total outdegree	-37	0.0020474	0.0046582	-0.0097958	0.0138907
Most	Audience outdegree/total outdegree	-36	0.0098833	0.0023758	0.0038429	0.0159237
Most	Audience outdegree/total outdegree	-35	-0.0108139	0.0034756	-0.0196504	-0.0019775
Most	Audience outdegree/total outdegree	-34	0.0123474	0.0035769	0.0032533	0.0214414
Most	Audience outdegree/total outdegree	-33	-0.0052023	0.0031709	-0.0132641	0.0028594
Most	Audience outdegree/total outdegree	-32	0.0011866	0.0027371	-0.0057721	0.0081454
Most	Audience outdegree/total outdegree	-31	0.0042334	0.0022798	-0.0015629	0.0100297
Most	Audience outdegree/total outdegree	-30	0.0009783	0.0023708	-0.0050492	0.0070058
Most	Audience outdegree/total outdegree	-29	-0.0007380	0.0020034	-0.0058315	0.0043555
Most	Audience outdegree/total outdegree	-28	-0.0006910	0.0011296	-0.0035628	0.0021808
Most	Audience outdegree/total outdegree	-27	-0.0000322	0.0028072	-0.0071693	0.0071049
Most	Audience outdegree/total outdegree	-26	-0.0683904	0.0650422	-0.2337555	0.0969748
Most	Audience outdegree/total outdegree	-25	0.0728294	0.0663504	-0.0958617	0.2415206
Most	Audience outdegree/total outdegree	-24	-0.0086616	0.0036053	-0.0178277	0.0005046
Most	Audience outdegree/total outdegree	-23	-0.0004952	0.0037168	-0.0099450	0.0089545
Most	Audience outdegree/total outdegree	-22	-0.0048047	0.0022725	-0.0105823	0.0009728
Most	Audience outdegree/total outdegree	-21	0.0061000	0.0022613	0.0003509	0.0118491
Most	Audience outdegree/total outdegree	-20	-0.0025966	0.0029401	-0.0100715	0.0048783
Most	Audience outdegree/total outdegree	-19	-0.0000889	0.0028885	-0.0074326	0.0072549
Most	Audience outdegree/total outdegree	-18	-0.0014710	0.0029281	-0.0089154	0.0059734
Most	Audience outdegree/total outdegree	-17	-0.0035256	0.0016311	-0.0076726	0.0006213
Most	Audience outdegree/total outdegree	-16	0.0055145	0.0022245	-0.0001413	0.0111702
Most	Audience outdegree/total outdegree	-15	0.0001160	0.0016435	-0.0040625	0.0042946
Most	Audience outdegree/total outdegree	-14	-0.0002251	0.0009665	-0.0026823	0.0022322
Most	Audience outdegree/total outdegree	-13	0.1011798	0.0910178	-0.1302262	0.3325859
Most	Audience outdegree/total outdegree	-12	-0.0946808	0.0996012	-0.3479097	0.1585482
Most	Audience outdegree/total outdegree	-11	-0.0066897	0.0017912	-0.0112438	-0.0021357
Most	Audience outdegree/total outdegree	-10	0.0054633	0.0029319	-0.0019909	0.0129176
Most	Audience outdegree/total outdegree	-9	-0.0219663	0.0154898	-0.0613479	0.0174154
Most	Audience outdegree/total outdegree	-8	0.0226101	0.0157268	-0.0173742	0.0625945

Most	Audience outdegree/total outdegree	-7	-0.0016224	0.0020069	-0.0067247	0.0034799
Most	Audience outdegree/total outdegree	-6	-0.0001542	0.0029444	-0.0076401	0.0073318
Most	Audience outdegree/total outdegree	-5	0.0020084	0.0019903	-0.0030517	0.0070686
Most	Audience outdegree/total outdegree	-4	-0.0009618	0.0017571	-0.0054291	0.0035054
Most	Audience outdegree/total outdegree	-3	-0.0033347	0.0015684	-0.0073222	0.0006528
Most	Audience outdegree/total outdegree	-2	0.0019731	0.0018101	-0.0026289	0.0065752
Most	Audience outdegree/total outdegree	-1	0.0005722	0.0014686	-0.0031616	0.0043060
Most	Audience outdegree/total outdegree	0	0.0927293	0.0912012	-0.1391433	0.3246019
Most	Audience outdegree/total outdegree	1	-0.0089370	0.0017884	-0.0134838	-0.0043901
Most	Audience outdegree/total outdegree	2	-0.0120144	0.0015726	-0.0160126	-0.0080163
Most	Audience outdegree/total outdegree	3	-0.0106466	0.0025300	-0.0170789	-0.0042142
Most	Audience outdegree/total outdegree	4	-0.0130902	0.0018979	-0.0179155	-0.0082648
Most	Audience outdegree/total outdegree	5	-0.0123491	0.0030614	-0.0201326	-0.0045657
Most	Audience outdegree/total outdegree	6	-0.0124393	0.0030358	-0.0201576	-0.0047211
Most	Audience outdegree/total outdegree	7	-0.0130588	0.0022589	-0.0188019	-0.0073156
Most	Audience outdegree/total outdegree	8	-0.0096416	0.0027125	-0.0165381	-0.0027451
Most	Audience outdegree/total outdegree	9	-0.0137421	0.0026907	-0.0205831	-0.0069011
Most	Audience outdegree/total outdegree	10	-0.0148030	0.0037568	-0.0243545	-0.0052516
Most	Audience outdegree/total outdegree	11	-0.0113638	0.0026038	-0.0179839	-0.0047438
Most	Audience outdegree/total outdegree	12	-0.0133309	0.0026441	-0.0200533	-0.0066086
Most	Audience outdegree/total outdegree	13	-0.0139815	0.0023693	-0.0200052	-0.0079578
Most	Audience outdegree/total outdegree	14	-0.0117602	0.0030271	-0.0194565	-0.0040639
Most	Audience outdegree/total outdegree	15	-0.0083556	0.0027265	-0.0152877	-0.0014236
Most	Audience outdegree/total outdegree	16	-0.0066411	0.0040572	-0.0169562	0.0036740
Most	Audience outdegree/total outdegree	17	-0.0052628	0.0043854	-0.0164123	0.0058868
Most	Audience outdegree/total outdegree	18	-0.0017212	0.0045077	-0.0131817	0.0097393
Most	Audience outdegree/total outdegree	19	-0.0048371	0.0026601	-0.0116001	0.0019260
Most	Audience outdegree/total outdegree	20	-0.0162757	0.0031868	-0.0243778	-0.0081736
Most	Audience outdegree/total outdegree	21	-0.0118611	0.0033040	-0.0202613	-0.0034609
Most	Audience outdegree/total outdegree	22	-0.0118274	0.0036987	-0.0212312	-0.0024237
Most	Audience outdegree/total outdegree	23	-0.0131196	0.0033957	-0.0217530	-0.0044862
Most	Audience outdegree/total outdegree	24	-0.0179197	0.0036818	-0.0272804	-0.0085590
Most	Audience outdegree/total outdegree	25	-0.0165129	0.0031939	-0.0246332	-0.0083926
Most	Audience outdegree/total outdegree	26	-0.0155044	0.0025098	-0.0218855	-0.0091233
Most	Audience outdegree/total outdegree	27	-0.0153963	0.0027490	-0.0223854	-0.0084071
Most	Audience outdegree/total outdegree	28	-0.0176334	0.0030223	-0.0253172	-0.0099495
Most	Audience outdegree/total outdegree	29	-0.0141790	0.0074540	-0.0331302	0.0047722
Most	Audience outdegree/total outdegree	30	-0.0101040	0.0028086	-0.0172448	-0.0029632
Most	Audience outdegree/total outdegree	31	-0.0197829	0.0061935	-0.0355295	-0.0040363
Most	Audience outdegree/total outdegree	32	-0.0221731	0.0055310	-0.0362354	-0.0081109
Most	Audience outdegree/total outdegree	33	-0.0163100	0.0047182	-0.0283057	-0.0043143

Most	Audience outdegree/total outdegree	34	-0.0147550	0.0027311	-0.0216987	-0.0078113
Most	Audience outdegree/total outdegree	35	-0.0114889	0.0027146	-0.0183906	-0.0045872
Most	Audience outdegree/total outdegree	36	-0.0128285	0.0045418	-0.0243756	-0.0012814
Most	Audience outdegree/total outdegree	37	-0.0112911	0.0030873	-0.0191404	-0.0034418
Most	Audience outdegree/total outdegree	38	-0.0117867	0.0032618	-0.0200796	-0.0034938
Most	Audience outdegree/total outdegree	39	-0.0112311	0.0015397	-0.0151457	-0.0073165
Most	Audience outdegree/total outdegree	40	-0.0079925	0.0053953	-0.0217096	0.0057246
Most	Audience outdegree/total outdegree	41	-0.0129179	0.0047160	-0.0249080	-0.0009278
Most	Audience outdegree/total outdegree	42	-0.0138404	0.0029229	-0.0212716	-0.0064092
Most	Audience outdegree/total outdegree	43	-0.0296513	0.0068322	-0.0470217	-0.0122808
Most	Audience outdegree/total outdegree	44	-0.0215829	0.0108471	-0.0491610	0.0059952
Most	Audience outdegree/total outdegree	45	-0.0223841	0.0110466	-0.0504694	0.0057011
Most	Audience outdegree/total outdegree	47	-0.0210915	NA	NA	NA
Most	Audience outdegree/total outdegree	48	-0.0225375	NA	NA	NA
Most	Audience outdegree/total outdegree	49	-0.0109978	NA	NA	NA
Most	Audience outdegree/total outdegree	50	-0.0129072	NA	NA	NA
Most	Audience outdegree/total outdegree	51	-0.0186995	0.0053186	-0.0322217	-0.0051774
Most	Audience outdegree/total outdegree	52	-0.0149145	0.0054777	-0.0288411	-0.0009878
Most	Audience outdegree/total outdegree	53	-0.0200603	0.0020068	-0.0251625	-0.0149581
Most	Audience outdegree/total outdegree	54	-0.0228197	0.0167988	-0.0655294	0.0198901
Most	Audience outdegree/total outdegree	55	-0.0139799	0.0009237	-0.0163283	-0.0116314
Most	Audience outdegree/total outdegree	56	-0.0145928	0.0082113	-0.0354694	0.0062839
Most	Audience outdegree/total outdegree	57	-0.0159452	0.0038769	-0.0258019	-0.0060884
Most	Audience outdegree/total outdegree	58	-0.0182136	0.0067069	-0.0352655	-0.0011618
Most	Audience outdegree/total outdegree	59	-0.0271664	0.0025259	-0.0335883	-0.0207445

sample	outcome	event.time	estimate	std.error	conf.low	conf.high
Middle	Audience outdegree/total outdegree	-44	0.0000018	0.0007232	-0.0018682	0.0018718
Middle	Audience outdegree/total outdegree	-43	0.0045827	0.0014560	0.0008179	0.0083474
Middle	Audience outdegree/total outdegree	-42	-0.0006818	0.0016474	-0.0049415	0.0035778
Middle	Audience outdegree/total outdegree	-41	-0.0021446	0.0022777	-0.0080341	0.0037449
Middle	Audience outdegree/total outdegree	-40	-0.1816234	0.1553913	-0.5834236	0.2201768
Middle	Audience outdegree/total outdegree	-39	0.1808141	0.1549246	-0.2197792	0.5814074
Middle	Audience outdegree/total outdegree	-38	0.0043100	0.0019042	-0.0006138	0.0092339
Middle	Audience outdegree/total outdegree	-37	-0.0021594	0.0020306	-0.0074101	0.0030913
Middle	Audience outdegree/total outdegree	-36	-0.0010899	0.0020688	-0.0064393	0.0042594
Middle	Audience outdegree/total outdegree	-35	0.0024115	0.0017269	-0.0020539	0.0068769
Middle	Audience outdegree/total outdegree	-34	0.0081172	0.0024572	0.0017634	0.0144709
Middle	Audience outdegree/total outdegree	-33	-0.0012426	0.0013997	-0.0048617	0.0023765
Middle	Audience outdegree/total outdegree	-32	0.0061893	0.0017752	0.0015992	0.0107793
Middle	Audience outdegree/total outdegree	-31	-0.0062955	0.0012436	-0.0095110	-0.0030799

Middle	Audience outdegree/total outdegree	-30	0.0004071	0.0006475	-0.0012672	0.0020815
Middle	Audience outdegree/total outdegree	-29	-0.0020617	0.0008223	-0.0041880	0.0000646
Middle	Audience outdegree/total outdegree	-28	0.0007847	0.0016057	-0.0033671	0.0049366
Middle	Audience outdegree/total outdegree	-27	0.0025590	0.0011665	-0.0004572	0.0055753
Middle	Audience outdegree/total outdegree	-26	-0.0704222	0.0639500	-0.2357797	0.0949353
Middle	Audience outdegree/total outdegree	-25	0.0689195	0.0637941	-0.0960348	0.2338739
Middle	Audience outdegree/total outdegree	-24	0.0006248	0.0010850	-0.0021807	0.0034303
Middle	Audience outdegree/total outdegree	-23	0.0000171	0.0011312	-0.0029079	0.0029422
Middle	Audience outdegree/total outdegree	-22	0.0006422	0.0015601	-0.0033917	0.0046761
Middle	Audience outdegree/total outdegree	-21	-0.0012596	0.0012463	-0.0044823	0.0019631
Middle	Audience outdegree/total outdegree	-20	-0.0023541	0.0015193	-0.0062825	0.0015744
Middle	Audience outdegree/total outdegree	-19	-0.0002158	0.0015110	-0.0041228	0.0036912
Middle	Audience outdegree/total outdegree	-18	-0.0027292	0.0010599	-0.0054700	0.0000115
Middle	Audience outdegree/total outdegree	-17	0.0034364	0.0017140	-0.0009956	0.0078684
Middle	Audience outdegree/total outdegree	-16	-0.0004744	0.0005882	-0.0019953	0.0010464
Middle	Audience outdegree/total outdegree	-15	0.0009826	0.0005268	-0.0003797	0.0023448
Middle	Audience outdegree/total outdegree	-14	0.0030472	0.0015172	-0.0008758	0.0069702
Middle	Audience outdegree/total outdegree	-13	0.1048906	0.1017738	-0.1582690	0.3680502
Middle	Audience outdegree/total outdegree	-12	-0.1079510	0.1106393	-0.3940344	0.1781325
Middle	Audience outdegree/total outdegree	-11	0.0030540	0.0014077	-0.0005861	0.0066940
Middle	Audience outdegree/total outdegree	-10	-0.0036757	0.0016621	-0.0079735	0.0006221
Middle	Audience outdegree/total outdegree	-9	-0.0154697	0.0163844	-0.0578353	0.0268960
Middle	Audience outdegree/total outdegree	-8	0.0163885	0.0148767	-0.0220786	0.0548556
Middle	Audience outdegree/total outdegree	-7	-0.0022419	0.0012938	-0.0055875	0.0011036
Middle	Audience outdegree/total outdegree	-6	0.0010343	0.0010686	-0.0017287	0.0037973
Middle	Audience outdegree/total outdegree	-5	0.0027586	0.0006609	0.0010497	0.0044674
Middle	Audience outdegree/total outdegree	-4	-0.0002703	0.0009448	-0.0027132	0.0021727
Middle	Audience outdegree/total outdegree	-3	-0.0042181	0.0011976	-0.0073148	-0.0011213
Middle	Audience outdegree/total outdegree	-2	0.0022501	0.0009437	-0.0001901	0.0046904
Middle	Audience outdegree/total outdegree	-1	0.0007977	0.0012985	-0.0025598	0.0041552
Middle	Audience outdegree/total outdegree	0	0.1002758	0.1014602	-0.1620731	0.3626247
Middle	Audience outdegree/total outdegree	1	-0.0057254	0.0016221	-0.0099198	-0.0015310
Middle	Audience outdegree/total outdegree	2	-0.0082110	0.0018107	-0.0128931	-0.0035289
Middle	Audience outdegree/total outdegree	3	-0.0092113	0.0020054	-0.0143968	-0.0040258
Middle	Audience outdegree/total outdegree	4	-0.0064438	0.0016243	-0.0106438	-0.0022437
Middle	Audience outdegree/total outdegree	5	-0.0042711	0.0018824	-0.0091386	0.0005963
Middle	Audience outdegree/total outdegree	6	-0.0085663	0.0020102	-0.0137641	-0.0033686
Middle	Audience outdegree/total outdegree	7	-0.0046346	0.0021903	-0.0102980	0.0010288
Middle	Audience outdegree/total outdegree	8	-0.0056236	0.0022718	-0.0114978	0.0002507
Middle	Audience outdegree/total outdegree	9	-0.0088037	0.0016816	-0.0131518	-0.0044556
Middle	Audience outdegree/total outdegree	10	-0.0088958	0.0023655	-0.0150123	-0.0027792

Middle	Audience outdegree/total outdegree	11	-0.0063859	0.0022579	-0.0122241	-0.0005476
Middle	Audience outdegree/total outdegree	12	-0.0067869	0.0019955	-0.0119468	-0.0016270
Middle	Audience outdegree/total outdegree	13	-0.0116477	0.0021083	-0.0170991	-0.0061963
Middle	Audience outdegree/total outdegree	14	-0.0074758	0.0020451	-0.0127639	-0.0021878
Middle	Audience outdegree/total outdegree	15	-0.0030544	0.0010204	-0.0056929	-0.0004159
Middle	Audience outdegree/total outdegree	16	-0.0015156	0.0017797	-0.0061174	0.0030863
Middle	Audience outdegree/total outdegree	17	-0.0019138	0.0011883	-0.0049866	0.0011589
Middle	Audience outdegree/total outdegree	18	0.0003656	0.0021483	-0.0051892	0.0059204
Middle	Audience outdegree/total outdegree	19	-0.0011855	0.0019822	-0.0063109	0.0039399
Middle	Audience outdegree/total outdegree	20	-0.0055556	0.0022531	-0.0113814	0.0002702
Middle	Audience outdegree/total outdegree	21	-0.0074395	0.0023096	-0.0134115	-0.0014674
Middle	Audience outdegree/total outdegree	22	-0.0059933	0.0024348	-0.0122891	0.0003024
Middle	Audience outdegree/total outdegree	23	-0.0097807	0.0016067	-0.0139351	-0.0056263
Middle	Audience outdegree/total outdegree	24	-0.0056245	0.0023731	-0.0117606	0.0005116
Middle	Audience outdegree/total outdegree	25	-0.0114166	0.0022496	-0.0172335	-0.0055998
Middle	Audience outdegree/total outdegree	26	-0.0097793	0.0015576	-0.0138069	-0.0057517
Middle	Audience outdegree/total outdegree	27	-0.0074505	0.0017631	-0.0120094	-0.0028915
Middle	Audience outdegree/total outdegree	28	-0.0090170	0.0019825	-0.0141431	-0.0038909
Middle	Audience outdegree/total outdegree	29	0.0044902	0.0043515	-0.0067616	0.0157421
Middle	Audience outdegree/total outdegree	30	0.0000428	0.0010402	-0.0026469	0.0027326
Middle	Audience outdegree/total outdegree	31	-0.0057747	0.0058716	-0.0209570	0.0094075
Middle	Audience outdegree/total outdegree	32	-0.0038671	0.0035428	-0.0130278	0.0052937
Middle	Audience outdegree/total outdegree	33	-0.0052834	0.0024383	-0.0115880	0.0010213
Middle	Audience outdegree/total outdegree	34	-0.0047540	0.0016298	-0.0089684	-0.0005397
Middle	Audience outdegree/total outdegree	35	-0.0033755	0.0019909	-0.0085233	0.0017723
Middle	Audience outdegree/total outdegree	36	-0.0033432	0.0009820	-0.0058824	-0.0008041
Middle	Audience outdegree/total outdegree	37	-0.0035553	0.0013824	-0.0071297	0.0000191
Middle	Audience outdegree/total outdegree	38	-0.0027782	0.0030754	-0.0107305	0.0051740
Middle	Audience outdegree/total outdegree	39	-0.0004104	0.0028835	-0.0078662	0.0070455
Middle	Audience outdegree/total outdegree	40	-0.0027195	0.0018424	-0.0074835	0.0020446
Middle	Audience outdegree/total outdegree	41	-0.0028120	0.0033006	-0.0113465	0.0057226
Middle	Audience outdegree/total outdegree	42	-0.0027217	0.0024142	-0.0089642	0.0035208
Middle	Audience outdegree/total outdegree	43	-0.0063799	0.0042567	-0.0173866	0.0046267
Middle	Audience outdegree/total outdegree	44	-0.0051971	0.0014731	-0.0090062	-0.0013880
Middle	Audience outdegree/total outdegree	45	-0.0072256	0.0031386	-0.0153411	0.0008899
Middle	Audience outdegree/total outdegree	47	-0.0079014	NA	NA	NA
Middle	Audience outdegree/total outdegree	48	-0.0086375	NA	NA	NA
Middle	Audience outdegree/total outdegree	49	-0.0075432	NA	NA	NA
Middle	Audience outdegree/total outdegree	50	-0.0048155	NA	NA	NA
Middle	Audience outdegree/total outdegree	51	-0.0039571	0.0007346	-0.0058565	-0.0020577
Middle	Audience outdegree/total outdegree	52	-0.0008426	0.0035298	-0.0099697	0.0082845

Middle	Audience outdegree/total outdegree	53	-0.0058717	0.0010061	-0.0084734	-0.0032701
Middle	Audience outdegree/total outdegree	54	0.0005092	0.0043227	-0.0106682	0.0116866
Middle	Audience outdegree/total outdegree	55	-0.0079326	0.0020506	-0.0132350	-0.0026302
Middle	Audience outdegree/total outdegree	56	-0.0032502	0.0047718	-0.0155890	0.0090885
Middle	Audience outdegree/total outdegree	57	-0.0076271	0.0026641	-0.0145158	-0.0007383
Middle	Audience outdegree/total outdegree	58	-0.0065771	0.0017415	-0.0110801	-0.0020741
Middle	Audience outdegree/total outdegree	59	-0.0101503	0.0081653	-0.0312637	0.0109631

sample	outcome	event.time	estimate	std.error	conf.low	conf.high
Least	Audience outdegree/total outdegree	-44	0.0000143	0.0019350	-0.0046842	0.0047128
Least	Audience outdegree/total outdegree	-43	0.0037741	0.0022587	-0.0017105	0.0092587
Least	Audience outdegree/total outdegree	-42	-0.0013118	0.0018954	-0.0059142	0.0032906
Least	Audience outdegree/total outdegree	-41	-0.0021959	0.0019718	-0.0069837	0.0025919
Least	Audience outdegree/total outdegree	-40	-0.1593566	0.1690351	-0.5698013	0.2510881
Least	Audience outdegree/total outdegree	-39	0.1598557	0.1679961	-0.2480664	0.5677777
Least	Audience outdegree/total outdegree	-38	0.0022081	0.0030856	-0.0052842	0.0097004
Least	Audience outdegree/total outdegree	-37	0.0019573	0.0035795	-0.0067344	0.0106490
Least	Audience outdegree/total outdegree	-36	-0.0020430	0.0016573	-0.0060672	0.0019811
Least	Audience outdegree/total outdegree	-35	0.0021219	0.0020433	-0.0028396	0.0070834
Least	Audience outdegree/total outdegree	-34	0.0036814	0.0015117	0.0000107	0.0073520
Least	Audience outdegree/total outdegree	-33	0.0026038	0.0014030	-0.0008030	0.0060106
Least	Audience outdegree/total outdegree	-32	0.0002878	0.0018562	-0.0042194	0.0047951
Least	Audience outdegree/total outdegree	-31	-0.0041366	0.0011512	-0.0069320	-0.0013412
Least	Audience outdegree/total outdegree	-30	0.0044222	0.0013094	0.0012427	0.0076016
Least	Audience outdegree/total outdegree	-29	-0.0033251	0.0013611	-0.0066299	-0.0000202
Least	Audience outdegree/total outdegree	-28	-0.0009405	0.0016474	-0.0049408	0.0030597
Least	Audience outdegree/total outdegree	-27	0.0049430	0.0005391	0.0036339	0.0062520
Least	Audience outdegree/total outdegree	-26	-0.0578098	0.0650124	-0.2156706	0.1000510
Least	Audience outdegree/total outdegree	-25	0.0548183	0.0640654	-0.1007429	0.2103796
Least	Audience outdegree/total outdegree	-24	0.0016352	0.0012489	-0.0013974	0.0046677
Least	Audience outdegree/total outdegree	-23	-0.0006869	0.0019136	-0.0053336	0.0039597
Least	Audience outdegree/total outdegree	-22	0.0011851	0.0013422	-0.0020739	0.0044442
Least	Audience outdegree/total outdegree	-21	0.0000569	0.0013957	-0.0033320	0.0034459
Least	Audience outdegree/total outdegree	-20	0.0005365	0.0005880	-0.0008912	0.0019643
Least	Audience outdegree/total outdegree	-19	-0.0018089	0.0011921	-0.0047035	0.0010856
Least	Audience outdegree/total outdegree	-18	-0.0016959	0.0012376	-0.0047011	0.0013092
Least	Audience outdegree/total outdegree	-17	0.0005324	0.0008612	-0.0015586	0.0026235
Least	Audience outdegree/total outdegree	-16	-0.0014012	0.0008561	-0.0034800	0.0006776
Least	Audience outdegree/total outdegree	-15	0.0029797	0.0010124	0.0005215	0.0054380
Least	Audience outdegree/total outdegree	-14	0.0016096	0.0014816	-0.0019879	0.0052072
Least	Audience outdegree/total outdegree	-13	0.0846203	0.1131653	-0.1901635	0.3594040

Least	Audience outdegree/total outdegree	-12	-0.0895044	0.1039163	-0.3418301	0.1628212
Least	Audience outdegree/total outdegree	-11	0.0033842	0.0008979	0.0012038	0.0055645
Least	Audience outdegree/total outdegree	-10	-0.0033160	0.0008098	-0.0052824	-0.0013496
Least	Audience outdegree/total outdegree	-9	-0.0124672	0.0129763	-0.0439758	0.0190415
Least	Audience outdegree/total outdegree	-8	0.0129697	0.0135248	-0.0198706	0.0458101
Least	Audience outdegree/total outdegree	-7	-0.0010506	0.0017798	-0.0053723	0.0032711
Least	Audience outdegree/total outdegree	-6	-0.0000982	0.0018179	-0.0045123	0.0043159
Least	Audience outdegree/total outdegree	-5	0.0012790	0.0005916	-0.0001576	0.0027156
Least	Audience outdegree/total outdegree	-4	-0.0004635	0.0007816	-0.0023613	0.0014343
Least	Audience outdegree/total outdegree	-3	0.0000468	0.0007855	-0.0018606	0.0019541
Least	Audience outdegree/total outdegree	-2	-0.0017112	0.0006943	-0.0033972	-0.0000253
Least	Audience outdegree/total outdegree	-1	0.0018356	0.0013376	-0.0014123	0.0050834
Least	Audience outdegree/total outdegree	0	0.0846732	0.0957495	-0.1478222	0.3171685
Least	Audience outdegree/total outdegree	1	-0.0045599	0.0010864	-0.0071977	-0.0019220
Least	Audience outdegree/total outdegree	2	-0.0064708	0.0010467	-0.0090122	-0.0039293
Least	Audience outdegree/total outdegree	3	-0.0038998	0.0008270	-0.0059078	-0.0018918
Least	Audience outdegree/total outdegree	4	-0.0064294	0.0007050	-0.0081413	-0.0047176
Least	Audience outdegree/total outdegree	5	-0.0055487	0.0013016	-0.0087092	-0.0023881
Least	Audience outdegree/total outdegree	6	-0.0051606	0.0011902	-0.0080506	-0.0022707
Least	Audience outdegree/total outdegree	7	-0.0064397	0.0009643	-0.0087812	-0.0040983
Least	Audience outdegree/total outdegree	8	-0.0051951	0.0007537	-0.0070252	-0.0033649
Least	Audience outdegree/total outdegree	9	-0.0081621	0.0011570	-0.0109716	-0.0053527
Least	Audience outdegree/total outdegree	10	-0.0080279	0.0021508	-0.0132503	-0.0028055
Least	Audience outdegree/total outdegree	11	-0.0046194	0.0013267	-0.0078408	-0.0013979
Least	Audience outdegree/total outdegree	12	-0.0074418	0.0014338	-0.0109234	-0.0039602
Least	Audience outdegree/total outdegree	13	-0.0068318	0.0016309	-0.0107919	-0.0028717
Least	Audience outdegree/total outdegree	14	-0.0068074	0.0013063	-0.0099793	-0.0036355
Least	Audience outdegree/total outdegree	15	-0.0046292	0.0020228	-0.0095409	0.0002825
Least	Audience outdegree/total outdegree	16	-0.0036792	0.0021321	-0.0088563	0.0014979
Least	Audience outdegree/total outdegree	17	-0.0058994	0.0027672	-0.0126186	0.0008198
Least	Audience outdegree/total outdegree	18	-0.0040569	0.0016626	-0.0080940	-0.0000198
Least	Audience outdegree/total outdegree	19	-0.0049294	0.0028917	-0.0119509	0.0020922
Least	Audience outdegree/total outdegree	20	-0.0062740	0.0013512	-0.0095550	-0.0029931
Least	Audience outdegree/total outdegree	21	-0.0070892	0.0010194	-0.0095643	-0.0046140
Least	Audience outdegree/total outdegree	22	-0.0082638	0.0013338	-0.0115025	-0.0050250
Least	Audience outdegree/total outdegree	23	-0.0080674	0.0012702	-0.0111517	-0.0049830
Least	Audience outdegree/total outdegree	24	-0.0098023	0.0014859	-0.0134104	-0.0061942
Least	Audience outdegree/total outdegree	25	-0.0073745	0.0012498	-0.0104091	-0.0043398
Least	Audience outdegree/total outdegree	26	-0.0089054	0.0015314	-0.0126239	-0.0051869
Least	Audience outdegree/total outdegree	27	-0.0072823	0.0010360	-0.0097978	-0.0047668
Least	Audience outdegree/total outdegree	28	-0.0081311	0.0015722	-0.0119486	-0.0043136

Least	Audience outdegree/total outdegree	29	-0.0094874	0.0044058	-0.0201855	0.0012107
Least	Audience outdegree/total outdegree	30	-0.0148635	0.0062028	-0.0299249	0.0001979
Least	Audience outdegree/total outdegree	31	-0.0063521	0.0033433	-0.0144702	0.0017660
Least	Audience outdegree/total outdegree	32	-0.0092772	0.0042217	-0.0195283	0.0009738
Least	Audience outdegree/total outdegree	33	-0.0087297	0.0067592	-0.0251421	0.0076827
Least	Audience outdegree/total outdegree	34	-0.0052598	0.0027604	-0.0119626	0.0014429
Least	Audience outdegree/total outdegree	35	-0.0042723	0.0020717	-0.0093028	0.0007582
Least	Audience outdegree/total outdegree	36	-0.0064877	0.0033209	-0.0145514	0.0015761
Least	Audience outdegree/total outdegree	37	-0.0053441	0.0020359	-0.0102876	-0.0004007
Least	Audience outdegree/total outdegree	38	-0.0043788	0.0032461	-0.0122609	0.0035032
Least	Audience outdegree/total outdegree	39	-0.0052018	0.0027576	-0.0118977	0.0014942
Least	Audience outdegree/total outdegree	40	-0.0053143	0.0027639	-0.0120255	0.0013969
Least	Audience outdegree/total outdegree	41	-0.0069614	0.0014415	-0.0104616	-0.0034612
Least	Audience outdegree/total outdegree	42	-0.0034235	0.0007618	-0.0052732	-0.0015738
Least	Audience outdegree/total outdegree	43	-0.0099171	0.0057728	-0.0239344	0.0041003
Least	Audience outdegree/total outdegree	44	-0.0052254	0.0041701	-0.0153511	0.0049003
Least	Audience outdegree/total outdegree	45	-0.0086335	0.0025320	-0.0147816	-0.0024854
Least	Audience outdegree/total outdegree	47	-0.0197709	NA	NA	NA
Least	Audience outdegree/total outdegree	48	-0.0146572	NA	NA	NA
Least	Audience outdegree/total outdegree	49	-0.0145026	NA	NA	NA
Least	Audience outdegree/total outdegree	50	-0.0165984	NA	NA	NA
Least	Audience outdegree/total outdegree	51	-0.0113113	0.0048550	-0.0230999	0.0004774
Least	Audience outdegree/total outdegree	52	-0.0103792	0.0055201	-0.0237831	0.0030246
Least	Audience outdegree/total outdegree	53	-0.0144723	0.0015017	-0.0181188	-0.0108258
Least	Audience outdegree/total outdegree	54	-0.0113703	0.0020728	-0.0164035	-0.0063371
Least	Audience outdegree/total outdegree	55	-0.0121561	0.0094281	-0.0350491	0.0107369
Least	Audience outdegree/total outdegree	56	-0.0099101	0.0018143	-0.0143155	-0.0055046
Least	Audience outdegree/total outdegree	57	-0.0089979	0.0032195	-0.0168154	-0.0011804
Least	Audience outdegree/total outdegree	58	-0.0118440	0.0011777	-0.0147037	-0.0089843
Least	Audience outdegree/total outdegree	59	-0.0135775	0.0079189	-0.0328058	0.0056508

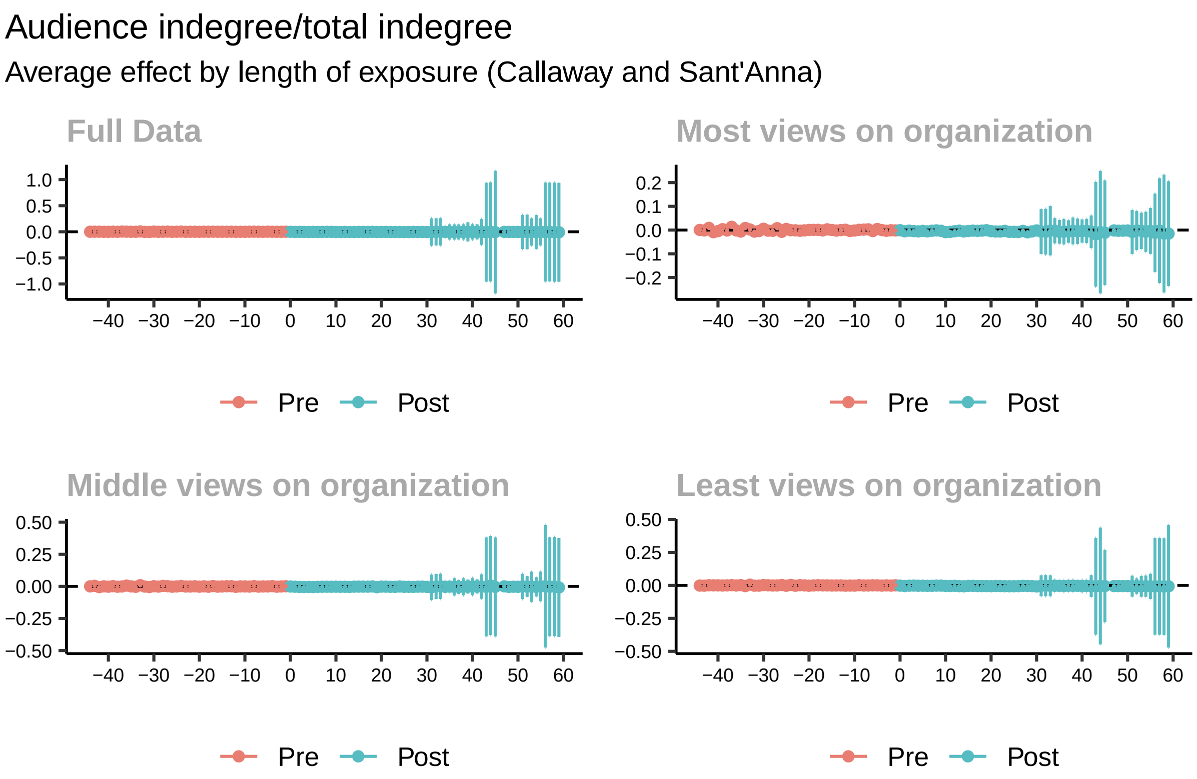

Long timeframe with sampled control groups

sample	outcome	event.time	estimate	std.error	conf.low	conf.high
Full	Audience indegree/total indegree	-44	-0.0001645	0.0004271	-0.0012667	0.0009377
Full	Audience indegree/total indegree	-43	0.0005147	0.0006974	-0.0012852	0.0023147
Full	Audience indegree/total indegree	-42	0.0007051	0.0007779	-0.0013025	0.0027127
Full	Audience indegree/total indegree	-41	-0.0016197	0.0042918	-0.0126962	0.0094567
Full	Audience indegree/total indegree	-40	-0.0016810	0.0049865	-0.0145505	0.0111884
Full	Audience indegree/total indegree	-39	0.0012926	0.0039827	-0.0089861	0.0115714
Full	Audience indegree/total indegree	-38	-0.0011122	0.0035515	-0.0102780	0.0080535
Full	Audience indegree/total indegree	-37	0.0038805	0.0010959	0.0010522	0.0067088
Full	Audience indegree/total indegree	-36	0.0017136	0.0004522	0.0005464	0.0028807
Full	Audience indegree/total indegree	-35	-0.0004525	0.0014139	-0.0041017	0.0031967
Full	Audience indegree/total indegree	-34	-0.0011843	0.0015051	-0.0050687	0.0027000
Full	Audience indegree/total indegree	-33	0.0063244	0.0010694	0.0035644	0.0090843
Full	Audience indegree/total indegree	-32	-0.0026553	0.0023255	-0.0086571	0.0033466
Full	Audience indegree/total indegree	-31	-0.0036227	0.0025024	-0.0100809	0.0028356
Full	Audience indegree/total indegree	-30	0.0028877	0.0008317	0.0007413	0.0050341
Full	Audience indegree/total indegree	-29	-0.0008067	0.0005296	-0.0021736	0.0005602
Full	Audience indegree/total indegree	-28	0.0003893	0.0006689	-0.0013370	0.0021156
Full	Audience indegree/total indegree	-27	0.0024197	0.0017959	-0.0022151	0.0070545
Full	Audience indegree/total indegree	-26	-0.0013661	0.0017522	-0.0058882	0.0031560

Full	Audience indegree/total indegree	-25	-0.0000748	0.0009640	-0.0025627	0.0024132
Full	Audience indegree/total indegree	-24	0.0024984	0.0011468	-0.0004614	0.0054581
Full	Audience indegree/total indegree	-23	-0.0013101	0.0006014	-0.0028622	0.0002420
Full	Audience indegree/total indegree	-22	-0.0002606	0.0005350	-0.0016415	0.0011202
Full	Audience indegree/total indegree	-21	0.0000439	0.0005938	-0.0014885	0.0015764
Full	Audience indegree/total indegree	-20	-0.0015356	0.0009094	-0.0038826	0.0008114
Full	Audience indegree/total indegree	-19	0.0003816	0.0008840	-0.0019000	0.0026632
Full	Audience indegree/total indegree	-18	-0.0001927	0.0010591	-0.0029261	0.0025408
Full	Audience indegree/total indegree	-17	0.0008291	0.0006409	-0.0008251	0.0024832
Full	Audience indegree/total indegree	-16	0.0000611	0.0006523	-0.0016225	0.0017446
Full	Audience indegree/total indegree	-15	-0.0003342	0.0004498	-0.0014951	0.0008268
Full	Audience indegree/total indegree	-14	-0.0001765	0.0010236	-0.0028182	0.0024652
Full	Audience indegree/total indegree	-13	0.0005212	0.0011909	-0.0025524	0.0035948
Full	Audience indegree/total indegree	-12	-0.0016230	0.0017129	-0.0060436	0.0027976
Full	Audience indegree/total indegree	-11	-0.0011933	0.0016693	-0.0055016	0.0031150
Full	Audience indegree/total indegree	-10	-0.0012586	0.0006366	-0.0029016	0.0003843
Full	Audience indegree/total indegree	-9	0.0004376	0.0007643	-0.0015350	0.0024102
Full	Audience indegree/total indegree	-8	0.0009977	0.0008071	-0.0010854	0.0030808
Full	Audience indegree/total indegree	-7	-0.0000432	0.0004725	-0.0012627	0.0011762
Full	Audience indegree/total indegree	-6	-0.0007500	0.0010641	-0.0034963	0.0019963
Full	Audience indegree/total indegree	-5	0.0011610	0.0006037	-0.0003970	0.0027190
Full	Audience indegree/total indegree	-4	0.0001543	0.0009769	-0.0023669	0.0026755
Full	Audience indegree/total indegree	-3	-0.0014389	0.0009139	-0.0037976	0.0009199
Full	Audience indegree/total indegree	-2	-0.0006485	0.0007800	-0.0026616	0.0013647
Full	Audience indegree/total indegree	-1	0.0006059	0.0003132	-0.0002025	0.0014142
Full	Audience indegree/total indegree	0	-0.0012083	0.0007473	-0.0031369	0.0007203
Full	Audience indegree/total indegree	1	-0.0040756	0.0005881	-0.0055935	-0.0025577
Full	Audience indegree/total indegree	2	-0.0032244	0.0007218	-0.0050874	-0.0013614
Full	Audience indegree/total indegree	3	-0.0034805	0.0010922	-0.0062994	-0.0006616
Full	Audience indegree/total indegree	4	-0.0040579	0.0012887	-0.0073838	-0.0007320
Full	Audience indegree/total indegree	5	-0.0037243	0.0012139	-0.0068573	-0.0005913
Full	Audience indegree/total indegree	6	-0.0041507	0.0005233	-0.0055012	-0.0028001
Full	Audience indegree/total indegree	7	-0.0031413	0.0009096	-0.0054889	-0.0007938
Full	Audience indegree/total indegree	8	-0.0020836	0.0012448	-0.0052963	0.0011291
Full	Audience indegree/total indegree	9	-0.0024625	0.0006818	-0.0042222	-0.0007028
Full	Audience indegree/total indegree	10	-0.0045353	0.0007158	-0.0063828	-0.0026879
Full	Audience indegree/total indegree	11	-0.0048600	0.0010479	-0.0075645	-0.0021554
Full	Audience indegree/total indegree	12	-0.0035975	0.0013777	-0.0071531	-0.0000419
Full	Audience indegree/total indegree	13	-0.0043350	0.0005020	-0.0056305	-0.0030395
Full	Audience indegree/total indegree	14	-0.0045437	0.0004757	-0.0057715	-0.0033159
Full	Audience indegree/total indegree	15	-0.0031003	0.0004494	-0.0042601	-0.0019405

Full	Audience indegree/total indegree	16	-0.0025869	0.0013115	-0.0059718	0.0007980
Full	Audience indegree/total indegree	17	-0.0033410	0.0029526	-0.0109612	0.0042793
Full	Audience indegree/total indegree	18	-0.0025055	0.0018107	-0.0071786	0.0021677
Full	Audience indegree/total indegree	19	-0.0045098	0.0009828	-0.0070462	-0.0019734
Full	Audience indegree/total indegree	20	-0.0039436	0.0006510	-0.0056236	-0.0022635
Full	Audience indegree/total indegree	21	-0.0037696	0.0009222	-0.0061496	-0.0013897
Full	Audience indegree/total indegree	22	-0.0045994	0.0009768	-0.0071204	-0.0020784
Full	Audience indegree/total indegree	23	-0.0044688	0.0006408	-0.0061226	-0.0028149
Full	Audience indegree/total indegree	24	-0.0043554	0.0005305	-0.0057245	-0.0029863
Full	Audience indegree/total indegree	25	-0.0053702	0.0009474	-0.0078152	-0.0029252
Full	Audience indegree/total indegree	26	-0.0051345	0.0009508	-0.0075882	-0.0026807
Full	Audience indegree/total indegree	27	-0.0037626	0.0009277	-0.0061567	-0.0013685
Full	Audience indegree/total indegree	28	-0.0047330	0.0013806	-0.0082961	-0.0011698
Full	Audience indegree/total indegree	29	-0.0042077	0.0036686	-0.0136759	0.0052605
Full	Audience indegree/total indegree	30	-0.0053313	0.0042619	-0.0163307	0.0056681
Full	Audience indegree/total indegree	31	-0.0051174	0.1008061	-0.2652826	0.2550477
Full	Audience indegree/total indegree	32	-0.0025203	0.1008061	-0.2626855	0.2576448
Full	Audience indegree/total indegree	33	-0.0014940	0.1008061	-0.2616592	0.2586711
Full	Audience indegree/total indegree	34	-0.0027743	0.0414323	-0.1097048	0.1041561
Full	Audience indegree/total indegree	35	-0.0036124	0.0564288	-0.1492465	0.1420217
Full	Audience indegree/total indegree	36	-0.0047488	0.0563342	-0.1501387	0.1406411
Full	Audience indegree/total indegree	37	-0.0041389	0.0563116	-0.1494705	0.1411927
Full	Audience indegree/total indegree	38	-0.0030381	0.0563318	-0.1484220	0.1423457
Full	Audience indegree/total indegree	39	-0.0028201	0.0711830	-0.1865325	0.1808924
Full	Audience indegree/total indegree	40	-0.0034416	0.0562318	-0.1485673	0.1416842
Full	Audience indegree/total indegree	41	-0.0025069	0.0562982	-0.1478041	0.1427903
Full	Audience indegree/total indegree	42	-0.0040936	0.0948915	-0.2489941	0.2408068
Full	Audience indegree/total indegree	43	-0.0090453	0.3672407	-0.9568377	0.9387471
Full	Audience indegree/total indegree	44	-0.0018591	0.3672407	-0.9496514	0.9459333
Full	Audience indegree/total indegree	45	-0.0064059	0.4546831	-1.1798740	1.1670622
Full	Audience indegree/total indegree	47	-0.0021325	NA	NA	NA
Full	Audience indegree/total indegree	48	-0.0041816	NA	NA	NA
Full	Audience indegree/total indegree	49	-0.0036928	NA	NA	NA
Full	Audience indegree/total indegree	50	-0.0038028	NA	NA	NA
Full	Audience indegree/total indegree	51	-0.0041758	0.1256002	-0.3283308	0.3199791
Full	Audience indegree/total indegree	52	-0.0028071	0.1282972	-0.3339227	0.3283085
Full	Audience indegree/total indegree	53	-0.0045058	0.1008061	-0.2646710	0.2556593
Full	Audience indegree/total indegree	54	-0.0051158	0.1264310	-0.3314149	0.3211833
Full	Audience indegree/total indegree	55	-0.0035512	0.1008061	-0.2637163	0.2566140
Full	Audience indegree/total indegree	56	-0.0056416	0.3672407	-0.9534340	0.9421507
Full	Audience indegree/total indegree	57	-0.0040510	0.3672407	-0.9518434	0.9437414

Full	Audience indegree/total indegree	58	-0.0080507	0.3672407	-0.9558431	0.9397417
Full	Audience indegree/total indegree	59	-0.0097282	0.3672407	-0.9575205	0.9380642

sample	outcome	event.time	estimate	std.error	conf.low	conf.high
Most	Audience indegree/total indegree	-44	0.0004794	0.0022392	-0.0053918	0.0063507
Most	Audience indegree/total indegree	-43	-0.0014375	0.0022342	-0.0072957	0.0044207
Most	Audience indegree/total indegree	-42	0.0090355	0.0020783	0.0035860	0.0144850
Most	Audience indegree/total indegree	-41	-0.0088141	0.0040316	-0.0193853	0.0017571
Most	Audience indegree/total indegree	-40	-0.0044285	0.0058474	-0.0197609	0.0109038
Most	Audience indegree/total indegree	-39	0.0035013	0.0047449	-0.0089403	0.0159429
Most	Audience indegree/total indegree	-38	-0.0015970	0.0034123	-0.0105444	0.0073505
Most	Audience indegree/total indegree	-37	0.0134967	0.0030940	0.0053840	0.0216095
Most	Audience indegree/total indegree	-36	-0.0013999	0.0024732	-0.0078849	0.0050850
Most	Audience indegree/total indegree	-35	-0.0061812	0.0024465	-0.0125961	0.0002338
Most	Audience indegree/total indegree	-34	0.0084525	0.0023861	0.0021959	0.0147091
Most	Audience indegree/total indegree	-33	0.0024875	0.0019745	-0.0026897	0.0076648
Most	Audience indegree/total indegree	-32	-0.0062995	0.0020958	-0.0117949	-0.0008041
Most	Audience indegree/total indegree	-31	-0.0040726	0.0024796	-0.0105743	0.0024292
Most	Audience indegree/total indegree	-30	0.0053423	0.0025369	-0.0013096	0.0119941
Most	Audience indegree/total indegree	-29	-0.0023786	0.0017463	-0.0069576	0.0022004
Most	Audience indegree/total indegree	-28	-0.0030346	0.0024891	-0.0095613	0.0034921
Most	Audience indegree/total indegree	-27	0.0077622	0.0021644	0.0020869	0.0134375
Most	Audience indegree/total indegree	-26	-0.0073072	0.0032746	-0.0158935	0.0012790
Most	Audience indegree/total indegree	-25	0.0045275	0.0020850	-0.0009397	0.0099946
Most	Audience indegree/total indegree	-24	-0.0007532	0.0014125	-0.0044570	0.0029505
Most	Audience indegree/total indegree	-23	-0.0010449	0.0033733	-0.0098899	0.0078001
Most	Audience indegree/total indegree	-22	-0.0028349	0.0018973	-0.0078099	0.0021401
Most	Audience indegree/total indegree	-21	-0.0012368	0.0014503	-0.0050396	0.0025660
Most	Audience indegree/total indegree	-20	0.0005364	0.0029775	-0.0072707	0.0083436
Most	Audience indegree/total indegree	-19	0.0007769	0.0020850	-0.0046901	0.0062440
Most	Audience indegree/total indegree	-18	0.0008247	0.0026613	-0.0061534	0.0078028
Most	Audience indegree/total indegree	-17	-0.0014459	0.0014273	-0.0051884	0.0022965
Most	Audience indegree/total indegree	-16	0.0030212	0.0011113	0.0001073	0.0059350
Most	Audience indegree/total indegree	-15	0.0004093	0.0006158	-0.0012053	0.0020239
Most	Audience indegree/total indegree	-14	-0.0020254	0.0010504	-0.0047796	0.0007288
Most	Audience indegree/total indegree	-13	-0.0001690	0.0025089	-0.0067476	0.0064095
Most	Audience indegree/total indegree	-12	0.0013457	0.0019709	-0.0038222	0.0065135
Most	Audience indegree/total indegree	-11	-0.0039206	0.0016378	-0.0082152	0.0003740
Most	Audience indegree/total indegree	-10	-0.0019314	0.0011313	-0.0048977	0.0010349
Most	Audience indegree/total indegree	-9	0.0009441	0.0026807	-0.0060849	0.0079731
Most	Audience indegree/total indegree	-8	0.0018627	0.0029332	-0.0058285	0.0095539

Most	Audience indegree/total indegree	-7	0.0030254	0.0013981	-0.0006404	0.0066913
Most	Audience indegree/total indegree	-6	-0.0042441	0.0021503	-0.0098824	0.0013942
Most	Audience indegree/total indegree	-5	0.0044430	0.0023881	-0.0018188	0.0107049
Most	Audience indegree/total indegree	-4	-0.0001906	0.0019604	-0.0053309	0.0049497
Most	Audience indegree/total indegree	-3	-0.0029652	0.0021072	-0.0084904	0.0025600
Most	Audience indegree/total indegree	-2	-0.0008257	0.0018847	-0.0057676	0.0041161
Most	Audience indegree/total indegree	-1	-0.0015187	0.0010384	-0.0042416	0.0012041
Most	Audience indegree/total indegree	0	-0.0005580	0.0014130	-0.0042630	0.0031471
Most	Audience indegree/total indegree	1	-0.0051160	0.0008255	-0.0072807	-0.0029514
Most	Audience indegree/total indegree	2	-0.0026369	0.0014372	-0.0064054	0.0011317
Most	Audience indegree/total indegree	3	-0.0043973	0.0020053	-0.0096553	0.0008607
Most	Audience indegree/total indegree	4	-0.0053911	0.0025263	-0.0120154	0.0012332
Most	Audience indegree/total indegree	5	-0.0034816	0.0021915	-0.0092278	0.0022646
Most	Audience indegree/total indegree	6	-0.0054155	0.0009803	-0.0079860	-0.0028450
Most	Audience indegree/total indegree	7	-0.0030108	0.0013689	-0.0066003	0.0005787
Most	Audience indegree/total indegree	8	-0.0013463	0.0025240	-0.0079645	0.0052719
Most	Audience indegree/total indegree	9	-0.0031207	0.0009438	-0.0055956	-0.0006459
Most	Audience indegree/total indegree	10	-0.0085923	0.0015295	-0.0126027	-0.0045819
Most	Audience indegree/total indegree	11	-0.0076330	0.0015110	-0.0115951	-0.0036710
Most	Audience indegree/total indegree	12	-0.0048940	0.0018156	-0.0096547	-0.0001333
Most	Audience indegree/total indegree	13	-0.0026748	0.0021482	-0.0083075	0.0029578
Most	Audience indegree/total indegree	14	-0.0056796	0.0016711	-0.0100613	-0.0012979
Most	Audience indegree/total indegree	15	-0.0040114	0.0025827	-0.0107835	0.0027607
Most	Audience indegree/total indegree	16	-0.0025145	0.0032053	-0.0109190	0.0058900
Most	Audience indegree/total indegree	17	-0.0034009	0.0036936	-0.0130858	0.0062839
Most	Audience indegree/total indegree	18	-0.0024226	0.0028964	-0.0100171	0.0051719
Most	Audience indegree/total indegree	19	-0.0006572	0.0021069	-0.0061817	0.0048674
Most	Audience indegree/total indegree	20	-0.0046194	0.0014751	-0.0084872	-0.0007517
Most	Audience indegree/total indegree	21	-0.0060548	0.0020610	-0.0114589	-0.0006507
Most	Audience indegree/total indegree	22	-0.0060710	0.0024567	-0.0125127	0.0003706
Most	Audience indegree/total indegree	23	-0.0033149	0.0013405	-0.0068298	0.0002000
Most	Audience indegree/total indegree	24	-0.0075468	0.0010661	-0.0103423	-0.0047513
Most	Audience indegree/total indegree	25	-0.0075531	0.0018479	-0.0123984	-0.0027078
Most	Audience indegree/total indegree	26	-0.0082295	0.0016196	-0.0124763	-0.0039826
Most	Audience indegree/total indegree	27	-0.0035150	0.0014584	-0.0073392	0.0003091
Most	Audience indegree/total indegree	28	-0.0092260	0.0024527	-0.0156573	-0.0027947
Most	Audience indegree/total indegree	29	-0.0056695	0.0056339	-0.0204422	0.0091032
Most	Audience indegree/total indegree	30	-0.0015887	0.0063865	-0.0183347	0.0151573
Most	Audience indegree/total indegree	31	-0.0063487	0.0359697	-0.1006644	0.0879670
Most	Audience indegree/total indegree	32	-0.0069812	0.0366716	-0.1031373	0.0891749
Most	Audience indegree/total indegree	33	-0.0027688	0.0396040	-0.1066140	0.1010764

Most	Audience indegree/total indegree	34	-0.0028618	0.0201348	-0.0556569	0.0499334
Most	Audience indegree/total indegree	35	-0.0073306	0.0190198	-0.0572021	0.0425409
Most	Audience indegree/total indegree	36	-0.0068472	0.0203297	-0.0601535	0.0464591
Most	Audience indegree/total indegree	37	-0.0061368	0.0179184	-0.0531203	0.0408468
Most	Audience indegree/total indegree	38	-0.0035260	0.0215697	-0.0600838	0.0530317
Most	Audience indegree/total indegree	39	-0.0050061	0.0202534	-0.0581124	0.0481001
Most	Audience indegree/total indegree	40	-0.0040708	0.0186191	-0.0528917	0.0447500
Most	Audience indegree/total indegree	41	-0.0036992	0.0193143	-0.0543430	0.0469446
Most	Audience indegree/total indegree	42	-0.0072364	0.0262403	-0.0760409	0.0615681
Most	Audience indegree/total indegree	43	-0.0177611	0.0840328	-0.2381026	0.2025805
Most	Audience indegree/total indegree	44	-0.0085452	0.0983440	-0.2664119	0.2493215
Most	Audience indegree/total indegree	45	-0.0112628	0.0840328	-0.2316044	0.2090788
Most	Audience indegree/total indegree	47	-0.0015964	NA	NA	NA
Most	Audience indegree/total indegree	48	-0.0028268	NA	NA	NA
Most	Audience indegree/total indegree	49	-0.0026322	NA	NA	NA
Most	Audience indegree/total indegree	50	-0.0017928	NA	NA	NA
Most	Audience indegree/total indegree	51	-0.0079637	0.0353076	-0.1005434	0.0846160
Most	Audience indegree/total indegree	52	-0.0019482	0.0312942	-0.0840045	0.0801080
Most	Audience indegree/total indegree	53	-0.0033866	0.0291929	-0.0799331	0.0731598
Most	Audience indegree/total indegree	54	-0.0073077	0.0320927	-0.0914577	0.0768422
Most	Audience indegree/total indegree	55	-0.0035560	0.0368208	-0.1001034	0.0929913
Most	Audience indegree/total indegree	56	-0.0114865	0.0628844	-0.1763750	0.1534019
Most	Audience indegree/total indegree	57	-0.0024069	0.0840328	-0.2227485	0.2179346
Most	Audience indegree/total indegree	58	-0.0151060	0.0945226	-0.2629526	0.2327406
Most	Audience indegree/total indegree	59	-0.0148555	0.0840328	-0.2351971	0.2054860

sample	outcome	event.time	estimate	std.error	conf.low	conf.high
Middle	Audience indegree/total indegree	-44	0.0005331	0.0023996	-0.0060865	0.0071527
Middle	Audience indegree/total indegree	-43	0.0042504	0.0033447	-0.0049763	0.0134772
Middle	Audience indegree/total indegree	-42	-0.0054050	0.0011568	-0.0085961	-0.0022139
Middle	Audience indegree/total indegree	-41	0.0004348	0.0043865	-0.0116659	0.0125355
Middle	Audience indegree/total indegree	-40	-0.0023181	0.0048018	-0.0155646	0.0109284
Middle	Audience indegree/total indegree	-39	0.0019809	0.0037455	-0.0083517	0.0123135
Middle	Audience indegree/total indegree	-38	-0.0024521	0.0037445	-0.0127818	0.0078775
Middle	Audience indegree/total indegree	-37	-0.0004375	0.0011799	-0.0036926	0.0028175
Middle	Audience indegree/total indegree	-36	0.0059844	0.0015850	0.0016120	0.0103568
Middle	Audience indegree/total indegree	-35	0.0003087	0.0021976	-0.0057538	0.0063712
Middle	Audience indegree/total indegree	-34	-0.0031303	0.0021600	-0.0090889	0.0028284
Middle	Audience indegree/total indegree	-33	0.0093070	0.0010263	0.0064757	0.0121383
Middle	Audience indegree/total indegree	-32	-0.0012401	0.0022552	-0.0074615	0.0049812
Middle	Audience indegree/total indegree	-31	-0.0049376	0.0024060	-0.0115749	0.0016997

Middle	Audience indegree/total indegree	-30	0.0015719	0.0012675	-0.0019246	0.0050685
Middle	Audience indegree/total indegree	-29	-0.0017187	0.0014634	-0.0057556	0.0023182
Middle	Audience indegree/total indegree	-28	0.0037316	0.0010501	0.0008348	0.0066284
Middle	Audience indegree/total indegree	-27	0.0016196	0.0017151	-0.0031117	0.0063508
Middle	Audience indegree/total indegree	-26	-0.0022444	0.0021196	-0.0080915	0.0036027
Middle	Audience indegree/total indegree	-25	-0.0003715	0.0018994	-0.0056112	0.0048682
Middle	Audience indegree/total indegree	-24	0.0031334	0.0012721	-0.0003758	0.0066426
Middle	Audience indegree/total indegree	-23	-0.0005164	0.0008531	-0.0028699	0.0018370
Middle	Audience indegree/total indegree	-22	-0.0007732	0.0009739	-0.0034598	0.0019133
Middle	Audience indegree/total indegree	-21	0.0008016	0.0013121	-0.0028180	0.0044212
Middle	Audience indegree/total indegree	-20	-0.0021622	0.0013660	-0.0059304	0.0016060
Middle	Audience indegree/total indegree	-19	0.0006650	0.0009321	-0.0019062	0.0032362
Middle	Audience indegree/total indegree	-18	-0.0027434	0.0015543	-0.0070311	0.0015442
Middle	Audience indegree/total indegree	-17	0.0026927	0.0016593	-0.0018848	0.0072701
Middle	Audience indegree/total indegree	-16	-0.0018229	0.0011080	-0.0048794	0.0012335
Middle	Audience indegree/total indegree	-15	-0.0003964	0.0009649	-0.0030583	0.0022654
Middle	Audience indegree/total indegree	-14	0.0010854	0.0012442	-0.0023469	0.0045176
Middle	Audience indegree/total indegree	-13	0.0017887	0.0020257	-0.0037996	0.0073769
Middle	Audience indegree/total indegree	-12	-0.0032294	0.0025807	-0.0103486	0.0038898
Middle	Audience indegree/total indegree	-11	0.0001295	0.0014872	-0.0039732	0.0042321
Middle	Audience indegree/total indegree	-10	-0.0000290	0.0018142	-0.0050338	0.0049758
Middle	Audience indegree/total indegree	-9	-0.0013201	0.0013129	-0.0049418	0.0023016
Middle	Audience indegree/total indegree	-8	0.0019263	0.0010408	-0.0009447	0.0047974
Middle	Audience indegree/total indegree	-7	-0.0016454	0.0015750	-0.0059904	0.0026996
Middle	Audience indegree/total indegree	-6	-0.0003604	0.0012827	-0.0038990	0.0031782
Middle	Audience indegree/total indegree	-5	-0.0001252	0.0007572	-0.0022140	0.0019635
Middle	Audience indegree/total indegree	-4	0.0018880	0.0008213	-0.0003776	0.0041537
Middle	Audience indegree/total indegree	-3	-0.0022157	0.0016463	-0.0067573	0.0023259
Middle	Audience indegree/total indegree	-2	-0.0002151	0.0010209	-0.0030315	0.0026013
Middle	Audience indegree/total indegree	-1	0.0013141	0.0011004	-0.0017216	0.0043498
Middle	Audience indegree/total indegree	0	-0.0007684	0.0013397	-0.0044642	0.0029275
Middle	Audience indegree/total indegree	1	-0.0024885	0.0013240	-0.0061411	0.0011640
Middle	Audience indegree/total indegree	2	-0.0042641	0.0006727	-0.0061199	-0.0024084
Middle	Audience indegree/total indegree	3	-0.0048157	0.0012977	-0.0083956	-0.0012359
Middle	Audience indegree/total indegree	4	-0.0042659	0.0008477	-0.0066045	-0.0019273
Middle	Audience indegree/total indegree	5	-0.0050278	0.0008944	-0.0074953	-0.0025604
Middle	Audience indegree/total indegree	6	-0.0036568	0.0016672	-0.0082559	0.0009423
Middle	Audience indegree/total indegree	7	-0.0029470	0.0008214	-0.0052130	-0.0006809
Middle	Audience indegree/total indegree	8	-0.0027471	0.0007369	-0.0047799	-0.0007142
Middle	Audience indegree/total indegree	9	-0.0028110	0.0013082	-0.0064198	0.0007978
Middle	Audience indegree/total indegree	10	-0.0024234	0.0005049	-0.0038163	-0.0010305

Middle	Audience indegree/total indegree	11	-0.0033355	0.0011366	-0.0064711	-0.0001999
Middle	Audience indegree/total indegree	12	-0.0029974	0.0010973	-0.0060245	0.0000297
Middle	Audience indegree/total indegree	13	-0.0041679	0.0010744	-0.0071319	-0.0012040
Middle	Audience indegree/total indegree	14	-0.0020660	0.0005348	-0.0035413	-0.0005906
Middle	Audience indegree/total indegree	15	-0.0015757	0.0016124	-0.0060237	0.0028723
Middle	Audience indegree/total indegree	16	-0.0017867	0.0019079	-0.0070498	0.0034764
Middle	Audience indegree/total indegree	17	-0.0021026	0.0021468	-0.0080248	0.0038197
Middle	Audience indegree/total indegree	18	-0.0003821	0.0014568	-0.0044009	0.0036367
Middle	Audience indegree/total indegree	19	-0.0062775	0.0015790	-0.0106335	-0.0019215
Middle	Audience indegree/total indegree	20	-0.0015997	0.0010781	-0.0045737	0.0013743
Middle	Audience indegree/total indegree	21	-0.0019567	0.0009389	-0.0045467	0.0006334
Middle	Audience indegree/total indegree	22	-0.0033307	0.0008046	-0.0055502	-0.0011111
Middle	Audience indegree/total indegree	23	-0.0035786	0.0005372	-0.0050605	-0.0020967
Middle	Audience indegree/total indegree	24	-0.0005224	0.0013817	-0.0043340	0.0032892
Middle	Audience indegree/total indegree	25	-0.0033385	0.0008442	-0.0056673	-0.0010097
Middle	Audience indegree/total indegree	26	-0.0032836	0.0008495	-0.0056272	-0.0009400
Middle	Audience indegree/total indegree	27	-0.0038692	0.0009540	-0.0065010	-0.0012374
Middle	Audience indegree/total indegree	28	-0.0021885	0.0010662	-0.0051297	0.0007527
Middle	Audience indegree/total indegree	29	-0.0017809	0.0006639	-0.0036125	0.0000506
Middle	Audience indegree/total indegree	30	-0.0039078	0.0023346	-0.0103482	0.0025326
Middle	Audience indegree/total indegree	31	-0.0069966	0.0353223	-0.1044380	0.0904449
Middle	Audience indegree/total indegree	32	-0.0007210	0.0353223	-0.0981624	0.0967205
Middle	Audience indegree/total indegree	33	0.0004966	0.0353223	-0.0969448	0.0979381
Middle	Audience indegree/total indegree	34	-0.0030240	0.0164655	-0.0484464	0.0423983
Middle	Audience indegree/total indegree	35	-0.0016980	0.0158217	-0.0453444	0.0419483
Middle	Audience indegree/total indegree	36	-0.0035166	0.0242301	-0.0703588	0.0633256
Middle	Audience indegree/total indegree	37	-0.0042173	0.0198072	-0.0588583	0.0504238
Middle	Audience indegree/total indegree	38	-0.0028350	0.0237901	-0.0684633	0.0627933
Middle	Audience indegree/total indegree	39	-0.0009809	0.0198264	-0.0556749	0.0537130
Middle	Audience indegree/total indegree	40	-0.0016246	0.0239968	-0.0678231	0.0645739
Middle	Audience indegree/total indegree	41	-0.0002086	0.0198725	-0.0550297	0.0546124
Middle	Audience indegree/total indegree	42	-0.0010067	0.0342591	-0.0955153	0.0935020
Middle	Audience indegree/total indegree	43	-0.0035151	0.1397588	-0.3890596	0.3820295
Middle	Audience indegree/total indegree	44	0.0063806	0.1397588	-0.3791639	0.3919252
Middle	Audience indegree/total indegree	45	-0.0036130	0.1397588	-0.3891575	0.3819316
Middle	Audience indegree/total indegree	47	0.0005478	NA	NA	NA
Middle	Audience indegree/total indegree	48	-0.0040072	NA	NA	NA
Middle	Audience indegree/total indegree	49	-0.0026699	NA	NA	NA
Middle	Audience indegree/total indegree	50	-0.0031979	NA	NA	NA
Middle	Audience indegree/total indegree	51	-0.0005040	0.0353223	-0.0979454	0.0969375
Middle	Audience indegree/total indegree	52	-0.0007546	0.0291532	-0.0811779	0.0796687

Middle	Audience indegree/total indegree	53	-0.0025443	0.0423452	-0.1193595	0.1142709
Middle	Audience indegree/total indegree	54	-0.0037885	0.0271418	-0.0786631	0.0710861
Middle	Audience indegree/total indegree	55	0.0004900	0.0418357	-0.1149195	0.1158996
Middle	Audience indegree/total indegree	56	0.0008654	0.1729021	-0.4761095	0.4778404
Middle	Audience indegree/total indegree	57	-0.0029501	0.1397588	-0.3884946	0.3825945
Middle	Audience indegree/total indegree	58	-0.0012839	0.1397588	-0.3868284	0.3842607
Middle	Audience indegree/total indegree	59	-0.0080084	0.1397588	-0.3935529	0.3775362

sample	outcome	event.time	estimate	std.error	conf.low	conf.high
Least	Audience indegree/total indegree	-44	-0.0011566	0.0010468	-0.0037901	0.0014770
Least	Audience indegree/total indegree	-43	-0.0019388	0.0012538	-0.0050931	0.0012155
Least	Audience indegree/total indegree	-42	0.0015877	0.0005392	0.0002311	0.0029442
Least	Audience indegree/total indegree	-41	0.0007869	0.0041678	-0.0096982	0.0112720
Least	Audience indegree/total indegree	-40	0.0004398	0.0044957	-0.0108703	0.0117499
Least	Audience indegree/total indegree	-39	-0.0006002	0.0039001	-0.0104119	0.0092115
Least	Audience indegree/total indegree	-38	0.0004566	0.0032841	-0.0078055	0.0087186
Least	Audience indegree/total indegree	-37	0.0020656	0.0019784	-0.0029116	0.0070428
Least	Audience indegree/total indegree	-36	-0.0005630	0.0010758	-0.0032695	0.0021435
Least	Audience indegree/total indegree	-35	0.0023448	0.0018373	-0.0022775	0.0069671
Least	Audience indegree/total indegree	-34	-0.0050367	0.0020433	-0.0101771	0.0001038
Least	Audience indegree/total indegree	-33	0.0057315	0.0015951	0.0017186	0.0097444
Least	Audience indegree/total indegree	-32	-0.0020211	0.0026402	-0.0086632	0.0046209
Least	Audience indegree/total indegree	-31	-0.0018150	0.0025877	-0.0083249	0.0046949
Least	Audience indegree/total indegree	-30	0.0026692	0.0011615	-0.0002528	0.0055912
Least	Audience indegree/total indegree	-29	0.0011082	0.0008626	-0.0010619	0.0032783
Least	Audience indegree/total indegree	-28	-0.0005116	0.0005530	-0.0019028	0.0008796
Least	Audience indegree/total indegree	-27	-0.0000219	0.0016950	-0.0042863	0.0042424
Least	Audience indegree/total indegree	-26	0.0030953	0.0019817	-0.0018903	0.0080809
Least	Audience indegree/total indegree	-25	-0.0025500	0.0017511	-0.0069553	0.0018554
Least	Audience indegree/total indegree	-24	0.0037881	0.0016351	-0.0003253	0.0079016
Least	Audience indegree/total indegree	-23	-0.0024018	0.0013857	-0.0058879	0.0010843
Least	Audience indegree/total indegree	-22	0.0018842	0.0008024	-0.0001344	0.0039027
Least	Audience indegree/total indegree	-21	0.0002647	0.0008529	-0.0018810	0.0024104
Least	Audience indegree/total indegree	-20	-0.0023451	0.0012255	-0.0054280	0.0007379
Least	Audience indegree/total indegree	-19	0.0000248	0.0012279	-0.0030644	0.0031140
Least	Audience indegree/total indegree	-18	0.0014758	0.0015108	-0.0023249	0.0052765
Least	Audience indegree/total indegree	-17	0.0003730	0.0007379	-0.0014833	0.0022294
Least	Audience indegree/total indegree	-16	0.0002164	0.0008183	-0.0018423	0.0022750
Least	Audience indegree/total indegree	-15	-0.0006539	0.0006696	-0.0023385	0.0010307
Least	Audience indegree/total indegree	-14	-0.0003147	0.0010775	-0.0030254	0.0023960
Least	Audience indegree/total indegree	-13	-0.0000835	0.0011072	-0.0028689	0.0027019

Least	Audience indegree/total indegree	-12	-0.0019496	0.0017468	-0.0063443	0.0024450
Least	Audience indegree/total indegree	-11	-0.0009458	0.0020209	-0.0060299	0.0041383
Least	Audience indegree/total indegree	-10	-0.0015832	0.0006992	-0.0033424	0.0001759
Least	Audience indegree/total indegree	-9	0.0017145	0.0008486	-0.0004203	0.0038494
Least	Audience indegree/total indegree	-8	-0.0005295	0.0017278	-0.0048763	0.0038173
Least	Audience indegree/total indegree	-7	-0.0002609	0.0014719	-0.0039638	0.0034420
Least	Audience indegree/total indegree	-6	0.0008728	0.0014079	-0.0026692	0.0044148
Least	Audience indegree/total indegree	-5	0.0005000	0.0008169	-0.0015553	0.0025552
Least	Audience indegree/total indegree	-4	-0.0013584	0.0013325	-0.0047107	0.0019939
Least	Audience indegree/total indegree	-3	0.0002654	0.0016730	-0.0039434	0.0044743
Least	Audience indegree/total indegree	-2	-0.0012973	0.0011897	-0.0042904	0.0016957
Least	Audience indegree/total indegree	-1	0.0009231	0.0008408	-0.0011921	0.0030383
Least	Audience indegree/total indegree	0	-0.0017446	0.0018482	-0.0063941	0.0029049
Least	Audience indegree/total indegree	1	-0.0047395	0.0011243	-0.0075679	-0.0019111
Least	Audience indegree/total indegree	2	-0.0022860	0.0007955	-0.0042872	-0.0002849
Least	Audience indegree/total indegree	3	-0.0013159	0.0018750	-0.0060329	0.0034010
Least	Audience indegree/total indegree	4	-0.0027148	0.0013673	-0.0061547	0.0007251
Least	Audience indegree/total indegree	5	-0.0024885	0.0013856	-0.0059743	0.0009972
Least	Audience indegree/total indegree	6	-0.0036080	0.0017472	-0.0080036	0.0007875
Least	Audience indegree/total indegree	7	-0.0031553	0.0015584	-0.0070758	0.0007652
Least	Audience indegree/total indegree	8	-0.0016742	0.0014755	-0.0053863	0.0020378
Least	Audience indegree/total indegree	9	-0.0017059	0.0009480	-0.0040910	0.0006791
Least	Audience indegree/total indegree	10	-0.0041438	0.0014922	-0.0078977	-0.0003898
Least	Audience indegree/total indegree	11	-0.0046119	0.0011999	-0.0076306	-0.0015932
Least	Audience indegree/total indegree	12	-0.0033143	0.0017398	-0.0076914	0.0010627
Least	Audience indegree/total indegree	13	-0.0053250	0.0015408	-0.0092013	-0.0014486
Least	Audience indegree/total indegree	14	-0.0063621	0.0014213	-0.0099377	-0.0027865
Least	Audience indegree/total indegree	15	-0.0040476	0.0014803	-0.0077717	-0.0003236
Least	Audience indegree/total indegree	16	-0.0036253	0.0017625	-0.0080592	0.0008087
Least	Audience indegree/total indegree	17	-0.0046392	0.0035404	-0.0135459	0.0042676
Least	Audience indegree/total indegree	18	-0.0046997	0.0031339	-0.0125838	0.0031844
Least	Audience indegree/total indegree	19	-0.0049547	0.0017703	-0.0094085	-0.0005010
Least	Audience indegree/total indegree	20	-0.0056484	0.0013335	-0.0090032	-0.0022937
Least	Audience indegree/total indegree	21	-0.0042846	0.0014803	-0.0080086	-0.0005606
Least	Audience indegree/total indegree	22	-0.0046960	0.0016167	-0.0087633	-0.0006287
Least	Audience indegree/total indegree	23	-0.0058223	0.0013202	-0.0091436	-0.0025009
Least	Audience indegree/total indegree	24	-0.0060591	0.0012584	-0.0092250	-0.0028932
Least	Audience indegree/total indegree	25	-0.0059505	0.0017457	-0.0103423	-0.0015587
Least	Audience indegree/total indegree	26	-0.0054278	0.0015287	-0.0092736	-0.0015820
Least	Audience indegree/total indegree	27	-0.0035793	0.0011867	-0.0065648	-0.0005937
Least	Audience indegree/total indegree	28	-0.0043973	0.0018046	-0.0089374	0.0001427

Least	Audience indegree/total indegree	29	-0.0051735	0.0049469	-0.0176188	0.0072718
Least	Audience indegree/total indegree	30	-0.0076071	0.0040337	-0.0177550	0.0025408
Least	Audience indegree/total indegree	31	-0.0027514	0.0314941	-0.0819832	0.0764804
Least	Audience indegree/total indegree	32	-0.0020459	0.0314941	-0.0812776	0.0771859
Least	Audience indegree/total indegree	33	-0.0027474	0.0314941	-0.0819792	0.0764844
Least	Audience indegree/total indegree	34	-0.0024899	0.0181630	-0.0481839	0.0432040
Least	Audience indegree/total indegree	35	-0.0033947	0.0154889	-0.0423611	0.0355716
Least	Audience indegree/total indegree	36	-0.0047698	0.0179123	-0.0498330	0.0402934
Least	Audience indegree/total indegree	37	-0.0029727	0.0172637	-0.0464042	0.0404588
Least	Audience indegree/total indegree	38	-0.0029640	0.0183220	-0.0490580	0.0431300
Least	Audience indegree/total indegree	39	-0.0033684	0.0146613	-0.0402528	0.0335159
Least	Audience indegree/total indegree	40	-0.0048197	0.0187026	-0.0518711	0.0422316
Least	Audience indegree/total indegree	41	-0.0040336	0.0180954	-0.0495575	0.0414903
Least	Audience indegree/total indegree	42	-0.0049238	0.0326802	-0.0871397	0.0772920
Least	Audience indegree/total indegree	43	-0.0065813	0.1455977	-0.3728710	0.3597084
Least	Audience indegree/total indegree	44	-0.0043627	0.1757386	-0.4464798	0.4377544
Least	Audience indegree/total indegree	45	-0.0047155	0.1088214	-0.2784847	0.2690538
Least	Audience indegree/total indegree	47	-0.0047242	NA	NA	NA
Least	Audience indegree/total indegree	48	-0.0048113	NA	NA	NA
Least	Audience indegree/total indegree	49	-0.0049808	NA	NA	NA
Least	Audience indegree/total indegree	50	-0.0050477	NA	NA	NA
Least	Audience indegree/total indegree	51	-0.0057900	0.0314941	-0.0850217	0.0734418
Least	Audience indegree/total indegree	52	-0.0051554	0.0231779	-0.0634656	0.0531549
Least	Audience indegree/total indegree	53	-0.0068950	0.0313731	-0.0858224	0.0720325
Least	Audience indegree/total indegree	54	-0.0052996	0.0320080	-0.0858243	0.0752251
Least	Audience indegree/total indegree	55	-0.0073507	0.0379216	-0.1027525	0.0880511
Least	Audience indegree/total indegree	56	-0.0070654	0.1455977	-0.3733550	0.3592243
Least	Audience indegree/total indegree	57	-0.0068726	0.1455977	-0.3731623	0.3594171
Least	Audience indegree/total indegree	58	-0.0085735	0.1455977	-0.3748632	0.3577162
Least	Audience indegree/total indegree	59	-0.0065929	0.1845631	-0.4709104	0.4577246

Hateful non−audience outdegree

Average effect by length of exposure (Callaway and Sant'Anna)

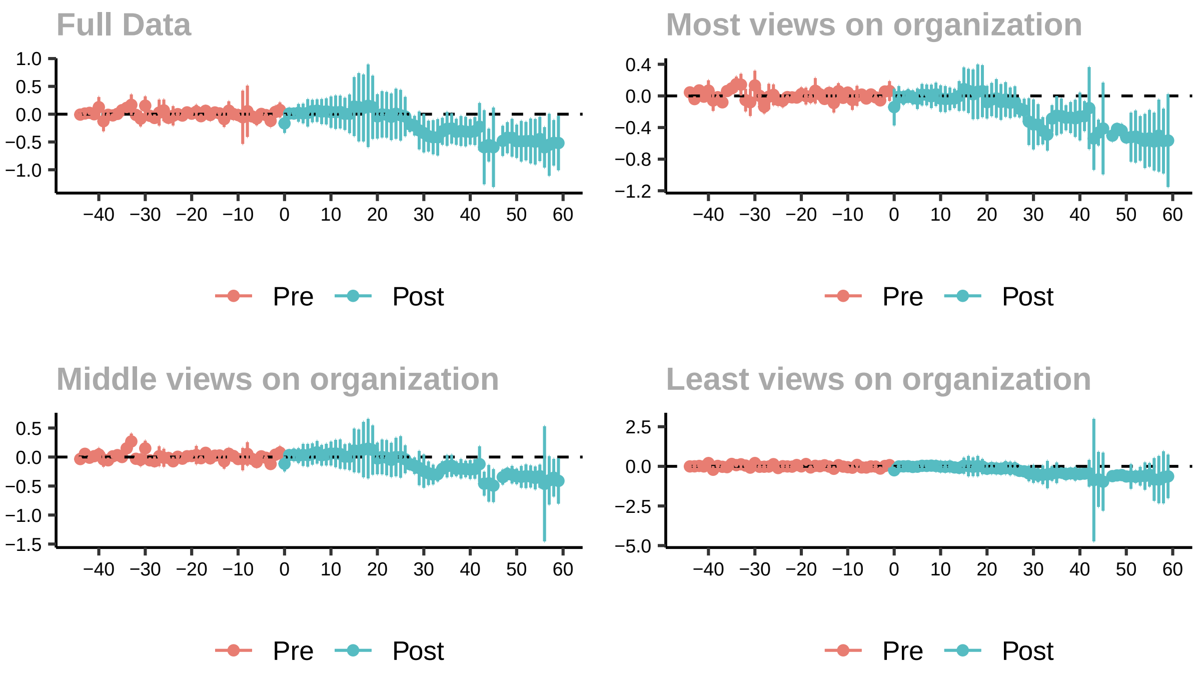

Long timeframe with sampled control groups

sample	outcome	event.time	estimate	std.error	conf.low	conf.high
Full	Hateful non-audience outdegree	-44	-0.0074997	0.0092556	-0.0321180	0.0171186
Full	Hateful non-audience outdegree	-43	0.0117591	0.0138476	-0.0250729	0.0485911
Full	Hateful non-audience outdegree	-42	0.0234647	0.0128138	-0.0106176	0.0575470
Full	Hateful non-audience outdegree	-41	-0.0013943	0.0149748	-0.0412245	0.0384360
Full	Hateful non-audience outdegree	-40	0.1257035	0.0687443	-0.0571436	0.3085507
Full	Hateful non-audience outdegree	-39	-0.1264571	0.0679175	-0.3071053	0.0541910
Full	Hateful non-audience outdegree	-38	-0.0115399	0.0154599	-0.0526605	0.0295807
Full	Hateful non-audience outdegree	-37	-0.0219639	0.0119140	-0.0536530	0.0097252
Full	Hateful non-audience outdegree	-36	0.0047796	0.0181834	-0.0435849	0.0531441
Full	Hateful non-audience outdegree	-35	0.0716713	0.0202255	0.0178752	0.1254674
Full	Hateful non-audience outdegree	-34	0.1125919	0.0352736	0.0187706	0.2064132
Full	Hateful non-audience outdegree	-33	0.1676575	0.0702067	-0.0190793	0.3543943
Full	Hateful non-audience outdegree	-32	-0.0136568	0.0379178	-0.1145111	0.0871974
Full	Hateful non-audience outdegree	-31	-0.0810827	0.0519842	-0.2193511	0.0571857
Full	Hateful non-audience outdegree	-30	0.1524454	0.0627831	-0.0145461	0.3194369
Full	Hateful non-audience outdegree	-29	-0.0297301	0.0230063	-0.0909226	0.0314624
Full	Hateful non-audience outdegree	-28	-0.0682782	0.0251207	-0.1350947	-0.0014618
Full	Hateful non-audience outdegree	-27	0.0284753	0.0878423	-0.2051689	0.2621195
Full	Hateful non-audience outdegree	-26	0.0681954	0.0732530	-0.1266441	0.2630349

Full	Hateful non-audience outdegree	-25	-0.0659563	0.0354992	-0.1603775	0.0284650
Full	Hateful non-audience outdegree	-24	-0.0290637	0.0649252	-0.2017526	0.1436253
Full	Hateful non-audience outdegree	-23	0.0005943	0.0162660	-0.0426702	0.0438588
Full	Hateful non-audience outdegree	-22	-0.0251616	0.0217600	-0.0830391	0.0327159
Full	Hateful non-audience outdegree	-21	0.0316543	0.0205707	-0.0230601	0.0863687
Full	Hateful non-audience outdegree	-20	0.0087402	0.0248739	-0.0574197	0.0749000
Full	Hateful non-audience outdegree	-19	0.0452574	0.0459112	-0.0768578	0.1673726
Full	Hateful non-audience outdegree	-18	-0.0365075	0.0162454	-0.0797174	0.0067023
Full	Hateful non-audience outdegree	-17	0.0605720	0.0328689	-0.0268533	0.1479973
Full	Hateful non-audience outdegree	-16	-0.0145084	0.0386222	-0.1172364	0.0882196
Full	Hateful non-audience outdegree	-15	0.0301041	0.0253868	-0.0374200	0.0976282
Full	Hateful non-audience outdegree	-14	0.0141445	0.0286236	-0.0619890	0.0902781
Full	Hateful non-audience outdegree	-13	-0.0882011	0.0529774	-0.2291113	0.0527090
Full	Hateful non-audience outdegree	-12	0.0630389	0.0627410	-0.1038406	0.2299184
Full	Hateful non-audience outdegree	-11	0.0012091	0.0380819	-0.1000818	0.1025000
Full	Hateful non-audience outdegree	-10	-0.0164536	0.0366290	-0.1138800	0.0809729
Full	Hateful non-audience outdegree	-9	-0.0557382	0.1795923	-0.5334207	0.4219443
Full	Hateful non-audience outdegree	-8	0.0531483	0.1732119	-0.4075634	0.5138601
Full	Hateful non-audience outdegree	-7	-0.0401718	0.0305715	-0.1214863	0.0411427
Full	Hateful non-audience outdegree	-6	-0.0756305	0.0464856	-0.1992735	0.0480125
Full	Hateful non-audience outdegree	-5	0.0040862	0.0286491	-0.0721152	0.0802875
Full	Hateful non-audience outdegree	-4	-0.0169293	0.0205099	-0.0714819	0.0376234
Full	Hateful non-audience outdegree	-3	-0.1196934	0.0422076	-0.2319579	-0.0074288
Full	Hateful non-audience outdegree	-2	0.0394984	0.0405116	-0.0682549	0.1472518
Full	Hateful non-audience outdegree	-1	0.0755239	0.0507121	-0.0593610	0.2104088
Full	Hateful non-audience outdegree	0	-0.1653384	0.0639846	-0.3355256	0.0048487
Full	Hateful non-audience outdegree	1	0.0111115	0.0375455	-0.0887526	0.1109756
Full	Hateful non-audience outdegree	2	0.0116489	0.0327966	-0.0755839	0.0988817
Full	Hateful non-audience outdegree	3	0.0237736	0.0598510	-0.1354190	0.1829661
Full	Hateful non-audience outdegree	4	0.0132410	0.0665191	-0.1636876	0.1901697
Full	Hateful non-audience outdegree	5	0.0225739	0.0923791	-0.2231375	0.2682854
Full	Hateful non-audience outdegree	6	0.0557041	0.0757668	-0.1458216	0.2572299
Full	Hateful non-audience outdegree	7	0.0643391	0.0756390	-0.1368468	0.2655249
Full	Hateful non-audience outdegree	8	0.0467387	0.0847740	-0.1787445	0.2722219
Full	Hateful non-audience outdegree	9	0.0501350	0.0878797	-0.1836089	0.2838789
Full	Hateful non-audience outdegree	10	0.0368251	0.1056256	-0.2441196	0.3177697
Full	Hateful non-audience outdegree	11	0.0357110	0.1127223	-0.2641096	0.3355317
Full	Hateful non-audience outdegree	12	0.0394285	0.1106675	-0.2549267	0.3337838
Full	Hateful non-audience outdegree	13	0.0054554	0.1084326	-0.2829555	0.2938662
Full	Hateful non-audience outdegree	14	0.0062084	0.1307360	-0.3415253	0.3539422
Full	Hateful non-audience outdegree	15	0.1374180	0.1993028	-0.3926906	0.6675266

Full	Hateful non-audience outdegree	16	0.1250800	0.2308032	-0.4888140	0.7389740
Full	Hateful non-audience outdegree	17	0.1118301	0.2263472	-0.4902116	0.7138719
Full	Hateful non-audience outdegree	18	0.1516580	0.2792599	-0.5911218	0.8944379
Full	Hateful non-audience outdegree	19	0.1244204	0.2135128	-0.4434844	0.6923251
Full	Hateful non-audience outdegree	20	-0.0405198	0.1499898	-0.4394650	0.3584253
Full	Hateful non-audience outdegree	21	-0.0029239	0.1563809	-0.4188682	0.4130204
Full	Hateful non-audience outdegree	22	-0.0115737	0.1545309	-0.4225974	0.3994499
Full	Hateful non-audience outdegree	23	-0.0472170	0.1576690	-0.4665875	0.3721535
Full	Hateful non-audience outdegree	24	0.0081661	0.1692863	-0.4421043	0.4584365
Full	Hateful non-audience outdegree	25	-0.0198341	0.1714592	-0.4758839	0.4362158
Full	Hateful non-audience outdegree	26	-0.0426998	0.1327391	-0.3957613	0.3103617
Full	Hateful non-audience outdegree	27	-0.1825020	0.0517068	-0.3200326	-0.0449715
Full	Hateful non-audience outdegree	28	-0.2069990	0.0661194	-0.3828644	-0.0311335
Full	Hateful non-audience outdegree	29	-0.2919561	0.1276948	-0.6316007	0.0476886
Full	Hateful non-audience outdegree	30	-0.3395595	0.1306805	-0.6871456	0.0080266
Full	Hateful non-audience outdegree	31	-0.3954700	0.1040125	-0.6721241	-0.1188159
Full	Hateful non-audience outdegree	32	-0.4148502	0.1149369	-0.7205612	-0.1091392
Full	Hateful non-audience outdegree	33	-0.4157136	0.1230842	-0.7430950	-0.0883323
Full	Hateful non-audience outdegree	34	-0.3078289	0.0908632	-0.5495082	-0.0661495
Full	Hateful non-audience outdegree	35	-0.2643952	0.1141359	-0.5679755	0.0391852
Full	Hateful non-audience outdegree	36	-0.2570465	0.1025408	-0.5297862	0.0156932
Full	Hateful non-audience outdegree	37	-0.3171333	0.0875937	-0.5501163	-0.0841503
Full	Hateful non-audience outdegree	38	-0.3017268	0.1009050	-0.5701157	-0.0333380
Full	Hateful non-audience outdegree	39	-0.3132248	0.0995326	-0.5779632	-0.0484864
Full	Hateful non-audience outdegree	40	-0.3191493	0.0853520	-0.5461699	-0.0921287
Full	Hateful non-audience outdegree	41	-0.3039282	0.0941805	-0.5544309	-0.0534255
Full	Hateful non-audience outdegree	42	-0.2259941	0.1605619	-0.6530591	0.2010709
Full	Hateful non-audience outdegree	43	-0.5950471	0.2499706	-1.2599228	0.0698285
Full	Hateful non-audience outdegree	44	-0.5561160	0.1108705	-0.8510111	-0.2612208
Full	Hateful non-audience outdegree	45	-0.5946890	0.2684453	-1.3087041	0.1193261
Full	Hateful non-audience outdegree	47	-0.4799257	0.1015231	-0.7499584	-0.2098930
Full	Hateful non-audience outdegree	48	-0.4237596	0.1046760	-0.7021784	-0.1453408
Full	Hateful non-audience outdegree	49	-0.4247607	0.1277057	-0.7644342	-0.0850871
Full	Hateful non-audience outdegree	50	-0.4872277	0.1122526	-0.7857989	-0.1886564
Full	Hateful non-audience outdegree	51	-0.4870978	0.1372084	-0.8520469	-0.1221486
Full	Hateful non-audience outdegree	52	-0.4844955	0.1296063	-0.8292245	-0.1397665
Full	Hateful non-audience outdegree	53	-0.4811047	0.1514023	-0.8838070	-0.0784024
Full	Hateful non-audience outdegree	54	-0.4958582	0.1535991	-0.9044035	-0.0873129
Full	Hateful non-audience outdegree	55	-0.4464968	0.1491555	-0.8432231	-0.0497706
Full	Hateful non-audience outdegree	56	-0.5967919	0.1366818	-0.9603404	-0.2332434
Full	Hateful non-audience outdegree	57	-0.5505257	0.2093625	-1.1073914	0.0063399

Full	Hateful non-audience outdegree	58	-0.5147238	0.1538038	-0.9238135	-0.1056341
Full	Hateful non-audience outdegree	59	-0.5190841	0.1838836	-1.0081806	-0.0299875

sample	outcome	event.time	estimate	std.error	conf.low	conf.high
Most	Hateful non-audience outdegree	-44	0.0444311	0.0087116	0.0216073	0.0672550
Most	Hateful non-audience outdegree	-43	-0.0404117	0.0136585	-0.0761962	-0.0046272
Most	Hateful non-audience outdegree	-42	0.0695978	0.0131015	0.0352726	0.1039230
Most	Hateful non-audience outdegree	-41	-0.0096461	0.0201759	-0.0625059	0.0432137
Most	Hateful non-audience outdegree	-40	0.0668319	0.0495698	-0.0630387	0.1967025
Most	Hateful non-audience outdegree	-39	-0.0581586	0.0500005	-0.1891574	0.0728402
Most	Hateful non-audience outdegree	-38	-0.0165354	0.0232843	-0.0775391	0.0444682
Most	Hateful non-audience outdegree	-37	-0.0811242	0.0113288	-0.1108052	-0.0514433
Most	Hateful non-audience outdegree	-36	0.0612354	0.0107919	0.0329612	0.0895096
Most	Hateful non-audience outdegree	-35	0.0953114	0.0267870	0.0251308	0.1654920
Most	Hateful non-audience outdegree	-34	0.1493356	0.0364683	0.0537903	0.2448808
Most	Hateful non-audience outdegree	-33	0.1421871	0.0523375	0.0050654	0.2793089
Most	Hateful non-audience outdegree	-32	-0.0534450	0.0542874	-0.1956754	0.0887855
Most	Hateful non-audience outdegree	-31	-0.0799866	0.0659664	-0.2528153	0.0928421
Most	Hateful non-audience outdegree	-30	0.1322872	0.0707658	-0.0531158	0.3176902
Most	Hateful non-audience outdegree	-29	0.0011551	0.0430318	-0.1115861	0.1138963
Most	Hateful non-audience outdegree	-28	-0.1359426	0.0331005	-0.2226644	-0.0492209
Most	Hateful non-audience outdegree	-27	-0.0094427	0.0616874	-0.1710606	0.1521753
Most	Hateful non-audience outdegree	-26	0.0132616	0.0503984	-0.1187798	0.1453030
Most	Hateful non-audience outdegree	-25	-0.0490356	0.0301711	-0.1280825	0.0300113
Most	Hateful non-audience outdegree	-24	-0.0613755	0.0316404	-0.1442718	0.0215208
Most	Hateful non-audience outdegree	-23	-0.0153434	0.0112337	-0.0447752	0.0140885
Most	Hateful non-audience outdegree	-22	-0.0182090	0.0186007	-0.0669420	0.0305240
Most	Hateful non-audience outdegree	-21	-0.0207507	0.0094040	-0.0453888	0.0038874
Most	Hateful non-audience outdegree	-20	0.0232394	0.0324428	-0.0617591	0.1082379
Most	Hateful non-audience outdegree	-19	-0.0021423	0.0404436	-0.1081026	0.1038180
Most	Hateful non-audience outdegree	-18	-0.0074119	0.0322990	-0.0920336	0.0772098
Most	Hateful non-audience outdegree	-17	0.0645960	0.0613151	-0.0960467	0.2252387
Most	Hateful non-audience outdegree	-16	0.0200074	0.0325563	-0.0652885	0.1053033
Most	Hateful non-audience outdegree	-15	-0.0384177	0.0215623	-0.0949099	0.0180746
Most	Hateful non-audience outdegree	-14	0.0383264	0.0278601	-0.0346658	0.1113186
Most	Hateful non-audience outdegree	-13	-0.0895222	0.0460252	-0.2101061	0.0310616
Most	Hateful non-audience outdegree	-12	0.0592695	0.0384791	-0.0415438	0.1600828
Most	Hateful non-audience outdegree	-11	-0.0245030	0.0168181	-0.0685655	0.0195596
Most	Hateful non-audience outdegree	-10	0.0421781	0.0226721	-0.0172217	0.1015779
Most	Hateful non-audience outdegree	-9	-0.0513440	0.0476692	-0.1762350	0.0735469
Most	Hateful non-audience outdegree	-8	-0.0000972	0.0484502	-0.1270344	0.1268400

Most	Hateful non-audience outdegree	-7	0.0167141	0.0165675	-0.0266918	0.0601201
Most	Hateful non-audience outdegree	-6	-0.0329427	0.0193421	-0.0836180	0.0177326
Most	Hateful non-audience outdegree	-5	0.0182682	0.0199891	-0.0341022	0.0706386
Most	Hateful non-audience outdegree	-4	-0.0182112	0.0271768	-0.0894130	0.0529907
Most	Hateful non-audience outdegree	-3	-0.0563671	0.0220241	-0.1140691	0.0013350
Most	Hateful non-audience outdegree	-2	0.0557280	0.0194398	0.0047966	0.1066593
Most	Hateful non-audience outdegree	-1	0.0611799	0.0476916	-0.0637697	0.1861295
Most	Hateful non-audience outdegree	0	-0.1416550	0.0881302	-0.3725520	0.0892420
Most	Hateful non-audience outdegree	1	-0.0106152	0.0512915	-0.1449963	0.1237660
Most	Hateful non-audience outdegree	2	-0.0234151	0.0336462	-0.1115666	0.0647364
Most	Hateful non-audience outdegree	3	0.0000102	0.0334791	-0.0877034	0.0877238
Most	Hateful non-audience outdegree	4	-0.0182849	0.0342331	-0.1079741	0.0714043
Most	Hateful non-audience outdegree	5	-0.0354442	0.0494388	-0.1649716	0.0940832
Most	Hateful non-audience outdegree	6	0.0152134	0.0519377	-0.1208610	0.1512877
Most	Hateful non-audience outdegree	7	0.0130310	0.0501278	-0.1183014	0.1443633
Most	Hateful non-audience outdegree	8	-0.0060021	0.0566236	-0.1543532	0.1423490
Most	Hateful non-audience outdegree	9	0.0198575	0.0562916	-0.1276237	0.1673388
Most	Hateful non-audience outdegree	10	-0.0337236	0.0637155	-0.2006551	0.1332078
Most	Hateful non-audience outdegree	11	-0.0403079	0.0630038	-0.2053747	0.1247590
Most	Hateful non-audience outdegree	12	-0.0373337	0.0545476	-0.1802458	0.1055783
Most	Hateful non-audience outdegree	13	-0.0323679	0.0465293	-0.1542723	0.0895365
Most	Hateful non-audience outdegree	14	-0.0011531	0.0717030	-0.1890116	0.1867054
Most	Hateful non-audience outdegree	15	0.0864936	0.1044350	-0.1871210	0.3601083
Most	Hateful non-audience outdegree	16	0.0631507	0.1063162	-0.2153927	0.3416940
Most	Hateful non-audience outdegree	17	0.0200520	0.1199415	-0.2941891	0.3342931
Most	Hateful non-audience outdegree	18	0.0534831	0.1315695	-0.2912225	0.3981887
Most	Hateful non-audience outdegree	19	0.0600715	0.1254121	-0.2685023	0.3886452
Most	Hateful non-audience outdegree	20	-0.0801678	0.0789685	-0.2870615	0.1267258
Most	Hateful non-audience outdegree	21	-0.0460106	0.0802615	-0.2562918	0.1642705
Most	Hateful non-audience outdegree	22	-0.0315964	0.0930548	-0.2753954	0.2122026
Most	Hateful non-audience outdegree	23	-0.0771098	0.0861519	-0.3028237	0.1486040
Most	Hateful non-audience outdegree	24	-0.0456636	0.0833655	-0.2640772	0.1727501
Most	Hateful non-audience outdegree	25	-0.0874750	0.0740145	-0.2813895	0.1064395
Most	Hateful non-audience outdegree	26	-0.0710889	0.0734623	-0.2635567	0.1213788
Most	Hateful non-audience outdegree	27	-0.1619010	0.0427158	-0.2738143	-0.0499876
Most	Hateful non-audience outdegree	28	-0.1825971	0.0558597	-0.3289468	-0.0362473
Most	Hateful non-audience outdegree	29	-0.3257447	0.1126191	-0.6208011	-0.0306882
Most	Hateful non-audience outdegree	30	-0.3605445	0.1205376	-0.6763471	-0.0447419
Most	Hateful non-audience outdegree	31	-0.3639269	0.0987360	-0.6226105	-0.1052432
Most	Hateful non-audience outdegree	32	-0.4408788	0.0654855	-0.6124476	-0.2693101
Most	Hateful non-audience outdegree	33	-0.4901230	0.0771479	-0.6922468	-0.2879991

Most	Hateful non-audience outdegree	34	-0.2862967	0.0643298	-0.4548376	-0.1177559
Most	Hateful non-audience outdegree	35	-0.2499873	0.0947391	-0.4981991	-0.0017754
Most	Hateful non-audience outdegree	36	-0.2553643	0.0848204	-0.4775897	-0.0331390
Most	Hateful non-audience outdegree	37	-0.2763487	0.0602308	-0.4341506	-0.1185468
Most	Hateful non-audience outdegree	38	-0.2737947	0.0748800	-0.4699767	-0.0776128
Most	Hateful non-audience outdegree	39	-0.2839181	0.0893858	-0.5181046	-0.0497317
Most	Hateful non-audience outdegree	40	-0.2596946	0.1153991	-0.5620346	0.0426454
Most	Hateful non-audience outdegree	41	-0.2560822	0.0723137	-0.4455404	-0.0666239
Most	Hateful non-audience outdegree	42	-0.1540320	0.1978863	-0.6724844	0.3644204
Most	Hateful non-audience outdegree	43	-0.5387359	0.1512920	-0.9351136	-0.1423582
Most	Hateful non-audience outdegree	44	-0.4644782	0.0627440	-0.6288646	-0.3000918
Most	Hateful non-audience outdegree	45	-0.4121667	0.2212801	-0.9919100	0.1675766
Most	Hateful non-audience outdegree	47	-0.5023863	0.0283660	-0.5767038	-0.4280687
Most	Hateful non-audience outdegree	48	-0.4169847	0.0244915	-0.4811513	-0.3528181
Most	Hateful non-audience outdegree	49	-0.4394691	0.0326470	-0.5250027	-0.3539355
Most	Hateful non-audience outdegree	50	-0.5260900	0.0263850	-0.5952174	-0.4569626
Most	Hateful non-audience outdegree	51	-0.5221810	0.1184530	-0.8325222	-0.2118398
Most	Hateful non-audience outdegree	52	-0.5147461	0.1259429	-0.8447103	-0.1847819
Most	Hateful non-audience outdegree	53	-0.5372249	0.1077627	-0.8195580	-0.2548919
Most	Hateful non-audience outdegree	54	-0.5700278	0.1305320	-0.9120152	-0.2280403
Most	Hateful non-audience outdegree	55	-0.5367898	0.1370378	-0.8958223	-0.1777573
Most	Hateful non-audience outdegree	56	-0.5775463	0.1396916	-0.9435316	-0.2115609
Most	Hateful non-audience outdegree	57	-0.5028651	0.1740322	-0.9588209	-0.0469093
Most	Hateful non-audience outdegree	58	-0.5710696	0.1565182	-0.9811396	-0.1609996
Most	Hateful non-audience outdegree	59	-0.5645681	0.2240916	-1.1516772	0.0225410

sample	outcome	event.time	estimate	std.error	conf.low	conf.high
Middle	Hateful non-audience outdegree	-44	-0.0346325	0.0178632	-0.0822352	0.0129703
Middle	Hateful non-audience outdegree	-43	0.0557311	0.0117847	0.0243265	0.0871357
Middle	Hateful non-audience outdegree	-42	-0.0113699	0.0154085	-0.0524313	0.0296915
Middle	Hateful non-audience outdegree	-41	0.0145374	0.0152635	-0.0261376	0.0552124
Middle	Hateful non-audience outdegree	-40	0.0431985	0.0419181	-0.0685074	0.1549043
Middle	Hateful non-audience outdegree	-39	-0.0417885	0.0469229	-0.1668313	0.0832543
Middle	Hateful non-audience outdegree	-38	-0.0562663	0.0179473	-0.1040933	-0.0084394
Middle	Hateful non-audience outdegree	-37	0.0119801	0.0118066	-0.0194828	0.0434430
Middle	Hateful non-audience outdegree	-36	0.0323831	0.0110663	0.0028930	0.0618732
Middle	Hateful non-audience outdegree	-35	0.0003453	0.0215424	-0.0570622	0.0577528
Middle	Hateful non-audience outdegree	-34	0.1498872	0.0225997	0.0896623	0.2101121
Middle	Hateful non-audience outdegree	-33	0.2686365	0.0504862	0.1340981	0.4031750
Middle	Hateful non-audience outdegree	-32	-0.0301725	0.0280676	-0.1049687	0.0446237
Middle	Hateful non-audience outdegree	-31	-0.0465039	0.0414533	-0.1569711	0.0639632

Middle	Hateful non-audience outdegree	-30	0.1490711	0.0495355	0.0170661	0.2810760
Middle	Hateful non-audience outdegree	-29	-0.0565530	0.0188213	-0.1067090	-0.0063970
Middle	Hateful non-audience outdegree	-28	-0.0727639	0.0320337	-0.1581291	0.0126012
Middle	Hateful non-audience outdegree	-27	0.0148815	0.0640740	-0.1558666	0.1856296
Middle	Hateful non-audience outdegree	-26	-0.0126619	0.0581093	-0.1675149	0.1421910
Middle	Hateful non-audience outdegree	-25	-0.0163453	0.0316833	-0.1007767	0.0680861
Middle	Hateful non-audience outdegree	-24	-0.0731884	0.0301326	-0.1534876	0.0071108
Middle	Hateful non-audience outdegree	-23	0.0051799	0.0199524	-0.0479904	0.0583503
Middle	Hateful non-audience outdegree	-22	-0.0281259	0.0192936	-0.0795405	0.0232887
Middle	Hateful non-audience outdegree	-21	0.0133554	0.0131752	-0.0217546	0.0484654
Middle	Hateful non-audience outdegree	-20	0.0169057	0.0134978	-0.0190639	0.0528753
Middle	Hateful non-audience outdegree	-19	0.0345951	0.0587682	-0.1220137	0.1912040
Middle	Hateful non-audience outdegree	-18	-0.0201198	0.0143639	-0.0583976	0.0181579
Middle	Hateful non-audience outdegree	-17	0.0726881	0.0251538	0.0056567	0.1397194
Middle	Hateful non-audience outdegree	-16	-0.0226722	0.0313954	-0.1063364	0.0609919
Middle	Hateful non-audience outdegree	-15	0.0240682	0.0218163	-0.0340693	0.0822056
Middle	Hateful non-audience outdegree	-14	0.0271964	0.0243221	-0.0376185	0.0920114
Middle	Hateful non-audience outdegree	-13	-0.0723791	0.0478757	-0.1999609	0.0552027
Middle	Hateful non-audience outdegree	-12	0.0560091	0.0367329	-0.0418789	0.1538970
Middle	Hateful non-audience outdegree	-11	0.0175966	0.0197985	-0.0351634	0.0703567
Middle	Hateful non-audience outdegree	-10	-0.0346789	0.0280465	-0.1094189	0.0400610
Middle	Hateful non-audience outdegree	-9	-0.0359793	0.0723094	-0.2286736	0.1567150
Middle	Hateful non-audience outdegree	-8	0.0535472	0.0755920	-0.1478946	0.2549890
Middle	Hateful non-audience outdegree	-7	-0.0326697	0.0186792	-0.0824471	0.0171078
Middle	Hateful non-audience outdegree	-6	-0.0872188	0.0339690	-0.1777413	0.0033038
Middle	Hateful non-audience outdegree	-5	0.0120130	0.0207947	-0.0434018	0.0674277
Middle	Hateful non-audience outdegree	-4	-0.0128467	0.0179390	-0.0606516	0.0349581
Middle	Hateful non-audience outdegree	-3	-0.1202765	0.0295845	-0.1991151	-0.0414379
Middle	Hateful non-audience outdegree	-2	0.0396843	0.0245170	-0.0256499	0.1050185
Middle	Hateful non-audience outdegree	-1	0.0759404	0.0417477	-0.0353113	0.1871920
Middle	Hateful non-audience outdegree	0	-0.1162170	0.0493927	-0.2478414	0.0154074
Middle	Hateful non-audience outdegree	1	0.0333378	0.0320725	-0.0521308	0.1188063
Middle	Hateful non-audience outdegree	2	0.0365373	0.0416177	-0.0743679	0.1474425
Middle	Hateful non-audience outdegree	3	0.0245061	0.0481115	-0.1037042	0.1527165
Middle	Hateful non-audience outdegree	4	0.0368749	0.0713706	-0.1533176	0.2270674
Middle	Hateful non-audience outdegree	5	0.0306859	0.0732691	-0.1645656	0.2259375
Middle	Hateful non-audience outdegree	6	0.0527507	0.0687348	-0.1304177	0.2359191
Middle	Hateful non-audience outdegree	7	0.0821385	0.0728328	-0.1119506	0.2762275
Middle	Hateful non-audience outdegree	8	0.0296433	0.0665389	-0.1476732	0.2069599
Middle	Hateful non-audience outdegree	9	0.0416835	0.0699314	-0.1446738	0.2280407
Middle	Hateful non-audience outdegree	10	0.0599714	0.0769219	-0.1450143	0.2649571

Middle	Hateful non-audience outdegree	11	0.0615278	0.0882018	-0.1735173	0.2965730
Middle	Hateful non-audience outdegree	12	0.0506894	0.0942413	-0.2004501	0.3018289
Middle	Hateful non-audience outdegree	13	0.0061377	0.0808308	-0.2092649	0.2215402
Middle	Hateful non-audience outdegree	14	0.0053911	0.0858871	-0.2234856	0.2342678
Middle	Hateful non-audience outdegree	15	0.1135051	0.1411031	-0.2625145	0.4895247
Middle	Hateful non-audience outdegree	16	0.1027172	0.1392611	-0.2683936	0.4738281
Middle	Hateful non-audience outdegree	17	0.1278406	0.1793343	-0.3500597	0.6057409
Middle	Hateful non-audience outdegree	18	0.1445679	0.1924635	-0.3683199	0.6574557
Middle	Hateful non-audience outdegree	19	0.1207334	0.1592573	-0.3036646	0.5451313
Middle	Hateful non-audience outdegree	20	-0.0243387	0.1021305	-0.2965020	0.2478245
Middle	Hateful non-audience outdegree	21	0.0018419	0.1123106	-0.2974499	0.3011336
Middle	Hateful non-audience outdegree	22	-0.0118946	0.1137657	-0.3150639	0.2912748
Middle	Hateful non-audience outdegree	23	-0.0489660	0.1105596	-0.3435914	0.2456595
Middle	Hateful non-audience outdegree	24	0.0005594	0.1243938	-0.3309322	0.3320511
Middle	Hateful non-audience outdegree	25	0.0014919	0.1341442	-0.3559831	0.3589670
Middle	Hateful non-audience outdegree	26	-0.0385584	0.0904154	-0.2795024	0.2023856
Middle	Hateful non-audience outdegree	27	-0.1228527	0.0373100	-0.2222785	-0.0234268
Middle	Hateful non-audience outdegree	28	-0.1494205	0.0534784	-0.2919327	-0.0069083
Middle	Hateful non-audience outdegree	29	-0.1897427	0.1104241	-0.4840072	0.1045219
Middle	Hateful non-audience outdegree	30	-0.2431881	0.1075815	-0.5298775	0.0435013
Middle	Hateful non-audience outdegree	31	-0.2793021	0.0761342	-0.4821889	-0.0764154
Middle	Hateful non-audience outdegree	32	-0.3019902	0.0638793	-0.4722194	-0.1317609
Middle	Hateful non-audience outdegree	33	-0.2964278	0.0467705	-0.4210644	-0.1717912
Middle	Hateful non-audience outdegree	34	-0.2021583	0.0486167	-0.3317149	-0.0726017
Middle	Hateful non-audience outdegree	35	-0.1585637	0.0722315	-0.3510504	0.0339230
Middle	Hateful non-audience outdegree	36	-0.1505361	0.0709968	-0.3397323	0.0386601
Middle	Hateful non-audience outdegree	37	-0.2030317	0.0476084	-0.3299012	-0.0761623
Middle	Hateful non-audience outdegree	38	-0.2026901	0.0627009	-0.3697789	-0.0356012
Middle	Hateful non-audience outdegree	39	-0.2058297	0.0531720	-0.3475255	-0.0641338
Middle	Hateful non-audience outdegree	40	-0.2159038	0.0608306	-0.3780085	-0.0537990
Middle	Hateful non-audience outdegree	41	-0.2054176	0.0629751	-0.3732372	-0.0375980
Middle	Hateful non-audience outdegree	42	-0.1218763	0.1151343	-0.4286928	0.1849402
Middle	Hateful non-audience outdegree	43	-0.4591996	0.0770999	-0.6646599	-0.2537394
Middle	Hateful non-audience outdegree	44	-0.4531408	0.1180857	-0.7678223	-0.1384592
Middle	Hateful non-audience outdegree	45	-0.4927007	0.1065028	-0.7765156	-0.2088859
Middle	Hateful non-audience outdegree	47	-0.3462727	0.0482467	-0.4748433	-0.2177022
Middle	Hateful non-audience outdegree	48	-0.2914883	0.0413600	-0.4017067	-0.1812700
Middle	Hateful non-audience outdegree	49	-0.3040336	0.0568843	-0.4556222	-0.1524450
Middle	Hateful non-audience outdegree	50	-0.3334814	0.0500010	-0.4667269	-0.2002358
Middle	Hateful non-audience outdegree	51	-0.3414430	0.0701139	-0.5282864	-0.1545996
Middle	Hateful non-audience outdegree	52	-0.3305604	0.0768528	-0.5353622	-0.1257587

Middle	Hateful non-audience outdegree	53	-0.3347334	0.0714811	-0.5252204	-0.1442465
Middle	Hateful non-audience outdegree	54	-0.3607013	0.0739917	-0.5578786	-0.1635239
Middle	Hateful non-audience outdegree	55	-0.3299589	0.0746236	-0.5288202	-0.1310977
Middle	Hateful non-audience outdegree	56	-0.4618894	0.3724332	-1.4543705	0.5305918
Middle	Hateful non-audience outdegree	57	-0.4022469	0.1562039	-0.8185080	0.0140142
Middle	Hateful non-audience outdegree	58	-0.3559488	0.1219155	-0.6808362	-0.0310614
Middle	Hateful non-audience outdegree	59	-0.4107590	0.1471461	-0.8028822	-0.0186358

sample	outcome	event.time	estimate	std.error	conf.low	conf.high
Least	Hateful non-audience outdegree	-44	-0.0029147	0.0231034	-0.0645796	0.0587502
Least	Hateful non-audience outdegree	-43	0.0019581	0.0322317	-0.0840711	0.0879873
Least	Hateful non-audience outdegree	-42	0.0242323	0.0315006	-0.0598455	0.1083102
Least	Hateful non-audience outdegree	-41	-0.0054250	0.0337094	-0.0953983	0.0845482
Least	Hateful non-audience outdegree	-40	0.2076275	0.1033817	-0.0683074	0.4835623
Least	Hateful non-audience outdegree	-39	-0.2141011	0.0907386	-0.4562903	0.0280881
Least	Hateful non-audience outdegree	-38	0.0262287	0.0372514	-0.0731985	0.1256560
Least	Hateful non-audience outdegree	-37	-0.0278252	0.0224342	-0.0877039	0.0320535
Least	Hateful non-audience outdegree	-36	-0.0498485	0.0607370	-0.2119607	0.1122637
Least	Hateful non-audience outdegree	-35	0.1513434	0.0264535	0.0807368	0.2219500
Least	Hateful non-audience outdegree	-34	0.0770391	0.0403008	-0.0305271	0.1846053
Least	Hateful non-audience outdegree	-33	0.1282132	0.1029352	-0.1465296	0.4029561
Least	Hateful non-audience outdegree	-32	0.0486310	0.0686165	-0.1345123	0.2317743
Least	Hateful non-audience outdegree	-31	-0.0802874	0.0488513	-0.2106758	0.0501010
Least	Hateful non-audience outdegree	-30	0.2076964	0.0995994	-0.0581431	0.4735359
Least	Hateful non-audience outdegree	-29	-0.0257546	0.0286077	-0.1021110	0.0506018
Least	Hateful non-audience outdegree	-28	-0.0225637	0.0223510	-0.0822205	0.0370931
Least	Hateful non-audience outdegree	-27	-0.0098702	0.1170958	-0.3224091	0.3026688
Least	Hateful non-audience outdegree	-26	0.1364081	0.0824037	-0.0835345	0.3563506
Least	Hateful non-audience outdegree	-25	-0.1070404	0.0568280	-0.2587192	0.0446383
Least	Hateful non-audience outdegree	-24	0.0073306	0.0789228	-0.2033212	0.2179824
Least	Hateful non-audience outdegree	-23	-0.0059765	0.0179438	-0.0538700	0.0419170
Least	Hateful non-audience outdegree	-22	-0.0180903	0.0503833	-0.1525677	0.1163870
Least	Hateful non-audience outdegree	-21	0.0828375	0.0297157	0.0035237	0.1621513
Least	Hateful non-audience outdegree	-20	-0.0027761	0.0489537	-0.1334377	0.1278856
Least	Hateful non-audience outdegree	-19	0.1458317	0.0380173	0.0443603	0.2473031
Least	Hateful non-audience outdegree	-18	-0.0696643	0.0431131	-0.1847368	0.0454082
Least	Hateful non-audience outdegree	-17	0.0337284	0.0433032	-0.0818515	0.1493083
Least	Hateful non-audience outdegree	-16	0.0067814	0.0400547	-0.1001280	0.1136908
Least	Hateful non-audience outdegree	-15	0.0646859	0.0427744	-0.0494828	0.1788546
Least	Hateful non-audience outdegree	-14	-0.0153528	0.0290832	-0.0929784	0.0622728
Least	Hateful non-audience outdegree	-13	-0.1529964	0.0654955	-0.3278096	0.0218168

Least	Hateful non-audience outdegree	-12	0.0736121	0.0722969	-0.1193546	0.2665789
Least	Hateful non-audience outdegree	-11	-0.0153779	0.0272058	-0.0879925	0.0572366
Least	Hateful non-audience outdegree	-10	-0.0519449	0.0343927	-0.1437420	0.0398522
Least	Hateful non-audience outdegree	-9	-0.0860086	0.0781504	-0.2945987	0.1225815
Least	Hateful non-audience outdegree	-8	0.0888649	0.0712365	-0.1012715	0.2790013
Least	Hateful non-audience outdegree	-7	-0.0679248	0.0369293	-0.1664922	0.0306427
Least	Hateful non-audience outdegree	-6	-0.0720629	0.0478897	-0.1998847	0.0557589
Least	Hateful non-audience outdegree	-5	-0.0060984	0.0320837	-0.0917325	0.0795358
Least	Hateful non-audience outdegree	-4	-0.0144386	0.0243021	-0.0793032	0.0504259
Least	Hateful non-audience outdegree	-3	-0.1405751	0.0461651	-0.2637937	-0.0173566
Least	Hateful non-audience outdegree	-2	0.0232218	0.0372140	-0.0761057	0.1225493
Least	Hateful non-audience outdegree	-1	0.0764354	0.0401306	-0.0306766	0.1835474
Least	Hateful non-audience outdegree	0	-0.2476496	0.0847148	-0.4737608	-0.0215384
Least	Hateful non-audience outdegree	1	-0.0063694	0.0422112	-0.1190348	0.1062961
Least	Hateful non-audience outdegree	2	-0.0024423	0.0398871	-0.1089045	0.1040199
Least	Hateful non-audience outdegree	3	0.0065489	0.0736048	-0.1899086	0.2030064
Least	Hateful non-audience outdegree	4	-0.0352492	0.0772589	-0.2414599	0.1709615
Least	Hateful non-audience outdegree	5	-0.0207431	0.1004739	-0.2889165	0.2474304
Least	Hateful non-audience outdegree	6	0.0339254	0.0812190	-0.1828552	0.2507059
Least	Hateful non-audience outdegree	7	0.0285622	0.0870144	-0.2036868	0.2608112
Least	Hateful non-audience outdegree	8	0.0442743	0.0916789	-0.2004246	0.2889732
Least	Hateful non-audience outdegree	9	0.0238990	0.1277482	-0.3170719	0.3648699
Least	Hateful non-audience outdegree	10	-0.0158160	0.1331160	-0.3711141	0.3394820
Least	Hateful non-audience outdegree	11	-0.0308886	0.1267148	-0.3691014	0.3073242
Least	Hateful non-audience outdegree	12	-0.0062639	0.1381613	-0.3750282	0.3625005
Least	Hateful non-audience outdegree	13	-0.0677182	0.0960182	-0.3239990	0.1885625
Least	Hateful non-audience outdegree	14	-0.0946189	0.1233819	-0.4239359	0.2346981
Least	Hateful non-audience outdegree	15	0.0383346	0.1897255	-0.4680592	0.5447284
Least	Hateful non-audience outdegree	16	0.0122707	0.2392133	-0.6262103	0.6507517
Least	Hateful non-audience outdegree	17	-0.0300482	0.2193807	-0.6155942	0.5554978
Least	Hateful non-audience outdegree	18	0.0159045	0.2400369	-0.6247748	0.6565838
Least	Hateful non-audience outdegree	19	-0.0140352	0.1792299	-0.4924153	0.4643449
Least	Hateful non-audience outdegree	20	-0.1489053	0.1240018	-0.4798770	0.1820663
Least	Hateful non-audience outdegree	21	-0.1058078	0.1400569	-0.4796319	0.2680163
Least	Hateful non-audience outdegree	22	-0.1328238	0.1377461	-0.5004801	0.2348326
Least	Hateful non-audience outdegree	23	-0.1517691	0.1382323	-0.5207231	0.2171849
Least	Hateful non-audience outdegree	24	-0.0839426	0.1567556	-0.5023370	0.3344518
Least	Hateful non-audience outdegree	25	-0.1317763	0.1613447	-0.5624193	0.2988668
Least	Hateful non-audience outdegree	26	-0.1346313	0.1565098	-0.5523696	0.2831070
Least	Hateful non-audience outdegree	27	-0.2844632	0.0880382	-0.5194447	-0.0494816
Least	Hateful non-audience outdegree	28	-0.3143072	0.0936053	-0.5641478	-0.0644667

Least	Hateful non-audience outdegree	29	-0.4484972	0.1833130	-0.9377754	0.0407809
Least	Hateful non-audience outdegree	30	-0.4757423	0.2131320	-1.0446102	0.0931256
Least	Hateful non-audience outdegree	31	-0.5488040	0.1662187	-0.9924561	-0.1051518
Least	Hateful non-audience outdegree	32	-0.5327188	0.2214435	-1.1237707	0.0583332
Least	Hateful non-audience outdegree	33	-0.5145576	0.3227580	-1.3760265	0.3469113
Least	Hateful non-audience outdegree	34	-0.4543215	0.1747795	-0.9208231	0.0121801
Least	Hateful non-audience outdegree	35	-0.4065484	0.2429505	-1.0550044	0.2419076
Least	Hateful non-audience outdegree	36	-0.3919405	0.1233723	-0.7212317	-0.0626493
Least	Hateful non-audience outdegree	37	-0.4864917	0.1402733	-0.8608932	-0.1120903
Least	Hateful non-audience outdegree	38	-0.4439644	0.1303658	-0.7919219	-0.0960069
Least	Hateful non-audience outdegree	39	-0.4668402	0.1624868	-0.9005316	-0.0331488
Least	Hateful non-audience outdegree	40	-0.4867697	0.1111198	-0.7833580	-0.1901814
Least	Hateful non-audience outdegree	41	-0.4581993	0.0914628	-0.7023214	-0.2140772
Least	Hateful non-audience outdegree	42	-0.4380644	0.3149870	-1.2787920	0.4026631
Least	Hateful non-audience outdegree	43	-0.8708919	1.4479657	-4.7356377	2.9938540
Least	Hateful non-audience outdegree	44	-0.8229087	0.6495535	-2.5566234	0.9108059
Least	Hateful non-audience outdegree	45	-0.9670791	0.6852011	-2.7959401	0.8617819
Least	Hateful non-audience outdegree	47	-0.6168713	0.0424499	-0.7301737	-0.5035689
Least	Hateful non-audience outdegree	48	-0.5714794	0.0355230	-0.6662933	-0.4766656
Least	Hateful non-audience outdegree	49	-0.5508072	0.0470851	-0.6764813	-0.4251331
Least	Hateful non-audience outdegree	50	-0.6392872	0.0471173	-0.7650474	-0.5135271
Least	Hateful non-audience outdegree	51	-0.6383981	0.2904137	-1.4135374	0.1367413
Least	Hateful non-audience outdegree	52	-0.6481434	0.1501065	-1.0487905	-0.2474963
Least	Hateful non-audience outdegree	53	-0.6216999	0.2216342	-1.2132608	-0.0301389
Least	Hateful non-audience outdegree	54	-0.6134177	0.3233128	-1.4763677	0.2495322
Least	Hateful non-audience outdegree	55	-0.5334071	0.2782489	-1.2760774	0.2092632
Least	Hateful non-audience outdegree	56	-0.8274929	0.5035870	-2.1716103	0.5166245
Least	Hateful non-audience outdegree	57	-0.8353434	0.5576970	-2.3238849	0.6531980
Least	Hateful non-audience outdegree	58	-0.6931418	0.6156324	-2.3363181	0.9500345
Least	Hateful non-audience outdegree	59	-0.6325110	0.5141476	-2.0048154	0.7397935

Tables of outcome variables aggregated at disruption and day level

Disruption	Day	Views on hateful content	Views on hateful content/total views	Views on non-organization content that is hateful	Hateful comments by audience members	Hateful comments/total comments	Hateful audience outdegree	Hateful non-audience outdegree	Audience indegree	Audience outdegree	Audience indegree/total indegree	Audience outdegree/total outdegree
Cluster 4	0	2.65	0.01	2.64	0.36	0.02	0.04	0.54	1.12	1.08	0.06	0.06
Cluster 4	1	2.63	0.01	2.62	0.35	0.02	0.04	0.55	1.12	1.12	0.06	0.07
Cluster 4	2	2.75	0.01	2.75	0.41	0.02	0.04	0.60	1.16	1.12	0.06	0.07
Cluster 4	3	2.73	0.01	2.72	0.38	0.02	0.04	0.57	1.11	1.04	0.05	0.07
Cluster 4	4	2.61	0.01	2.59	0.43	0.02	0.05	0.61	1.16	1.05	0.06	0.07
Cluster 4	5	2.62	0.01	2.61	0.42	0.02	0.05	0.57	1.12	1.10	0.06	0.07
Cluster 4	6	2.75	0.01	2.73	0.43	0.02	0.05	0.62	1.14	1.10	0.06	0.07
Cluster 4	7	2.59	0.01	2.56	0.35	0.02	0.04	0.53	1.16	1.04	0.06	0.07
Cluster 4	8	2.60	0.01	2.58	0.35	0.03	0.03	0.51	1.16	1.05	0.05	0.06
Cluster 4	9	2.71	0.01	2.70	0.36	0.02	0.05	0.56	1.15	1.10	0.06	0.07
Cluster 4	10	3.29	0.01	3.29	0.45	0.03	0.05	0.63	1.15	1.03	0.06	0.06
Cluster 4	11	4.01	0.01	4.00	0.54	0.03	0.06	0.77	1.10	1.05	0.06	0.07
Cluster 4	12	5.23	0.01	5.23	0.71	0.04	0.09	1.06	1.20	1.13	0.06	0.07
Cluster 4	13	6.03	0.02	6.03	0.77	0.04	0.09	1.14	1.23	1.14	0.06	0.07
Cluster 4	14	7.20	0.02	7.20	0.85	0.04	0.10	1.34	1.20	1.21	0.05	0.07
Cluster 4	15	9.42	0.02	9.42	0.95	0.05	0.12	1.57	1.23	1.23	0.06	0.07
Cluster 4	16	9.15	0.02	9.15	0.92	0.05	0.12	1.61	1.22	1.20	0.06	0.07
Cluster 4	17	8.59	0.02	8.59	0.91	0.05	0.10	1.49	1.35	1.22	0.05	0.07
Cluster 4	18	6.78	0.02	6.78	0.75	0.04	0.09	1.24	1.41	1.21	0.06	0.07
Cluster 4	19	5.81	0.02	5.81	0.67	0.04	0.09	1.11	1.20	1.13	0.06	0.06
Cluster 4	20	5.40	0.02	5.40	0.63	0.03	0.08	1.07	1.12	1.09	0.05	0.06
Cluster 4	21	4.96	0.01	4.95	0.53	0.04	0.07	0.89	1.11	1.09	0.06	0.06
Cluster 4	22	4.86	0.01	4.85	0.58	0.03	0.07	0.89	1.03	1.04	0.05	0.06
Cluster 4	23	4.84	0.01	4.84	0.54	0.03	0.07	0.91	1.02	1.07	0.05	0.07

Cluster 4	24	4.64	0.01	4.64	0.46	0.03	0.06	0.83	1.01	1.02	0.06	0.07
Cluster 4	25	4.53	0.01	4.53	0.52	0.03	0.07	0.86	1.07	0.99	0.05	0.06
Cluster 4	26	4.71	0.01	4.71	0.55	0.03	0.07	0.90	1.04	1.03	0.05	0.06
Cluster 4	27	4.61	0.01	4.61	0.54	0.03	0.07	0.87	1.11	1.02	0.06	0.07
Cluster 4	28	3.99	0.01	3.99	0.46	0.03	0.06	0.78	1.06	0.92	0.05	0.06
Cluster 4	29	4.08	0.01	4.08	0.50	0.03	0.05	0.80	0.92	0.95	0.05	0.06
Cluster 4	30	3.99	0.01	3.99	0.51	0.03	0.05	0.76	0.99	0.93	0.05	0.06
Cluster 4	31	3.96	0.01	3.96	0.52	0.03	0.04	0.80	1.01	0.91	0.05	0.06
Cluster 4	32	4.32	0.01	4.32	0.53	0.03	0.05	0.86	0.93	0.88	0.05	0.06
Cluster 4	33	4.17	0.01	4.17	0.54	0.03	0.05	0.85	0.87	0.85	0.05	0.06
Cluster 4	34	4.11	0.01	4.11	0.51	0.03	0.05	0.81	0.92	0.90	0.05	0.06
Cluster 4	35	3.85	0.01	3.85	0.46	0.03	0.04	0.74	0.89	0.88	0.05	0.06
Cluster 4	36	3.30	0.01	3.30	0.41	0.03	0.04	0.68	1.10	1.05	0.05	0.06
Cluster 4	37	3.81	0.01	3.81	0.48	0.03	0.04	0.74	0.88	0.84	0.05	0.06
Cluster 4	38	4.07	0.01	4.07	0.54	0.03	0.05	0.81	0.92	0.88	0.05	0.06
Cluster 4	39	3.82	0.01	3.82	0.50	0.03	0.05	0.83	0.95	0.89	0.05	0.06
Cluster 4	40	3.89	0.01	3.89	0.50	0.03	0.05	0.80	0.97	0.88	0.05	0.06
Cluster 4	41	3.70	0.01	3.70	0.46	0.03	0.04	0.71	0.85	0.84	0.05	0.06
Cluster 4	42	3.63	0.01	3.63	0.45	0.03	0.04	0.74	0.87	0.84	0.05	0.06
Cluster 4	43	3.60	0.01	3.60	0.47	0.03	0.04	0.72	0.85	0.83	0.05	0.05
Cluster 4	44	4.03	0.01	4.03	0.58	0.03	0.05	0.84	0.88	0.85	0.05	0.06
Cluster 4	45	3.76	0.01	3.76	0.52	0.03	0.05	0.81	0.90	0.85	0.05	0.06
Cluster 4	46	3.52	0.01	3.52	0.47	0.03	0.04	0.78	0.87	0.86	0.05	0.05
Cluster 4	47	3.55	0.01	3.55	0.48	0.03	0.05	0.71	0.85	0.82	0.05	0.05
Cluster 4	48	3.29	0.01	3.29	0.41	0.03	0.04	0.66	0.90	0.83	0.05	0.06
Cluster 4	49	3.29	0.01	3.29	0.41	0.03	0.05	0.69	1.00	0.93	0.05	0.06
Cluster 4	50	3.61	0.01	3.61	0.47	0.03	0.04	0.72	0.78	0.81	0.05	0.05
Cluster 4	51	3.85	0.01	3.85	0.50	0.03	0.04	0.78	0.90	0.80	0.05	0.06
Cluster 4	52	3.77	0.01	3.77	0.50	0.03	0.05	0.76	0.98	0.85	0.05	0.06

Cluster 4	53	3.51	0.01	3.51	0.49	0.03	0.04	0.72	0.88	0.84	0.05	0.06
Cluster 4	54	3.28	0.01	3.28	0.46	0.03	0.04	0.73	0.80	0.82	0.05	0.06
Cluster 4	55	3.20	0.01	3.20	0.48	0.03	0.03	0.72	0.86	0.82	0.05	0.06
Cluster 4	56	3.02	0.01	3.02	0.37	0.02	0.03	0.66	0.89	0.80	0.05	0.06
Cluster 4	57	3.09	0.01	3.09	0.39	0.03	0.04	0.65	0.81	0.80	0.05	0.06
Cluster 4	58	3.29	0.01	3.29	0.46	0.03	0.04	0.74	0.95	0.81	0.05	0.05
Cluster 4	59	3.35	0.01	3.35	0.49	0.03	0.03	0.71	0.83	0.80	0.05	0.06
Cluster 4	65	2.36	0.01	2.36	0.43	0.03	0.03	0.58	0.90	0.80	0.05	0.06
Cluster 4	66	3.08	0.01	3.08	0.47	0.03	0.04	0.70	0.83	0.83	0.04	0.06
Cluster 4	67	3.25	0.01	3.25	0.46	0.03	0.04	0.70	0.90	0.81	0.05	0.05
Cluster 4	68	2.32	0.01	2.32	0.46	0.03	0.03	0.61	0.80	0.81	0.05	0.05
Cluster 4	69	2.80	0.01	2.80	0.49	0.03	0.03	0.69	0.90	0.81	0.05	0.05
Cluster 4	70	2.93	0.01	2.93	0.47	0.03	0.04	0.68	0.84	0.75	0.04	0.06
Cluster 4	71	3.02	0.01	3.02	0.41	0.03	0.04	0.66	0.80	0.84	0.05	0.05
Cluster 4	72	3.15	0.01	3.15	0.43	0.03	0.04	0.64	0.89	0.78	0.05	0.05
Cluster 4	73	3.21	0.01	3.21	0.43	0.03	0.04	0.62	0.80	0.79	0.05	0.05
Cluster 1	0	3.45	0.01	3.42	0.41	0.02	0.09	0.72	1.74	1.68	0.06	0.08
Cluster 1	1	3.46	0.01	3.44	0.52	0.03	0.12	0.75	1.76	1.75	0.06	0.07
Cluster 1	2	3.41	0.01	3.38	0.58	0.03	0.10	0.73	1.72	1.78	0.06	0.07
Cluster 1	3	3.52	0.01	3.49	0.40	0.02	0.09	0.65	1.64	1.64	0.07	0.08
Cluster 1	4	3.25	0.01	3.24	0.48	0.02	0.10	0.70	1.58	1.58	0.06	0.07
Cluster 1	5	3.21	0.01	3.19	0.54	0.03	0.10	0.70	1.63	1.71	0.06	0.07
Cluster 1	6	3.50	0.01	3.39	0.55	0.03	0.11	0.68	1.51	1.56	0.06	0.07
Cluster 1	7	3.32	0.01	3.25	0.40	0.03	0.08	0.64	1.45	1.64	0.06	0.07
Cluster 1	8	3.28	0.01	3.27	0.37	0.02	0.08	0.61	1.78	1.75	0.07	0.08
Cluster 1	9	3.45	0.01	3.44	0.47	0.03	0.09	0.72	1.49	1.64	0.06	0.08
Cluster 1	10	4.00	0.01	3.99	0.54	0.03	0.12	0.81	1.54	1.60	0.06	0.07
Cluster 1	11	4.59	0.01	4.57	0.70	0.03	0.14	0.97	1.70	1.63	0.07	0.08
Cluster 1	12	5.92	0.02	5.92	1.05	0.05	0.17	1.28	1.79	1.86	0.06	0.07

Cluster 1	13	7.26	0.02	7.25	1.25	0.05	0.23	1.57	1.74	1.93	0.07	0.08
Cluster 1	14	8.45	0.02	8.45	1.10	0.05	0.22	1.81	1.73	1.84	0.06	0.07
Cluster 1	15	11.94	0.03	11.94	1.27	0.06	0.28	2.10	1.65	1.84	0.05	0.07
Cluster 1	16	10.20	0.03	10.20	1.37	0.06	0.25	1.93	1.72	1.80	0.06	0.07
Cluster 1	17	9.85	0.02	9.85	1.26	0.05	0.21	1.82	2.06	1.84	0.06	0.08
Cluster 1	18	8.17	0.02	8.17	1.13	0.05	0.18	1.58	1.55	1.62	0.06	0.08
Cluster 1	19	7.21	0.02	7.21	0.92	0.04	0.14	1.55	1.51	1.56	0.06	0.07
Cluster 1	20	6.53	0.02	6.53	0.95	0.04	0.15	1.44	1.46	1.54	0.06	0.07
Cluster 1	21	6.21	0.02	6.21	0.75	0.04	0.16	1.25	1.56	1.58	0.06	0.07
Cluster 1	22	5.94	0.02	5.94	0.85	0.04	0.12	1.26	1.38	1.51	0.05	0.07
Cluster 1	23	5.46	0.02	5.46	0.71	0.04	0.12	1.06	1.30	1.34	0.05	0.06
Cluster 1	24	5.36	0.02	5.36	0.78	0.04	0.13	1.09	1.43	1.44	0.06	0.08
Cluster 1	25	5.43	0.02	5.43	0.88	0.04	0.15	1.13	1.42	1.47	0.05	0.08
Cluster 1	26	5.77	0.02	5.77	0.86	0.05	0.14	1.16	1.39	1.56	0.05	0.07
Cluster 1	27	5.63	0.02	5.63	0.78	0.03	0.15	1.18	1.54	1.39	0.06	0.07
Cluster 1	28	5.27	0.01	5.27	0.61	0.03	0.11	0.93	1.42	1.42	0.05	0.07
Cluster 1	29	4.81	0.01	4.81	0.56	0.03	0.10	1.01	1.40	1.36	0.06	0.07
Cluster 1	30	4.67	0.01	4.67	0.58	0.03	0.09	0.93	1.25	1.26	0.06	0.07
Cluster 1	31	4.79	0.01	4.79	0.63	0.03	0.07	0.95	1.16	1.33	0.05	0.07
Cluster 1	32	5.31	0.01	5.31	0.79	0.04	0.12	1.27	1.30	1.29	0.06	0.07
Cluster 1	33	5.15	0.01	5.15	0.70	0.04	0.11	1.09	1.32	1.22	0.06	0.06
Cluster 1	34	5.24	0.01	5.24	0.67	0.04	0.11	1.04	1.46	1.32	0.05	0.06
Cluster 1	35	5.24	0.01	5.24	0.58	0.04	0.10	1.05	1.43	1.40	0.06	0.07
Cluster 1	36	4.26	0.01	4.26	0.50	0.02	0.10	0.93	1.40	1.42	0.06	0.06
Cluster 1	37	4.86	0.01	4.86	0.78	0.04	0.13	1.02	1.27	1.25	0.06	0.06
Cluster 1	38	5.49	0.01	5.49	0.70	0.04	0.14	1.10	1.57	1.45	0.06	0.07
Cluster 1	39	4.84	0.01	4.84	0.67	0.04	0.10	0.95	1.45	1.33	0.05	0.06
Cluster 1	40	4.80	0.01	4.80	0.82	0.04	0.11	1.00	1.34	1.36	0.05	0.06
Cluster 1	41	4.68	0.01	4.68	0.64	0.03	0.09	0.93	1.27	1.25	0.05	0.06

Cluster 1	42	4.51	0.01	4.51	0.59	0.03	0.10	0.89	1.19	1.23	0.05	0.07
Cluster 1	43	4.55	0.01	4.55	0.67	0.03	0.09	0.93	1.25	1.27	0.05	0.06
Cluster 1	44	4.69	0.01	4.69	0.66	0.03	0.09	1.00	1.20	1.28	0.05	0.06
Cluster 1	45	4.44	0.01	4.44	0.73	0.03	0.08	1.16	1.23	1.12	0.06	0.06
Cluster 1	46	4.22	0.01	4.22	0.59	0.04	0.08	0.96	1.23	1.18	0.06	0.06
Cluster 1	47	3.97	0.01	3.97	0.57	0.03	0.10	0.83	1.33	1.27	0.06	0.06
Cluster 1	48	3.94	0.01	3.94	0.57	0.03	0.07	0.90	1.32	1.17	0.05	0.06
Cluster 1	49	3.76	0.01	3.76	0.50	0.04	0.07	0.77	1.26	1.26	0.05	0.06
Cluster 1	50	4.50	0.01	4.50	0.69	0.04	0.11	0.96	1.33	1.33	0.06	0.07
Cluster 1	51	4.72	0.01	4.72	0.77	0.04	0.10	1.02	1.17	1.25	0.05	0.07
Cluster 1	52	4.72	0.01	4.72	0.67	0.03	0.11	1.00	1.48	1.32	0.06	0.06
Cluster 1	53	4.19	0.01	4.19	0.56	0.04	0.11	0.96	1.40	1.22	0.06	0.05
Cluster 1	54	3.92	0.01	3.92	0.60	0.03	0.10	0.98	1.41	1.19	0.05	0.05
Cluster 1	55	3.85	0.01	3.85	0.60	0.03	0.10	1.06	1.29	1.22	0.05	0.06
Cluster 1	56	3.63	0.01	3.63	0.53	0.03	0.08	0.87	1.32	1.24	0.05	0.06
Cluster 1	57	3.73	0.01	3.73	0.47	0.03	0.08	0.81	1.13	1.16	0.06	0.06
Cluster 1	58	3.85	0.01	3.85	0.69	0.03	0.08	0.93	1.21	1.17	0.05	0.06
Cluster 1	59	4.08	0.01	4.08	0.52	0.03	0.09	0.76	1.32	1.10	0.05	0.06
Cluster 1	65	3.02	0.01	3.02	0.59	0.03	0.07	0.75	1.18	1.09	0.05	0.06
Cluster 1	66	3.69	0.01	3.69	0.59	0.04	0.07	0.91	1.22	1.19	0.06	0.06
Cluster 1	67	3.90	0.01	3.90	0.57	0.03	0.06	0.83	1.15	1.09	0.05	0.07
Cluster 1	68	3.03	0.01	3.03	0.48	0.03	0.04	0.66	1.42	1.17	0.06	0.06
Cluster 1	69	3.71	0.01	3.71	0.49	0.03	0.06	0.75	1.24	1.14	0.06	0.06
Cluster 1	70	3.98	0.01	3.98	0.69	0.03	0.08	0.91	1.34	1.21	0.06	0.07
Cluster 1	71	4.04	0.01	4.04	0.56	0.03	0.09	0.87	1.29	1.28	0.05	0.06
Cluster 1	72	4.23	0.01	4.23	0.68	0.04	0.08	0.94	1.08	1.17	0.05	0.06
Cluster 1	73	4.26	0.01	4.26	0.59	0.03	0.07	0.89	1.12	1.12	0.05	0.05
Cluster 6	0	4.28	0.01	4.25	0.45	0.03	0.11	0.67	1.62	1.63	0.06	0.08
Cluster 6	1	4.65	0.01	4.61	0.45	0.03	0.12	0.69	1.61	1.66	0.06	0.08

Cluster 6	2	4.70	0.01	4.66	0.50	0.03	0.12	0.72	1.69	1.69	0.06	0.08
Cluster 6	3	4.41	0.01	4.39	0.48	0.03	0.12	0.71	1.74	1.71	0.07	0.08
Cluster 6	4	4.35	0.01	4.32	0.48	0.03	0.11	0.74	1.69	1.67	0.06	0.08
Cluster 6	5	4.16	0.01	4.14	0.51	0.03	0.11	0.69	1.64	1.66	0.07	0.08
Cluster 6	6	4.56	0.01	4.53	0.50	0.03	0.10	0.74	1.60	1.67	0.07	0.08
Cluster 6	7	4.38	0.01	4.36	0.46	0.03	0.10	0.69	1.60	1.65	0.06	0.08
Cluster 6	8	4.52	0.01	4.50	0.49	0.03	0.11	0.65	1.58	1.66	0.06	0.08
Cluster 6	9	4.62	0.01	4.57	0.47	0.03	0.12	0.70	1.69	1.74	0.06	0.08
Cluster 6	10	5.31	0.01	5.30	0.60	0.04	0.16	0.84	1.91	1.90	0.07	0.09
Cluster 6	11	6.65	0.02	6.64	0.80	0.04	0.19	1.05	1.82	2.05	0.07	0.09
Cluster 6	12	7.81	0.02	7.79	1.11	0.05	0.23	1.43	2.12	2.18	0.07	0.09
Cluster 6	13	8.60	0.02	8.60	1.19	0.06	0.22	1.51	2.17	2.25	0.07	0.09
Cluster 6	14	9.57	0.02	9.56	1.17	0.05	0.21	1.55	2.19	2.20	0.07	0.09
Cluster 6	15	12.49	0.03	12.49	1.42	0.06	0.28	2.02	2.47	2.45	0.07	0.09
Cluster 6	16	11.95	0.03	11.94	1.34	0.06	0.25	1.91	2.27	2.40	0.07	0.09
Cluster 6	17	11.32	0.02	11.32	1.28	0.06	0.25	1.80	2.32	2.39	0.07	0.09
Cluster 6	18	9.43	0.02	9.42	1.16	0.05	0.21	1.53	2.25	2.26	0.07	0.09
Cluster 6	19	8.25	0.02	8.25	1.04	0.05	0.21	1.42	2.31	2.22	0.07	0.09
Cluster 6	20	8.02	0.02	8.02	1.01	0.05	0.20	1.39	2.14	2.16	0.07	0.09
Cluster 6	21	7.25	0.02	7.25	0.85	0.04	0.16	1.23	2.06	2.03	0.07	0.09
Cluster 6	22	7.14	0.02	7.13	0.85	0.04	0.16	1.21	2.04	2.00	0.07	0.09
Cluster 6	23	7.18	0.02	7.18	0.87	0.04	0.17	1.16	1.99	2.01	0.07	0.09
Cluster 6	24	7.07	0.02	7.07	0.87	0.04	0.16	1.17	2.11	1.97	0.07	0.09
Cluster 6	25	6.89	0.02	6.87	0.81	0.04	0.16	1.14	2.00	1.96	0.07	0.09
Cluster 6	26	7.12	0.02	7.12	0.86	0.04	0.16	1.18	2.00	1.97	0.07	0.08
Cluster 6	27	7.06	0.02	7.05	0.81	0.04	0.15	1.11	2.05	1.87	0.07	0.09
Cluster 6	28	6.22	0.02	6.22	0.74	0.04	0.14	1.02	1.85	1.74	0.07	0.08
Cluster 6	29	6.26	0.01	6.26	0.75	0.04	0.13	1.00	1.80	1.76	0.07	0.08
Cluster 6	30	6.28	0.01	6.28	0.76	0.04	0.13	1.01	1.87	1.84	0.07	0.09

Cluster 6	31	6.38	0.02	6.38	0.78	0.04	0.17	1.06	1.96	1.98	0.07	0.09
Cluster 6	32	6.70	0.02	6.70	0.85	0.04	0.16	1.14	1.99	2.01	0.07	0.09
Cluster 6	33	6.50	0.02	6.50	0.80	0.04	0.14	1.11	1.94	2.03	0.07	0.09
Cluster 6	34	6.36	0.02	6.36	0.80	0.04	0.15	1.05	1.85	1.96	0.06	0.09
Cluster 6	35	6.10	0.01	6.10	0.77	0.04	0.14	1.01	1.82	1.87	0.07	0.09
Cluster 6	36	5.74	0.01	5.74	0.67	0.04	0.13	0.90	1.96	1.94	0.07	0.08
Cluster 6	37	6.39	0.01	6.39	0.77	0.04	0.16	1.03	2.16	2.02	0.07	0.09
Cluster 6	38	6.68	0.02	6.68	0.80	0.04	0.16	1.10	2.06	1.97	0.07	0.09
Cluster 6	39	6.32	0.02	6.32	0.77	0.04	0.15	1.04	2.22	2.07	0.07	0.09
Cluster 6	40	6.41	0.02	6.41	0.76	0.04	0.16	1.05	2.13	2.03	0.07	0.09
Cluster 6	41	5.98	0.01	5.98	0.73	0.04	0.15	1.03	1.96	2.00	0.07	0.09
Cluster 6	42	5.74	0.01	5.74	0.61	0.04	0.13	0.92	1.91	1.88	0.07	0.08
Cluster 6	43	5.92	0.01	5.92	0.68	0.04	0.15	0.96	1.91	1.95	0.07	0.08
Cluster 6	44	6.39	0.02	6.39	0.79	0.04	0.17	1.15	2.18	2.15	0.07	0.09
Cluster 6	45	6.09	0.01	6.09	0.80	0.04	0.14	1.11	1.89	1.93	0.06	0.08
Cluster 6	46	5.74	0.01	5.74	0.72	0.04	0.13	1.00	1.77	1.77	0.06	0.07
Cluster 6	47	5.85	0.01	5.85	0.67	0.04	0.13	1.02	1.64	1.74	0.06	0.08
Cluster 6	48	5.44	0.01	5.44	0.63	0.04	0.11	0.92	1.67	1.65	0.06	0.07
Cluster 6	49	5.47	0.01	5.47	0.60	0.04	0.12	0.91	1.55	1.64	0.06	0.07
Cluster 6	50	5.71	0.01	5.71	0.64	0.04	0.11	0.92	1.57	1.60	0.06	0.07
Cluster 6	51	5.91	0.01	5.91	0.70	0.04	0.13	1.01	1.58	1.61	0.06	0.07
Cluster 6	52	6.02	0.01	6.02	0.73	0.04	0.13	1.01	1.70	1.63	0.06	0.07
Cluster 6	53	5.82	0.01	5.82	0.71	0.04	0.13	0.97	1.72	1.67	0.06	0.07
Cluster 6	54	5.46	0.01	5.45	0.67	0.04	0.12	0.95	1.72	1.63	0.06	0.07
Cluster 6	55	5.18	0.01	5.18	0.66	0.04	0.11	0.93	1.58	1.53	0.06	0.07
Cluster 6	56	5.03	0.01	5.03	0.62	0.04	0.10	0.85	1.61	1.49	0.05	0.07
Cluster 6	57	5.17	0.01	5.17	0.56	0.04	0.10	0.86	1.61	1.52	0.06	0.07
Cluster 6	58	5.23	0.01	5.22	0.68	0.04	0.11	0.91	1.57	1.51	0.06	0.07
Cluster 6	59	5.19	0.01	5.19	0.65	0.04	0.11	0.88	1.60	1.51	0.06	0.07

Cluster 6	65	3.86	0.01	3.86	0.65	0.04	0.09	0.75	1.45	1.43	0.06	0.07
Cluster 6	66	5.04	0.01	5.04	0.63	0.04	0.10	0.86	1.45	1.46	0.06	0.07
Cluster 6	67	5.31	0.01	5.31	0.65	0.04	0.11	0.88	1.46	1.49	0.06	0.07
Cluster 6	68	3.90	0.01	3.90	0.62	0.04	0.09	0.73	1.38	1.42	0.06	0.07
Cluster 6	69	4.77	0.01	4.77	0.60	0.04	0.11	0.85	1.44	1.45	0.06	0.07
Cluster 6	70	4.93	0.01	4.93	0.63	0.04	0.11	0.85	1.38	1.43	0.06	0.07
Cluster 6	71	4.96	0.01	4.96	0.59	0.04	0.12	0.90	1.41	1.47	0.06	0.07
Cluster 6	72	5.46	0.01	5.46	0.65	0.04	0.13	0.96	1.41	1.52	0.06	0.07
Cluster 6	73	5.23	0.01	5.23	0.61	0.04	0.12	0.91	1.55	1.48	0.06	0.07
Cluster 3	0	2.11	0.01	2.10	0.22	0.02	0.02	0.32	0.73	0.75	0.04	0.05
Cluster 3	1	2.19	0.01	2.18	0.20	0.02	0.02	0.34	0.77	0.78	0.05	0.05
Cluster 3	2	2.16	0.01	2.16	0.21	0.02	0.02	0.37	0.78	0.75	0.04	0.05
Cluster 3	3	2.17	0.01	2.16	0.21	0.02	0.02	0.35	0.76	0.74	0.04	0.05
Cluster 3	4	2.13	0.01	2.12	0.21	0.02	0.02	0.35	0.74	0.71	0.05	0.05
Cluster 3	5	2.05	0.01	2.04	0.20	0.02	0.02	0.00	0.66	0.65	0.05	0.99
Cluster 3	6	2.19	0.01	2.17	0.22	0.02	0.02	0.35	0.70	0.71	0.04	0.05
Cluster 3	7	2.12	0.01	2.11	0.20	0.02	0.02	0.34	0.78	0.77	0.05	0.06
Cluster 3	8	2.08	0.01	2.07	0.20	0.02	0.02	0.33	0.81	0.79	0.05	0.05
Cluster 3	9	2.22	0.01	2.20	0.20	0.02	0.02	0.35	0.71	0.74	0.05	0.05
Cluster 3	10	2.74	0.01	2.74	0.28	0.02	0.02	0.42	0.73	0.72	0.05	0.05
Cluster 3	11	3.16	0.01	3.16	0.27	0.02	0.03	0.48	0.71	0.74	0.05	0.05
Cluster 3	12	3.88	0.01	3.88	0.38	0.02	0.04	0.60	0.72	0.76	0.05	0.05
Cluster 3	13	4.21	0.01	4.21	0.44	0.03	0.04	0.68	0.83	0.78	0.05	0.05
Cluster 3	14	4.63	0.01	4.63	0.43	0.03	0.04	0.72	0.79	0.79	0.04	0.05
Cluster 3	15	5.73	0.01	5.73	0.45	0.03	0.05	0.84	0.83	0.82	0.05	0.05
Cluster 3	16	5.68	0.01	5.68	0.42	0.03	0.04	0.81	0.79	0.77	0.05	0.05
Cluster 3	17	5.59	0.01	5.59	0.45	0.03	0.04	0.81	0.78	0.78	0.05	0.05
Cluster 3	18	4.54	0.01	4.54	0.37	0.03	0.03	0.00	0.70	0.69	0.05	0.98
Cluster 3	19	3.90	0.01	3.90	0.32	0.03	0.03	0.58	0.72	0.72	0.04	0.05

Cluster 3	20	3.66	0.01	3.66	0.34	0.03	0.04	0.57	0.72	0.72	0.05	0.05
Cluster 3	21	3.37	0.01	3.37	0.30	0.02	0.03	0.52	0.69	0.70	0.04	0.05
Cluster 3	22	3.30	0.01	3.30	0.28	0.02	0.03	0.52	0.72	0.70	0.04	0.05
Cluster 3	23	3.19	0.01	3.19	0.29	0.02	0.03	0.50	0.68	0.67	0.04	0.05
Cluster 3	24	3.19	0.01	3.19	0.29	0.02	0.02	0.49	0.64	0.64	0.04	0.05
Cluster 3	25	3.15	0.01	3.15	0.30	0.02	0.03	0.48	0.66	0.66	0.04	0.05
Cluster 3	26	3.20	0.01	3.20	0.28	0.03	0.03	0.49	0.65	0.66	0.04	0.05
Cluster 3	27	3.13	0.01	3.13	0.29	0.02	0.03	0.49	0.68	0.66	0.04	0.05
Cluster 3	28	2.87	0.01	2.87	0.25	0.02	0.03	0.44	0.62	0.63	0.04	0.05
Cluster 3	29	2.89	0.01	2.89	0.24	0.02	0.02	0.45	0.63	0.66	0.04	0.05
Cluster 3	30	2.91	0.01	2.91	0.28	0.02	0.03	0.44	0.66	0.67	0.04	0.05
Cluster 3	31	2.81	0.01	2.81	0.25	0.02	0.03	0.42	0.65	0.66	0.04	0.05
Cluster 3	32	2.96	0.01	2.96	0.27	0.02	0.02	0.46	0.63	0.65	0.04	0.05
Cluster 3	33	2.91	0.01	2.91	0.29	0.02	0.03	0.48	0.62	0.64	0.04	0.05
Cluster 3	34	2.86	0.01	2.86	0.27	0.02	0.03	0.44	0.61	0.64	0.04	0.05
Cluster 3	35	2.77	0.01	2.77	0.23	0.02	0.02	0.42	0.65	0.64	0.04	0.05
Cluster 3	36	2.51	0.01	2.51	0.22	0.02	0.02	0.39	0.81	0.76	0.04	0.05
Cluster 3	37	2.77	0.01	2.77	0.23	0.02	0.02	0.43	0.66	0.65	0.04	0.05
Cluster 3	38	2.82	0.01	2.82	0.26	0.02	0.03	0.46	0.69	0.67	0.04	0.04
Cluster 3	39	2.65	0.01	2.65	0.25	0.02	0.02	0.43	0.67	0.67	0.04	0.05
Cluster 3	40	2.73	0.01	2.73	0.25	0.02	0.02	0.42	0.65	0.66	0.04	0.05
Cluster 3	41	2.62	0.01	2.62	0.24	0.02	0.02	0.43	0.64	0.64	0.04	0.05
Cluster 3	42	2.52	0.01	2.52	0.21	0.02	0.02	0.41	0.62	0.63	0.04	0.05
Cluster 3	43	2.62	0.01	2.62	0.24	0.02	0.02	0.40	0.63	0.65	0.04	0.05
Cluster 3	44	2.66	0.01	2.66	0.28	0.02	0.02	0.46	0.63	0.63	0.04	0.05
Cluster 3	45	2.61	0.01	2.61	0.26	0.02	0.03	0.46	0.61	0.64	0.04	0.04
Cluster 3	46	2.40	0.01	2.40	0.26	0.02	0.02	0.40	0.64	0.64	0.04	0.05
Cluster 3	47	2.46	0.01	2.46	0.26	0.02	0.02	0.43	0.62	0.62	0.04	0.05
Cluster 3	48	2.36	0.01	2.36	0.23	0.02	0.02	0.38	0.60	0.60	0.04	0.05

Cluster 3	49	2.35	0.01	2.35	0.20	0.02	0.03	0.38	0.66	0.65	0.04	0.05
Cluster 3	50	2.49	0.01	2.49	0.22	0.02	0.02	0.39	0.64	0.62	0.04	0.05
Cluster 3	51	2.52	0.01	2.52	0.24	0.02	0.02	0.41	0.60	0.62	0.04	0.04
Cluster 3	52	2.51	0.01	2.51	0.25	0.02	0.02	0.42	0.59	0.60	0.04	0.05
Cluster 3	53	2.48	0.01	2.48	0.24	0.02	0.02	0.44	0.61	0.61	0.04	0.05
Cluster 3	54	2.35	0.01	2.35	0.24	0.02	0.02	0.40	0.60	0.60	0.04	0.05
Cluster 3	55	2.28	0.01	2.28	0.24	0.02	0.02	0.39	0.63	0.59	0.04	0.04
Cluster 3	56	2.24	0.01	2.24	0.22	0.02	0.02	0.37	0.61	0.62	0.04	0.05
Cluster 3	57	2.26	0.01	2.26	0.21	0.02	0.02	0.37	0.64	0.66	0.04	0.05
Cluster 3	58	2.26	0.01	2.26	0.21	0.02	0.02	0.36	0.60	0.59	0.04	0.04
Cluster 3	59	2.26	0.01	2.26	0.22	0.02	0.02	0.36	0.60	0.62	0.04	0.05
Cluster 3	65	1.61	0.00	1.61	0.23	0.02	0.02	0.31	0.56	0.56	0.04	0.04
Cluster 3	66	2.13	0.01	2.13	0.21	0.02	0.02	0.37	0.59	0.58	0.04	0.05
Cluster 3	67	2.23	0.01	2.23	0.21	0.02	0.02	0.37	0.56	0.57	0.04	0.05
Cluster 3	68	1.66	0.00	1.66	0.21	0.02	0.01	0.30	0.58	0.56	0.04	0.04
Cluster 3	69	2.07	0.01	2.07	0.21	0.02	0.02	0.35	0.58	0.57	0.04	0.05
Cluster 3	70	2.15	0.01	2.15	0.20	0.02	0.01	0.34	0.56	0.56	0.04	0.05
Cluster 3	71	2.19	0.01	2.19	0.18	0.02	0.02	0.34	0.55	0.59	0.04	0.05
Cluster 3	72	2.15	0.01	2.15	0.21	0.02	0.02	0.35	0.56	0.59	0.04	0.05
Cluster 3	73	2.22	0.01	2.22	0.22	0.02	0.02	0.38	0.57	0.57	0.04	0.04
Cluster 5	0	3.47	0.01	3.46	0.47	0.03	0.09	0.70	1.17	1.23	0.05	0.05
Cluster 5	1	3.60	0.01	3.59	0.43	0.02	0.10	0.73	1.09	1.34	0.05	0.06
Cluster 5	2	3.71	0.01	3.70	0.45	0.03	0.09	0.76	1.07	1.30	0.04	0.05
Cluster 5	3	3.61	0.01	3.59	0.40	0.02	0.08	0.69	1.30	1.26	0.05	0.05
Cluster 5	4	3.61	0.01	3.59	0.45	0.02	0.10	0.79	1.26	1.38	0.05	0.06
Cluster 5	5	3.43	0.01	3.42	0.46	0.02	0.08	0.75	1.09	1.33	0.04	0.06
Cluster 5	6	3.72	0.01	3.71	0.48	0.03	0.09	0.79	1.10	1.37	0.04	0.06
Cluster 5	7	3.49	0.01	3.47	0.40	0.03	0.08	0.72	1.16	1.30	0.05	0.06
Cluster 5	8	3.41	0.01	3.39	0.41	0.02	0.08	0.65	1.09	1.25	0.05	0.06

Cluster 5	9	3.55	0.01	3.54	0.42	0.03	0.08	0.75	1.14	1.34	0.04	0.06
Cluster 5	10	4.32	0.01	4.32	0.51	0.03	0.09	0.83	1.10	1.34	0.05	0.06
Cluster 5	11	5.04	0.01	5.04	0.66	0.04	0.11	1.04	1.01	1.23	0.04	0.05
Cluster 5	12	6.62	0.02	6.61	0.97	0.05	0.16	1.39	1.22	1.40	0.05	0.06
Cluster 5	13	7.30	0.02	7.30	0.98	0.04	0.15	1.47	1.12	1.34	0.04	0.06
Cluster 5	14	8.89	0.02	8.89	1.00	0.05	0.20	1.75	1.17	1.43	0.05	0.06
Cluster 5	15	11.75	0.02	11.75	1.13	0.06	0.25	2.10	1.43	1.62	0.04	0.06
Cluster 5	16	10.40	0.02	10.40	1.14	0.05	0.18	1.94	1.32	1.52	0.04	0.05
Cluster 5	17	10.31	0.02	10.31	1.03	0.05	0.19	2.01	1.36	1.56	0.04	0.05
Cluster 5	18	8.31	0.02	8.31	0.91	0.05	0.16	1.60	1.20	1.45	0.05	0.06
Cluster 5	19	7.42	0.02	7.42	0.84	0.04	0.14	1.42	1.21	1.44	0.04	0.06
Cluster 5	20	7.11	0.02	7.11	0.80	0.04	0.14	1.47	1.15	1.30	0.05	0.06
Cluster 5	21	6.53	0.02	6.53	0.71	0.04	0.12	1.31	1.03	1.26	0.04	0.05
Cluster 5	22	6.33	0.02	6.33	0.67	0.04	0.11	1.22	1.00	1.22	0.04	0.06
Cluster 5	23	6.25	0.02	6.25	0.69	0.04	0.11	1.26	1.06	1.24	0.04	0.05
Cluster 5	24	6.08	0.02	6.08	0.64	0.04	0.12	1.16	0.97	1.20	0.04	0.05
Cluster 5	25	5.56	0.01	5.56	0.63	0.04	0.11	1.11	1.05	1.18	0.04	0.05
Cluster 5	26	6.11	0.02	6.11	0.58	0.04	0.11	1.13	1.11	1.24	0.04	0.06
Cluster 5	27	5.86	0.02	5.86	0.59	0.03	0.12	1.12	1.02	1.19	0.04	0.05
Cluster 5	28	5.17	0.01	5.17	0.52	0.04	0.10	0.99	1.00	1.14	0.04	0.05
Cluster 5	29	5.16	0.01	5.16	0.56	0.04	0.10	1.04	0.96	1.16	0.04	0.05
Cluster 5	30	4.92	0.01	4.92	0.53	0.04	0.09	0.93	0.98	1.07	0.04	0.05
Cluster 5	31	4.93	0.01	4.93	0.60	0.04	0.09	1.02	0.99	1.10	0.04	0.05
Cluster 5	32	5.26	0.01	5.26	0.61	0.04	0.09	1.07	1.00	1.06	0.04	0.05
Cluster 5	33	5.32	0.01	5.32	0.55	0.04	0.07	1.03	0.90	1.03	0.04	0.05
Cluster 5	34	5.17	0.01	5.17	0.60	0.04	0.09	1.05	0.86	1.02	0.04	0.05
Cluster 5	35	4.83	0.01	4.83	0.52	0.04	0.08	0.95	0.90	1.05	0.04	0.04
Cluster 5	36	4.51	0.01	4.51	0.47	0.03	0.07	0.89	1.00	1.10	0.04	0.05
Cluster 5	37	5.28	0.01	5.28	0.57	0.04	0.10	1.03	0.97	1.06	0.04	0.05

Cluster 5	38	5.43	0.01	5.43	0.62	0.04	0.10	1.13	0.97	1.10	0.04	0.05
Cluster 5	39	4.99	0.01	4.99	0.53	0.04	0.10	1.03	1.02	1.12	0.04	0.05
Cluster 5	40	4.96	0.01	4.96	0.55	0.04	0.08	1.04	0.99	1.04	0.04	0.04
Cluster 5	41	4.55	0.01	4.55	0.54	0.03	0.07	0.96	0.92	1.00	0.04	0.05
Cluster 5	42	4.28	0.01	4.28	0.53	0.04	0.08	0.92	0.83	0.98	0.04	0.04
Cluster 5	43	4.58	0.01	4.58	0.51	0.04	0.08	0.97	0.89	1.03	0.04	0.04
Cluster 5	44	4.88	0.01	4.88	0.61	0.04	0.09	1.15	0.91	1.12	0.04	0.05
Cluster 5	45	4.71	0.01	4.71	0.52	0.03	0.09	1.05	0.92	1.04	0.04	0.05
Cluster 5	46	4.36	0.01	4.36	0.49	0.03	0.07	0.97	0.92	1.00	0.04	0.04
Cluster 5	47	4.43	0.01	4.43	0.50	0.03	0.08	0.96	0.87	0.97	0.04	0.05
Cluster 5	48	4.02	0.01	4.02	0.50	0.03	0.07	0.93	0.86	1.02	0.04	0.05
Cluster 5	49	3.94	0.01	3.94	0.53	0.03	0.07	0.95	0.96	1.06	0.04	0.05
Cluster 5	50	4.36	0.01	4.36	0.49	0.04	0.08	0.93	0.89	0.99	0.03	0.04
Cluster 5	51	4.84	0.01	4.84	0.57	0.04	0.07	0.95	0.87	0.98	0.04	0.05
Cluster 5	52	4.80	0.01	4.80	0.49	0.03	0.07	0.93	0.88	0.98	0.04	0.05
Cluster 5	53	4.49	0.01	4.49	0.54	0.03	0.07	0.95	0.88	0.97	0.04	0.05
Cluster 5	54	4.06	0.01	4.06	0.56	0.04	0.06	0.94	0.80	0.92	0.04	0.05
Cluster 5	55	3.93	0.01	3.93	0.50	0.03	0.06	0.93	0.83	0.93	0.04	0.04
Cluster 5	56	3.78	0.01	3.78	0.45	0.03	0.06	0.88	0.83	0.91	0.03	0.04
Cluster 5	57	3.79	0.01	3.79	0.46	0.03	0.07	0.86	0.88	1.00	0.04	0.05
Cluster 5	58	4.13	0.01	4.13	0.49	0.03	0.06	0.89	0.89	0.96	0.04	0.05
Cluster 5	59	4.05	0.01	4.05	0.44	0.03	0.07	0.90	0.83	0.95	0.04	0.05
Cluster 5	65	2.84	0.01	2.84	0.51	0.03	0.05	0.73	0.78	0.94	0.03	0.04
Cluster 5	66	3.66	0.01	3.66	0.51	0.03	0.06	0.84	0.79	0.92	0.04	0.04
Cluster 5	67	3.94	0.01	3.94	0.54	0.03	0.06	0.92	0.81	0.95	0.04	0.05
Cluster 5	68	2.88	0.01	2.88	0.49	0.03	0.05	0.70	0.77	0.91	0.04	0.05
Cluster 5	69	3.43	0.01	3.43	0.47	0.04	0.06	0.76	0.79	0.94	0.04	0.05
Cluster 5	70	3.53	0.01	3.53	0.40	0.03	0.06	0.74	0.73	0.86	0.03	0.04
Cluster 5	71	3.86	0.01	3.86	0.45	0.03	0.06	0.76	0.77	0.94	0.04	0.04

Cluster 5	72	3.81	0.01	3.81	0.42	0.03	0.06	0.81	0.77	0.91	0.04	0.05
Cluster 5	73	3.79	0.01	3.79	0.48	0.03	0.07	0.83	0.79	0.97	0.03	0.05
Cluster 2	0	2.91	0.01	2.87	0.39	0.02	0.07	0.53	1.18	1.20	0.06	0.07
Cluster 2	1	3.05	0.01	3.00	0.45	0.03	0.05	0.59	1.33	1.16	0.06	0.07
Cluster 2	2	3.16	0.01	3.14	0.43	0.03	0.07	0.54	1.34	1.16	0.06	0.07
Cluster 2	3	3.12	0.01	3.08	0.40	0.03	0.06	0.53	1.32	1.17	0.06	0.07
Cluster 2	4	3.10	0.01	3.08	0.35	0.03	0.06	0.53	1.25	1.17	0.05	0.07
Cluster 2	5	3.00	0.01	3.00	0.34	0.02	0.06	0.48	1.35	1.24	0.06	0.07
Cluster 2	6	3.32	0.01	3.28	0.38	0.03	0.07	0.55	1.39	1.20	0.06	0.06
Cluster 2	7	2.94	0.01	2.90	0.32	0.02	0.06	0.47	1.24	1.09	0.06	0.07
Cluster 2	8	2.98	0.01	2.97	0.35	0.02	0.06	0.51	1.47	1.16	0.06	0.07
Cluster 2	9	3.22	0.01	3.18	0.33	0.02	0.07	0.55	1.41	1.23	0.06	0.07
Cluster 2	10	4.25	0.01	4.25	0.50	0.03	0.08	0.66	1.45	1.29	0.06	0.07
Cluster 2	11	4.90	0.01	4.90	0.57	0.04	0.08	0.73	1.39	1.16	0.06	0.07
Cluster 2	12	6.29	0.02	6.28	0.71	0.04	0.13	0.98	1.43	1.35	0.06	0.07
Cluster 2	13	7.04	0.02	7.04	0.85	0.04	0.12	1.13	1.56	1.36	0.06	0.07
Cluster 2	14	8.13	0.02	8.13	0.85	0.04	0.15	1.28	1.48	1.29	0.06	0.06
Cluster 2	15	10.84	0.02	10.84	0.96	0.04	0.16	1.62	1.55	1.31	0.05	0.06
Cluster 2	16	9.71	0.02	9.71	0.89	0.04	0.12	1.46	1.41	1.24	0.05	0.06
Cluster 2	17	9.44	0.02	9.44	0.86	0.05	0.14	1.38	1.47	1.26	0.05	0.06
Cluster 2	18	7.84	0.02	7.84	0.79	0.04	0.13	1.17	1.34	1.23	0.04	0.06
Cluster 2	19	6.55	0.02	6.55	0.63	0.04	0.10	1.02	1.37	1.21	0.05	0.06
Cluster 2	20	6.19	0.02	6.19	0.69	0.03	0.12	1.02	1.26	1.17	0.05	0.06
Cluster 2	21	5.69	0.01	5.69	0.64	0.04	0.10	0.95	1.21	1.07	0.05	0.06
Cluster 2	22	5.72	0.01	5.72	0.61	0.04	0.09	0.85	1.37	1.19	0.05	0.06
Cluster 2	23	5.56	0.01	5.56	0.58	0.03	0.09	0.89	1.33	1.13	0.05	0.06
Cluster 2	24	5.25	0.01	5.25	0.69	0.03	0.09	0.91	1.22	1.02	0.06	0.06
Cluster 2	25	5.20	0.02	5.20	0.56	0.03	0.08	0.87	1.29	1.15	0.05	0.06
Cluster 2	26	5.48	0.01	5.48	0.74	0.03	0.10	0.89	1.22	1.18	0.05	0.06

Cluster 2	27	5.37	0.01	5.37	0.55	0.03	0.10	0.76	1.22	1.10	0.05	0.06
Cluster 2	28	4.41	0.01	4.41	0.48	0.04	0.08	0.81	1.15	1.00	0.06	0.06
Cluster 2	29	4.86	0.01	4.86	0.46	0.03	0.07	0.87	1.15	1.03	0.05	0.06
Cluster 2	30	4.80	0.01	4.80	0.67	0.04	0.08	0.81	1.24	1.00	0.05	0.06
Cluster 2	31	4.71	0.01	4.71	0.54	0.03	0.07	0.78	1.13	1.02	0.05	0.05
Cluster 2	32	5.06	0.01	5.06	0.57	0.04	0.09	0.81	1.18	1.00	0.05	0.06
Cluster 2	33	4.98	0.01	4.98	0.52	0.03	0.08	0.81	1.19	0.98	0.05	0.05
Cluster 2	34	4.70	0.01	4.70	0.53	0.04	0.07	0.75	1.24	0.97	0.04	0.06
Cluster 2	35	4.53	0.01	4.53	0.51	0.04	0.07	0.70	1.08	0.95	0.04	0.05
Cluster 2	36	4.06	0.01	4.06	0.35	0.02	0.05	0.58	1.40	1.07	0.05	0.05
Cluster 2	37	4.85	0.01	4.85	0.49	0.03	0.07	0.75	1.21	0.97	0.05	0.05
Cluster 2	38	5.20	0.01	5.20	0.56	0.04	0.08	0.76	1.14	0.99	0.05	0.06
Cluster 2	39	4.52	0.01	4.52	0.52	0.04	0.07	0.76	1.19	0.89	0.05	0.06
Cluster 2	40	4.66	0.01	4.66	0.56	0.03	0.06	0.74	1.18	0.93	0.05	0.06
Cluster 2	41	4.30	0.01	4.30	0.44	0.03	0.05	0.67	1.15	0.90	0.05	0.05
Cluster 2	42	4.35	0.01	4.35	0.49	0.03	0.08	0.73	1.13	0.95	0.05	0.06
Cluster 2	43	4.29	0.01	4.29	0.46	0.03	0.05	0.72	1.00	0.86	0.05	0.05
Cluster 2	44	4.48	0.01	4.48	0.48	0.03	0.07	0.82	1.19	0.98	0.05	0.05
Cluster 2	45	4.64	0.01	4.64	0.49	0.03	0.06	0.85	1.09	0.95	0.05	0.05
Cluster 2	46	4.18	0.01	4.18	0.47	0.03	0.07	0.75	1.21	0.95	0.05	0.05
Cluster 2	47	4.12	0.01	4.12	0.49	0.03	0.07	0.72	1.14	0.97	0.05	0.06
Cluster 2	48	3.86	0.01	3.86	0.50	0.03	0.07	0.73	1.03	0.92	0.05	0.05
Cluster 2	49	3.84	0.01	3.84	0.48	0.03	0.06	0.70	1.26	0.98	0.05	0.06
Cluster 2	50	4.29	0.01	4.29	0.51	0.03	0.05	0.69	1.17	0.89	0.04	0.05
Cluster 2	51	4.80	0.01	4.80	0.46	0.03	0.07	0.74	1.18	0.91	0.05	0.05
Cluster 2	52	4.46	0.01	4.46	0.48	0.03	0.07	0.67	1.15	0.94	0.04	0.05
Cluster 2	53	4.09	0.01	4.09	0.40	0.03	0.06	0.68	1.06	0.98	0.04	0.05
Cluster 2	54	3.81	0.01	3.81	0.44	0.03	0.06	0.66	1.09	0.95	0.05	0.06
Cluster 2	55	3.80	0.01	3.80	0.44	0.03	0.06	0.67	1.06	0.87	0.05	0.05

Cluster 2	56	3.57	0.01	3.57	0.41	0.03	0.06	0.62	1.09	0.93	0.05	0.06
Cluster 2	57	3.55	0.01	3.55	0.36	0.02	0.05	0.61	1.09	0.89	0.04	0.05
Cluster 2	58	3.84	0.01	3.84	0.40	0.02	0.06	0.61	1.17	0.96	0.05	0.06
Cluster 2	59	3.82	0.01	3.82	0.47	0.03	0.06	0.63	1.25	0.94	0.05	0.06
Cluster 2	65	2.69	0.01	2.69	0.42	0.02	0.04	0.54	1.00	0.82	0.05	0.06
Cluster 2	66	3.50	0.01	3.50	0.37	0.03	0.05	0.57	1.03	0.90	0.05	0.06
Cluster 2	67	3.73	0.01	3.73	0.36	0.03	0.05	0.64	0.99	0.91	0.04	0.05
Cluster 2	68	2.78	0.01	2.78	0.45	0.03	0.04	0.56	1.05	0.89	0.05	0.06
Cluster 2	69	3.22	0.01	3.22	0.42	0.03	0.04	0.64	1.06	0.88	0.05	0.05
Cluster 2	70	3.31	0.01	3.31	0.33	0.02	0.05	0.54	0.94	0.84	0.04	0.06
Cluster 2	71	3.45	0.01	3.45	0.48	0.03	0.04	0.63	1.04	0.87	0.05	0.05
Cluster 2	72	3.49	0.01	3.49	0.40	0.03	0.05	0.65	1.09	0.85	0.05	0.05
Cluster 2	73	3.53	0.01	3.53	0.40	0.02	0.04	0.67	1.07	0.83	0.04	0.05
